# Synthesis and Evaluation of a 2,11‐Cembranoid‐Inspired Library

**DOI:** 10.1002/chem.201505093

**Published:** 2016-03-01

**Authors:** Amanda J Welford, John J. Caldwell, Manjuan Liu, Meirion Richards, Nathan Brown, Cara Lomas, Graham J. Tizzard, Mateusz B. Pitak, Simon J. Coles, Suzanne A. Eccles, Florence I. Raynaud, Ian Collins

**Affiliations:** ^1^ Division of Cancer TherapeuticsThe Institute of Cancer ResearchLondonSM2 5NGUK; ^2^ UK National Crystallography ServiceUniversity of SouthamptonSouthamptonSO17 1BJUK

**Keywords:** medicinal chemistry, medium-ring compounds, molecular diversity, oxygen heterocycles, synthesis design

## Abstract

The 2,11‐cembranoid family of natural products has been used as inspiration for the synthesis of a structurally simplified, functionally diverse library of octahydroisobenzofuran‐based compounds designed to augment a typical medicinal chemistry library screen. Ring‐closing metathesis, lactonisation and SmI_2_‐mediated methods were exemplified and applied to the installation of a third ring to mimic the nine‐membered ring of the 2,11‐cembranoids. The library was assessed for aqueous solubility and permeability, with a chemical‐space analysis performed for comparison to the family of cembranoid natural products and a sample set of a screening library. Preliminary investigations in cancer cells showed that the simpler scaffolds could recapitulate the reported anti‐migratory activity of the natural products.

## Introduction

**Figure 1:**
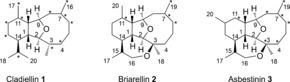
Cladiellin, briarellin and asbestinin core scaffolds. Carbon atoms bearing an asterisk are commonly oxygenated or unsaturated within the class.

The 2,11‐cembranoids belong to a structurally complex family of natural products isolated from the Octocorallia species, consisting of a polyoxygenated 2,11‐cyclised diterpenoid core scaffold.^1^ The structures are further subcategorised into cladiellins **1**, briarellins **2** and asbestinins **3** (Figure 1). Due to their structural complexity and broad ranging biological activities, the members of these structural class have been and continue to be attractive targets for total synthesis.^2^, ^3^, ^4^, ^5^, ^6^, ^7^, ^8^, ^9^, ^10^, ^11^, ^12^, ^13^, ^14^, ^15^, ^16^, ^17^ By necessity, such approaches require multi‐step routes, with a particular challenge being the introduction of the nine‐membered cyclic ether ring and the installation of the specific complex substitution patterns associated with each natural product. Methods used to form such a ring include Nozaki–Hiyama–Kishi coupling,^7^, ^8^, ^11^, ^14^ Claisen ring‐expansion,^6^ ring‐closing metathesis (RCM),^4^, ^9^, ^13^, ^15^ diazo ketone cyclisations^10^, ^16^ and oxidative ring‐expansion.^17^ A number of these total synthesis routes have been shown to afford access to more than one member of the natural product family^5^, ^7^, ^8^, ^10^, ^11^, ^13^, ^17^ allowing the preparation of small libraries of the natural products. The broad range of biological activities of the cembranoid family has been reviewed,^1^ and has been found to encompass anticancer, anti‐inflammatory, antiviral and antibacterial properties, amongst others.

Natural products have been a great source of inspiration in drug discovery, being used either in their own right as drugs or as the starting point for drug discovery projects.^18^ Libraries of natural products, natural‐product‐inspired or other structurally complex scaffolds have been shown to be a rich source of hits in phenotypic screens,^19^ which probe phenotypes arising from biological manipulation with small molecules in cells or small organisms (e.g., zebra fish embryos^20^). As biological assay screening tools they frequently occupy a complementary chemical space to commercially available synthetic libraries, providing novelty from both a structural and intellectual property perspective.^21^ Those natural products found to display interesting biological activities can, however, be stereochemically complex molecules, thus presenting significant synthetic challenges regarding analogue synthesis. Numerous approaches have been taken to overcome such challenges, in particular diversity‐oriented synthesis (DOS),^19^, ^22^ biology‐oriented synthesis (BIOS)^23^ and lead‐oriented synthesis.^24^ A principal challenge is to retain structural features of the natural products that differentiate them from chemical space explored by traditional small molecule screening libraries, and may be associated with the bioactivities of the natural products, while simplifying the structures to give easier synthetic access and control of the physicochemical properties.

Within the context of drug discovery, numerous metrics have been suggested as design aids in attempts to predict the likelihood of drug‐likeness,^25^ one of the most prevalent being Lipinski's rule‐of‐five.^26^ The central tenet of many of these metrics is to predict the ability of a compound to dissolve in aqueous media and to pass through a lipid membrane, both properties that may also be readily measured. To be useful for cell‐ or organism‐based biology, compounds must be sufficiently cell permeable and water soluble. It is therefore desirable to design new libraries around scaffolds where these properties are intrinsic.

Here, we present the synthesis of a 2,11‐cembranoid‐inspired small library of compounds, designed to retain the biological activities of the natural product class within structurally simplified library analogues, while displaying desirable lead‐like properties. The cembranoid class of natural products has demonstrated multiple biological activities in cells and parasites,^1^ making the core scaffold an ideal starting point to make libraries of cell‐permeable compounds. It was envisaged that the octahydroisobenzofuran core, with and without the third fused medium ring from the 2,11‐cembranoid motif, could be used as a privileged structure.^28^ It was anticipated that the library of compounds produced from such a privileged core would be able to recapitulate some of the biological activities observed in the natural product class, while reducing the synthetic complexity. The main synthetic challenge, therefore, was to find quick and concise routes to introduce substituent variations around the chosen core and methods to install a third ring system to mimic the nine‐membered ring of the 2,11‐cembranoids.

**Scheme 1:**
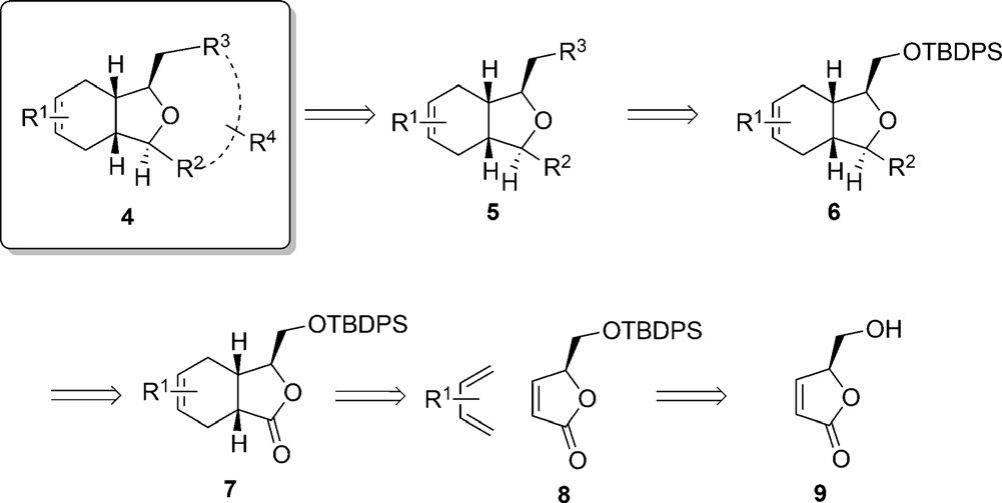
Library design and retrosynthetic analysis.

From the many successful synthetic strategies exemplified for the 2,11‐cembranoid natural products, we chose to explore a Diels–Alder reaction to construct the [6,5]‐bicyclic core of the library^6^, ^7^ We anticipated a rapid and flexible entry to the octahydroisobenzofuran scaffold through a Diels–Alder cyclisation, with the potential to vary the substituent patterns through choice of starting materials or manipulation of intermediates. Such an approach would provide a platform to investigate both the functionalization of the bicycle as well as the installation of a medium or larger ring equivalent to the nine‐membered ring of the natural products. The retrosynthetic approach is outlined in Scheme 1. From (*S*)‐5‐(hydroxymethyl)furan‐2(5 H)‐one (**9**), a Diels–Alder reaction with a range of dienes was envisaged to install the [6,5] core of compound **7**, which can be further manipulated to produce bicyclic analogues of the general structure **5**, or further cyclised to tricyclics such as compound **4**. Following the generation of the library, aqueous solubility, permeability and structural diversity in relation to in‐house screening libraries were assessed. In addition, the ability to recapitulate some of the biological activities of the more complex natural products with simpler structures from the library was demonstrated.

## Results and Discussion

**Scheme 2:**
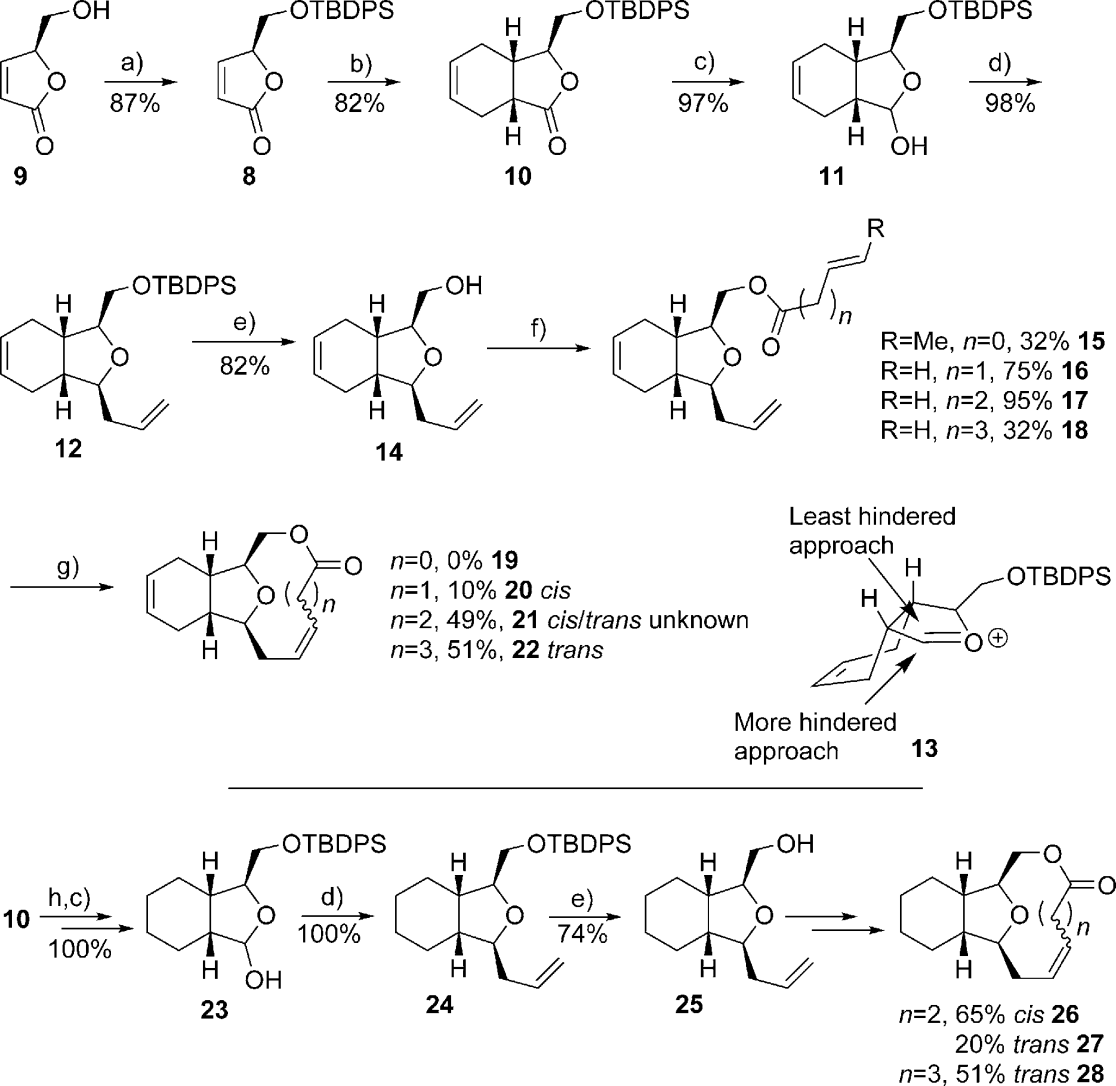
Reagents and conditions: a) TBDPSCl, imidazole, DMF, 0 °C–RT, 2.5 h; b) butadiene, TfN(AlMeCl)_2_, CH_2_Cl_2_, 60 °C; c) DIBAL‐H, CH_2_Cl_2_, −78 °C; d) allylTMS (TMS=trimethylsilyl), BF_3_ **⋅**OEt_2_, −78 °C; e) tetrabutylammonium fluoride (TBAF), 0 °C; f) acid chloride, pyridine or Et_3_N; g) Grubbs II, 0.0005 m CH_2_Cl_2_, RT; h) Pd/C (5 wt %), H_2_, EtOAc (Ac=acetyl), RT.

Protection of the commercially available alcohol **9** with *tert*‐butyl(chloro)diphenylsilane (TBDPSCl) gave the lactone **8** in 87 % yield; chiral HPLC confirmed the presence of a single enantiomer (Scheme 2). Reaction of the silyl ether **8** with butadiene in the presence of AlCl_3_ in dichloromethane at 55 °C in a sealed tube for one week under literature conditions^29^ gave the bicyclic compound **10** in 40 % yield. Although forming the desired product, yields were found to be highly variable. Screening of a number of Lewis acids found TfN(AlMeCl)_2_ 30 (Tf=triflate) to give a consistently high yield of around 70–80 % after a two day reaction. Furthermore, it was found possible to use a solution of 30 % butadiene in toluene as the diene source, avoiding the inconvenience and hazards associated with condensing gaseous butadiene. Reduction of the lactone **10** with diisobutylaluminum hydride (DIBAL‐H) gave the lactol **11** in high yield, which in turn was reacted with allyltrimethylsilane^31^, ^32^ to give compound **12** as a single enantiomer. The exclusive *cis* selectivity from reaction of the bicyclic lactol **11** contrasts with literature examples of monocyclic lactols, where the *trans* isomer predominates from an intermolecular allyltrimethylsilane addition.^30^ The observed difference can be rationalised by looking at a proposed conformation of the oxonium intermediate **13**, where the least hindered approach of the nucleophile leads to the *cis* product **12**.

Deprotection of silyl ether **12** using TBAF gave the alcohol **14** in 82 % yield, which was esterified to form esters of various chain lengths, that is, compounds **15**, **16**, **17** and **18**. Following considerable optimisation, ring‐closing metathesis (RCM) cyclisation using the Grubbs II catalyst at high dilution in dichloromethane gave the tricyclic compounds **20**, **21** and **22**. The nine‐membered lactone **19** could not be formed, whereas the ten‐membered lactone **20** was produced in low yield. The alkene conformation of compound **20** was presumed to be *cis* due to the strain of a *trans* double bond in such a ten‐membered lactone. The larger, less strained eleven‐ and twelve‐membered lactones were more readily constructed. The alkene conformation of compound **21** was undetermined due to overlapping NMR signals, whereas compound **22** was assigned to be *trans* by analogy to the crystal‐structure conformations of compounds **40** and **42** (see below). By using a similar reaction sequence, but reducing the alkene moiety of compound **10** earlier in the route, the cyclohexane analogues **26**, **27** and **28** were also formed. The structure of compound **26** was confirmed by X‐ray crystallography (see the Supporting Information).

**Scheme 3:**
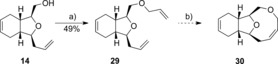
Reagents and conditions: a) NaH, allylbromide; b) Grubbs II, 0.0005 m, CH_2_Cl_2_, RT. 0 %.

As lactone **19** could not be formed, speculated to be due to the transoid nature of the ester moiety in compound **15** adding to the strain in forming a nine‐membered ring, synthesis of the ether analogue **30** was attempted (Scheme 3). Alkylation of alcohol **14** with allyl bromide gave the cyclisation precursor **29**. However, no RCM product could be observed under the above‐described optimised conditions, highlighting the difficulty in forming the strained nine‐membered ring. Previous RCM approaches to nine‐membered ring formation in the context of 2,11‐cembranoid synthesis have met with mixed success,^3^, ^4^, ^15^, ^33^ and appear highly substrate dependent.

**Scheme 4:**
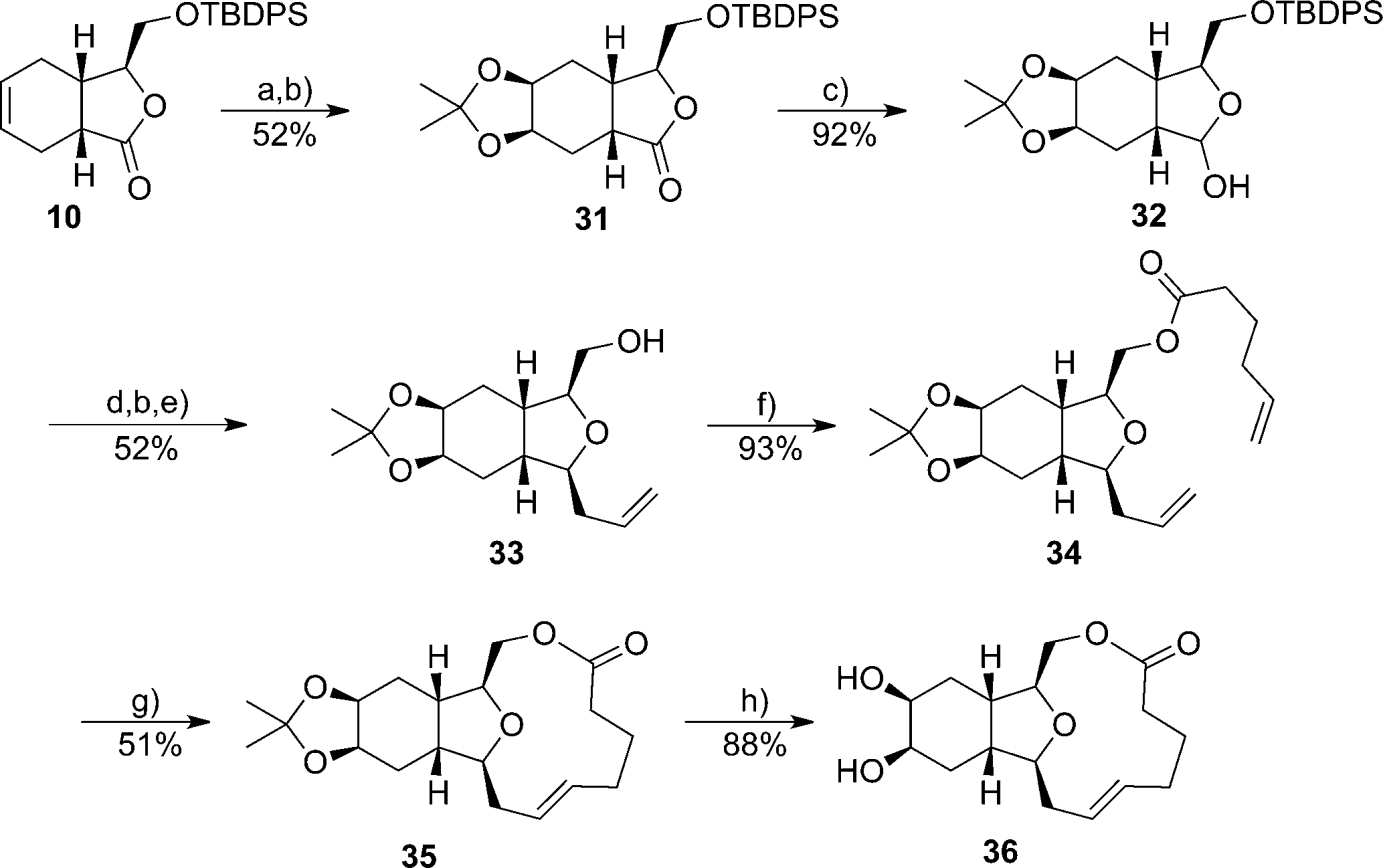
Reagents and conditions: a) OsEnCat (0.5 mol %), *N*‐methylmorpholine *N*‐oxide (NMO); b) 2,2‐dimethoxypropane, pyridinium tosylate (PPTS); c) DIBAL‐H, −78 °C; d) allylTMS, BF_3_ **⋅**OEt_2_, −78 °C; e) TBAF; f) hex‐5‐enoyl chloride, pyridine (py), CH_2_Cl_2_; g) Grubbs II, 0.0005 m CH_2_Cl_2_; h) HCl, THF.

By using the alkene functionality of lactone **10** as a point of derivatisation, dihydroxylation of the alkene from the least sterically crowded face using catalytic osmium tetroxide (OsEnCat) followed by dimethylacetal protection gave the tricyclic compound **31** in 52 % yield over two steps (Scheme 4). By using a similar sequence to that shown in Scheme 2, lactone **31** was reduced to lactol **32**, reacted with allyltrimethylsilane in the presence of BF_3_ **⋅**OEt_2_ and deprotected to give alcohol **33**. Esterification, ring‐closing metathesis and acetal deprotection with acid gave the diol **36**.

**Scheme 5:**
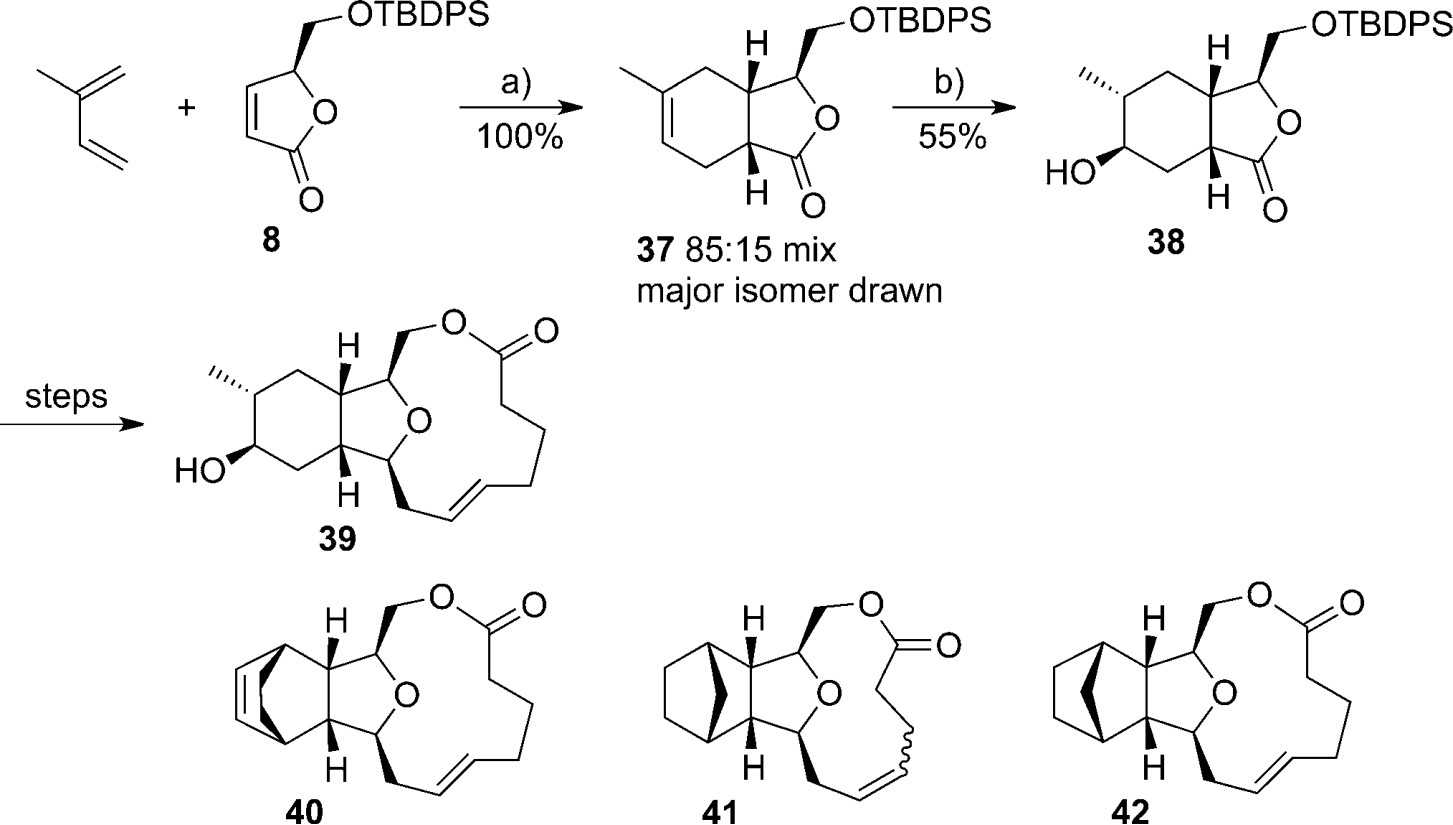
Reagents and conditions: a) TfN(AlMeCl)_2_, CH_2_Cl_2_, 60 °C, 100 %; b) BH_3_ **⋅**THF, then H_2_O_2_, H_2_O 55 %.

Analogue synthesis by changing the diene component was also demonstrated (Scheme 5). The use of 2‐methylbutadiene gave an 85:15 inseparable mix of methyl regioisomers (major isomer **37** shown). The major 5‐methyl component was isolated following oxidative hydroborylation to give the alcohol **38**. As above, the alcohol **38** was in turn converted to the lactone **39**. As further exemplification of diene variations in the Diels–Alder reaction, cyclohexa‐1,3‐diene and cyclopentadiene were used to make the tetracyclic compounds **40**, **41** and **42**. Confirmation of the structure of compounds **40** and **42** was achieved by X‐ray crystallography (see the Supporting Information). During the synthesis of compound **42**, it was found necessary to reduce the alkene derived from the diene due to undesired ring‐opening occurring in the subsequent RCM reaction, presumably as a consequence of release of inherent ring strain in the embedded cyclopentene ring that was not present in the cyclohexene analogue.

**Scheme 6:**
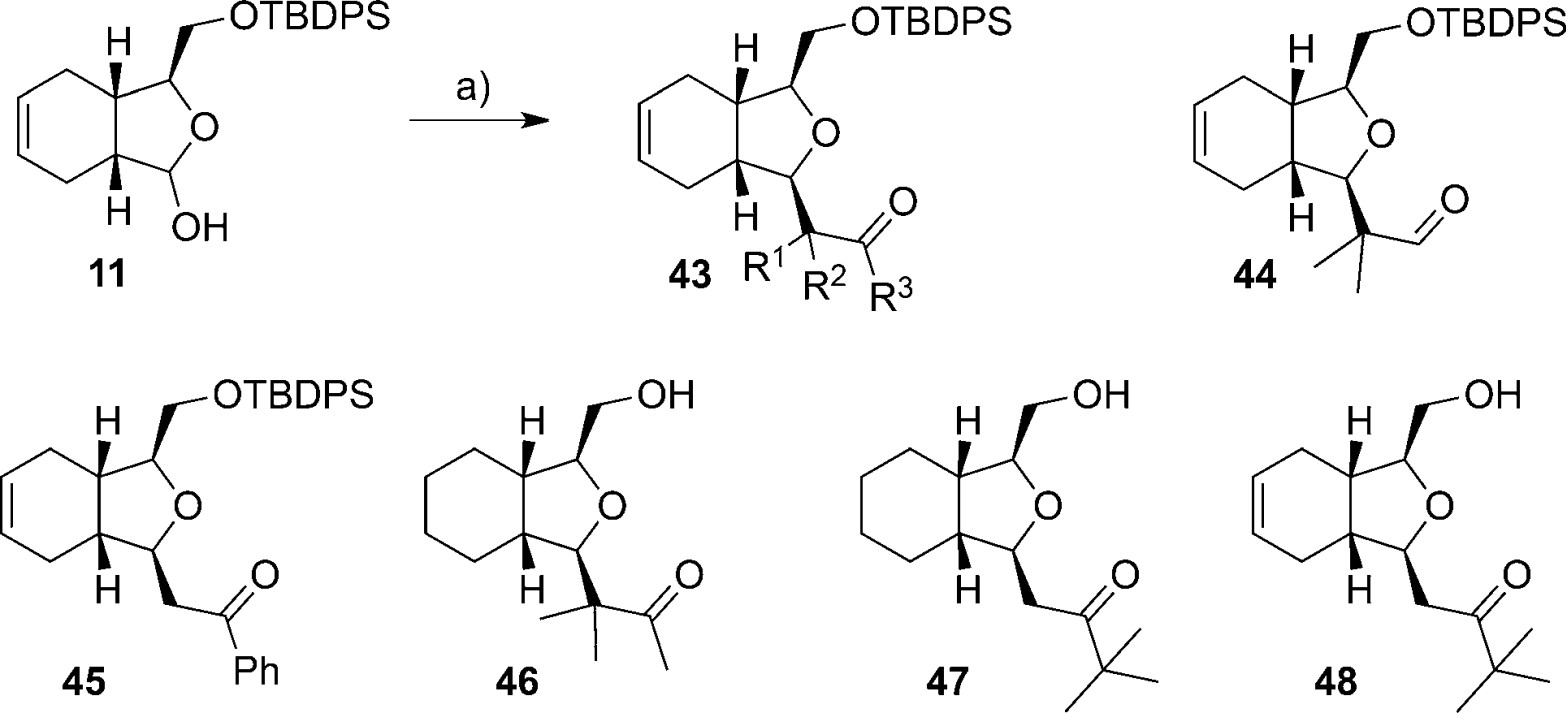
Reagents and conditions: a) silyl enol ether, BF_3_ **⋅**OEt_2_ (TMSOTf for compound **46**), CH_2_Cl_2_, −78 °C 36–97 %.

The Mukaiyama aldol reaction on lactol **11** provided a means of introducing further substitution and a handle for elaboration. To this end, adducts of the general structure **43** were formed as single enantiomers by reacting the hemiacetal **11** with a range of silyl enol ethers. As in the example of allyltrimethylsilane addition described above (Scheme 2), the *cis* products were observed exclusively or as the major component (85:15 in the formation of compound **48**). The silyl ethers **44** and **45** were obtained, as well as alcohols **46**, **47** and **48** following silyl deprotection (Scheme 6).

**Scheme 7:**
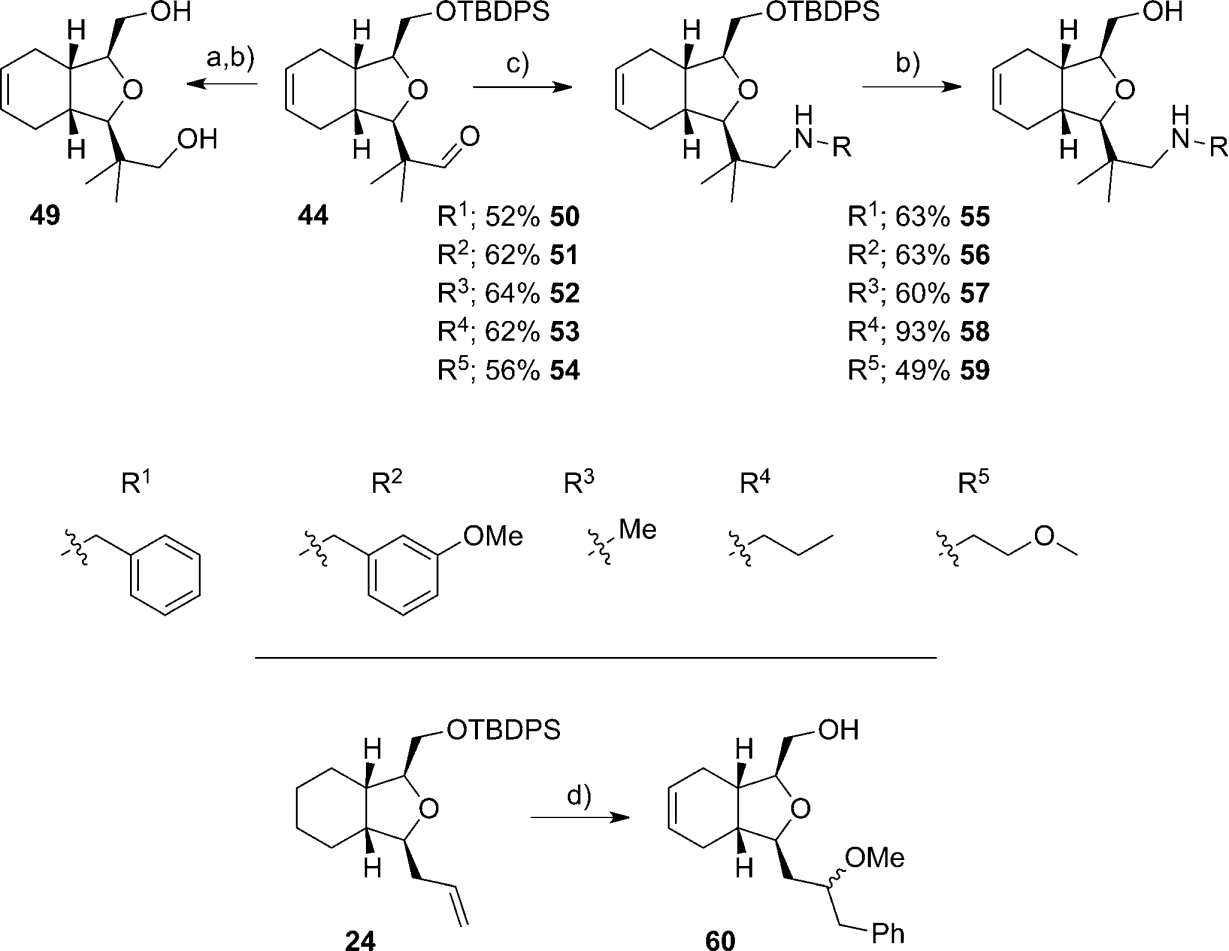
Reagents and Conditions: a) NaBH_4_, THF, MeOH 82 %; b) TBAF, THF, 41–93 %; c) amine, NaBH(OAc)_3_, 1,2‐dichloroethane (DCE), 52–64 %; d) PhSiMe_3_, [AuCl(PPh_3_)], selectfluor, MeOH, MeCN, then TBAF 29 %.

With the aldehyde **44** in hand, derivatisation was exemplified either by reduction to the diol **49**, or through reductive amination with the amines listed in Scheme 7. Deprotection of amines **50**–**54** with TBAF provided alcohols **55**–**59**, respectively, in good yield.

Modification of the terminal olefin in compound **24** was also exemplified. Oxyarylation^34^ by using a gold(I) catalyst in the presence of Selectfluor with phenyltrimethylsilane gave the ether **60** as an inseparable 4:1 mix of diastereomers (Scheme 7).

**Scheme 8:**
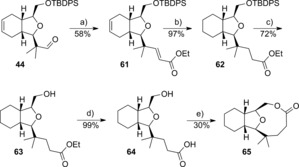
Reagents and Conditions: a) triethylphosphonoacetate, NaH, THF; b) Pd/C, EtOAc, H_2_; c) TBAF, THF; d) LiCl, H_2_O, THF; e) 2‐methyl‐6‐nitrobenzoic anhydride, DMAP, CH_2_Cl_2_.

**Figure 2:**
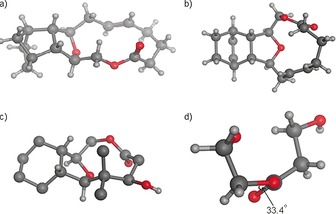
X‐ray crystal structure of compounds a) **42**, b) **40**, and c) **69**. d) Partial view of compound **69** along the O−C lactone single bond.

An alternative lactonisation approach to forming an oxygen‐containing nine‐membered ring system and thus providing library members with closer correspondence to the [6,5,9]‐tricyclic core of the natural product family was attempted from aldehyde **44**. A Horner–Wadsworth–Emmons reaction gave the α,β‐unsaturated ester **61** (Scheme 8). Reduction of both alkenes by hydrogenation over palladium gave the ester **62**. Deprotection of the silyl group with TBAF followed by ester hydrolysis gave the acid **64** in 71 % over two steps. Lactonisation was initially attempted by using the protocol of Yamaguchi et al.,^35^ through the intermediate 2,4,6‐trichlorobenzoyl anhydride. None of the desired lactone was observed in a number of attempts, with a dimeric species being the only isolated product seen. A more successful approach used conditions developed by Shiina et al.,^36^ whereby a solution of the acid **64** was added slowly by using a syringe pump over a time period of 15 h to a solution of 2‐methyl‐6‐nitrobenzoic anhydride and 4‐dimethylaminopyridine (DMAP) in dichloromethane. By using these conditions, lactone **65** was isolated in 30 % yield. Interestingly, an IR absorption for the carbonyl group was observed at $\tilde{\nu }$ =1730 cm^−1^, suggesting the carbonyl group to be significantly twisted out of plane from the lowest energy lactone conformation (compare with the crystal structure of compound **69**, Figure 2).

**Scheme 9:**
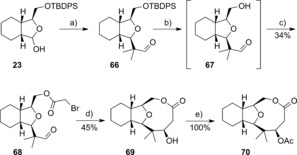
Reagents and conditions: a) BF_3_ **⋅**OEt_2_, trimethyl((2‐methylprop‐1‐en‐1‐yl)oxy)silane, CH_2_Cl_2_, −78 °C–RT; b) TBAF, THF; c) bromoacetyl bromide, Et_3_N, CH_2_Cl_2_, 34 % over three steps; d) SmI_2_, THF; e) Ac_2_O, DMAP, RT.

A SmI_2_‐mediated Reformatsky reaction approach was also found successful in the synthesis of a nine‐membered lactone. The Reformatsky reaction has been shown previously to give high yields and a controlled stereochemistry in the synthesis of a variety of natural product ring systems,^37^ and more pertinently in medium ring synthesis.^38^ Here, the alcohol **23** was subjected to the Mukaiyama aldol/TBAF deprotection sequence described above to give the unstable alcohol **67** (Scheme 9). Bromoacetyl bromide acylation followed by SmI_2_‐induced cyclisation gave the alcohol **69** in 45 % yield. In addition, the alcohol **69** could be further derivatised if required, such as to the acetate **70**. The structure and absolute stereochemistry of compound **69** was confirmed by X‐ray crystallography analysis (Figure 2). As with lactone **65**, an unusual IR carbonyl stretch at $\tilde{\nu }$ =1723 cm^−1^ was observed for compound **69** as a consequence of a 33° twist out of conjugation with the ester oxygen atom.

**Scheme 10:**
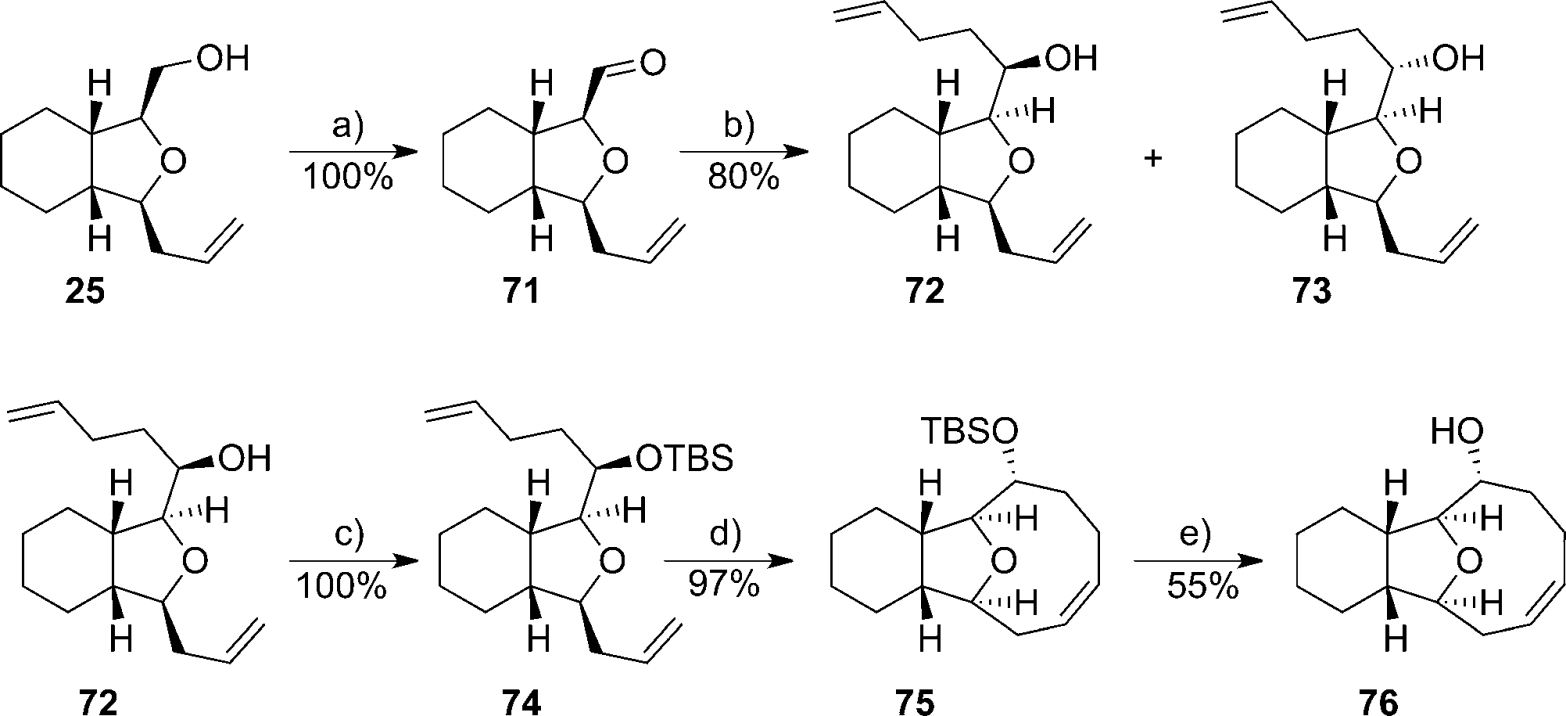
Reagents and conditions: a) Dess–Martin reagent, CH_2_Cl_2_; b) but‐3‐en‐1‐ylmagnesium bromide, THF; c) trimethylsilyl trifluoromethanesulfonate (TBSOTf), diisopropylethylamine (DIPEA), CH_2_Cl_2_; d) Grubbs II (10 mol %), CH_2_Cl_2_, heating to reflux; e) HCl, EtOH, Et_2_O.

**Figure 3:**
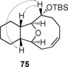
^1^H–^1^H NOESY correlations in the tricyclic compound **75**.

With different synthetic routes to nine‐membered lactones in hand, our focus returned to accessing nine‐membered ethers. Starting from alcohol **25**, synthesised as shown in Scheme 2, oxidation to the aldehyde **71** and subsequent reaction with but‐3‐en‐1‐ylmagnesium bromide gave alcohols **72** and **73** as a 5:3 separable mixture of isomers (Scheme 10). Following TBS protection, only the major isomer **72** was found to cyclise in the RCM reaction, giving the tricyclic compound **75** in 97 % yield. A NOESY correlation between both the [6,5] bridgehead hydrogen atoms and the siloxy‐substituted C−H hydrogen atom in compound **75** as highlighted in Figure 3 was used to confirm the sterochemistry of the Grignard addition. Deprotection with TBAF gave the alcohol **76** in 55 % yield.

**Figure 4:**
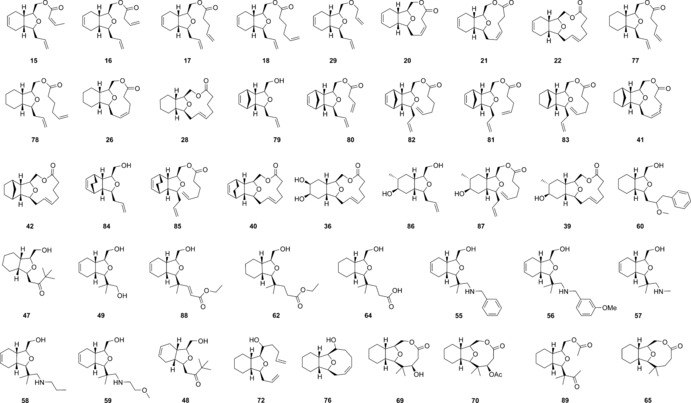
Cembranoid‐like library.

**Figure 5:**
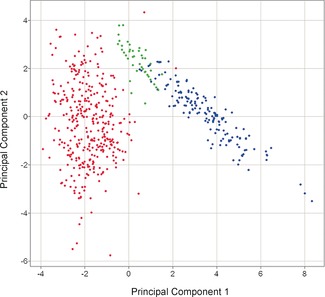
Principal component analysis (PCA) of the cembranoid‐inspired library (green, 44 structures), the 2,11‐cyclised cembranoid natural products (blue, 183 structures) and the ICR screening sample (red, 358 structures). Performed by using SIMCA‐P+^44^ and displayed by using Spotfire.^45^

The 2,11‐cembranoid based library of 44 compounds synthesised by using the routes and methods outlined above is shown in full in Figure 4. As the compounds were intended for the use in drug discovery screening programmes, an assessment of the library in terms of structural diversity, physicochemical properties and potential biological activity was undertaken. An analysis of the molecular properties of the compounds given in Figure 4 is compiled in Table 1. For comparison, the properties of 183 cembranoid natural products taken from reviews^1^, ^39^ and a random sampling set of 358 compounds taken from a substantially larger 75 000 screening library at the Institute of Cancer Research (ICR) are also listed. The ability of a random sampling set of a large screening library to represent the entirety has been demonstrated by Feher and Schmidt,^21^ and has been applied here. The ICR screening library itself was chosen by typical criteria for lead‐like/drug‐like libraries, including limits on predicted/calculated physicochemical parameters and choice of scaffolds, with a high frequency of heterocyclic aromatic and aliphatic scaffolds.^26^, ^27^ As a means of visually assessing the diversity between the groups, a principal component analysis (PCA)^41^ was performed. PCA has been used widely in chemoinformatics^42^ and particularly in the description of chemical space.^22^, ^43^ The analysis, carried out by using SIMCA‐P+^44^ and visualised by using Spotfire,^45^ showed (Figure 5) that the ICR screening compounds (red in Figure 5) cover a wide range of chemical space as would be expected from a well‐designed screening deck;^40^ however, little overlap exists with the cembranoid natural products (blue in Figure 5), which occupied a discrete and well‐defined region defined by the PCA. By contrast, the cembranoid‐inspired library as synthesised here (green in Figure 5) was found to occupy an area of chemical space between the natural products and the ICR screening collection, showing that the cembranoid‐inspired library begins to effectively bridge the chemical space between classical drug discovery screening compounds and the natural products.

**Table 1:** Arithmetic mean and standard deviation of the molecular properties of the cembranoid‐inspired library, the 2,11‐cyclised cembranoid natural products and a random sampling set from the Institute of Cancer Research screening collection.

	Cembranoid‐ inspired library	2,11‐Cyclised cembranoids	ICR screening collection sample
no. of compound	44	183	358
MW	275 (28)	441 (77)	332 (59)
*A*log *P*	2.6 (0.8)	3.1 (1.1)	2.6 (1.1)
no. of hydrogen acceptors	3.2 (0.7)	6.4 (2.0)	4.1 (1.4)
no. of hydrogen donors	0.7 (0.8)	1.0 (0.9)	1.1 (0.8)
no. of rings	2.6 (0.6)	3.4 (0.6)	2.9 (0.9)
no. of aromatic rings	0.07 (0.26)	0 (0)	2.0 (1.0)
no. of oxygen atoms	3.1 (0.7)	6.4 (2.0)	2.4 (1.4)
no. of hydrogen atoms	25 (2.9)	39 (5.9)	18 (5.3)
no. of stereoatoms	4.7 (0.9)	8.9 (1.5)	0.04 (0.33)
no. of bonds	21 (2.2)	34 (5.4)	25 (4.7)
no. of aromatic bonds	0.43 (1.6)	0 (0)	11 (5.2)
no. of rotatable bonds	4.8 (3.2)	5.9 (3.1)	4.5 (2.0)

The library prepared was assessed for both solubility and permeability, with representative results shown in Table 2. Due to a lack of UV chromophore and/or poor mass spectrometry ionisation, nephelometry^46^ was used to measure the solubility. PAMPA (parallel artificial membrane permeability assay)^47^ was used to assess the permeability. Generally, the compounds were found to have high aqueous solubility and permeability. Of the 44 compounds given in Figure 4, all were measured to have greater than 100 µm aqueous solubility, with 34 greater than 500 µm and 21 greater than 1000 µm. In regard to the 28 compounds that ionised sufficiently well to be detected by the PAMPA, 25 were found to have a high permeability, greater than 25×10^−6^ cms^−1^. For the specific example of compound **28** in addition to a good aqueous solubility and high PAMPA, the compound showed moderate permeability across a monolayer of Caco‐2 human intestinal cancer cells,^48^ (16×10^−6^ cms^−1^) with no evidence of transporter efflux.

**Table 2:** Solubility and permeability of selected representative compounds.

	Compound			
	**28**	**15**	**65**	**76**
solubility [μm]	550	500	550	500
permeability (Pe) [×10^−6^ cm^−1^]	52	25	>150	not detected^[a]^

Solubility measured by nephelometry. [a] Undetectable due to low ionisation.

**Figure 6:**
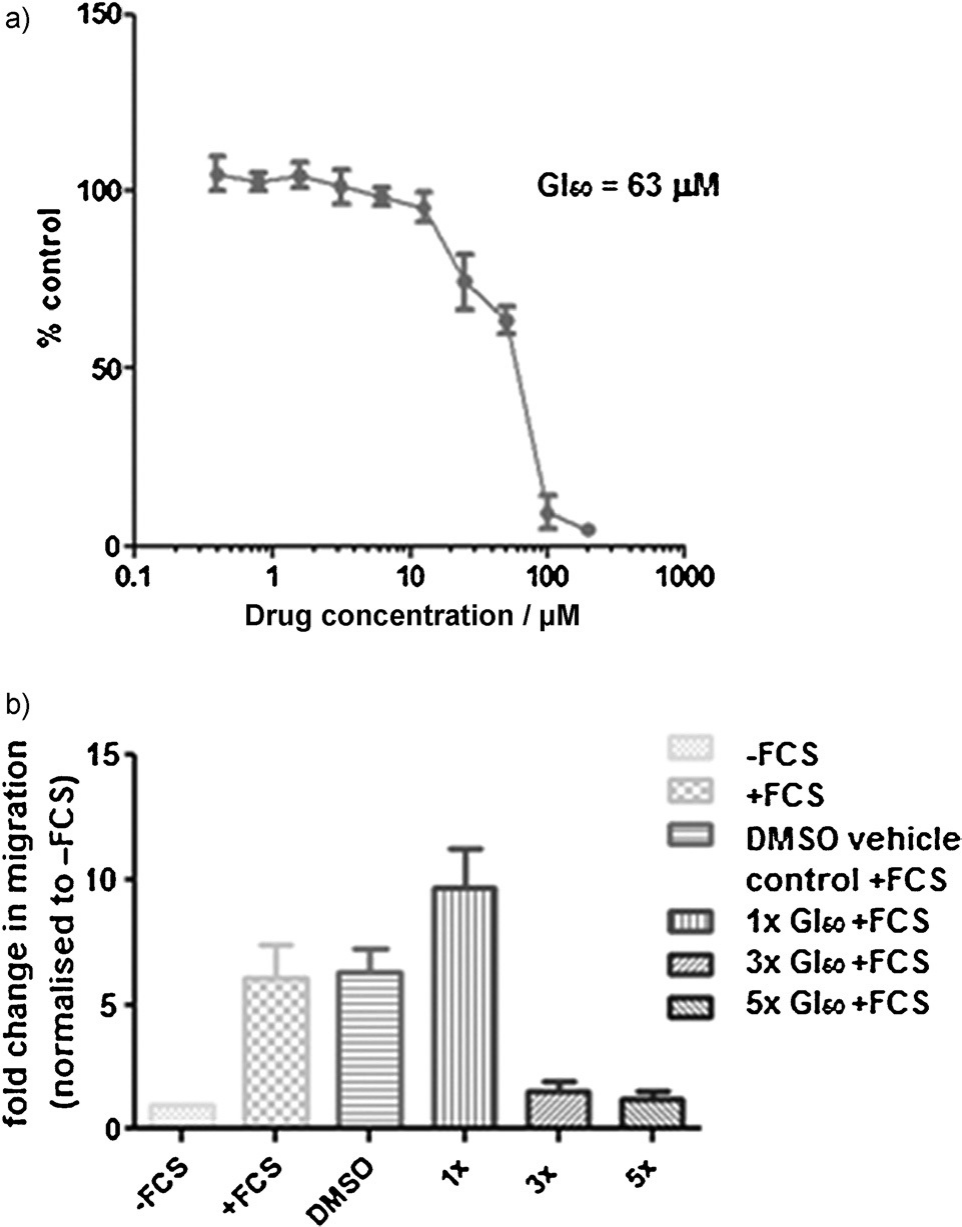
a) Concentration–response curve for the antiproliferative effect of compound **28** on PC3 cells in vitro 96 h assay. b) Compound **28** inhibiting the migration properties of PC3 TEM cancer cells. FCS=fetal calf serum. The *y* axis shows the fold‐changes in the cell migration normalised to the absence of FCS.

A selection of fifteen of the cembranoid‐inspired compounds was screened against a panel of 60 cancer cell lines at the National Cancer Institute screening panel (see the Supporting Information).^49^ This cell‐based assay aims to discover compounds with a strong cytostatic or cytotoxic growth inhibition over 48 h. Unfortunately, only low levels (up to 20 %) of cell growth inhibition were seen when screened at a single concentration of 10 µm. Of these, compound **28** gave 17 % inhibition of proliferation in the PC3 human prostate cancer cell line. A full dose response curve obtained for compound **28** (Figure 6) found a GI_50_ value of 63 µm. It is important to note that the cembranoid family itself is not overtly cytotoxic, with most examples screened to date ranging from single figure micromolar IC_50_ values to inactive in the cytotoxic assays used.^1^ One obvious exception to this is sclerophytin A, with an in vitro IC_50_ value of 3 nm in L1210 mouse lymphocytic leukemia cells,^51^ although the natural product showed no effect on proliferation in PC3 cells at 50 µm. Therefore, within this context, compound **28** displays typical cytotoxicity for the natural product family,^39^ with reduced structural and synthetic complexity.

Whereas cembranoid natural products may not be inherently cytotoxic, an appreciable number do display an ability to inhibit the migratory and invasive properties of cancer cells.^50^ The effect of compound **28** on a PC3‐based migratory assay was therefore determined. The tricyclic compound **28** at 3‐ and 5‐fold GI_50_ concentrations demonstrated a reduction to near basal levels of migration (Figure 6). Although less potent than a number of the 2,11‐cembranoid natural products (most active being sclerophytin A, with 85 % inhibition of migration at 50 µm),^50^ it does demonstrate that the anti‐migratory activity can be retained in a greatly simplified structural motif.

## Conclusion

The 2,11‐cembranoid class of natural products has served as a rich source of inspiration from both a synthetic and biological perspective. Here, we have developed a number of synthetic approaches to produce a library of structurally simplified cembranoid‐inspired derivatives. An assessment of the molecular properties of the octahydroisobenzofuran‐based library found them to be complementary to both the cembranoid natural products as well as a sample set of an in‐house medicinal chemistry screening collection, providing potential access to novel chemical space. A screen of the physicochemical properties found the library to have desirable properties, namely aqueous solubility and cell membrane permeability, essential to be useful in both biochemical and cell‐based assays. The illustrative synthetic routes developed could readily be expanded to more densely populate the chemical space occupied by the small library and the natural products, or indeed to follow up any hits from future screening. A preliminary screen of a sample set of the library found compound **28** to weakly inhibit the growth of PC3 cells, as well as having an inhibitory effect when screened in a cell migration assay. These data suggest that some of the bioactivities observed in cancer cells for the structurally complex 2,11‐cembranoid natural products can be recapitulated with much simpler scaffolds derived from the embedded octahydroisobenzofuran core.

## Experimental Section

All synthetic methods are included in the Supporting Information. CCDC 1010197 http://www.ccdc.cam.ac.uk/cgi‐bin/catreq.cgi (**26**), 1011320 http://www.ccdc.cam.ac.uk/cgi‐bin/catreq.cgi (**40**), 1435146 http://www.ccdc.cam.ac.uk/cgi‐bin/catreq.cgi (**42**) and 1435148 http://www.ccdc.cam.ac.uk/cgi‐bin/catreq.cgi (**69**) contain the supplementary crystallographic data. These data can be obtained free of charge from The Cambridge Crystallographic Data Centre.

## Supporting information

As a service to our authors and readers, this journal provides supporting information supplied by the authors. Such materials are peer reviewed and may be re‐organized for online delivery, but are not copy‐edited or typeset. Technical support issues arising from supporting information (other than missing files) should be addressed to the authors.

Supplementary
