# Supplementary material for: Synthesis and Evaluation of a 2,11‐Cembranoid‐Inspired Library
Source: Chemistry. 2016 Mar 1;22(16):5657–64. doi: 10.1002/chem.201505093 (PMC4869678; doi:10.1002/chem.201505093)

CHEMISTRY
A European Journal

Supporting Information

Synthesis and Evaluation of a 2,11-Cembranoid-Inspired Library

Amanda J Welford,[a] John J. Caldwell,*[a] Manjuan Liu,[a] Meirion Richards,[a] Nathan Brown,[a] Cara Lomas,[a] Graham J. Tizzard,[b] Mateusz B. Pitak,[b] Simon J. Coles,[b] Suzanne A. Eccles,[a] Florence I. Raynaud,[a] and Ian Collins[a]

chem_201505093_sm_miscellaneous_information.pdf

Contents

S2	General experimental
S5	Experimental procedures
S61	Assay methods
S62	PCA methodolodgy
S64	crystallography data
S68	NCI cytotoxicity screening compounds
S69	References
S70	^{1}H and ^{13}C NMR spectra

General experimental

All anhydrous solvents and reagents were obtained from commercial suppliers and used without further purification unless otherwise noted. Flash column chromatography was carried out on Merck silica gel 60 (0.040-0.063 mm).

All melting points were determined on a Stanford Research Systems MPA120 EZ-Melt melting point apparatus.

Analytical thin layer chromatography (TLC) was performed on pre-coated aluminium sheets of silica (60 F_{254}, Merck) and visualised by short-wave UV light, potassium permanganate vanillin or anisaldehyde dip. Infrared spectra were recorded on a Perkin Elmer Spectrum RX-1 or a Bruker Alpha-P FT-IR spectrometer. Optical rotations were recorded on a Bellingham and Stanley ADP440 Polarimeter.

^{1}H-nuclear magnetic resonance spectra were recorded at 500 MHz on Bruker Avance 500 spectrometers using an internal deuterium lock. Chemical shifts were measured in parts per million (ppm) relative to tetramethylsilane (δ = 0) using the following internal references for residual protons in the solvent: $CDCl_3$ (δ 7.26), CD_3OD (δ 3.32), pyridine-*d6* (δ 8.74) and acetone-*d6* (δ 2.05).

^{13}C-nuclear magnetic resonance spectra were recorded at 126 MHz on Bruker Avance 500 spectrometers using an internal deuterium lock. All chemical shift values were reported in ppm relative to tetramethylsilane (δ = 0). The following internal references were used: $CDCl_3$ (δ_C 77.0), CD_3OD (δ 49.0) and Pyridine-*d5* (δ 150.3).

LC-MS analyses were performed on a Micromass LCT / Water's Alliance 2795 HPLC system using the column, flow rates and solvent gradient specified below at a temperature of 22 ^{o}C.

UV Detector: Waters 2487 Dual λ Absorbance Detector (detecting at 254nm)

LCT Gradients:

Method A 6min Grad

Flow rate 1 mL/min; Phenomenex Gemini 3u C18 column (3cm x 4.6mm i.d), solvent system-aqueous (0.1% Formic Acid) and methanol

**Time / min**	**% MeOH in MP**
0	10
0.3	10
0.6	20
4.5	90
5.4	90
5.7	10
6.0	10

Method B 6min Non-Polar

Flow rate 1mL/min; Phenomenex Gemini 3u C18 column (3cm x 4.6mm i.d), solvent system aqueous (0.1% Formic Acid) & Methanol

Time / min	% MeOH in MP
0	10
0.3	10
0.6	20
3	90
5.4	90
5.7	10
6.0	10

Method C Fast4min

Flow rate 2 mL/min; Merck Chromolith SpeedROD RP-18e 50x4.6mm column, solvents A = MeOH, B = Aqueous (0.1% Formic Acid)

Time / min	A (%)	B(%)
0	10	90
2.25	90	10
3	90	10
3.3	10	90
3.5	10	90

Method D Fast4minLipophilic

Flow: 2 mL/min; Merck Chromolith SpeedROD RP-18e 50x4.6mm column, solvents A = MeOH, B = Aqueous (0.1% Formic Acid)

Time/min	A (%)	B (%)
0	10	90
1.75	90	10
3	90	10
3.3	10	90
3.5	10	90

Method E Fast4minLipophilic II

Flow: 2 mL/min; Merck Chromolith SpeedROD RP-18e 50x4.6mm column, solvents A = MeOH, B = Aqueous (0.1% Formic Acid)

Time/min	A (%)	B (%)
0	10	90
1	100	0
3	100	0

3.3	10	90
3.5	10	90

High resolution mass spectrometry data was collected using an Agilent 6210 Time Of Flight Mass Spectrometer with a Merck Chromolith SpeedROD RP-18e 50x4.6mm column and the solvent gradients as specified below-

Flow: 2 mL/min, Merck Chromolith SpeedROD RP-18e 50x4.6mm column, solvent A = MeOH, B = Aqueous (0.1% Formic Acid)

Time / min	A (%)	B (%)
0	10	90
2.5	90	10
3.5	90	10
3.8	10	90
4	10	90

Reference Masses:

Caffeine $[M+H]^+$ = 195.087652; Reserpine $[M+H]^+$ = 609.280657; (1H,1H,3H-tetrafluoropentoxy)phosphazene $[M+H]^+$ = 922.009798.

UV detection was at 254 nm and ionisation was positive ion electrospray. Molecular weight scan range was 50-1000. Samples were prepared as 1 mg/mL in methanol or DMSO with 3 µL injected on a partial loop fill.

Microanalysis was carried out by Stephen Boyer, London Metropolitan Elemental Analysis Service. (Stephen Boyer, School of Human Sciences, Science Centre, London Metropolitan University, 29 Hornsey Road, London N7 7DD)

Experimental procedures

(S)-5-((*tert*-Butyldiphenylsilyloxy)methyl)furan-2-(5*H*)one 8

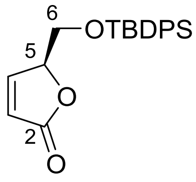

8

(S)-5-Hydroxymethylfuran-2-(5*H*)one **9** (1.00 g, 8.76 mmol) was added to a stirred solution of imidazole (660 mg, 9.76 mmol) in DMF (3 mL) and cooled to 0 °C. TBDPSCl (2.61 g, 2.47 mL, 9.76 mmol) was added dropwise and the reaction mixture was stirred for 30 min at 0 °C. The reaction was allowed to warm to RT and stirring continued for 2.5 h. The reaction mixture was quenched with sat. aq. $NaHCO_3$ (50 mL) and extracted with EtOAc (2 x 50 mL). The combined organic extracts were washed with water (50 mL) and brine (50 mL), then dried ($MgSO_4$) and concentrated to yield a clear oil which crystallised on standing. The product was recrystallised from hexane to yield **8** as white crystals (2.38 g, 87%). mp (79-81 °C) [lit.[1] 79-80 °C]; $[\alpha]^{23}_D$ -85 (c=1.50, $CHCl_3$) [lit.[2] -81.5]; IR (film) 2930, 2857, 1750 (CO), 1428, 1134 cm^{-1}; ^1H NMR (500 MHz, $CDCl_3$) δ 7.78-7.59 (4H, m, Ph), 7.53-7.34 (7H, m, Ph, H-4), 6.19 (1H, dd, *J*=2.0, 6.0, H-3), 5.09-5.07 (1H, m, H-5), 3.92 (1H, dd, *J*=5.0, 11.0, Ha-6), 3.88 (1H, dd, *J*=5.0, 11.0, Hb-6), 1.06 (9H, s, ^tBu); ^{13}C NMR (125 MHz, $CDCl_3$) δ 172.8 (C2), 154.0 (C4), 135.6 (2 x Ph), 135.5 (2 x Ph), 132.8 (^iPh), 132.5 (^iPh), 130.0 (2 x Ph), 127.9 (4 x Ph), 122.7 (C3), 83.2 (C5), 63.4 (C6), 26.7 (tBu), 19.2 (Si-C); LC-MS (ESI^+) *m/z* 375 [$M+Na^+$], R_t 5.43 min [method B]; HRMS [$M+Na^+$] calcd for $C_{21}H_{24}O_3SiNa$ 375.1387; found 375.1390; Found: C, 71.33; H, 6.88%; $C_{21}H_{24}O_3Si$ requires: C, 71.33; H, 6.86%.

(3*S*)-3-((*tert*-Butyldiphenylsilyloxy)methyl)-3a,4,7,7a-tetrahydroisobenzofuran-1-(3*H*)-one 10

Method A[1,3]

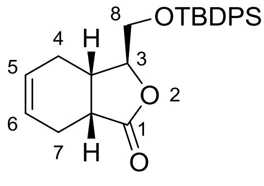

10

(S)-5-((*tert*-Butyldiphenylsilyloxy)methyl)furan-2-(5*H*)one **8** (500 mg, 1.42 mmol) was added to a stirring solution of $AlCl_3$ (50 mg, 0.375 mmol) in CH_2CL_2 (6 mL) in a pressure tube and cooled to 0 °C. A butadiene canister was cooled to 0 °C and butadiene (approx 3.0 mL, 25 equiv) poured into the pressure tube. The tube was sealed and heated to 55 °C for 6 d. The reaction was quenched in ice-cold sat. aq. $NaHCO_3$ (50 mL) and extracted with CH_2Cl_2 (25 mL x 2). The organic layers were separated, washed with brine (50 mL x 2) and dried over $MgSO_4$. The solvent was removed *in vacuo*. The crude mixture was purified by column chromatography on silica gel (15% EtOAc:hexane) to yield **10** as a clear oil (227 mg, 40%).

Method B

Carried out in triplicate- Trifluoromethanesulfonamide (465 mg, 3.125 mmol) was dissolved in 3.0 mL CH_2Cl_2 in a pressure tube. 1 M Dimethylaluminium chloride in hexane (6.25 mL, 6.250 mmol) was added slowly dropwise by syringe (ensuring no contact during addition with the vessel wall) under argon (gas evolution) and the solution stirred for 30 minutes. (*S*)-5-((*tert*-Butyldiphenylsilyloxy)methyl)furan-2-(5*H*)one **8** (1.00 g, 2.841 mmol) and butadiene (20% w/w in toluene, 9.5 mL, 28.4 mmol) were added, the tube sealed and heated to 60 °C for 2 d. The three reactions were combined for work-up; The vessels was cooled to 0 °C before opening and diluted with Et_2O (300 mL). The mixture was quenched cautiously by dropwise addition of 1M aqueous NaOH (200 mL). Gas was evolved. The aqueous layer was further extracted into Et_2O (60 mL x 2). The combined organics were washed with brine (100 mL) and dried over $MgSO_4$. The solvent was removed *in vacuo*. The crude mixture was purified by column chromatography on silica gel (10-20% EtOAc:hexane) to yield **10** as a clear oil (2.82 g, 82%). $[\alpha]^{20}_D$ +13.0 (c=1.00, $CHCl_3$) [lit.[1] +19.8]; IR (film) 3070, 3046, 2930, 2856, 1778 (CO), 1589 cm^{-1}; ^1H NMR (500 MHz, $CDCl_3$) δ 7.73-7.67 (4H, m, Ph), 7.50-7.39 (6H, m, Ph), 5.89-5.79 (2H, m, H-6, H-5), 4.17 (1H, dt, *J*=4.0, 4.0, H-3), 3.89 (1H, dd, *J*=4.0, 11.5, H-8), 3.79 (1H, dd, *J*=4.5, 11.5, H-8), 3.01 (1H, ddd, *J*=4.5, 8.5, 8.5, H-3a), 2.74-2.69 (1H, m, H-7a), 2.48-2.24 (3H, m, H-4, Ha-7), 1.97-1.90 (1H, m, Hb-7), 1.10 (9H, s, tBu); ^{13}C NMR (125 MHz, $CDCl_3$) δ 179.5 (C1), 135.6 (2 x Ph), 135.5 (2 x Ph), 132.9 (^{i}Ph), 132.6 (^{i}Ph), 129.9 (Ph), 127.9 (2x Ph), 126.4 (C5 or C6), 125.6 (C5 or C6), 84.8 (C3), 64.3 (C8), 37.4 (C7a), 34.1 (C3a), 26.8 (tBu), 25.5 (C7), 22.5 (C4), 19.1 (Si-C); LC-MS (ESI^+) *m/z* 429.18 [$M+Na^+$], R_t 3.08 [method A]; HRMS [$M+Na^+$] calcd for $C_{25}H_{30}O_3Na$ 429.1856; found 429.1866. Found: C, 73.97; H, 7.51%, $C_{25}H_{30}O_3$ requires: C, 73.85; H, 7.44%.

(3*S*,3a*S*,7a*R*)-3-((*tert*-Butyldiphenylsilyloxy)methyl)-1,3,3a,4,7,7a-hexahydroisobenzofuran-1-ol 11

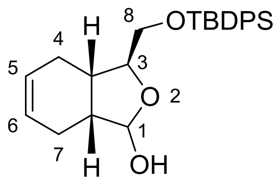

11

(3*S*)-3-((*tert*-Butyldiphenylsilyloxy)methyl)-3a,4,7,7a-tetrahydroisobenzofuran-1-(3*H*)one **10** (150 mg, 0.37 mmol) was dissolved in anhydrous CH_2Cl_2 (1 mL) and cooled to -78 °C under N_2. DIBAL-H (1.0 M in toluene, 0.40 mL, 0.40 mmol) was added dropwise. The reaction was stirred at -78 °C for 80 min then quenched with EtOAc (4 mL). The solution was warmed to RT and a sat. aq. solution of Rochelles salt (6 mL) was added and the mixture was stirred for 3 h. The aqueous layer was separated and extracted with CH_2CL_2 (3 x 5 mL). The organic layers were combined, dried over $MgSO_4$ and solvent removed *in vacuo* to yield **11** as a clear oil (145 mg, 97%). $[\alpha]^{23}_D$ -9.13 (c=2.43 CH_2Cl_2); IR (film) 3409, 3024, 2929, 2856, 1427 cm^{-1}; ^1H NMR (500 MHz, $CDCl_3$) δ 7.75-7.70 (4H, m, Ph), 7.51-7.39 (6H, m, Ph), 5.75-5.72 (1H, m, H-6), 5.64-5.58 (1H, m, H-5), 5.09 (1H, d, *J*=6.0, H-3), 3.87-3.82 (2H, m, H-1, Ha-8), 3.63 (1H, dd, *J*=3.0, 10.0, Hb-8), 2.90 (1H, d, *J*=6.0, OH), 2.74 (1H, dd, *J*=7.5, 15.0, H-7a),

2.31-2.16 (3H, m, H-3a, H-7, H-4), 1.88-1.79 (2H, m, H-7, H-4), 1.11 (9H, s, ^{t}Bu); ^{13}C NMR (125 MHz, $CDCl_3$) δ 135.7 (2 x Ph), 135.6 (2 x Ph), 133.0 (Ph), 132.9 (Ph), 129.8 (^{i}Ph), 129.7 (^{i}Ph), 127.7 (2 x Ph), 127.6 (2 x Ph), 125.0 (C5 or C6), 124.4 (C5 or C6), 103.1 (C3), 83.5 (C1), 64.6 (C8), 41.7 (C3a), 32.2 (C7a), 26.9 (tBu), 23.3 (C7 or C4), 22.8 (C7 or C4), 19.2 (Si-C); LC-MS (ESI$^+$) m/z 431 [M+Na$^+$], Rt 3.19 min [method B], HRMS [M+Na$^+$] calcd for $C_{25}H_{32}O_3SiNa$ 431.2013; found 431.2009.

(((1*S*,3*S*,3a*R*,7a*S*)-3-Allyl-1,3,3a,4,7,7a-hexahydroisobenzofuran-1-yl)methoxy)(*tert*-butyl)diphenylsilane 12

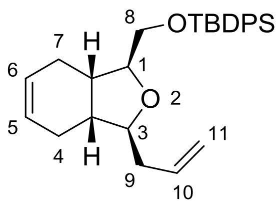

12

(3*S*,3a*S*,7a*R*)-3-((*tert*-Butyldiphenylsilyloxy)methyl)-1,3,3a,4,7,7a-hexahydroisobenzofuran-1-ol **11** (366 mg, 0.897 mmol) was dissolved in CH_2Cl_2 (4 mL) and cooled to -78 °C. $BF_3.OEt_2$ (0.34 mL, 2.70 mmol) was added by syringe and the solution was stirred for 5 min. Allyltrimethylsilane (0.43 mL, 2.70 mmol) was added by syringe and the reaction then warmed to RT. The reaction was stirred for 18 h before quenching with H_2O (9 mL), and extracting into CH_2Cl_2 (3x9 mL). The combined organic layers were dried over $MgSO_4$. The solvent was removed *in vacuo* to yield **12** as a clear oil (379 mg, 98%). $[\alpha]^{22}_{D}$ -13.1 (c=1.5, MeOH); IR (film) 3071, 3027, 2929, 2857, 1428 cm^{-1}; ^{1}H NMR (500 MHz, d6-Acetone) δ 7.77-7.74 (4H, m, Ph), 7.49-7.42 (6H, m, Ph), 5.89 (1H, dddd, *J*=7.0, 7.0, 10.0, 17.0, H-10), 5.75-5.69 (2H, m, H-5, H-6), 5.06-4.97 (2H, m, H-11), 3.79-3.65 (2H, m, H-8), 3.75-3.68 (1H, m, H-1 or H-3), 3.67-3.62 (1H, m, H-1 or H-3), 2.41-1.89 (8H, m, H-4, H-7, H-3a, H-7a, H-9), 1.05 (9H, s, ^{t}Bu); ^{13}C NMR (125 MHz, d6-Acetone) δ 136.7 (C10), 136.4 (2 x Ph), 136.4 (2 x Ph), 134.6 (^{i}Ph), 134.5 (^{i}Ph), 130.6 (Ph), 130.6 (Ph), 128.7 (2 x Ph), 128.7 (2 x Ph), 126.7 (C5 or C6), 126.6 (C5 or C6), 117.1 (C11), 85.1 (C3), 84.2 (C1), 66.8 (C8), 40.4 (C3a or C7a), 40.3 (C3a or C7a), 37.4 (C9), 27.5 (^{t}Bu), 25.6 (C4 or C7), 25.4 (C4 or C7), 19.9 (Si-C).

((1*S*,3*S*,3a*R*,7a*S*)-3-Allyl-1,3,3a,4,7,7a-hexahydroisobenzofuran-1-yl)methanol 14

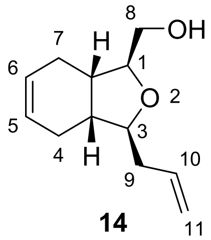

14

(((1*S*,3*S*,3a*R*,7a*S*)-3-Allyl-1,3,3a,4,7,7a-hexahydroisobenzofuran-1-yl)methoxy)(*tert*-butyl)diphenylsilane **12** (360 mg, 0.83 mmol) was dissolved in THF (4.2 mL). The solution was cooled to 0 °C under N_2, then TBAF (2.49 mL, 1.0 Mol in THF, 1.49 mmol) was added by syringe. The reaction was stirred for 2 h at 0 °C before quenching with Et_2O (30 mL). The reaction mixture was washed with H_2O (30 mL) and extracted into Et_2O (2 x 30 mL). The combined organic layers were dried with $MgSO_4$ and solvents were removed *in vacuo* to yield the crude product as a clear oil. This was purified by column chromatography on silica gel (10% EtOAc:hexane) to yield **14** as a clear oil (132 mg, 82%).

$[\alpha]^{22}_D$ +17.6 (c=2.60 CH_2Cl_2); IR (film) 3414, 3075, 3026, 2975, 2841, 1435 cm^{-1}; ^{1}H NMR (500 MHz, $CDCl_3$) δ 5.79 (1H, tdd, *J*=7.0, 10.0, 17.5, H-10), 5.78-5.73 (2H, m, H-5, H-6), 5.11-5.10 (2H, m, H-11), 3.82-3.75 (3H, m, Ha-8, H-1, H-3), 3.51 (1H, dd, *J*=6.5, 12.5, Hb-8), 2.39-2.28 (5H, m, Ha-9, H-7a, H-4a, Ha-7, Ha-4), 2.08 (1H, ddd, *J*=6.0, 6.5, 13.0, Hb-9), 1.97-1.93 (2H, Hb-7, Hb-4); ^{13}C NMR (125 MHz, $CDCl_3$) δ 134.9 (C10), 125.8 (C5 or C6), 125.5 (C6 or C5), 117.1 (C11), 83.9 (C1 or C3), 83.7 (C1 or C3), 63.7 (C8), 39.7 (C4a or C7a), 39.5 (C9), 35.7 (C4a or C7a), 25.0 (C4 or C7), 24.4 (C4 or C7); LC-MS (ESI$^+$) *m/z* 217 [M+Na$^+$], R_t 2.38 min [method D]; HRMS [M+Na$^+$] calcd for $C_{12}H_{18}O_3Na$ 217.1192; found 217.1197.

But-2'-enoic acid (1*S*, 3*S*, 3a*R*, 7a*S*)-allyl-1, 3, 3a, 4, 7, 7a-hexahydro-isobenzofuran-8-ylmethyl ester 15

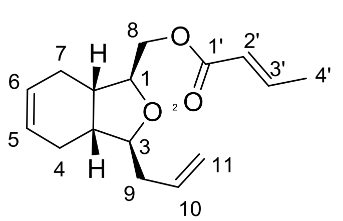

15

((1*S*,3*S*,3a*R*,7a*S*)-3-Allyl-1,3,3a,4,7,7a-hexahydroisobenzofuran-1-yl)methanol **14** (40 mg, 0.21 mmol) was dissolved in CH_2Cl_2 (1.0 mL). Pyridine (0.05 mL, 0.63 mmol) and *trans*-crotonyl chloride (26 µL, 0.27 mmol) were added and the reaction stirred at RT for 16 h. The reaction mixture was then diluted with CH_2Cl_2 (10 mL), and washed with H_2O (2 x 10 mL) and brine (10 mL). The combined aqueous layers were extracted with CH_2Cl_2 (2 x 10 mL). The organic layer was dried with $MgSO_4$ and solvent was removed *in vacuo*. The resultant crude oil was purified by column chromatography on silica gel (15% EtOAc:hexane) to yield **15** as a clear oil (15 mg, 32%). $[\alpha]^{19}_D$ -1.90 (c=0.8, CH_2Cl_2); IR (film) 3027, 2916, 2843, 1723 (CO), 1443 cm^{-1}; ^{1}H NMR (500 MHz, $CDCl_3$) δ 7.01 (1H, qd, *J*=7.0, 14.0, H-3'), 5.98-5.80 (2H, m, H-2', H-10), 5.79-5.65 (2H, m, H-5, H-6), 5.15-5.04 (2H, m, H-11), 4.25 (1H, dd, *J*=4.0, 11.5, Ha-8), 4.11 (1H, dd, *J*=6.0, 11.5, Hb-8), 3.88 (1H, ddd, *J*=4.5, 6.0, 6.0 , H-1), 3.76 (1H, ddd, *J*=6.0, 6.0, 6.0, H-3), 2.32-2.19 (5H, m, H-9, H-7a, H-7, H-4), 2.17-2.09 (1H, m, H-3a), 2.01-1.91 (2H, m, H-4, H-7), 1.89 (3H, dd, *J*=1.5, 7.0, H-4'); ^{13}C NMR (125 MHz, $CDCl_3$) δ 199.4 (C1'), 145.0 (C3'), 134.9 (C2'), 125.7 (C5 or C6), 125.3 (C5 or C6), 122.5 (C10), 117.0 (C11), 83.9 (C3), 81.3 (C1), 65.8 (C8), 39.5 (C9), 39.1 (C3a), 37.0 (C7a), 24.8 (C4 or C7), 24.5 (C4 or C7), 17.9 (C4'); LC-MS (ESI$^+$) *m/z* 285 [M+Na$^+$], R_t 2.83 min [method D]; HRMS [M+Na$^+$] calcd for $C_{16}H_{22}O_3Na$ 285.1461; found 285.1465.

((1*S*,3*S*,3a*R*,7a*S*)-3-Allyl-1,3,3a,4,7,7a-hexahydroisobenzofuran-1-yl)methyl but-3'-enoate 16

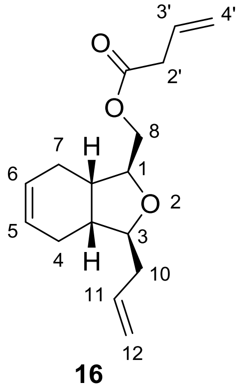

16

((1*S*,3*S*,3a*R*,7a*S*)-3-Allyl-1,3,3a,4,7,7a-hexahydroisobenzofuran-1-yl)methanol **14** (40 mg, 0.213 mmol) was dissolved in CH_2Cl_2 (1.0 mL). Triethylamine (90 µL, 0.630 mmol) and *trans*-crotonyl chloride (26 µL,

0.272 mmol) were added and the reaction left to stir at RT for 16 h. The reaction mixture was then diluted with CH_2Cl_2 (10 mL), and washed with H_2O (10 mL x 2) followed by brine (10 mL). The combined aqueous layers were extracted in to CH_2Cl_2 (10 mL x 2). The organic layer was dried with $MgSO_4$ and solvents were removed *in vacuo*. The resultant crude oil was purified by column chromatography on silica gel (15% EtOAc:hexane) to yield **16** as a clear oil (41 mg, 75%). $[\alpha]^{22}_D$ -6.06 (c=0.80 CH_2Cl_2); IR (film) 3353, 2906, 1752 (C=O), 1435, 1174 cm^{-1}; ^{1}H NMR (500 MHz, $CDCl_3$) δ 5.95 (1H, dddd, *J*=7.0, 7.0, 10.0, 17.0, H-3'), 5.85 (1H, dddd, *J*=7.0, 7.0, 10.0, 17.0, H-11), 5.73-5.69 (2H, m, H-5, H-6), 5.20-5.15 (2H, m H-12), 5.10-5.05 (2H, m, H-4'), 4.23 (1H, dd, *J*= 4.0, 11.5, Ha-8), 4.07 (1H, dd, *J*= 6.0, 11.5, Hb-8), 3.86 (1H, ddd, *J*=4.0, 6.0, 6.5, H-1), 3.76 (1H, ddd, *J*=6.0, 6.0, 6.0 H-3), 3.15 (2H, dd, *J*=1.5, 6.0, H-2'), 2.34-2.23 (5H, m, H-7a, H-10, H-4, H-7) 2.13-2.10 (1H, m, H-3a), 1.99-1.89 (2H, m, H-4, H-7); ^{13}C NMR (125 MHz, $CDCl_3$) 171.5 (C1'), 134.8 (C3'), 130.2 (C11), 125.7 (C6 or C5), 125.3 (C6 or C5), 118.6 (C4'), 117 (C12), 83.9 (C3), 81.2 (C1), 66.3 (C8), 39.5 (C4a or C7a), 39.0 (C2'), 39.0 (C4a or C7a), 37.0 (C10), 24.8 (C4), 24.6 (C7); LC-MS (ESI^+) *m/z* 285 [$M+Na^+$], R_t 2.80 [method D]; HRMS [$M+H^+$] calcd for $C_{16}H_{23}O_3Si$ 263.1641; found 263.1645.

Pent-4'enoic acid (1*S*, 3*S*, 3a*R*, 7a*S*)-allyl-1, 3, 3a, 4, 7, 7a-hexahydro-isobenzofuran-8-ylmethyl ester 17

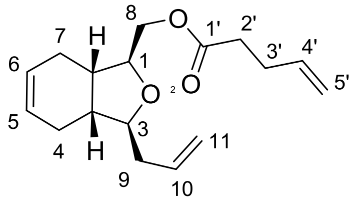

17

((1*S*,3*S*,3a*R*,7a*S*)-3-Allyl-1,3,3a,4,7,7a-hexahydroiso-benzofuran-1-yl)methanol **14** (34 mg, 0.175 mmol) was dissolved in CH_2Cl_2 (0.6 mL). Triethylamine (73 µL, 0.525 mmol) and pentenonyl chloride (25 µL, 0.223 mmol) was added and the reaction was stirred at RT for 16 h. The reaction mixture was then diluted with CH_2Cl_2 (10 mL), washed with H_2O (10 mL x 2) and brine (10 mL). The combined aqueous layers were extracted into CH_2Cl_2 (10 mL x 2). The organic layer was dried with $MgSO_4$ and the solvent was removed *in vacuo*. The resultant crude oil was purified by column chromatography on silica gel (15% EtOAc:hexane) to yield **17** as a clear oil (45 mg, 95%). $[\alpha]^{22}_D$ -7.84 (c=0.30, CH_2Cl_2); IR (film) 2923, 2919, 2854, 1738, 1440 cm^{-1}; ^{1}H NMR (500 MHz, $CDCl_3$) δ 5.91-5.77 (2H, m, H-10, H-4'), 5.75-5.68 (2H, m, H-5, H-6), 5.14-4.98 (4H, m, H-5', H-11), 4.21 (1H, dd, *J*=4.0, 11.5, Ha-8), 4.03 (1H, dd, *J*=6.0, 6.0, 6.0, Hb-8), 3.85 (1H, ddd, *J*=4.0, 6.5, 6.5, H-1), 3.76 (1H, ddd, *J*=6.0, 6.0, H-3), 2.52-2.35 (4H, m, H-2', H-3'), 2.35-2.16 (5H, m, H-9, H-7a, H-4, H-7), 2.12-2.16 (1H, m, H-3a), 2.01-1.88 (2H, m, H-4, H-7); ^{13}C NMR (125 MHz, $CDCl_3$) δ 173.0 (C1'), 136.6 (C10 or C4'), 134.8 (C10 or C4'), 125.7 (C5 or C6), 125.3 (C5 or C6), 117.1 (C11), 115.5 (C5'), 83.8 (C3), 81.2 (C1), 66.1 (C8), 39.5 (C9), 38.9 (C7a or C3a) 36.9 (C3a or C7a), 33.5 (C3'), 28.1 (C2), 24.8 (C7 or C4), 24.5 (C4 or C7); LC-MS (ESI^+) *m/z* 299 [$M+Na^+$], R_t 2.97 min [method D]; HRMS [$M+Na^+$] calcd for $C_{17}H_{24}O_3Na$ 299.1618; found 299.1619.

((1S,3S,3aR,7aS)-3-Allyl-1,3,3a,4,7,7a-hexahydroisobenzofuran-1-yl)methyl hex-5-enoate 18

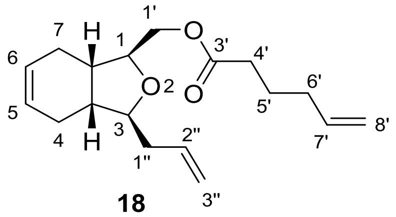

18

((1*S*,3*S*,3a*R*,7a*S*)-3-Allyl-1,3,3a,4,7,7a-hexahydroisobenzofuran-1-yl)methanol **14** (40 mg, 0.206 mmol) was dissolved in pyridine (1.0 mL). Hex-5-enoyl chloride (35 mg, 0.238 mmol) was added and the reaction stirred at RT for 16 h. The reaction mixture was then diluted with CH_2Cl_2 (10 mL) and solvent removed *in vacuo* to yield a brown residue. The residue was dissolved in CH_2Cl_2 (10 mL), then washed with H_2O (10 mL) and brine (10 mL). The combined aqueous layers were extracted with CH_2Cl_2 (2 x 10 mL). The organic layer was dried with $MgSO_4$ and solvent was removed *in vacuo*. The resultant crude oil was purified by column chromatography on silica gel (5% EtOAc:hexane) to yield **18** as a clear oil (20 mg, 32%). $[\alpha]^{19}_{D}$ -1.9 (c=0.8 CH_2Cl_2); IR (film) 1731 (CO), 1459 cm^{-1}; ^{1}H NMR (500 MHz, $CDCl_3$) δ 5.91-5.67 (4H, m, H-5, H-6, H-2'', H-7'), 5.15-4.96 (4H, m, H-3'', H-8'), 4.21 (1H, dd, *J*=4.0, 11.5, Ha-1'), 4.03 (1H, dd, *J*=6.0, 11.5, Hb-1'), 3.86 (1H, ddd, *J*=4.0, 6.0, 6.0, H-1), 3.76 (1H, ddd, *J*=6.0, 6.0, 6.0, H-3), 2.36 (2H, t, *J*=7.5, H-4'), 2.33-2.17 (5H, m, H-7, H-4, H-6', H-3a or H-7a), 2.15-2.07 (2H, m, H-1'', H-3a or H-7a), 2.02-1.87 (2H, m, H-7, H-4), 1.75 (2H, tt, *J*=7.5, 7.5, H-5'); ^{13}C NMR (125 MHz, $CDCl_3$) δ 173.6 (C3'), 137.7 (C2'' or C7'), 134.8 (C2'' or C7'), 125.7 (C5 or C6), 125.3 (C5 or C6), 117.5 (C8'), 115.4 (C3''), 83.9 (C1), 81.2 (C3), 66.0 (C1'), 39.5 (C6'), 39.0 (C3a or C7a), 36.9 (C3a or C7a), 33.5 (C4'), 33.1 (C1''), 24.8 (C4 or C7), 24.5 (C4 or C7), 24.0 (C5'); LC-MS (ESI$^+$) *m/z* 313 [M+Na$^+$], R_t 2.55 min [method D]; HRMS [M+Na$^+$] calcd for $C_{18}H_{26}O_3Na$ 313.1774; found 313.1775.

(*Z*)-(1*S*, 9*S*, 10*R*, 15*S*)-3, 16-Dioxa-tricyclo[7.6.1.0 ^{10,15}]hexadeca-6,12-diene-4-one 20

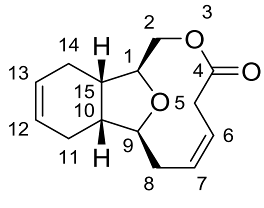

20

((1*S*,3*S*,3a*R*,7a*S*)-3-Allyl-1,3,3a,4,7,7a-hexahydroisobenzofuran-1-yl)methylbut-3'-enoate **16** (13 mg, 0.05 mmol) was dissolved in CH_2Cl_2 (100 mL, 0.0005 M). Argon was bubbled though the solution for 5 min. Grubbs II (4 mg, 0.005 mmol, 5 mol%) was added under argon and the reaction stirred for 16 h. DMSO (~10 µL) was added and the solution stirred overnight. The solvents were removed *in vacuo* and the resultant mixture purified by column chromatography on silica gel (15% EtOAc:hexane) to yield **20** as a white crystalline solid (2 mg, 10%). mp (79-81 °C); $[\alpha]^{22}_{D}$ +132.0 (c=0.50 CH_2Cl_2); IR (film) 3020, 2952, 2889, 1731 cm^{-1}; ^{1}H NMR (500 MHz, $CDCl_3$) δ 5.96-5.85 (1H, m, H-7), 5.76 (1H, ddd, *J*=2.5, 5.0, 10.0, H-12), 5.74-5.67 (1H, m, H-6), 5.67-5.62 (1H, m, H-13), 5.13 (1H, dd, *J*=3.5, 11.5, Ha-2), 3.89 (1H, dd, *J*=2.5, 11.5, H-9), 3.78 (1H, dd, *J*=3.5, 11.5, H-1), 3.59 (1H, dd, *J*=11.5, Hb-2), 3.23 (1H, ddd, *J*=2.0, 7.5, 11.5, Ha-5), 2.92 (1H, dd, *J*=12.0,

9.0, Hb-5), 2.82-2.68 (2H, m, H-15, Ha-8), 2.45-2.33 (1H, m, Ha-14), 2.33-2.20 (1H, m, Ha-11) 2.10-2.06 (1H, m, H-10), 2.05-1.88 (3H, m, Hb-8, Hb-11, Hb-14); ^{13}C NMR (125 MHz, $CDCl_3$) *δ* 172.4 (C4), 131.8 (C13), 125.6 (C6), 124.3 (C12), 123.4 (C7), 85.0 (C9), 80.7 (C1), 61.1 (C2), 39.4 (C10), 34.5 (C15), 34.4 (C8), 33.5 (C5), 26.1 (C11), 22.6 (C14); LC-MS (ESI$^+$) *m/z* 257 [M+Na$^+$], R_t 2.43 min [method D]; HRMS [M+H$^+$] calcd for $C_{14}H_{18}O_3H$ 235.1329; found 235.1327.

(*Z*)-(1*S*,10*S*,11*R*,16*S*)-3,17-Dioxa-tricyclo[8.6.1.0^{11,16}]heptadeca-7,13-diene-4-one 21

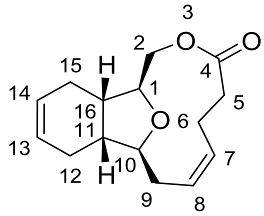

21

Pent-4′-enoic acid(1S,3S,3aR,7aS)-allyl-1,3,3a,4,7,7a-hexahydroisobenzofuran-8-ylmethyl ester **17** (23 mg, 0.083 mmol) was dissolved in CH_2Cl_2 (166 mL, 0.0005 M). Argon was bubbled though the solution for 5 min. Grubbs II (3.5 mg, 0.004 mmol, 5 mol%) was added under argon and the reaction mixture was stirred for 16 h. DMSO (~10 µL) was added and the solution was stirred overnight. The solvents were removed *in vacuo* and the resultant mixture purified by column chromatography on silica gel (15% EtOAc:hexane) to yield **21** as a white crystalline solid (10 mg, 49%). mp (81-86 °C); $[\alpha]^{22}_D$ -114.9 (c=0.10, CH_2Cl_2); IR (film) 3071, 2930, 2855, 1738, 1428 cm^{-1}; ^{1}H NMR (500 MHz, $CDCl_3$) *δ* 5.77-5.69 (1H, m, CH), 5.68-5.60 (1H, m, CH), 5.58-5.49 (2H, m, CH), 5.17 (1H, d, *J*=11.5, Ha-2), 3.97-3.88 (1H, dd, *J*=3.0, 11.5, H-10), 3.80 (1H, dd, *J*=3.0, 11.0, H-1) 3.60-3.47 (1H, d, *J*=12.0, Hb-2), 3.02-2.79 (1H, m, CH_2), 2.78-2.53 (3H, m, H-11, CH_2), 2.45-2.34 (2H, m, CH_2), 2.33-2.22 (1H, m, CH_2), 2.12-1.99 (3H, m, H-16, CH_2), 1.98-1.81 (2H, m, CH_2); ^{13}C NMR (125 MHz, $CDCl_3$) 172.1 (C4), 128.5 (CH), 128.0 (CH), 125.8 (CH), 123.7 (CH), 83.4 (C10), 79.4 (C1), 59.4 (C2), 39.7 (C16), 35.2 (CH_2), 34.1 (CH_2), 33.9 (C11), 26.1 (CH_2), 23.0 (CH_2), 22.6 (CH_2); LC-MS (ESI$^+$) *m/z* 271 [M+Na$^+$], R_t 2.55 min [method D]; HRMS [M+Na$^+$] calcd for $C_{15}H_{20}O_3Na$ 271.1310; found 271.1301.

(Z)-(1*S*,11*S*,12*R*,17*S*)-3,18-Dioxatricyclo[9.6.1.0^{12,17}]octadeca-8,14-dien-4-one 22

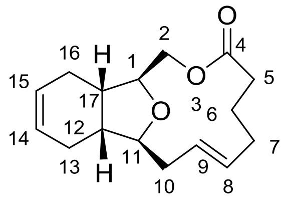

22

((1S,3S,3aR,7aS)-3-Allyl-1,3,3a,4,7,7a-hexahydroisobenzofuran-1-yl)methyl hex-5-enoate **18** (9 mg, 0.030 mmol) was dissolved in CH_2Cl_2 (63 mL, 0.0005 M). Argon was bubbled though the solution for 5 min. Grubbs II (3 mg, 0.003 mmol, 10 mol%) was added under argon and the reaction mixture was stirred for 16 h. DMSO (10 µL) was added and the solution was stirred overnight. The solvents were removed *in vacuo* and the resultant mixture purified by column chromatography on silica gel (10% EtOAc:hexane) to yield **22** as a white crystalline solid (4 mg, 51%). mp (122-126 ˚C); $[\alpha]^{19}_D$ -5.0 (c=1.0 CH_2Cl_2); IR (film) 3030, 2938, 2909, 1732 (CO), 1439 cm^{-1}; ^{1}H NMR (500 MHz, $CDCl_3$) *δ* 5.81-5.70 (2H, m, CH), 5.48-5.35 (2H, m, CH), 5.20 (1H, dd, *J*=3.0, 12.0, Ha-2),

3.78 (1H, ddd, *J*=3.0, 3.0, 6.0, H-1), 3.75 (1H, ddd, *J*=1.5, 2.0, 5.0, H-11), 3.51 (1H, dd, *J*=3.0, 12.0, Hb-2), 2.48-2.22 (7H, m, CH_2), 2.18-2.01 (3H, m, CH_2), 1.97-1.84 (3H, m, CH_2), 1.73-1.65 (1H, m, CH_2); ^{13}C NMR (125 MHz, $CDCl_3$) δ 173.8 (C4), 133.8 (CH), 126.7 (CH), 125.7 (CH), 125.5 (CH), 82.9 (C11 or C1), 82.8 (C11 or C1), 62.6 (C2), 37.4 (C12 or C17), 37.2 (C12 or C17), 34.4 (CH_2), 34.1 (CH_2), 33.6 (CH_2), 25.6 (CH_2), 24.4 (CH_2), 22.5 (CH_2); LC-MS (ESI$^+$) *m/z* 285 [M+Na$^+$], R_t 2.33 min [method D]; HRMS [M+Na$^+$] calcd for $C_{16}H_{22}O_3Na$ 285.1462; found 285.1465.

(3*S*,3a*S*,7a*R*)-3-((*tert*-Butyldiphenylsilyloxy)methyl)octahydroisobenzofuran-1-ol 23

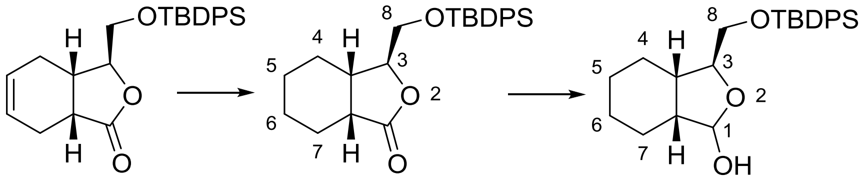

10 23a 23

(3*S*)-3-((*tert*-Butyldiphenylsilyloxy)-methyl)-3a,4,7,7a-tetrahydroisobenzofuran-1(3*H*)-one **10** (317 mg, 0.781 mmol) was dissolved in EtOAc (8 mL) and Pd/C (16 mg, 5% w/w Pd) was added. The solution was stirred overnight under H_2 (1 atm). The solution was filtered through silica eluted with EtOAc. The solvents were removed *in vacuo* to yield **23a** as a white crystalline solid (318 mg, 100%). mp (95-98 °C); $[\alpha]^{23}_D$ 6.1 (c=1.00 CH_2Cl_2); IR (film) 3070, 2998, 2931, 2856, 1774 (CO) cm^{-1}; ^1H NMR (500 MHz, $CDCl_3$) δ 7.69-7.63 (4H, m, Ph), 7.43-7.37 (6H, m, Ph), 4.13 (1H, dd, *J*=7.0, 7.0, H-3), 3.83 (1H, dd, *J*=4.5, 11.5, Ha-8), 3.76 (1H, dd, *J*=4.5, 11.5, Hb-8), 2.84 (1H, dd, *J*=7.0, 11.5, H-7a), 2.41-2.50 (1H, m, H-3a), 2.05-1.94 (1H, m, Ha-7), 1.85-1.74 (1H, m, Ha-4), 1.71-1.48 (4H, m, H-5, H-6), 1.38-1.21 (2H, m, Hb-4, Hb-7), 1.06 (9H, s, ^tBu); ^{13}C NMR (125 MHz, $CDCl_3$) δ 178.6 (C1), 135.6 (2 x Ph), 135.6 (2 x Ph), 132.9 (^iPh), 132.7 (^iPh), 129.9 (2 x Ph), 127.8 (4 x Ph), 83.1 (C3), 64.1 (C8), 38.7 (C7a), 36.6 (C3a), 27.7 (C4), 26.8 (^tBu), 23.2 (C5), 23.1 (C6), 22.8 (C7), 19.2 (Si-C); LC-MS (ESI$^+$) *m/z* 431 [M+Na$^+$], R_t 3.16 min [method D]; HRMS [M+Na$^+$] calcd for $C_{25}H_{32}O_3SiNa$ 431.2012; found 431.2020.

(3*S*,3a*S*,7a*R*)-3-((*tert*-Butyldiphenylsilyloxy)methyl)hexahydroiso-benzofuran-1-(3*H*)one **23a** (240 mg, 0.584 mmol) was dissolved in anhydrous CH_2Cl_2 (4.4 mL) and cooled to 78 °C under N_2. DIBAL-H (1.0 Mol in toluene, 0.97 mL, 0.97 mmol) was added dropwise. The reaction was stirred at 78 °C for 2 h then quenched with EtOAc (10 mL). The solution was warmed to RT and a sat. aq. solution of Rochelle's salt (30 mL) was added and the mixture was stirred for 2 h. The organic layer was separated and washed with brine (30 mL). The aqueous layers were extracted with CH_2CL_2 (3 x 30 mL). The combined organic layers were dried over $MgSO_4$ and solvent removed *in vacuo* to yield the product as a clear oil (241 mg, 100%).$[\alpha]^{212}_D$ -12.0 (c=0.15 Et_2O); IR (film) 3416, 3070, 2960, 2938, 2857, 1428 cm^{-1}; ^1H NMR (500 MHz, $CDCl_3$) δ 7.77-7.71 (4H, m, Ph), 7.52-5.43 (6H, m, Ph), 5.02 (1H, d, *J*=6.0, H-1), 4.02 (1H, dd, *J*=3.5, 6.0, H-3), 3.83 (1H, dd, *J*=3.0, 11.0, Ha-8), 3.60 (1H, dd, *J*=3.5, 11.0, Hb-8), 2.94 (1H, d, *J*=6.0, OH),

2.63-2.50 (1H, m, H-3a), 2.10 (1H, ddd, *J*= 6.0, 6.0, 6.0 H-7a), 1.74-1.46 (6H, m, CH_2), 1.29-1.16 (2H, m, CH_2), 1.05 (9H, s, ^{t}Bu); ^{13}C NMR (125 MHz, $CDCl_3$) δ 135.7 (2 x Ph), 135.7 (2 x Ph), 133.0 (Ph), 132.9 (Ph), 129.8 (Ph), 129.8 (Ph), 127.8 (2 x Ph), 127.8 (2 x Ph), 102.8 (C1), 81.2 (C3), 64.9 (C8), 46.2 (C7a), 34.0 (3a), 26.9 (^{t}Bu), 25.2 (CH_2), 24.0 (CH_2), 23.7 (CH_2), 21.4 (CH_2), 19.2 (Si-C); LC-MS (ESI$^+$) *m/z* 433 [M+Na$^+$], R_t 3.27 min [method D], HRMS [M + Na$^+$] calcd for $C_{25}H_{34}O_3SiNa$ 433.2169; found 433.2166.

((1*S*,3*S*,3a*R*,7a*S*)-3-Allyloctahydroisobenzofuran-1-ylmethoxy)*tert*-butyldiphenylsilane 24

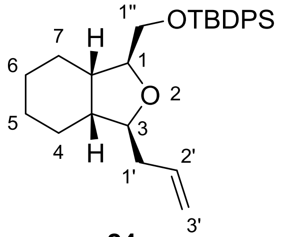

24

(3*S*,3a*S*,7a*R*)-3-((*tert*-Butyldiphenylsilyloxy)methyl)octahydroiso-benzofuran-1-ol **23** (120 mg, 0.618 mmol) was dissolved in CH_2Cl_2 (6 mL) and the solution was cooled to -78 °C. $BF_3.OEt_2$ (240 µL, 1.849 mmol) was added by syringe and the solution was stirred for 5 min. Allyltrimethylsilane (300 µL, 1.85 mmol) was added by syringe and the reaction then warmed to RT over 18 h. The reaction was diluted with 15 mL CH_2Cl_2 (15 mL) and washed with H_2O (7 mL). The aqueous layer was extracted with CH_2Cl_2 (2 x 20 mL). The combined organic layers were washed with brine (10 mL) and dried over $MgSO_4$. The solvents were removed *in vacuo* to yield 24 as a clear oil (268 mg, 100%). $[\alpha]^{22}_D$ 2.11 (c=0.46 CH_2Cl_2); IR (film) 3072, 3050, 2929, 2858, 1472, 1428 cm^{-1}; ^{1}H NMR (500 MHz, $CDCl_3$) δ 7.80-7.65 (4H, m, Ph), 7.50-7.35 (6H, m, Ph), 5.86 (1H, tdd, *J*=7.0, 10.0, 17.0, H-2'), 5.10-5.02 (2H, m, H-3'), 3.91-3.83 (2H, m, H-1, H-3), 3.65 (2H, d, *J*=5.0, H-1''), 2.30-2.18 (3H, m, H-1', H-7a), 1.93 (1H, ddd, *J*=6.0, 6.0, 6.0, H-3a), 1.70-1.58 (2H, m, Ha-7, Ha-4), 1.57-1.45 (4H, m, Hb-4, Ha-5, Ha-6, Hb-7), 1.42-1.32 (2H, m, Hb-5, Hb-6), 1.08 (9H, s, tBu); ^{13}C NMR (125 MHz, $CDCl_3$) 135.7 (2 x Ph), 135.6 (2 x Ph), 134.5 (^{i}Ph), 134.4 (^{i}Ph), 133.8 (Ph), 133.7 (Ph), 130.2 (C2'), 129.6 (Ph), 129.6 (Ph), 127.9 (Ph), 127.6 (Ph), 116.4 (C3'), 82.7 (C1), 81.7 (C3), 65.9 (C1''), 41.8 (C3a), 40.1 (C9), 38.9 (C7a), 26.9 (^{t}Bu), 26.0 (C7 or C4), 25.9 (C4 or C7), 23.4 (C5 or C6), 23.2 (C5 or C6), 19.2 (Si-C).

((1*S*,3*S*,3a*R*,7a*S*)-3-Allyloctahydr-isobenzofuran-1-yl)methanol 25

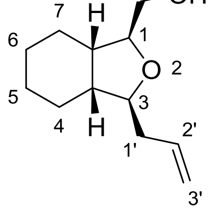

25

((1*S*,3*S*,3a*R*,7a*S*)-3-Allyloctahydroisobenzofuran-1-ylmethoxy)*tert*-butyldiphenylsilane **24** (410 mg, 0.945 mmol) was dissolved in THF (9.4 mL). The solution was cooled to 0 °C under N_2 and then TBAF (2.8 mL, 2.8 mmol, 1.0 M in THF) was added by syringe. The reaction was stirred for 2 h at 0 °C before quenching with Et_2O (30 mL). The reaction mixture was washed with H_2O (2 x 30 mL) and the aqueous layers extracted into Et_2O (2 x 30 mL). The combined organic layers were dried over $MgSO_4$ and the solvents were removed *in vacuo*. The crude residue was purified by column chromatography on silica gel (50% EtOAc:hexane) to yield **25** as a clear oil (131 mg, 74%). $[\alpha]^{22}_D$ -9.0 (c=1.26

CH_2Cl_2); IR (film) 3414, 2929, 2856, 1449 cm^{-1}; ^1H NMR (500 MHz, $CDCl_3$) δ 5.92-5.74 (1H, dddd, *J*=7.0, 7.0, 10.0, 17.0, H-2’), 5.19-5.03 (2H, m, H-3’), 3.97-3.79 (2H, m, H-3, H-1), 3.67 (1H, dd, *J*=3.5, 11.5, H-1’’), 3.45 (1H, dd, *J*=5.5, 11.5, H-1’’), 2.33-2.16 (3H, m, H-1’), 2.11 (1H, ddd, *J*=6.5, 6.5, 12.5, H-7a), 3.50-3.43 (1H, m, H-3a), 1.70-1.69 (2H, m, CH_2), 1.59-1.28 (6H, m, 6 x CH_2); ^{13}C NMR (125 MHz, $CDCl_3$) δ 135.1 (C2’), 117.1 (C3’), 82.2 (C3 or C1), 82.1 (C3 or C1), 64.3 (C1’’), 42.2 (C3a), 40.1 (C1’), 37.8 (C7a), 26.4 (CH_2, 25.6 (CH_2), 23.4 (CH_2), 22.8 (CH_2); LC-MS (ESI$^+$) *m/z* 219 [M+Na$^+$], R_t 2.47 min [method D]; HRMS [M+Na$^+$] calcd for $C_{12}H_{20}O_3Na$ 219.1362; found 219.1363.

(Z)-(1*S*,10*S*,11*R*,16*S*)-3,17-Dioxatricyclo[8.6.1.0^{11,16}]heptadeca-7-en-4-one 26 and (E)-(1*S*,10*S*,11*R*,16*S*)-3,17-Dioxatricyclo[8.6.1.0^{11,16}]heptadeca-7-en-4-one 27

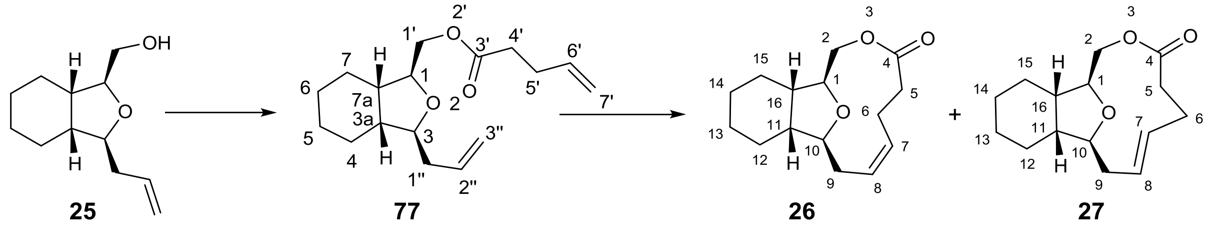

((1*S*,3*S*,3a*R*,7a*S*)-3-Allyloctahydroisobenzofuran-1-yl) methanol **25** (40 mg, 0.203 mmol) was dissolved in CH_2Cl_2 (0.9 mL). Pent-4-enoyl chloride (29 µL, 0.275 mmol) and triethylamine (89 µL, 0.609 mmol) were added and the reaction mixture was stirred at RT for 18 h. The reaction mixture was diluted with CH_2Cl_2 (10 mL) and washed with H_2O (10 mL x 2) and brine (10 mL). The aqueous layers were combined and extracted with CH_2Cl_2 (10 mL x 2). The combined organic layers were dried with $MgSO_4$ and the solvents removed *in vacuo*. The crude reaction mixture was purified by column chromatography on silica gel (10% EtOAc:hexane) to yield **77** as a clear oil (39 mg, 69%).$[\alpha]^{20}_D$ -1.2 (c=1.0 $CHCl_3$); IR ($CDCl_3$ solution cell) 3081, 2932, 1732 (CO), 1641, 1451 cm^{-1}; ^1H NMR (500 MHz, $CDCl_3$) δ 5.93-5.73 (2H, m, H-6’, H-2’’), 5.17-4.97 (4H, m, H-7’, H-3’’), 4.14 (1H, dd, *J*=6.5, 14.0, Ha-1’), 4.03-3.93 (2H, m, Hb-1’, H-1), 3.86 (1H, dd, *J*=6.0, 11.5, H-3), 2.50-2.35 (4H, m, H-4’, H-5’), 2.29-2.17 (2H, m, H-1’’), 2.09-2.01 (1H, m, H-7a), 2.00-1.91 (1H, m, H-3a), 1.70-1.59 (2H, m, CH_2), 1.58-1.28 (6H, m, CH_2); ^{13}C NMR (125 MHz, $CDCl_3$) δ 173.1 (C3’), 136.7 (C6’ or C2’’), 135.0 (C6’ or C2’’), 116.9 (C7’ or C3’’), 115.5 (C7’ or C3’’), 82.4 (C3), 79.8 (C1), 66.6 (C1’), 41.6 (C3a), 40.0 (C1’’), 39.1 (C7a), 33.5 (C4’), 28.8 (C5’), 26.2 (CH_2), 25.8 (CH_2), 23.2 (CH_2), 22.9 (CH_2); LC-MS (ESI$^+$) *m/z* 301 [M+Na$^+$], R_t 2.93 min [method D]; HRMS [M+Na$^+$] calcd for $C_{17}H_{26}O_3$ Na 301.1774 found 301.1765.

((1S,3S,3aR,7aS)-3-Allyloctahydroisobenzofuran-1-yl)methyl pent-4-enoate **77** (23 mg, 0.083 mmol) was dissolved in CH_2Cl_2 (165 mL, 0.0005 M). Argon was bubbled though the solution for 5 min. Grubbs II (7 mg, 0.008 mmol, 10 mol%) was added under argon and the reaction

mixture was stirred for 16 h. DMSO (10 µL) was added and the solution was stirred overnight. The solvents were removed *in vacuo* and the resultant mixture purified by column chromatography on silica gel (10% EtOAc:hexane) to yield **26** and **27** as a white crystalline solid (13 mg, 65%) and a clear oil (4 mg, 20%) respectively. **26**: mp (141-143 ˚C); $[\alpha]^{19}_{D}$ -1.9 (c=0.36 CH_2Cl_2); IR (film) 2980, 1732 (CO), 1551 cm^{-1}; ^{1}H NMR (500 MHz, Pyr-d5) δ 5.53-5.38 (2H, m, H-8, H-7), 5.25 (1H, d, *J*=11.5 Ha-2), 3.92 (1H, d, *J*=11.0, H-1), 3.71 (1H, d, *J*=11.5, H-10), 3.39 (1H, d, *J*=11.5, Hb-2), 2.85-2.81 (1H, m, Ha-6), 2.61-2.46 (2H, m, Ha-5, Ha-9), 2.43-2.27 (2H, m, H-16, Hb-5), 1.83-1.79 (1H, m, Hb-6), 1.75-1.61 (2H, m, Ha-9, H-11), 1.59-1.48 (4H, m, Ha-12, Ha-14, H-15), 1.38-1.36 (1H, m, Ha-13), 1.21-0.89 (3H, m, Hb-14, Hb-13, Hb-12)); ^{13}C NMR (125 MHz, $CDCl_3$) δ 172.2 (C4), 129.7 (C8), 128.8 (C7), 83.5 (C10), 77.6 (C1), 60.3 (C2), 45.2 (C11), 36.7 (C16), 35.9 (C14), 35.0 (C9), 28.2 (C5), 25.6 (C12), 23.7 (C15), 23.5 (C13 or C14), 21.7 (C13 or C14); LC-MS (ESI^+) *m/z* 273 $[M+Na^+]$, R_t 2.30 min [method D]; HRMS $[M+H^+]$ calcd for $C_{15}H_{23}O_3$ 251.1642; found 251.1634. **27**: $[\alpha]^{19}_{D}$ -49.1 (c=0.24 CH_2Cl_2); IR (film) 2925, 1735 (CO) cm^{-1}; ^{1}H NMR (500 MHz, Pyr-d5) δ 5.57-5.50 (1H, m, H-8), 5.48-5.40 (1H, m, H-7), 4.92-4.68 (1H, m, Ha-2), 4.02-3.95 (1H, m, H-10), 3.77-3.74 (2H, m, Hb-2, H-1), 2.49-2.46 (1H, m, Ha-9), 2.39-2.30 (2H, m, Ha-5, Ha-6), 2.26-2.19 (3H, m Hb-5, Hb-6, H-11), 2.11-2.09 (1H, m, H-16), 1.95-1.91 (1H, m, Hb-9), 1.49-1.40 (2H, m, Ha-13, Ha-12), 1.39-1.35 (2H, m, Hb-12, Ha-15), 1.34-1.30 (3H, m, H-14, Hb-15), 1.22-1.17 (1H, m, Hb-13); ^{13}C NMR (125 MHz, $CDCl_3$) δ 173.5 (C4), 131.2 (C7), 127.9 (C8), 82.2 (C1), 80.5 (C10), 64.9 (C2), 40.0 (C16), 38.8 (C11), 36.1 (C5), 34.2 (C9), 29.5 (C6), 27.3 (C15), 25.1 (C12), 23.9 (C13), 22.3 (C14); LC-MS (ESI^+) *m/z* 273 $[M+Na^+]$, R_t 2.27 min [method D]; HRMS $[M+H^+]$ calcd for $C_{15}H_{23}O_3$ 251.1642; found 251.1636.

(Z)-(1S,11S,12R,17S)-3,18-Dioxatricyclo[9.6.1.0^{12,17}]octadec-8-en-4-one 28

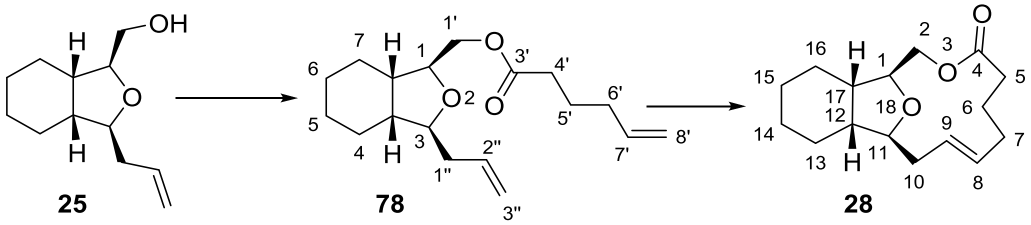

((1*S*,3*S*,3a*R*,7a*S*)-3-Allyloctahydroisobenzofuran-1-yl)methanol **25** (35 mg, 0.178 mmol) was dissolved in CH_2Cl_2 (0.9 mL). Freshly prepared hex-5-enoyl chloride (30 mg, 0.267 mmol) and triethylamine (77 µL, 0.534 mmol) were added and the reaction mixture was stirred at RT for 18 h. The reaction mixture was diluted with CH_2Cl_2 (10 mL) and washed with H_2O (10 mL) and brine (10 mL). The aqueous layers were combined and extracted with CH_2Cl_2 (10 mL x 2). The combined organic layers were dried with $MgSO_4$ and the solvents removed *in vacuo*. The crude reaction mixture was purified by column chromatography on silica gel (5% EtOAc:hexane) to yield **78** as a clear oil (27 mg, 57%). $[\alpha]^{19}_{D}$ -9.0 (c=1.0 CH_2Cl_2); IR ($CDCl_3$ solution cell) 3080, 2932, 1731 (CO), 1641, 1451 cm^{-1}; ^{1}H NMR (500 MHz, $CDCl_3$) δ 5.89-5.71 (2H, m, H-2'', H-7'), 5.13-4.95 (4H, m, H-3'', H-8'), 4.14 (1H, dd, *J*=6.5, 6.5, Ha-1'), 4.05-3.94

(2H, m, Hb-1′, H-1), 3.87 (1H, dd, *J*=6.0, 11.5, H-3), 2.38 (2H, t, *J*=7.0, H-4′), 2.31-2.17 (2H, m, H-1′′), 2.14-2.00 (3H, m, H-6′, H-7a), 2.00-1.92 (1H, m, H-3a), 1.75 (2H, tt, *J*=7.5, 7.5, H-5′), 1.70-1.58 (2H, m, CH_2), 1.57-1.23 (6H, m, CH_2); ^{13}C NMR (125 MHz, $CDCl_3$) δ 173.7 (C3′), 137.7 (C7′ or C2′′), 135.1 (C7′ or C2′′), 116.9 (C3′′ or C8′), 115.4 (C3′′ or C8′), 82.5 (C1), 79.4 (C3), 66.5 (C8), 41.6 (C3a), 40.0 (C1′′), 39.2 (C7a), 33.5 (C4′), 33.1 (C6′), 26.2 (CH_2), 25.7 (CH_2), 24.0 (C5′), 23.3 (CH_2), 22.9 (CH_2); LC-MS (ESI$^+$) *m/z* 315 [M+Na$^+$], R_t 2.63 min [method D]; HRMS [M+Na$^+$] calcd for $C_{18}H_{28}O_3Na$ 315.1930; found 315.1935.

((1S,3S,3aR,7aS)-3-Allyloctahydroisobenzofuran-1-yl)methyl hex-5-enoate **78** (25 mg, 0.080 mmol) was dissolved in CH_2Cl_2 (100 mL, 0.0008 M). Argon was bubbled though the solution for 5 min. Grubbs II (7 mg, 0.008 mmol, 10 mol%) was added under argon and the reaction mixture was stirred for 16 h. DMSO (10 µL) was added and the solution was stirred overnight. The solvents were removed *in vacuo* and the resultant mixture purified by column chromatography on silica gel (10% EtOAc:hexane) to yield **28** as a white crystalline solid (10 mg, 51%). mp (141-143 ˚C); $[\alpha]^{25}_D$ -33.4 (c=1.0 $CHCl_3$); IR (film) 3081, 2931, 2858, 1731, 1642, 1451 cm^{-1}; ^1H NMR (500 MHz, $CDCl_3$) δ 5.39-5.35 (2H, m, H-8, H-9), 5.16 (1H, d, *J*=11.0, Ha-2), 3.95-3.91 (1H, m, H-11), 3.81-3.78 (1H, m, H-1), 3.47 (1H, d, *J*=11.0, Hb-2), 2.45-2.28 (3H, m, 3 x CH_2), 2.26-2.18 (1H, m, H-17), 2.17-1.97 (4H, m, 4 x CH_2), 1.95-1.85 (1H, m, CH_2), 1.73-1.61 (4H, m, CH_2), 1.57-1.29 (5H, m, CH_2); ^{13}C NMR (125 MHz, $CDCl_3$) δ 173.8 (C4), 133.5 (C8 or C9), 125.8 (C8 or C9), 81.2 (C1), 80.3 (C11), 63.0 (C2), 39.8 (C17), 39.1 (C12), 34.7 (CH_2), 34.0 (CH_2), 33.7 (CH_2), 27.1 (CH_2), 25.5 (CH_2), 23.9 (CH_2), 22.9 (CH_2), 22.5 (CH_2); LC-MS (ESI$^+$) *m/z* 287 [M+Na$^+$], R_t 2.50 min [method D]; HRMS [M+H$^+$] calcd for $C_{16}H_{25}O_3$ 265.1798 found 265.1796.

(1*S*,3*S*,3a*S*,7a*R*)-1-Allyl-3-(allyloxymethyl)-1,3,3a,4,7,7a-hexahydroisobenzofuran 29

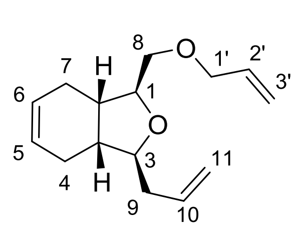

29

((1*S*,3*S*,3a*R*,7a*S*)-3-Allyl-1,3,3a,4,7,7a-hexahydroisobenzofuran-1-yl)methanol **14** (48 mg, 0.247 mmol) was dissolved in DMF (1 mL). NaH (24 mg, 0.618 mmol, 60% dispersion in mineral oil) was then added to the solution followed by allyl bromide (25 µL, 0.296 mmol) by syringe. The reaction was stirred overnight at RT. The reaction was then quenched with H_2O (2 mL) and extracted with Et_2O (5 x 10 mL) and the organic layers subsequently washed with H_2O (5 x 20 mL). The organic layer was dried with $MgSO_4$ and solvents removed *in vacuo*. The resultant crude oil was purified by column chromatography on silica gel (15% EtOAc:hexane) to yield **29** as a clear oil (28 mg, 49%). $[\alpha]^{19}_D$ -9.8 (c=2.20, CH_2Cl_2); IR (film) 3076, 3025, 2900, 2843, 1641, 1437 cm^{-1}; ^1H NMR (500 MHz, $CDCl_3$) δ 6.01-5.76 (2H, m, H-2′, H-10), 5.79-5.66 (2H, m, H-5, H-6), 5.33-5.32 (1H, m, Ha-3′), 5.23-5.13 (1H, m, Hb-3′), 5.13-5.02 (2H, m, H-11), 4.10-4.00 (2H, m, H-1′), 3.81 (1H, ddd, *J*=5.5, 5.5, 5.5, H-1), 3.73 (1H, dd, *J*=6.0, 6.0, 6.0, H-3), 3.52-3.44 (2H, m, H-8), 2.38-2.16 (5H, m, H-9, H-7a, Ha-4, Ha-7), 2.11 (1H, ddd, *J*=6.5, 6.5, 13.0, H-3a), 2.01-1.87 (2H, m,

Hb-7, Hb-4); ^{13}C NMR (125 MHz, $CDCl_3$) δ 135.2 (C2'), 134.9 (C10), 125.8 (C5 or C6), 125.7 (C5 or C6), 116.7 (C3' or C11), 116.7 (C3' or C11), 83.6 (C3), 82.7 (C1), 72.5 (C8 or C1'), 72.4 (C8 or C1'), 39.5 (C9), 39.3 (C3a), 37.3 (C7a), 24.8 (C7), 24.8 (C4); LC-MS (ESI^+) *m/z* 257 [$M+Na^+$], R_t 2.90 min [method D]; HRMS [$M+Na^+$] calcd for $C_{15}H_{22}O_2Na$ 257.1512; found 257.1513.

(3aR,4aR,7S,7aS,8aS)-7-(*tert*-Butyldiphenylsilanyloxymethyl)-2,2-dimethyl-hexahydro-furo[3',4':4,5]benzo[1,2-d][1,3]dioxol-5-one 31

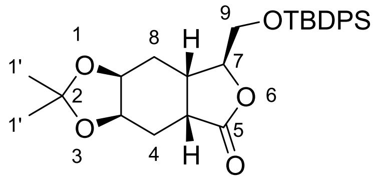

31

(3S)-3-((tert-Butyldiphenylsilyloxy)methyl)-3a,4,7,7a-tetrahydroisobenzofuran-1-(3H)-one **10** (1.30 g, 3.21 mmol) was dissolved in acetone:water (10:1, 32 mL). NMO (114 mg, 4.82 mmol) was added followed by Os EnCat (500 mg, 0.25 mmol/g, 0.125 mmol). The resulting solution was stirred at RT for 3 d. The Os Encat was removed by vacuum filtration, and washed with acetone (100 mL). Sat. aq $Na_2S_2O_5$ was added (100 mL) and stirred for 2 h. The acetone was removed *in vacuo.* The crude aqueous layer was extracted in the EtOAc (3 x 100 mL). The combined organic layers were dried over $MgSO_4$ and solvent removed in vacuo to yield a light brown oil. The oil was redissolved in CH_2Cl_2 (30 mL). 2,2-Dimethoxypropane (7.9 mL, 64.2 mmol) and pyridinium p-toluenesulfonate (241 mg, 1.08 mmol) were added and the reaction stirred at RT for 18 h. The reaction was then diluted in CH_2Cl_2 (100 mL) and washed with H_2O (50 mL) and sat aq NH_4Cl (50 mL). The aqueous layers were re-extracted into CH_2Cl_2 (100 mL) and the combined organic layers dried over $MgSO_4$. The solvent was removed *in vacuo*. The crude residue was recrystallised from Et_2O to yield **31** as a white crystalline solid (781 mg, 52%). mp (105-109 ˚C); $[\alpha]^{22}_D$ -16.2 (c=1.0 $CHCl_3$); IR (film) 3071, 2931, 1770 cm^{-1}; ^1H NMR (500 MHz, $CDCl_3$) δ 7.70-7.63 (4H, m, Ph), 7.49-7.39 (6H, m, Ph), 4.51 (1H, ddd, *J*=2.5, 2.5, 8.0, H-3a), 4.42 (1H, ddd, *J*=2.5, 2.5, 8.0, H-8a), 4.20-4.17 (1H, m, H-7), 3.88 (1H, dd, *J*=2.5, 11.0, Ha-9), 3.69 (1H, dd, *J*=2.5, 11.0, Hb-9), 3.13 (1H, ddd, *J*=7.5, 11.0, 11.0, H-4a), 2.94-2.87 (1H, m, H-7a), 2.43 (1H, ddd, *J*=3.0, 7.5, 15.0, Ha-4), 1.97 (1H, ddd, *J*=2.5, 4.5, 14.0, Ha-8), 1.59-1.44 (2H, m, Hb-4, Hb-8), 1.52 (3H, s, H-1'), 1.38 (3H, m, H-1'), 1.09 (9H, s, ^{t}Bu); ^{13}C NMR (125 MHz, $CDCl_3$) δ 180.2 (C5), 135.6 (2 x Ph), 135.5 (2 x Ph), 132.8 (iPh), 132.5 (iPh), 129.9 (2 x Ph), 127.9 (4 x Ph), 107.4 (C2), 82.8 (C7), 71.5 (C8a or C3a), 71.3 (C8a or C3a), 65.4 (C9), 34.4 (C4a), 30.2 (C7a), 29.6 (C8), 26.7 (^{t}Bu), 25.9 (C1'), 25.0 (C4), 23.8 (C1'), 19.2 (Si-C); LC-MS (ESI^+) *m/z* 503 [$M+Na^+$], R_t 2.58 min [method D]; HRMS [$M+Na^+$] calcd for $C_{28}H_{36}O_5SiNa$ 503.2224 found 503.2221.

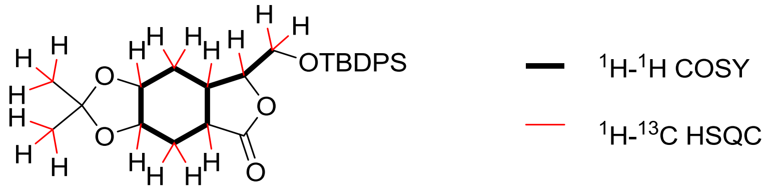

(3aR,4aR,7S,7aS,8aS)-7-(*tert*-Butyldiphenylsilanyloxymethyl)-2,2-dimethyloctahydrofuro [3',4':4,5]benzo[1,2-*d*][1,3]dioxol-5-ol 32

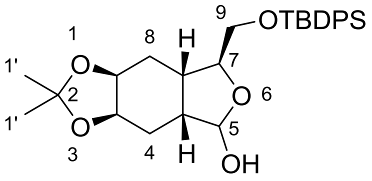

32

(3aR,4aR,7S,7aS,8aS)-7-(*tert*-Butyldiphenylsilanyloxymethyl)-2,2-dimethyl-hexahydro-furo[3',4':4,5]benzo[1,2-d][1,3]dioxol-5-one **31** (96 mg, 0.200 mmol) was dissolved in anhydrous CH_2Cl_2 (2.0 mL) and cooled to -78 °C under N_2. DIBAL-H (1.0 M in toluene, 0.22 mL, 0.22 mmol) was added dropwise. The reaction was stirred at -78 °C for 4 h then quenched with EtOAc (10 mL). The solution was warmed to RT and a sat. aq. solution of Rochelles salt (6 mL) was added and the mixture stirred overnight. The aqueous layer was separated and extracted with CH_2Cl_2 (20 mL x 3). The organic layers were combined, dried over $MgSO_4$ and the solvent removed *in vacuo.* TLC analysis showed the presence of starting material, therefore the crude mixture was resubmitted to the above conditions for 5 h. The same work-up conditions were employed which yielded a clear oil **32** (89 mg, 92%) as an inseparable mixture of anomers. IR (film) 3418, 2930, 1113, 1040 cm^{-1}; **Major Anomer** ^{1}H NMR (500 MHz, $CDCl_3$) δ 7.72-7.67 (4H, m, Ph), 7.49-7.36 (6H, m, Ph), 5.10 (1H, d, *J*= 9.0, H-5), 4.41-4.37 (2H, m, H-3a, H-8a), 4.15-4.10 (1H, m, OH), 3.92 (1H, dd, *J*=4.0, 4.0, H-7), 3.82 (1H, dd, *J*=3.0, 11.0, Ha-9), 3.60 (1H, dd, *J*=3.5, 11.0, Hb-9), 2.73-2.63 (1H, m, H-7a), 2.55-2.46 (1H, m, H-4a), 2.03 (1H, ddd, *J*=2.5, 5.0, 14.5, Ha-4), 1.96 (1H, ddd, *J*=3.0, 6.0, 14.5, Ha-8), 1.50 (3H, s, H-1’), 1.36 (3H, s, H-1’), 1.37-1.28 (1H, m, Hb-8), 1.39-1.31 (1H, m, Hb-4), 1.09 (9H, s, tBu); ^{13}C NMR (125 MHz, $CDCl_3$) δ 135.8 (2 x Ph), 135.6 (2 x Ph), 132.4 (iPh), 132.3 (iPh), 130.1 (Ph), 130.0 (Ph), 127.9 (2 x Ph), 127.9 (2 x Ph), 107.1 (C2), 105.1 (C5), 87.6 (C7), 72.0 (C8a or C3a), 71.7 (C8a or C3a), 66.9 (C9), 41.5 (C4a), 32.0 (C7a), 29.0 (C8), 27.5 (C4), 26.8 (^{t}Bu), 26.0 (C1’), 23.8 (C1’), 19.2 (Si-C); **Minor Anomer** ^{1}H NMR (500 MHz, $CDCl_3$) δ 7.72-7.67 (4H, m, Ph), 7.49-7.36 (6H, m, Ph), 5.53 (1H, dd, *J*= 2.0, 5.5, H-5), 4.52 (1H, dd, *J*= 3.0, 3.0, H-3a or H-8a), 4.51 (1H, dd, *J*= 3.0, 3.0, H-3a or H-8a) 4.15-4.10 (1H, m, OH), 3.96 (1H, ddd, *J*=4.5, 4.5, 7.0, H-7), 3.75 (2H, d, *J*= 4.5, H-9), 2.36-2.26 (1H, m, H-7a), 1.95-1.91 (1H, m, H-4a), 1.80 (1H, ddd, *J*=2.0, 5.0, 14.5, Ha-4), 1.74 (1H, ddd, *J*=2.5, 12.0, 14.5, Ha-8), 1.45 (3H, s, H-1’), 1.35 (3H, s, H-1’), 1.44-1.40 (1H, m, Hb-8), 1.27-1.23 (1H, m, Hb-4), 1.06 (9H, s, ^{t}Bu); ^{13}C NMR (125 MHz, $CDCl_3$) δ 135.7 (2 x Ph), 135.2 (2 x Ph), 133.6 (iPh), 133.5 (iPh), 129.6 (2 x Ph), 127.9 (2 x Ph), 127.9 (2 x Ph), 106.8 (C2), 99.4 (C5), 84.0 (C7), 72.7 (C8a or C3a), 71.5 (C8a or C3a), 65.5 (C9), 36.8 (C4a), 31.7 (C7a), 29.8 (C8 or C4), 26.8 (^{t}Bu), 26.0 (C1’), 23.8

(C1'), 22.9 (C8 or C4) 19.3 (Si-C); LC-MS (ESI$^+$) *m/z* 505 [M+Na$^+$], R_t 2.60 min [method D]; C, 70.07; H, 7.53%; $C_{28}H_{38}O_5Si$ requires: C, 69.97; H, 7.55%.

((3aS,4aS,5S,7S,7aR,8aR)-7-allyl-2,2-dimethyloctahydroisobenzofuro[5,6-d][1,3]dioxol-5-yl)methanol 33

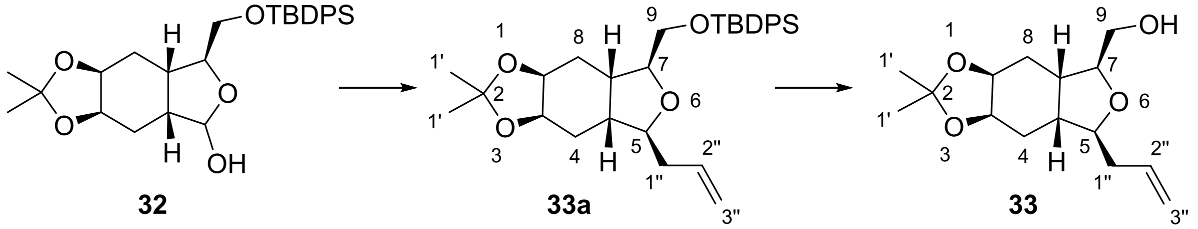

32 33a 33

(3aR,4aR,7S,7aS,8aS)-7-(*tert*-Butyldiphenylsilanyloxymethyl)-2,2-dimethyloctahydrofuro [3',4':4,5]benzo[1,2-d][1,3]dioxol-5-ol **32** (95 mg, 0.197 mmol) was dissolved in CH_2Cl_2 (2.0 mL) and cooled to -78 °C. $BF_3.OEt_2$ (0.027 mL, 0.217 mmol) was added by syringe and the solution stirred for 5 min. Allyltrimethylsilane (0.096 mL, 0.519 mmol) was added by syringe and the reaction warmed to RT over 18 h. The reaction was quenched with H_2O (6 mL) and extracted into CH_2Cl_2 (6 mL x 3). The combined organic layers were dried over $MgSO_4$. The solvent was removed in vacuo to yield a clear oil. Partial acetal deprotection had occurred. The oil was dissolved in CH_2Cl_2 (2.0 mL). 2,2-Dimethoxypropane (0.5 mL, 3.94 mmol) and pyridinium p-toluenesulfonate (12 mg, 0.059 mmol) were added and the reaction stirred as RT overnight. The reaction was then diluted with CH_2CL_2 (50 mL), washed with H_2O (50 mL) and sat. aqueous NH_4Cl (50 mL). The aqueous layers were extracted with CH_2CL_2 (50 mL) and the combined organic layers were dried over $MgSO_4$. The solvent was removed in vacuo to yield **33a** a clear oil (79 mg, 79%). $[\alpha]^{20}_D$ +0.64 (c=2.0 $CHCl_3$); IR (film) 3049, 2931, 1472, 1428 cm^{-1}; ^1H NMR (500 MHz, $CDCl_3$) δ 7.75-7.69 (4H, m, Ph), 7.44-7.36 (6H, m, Ph), 5.89 (1H, dddd, *J*=7.5, 7.5, 10.0, 17.5, H-2''), 5.15-5.05 (2H, m, H-3''), 4.45-4.38 (2H, m, H-3a, H-8a), 3.79 (1H, dd, *J*=5.0, 10.5, Ha-9), 3.74 (1H, dd, *J*=5.0, 10.5, Hb-9), 3.57 (1H, ddd, *J*=4.5, 4.5, 7.5, H-7), 3.50 (1H, ddd, *J*=5.5, 5.5, 14.5, H-5), 2.39-2.35 (3H, m, H-7a, H-1''), 2.14-2.05 (1H, m, H-4a), 1.97 (1H, ddd, *J*=2.5, 6.0, 14.5, Ha-4 or Ha-8), 1.90 (1H, ddd, *J*=2.5, 5.5, 14.5, Ha-4 or Ha-8), 1.47 (3H, s, H-1'), 1.34 (3H, s, H-1'), 1.32-1.20 (2H, m, Hb-4, Hb-8), 1.07 (9H, s, tBu); ^{13}C NMR (125 MHz, $CDCl_3$) δ 135.7 (2 x Ph), 135.7 (2 x Ph), 135.0 (C2''), 133.7 (2 x iPh), 129.5 (Ph), 129.5 (Ph), 127.6 (2 x Ph), 127.5 (2 x Ph), 116.8 (C3'), 107.1 (C2), 85.5 (C5 or C7), 85.0 (C5 or C7), 72.6 (C3a or C8a), 72.4 (C3a or C8a), 65.8 (C9), 38.9 (C1''), 37.1 (C4a), 34.1 (C7a), 28.8 (C4 or C8), 27.7 (C4 or C8), 26.8 (tBu), 26.0 (C1'), 23.8 (C1'), 19.2 (Si-C); LC-MS (ESI$^+$) *m/z* 529 [M+Na$^+$], R_t 2.47 min [method E]; HRMS [M+Na$^+$] calcd for $C_{31}H_{42}O_4SiNa$ 529.2755 found 529.2744; Found: C, 73.58; H, 8.40%; $C_{31}H_{42}O_4Si$ requires: C, 73.47; H, 8.35%.

(((3aS,4aS,5S,7S,7aR,8aR)-7-allyl-2,2-dimethyloctahydroisobenzofuro[5,6-d][1,3]dioxol-5-yl)methoxy)(tert-butyl)diphenylsilane **33a** (506 mg, 1.09 mmol) was dissolved in THF (10

mL). The solution was cooled to 0 °C under N_2 and TBAF (3.26 mL, 3.26 mmol, 1.0 M in THF) was added by syringe. The reaction was stirred for 3 h at 0 °C before diluted with Et_2O (50 mL). The reaction mixture was washed with H_2O (50 mL x 3) and extracted into Et_2O (50 mL x 2). The combined organic layers were washed with brine (50 mL) and dried over $MgSO_4$. The solvent was removed *in vacuo* to give the crude product. This was purified by column chromatography on silica gel (5% MeOH:CH_2Cl_2) to yield **33** as a clear oil (193 mg, 66%). $[\alpha]^{23}_D$ +6.9 (c=1.0, $CHCl_3$); IR (film) 3448, 2931, 1381, 1042 cm^{-1}; ^1H NMR (500 MHz, $CDCl_3$) δ 5.86 (1H, dddd, *J*=7.0, 7.0, 10.5, 17.0, H-2''), 5.16-5.06 (2H, m, H-3''), 4.45-4.40 (2H, m, H-3a, H-8a), 3.79 (1H, dd, *J*=2.5, 12.0, Ha-9), 3.63-3.51 (3H, m, Hb-9, H-7, H-5), 2.41-2.36 (2H, m, H-1''), 2.27-2.19 (1H, m, H-7a), 2.13-2.05 (1H, m, H-4a), 1.95-1.89 (2H, m, Ha-4, Ha-8), 1.46 (3H, s, H-1'), 1.33 (3H, s, H-1'), 1.32-1.21 (2H, m, Hb-4, Hb-8); ^{13}C NMR (125 MHz, $CDCl_3$) δ 134.5 (C2''), 177.2 (C3''), 107.2 (C2), 85.7 (C5 or C7), 85.0 (C5 or C7), 72.3 (C8a or C3a), 72.3 (C8a or C3a), 64.0 (C9), 38.7 (C1''), 37.0 (C4a), 33.0 (C7a), 28.1 (C4 or C8), 27.9 (C4 or C8), 26.0 (C1'), 23.7 (C1'); LC-MS (ESI^+) *m/z* 291 $[M+Na^+]$, R_t 2.12 min [method D]; HRMS $[M+Na^+]$ calcd for $C_{15}H_{24}O_4$ Na 291.1567 found 291.1575; Found: C, 67.21; H, 9.02%; $C_{15}H_{24}O_4$ requires: C, 67.14; H, 9.01%.

((3aS,4aS,5S,7S,7aR,8aR)-7-allyl-2,2-dimethyloctahydroisobenzofuro[5,6-d][1,3]dioxol-5-yl)methyl hex-5-enoate 34

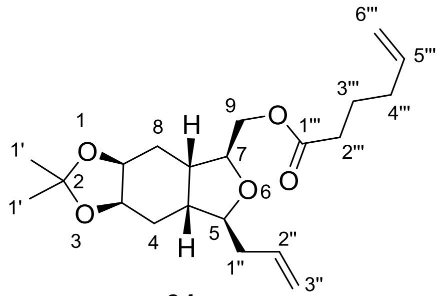

34

((3aS,4aS,5S,7S,7aR,8aR)-7-Allyl-2,2-dimethyloctahydroisobenzofuro[5,6-d][1,3]dioxol-5-yl)methanol **33** (92 mg, 0.343 mmol) was dissolved in CH_2Cl_2 (3.4 mL). Hex-5-enoyl chloride (136 mg, 1.029 mmol) and triethylamine (148 µL, 1.029 mmol) were added and the reaction mixture was stirred at RT for 18 h. The reaction mixture was diluted with CH_2Cl_2 (10 mL) and washed with sat. aqueous $NaHCO_3$ (15 mL) and H_2O (15 mL x 2). The aqueous layers were combined and extracted with CH_2Cl_2 (10 mL x 2). The combined organic layers were dried over $MgSO_4$ and the solvents removed *in vacuo*. The crude reaction mixture was purified by column chromatography on silica gel (10% EtOAc:hexane) to yield **34** as a clear oil (116 mg, 93%). The compound was observed to decomposed on RT storage over several weeks. $[\alpha]^{23}_D$ +10.5 (c=1.0 $CHCl_3$); IR (film) 2937, 1732, 1381, 1163, 1040 cm^{-1}; ^1H NMR (500 MHz, $CDCl_3$) δ 5.90-5.74 (2H, m, H-2', H-5'''), 5.16-4.97 (4H, m, H-3'', H-6'''), 4.45-4.40 (2H, m, H-3a, H-8a), 4.28 (1H, dd, *J*=3.5, 12.0, Ha-9), 4.09 (1H, dd, *J*=7.0, 12.0, Hb-9), 3.64 (1H, ddd, *J*=3.5, 7.0, 7.0, H-5), 3.52 (1H, ddd, *J*=6.0, 6.0, 7.5, H-7), 2.45-2.34 (4H, m, H-2''', H-1''), 2.20-2.07 (4H, m, H-4''', H-4a, H-7a), 1.94 (2H, m, Ha-4, Ha-8), 1.76 (1H, dddd, *J*=7.0, 7.0, 7.0, 7.0, Ha-3'''), 1.75 (1H, dddd, *J*=7.0, 7.0, 7.0, 7.0, Hb-3'''), 1.46 (3H, s, H-1'), 1.34 (3H, s, H-1'), 1.33-1.20 (2H, m, Hb-4, Hb-8); ^{13}C NMR

(125 MHz, $CDCl_3$) δ 173.5 (C1'''), 137.7 (C2' or C5'''), 137.5 (C2' or C5'''), 177.2, (C3' or C8'''), 115.3 (C3' or C8'''), 107.2 (C2), 85.2 (C7), 82.7 (C5), 72.3 (C3a or C8a), 72.2 (C3a or C8a), 65.7 (C9), 38.8 (C1''), 36.5 (C7a or C5a), 34.5 (C7a or C5a), 33.4 (C2''' or C4'''), 33.0 (C2''' or C4'''), 28.0 (C4 or C8), 28.0 (C4 or C8), 26.0 (C1'), 24.0 (C3'''), 23.7 (C1'); LC-MS (ESI^+) m/z 387 [$M+Na^+$], R_t 2.78 min [method D]; Found: C, 69.17; H, 8.85%; $C_{21}H_{32}O_5Na$ requires: C, 69.20; H, 8.85%.

(1S,2R,4R,8S,10S,11S,18E)-6,6-dimethyl-5,7,13,21-tetraoxatetracyclo[9.9.1.0^{2,10}.0^{4,8}]henicos-18-en-14-one 35

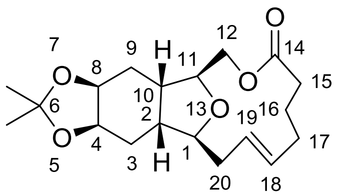

35

((3aS,4aS,5S,7S,7aR,8aR)-7-Allyl-2,2-dimethyloctahydroisobenzofuro[5,6-d][1,3]dioxol-5-yl)methyl hex-5-enoate **34** (53 mg, 0.145 mmol) was dissolved in CH_2Cl_2 (100 mL, 0.0014 M). Argon was bubbled though the solution for 5 min. Grubbs II (12 mg, 0.0001 mmol, 10 mol%) was added under argon and the reaction mixture was stirred for 5 d. DMSO (10 µL) was added and the solution was stirred overnight. The solvents were removed *in vacuo* and the resultant mixture purified by column chromatography on silica gel (15% EtOAc:hexane) to yield **35** as a purple crystalline solid containing trace ruthenium impurities (35 mg, 51%). ^1H NMR (500 MHz, $CDCl_3$) *δ* 5.46-5.36 (2H, m, H-18, H-19), 5.56 (1H, dd, *J*=2.0, 12.0, Ha-12), 4.47-4.42 (2H, m, H-8, H-4), 3.66-3.61 (2H, m, H-1, H-11), 3.46 (1H, d, *J*=12.0, Hb-12), 2.55-2.47 (1H, m, H-2 or H-10), 2.78-2.26 (4H, m, Ha-15, Ha-16, Ha-20, H-2 or H-10), 2.13-2.09 (3H, m, Ha-17, Hb-15, Hb-16), 1.97-1.83 (3H, m, Ha-9, Ha-3, Hb-20), 1.68-1.60 (1H, m, Hb-17), 1.53 (3H, s, H-6'), 1.37 (3H, s, H-6'), 1.38-1.21 (2H, m, Hb-9, Hb-3); ^{13}C NMR (125 MHz, $CDCl_3$) *δ* 174.0 (C14), 134.8 (C18), 124.3 (C19), 107.3 (C6), 84.2 (C11 or C1), 83.6 (C11 or C1), 72.5 (C8 or C4), 72.4 (C4 or C8), 61.3 (C12), 34.6 (C17), 34.0 (C16 or C15), 33.6 (C10 or C2), 33.5 (C15 or C16), 33.3 (C10 or C2), 28.4 (C9 or C3), 28.0 (C9 or C3), 26.1 (C6'), 23.9 (C6'), 22.6 (C17); LC-MS (ESI^+) *m/z* 359 [$M+Na^+$], R_t 2.47 min [method D].

(E)-(1S,11S,12R,14R,15S,17S)-14,15-Dihydroxy-3,18-dioxa-tricyclo[9.6.1.0^{12,17}]octadec-8-en-4-one 36

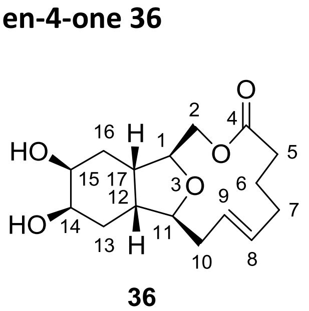

36

(1S,2R,4R,8S,10S,11S,18E)-6,6-Dimethyl-5,7,13,21-tetraoxatetracyclo[9.9.1.0^{2,10}.0^{4,8}]henicos-18-en-14-one **35** (17 mg, 0.051 mmol), was dissolved in THF (0.5 mL). HCl (0.100 mL, 1.00 mmol in H_2O) was added and the solution was stirred overnight at RT. The acid and ruthenium impurities from the previous step were removed by basic ion exchange chromatography to yield **36** as an

oil (13 mg, 88%). $[\alpha]^{24}_{D}$ -25.6 (c=1.0 $CHCl_3$); IR (film) 3431, 2929, 1732, 1441 cm^{-1}; ^{1}H NMR (500 MHz, $CDCl_3$) δ 5.42-5.34 (2H, m, H-8, H-9), 5.16 (2H, dd, *J*=2.0, 12.0, Ha-2), 3.91-3.79 (3H, m, H-11, H-14, H-15), 3.79-3.76 (1H, m, H-1), 3.49 (1H, dd, *J*=1.0, 12.5, Hb-2), 2.54-2.47 (1H, m, H-17), 2.49-2.31 (4H, m, Ha-5, Ha-6, Ha-10, H-12), 2.17-2.11 (2H, m, Hb-5, Hb-6), 2.12-1.88 (4H, m, Ha-7, Ha-13, Ha-16, Hb-10), 1.72-1.56 (3H, m, Hb-7, Hb-13, Hb-16); ^{13}C NMR (125 MHz, $CDCl_3$) δ 173.8 (C4), 133.9 (C8), 125.3 (C9), 80.8 (C14 or C15), 80.3 (C14 or C15), 68.9 (C1 or C11), 68.4 (C1 or C11), 62.8 (C2), 37.4 (C12), 36.2 (C17), 34.6 (C5 or C6 or C10), 33.9 (C5 or C6 or C10), 33.6 (C5 or C6 or C10), 30.6 (C7 or C13 or C16), 28.6 (C7 or C13 or C16), 22.5 (C7 or C13 or C16); LC-MS (ESI^+) m/z 319 [$M+Na^+$], R_t 1.58 min [method D]; HRMS [$M+Na^+$] calcd for $C_{16}H_{24}O_5Na$ 319.1521 found 319.1518.

(3S,3aS,7aR)-3-(((tert-butyldiphenylsilyl)oxy)methyl)-5-methyl-3a,4,7,7a-tetrahydroisobenzofuran-1(3H)-one (37 major) and (3S,3aS,7aR)-3-(((tert-butyldiphenylsilyl)oxy)methyl)-6-methyl-3a,4,7,7a-tetrahydroisobenzofuran-1(3H)-one (37 minor)

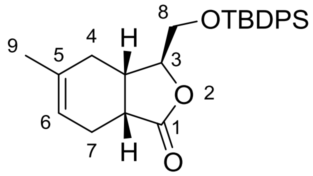

37 major

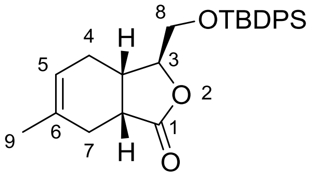

37 minor

Trifluoromethanesulfonamide (465 mg, 3.13 mmol) was dissolved in CH_2Cl_2 (3.0 mL) in a large pressure tube. Dimethylaluminium chloride (6.3 mL, 6.30 mmol) was added slowly under argon and the solution stirred for 30 min. *(S)*-5-((*tert*-Butyldiphenylsilyloxy)methyl)furan-2-(5*H*)one **8** (1.00 g, 2.841 mmol) and isoprene (2.84 mL, 28.41 mmol) were added and the tube was sealed and heated to 60 °C for 2 d. The reaction was cooled and diluted with Et_2O (100 mL). The mixture was quenched with NaOH (70 mL, 1M aq soln) and gas was evolved. The aqueous layer was extracted into Et_2O (100 mL x 2) washed with brine (100 mL x 1) and dried over $MgSO_4$. The solvent was removed *in vacuo*. The crude mixture was purified by column chromatography on silica gel (10% EtOAc:hexane) to yield an inseparable mixture of products as a clear oil (1.190 g, 100%). $[\alpha]^{24}_{D}$ +4.5 (c=1.0 $CHCl_3$); IR ($CDCl_3$ solution cell) 2929, 1769, 1427, 1113 cm^{-1}; ^{1}H NMR (500 MHz, $CDCl_3$) δ 7.66-7.64 (4H, m, Ph), 7.49-7.40 (6H, m, Ph), 5.56-5.51 (0.85H, m, H-6 major), 5.50-5.46 (0.15H, m, H-5 minor), 4.15-4.10 (1H, m, H-3), 3.89-3.84 (1H, m, Ha-8), 3.80-3.75 (1H, m, Hb-8), 3.03 (0.15H, ddd, *J*=4.0, 8.5, 8.5, H-7a minor), 2.93 (0.85H, m, ddd, *J*=4.0, 8.5, 8.5, H-7a major), 2.77-2.70 (0.85H, m, H-3a major), 2.68-2.63 (0.15H, m, H-3a minor), 2.44-2.37 (1H, m, Ha-7), 2.35-2.27 (1H, m, Hb-7), 2.21-2.17 (0.15H, m, Ha-4 minor), 2.16 (0.85H, dd, *J*=7.5, 16.0, Ha-4 major), 1.95-1.88 (0.15H, m, Hb-4 minor), 1.85 (0.85H, dd, *J*=6.5, 16.5, Hb-4 major), 1.74 (0.45H, br.s, H-9 minor), 1.71 (2.55H, br.s, H-9 major), 1.09 (1.5H, s, ^{t}Bu), 1.07 (7.5H, s, ^{t}Bu); ^{13}C NMR (125 MHz, $CDCl_3$) δ 184.5 (C1), 135.6 (Ph), 135.5 (Ph), 134.7, 133.2, 132.9, 132.6, 129.9, 129.6, 127.8, 127.7, 119.8, 119.2, 110.7 (C6 major),

110.2 (C5 minor), 84.7 (C3), 64.4 (C8), 37.2 (C7a), 34.8 (C3a), 30.7 (C4), 26.6 (^{t}Bu major), 26.5 (^{t}Bu minor), 23.7 (C9 major), 23.1 (C7), 22.6 (C9 minor), 14.0 (Si-C); LC-MS (ESI^+) *m/z* 443 [$M+Na^+$], R_t 2.70 min [method D].

(3S, 3aS, 5R, 6R, 7aR)-3-(*tert*-Butyldiphenylsilanyloxymethyl)-6-hydroxy-5-methylhexahydroisobenzofuran-1-one 38

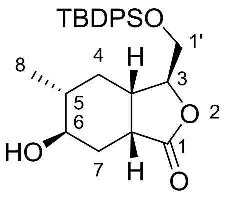

38

(3S,3aS,7aR)-3-(((*tert*-Butyldiphenylsilyl)oxy)methyl)-5-methyl-3a,4,7,7a-tetrahydroisobenzofuran-1(3H)-one **37** (1.17 g, 2.66 mmol) was dissolved in THF (26 mL), and cooled to -78°C. BH_3.THF (5.3 mL, 5.3 mmol, 1.0 M solution in THF) was added dropwise and stirred at 0°C for 2 h. The reaction was quenched slowly with H_2O (6 mL). NaOH (6 mL, 20% aqueous solution) was then added dropwise, followed by H_2O_2 (6 mL, 30% aqueous solution). The mixture was left to stir for 30 min. Sat. sodium sulphite (40 mL) was then added, and the layers separated. The aqueous layer was extracted with Et_2O (100 mL x 3). The combined organic layers were dried over $MgSO_4$ and the solvent removed *in vacuo* to yield the crude product as a clear oil. The crude reaction mixture was purified by column chromatography on silica gel (33% EtOAc:hexane) to yield **38** as a white solid (612 mg, 55%). mp (117-120°C); $[\alpha]^{24}_D$ +8.2 (c=1.0 $CHCl_3$); IR (film) 3421, 2932, 2859, 1772, 1428 cm^{-1}; ^{1}H NMR (500 MHz, $CDCl_3$) δ 7.66-7.63 (4H, m, Ph), 7.48-7.39 (6H, m, Ph), 4.05 (1H, dd, *J*=4.5, 4.5, H-3), 3.81 (1H, dd, *J*=4.5, 11.0, Ha-1'), 3.75 (1H, dd, *J*=4.0, 11.5, Hb-1'), 3.15 (1H, ddd, *J*=5.0, 10.5, 10.5, H-6), 3.09 (1H, dd, *J*=7.0, 7.0, H-7a), 2.49-2.40 (2H, m, Ha-4, Ha-7), 1.88-1.82 (1H, m, Hb-4), 1.54 (1H, ddd, *J*=7.0, 11.0, 14.0, Hb-7), 1.37 (1H, qddd, *J*=6.5, 3.0, 9.5, 12.5, H-5), 1.11-1.04 (1H, m, H-3a), 1.06 (9H, s, ^{t}Bu), 1.01 (3H, d, *J*=6.5, H-8); ^{13}C NMR (125 MHz, $CDCl_3$) δ 177.9 (C1), 135.7 (2 x Ph), 135.6 (2 x Ph), 132.7 (iPh), 132.5 (iPh), 130.0 (2 x Ph), 127.9 (2 x Ph), 127.7 (2 x Ph), 83.3 (C3), 72.5 (C6), 64.4 (C8), 39.3 (C7a), 37.2 (C5), 36.5 (C4), 36.4 (C3a), 31.7 (C7), 26.8 (tBu), 19.1 (Si-C), 17.9 (C8); LC-MS (ESI^+) *m/z* 461 [$M+Na^+$], R_t 2.95 min [method D]; HRMS [$M+Na^+$] calcd for $C_{26}H_{34}O_4SiNa$ 461.2119 found 461.2116; Found: C,71.23; H, 7.75%; $C_{26}H_{34}O_4Si$ requires: C, 71.19; H, 7.81%.

(1S,8E,11S,12R,14R,15R,17S)-14-hydroxy-15-methyl-3,18-dioxatricyclo[9.6.1.0^{12,17}]octadec-8-en-4- one 39

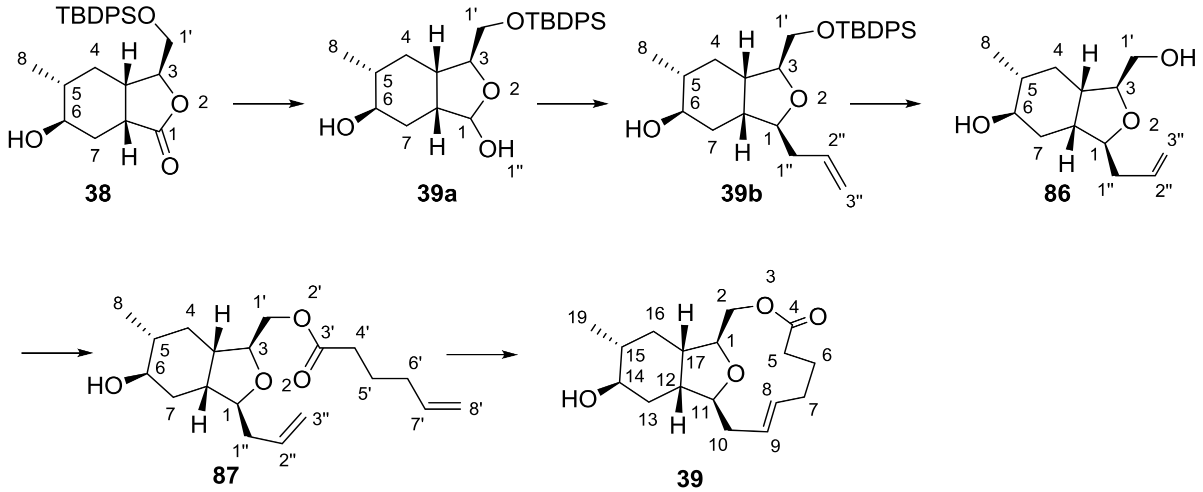

(3S, 3aS, 5R, 6R, 7aR)-3-(*tert*-Butyldiphenylsilanyloxymethyl)-6-hydroxy-5-methylhexahydroisobenzofuran-1-one **38** (612 mg, 1.397 mmol) was dissolved in anhydrous CH_2Cl_2 (13 mL) and cooled to -78 °C under N_2. DIBAL-H (1.0 M in toluene, 1.53 mL, 1.53 mmol) was added dropwise. The reaction was stirred at -78 °C for 4 h then quenched with EtOAc (40 mL). The solution was warmed to RT and a sat. aq. solution of Rochelles salt (40 mL) was added and the mixture was stirred overnight. The aqueous layer was separated and extracted with CH_2Cl_2 (3 x 50 mL). The organic layers were combined, dried over $MgSO_4$ and the solvent was removed *in vacuo* NMR analysis showed only 50% conversion. Complete conversation was observed after a further 3 iterations of the above conditions. The final work up yielded a clear oil **39a** (580 mg, 94%) as an inseparable mixture of anomers. IR (film) 3312, 2930, 1428 cm^{-1}; **MAJOR** ^1H NMR (500 MHz, $CDCl_3$) δ 7.71-7.66 (4H, m, Ph), 7.47-7.37 (6H, m, Ph), 5.21 (1H, dd, *J*=6.0, 7.0, H-1), 3.81-3.78 (1H, m, H-3), 3.73-3.55 (2H, m, H-1'), 3.37-3.31 (1H, m, H-6), 2.83 (1H, d, *J*=7.5, OH), 2.34-2.28 (1H, m, H-7a), 2.15-2.09 (1H, m, HA-7), 1.75-1.66 (1H, m, HA-4), 1.57-1.44 (1H, m, HB-7), 1.44-1.35 (2H, m, H-5, H-3a), 1.11-1.05 (1H, m, HB-4), 1.09 (9H, s, ^{t}Bu), 1.03 (3H, d, *J*=6.5, H-8); ^{13}C NMR (125 MHz, $CDCl_3$) δ 135.6 (2 x Ph), 135.6 (2 x Ph), 133.2 (iPh), 133.1 (iPh) 129.9 (2 x Ph), 127.9 (2 x Ph), 127.7 (2 x Ph), 102.1 (C1), 85.2 (C3), 72.7 (C6), 66.0 (C1''), 45.3 (C7a), 38.2 (C5), 37.8 (C3a), 35.3 (C4), 32.1 (C7), 26.8 (tBu), 19.2 (Si-C), 18.4 (C8); **MINOR** ^1H NMR (500 MHz, $CDCl_3$) δ 7.71-7.66 (4H, m, Ph), 7.47-7.37 (6H, m, Ph), 5.38 (1H, dd, *J*=3.0, 5.0, H-1), 4.00-3.97 (1H, m, H-3), 3.73-3.55 (3H, m, H-1', H-6), 2.61-2.59 (1H, m, OH), 2.46-2.40 (1H, m, H-7a), 2.27-2.21 (1H, m, H-3a), 2.08-2.03 (1H, m, Ha-7), 1.75-1.66 (1H, m, HA-4), 1.57-1.44 (1H, m, Hb-7), 1.44-1.35 (1H, m, H-5), 1.11-1.05 (1H, m, Hb-4), 1.06 (9H, s, tBu), 1.04 (3H, d, *J*=6.5, H-8); ^{13}C NMR (125 MHz, $CDCl_3$) δ 135.6 (2 x Ph), 135.6 (2 x Ph), 1.33 (iPh), 133.1 (iPh), 129.9 (2 x Ph), 127.9 (2 x Ph), 127.7 (2 x Ph), 101.1 (C1), 85.8 (C3), 73.5 (C6), 66.0 (C1'), 42.4 (C7a), 39.1 (C3a), 38.1 (C5), 35.3 (C4), 32.1 (C7), 26.8 (tBu), 19.2 (Si-C), 18.4 (C8); LC-MS (ESI$^+$) *m/z* 463 [M+Na$^+$], R_t 3.00 min [method D]; HRMS [M+Na$^+$] calcd 463.2275 for $C_{26}H_{36}O_4SiNa$ found 463.2279.

(3S,3aS,5R,6R,7aR)-3-((tert-Butyldiphenylsilyloxy)methyl)-5-methyloctahydroisobenzofuran-1,6-diol **39a** (569 mg, 1.293 mmol) was dissolved in CH_2Cl_2 (13 mL) and cooled to -78 °C. $BF_3.OEt_2$ (0.50 mL, 3.880 mmol) was added and the solution stirred for 5 min. Allyltrimethylsilane (0.63 mL, 3.880 mmol) was then added and the reaction warmed to RT over 18 h. The reaction was quenched with H_2O (20 mL) and extracted into CH_2Cl_2 (30 mL x 3). The combined organic layers were dried over $MgSO_4$. The solvent was removed *in vacuo* to yield **39b** as a clear oil (549 mg, 91%). $[\alpha]^{20}_{D}$ -16.4 (c=1.0 CH_2Cl_2); IR (film) 3384, 2929, 2885 1428 cm^{-1}; ^{1}H NMR (500 MHz, $CDCl_3$) δ 7.75-7.63 (4H, m, Ph), 7.48-7.37 (6H, m, Ph), 5.86 (1H, dddd, *J*=7.0, 7.0, 10.5, 17.5, H-2''), 5.11-5.02 (2H, m, H-3''), 3.85 (1H, ddd, *J*=4.0, 8.0, 10.5, H-1), 3.75 (1H, dd, *J*=4.0, 6.5, H-3), 3.60 (1H, dd, *J*=4.5, 10.5, Ha-1'), 3.50 (1H, dd, *J*=6.5, 10.5, Hb-1'), 3.28 (1H, dd, *J*=4.5, 10.5, 10.5, H-6), 2.39-2.32 (1H, m, Ha-1''), 2.24 (1H, ddd, *J*=6.5, 6.5, 12.5, H-3a), 2.18 (1H, ddd, *J*=7.0, 7.5, 14.5, Hb-1''), 2.10-2.04 (1H, m, H-7a), 1.93 (1H, ddd, *J*=2.0, 5.0, 14.0, Ha-7), 1.69 (1H, ddd, *J*=3.0, 5.5, 12.5, Ha-4), 1.55-1.49 (1H, m, Hb-7), 1.47-1.35 (1H, m, H-5), 1.29 (1H, ddd, *J*=12.5, 12.5, 12.5, Hb-4), 1.07 (9H, s, ^{t}Bu), 1.05 (3H, d, *J*=6.5, H-8); ^{13}C NMR (125 MHz, $CDCl_3$) δ 135.6 (4 x Ph), 135.4 (C2''), 133.7 (iPh), 133.6 (iPh), 129.7 (Ph), 129.6 (Ph), 127.9 (2 x Ph), 127.7 (2 x Ph), 116.8 (C3''), 84.6 (C3), 79.6 (C1), 72.6 (C6), 66.1 (C1'), 42.6 (C7a), 40.6 (C3a), 39.2 (C1''), 39.0 (C5), 36.3 (C4), 32.8 (C7), 26.8 (^{t}Bu), 19.2 (Si-C), 18.2 (C8); LC-MS (ESI^{+}) *m/z* 487 [M+Na^{+}], R_t 3.25 min [method D]; HRMS [M+Na^{+}] calcd for $C_{29}H_{40}O_3SiNa$ 487.2638 found 487.2647; Found: C, 74.87; H, 8.59%; $C_{29}H_{40}O_3Si$ requires: C, 74.95; H, 8.68%.

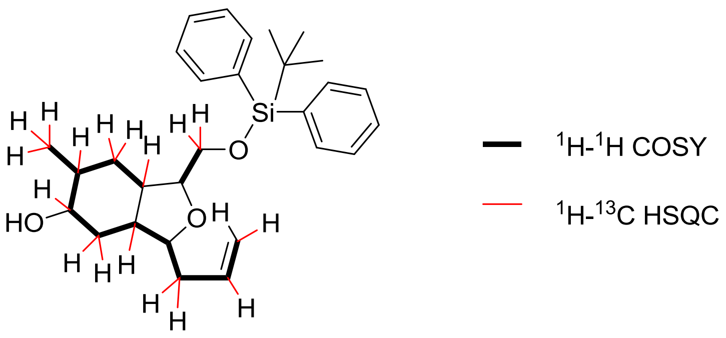

(1S,3S,3aR,5R,6R,7aS)-3-Allyl-1-((tertbutyldiphenylsilyloxy)methyl)-6-methyloctahydroisobenzofuran-5-ol **39b** (549 mg, 1.362 mmol) was dissolved in THF (13 mL). The solution was cooled to 0 °C under N_2 and TBAF (4.00 mL, 4.00 mmol, 1.0 M in THF) was added. The reaction was stirred for 4 h at 0 °C before diluting with Et_2O (50 mL). The reaction mixture was washed with H_2O (3 x 50 mL) and extracted into Et_2O (2 x 50 mL). The combined organic layers were washed with brine (50 mL) and dried over $MgSO_4$. The solvent was removed *in vacuo* to give the crude product. This was purified by column chromatography on silica gel (5% MeOH:CH_2CL_2) to yield **86** as a clear oil (160 mg, 52%). $[\alpha]^{20}_{D}$ –30.7 (c=1.0 CH_2Cl_2); IR (film) 3368, 2922, 1456, 1031 cm^{-1}; ^{1}H NMR (500 MHz, $CDCl_3$) δ 5.88 (1H, dddd, *J*=7.0, 7.0, 10.0, 17.0, H-2''), 5.15-5.09 (2H, m, H-3''), 3.92 (1H, ddd, *J*=4.0,

7.0, 10.5, H-1), 3.72-3.70 (1H, m, H-3), 3.53 (1H, dd, *J*=4.0, 11.0, Ha-1'), 3.43 (1H, dd, *J*=7.0, 11.5, Hb-1'), 3.31 (1H, ddd, *J*=4.5, 10.0, 10.0, H-6), 2.48-2.42 (1H, m, Ha-1''), 2.24-2.17 (1H, m, Hb-1''), 2.16-2.10 (1H, m, H-7a), 2.04 (1H, ddd, *J*=6.5, 6.5, 13.0, H-3a), 1.93 (1H, ddd, *J*=2.5, 4.5, 14.0, Ha-7), 1.72 (1H, ddd, *J*=3.0, 6.0, 14.0, Ha-4), 1.56 (1H, ddd, *J*=6.0, 11.0, 14.0, Hb-7), 1.38 (1H, dqdd, *J*=3.0, 6.5, 10.0, 13.0, H-5), 1.19 (1H, ddd, *J*=13.0, 13.0, 14.0, Hb-4), 1.04 (3H, d, *J*=6.5, H-8); ^{13}C NMR (125 MHz, $CDCl_3$) *δ* 134.5 (C2''), 117.5 (C3''), 84.5 (C3), 79.7 (C1), 72.4 (C6), 65.5 (C1'), 42.8 (C3a), 40.5 (C7a), 38.8 (C5), 38.7 (C1''), 36.0 (C4), 32.7 (C7), 18.2 (C8); LC-MS (ESI$^+$) *m/z* 271 [M+Na$^+$], R_t 2.55 min [method D].

(1S,3S,3aR,5R,6R,7aS)-3-Allyl-1-(hydroxymethyl)-6-methyloctahydroisobenzofuran-5-ol **86** (50 mg, 0.221 mmol) was dissolved in CH_2Cl_2 (1.2 mL). Hex-5-enoyl chloride (32 mg, 0.243 mmol) and triethylamine (95 µL, 0.663 mmol) were added and the reaction mixture was stirred at RT for 18 h. TLC analysis indicated the presence of starting material, therefore a further quantity of hex-5-enoyl chloride (32 mg, 0.243 mmol) was added and the solution was stirred for a further 5 h. The reaction mixture was diluted with CH_2Cl_2 (10 mL) and washed with H_2O (10 mL) and brine (10 mL). The aqueous layers were combined and extracted with CH_2Cl_2 (10 mL x 2). The combined organic layers were dried over $MgSO_4$ and the solvents removed *in vacuo*. Purification by column chromatography (33% EtOAc:hexane) gave **87** (30 mg, 42%) as a clear oil. $[\alpha]^{20}_D$ -25.0 (c=0.5, CH_2Cl_2); IR (film) 3444, 2924, 1438, 1642, 1455 cm^{-1}; ^{1}H NMR (500 MHz, $CDCl_3$) δ 5.87 (1H, dddd, *J*=7.0, 7.0, 10.0, 17.0, H-2''), 5.78 (1H, dddd, *J*=6.5, 6.5, 10.0, 17.0, H-7'), 5.13-4.98 (4H, m, H-3'', H-8'), 4.01 (1H, dd, *J*=6.0, 11.0, Ha-1'), 3.96 (1H, dd, *J*=5.5, 11.0, Hb-1'), 3.90 (1H, ddd, *J*=4.0, 7.0, 10.5, H-1), 3.82 (1H, dd, *J*=6.0, 6.0, H-3), 3.33 (1H, ddd, *J*=4.5, 10.5, 10.5, H-6), 2.45-2.39 (1H, m, Ha-1''), 2.39 (2H, t, *J*=7.5, H-4'), 2.23-2.03 (5H, m, Hb-1'', H-6', H-3a, H-7a), 1.96 (1H, ddd, *J*=2.0, 4.5, 14.0, Ha-7), 1.78-1.69 (3H, m, H-4A, Ha-5'), 1.56 (2H, ddd, *J*=7.0, 10.5, 14.0, H-7, OH), 1.39 (1H, dqdd, 3.0, 6.5, 9.5, 12.5, H-5), 1.20 (1H, ddd, *J*=14.0, 14.0, 14.0, HB -4), 1.03 (3H, d, *J*=6.5, H-8); ^{13}C NMR (125 MHz, $CDCl_3$) δ 173.4 (C3'), 137.6 (C7''), 134.7 (C2''), 117.2 (C3''), 115.4 (C8'), 81.6 (C3), 79.9 (C1), 72.3 (C6), 66.3 (C1'), 42.4 (C3a or C7a), 41.1 (C3a or C7a), 38.9 (C1''), 38.8 (C5), 35.9 (C4), 33.5 (C4'), 33.0 (C6'), 32.7 (C7), 24.0 (C5'), 18.0 (C8); LC-MS (ESI$^+$) *m/z* 345 [M+Na$^+$], R_t 2.62 min [method D]; HRMS [M+Na$^+$] calcd for $C_{19}H_{30}O_4Na$ 345.2036 found 345.2046; Found: C,70.73; H, 9.35%; $C_{19}H_{30}O_4$ requires: C, 70.77; H, 9.38%.

((1S,3S,3aR,5R,6R,7aS)-3-Allyl-5-hydroxy-6-methyloctahydroisobenzofuran-1-yl)methyl hex-5-enoate **87** (26 mg, 0.081 mmol) was dissolved in CH_2Cl_2 (160 mL, 0.0005 M). Argon was bubbled though the solution for 5 min. Grubbs II (7 mg, 0.008 mmol, 10 mol%) was added under argon and the reaction mixture was stirred for 3 d. DMSO (10 µL) was added and the solution was stirred overnight. The solvents were removed *in vacuo* and the resultant mixture purified by column chromatography on silica gel (15% EtOAc:hexane) to yield **39** as a clear oil (19 mg, 80%). $[\alpha]^{24}_D$ -20.8 (c=1.0 $CHCl_3$); IR (film) 3432, 2919, 1738, 1446 cm^{-1}; ^{1}H NMR (500 MHz, $CDCl_3$) *δ* 5.42-5-35 (2H, m, H-8, H-9), 5.13 (1H, dd, *J*=3.0, 12.0, Ha-2), 3.94 (1H, ddd, *J*=3.0, 3.0, 11.0, H-11), 3.69 (1H, m, H-1), 3.42 (1H, d, *J*=12.0, Hb-2), 3.30 (1H, ddd,

J=5.0, 11.0, 11.0, H-14), 2.49-2.34 (4H, m, Ha-7, Ha-10, H-12, Ha-5 or Ha-6), 2.28-2.22 (1H, m, H-17), 2.17-2.03 (3H, m, Hb-7, Hb-10, Ha-5 or Ha-6), 1.95-1.87 (2H, m, Ha-13, Hb-5 or Hb-6), 1.74-1.70 (1H, m, Ha-16), 1.71-1.57 (2H, m, Hb-13, Hb-5 or Hb-6), 1.45-1.38 (1H, m, Ha-15), 1.18 (1H, ddd, J=13.0, 13.0, 13.0, Hb-16), 1.03 (3H, d, J=6.5, H-19); ^{13}C NMR (125 MHz, $CDCl_3$) δ 173.9 (C4), 133.1 (C8), 125.8 (C9), 81.9 (C1), 79.2 (C11), 72.5 (C14), 64.4 (C2), 41.5 (C17), 39.3 (C12), 38.9 (C15), 36.8 (C16), 33.8 (C5 or C6), 33.0 (C7), 32.3 (C10 or C13), 32.3 (C10 or C13), 21.8 (C5 or C6), 18.1 (C19); LC-MS (ESI$^+$) *m/z* 317 [M+Na$^+$], R_t 1.90 min [method D]; HRMS [M+Na$^+$] calcd for $C_{17}H_{26}O_4Na$ 317.1723 found 317.1726.

(1R,2S,3S,10E,13S,14R,15S)-5,20-dioxatetracyclo[13.2.2.1^{3,13}.0^{2,14}]icosa-10,16-dien-6-one 40

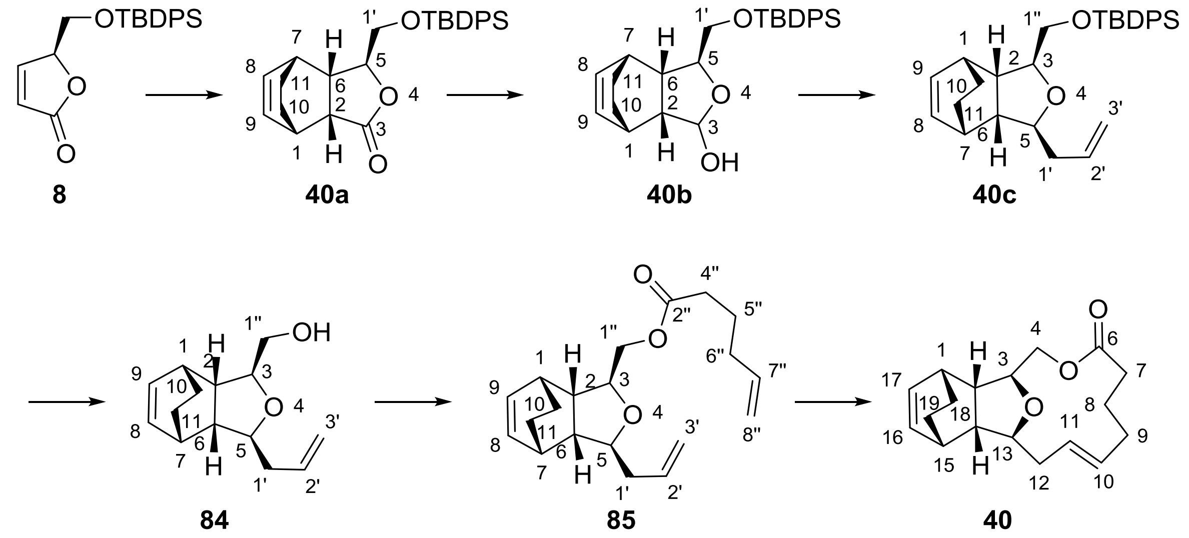

Trifluoromethanesulfonamide (465 mg, 3.12 mmol) was dissolved in CH_2Cl_2 (3.0 mL) in a pressure tube. Dimethylaluminium chloride (6.2 mL, 6.2 mmol) was added slowly under argon and the solution stirred for 30 min. *(S)*-5-((*tert*-Butyldiphenylsilyloxy)methyl)furan-2-(5*H*)one **8** (1.00 g, 2.841 mmol) and cyclohexadiene (2.7 mL, 28.4 mmol) were added and the tube was sealed and heated to 60 °C for 2 d. The reaction was cooled and diluted with Et_2O (100 mL), the mixture was quenched with NaOH (70 mL, 1M aq sol) and gas was evolved. The aqueous layer was extracted into Et_2O (100 mL x 2) washed with brine (100 mL x 1) and dried over $MgSO_4$. The solvent was removed *in vacuo*. The crude mixture was purified by column chromatography on silica gel (10% EtOAc:hexane) to yield **40a** a clear oil (930 mg, 75%). $[\alpha]^{23.7}_D$ +10.0 (c=1.0 $CHCl_3$); IR (film) 2934, 1767 (C=O) cm^{-1}; ^{1}H NMR (500 MHz, $CDCl_3$) δ 7.69-7.64 (4H, m, Ph), 7.48-7.39 (6H, m, Ph), 6.35-6.31 (1H, m, H-9), 6.27-6.23 (1H, m, H-8), 4.04 (1H, dd, J=3.5, 6.5, H-5), 3.80 (1H, dd, J=4.0, 11.0, Ha-1'), 3.67 (1H, dd, J=3.0, 11.0, Hb-1'), 3.10-3.06 (1H, m, H-1), 2.83 (1H, dd, J=3.5, 10.0, H-2), 2.67-2.63 (1H, m, H-7), 2.58 (1H, ddd, J=3.5, 3.5, 10.0, H-6), 1.62-1.56 (1H, m, Ha-10), 1.52-1.57 (1H, m, Ha-11), 1.35 (1H, dddd, J=3.0, 3.0, 12.0, 12.0, Hb-10), 1.30 (1H, dddd, J=3.0, 3.0, 12.0, 12.0, Hb-

11), 1.07 (9H, s, ^{t}Bu); ^{13}C NMR (125 MHz, $CDCl_3$) δ 178.8 (C3), 135.7 (2 x Ph), 135.6 (2 x Ph), 134.2 (C9), 133.2 (iPh), 132.7 (C8), 132.6 (iPh) 129.9 (2 x Ph), 127.8 (2 x Ph), 127.8 (2 x Ph), 84.3 (C5), 65.4 (C1'), 46.3 (C2), 41.0 (C6), 33.3 (C7), 31.7 (C1), 26.7 (^{t}Bu), 23.7 (C10), 23.3 (C11), 19.2 (Si-C); LC-MS (ESI$^+$) *m/z* 455 [M+Na$^+$], R_t 2.68 min [method D]; HRMS [M+Na$^+$] calcd for $C_{27}H_{32}O_3SiNa$ 455.2013 found 455.2010; Found: C, 74.90; H, 7.42%; $C_{27}H_{32}O_3Si$ requires: C, 74.96; H, 7.49%.

(1S,2R,5S,6S,7R)-5-(*tert*-Butyldiphenylsilanyloxymethyl)-4-oxatricyclo[5.2.2.0^{2,6}]undec-8-en-3-one **40a** (481 mg, 1.113 mmol) was dissolved in anhydrous CH_2Cl_2 (5.6 mL) and cooled to -78 °C under N_2. DIBAL-H (1.0 M in toluene, 1.23 mL, 1.23 mmol) was added dropwise. The reaction was stirred at -78 °C for 3 h then quenched with EtOAc (10 mL). The solution was warmed to RT and a sat. aq. solution of Rochelles salt (6 mL) was added and the mixture stirred overnight. The aqueous layer was separated and extracted with CH_2Cl_2 (20 mL x 3). The organic layers were combined, dried over $MgSO_4$ and the solvent was removed *in vacuo* The crude mixture was purified by column chromatography on silica gel (15% EtOAc:hexane) to yield a clear oil **40b** (530 mg, 86%) as the kinetic anomer which converts to a mixture of anomers overnight in $CDCl_3$. $[\alpha]^{23.7}_{D}$ -15.6 (c=1.0 $CHCl_3$); IR (film) 3411, 2933, 1472, 1427 cm^{-1}; ^{1}H NMR (500 MHz, $CDCl_3$) δ **Anomer A** 7.74-7.66 (4H, m, Ph), 7.49-7.36 (6H, m, Ph), 6.28-6.20 (2H, m, H-9, H-8), 5.01 (1H, s, H-3), 3.82-3.76 (2H, m, H-5, Ha-1'), 3.58 (1H, dd, *J*=2.0, 10.0, Hb-1'), 2.79-2.75 (1H, m, H-1), 2.53-2.48 (1H, m, H-7), 2.41-2.38 (2H, m, H-2, H-6), 1.56-1.14 (4H, m, H-10, H-11), 1.12 (9H, s, tBu); **Anomer B** 7.74-7.66 (4H, m, Ph), 7.49-7.36 (6H, m, Ph), 6.25-6.18 (1H, m, H-9 or H-8), 6.11-6.08 (1H, m, H-9 or H-8), 4.80 (1H, d, *J*=2.0, H-3), 3.74-3.67 (2H, m, H-5, Ha-1'), 3.61-3.52 (1H, m, Hb-1'), 2.57-2.53 (1H, m, H-1 or H-7), 2.44-2.41 (1H, m, H-1 or H-7), 2.34 (1H, ddd, *J*=3.5, 3.5, 10.0, H-6), 2.14 (1H, ddd, *J*=2.0, 3.5, 10.0 H-2), 1.56-1.14 (4H, m, H-10, H-11), 1.09 (9H, s, tBu); ^{13}C NMR (125 MHz, $CDCl_3$) δ 135.8 (2 x Ph A or B), 135.7 (2 x Ph A or B), 135.6 (2 x Ph A or B), 135.5 (2 x Ph A or B), 133.8 (iPh A or B), 133.8 (iPh A or B), 133.4 (C8 or C9, A or B), 133.2 (C8 or C9, A or B), 133.2 (C8 or C9, A or B), 133.0 (C8 or C9, A or B), 130.0 (Ph A or B), 129.9 (Ph A or B), 129.6 (Ph A or B), 127.8 (Ph A or B), 127.6 (Ph A or B), 127.6 (Ph A or B), 105.7 (C3B), 104.6 (C3A), 86.5 (C5A), 85.6 (C5A), 67.3 (C1' A or B), 67.1 (C1' A or B), 55.5 (C6A or C2A), 52.2 (C6B or C2B), 47.3 (C6B or C2B), 46.1 (C6A or C2A), 33.9 (C1 or C7, A or B), 33.9 (C1 or C7, A or B), 32.3 (C1A or C7A), 31.5 (C1A or C7A), 27.0 (tBu A or B), 26.9 (tBu A or B), 24.5 (C10 or C11, A or B), 24.5 (C10 or C11, A or B), 23.9 (C10 or C11, A or B), 19.3 (Si-C, A or B), 19.2 (Si-C, A or B); LC-MS (ESI$^+$) *m/z* 457 [M+Na$^+$], R_t 2.78 min [method D]; HRMS [M+Na$^+$] calcd for $C_{27}H_{34}O_3SiNa$ 457.2169 found 457.2167; Found: C, 74.57; H, 7.92%; $C_{27}H_{34}O_3Si$ requires: C, 74.57; H, 7.92%.

(1S,2R,5S,6S,7R)-5-(*tert*-Butyldiphenylsilanyloxymethyl)-4-oxa-tricyclo[5.2.2.0^{2,6}]undec-8-en-3-ol **40b** (74 mg, 0.734 mmol) was dissolved in CH_2Cl_2 (2.0 mL) and cooled to -78 °C. $BF_3.OEt_2$ (0.065 mL, 0.51 mmol) was added by syringe and the solution stirred for 5 min. Allyltrimethylsilane (0.083 mL, 0.51 mmol) was added by syringe and the reaction then warmed to RT over 18 h. The reaction was then quenched with H_2O (6 mL) and extracted

into CH_2Cl_2 (6 mL x 3). The combined organic layers were dried over $MgSO_4$. The solvent was removed in vacuo to yield **40c** as a clear oil (60 mg, 76%). $[\alpha]^{23.7}_D$ +5.5 (c=1.0 CH_2Cl_2); IR (film) 3046, 2932, 2860, 1472, 1428 cm^{-1}; ^1H NMR (500 MHz, $CDCl_3$) δ 7.76-7.71 (4H, m, Ph), 7.47-7.38 (6H, m, Ph), 6.22-6.19 (2H, m, H-8, H-9), 5.88 (1H, dddd, *J*=7.0, 7.0, 10.0, 17.0, H-2'), 5.15-5.05 (2H, m, H-3'), 3.80 (1H, dd, *J*=5.0, 10.5, Ha-1''), 3.70 (1H, dd, *J*=5.0, 10.5, Hb-1''), 3.60 (1H, ddd, *J*=5.0, 5.0, 8.0, H-3), 3.55 (1H, ddd, *J*=6.0, 6.0, 8.0, H-5), 2.55-2.49 (2H, m, H-1, H-7), 2.41-2.28 (2H, m, H-1''), 2.25 (1H, ddd, *J*=3.0, 8.0, 11.0, H-2), 2.05 (1H, ddd, *J*=3.0, 8.0, 11.0, H-6), 1.50-1.42 (2H, m, Ha-10, Ha-11), 1.34-1.26 (2H, m, Hb-10, Hb-11), 1.10 (9H, s, ^tBu); ^{13}C NMR (125 MHz, $CDCl_3$) δ 135.7 (2 x Ph), 135.7 (2 x Ph), 135.3 (C2'), 134.3 (C8 or C9), 134.1 (C8 or C9), 133.8 (iPh), 133.8 (iPh), 129.5 (2 x Ph), 127.6 (4 x Ph), 116.5 (C3'), 83.6 (C3), 82.9 (C5), 66.3 (C1''), 51.9 (C6), 49.6 (C2), 39.8 (C1'), 32.1 (C1 or C7), 31.9 (C1 or C7), 26.9 (^tBu), 24.6 (C10 or C11), 24.6 (C10 or C11), 19.3 (Si-C); LC-MS (ESI^+) *m/z* 481 [$M+Na^+$], R_t 3.35 min [method E]; HRMS [$M+Na^+$] calcd for $C_{30}H_{38}O_2SiNa$ 481.2533 found 481.2547; Found: C,78.56; H, 8.29%; $C_{30}H_{38}O_2Si$ requires: C, 78.55; H, 8.35%.

((1R, 2S, 3S, 5S, 6R, 7S)-5-allyl-4-oxatricyclo[5.2.2.0$^{2.6}$]undec-8-en-3-ylmethoxy)*tert*butyldiphenylsilane **40c** (969 mg, 2.173 mmol) was dissolved in THF (22 mL). The solution was cooled to 0 °C under N_2 and TBAF (6.5 mL, 6.5 mmol, 1.0 M in THF) was added by syringe. The reaction was stirred for 3.5 h at 0 °C before diluting with Et_2O (50 mL). The reaction mixture was washed with H_2O (3 x 50 mL) and extracted into Et_2O (3 x 50 mL). The combined organic layers were dried over $MgSO_4$ and the solvent was removed *in vacuo* to give the crude product. This was purified by column chromatography on silica gel (50% EtOAc:hexane) to yield **84** as a clear oil (332 mg, 72%). $[\alpha]^{23.7}_D$ -23.5 (c=1.0 $CHCl_3$); IR (film) 3431, 2936 cm^{-1}; ^1H NMR (500 MHz, $CDCl_3$) δ 6.22-6.16 (2H, m, H-8, H-9), 5.84 (1H, dddd, *J*=7.0, 7.0, 10.0, 17.0, H-2'), 5.14-5.04 (2H, m, H-3'), 3.79-3.73 (1H, m, Ha-1''), 3.58-3.51 (3H, m, Hb-1'', H-3, H-5), 2.52-2.45 (2H, m, H-1, H-7), 2.39-2.27 (3H, m, H-1', OH), 2.25-2.19 (1H, m, H-2), 2.08-1.98 (1H, m, H-6), 1.49-1.38 (2H, m, Ha-10, Ha-11), 1.34-1.24 (2H, m, Hb-10, Hb-11); ^{13}C NMR (125 MHz, $CDCl_3$) δ 134.8 (C2'), 134.2 (C8 or C9), 134.1 (C8 or C9), 116.9 (C3'), 83.9 (C3 or C5), 83.0 (C3 or C5), 64.0 (C1''), 52.0 (C6), 47.6 (C2), 39.4 (C1'), 31.8 (C1 or C7), 31.6 (C1 or C7), 24.5 (C10 or C11), 24.5 (C10 or C11); LC-MS (ESI^+) *m/z* 243.24 [$M+Na^+$], R_t 2.22 min [method D]; HRMS [$M+H^+$] calcd for $C_{14}H_{21}O_2$ 221.1536 found 221.1538; Found: C,76.21; H, 9.15%; $C_{14}H_{20}O_2$ requires: C, 76.33; H, 9.15%.

((1R, 2S, 3S, 5S, 6R, 7S)-5-Allyl-4-oxatricyclo[5.2.2.0$^{2.6}$]undec-8-en-3-yl)-methanol **84** (82 mg, 0.372 mmol) was dissolved in CH_2Cl_2 (3.0 mL). Hex-5-enoyl chloride (152 mg, 1.112 mmol) and triethylamine (160 µL, 1.112 mmol) were added and the reaction mixture was stirred at RT for 36 h. The reaction mixture was diluted with CH_2Cl_2 (10 mL) and washed with H_2O (10 mL x 2) and brine (10 mL). The aqueous layers were combined and extracted with CH_2Cl_2 (10 mL x 2). The combined organic layers were washed with brine (10 mL), dried with $MgSO_4$ and the solvents removed *in vacuo*. The crude reaction mixture was purified by column chromatography on silica gel (10% EtOAc:hexane) to yield **85** as a clear oil (81 mg, 70%).

$[\alpha]^{23.7}_D$ +3.4 (c=1.0 $CHCl_2$); IR (film) 2938, 2868, 1737 cm^{-1}; ^1H NMR (500 MHz, $CDCl_3$) δ 6.23-6.18 (2H, m, H-9, H-8), 5.89-5.75 (2H, m, H-2', H-7''), 5.14-4.94 (4H, m, H-3', H-8''), 4.25 (1H, dd, *J*=3.5, 11.5, Ha-1''), 4.03 (1H, dd, *J*=7.0, 11.5, Hb-1''), 3.66 (1H, ddd, *J*=3.5, 7.0, 7.0, H-3), 3.55 (1H, ddd, *J*=6.5, 6.5, 6.5, H-5), 2.55-2.49 (2H, m, H-1, H-7), 2.45-2.34 (2H, m, H-1, H-7), 2.34-2.27 (4H, m, H-1', H-4''), 2.14-2.04 (4H, m, H-2, H-6, H-6''), 1.78-1.73 (2H, m, H-5''), 1.48-1.43 (2H, m, Ha-10, Ha-11), 1.36-1.25 (2H, m, Hb-10, Hb-11), ^{13}C NMR (125 MHz, $CDCl_3$) δ 173.6 (C2''), 137.2 (C2' or C7''), 134.7 (C2' or C7''), 134.5 (C8 or C9), 133.9 (C8 or C9), 116.9 (C8'' or C3'), 115.3 (C8'' or C3'), 83.2 (C5), 80.9 (C3), 66.1 (C1''), 51.4 (C2 or C6), 49.3 (C2 or C6), 39.4 (C1'), 33.5 (C4''), 33.0 (C6''), 31.8 (C1 or C6), 31.6 (C1 or C6), 24.5 (C10 or C11), 24.5 (C10 or C11), 24.0 (C5''); LC-MS (ESI^+) *m/z* 339 [$M+Na^+$], R_t 3.05 min [method D]; HRMS [$M+H^+$] calcd for $C_{20}H_{29}O_3$ 317.2111 found 317.2107.

(1R,2S,3S,5S,6R,7S)-5-Allyl-4-oxatricyclo[5.2.2.$0^{2,6}$]undec-8-en-3-ylmethyl hex-5-enoate **85** (30 mg, 0.095 mmol) was dissolved in CH_2Cl_2 (94 mL, 0.001 M). Argon was bubbled though the solution for 5 min. Grubbs II (8 mg, 0.0095 mmol, 10 mol%) was added under argon and the reaction mixture was stirred for 5 d. DMSO (10 µL) was added and the solution was stirred overnight. The solvents were removed *in vacuo* and the resultant mixture purified by column chromatography on silica gel (15% EtOAc:hexane) to yield **40** as a crystalline solid (14 mg, 51%). mp (129-134 ˚C); $[\alpha]^{24}_D$ -60.2 (c=1.0 $CHCl_3$); IR (film) 2930, 2865, 1738, 1433 cm^{-1}; ^1H NMR (500 MHz, $CDCl_3$) δ 6.28-6.14 (2H, m, H-17, H-16), 5.46 (1H, dddd, *J*=1.5, 4.5, 10.0, 15.0, H-11), 5.37 (1H, dddd, *J*=1.0, 4.0, 11.0, 15.0, H-10), 5.18 (1H, dd, *J*=2.0, 11.5, Ha-4), 3.67-3.60 (2H, m, H-3, H-13), 3.44 (1H, d, *J*=11.5, Hb-4), 2.54-2.48 (3H, m, H-2, H-14, H-1 or H-15), 2.45-2.33 (3H, m, Ha-12, Ha-9, Ha-7), 2.29 (1H, ddd, *J*=3.0, 7.5, 10.5, H-1 or H-15), 2.12-2.01 (3H, m, Ha-8, Hb-7, Hb-12), 1.90-1.81 (1H, m, Hb-9), 1.67-1.60 (1H, m, Hb-8), 1.55-1.47 (2H, m, H-19 or H-18), 1.35-1.29 (2H, m, H-19 or H-18); ^{13}C NMR (125 MHz, $CDCl_3$) δ 173.9 (C6), 134.7 (C16 or C17 or C10), 134.6 (C16 or C17 or C10), 134.1 (C16 or C17 or C10), 124.4 (C11), 82.7 (C3 or C13), 82.1 (C3 or C13), 61.3 (C4), 48.6 (C1 or C15), 47.8 (C1 or C15), 34.9 (C7 or C12 or C9), 34.7 (C7 or C12 or C9), 33.6 (C7 or C8 or C9), 32.1 (C2 or C14), 31.7 (C2 or C14), 24.6 (C18 or C19), 24.3 (C18 or C19), 22.4 (C8); LC-MS (ESI^+) *m/z* 311 [$M+Na^+$], R_t 2.87 min [method D]; HRMS [$M+H^+$] calcd for $C_{17}H_{25}O_3$ 289.1804 found 289.1798; Found: C,73.81; H, 8.63%; $C_{18}H_{24}O_3$ requires: C, 73.88; H, 8.75%.

(1R,7E/R,10S,11R,12R,15S,16S)-2,17-dioxatetracyclo[8.6.1.$1^{12,15}$.$0^{11,16}$]octadec-7-en-3-one 41

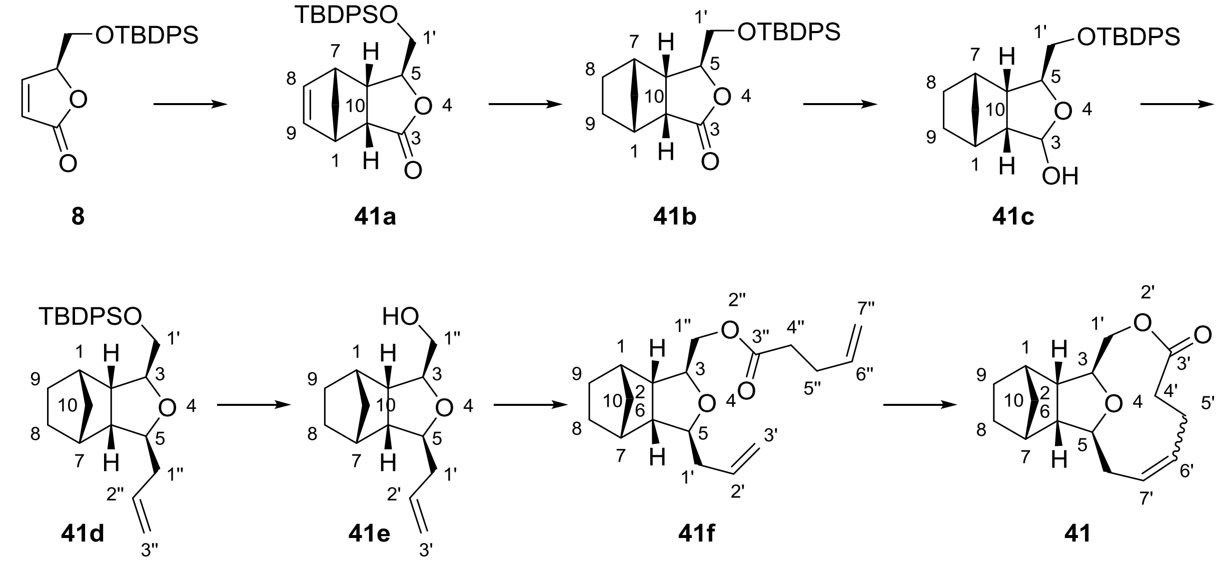

Dicyclopentadiene was cracked by heating to 170 °C and distillation of the monomer product. *(S)*-5-((*tert*-Butyldiphenylsilyloxy)methyl)furan-2-(5*H*)-one **8** (500 mg, 1.433 mmol) was weighed into a pressure tube, and cyclopentadiene (4.6 mL, 56.0 mmol) was added. The tube was sealed and heated to 110 °C for 3 d. The pressure tube was then cooled to 0 °C to reduce excess pressure, and the reaction was diluted in CH_2Cl_2 (10 mL). Solvent and unreacted cyclopentadiene were removed *in vacuo*. Non-polar side-products were removed by loading the crude reaction mixture onto a plug of silica and washing with hexane. The products were then washed though the silica with EtOAc and concentrated *in vacuo*. The products were then separated by column chromatography (25% EtOAc:hexane) to give the *endo* product **41a** as a white crystalline solid (305 mg, 69%) and the *exo* product as a clear oil (116 mg, 16%). *Endo* Product **41a** $[\alpha]^{23}_{D}$ -17.3 (c=2.78, CH_2Cl_2); IR (film) 3070, 2931, 2857, 1770 (CO), 1427 cm^{-1}; ^{1}H NMR (500 MHz, $CDCl_3$) δ 7.70-7.65 (4H, m, Ph), 7.50-7.38 (6H, m, Ph), 6.32 (1H, dd, *J*=3.0, 6.0, H-9), 6.23 (1H, dd, *J*=3.0, 6.5, H-8), 3.97 (1H, dd, *J*=3.0, 6.0, H-5), 3.79 (1H, dd, *J*=3.5, 11.0, Ha-1'), 3.75 (1H, dd, 3.0, 11.0, Hb-1'), 3.50-3.35 (2H, m, H-7, H-2), 3.10-3.07 (1H, m, H-6), 3.05-3.01 (1H, m, H-1), 1.66 (1H, d, *J*=8.5, Ha-10), 1.46 (1H, d, *J*=8.5, Hb-10), 1.07 (9H, s, ^{t}Bu); ^{13}C NMR (125 MHz, $CDCl_3$) δ 177.8 (C3), 137.7 (C8), 137.3 (C9), 135.6 (Ph), 135.5 (Ph), 135.2 (Ph), 134.7 (Ph), 133.2 (^{i}Ph), 132.6 (^{i}Ph), 129.9 (Ph), 129.6 (Ph), 127.8 (2 x Ph), 127.8 (2 x Ph), 82.1 (C5), 65.7 (C1'), 51.7 (C10), 48.8 (C2), 45.9 (C1), 45.7 (C7), 43.1 (C6), 26.9 (tBu), 19.2 (Si-C); LC-MS (ESI$^+$) *m/z* 441 [M+Na$^+$], R_t 5.80 min [method B]; HRMS [M+Na$^+$] calcd for $C_{26}H_{30}O_3SiNa$ 441.1856; found 441.1857. Found: C, 74.58; H, 7.23%, requires: C, 74.60; H, 7.22%. *Exo* Product $[\alpha]^{23}_{D}$ -0.61 (c=2.14, CH_2Cl_2); IR (film) 3070, 3049, 2930, 2857, 1745 (CO), 1428 cm^{-1}; ^{1}H NMR (500 MHz, $CDCl_3$) δ 7.70-7.65 (4H, m, Ph), 7.48-7.38 (6H, m, Ph), 6.26 (1H, dd, *J*=3.0, 6.0, H-9), 6.19 (1H, dd, *J*=3.0, 6.0, H-8), 4.20 (1H, dd, *J*=3.0, 3.0, H-5), 3.84 (1H, dd, *J*=3.5, 11.0, Ha-1'), 3.75 (1H, dd, 3.5, 11.0, Hb-1'), 3.29 (1H, m, H-1), 2.88 (1H, m, H-7), 2.72 (1H, d, *J*=8.5, H-2), 2.46 (1H, dd, *J*=3, 8.5, H-6) 1.53 (2H, m, H-10), 1.05 (9H, s, ^{t}Bu); ^{13}C NMR (125 MHz, $CDCl_3$) δ 177.4 (C1), 137.8 (C8), 137.5 (C9), 135.7 (Ph), 135.6 (Ph), 135.2 (Ph), 134.8 (Ph), 133.1 (^{i}Ph), 132.5 (^{i}Ph), 129.9 (Ph), 129.9 (Ph), 127.8

(2 x Ph), 127.7 (2 x Ph), 83.3 (C5), 65.7 (C1’), 49.1 (C2), 47.6 (C7), 46.6 (C1), 44.8 (C6), 43.6 (C10), 26.7 (^{t}Bu), 19.1 (Si-C); LC-MS (ESI$^+$) m/z 441 [M+Na$^+$], Rt 5.80 min [method B]; HRMS [M+Na$^+$] $C_{26}H_{30}O_3SiNa$ calcd for 441.1856; found 441.1857.

(1*S*,2*R*,5*S*,6*S*,7*R*)-5-(*tert*-Butyldiphenylsilanoxymethyl)-4-oxa-tricyclo[5.2.1.0.^{2,6}]dec-8-en-3-one **41a** (150 mg, 0.36 mmol) was dissolved in EtOAc (3.6 mL) and Pd/C (7.5 mg, 10% w/w Pd) was added. The solution was stirred under H_2 (1 atm). The solution was stirred overnight at RT before being filtered through silica, washing with EtOAc. The solvent was removed *in vacuo* to yield **41b** a white crystalline solid (149 mg, 100%). mp (78-81 °C) $[\alpha]^{22}_D$ 36.9 (c=0.89 CH_2Cl_2); IR (film) 3076, 3026, 2901, 2841, 1740, 1641 cm^{-1}; ^{1}H NMR (500 MHz, $CDCl_3$) δ 7.73-7.60 (4H, m, Ph), 7.52-7.33 (6H, m, Ph), 4.39 (1H, dd, *J*=2.5, 5.0, H-5), 3.87 (1H, dd, *J*=3.0, 11.0, Ha-1’), 3.62 (1H, dd, *J*=3.0, 11.0, Hb-1’), 3.05 (1H, dd, *J*=5.5, 11.0, H-2), 2.76-2.74 (1H, m, H-6), 2.70-2.65 (1H, m, H-1), 2.42-2.30 (1H, m, H-7), 1.66-1.44 (6H, m, H-8, H-10, H-9), 1.06 (9H, s, ^{t}Bu); ^{13}C NMR (125 Hz, $CDCl_3$) δ 178.4 (C3), 135.7 (2 x Ph), 135.6 (2 x Ph), 133.1 (^{i}Ph), 132.5 (^{i}Ph), 129.9 (2 x Ph), 127.8 (4 x Ph), 79.8 (C5), 66.2 (C1’), 48.2 (C2), 45.1 (C6), 41.6 (C10), 40.2 (C1), 39.7 (C7), 26.7 (^{t}Bu), 25.4 (C8 or C9), 19.1 (C8 or C9), 14.2 (Si-C); LC-MS (ESI$^+$) *m/z* 443 [M+Na$^+$], R_t 5.65 min [method B], HRMS [M+Na$^+$] calcd for $C_{26}H_{32}O_3SiNa$ 443.2018; found 443.2037.

(1*S*,2*R*,5*S*,6*S*,7*R*)-5-(*tert*-Butyldiphenylsilanoxymethyl)-4-oxa-tricyclo[5.2.1.0.^{2,6}]decan-3-one **41b** (140 mg, 0.332 mmol) was dissolved in anhydrous CH_2Cl_2 (1.7 mL) and cooled to -78 °C under N_2. DIBAL-H (1.0 M in toluene, 0.36 mL, 0.36 mmol) was added dropwise. The reaction was stirred at -78 °C for 2 h then quenched with EtOAc (2 mL). The solution was warmed to RT and a sat. aq. solution of Rochelles salt (6mL) was added and stirred for 3 h. The aqueous layer was separated and extracted into CH_2Cl_2 (3 x 10 mL). The organic layers were combined, dried over $MgSO_4$ and the solvent was removed *in vacuo* to yield **41c** as a clear oil (127 mg, 100%). $[\alpha]^{21}_D$ -52.6 (c=0.15, CH_2Cl_2) IR (film) 3424, 3070, 2956, 2857, 1427 cm^{-1}; ^{1}H NMR (500 MHz, $CDCl_3$) δ 7.77-7.63 (4H, m, Ph), 7.54-7.36 (6H, m, Ph), 5.25 (1H, d, *J*=9.0, H-3), 4.42 (1H, d, *J*=10.0, OH), 4.15 (1H, dd, *J*=3.0, 3.0, H-5), 3.87 (1H, dd, *J*=3.0, 11.0, H-1’), 3.56 (1H, dd, *J*=3.0, 11.0, H-1’), 2.61-2.51 (2H, m, H-2, H-6), 2.42 (1H, m, H-7 or H-1), 2.16 (1H, m, H-1 or H-7), 1.55-1.41 (4H, m, CH_2), 1.38-1.30 (2H, m, CH_2), 1.10 (9H, s, ^{t}Bu); ^{13}C NMR (125 MHz, $CDCl_3$) δ 135.8 (2 x Ph), 135.6 (2 x Ph), 132.3 (^{i}Ph), 132.3 (^{i}Ph), 129.9 (Ph), 128.2 (Ph), 127.8 (4 x Ph), 100.2 (C3), 81.3 (C5), 68.0 (C1’), 56.0 (C2 or C6), 46.5 (C2 or C6), 42.0 (CH_2), 40.5 (C7), 39.4 (C1), 26.9 (tBu), 23.2 (CH_2), 23.0 (CH_2), 19.2 (Si-C); LC-MS (ESI$^+$) m/z 441 [M+Na$^+$], R_t 4.59 min [method B]; HRMS [M+K$^+$] calcd for $C_{26}H_{34}O_3SiK$ 461.1909; found 461.1912.

(1*S*,2*R*,5*S*,6*S*,7*R*)-5-(*tert*-Butyldiphenylsilanoxymethyl)-4-oxatricyclo[5.2.1.0.^{2,6}]decan-3-ol **41c** (101 mg, 0.239 mmol) was dissolved in CH_2Cl_2 (1 mL) and cooled to -78 °C. $BF_3.OEt_2$ (90 µL, 0.718 mmol) was added and the solution stirred for 5 min. Allyltrimethylsilane (114 µL, 0.719 mmol) was added and the reaction then warmed to RT. The reaction was stirred for

18 h before quenching with H_2O (7 mL), and extracted with CH_2Cl_2 (3 x 7 mL). The combined organic layers were washed with brine (10 mL) and dried with $MgSO_4$. The solvent was removed *in vacuo* to yield **41d** a clear oil (97 mg, 92%). $[\alpha]^{24.9}_D$ 4.1 (c=0.75, CH_2Cl_2); IR (film) 3071, 3048, 2956, 1427 cm^{-1}; ^{1}H NMR (500 MHz, $CDCl_3$) δ 7.70-7.75 (4H, m, Ph), 7.41-7.48 (6H, m, Ph), 5.77-5.82 (1H, m, H-2''), 5.03-5.06 (2H, m, H-3''), 3.93 (1H, ddd, *J*=5.0, 6.0, 6.0, H-3), 3.85 (1H, ddd, *J*=5.0, 5.0, 7.0, H-5) 3.69 (1H, dd, *J*=5.5, 10.0, Ha-1'), 3.53 (1H, dd, *J*=6.5, 10.0, Hb-1'), 2.55-2.52 (1H, m, H-2), 2.35-2.29 (2H, m, H-6, Ha-1'') 2.20 (1H, br s, H-7), 2.15 (1H, br s, H-1), 2.17-2.11 (1H, m, Hb-1''), 1.68-1.60 (4H, m, H-8, H-9), 1.41 (2H, d, *J*=8.0, H-10), 1.05 (9H, s, tBu); ^{13}C NMR (125 MHz, $CDCl_3$) δ 135.7 (2 x Ph), 135.7 (2 x Ph), 135.4 (C2''), 133.8 (^{i}Ph), 133.7 (^{i}Ph), 129.6 (Ph), 129.5 (Ph), 127.6 (4 x Ph), 116.5 (C3''), 79.7 (C3), 78.9 (C5), 67.4 (C1'), 52.6 (C6), 50.4 (C2), 44.0 (C8), 42.0 (C2''), 39.9 (C7), 39.9 (C1), 26.9 (tBu), 22.9 (C9), 19.2 (Si-C).

((1*R*,2*S*,3*S*,5*S*,6*R*,7*S*)-5-Allyl-4-oxatricyclo[5.2.1.0^{2,6}]decane-3-ylmethoxy-*tert*-butyldiphenylsilane **41d** (90 mg, 0.202 mmol) was dissolved in THF (1.1 mL). The solution was cooled to 0 °C under N_2 and TBAF (0.60 mL, 0.60 mmol, 1.0 M in THF) was added by syringe. The reaction was stirred for 2 h at 0 °C before diluting with Et_2O (10 mL). The reaction mixture was washed with H_2O (3 x 10 mL) and extracted into Et_2O (3 x 10 mL). The combined organic layers were dried over $MgSO_4$ and the solvent was removed *in vacuo* to give the crude product. This was purified by column chromatography on silica gel (15% EtOAc:hexane) to yield **41e** as a clear oil (39 mg, 92%). $[\alpha]^{22}_D$ 15.3 (c=0.53 CH_2Cl_2); IR (film) 3416, 2950, 2879, 1641, 1455, 1048 cm^{-1}; ^{1}H NMR (500 MHz, $CDCl_3$) δ 5.76 (1H, tdd, *J*=7.0, 10.5, 17.0, Ha-3'), 5.11-5.06 (2H, m, H-3'), 4.04-3.99 (2H, m, H-3, H-5), 3.52 (1H, dd, *J*=4.0, 11.5, Ha-1''), 3.42 (1H, dd, *J*=8.0, 11.5, Hb-1''), 2.41-2.34 (3H, m, Ha-1', H-7 or H-1, H-2 or H-6), 2.27-2.23 (3H, m, Hb-1', H-1 or H-7, H-2 or H-6), 2.07 (1H, m, OH), 1.61-1.69 (4H, m, H-8, H-9), 1.46-1.49 (2H, m, H-10); ^{13}C NMR (125 MHz, $CDCl_3$) δ 135.2 (C2'), 116.9 (C3'), 79.8 (C3), 78.8 (C5), 66.3 (C1''), 52.7 (C6 or C2), 49.0 (C6 or C2), 43.8 (C10), 42.4 (C1'), 39.9 (C1), 39.5 (C7), 22.9 (C8 or C9), 22.9 (C8 or C9); LC-MS (ESI$^+$) *m/z* 231 [M+Na$^+$], R_t 2.20 min [method D]; HRMS [M+H$^+$] calcd for $C_{13}H_{21}O_2$ 209.1536; found 209.1539.

((1R,2S,3S,5S,6R,7S)-5-Allyl-4-oxatricyclo[5.2.1.0^{2,6}]dec-3-yl)-methanol **41e** (50 mg, 0.243 mmol) was dissolved in pyridine (1.2 mL). Pent-4-enoyl chloride (83 µL, 0.762 mmol) was added and the reaction mixture was stirred at RT for 18 h. The solvents were removed *in vacuo* and the remaining residue was dissolved in CH_2Cl_2 (10 mL) and washed with H_2O (10 mL) and brine (10 mL). The aqueous layers were combined and extracted with CH_2Cl_2 (10 mL x 2). The combined organic layers were dried with $MgSO_4$ and the solvents removed *in vacuo*. The crude reaction mixture was purified by column chromatography on silica gel (25% EtOAc:hexane) to yield **41f** as a clear oil (38 mg, 55%). $[\alpha]^{25}_D$ +15.8 (c=1.0 $CHCl_3$); IR (film) 2952, 1737 cm^{-1}; ^{1}H NMR (500 MHz, $CDCl_3$) δ 5.89-5.73 (2H, m, H-2', H-6''), 5.11-4.94 (4H, m, H-3', H-7''), 4.11-4.02 (3H, m, H-1'', H-3), 3.95 (1H, ddd, *J*=4.0, 7.0, 7.0, H-5), 2.49-2.32 (7H, m, H-5'', H-4'', H-6, H-2, HA-1'), 2.22-2.16 (3H, m, H-1, H-7, HB-1'), 1.65-1.56 (4H,

m, H-10, H-8 or H-9), 1.45-1.39 (2H, m, H-9 or H-8); ^{13}C NMR (125 MHz, $CDCl_3$) *δ* 173.0 (C3''), 136.7 (C2' or C6''), 134.9 (C2' or C6''), 116.8 (C3' or C7''), 115.5 (C3' or C7''), 79.2 (C5), 76.8 (C3), 67.6 (C1''), 52.5 (C6 or C2), 50.0 (C6 or C2), 43.9 (C10), 41.9 (C1'), 39.8 (C1 or C7), 39.5 (C1 or C7), 33.5 (C5'' or C4''), 28.8 (C5'' or C4''), 22.8 (C9 or C8), 22.8 (C9 or C8); LC-MS (ESI^+) *m/z* 313 [M+Na^+], R_t 2.50 min [method D]; HRMS [M+H^+] calcd for $C_{18}H_{27}O_3$ 291.1955 found 291.1955.

(1S,2S,3S,5S,6R,7R)-5-allyl-4-oxatricyclo[5.2.1.$0^{2,6}$]dec-3-ylmethyl pent-5-enoate **41f** (20 mg, 0.069 mmol) was dissolved in CH_2Cl_2 (137 mL, 0.0005 M). Argon was bubbled though the solution for 5 min. Grubbs II (6 mg, 0.008 mmol, 10 mol%) was added under argon and the reaction mixture was stirred for 16 h. DMSO (10 µL) was added and the solution was stirred overnight. The solvents were removed *in vacuo* and the resultant mixture purified by column chromatography on silica gel (15% EtOAc:hexane) to yield **41** as a mixture of E and Z (clear oil) (4 mg, 22%). $[\alpha]^{24}_D$ -6.8 (c=0.5 $CHCl_3$); IR (film) 2951, 1737 (C=O), 1171, 1106 cm^{-1}; ^1H NMR (500 MHz, $CDCl_3$) *δ* 5.59-5.42 (2H, m, H-7', H-6'), 4.18-3.90 (4H, m, H-1', H-3, H-5), 2.40-2.28 (6H, m, CH_2, CH), 2.26-2.14 (4H, m, CH, CH_2), 1.70-1.52 (4H, m, CH_2), 1.47-1.37 (2H, m, CH_2); ^{13}C NMR (125 MHz, $CDCl_3$) *δ* 173.2, 173.0 (C3'), 130.2, 129.5, 129.0, 128.0 (2C, C7', C6'), 79.8, 79.6, 77.6, 77.5 (2C, C3, C5), 67.4, 67.5 (C1'), 52.4, 52.4 (CH), 49.0, 49.0 (CH), 43.6, 43.5 (CH_2), 41.3 (CH_2), 40.6 (CH_2), 40.0, 39.8 (CH), 39.6 (CH), 34.4, 34.3 (CH_2), 28.0, 27.9 (CH_2), 22.9 (CH_2), 22.8 (CH_2); LC-MS (ESI^+) *m/z* 285 [M+Na^+], R_t 2.32 min [method D]; HRMS [M+H^+] calcd for $C_{16}H_{23}O_3$ 263.1642 found 263.1643.

(E)-(1S,11S,12R,17S)-3,18-Dioxatetracyclo[9.6.1.$1^{13,16}$.0 ^{12,17}]nonadec-8-en-4-one 42

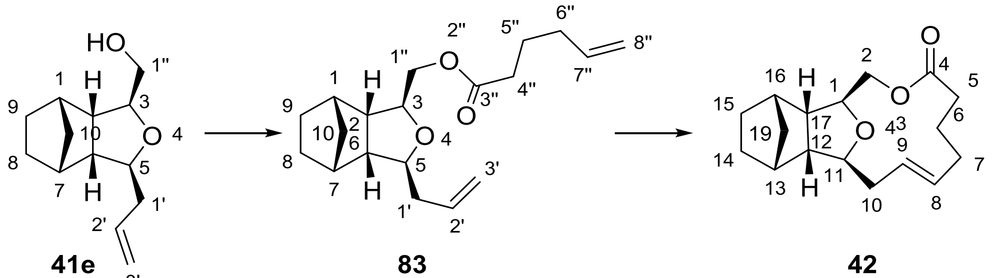

41e **83** **42**

((1R,2S,3S,5S,6R,7S)-5-Allyl-4-oxatricyclo[5.2.1.$0^{2,6}$]dec-3-yl)methanol **41e** (50 mg, 0.243 mmol) was dissolved in pyridine 1.2 mL. Hex-5-enoyl chloride (100 mg, 0.762 mmol) was added and the reaction mixture was stirred at RT for 18 h. The solvents were removed *in vacuo* and the remaining residue was dissolved in CH_2Cl_2 (10 mL) and washed with H_2O (10 mL) and brine (10 mL). The aqueous layers were combined and extracted with CH_2Cl_2 (10 mL x 2). The combined organic layers were dried with $MgSO_4$ and the solvents removed *in vacuo*. The crude reaction mixture was purified by column chromatography on silica gel (15% EtOAc:hexane) to yield **83** as a clear oil (73 mg, 100%). $[\alpha]^{25}_D$ -5.5 (c=1.0 $CHCl_3$); IR (film) 2952, 1737 cm^{-1}; ^1H NMR (500 MHz, $CDCl_3$) *δ* 5.79 (1H, dddd, *J*=7.0, 7.0 10.5, 17.0, H-2'

or H-7''), 5.76 (1H, dddd, *J*=7.0, 7.0 10.0, 17.0, H-2' or H-7''), 5.09-4.97 (4H, m, H-3', H-8''), 4.09-4.00 (3H, m, H-1'', H-3), 3.90 (1H, ddd, *J*=4.0, 7.0, 7.0, H-5), 2.45-2.32 (5H, m, HA-1', H-4'', H-2, H-6), 2.21-2.16 (3H, m, H-1, H-7, HB-1'), 2.16-2.08 (2H, m, H-6''), 1.75 (2H, tt, *J*=7.5, 7.5, H-5''), 1.63-1.56 (4H, m, HA-8, HA-9, H-10), 1.13-1.43 (2H, m, HB-9, HB-8); ^{13}C NMR (125 MHz, $CDCl_3$) δ 173.5 (C3''), 137.7 (C7''), 134.9 (C2'), 116.8 (C8'' or C3'), 115.3 (C8'' or C3'), 79.2 (C5), 76.7 (C3), 67.5 (C1''), 52.5 (C6 or C2), 50.1 (C6 or C2), 43.9 (C10), 41.9 (C1'), 39.8 (C1 or C7), 39.5 (C1 or C7), 33.4 (C4''), 33.0 (C6''), 24.0 (C5''), 22.8 (C9 or C8), 22.8 (C9 or C9); LC-MS (ESI$^+$) *m/z* 327 [M+Na$^+$], R_t 2.55 min [method D]; HRMS [M+H$^+$] calcd for $C_{19}H_{30}O_3$ 305.2111 found 305.2115; Found: C,74.91; H, 9.26%; $C_{17}H_{28}O_3$ requires: C, 74.96; H, 9.27%.

(1S,2S,3S,5S,6R,7R)-5-Allyl-4-oxatricyclo[5.2.1.0^{2,6}]dec-3-ylmethyl hex-5-enoate **83** (24 mg, 0.078 mmol) was dissolved in CH_2Cl_2 (156 mL, 0.0005 M). Argon was bubbled though the solution for 5 min. Grubbs II (7 mg, 0.008 mmol, 10 mol%) was added under argon and the reaction mixture was stirred for 5 d. DMSO (10 µL) was added and the solution was stirred overnight. The solvents were removed *in vacuo* and the resultant mixture purified by column chromatography on silica gel (15% EtOAc:hexane) to yield **42** as a white crystalline solid (11 mg, 51%). mp (94-96 ˚C); $[\alpha]^{24}_D$ -10.4 (c=1.0 $CHCl_3$); IR (film) 2948, 1729, 1440 cm^{-1}; ^{1}H NMR (500 MHz, $CDCl_3$) δ 5.54 (1H, ddd, *J*=7.0, 7.0, 14.0, H-9 or H-8), 5.28 (1H, ddd, *J*=7.0, 7.0, 14.0, H-9 or H-8), 4.33 (1H, dd, *J*=3.0, 12.0, Ha-2), 4.14-4.09 (2H, m, H-1, H-11), 3.69 (1H, dd, *J*=7.5, 12.0, Hb-2), 2.56-2.49 (1H, m, H-12 or H-17), 2.77-2.32 (3H, m, H-16 or H-17, CH_2, CH_2), 2.29-1.98 (7H, m, H-12 or H-17, H-16 or H-13, CH_2, CH_2, CH_2), 1.96-1.81 (2H, m, CH_2), 1.68-1.53 (4H, m, Ha-14, Ha-15, CH_2), 1.46-1.38 (2H, m, Hb-14, Hb-15); ^{13}C NMR (125 MHz, $CDCl_3$) δ 175.3 (C4), 132.0 (C8 or C9), 127.4 (C8 or C9), 77.6 (C1 or C11), 76.1 (C1 or C11), 65.8 (C2), 51.5 (C12 or C17), 48.8 (C16 or C13), 43.7 (CH_2), 39.8 (CH), 39.5 (CH), 39.5 (CH), 33.9 (CH_2), 33.8 (CH_2), 24.0 (CH_2), 22.9 (C14 or C15), 22.7 (C14 or C15); LC-MS (ESI$^+$) *m/z* 299 [M+Na$^+$], R_t 2.43 min [method D]; HRMS [M+H$^+$] calcd for $C_{17}H_{26}O_3$ 277.1798 found 277.1799.

2-[(1R,3S,3aS,7aR)-3-{[(tert-butyldiphenylsilyl)oxy]methyl}-1,3,3a,4,7,7a-hexahydro-2-benzofuran-1-yl]-2-methylpropanal 44

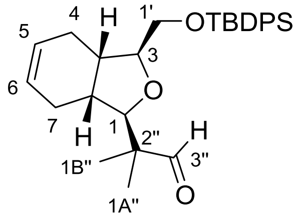

44

(3*S*,3a*S*,7a*R*)-3-((*tert*-Butyldiphenylsilyloxy)methyl)-1,3,3a,4,7,7a-hexahydroisobenzofuran-1-ol **11** (100 mg, 0.245 mmol) was dissolved in CH_2Cl_2 (2.8 mL) and cooled to -78 °C. $BF_3.OEt_2$ (94 µL, 0.735 mmol) was added and the solution was stirred for 5 min. Trimethyl(2-methylprop-1-enyloxy)silane (49 µL, 0.269 mmol) was then added and the reaction was warmed to RT and stirred for 3 h. The reaction was diluted with CH_2Cl_2 (20 mL), and washed with H_2O (2 x 20 mL), followed by brine (20 mL). The organic layer was dried with $MgSO_4$. The solvents were removed *in vacuo* and the resultant crude residue was purified by column

chromatography on silica gel (15% EtOAc:hexane) to yield the product as a clear oil (110 mg, 97%). $[\alpha]^{21}_{D}$ +1.1 (c=5.0 $CHCl_3$); IR (film) 3018, 1708, 1218 cm^{-1}; ^1H NMR (500 MHz, $CDCl_3$) δ 9.66 (1H, s, H-3''), 7.73-7.68 (4H, m, Ph), 7.46-7.37 (6H, m, Ph), 5.81-5.78 (2H, m, H-5, H-6), 3.81-3.75 (2H, m, Ha-1', H-1 or H-3), 3.73-3.68 (2H, m, Hb-1', H-1 or H-3), 2.29-2.17 (4H, m, H-3a, H-7a, Ha-4, Ha-7), 1.97-1.86 (2H, Hb-4, Hb-7), 1.11 (3H, s, Me), 1.08 (3H, s, Me), 1.07 (9H, s, tBu); ^{13}C NMR (125 MHz, $CDCl_3$) δ 206.1 (C3''), 135.7 (2 x Ph), 135.6 (2 x Ph), 133.6 (iPh), 133.4 (iPh), 129.6 (Ph), 129.6 (Ph), 127.7 (4 x Ph), 127.0 (C5 or C6), 126.3 (C5 or C6), 89.1 (C1), 83.8 (C3), 64.7 (C1'), 49.6 (C2''), 37.8 (C3a or C7a), 37.2 (C3a or C7a), 27.4 (C7), 26.8 (tBu), 24.0 (C4), 19.2 (C-Si), 19.2 (Me), 17.6 (Me); LC-MS (ESI^+) *m/z* 485 [$M+Na^+$], R_t 2.60 min [method E]; Found: C, 75.35; H, 8.15%; $C_{29}H_{38}O_3Si$ requires: C, 75.28; H, 8.28%.

2-((1S,3S,3aS,7aR)-3-((tert-butyldiphenylsilyloxy)methyl)-1,3,3a,4,7,7a-hexahydroisobenzofuran-1-yl)-1-phenylethanone 45

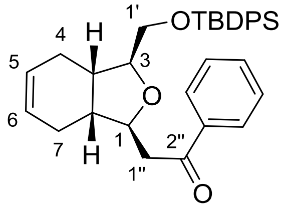

45

(3*S*,3a*S*,7a*R*)-3-((*tert*-Butyldiphenylsilyloxy)methyl)-1,3,3a,4,7,7a-hexahydroisobenzofuran-1-ol **11** (100 mg, 0.245 mmol) was dissolved in CH_2Cl_2 (2.8 mL) and cooled to -20 °C. $BF_3.OEt_2$ (63 µL, 0.490 mmol) was added and the solution was stirred for 5 min. Trimethyl(1-phenylvinyloxy)silane (100 µL, 0.490 mmol) was then added and the reaction was warmed to RT and stirred for a further 16 h. The reaction was diluted with CH_2Cl_2 (20 mL), and washed with H_2O (2 x 20 mL), followed by brine (20 mL). The organic layer was dried with $MgSO_4$. The solvents were removed *in vacuo* and the resultant crude residue was a green oil. The product was isolated by column chromatography on silica gel (10% EtOAc:hexane) to yield **45** as a clear oil (95 mg, 78%). $[\alpha]^{21}_{D}$ -1.66 (c=1.0 $CHCl_3$); IR (film) 3070, 2930, 2857, 1684 (CO) cm^{-1}; ^1H NMR (500 MHz, $CDCl_3$) δ 7.94-7.90 (2H, m, Ph), 7.75-7.67 (5H, m, Ph), 7.44-7.35 (8H, m, Ph), 5.77-5.68 (2H, m, H-5, H-6), 4.35 (1H, dd, J=6.0, 12.0, H-1), 3.82-3.77 (1H, m, H-3), 3.76 (1H, dd, J=4.5, 11.0, Ha-1''), 3.68 (1H, dd, J=4.0, 11.0, Hb-1''), 3.30 (1H, dd, J=6.5, 16.5, Ha-1'), 3.07 (1H, dd, J=6.0, 16.5, Hb-1'), 2.54-2.48 (1H, m, H-3a), 2.47-2.36 (2H, m, Ha-4, Ha-7), 2.26-2.18 (1H, m, H-7a), 2.03-1.93 (2H, m, Hb-4, Hb-7), 1.09 (9H, s, tBu); ^{13}C NMR (125 MHz, $CDCl_3$) δ 198.7 (C2''), 137.2 (iPh), 135.6 (2 x Ph), 135.6 (2 x Ph), 134.8 (Ph), 133.6 (Ph), 133.4 (iPh), 133.0 (iPh), 129.7 (Ph), 129.6 (Ph), 129.6 (Ph), 128.5 (Ph), 128.2 (Ph), 127.7 (Ph), 127.7 (2 x Ph), 127.7 (Ph), 125.4 (C5 or C6), 125.2 (C5 or C6), 84.1 (C3), 80.2 (C1), 64.9 (C1'), 44.4 (C1''), 40.1 (C7a), 35.5 (C3a), 26.9 (tBu), 24.8 (C4 or C7), 24.5 (C4 or C7), 19.3 (Si-C); LC-MS (ESI^+) *m/z* 533 [$M+Na^+$], R_t 2.52 min [method D]; HRMS [$M+Na^+$] calcd for $C_{33}H_{37}O_3SiNa$ 533.2482 found 533.2492; Found: C, 77.51; H, 7.40%; $C_{33}H_{38}O_3Si$ requires: C, 77.60; H, 7.50%.

3-[(1R,3S,3aS,7aR)-3-(Hydroxymethyl)-octahydro-2-benzofuran-1-yl]-3-methylbutan-2-one 46

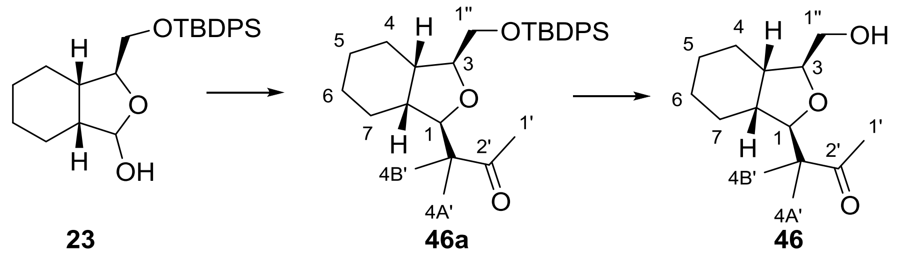

(3S,3aS,7aR)-3-{[(*tert*-Butyldiphenylsilyl)oxy]methyl}-octahydro-2-benzofuran-1-ol **23** (235 mg, 0.573 mmol) was dissolved in CH_2Cl_2 (5 mL) and trimethyl(3-methylbut-2-en-2-yloxy)silane (100 mg, 0.630 mmol) was added. The solution was cooled to -78 °C and TMSOTf (286 µL, 0.1 M in CH_2Cl_2, 0.029 mmol) was added and the reaction was warmed to -18 °C for 18 h. The reaction was quenched with sat. aqueous $NaHCO_3$ (20 mL) and extracted in CH_2Cl_2 (3 × 30 mL). The combined organic layers were dried over $MgSO_4$ and the solvent removed *in vacuo*. The crude residue was purified by column chromatography on silica gel (15% EtOAc:hexane) to yield **46a** as a clear oil (101 mg, 36%). $[\alpha]^{24.3}_D$ -22.7 (c=0.75 $CHCl_3$); IR (film) 2929, 2856, 1774 cm^{-1}; ^1H NMR (500 MHz, $CDCl_3$) δ 7.74-7.65 (4H, m, Ph), 7.46-7.35 (6H, m, Ph), 3.91 (1H, ddd, J=4.0, 4.0, 9.0, H-3), 3.87 (1H, d, J=3.5, H-1), 3.76 (1H, dd, J=4.0, 11.0, Ha-1''), 3.67 (1H, dd, J=5.0, 11.0, Hb-1'') 2.17 (3H, s, H-1'), 2.05-1.94 (2H, m, H-3a, H-7a), 1.73-1.54 (4H, m, CH_2), 1.49-1.23 (4H, m, CH_2), 1.14 (3H, s, Me), 1.09 (3H, s, Me), 1.08 (9H, s, tBu); ^{13}C NMR (125 MHz, $CDCl_3$) δ 213.2 (C2'), 135.7 (2 x Ph), 135.6 (2 x Ph), 133.7 (iPh), 133.5 (iPh), 129.6 (Ph), 129.5 (Ph), 127.8 (2 x Ph), 127.6 (2 x Ph), 88.8 (C1), 80.7 (C3), 65.2 (C1''), 51.5 (C3'), 39.3 (C7a or C3a), 38.8 (C7a or C3a), 29.6 (CH_2), 26.7 (tBu), 26.7 (C1'), 24.3 (CH_2), 24.2 (CH_2), 22.2 (CH_2), 20.8 (C4A' or C4B'), 20.3 (C4A' or C4B'), 19.2 (C-Si); HRMS [M+Na$^+$] calcd for $C_{30}H_{42}O_3SiNa$ 501.2795 found 501.2799.

3-[(1R,3S,3aS,7aR)-3-{[(*tert*-Butyldiphenylsilyl)oxy]methyl}-octahydro-2-benzofuran-1-yl]-3-methylbutan-2-one **46a** (81 mg, 0.169 mmol), was dissolved in THF (2 mL). TBAF (1.0 M in THF, 0.51 mL, 0.51 mmol) and AcOH (0.31 mL, 0.508 mmol) were added and the reaction stirred at RT for 16 h. The solution was diluted with Et_2O (20 mL) and washed with H_2O (2 x 20 mL), brine (20 mL) and the organic layer was dried with $MgSO_4$. The crude residue was purified by column chromatography on silica gel to yield **46** as a clear oil (32 mg, 78%). $[\alpha]^{24.6}_D$ +12.4 (c=1.0 $CHCl_3$); IR (film) 3428, 2926, 2857, 1698, 1450 cm^{-1}; ^1H NMR (500 MHz, $CDCl_3$) δ 3.89 (1H, ddd, J=3.0, 5.5, 13.0, H-3), 3.87 (1H, d, J=3.5, H-1), 3.74 (1H, ddd, J=3.0, 6.5, 12.0, Ha-1'), 3.51 (1H, ddd, J=3.5, 3.5, 12.0, Hb-1'), 2.18 (3H, s, H-1''), 2.06-1.99 (2H, m, OH, H-7a), 1.96-1.90 (1H, m, H-3a), 1.74-1.52 (4H, m, CH_2), 1.43-1.21 (4H, m, CH_2), 1.11 (3H, s, Me), 1.09 (3H, s, Me); ^{13}C NMR (125 MHz, $CDCl_3$) δ 212.8 (C2''), 89.3 (C1), 80.6 (C3), 63.8 (C1'), 51.5 (C3''), 39.6 (C7a), 38.0 (C3a), 29.8 (CH_2), 26.4 (C1''), 24.2 (CH_2), 23.9 (CH_2), 21.9 (CH_2), 21.3 (Me), 19.8 (Me); LC-MS (ESI$^+$) *m/z* 263 [M+Na$^+$], R_t 1.42 min [method D]; HRMS [M+Na$^+$] calcd for $C_{14}H_{24}O_3Na$ 263.1617 found 263.1617.

1-[(1S,3S,3aS,7aR)-3-(hydroxymethyl)-octahydro-2-benzofuran-1-yl]-3,3-dimethylbutan-2-one 47

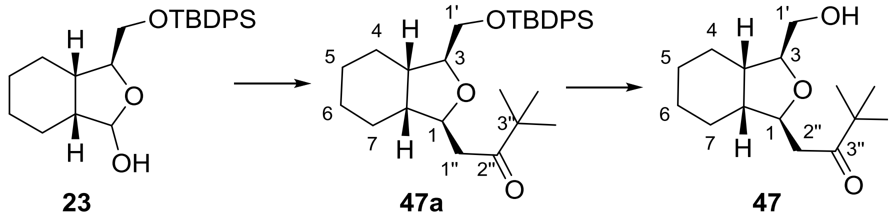

(3*S*,3a*S*,7a*R*)-3-((*tert*-Butyldiphenylsilyloxy)methyl)octahydroisobenzofuran-1-ol **23** (615 mg, 1.50 mmol) was dissolved in CH_2Cl_2 (15 mL) and cooled to -20 °C. $BF_3.OEt_2$ (578 µL, 4.50 mmol) was added and the solution was stirred for 5 min. (3,3-Dimethylbut-1-en-2-loxy)trimethylsilane (797 µL, 4.50 mmol) was then added and the reaction was warmed to RT and stirred for 16 h. The reaction was diluted with CH_2Cl_2 (20 mL), and washed with H_2O (2 x 20 mL), followed by brine (20 mL). The organic layer was dried with $MgSO_4$. The product was isolated by column chromatography on silica gel (15% EtOAc:hexane) to yield **47a** as a clear oil (483 mg, 65%). $[\alpha]^{21}_D$ +9.0 (c=1.0 $CHCl_3$); IR (film) 2931, 2858, 1702, 1473 cm^{-1}; ^1H NMR (500 MHz, $CDCl_3$) δ 7.71-7.66 (4H, m, Ph), 7.43-4.39 (6H, m, Ph), 4.30-4.25 (1H, m, H-1), 3.89-3.85 (1H, m, H-3), 3.68 (1H, dd, J=5.0, 11.0, Ha-1'), 3.61 (1H, dd, J=4.0, 11.0, Hb-1'), 2.78 (1H, dd, J=6.0, 17.0, Ha-1''), 2.53 (1H, dd, J=6.0, 17.0, Hb-1''), 2.27-2.22 (1H, m, H-7a), 1.93-1.87 (1H, m, 3a), 1.65-1.26 (8H, m, 4 x CH_2), 1.10 (9H, s, tBu), 1.08 (9H, s, tBu); ^{13}C NMR (125 MHz, $CDCl_3$) δ 214.6 (C-3''), 135.6 (2 x Ph), 134.8 (2 x Ph), 133.7 (iPh), 133.5 (iPh), 129.6 (Ph), 129.6 (Ph), 127.7 (2 x Ph), 127.6 (2 x Ph), 82.3 (C3), 78.4 (C1), 65.0 (C1'), 44.1 (C3''), 43.1 (C3a), 42.7 (C1''), 38.3 (C7a), 26.1 (tBu), 25.9 (tBu), 25.9 (CH_2), 23.3 (CH_2), 23.1 (CH_2), 22.6 (CH_2), 19.3 (Si-C), HRMS [M+Na$^+$] calcd for $C_{31}H_{44}O_3SiNa$ 515.2957 found 515.2938.

1-[(1S,3S)-3-{[(*tert*butyldiphenylsilyl)oxy]methyl}-octahydro-2-benzofuran-1-yl]-3,3-dimethylbutan-2-one **47a** (467mg, 0.949 mmol) was dissolved in THF (10 mL). The solution was cooled to 0 °C under N_2, then TBAF (2.8 mL, 1.0 M in THF, 2.8 mmol) and AcOH (1.7 mL, 2.8 mmol) was added by syringe. The reaction was stirred for 16 h at RT before diluting with Et_2O (30 mL). The reaction mixture was washed with H_2O (30 mL) and extracted into Et_2O (2 x 30 mL). The combined organic layers were dried with $MgSO_4$ and solvents were removed *in vacuo* to yield the crude product as a clear oil. This was purified by column chromatography on silica gel (10% EtOAc:hexane) to yield **47** as a clear oil (161 mg, 67%). $[\alpha]^{22}_D$ + 17.6 (c=2.60 CH_2Cl_2); IR (film) 3414, 3075, 3026, 2975, 2841, 1700, 1478 cm^{-1}; ^1H NMR (500 MHz, $CDCl_3$) δ 4.26-4.21 (1H, m, H-1), 3.93-3.89 (1H, m, H-3), 3.76-3.71 (1H, m, Ha-1'), 3.49-3.44 (1H, m, Hb-1'), 2.71 (1H, dd, J=7.5, 15.5, Ha-1''), 2.63 (1H, dd, J=5.5, 15.5, Hb-1''), 2.24-2.18 (1H, m, H-3a), 1.95-1.89 (1H, m, H-7a), 1.79-1.71 (1H, m, CH_2), 1.68-1.51 (3H, m, 3 x CH_2), 1.38-1.35 (3H, m, 3 x CH_2) 1.35-1.26 (1H, m, CH_2), 1.16 (9H, s, tBu); ^{13}C NMR

(125 MHz, $CDCl_3$) δ 82.0 (C3), 79.3 (C1), 63.8 (C1'), 44.3 (C3''), 43.7 (C1''), 42.0 (C7a), 37.0 (C3a), 26.5, (CH_2), 26.4 (^tBu), 25.1 (CH_2), 23.6 (CH_2), 22.5 (CH_2), missing quaternary C=O; LC-MS (ESI^+) *m/z* 255 [M+Na$^+$], R_t 1.52 min [method E]; HRMS [M+Na$^+$] calcd for $C_{15}H_{26}O_3Na$ 277.1779; found 217.1774.

1-[(1S,3S,3aS,7aR)-3-(hydroxymethyl)-1,3,3a,4,7,7a-hexahydro-2-benzofuran-1-yl]-3,3-imethylbutan-2-one 48

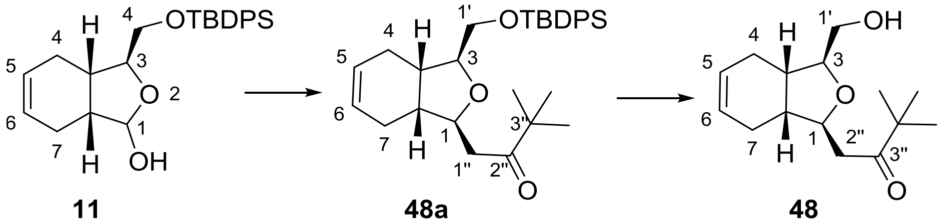

11 **48a** **48**

(3*S*,3a*S*,7a*R*)-3-((*tert*-Butyldiphenylsilyloxy)methyl)-1,3,3a,4,7,7a-hexahydroisobenzofuran-1-ol **11** (100 mg, 0.245 mmol) was dissolved in CH_2Cl_2 (2.8 mL) and cooled to -20 °C. $BF_3.OEt_2$ (94 µL, 0.735 mmol) was added and the solution was stirred for 5 min. (3,3-Dimethylbut-1-en-2-loxy)trimethylsilane (126 µL, 0.735 mmol) was then added and the reaction was warmed to RT and stirred for 16 h. The reaction was diluted with CH_2Cl_2 (20 mL), and washed with H_2O (2 x 20 mL), followed by brine (20 mL). The organic layer was dried with $MgSO_4$. The solvents were removed *in vacuo* and the resultant crude residue was analysed by ^{1}H NMR and found to be a mixture of diastereoisomers (85:15). The major isomer was isolated by column chromatography on silica gel (10% EtOAc:hexane) to yield **48a** as a clear oil (85 mg, 62%). $[\alpha]^{21}_D$ +9.0 (c=1.0 $CHCl_3$); IR (film) 2931, 2858, 1702, 1473 cm^{-1}; ^{1}H NMR (500 MHz, $CDCl_3$) δ 7.75-7.65 (4H, m, Ph), 7.47-4.36 (6H, m, Ph), 5.77-5.67 (2H, m, H-5, H-6), 4.21 (1H, dd, J=6.0, 12.0, H-1), 3.79 (2H, m, H-3, Ha-1'), 3.71-3.66 (1H, m, Hb-1'), 2.84 (1H, dd, J=6.5, 17.0, Ha-1''), 2.59 (1H, dd, J=6.0, 17.0, Hb-1''), 2.48-2.41 (1H, m, H-3a), 2.35-2.22 (2H, m, Ha-4, Ha-7), 2.18-2.15 (1H, m, H-7a), 2.12-2.06 (2H, m, Hb-4, Hb-7), 1.11 (9H, s, tBu), 1.08 (9H, s, tBu); ^{13}C NMR (125 MHz, $CDCl_3$) δ 135.6 (2 x Ph), 134.8 (2 x Ph), 133.6 (iPh), 133.5 (iPh), 129.7 (Ph), 129.6 (Ph), 127.7 (2 x Ph), 127.7 (2 x Ph), 125.5 (C5 or C6), 125.2 (C5 or C6), 83.8 (C3), 80.0 (C1), 65.0 (C1'), 44.2 (C3'), 42.4 (C1''), 40.1 (C7a), 35.7 (C3a), 26.9 (tBu), 26.1 (tBu), 24.9 (C4 or C5), 24.6 (C4 or C7), 19.3 (Si-C), missing quaternary C=O; LC-MS (ESI^+) *m/z* 513 [M+Na$^+$], R_t 2.58 min [method E]; HRMS [M+Na$^+$] calcd for $C_{31}H_{42}O_3SiNa$ 513.2795 found 513.2808; Found: C, 75.81; H, 8.73%; $C_{31}H_{42}O_3Si$ requires: C, 75.87; H, 8.73%.

1-((1S,3S,3aS,7aR)-3-((*tert*-Butyldiphenylsilyloxy)methyl)-1,3,3a,4,7,7a-hexahydroisobenzo furan-1-yl)-3,3-dimethylbutan-2-one **48a** (216 mg, 0.485 mmol) was dissolved in THF (10.0 mL). The solution was cooled to 0 °C under N_2, then TBAF (1.45 mL, 1.0 M in THF, 1.45 mmol) was added by syringe. The reaction was stirred for 16 h at RT before quenching with

Et_2O (30 mL). The reaction mixture was washed with H_2O (30 mL) and extracted into Et_2O (2 x 30 mL). The combined organic layers were dried with $MgSO_4$ and solvents were removed *in vacuo* to yield the crude product as a clear oil. This was purified by column chromatography on silica gel (10% EtOAc:hexane) to yield **48** as a clear oil (84 mg, 67%). $[\alpha]^{22}_{D}$ +17.6 (c=2.60 CH_2Cl_2); IR (film) 3418, 3026, 2967, 2907, 1700, 1478 cm^{-1}; ^1H NMR (500 MHz, $CDCl_3$) δ 5.77-5.67 (2H, m, H-5, H-6), 4.16 (1H, dd, J=6.0, 12.0, H-1), 3.80-3.75 (2H, m, H-3, Ha-1'), 3.52-3.47 (1H, m, Hb-1'), 2.73 (1H, dd, J=6.5, 17.0, Ha-1''), 2.59 (1H, dd, J=6.0, 17.0, Hb-1''), 2.42-2.34 (2H, m, H-3a, OH), 2.31-2.27 (2H, m, Ha-4, Hb-7), 2.10-2.03 (1H, m, H-7a), 1.97-1.88 (2H, m, Hb-4, Hb-7); ^{13}C NMR (125 MHz, $CDCl_3$) δ 125.5 (C5 or C6), 125.1 (C5 or C6), 83.8 (C3), 81.4 (C1), 63.2 (C1'), 44.4 (C3'), 42.2 (C1''), 40.6 (C7a), 35.8 (C3a), 24.9 (C4 or C5), 23.6 (C4 or C7), missing quaternary C=O; LC-MS (ESI$^+$) *m/z* 275 [M+Na$^+$], R_t 1.47 min [method E].

2-((1R,3S,3aS,7aR)-3-(hydroxymethyl)-1,3,3a,4,7,7a-hexahydroisobenzofuran-1-yl)-2-methylpropan-1-ol 49

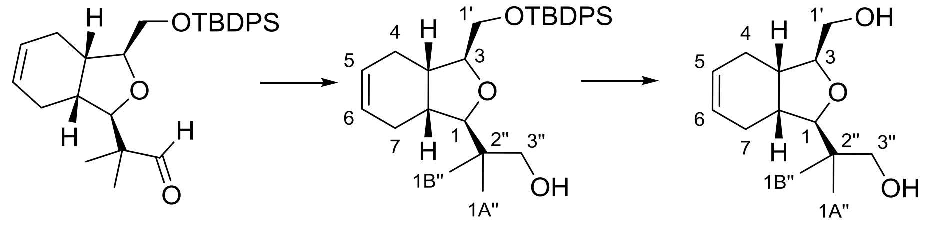

44 **49a** **49**

2-[(1R,3S,3aS,7aR)-3-{[(*tert*-Butyldiphenylsilyl)oxy]methyl}-1,3,3a,4,7,7a-hexahydro-2-benzofuran-1-yl]-2-methylpropanal **44** (110 mg, 0.239 mmol) was dissolved in THF:MeOH (4:1 ratio, 2.5 mL) and cooled to 0 °C. $NaBH_4$ (27 mg, 0.717 mmol) was added and the solution stirred for 1.5 h. The solution was diluted with Et_2O (10 mL), then washed with H_2O (10 mL) and brine (10 mL). The organic layer was then dried with $MgSO_4$. The solvents were removed *in vacuo* to yield **49a** as a clear oil (93 mg, 82%). $[\alpha]^{22}_{D}$ -8.4 (c=1.0 $CHCl_3$); IR (film) 3424, 1472 cm^{-1}; ^1H NMR (500 MHz, $CDCl_3$) δ 7.76-7.67 (4H, m, Ph), 7.50-7.37 (6H, m, Ph), 5.88-5.74 (2H, m, H-5, H-6), 3.83 (1H, dd, J=5.0, 7.0, Ha-1'), 3.70-3.66 (2H, m, Hb-1', H-3), 3.61 (1H, d, J=5.0, H-1), 3.51-3.47 (2H, m, H-3''), 3.12 (1H, t, J=7.0, OH), 2.33-2.24 (2H, m, H-3a, H-7a), 2.24-2.14 (2H, m, Ha-4, Ha-7), 1.97-1.90 (1H, Hb-4, or Hb-7), 1.86-1.79 (1H, Hb-4, or Hb-7), 1.09 (9H, s, ^{t}Bu), 1.01 (3H, s, Me), 0.90 (3H, s, Me); ^{13}C NMR (125 MHz, $CDCl_3$) δ 135.7 (2 x Ph), 135.6 (2 x Ph), 133.4 (iPh), 133.4 (iPh), 129.7 (Ph), 129.6 (Ph), 127.7 (4 x Ph), 127.1 (C5 or C6), 126.7 (C5 or C6), 93.4 (C1), 83.7 (C3), 72.4 (C1'), 64.5 (C3''), 37.9 (C2''), 37.8 (C3a or C7a), 37.4 (C3a or C7a), 27.7 (C4 or C7), 26.8 (tBu), 23.9 (C4 or C7), 22.7 (Me), 19.2 (Si-C), 19.0 (Me); LC-MS (ESI$^+$) *m/z* 487 [M+Na$^+$], R_t 3.12 min [method E]; HRMS [M+Na$^+$] calcd for $C_{29}H_{40}O_3SiNa$ 487.2639 found 487.2637; Found: C, 75.07; H, 8.64%; $C_{29}H_{40}O_3SiNa$ requires: C, 74.95; H, 8.68%.

2-[(1R,3S,3aS,7aR)-3-{[(*tert*-butyldiphenylsilyl)oxy]methyl}-1,3,3a,4,7,7a-hexahydro-2-benzofuran-1-yl]-2-methylpropan-1-ol **49a** (80 mg, 0.172 mmol) was dissolved in THF (0.5 mL) and TBAF (1.0 M in THF, 1.70 mL) was added. The reaction was stirred at RT for 16 h. The reaction mixture was then diluted with Et_2O (5 mL) and washed with H_2O (5 mL x 2). Solvents were removed *in vacuo* and the crude residue was purified by column chromatography on silica gel (15% EtOAc:hexane) to yield **49** as a clear oil (16 mg, 41%). $[\alpha]^{22}_D$ -8.4 (c=1.0 $CHCl_3$); ^1H NMR (500 MHz, $CDCl_3$) 5.85-5.78 (2H, m, H-5, H-6), 3.84 (1H, dd, J=5.0, 7.0, Ha-1'), 3.71-3.68 (1H, m, H-1), 3.64-3.60 (2H, m, Hb-1', H-3), 3.50 (1H, d, J=11.0, Ha-3''), 3.46 (1H, d, J=11.0, Hb-3''), 2.31-2.17 (4H, m, H-3a, H-7a, Ha-4, Ha-7), 1.98-1.84 (2H, Hb-4, Hb-7), 0.94 (3H, s, Me), 0.91 (3H, s, Me); ^{13}C NMR (125 MHz, $CDCl_3$) δ, 126.7 (C5 or C6), 126.6 (C5 or C6), 93.2 (C1), 83.6 (C3), 71.7 (C1'), 63.0 (C3''), 38.1 (C2''), 37.3 (C3a or C7a), 36.8 (C3a or C7a), 27.8 (C4 or C7), 23.6 (C4 or C7), 22.2 (Me), 19.3 (Me), LC-MS (ESI^+) *m/z* 227 [$M+Na^+$], R_t 1.38 min [method E]; HRMS [$M+Na^+$] calcd for $C_{13}H_{22}O_3$ Na 249.1461 found 249.1463.

{2-[(1R,3S,3aS,7aR)-3-{[(*tert*-Butyldiphenylsilyl)oxy]methyl}-1,3,3a,4,7,7a-hexahydro-2-benzofuran-1-yl]-2-methylpropyl}(benzyl)amine 50

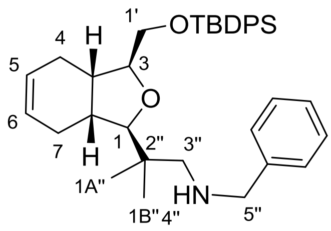

50

2-[(1R,3S,3aS,7aR)-3-{[(*tert*-Butyldiphenylsilyl)oxy]methyl}-1,3,3a,4,7,7a-hexahydro-2-benzofuran-1-yl]-2-methylpropanal **44** (107 mg, 0.232 mmol) and benzyl amine (25 µM, 0.232 mmol) was dissolved in 1,2-dichloroethane (3.0 mL). Sodium triacetoxyborohydride (68 mg, 0.324 mmol) was then added and the solution stirred at RT for 3 h**.** The reaction mixture was quenched by adding aqueous sat. $NaHCO_3$ (10 mL), and the solution was extracted with EtOAc. The organic extract was dried ($MgSO_4$), and the crude residue was purified by ionic exchange chromatography on acidic resin (5 g) to yield **50** as a clear oil (67 mg, 52%). $[\alpha]^{21.9}_D$ -7.0 (c=1.0 CH_2Cl_2); IR (film) 3028, 2930, 2856, 1471, 1428 cm^{-1}; ^1H NMR (500 MHz, $CDCl_3$) δ 7.75-7.69 (4H, m, Ph), 7.46-7.20 (11H, m, Ph), 5.83-5.74 (2H, m, H-5, H-6), 3.89-3.67 (5H, m, H-1, H-1', H-3''), 3.57-3.54 (1H, m, H-3), 2.62 (1H, d, J=12.0, Ha-5''), 2.44 (1H, d, J=12.0, Hb-5''), 2.23-2.11 (4H, m, H-3a, H-7a, Ha-4, Ha-7), 1.97-1.84 (2H, m, Hb-4, Hb-7), 1.07 (9H, s, tBu), 0.93 (3H, s, Me), 0.92 (3H, s, Me); ^{13}C NMR (125 MHz, $CDCl_3$) δ 135.7 (Ph), 135.6 (Ph), 133.8 (iPh), 133.6 (iPh), 129.6 (Ph), 129.5 (Ph), 128.2 (2 x Ph), 128.6 (2 x Ph), 127.62 (4 x Ph), 127.0 (2 x Ph), 126.8 (C5 or C6), 126.7 (C5 or C6), 91.3 (C3), 83.3 (C1), 65.3 (C1'), 58.5 (C3''), 54.7 (C5''), 38.3 (C3a or C7a), 36.9 (C3a or C7a), 28.0 (C4 or C7), 26.8 (tBu), 24.0 (C2''), 22.9 (C4 or C7), 21.2 (Me), 19.2 (Me); LC-MS (ESI^+) *m/z* 553 [$M+Na^+$], R_t 2.37 min [method D]; HRMS [$M+Na^+$] calcd for $C_{36}H_{48}NO_2Si$ 554.3450 found 554.3452.

{2-[(1R,3S,3aS,7aR)-3-{[(*tert*-Butyldiphenylsilyl)oxy]methyl}-1,3,3a,4,7,7a-hexahydro-2-benzofuran-1-yl]-2-methylpropyl}[(3-methoxyphenyl)methyl]amine 51

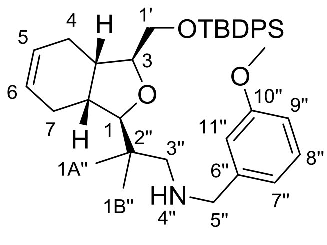

51

2-[(1R,3S,3aS,7aR)-3-{[(*tert*-Butyldiphenylsilyl)oxy]methyl}-1,3,3a,4,7,7a-hexahydro-2-benzofuran-1-yl]-2-methylpropanal **44** (145 mg, 0.315 mmol) and (3-methoxyphenyl)methanamine (40 µM, 0.315 mmol) was dissolved in 1,2-dichloroethane (3.0 mL). Sodium triacetoxyborohydride (86 mg, 0.441 mmol) was then added and the solution stirred at RT for 3 h. The reaction mixture was quenched by adding aqueous sat. $NaHCO_3$ (10 mL), and the solution was extracted with EtOAc. The EtOAc extract was dried ($MgSO_4$), and the crude residue was purified by ionic exchange chromatography on acidic resin (2 g) to yield **51** as a clear oil (115 mg, 62%)**.** $[\alpha]^{24.4}_D$ -33.7 (c=1.0 $CHCl_3$); IR (film) 2931, 2857, 1600, 1586, 1488 cm^{-1}; ^1H NMR (500 MHz, $CDCl_3$) δ 7.74-7.70 (4H, m, Ph), 7.45-7.36 (6H, m, Ph), 7.21 (1H, dd, J=8.0, 8.0, H-8’’), 6.96-6.89 (2H, m, H-9’’, H-11’’), 6.84-6.77 (1H, m, H-7’’), 5.83-5.76 (2H, m, H-5, H-6), 3.85-3.68 (8H, m, H-1’, H-5’’, OMe, H-3), 3.58-3.55 (1H, d, J=4.0, H-1), 2.60 (1H, d, J=11.0, Ha-3’’), 2.42 (1H, d, J=11.0, Hb-3’’), 2.43-2.13 (4H, m, H-3a, H-7a, Ha-4, Ha-7), 1.97-1.86 (2H, Hb-4, Hb-7), 1.07 (9H, s, tBu), 0.93 (3H, s, Me), 0.93 (3H, s, Me); ^{13}C NMR (125 MHz, $CDCl_3$) δ 159.7 (C8’’), 142. 7 (C6’’), 135.7 (2 x Ph), 135.6 (2 x Ph), 133.7 (iPh), 133.6 (iPh), 129.5 (Ph), 129.5 (Ph), 129.2 (C10’’), 127.6 (4 x Ph), 127.0 (C5 or C6), 126.8 (C5 or C6), 120.3 (C11’’), 113.3 (C7’’ or C9’’), 112.4 (C7’’ or C9’’), 91.3 (C1), 83.3 (C3), 65.4 (C1’), 58.3 (C3’’), 55.2 (C12’’), 54.0 (C5’’), 38.2 (C3a or C7a), 37.8 (C2’’), 36.9 (C3a or C7a), 28.0 (C4 or C7), 26.8 (tBu), 24.1 (C4 or C7), 22.9 (Me), 21.9 (Me), 19.2 (Si-C); LC-MS (ESI^+) *m/z* 584.37 $[M+H^+]$, R_t 2.33 min [method D]; HRMS $[M+H^+]$ calcd for $C_{37}H_{50}NO_3Si$ 584.3554 found 584.3568.

{2-[(1R,3S,3aS,7aR)-3-{[(*tert*-Butyldiphenylsilyl)oxy]methyl}-1,3,3a,4,7,7a-hexahydro-2-benzofuran-1-yl]-2-methylpropyl}(methyl)amine 52

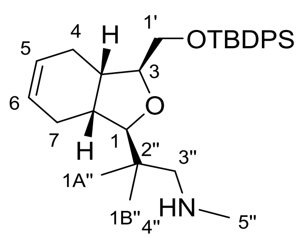

52

2-[(1R,3S,3aS,7aR)-3-{[(*tert*-Butyldiphenylsilyl)oxy]methyl}-1,3,3a,4,7,7a-hexahydro-2-benzofuran-1-yl]-2-methylpropanal **44** (145 mg, 0.315 mmol) and methylamine (25 µM, 0.315 mmol) was dissolved in 1,2-dichloroethane (3.0 mL). Sodium triacetoxyborohydride (86 mg, 0.441 mmol) was added and the solution stirred at RT for 3 h. The reaction mixture was quenched by adding aqueous sat. $NaHCO_3$ (10 mL), and the solution was extracted with EtOAc. The EtOAc extract was dried over $MgSO_4$, and the crude residue was purified by ionic exchange chromatography on acidic resin (2 g) to yield **52** as a clear oil (92 mg, 64%). $[\alpha]^{24.4}_D$ -28.5 (c=0.41 $CHCl_3$); IR (film) 2931, 2857, 1472, 1428 cm^{-1}; ^1H NMR (500 MHz, $CDCl_3$) δ 7.75-7.68 (4H, m, Ph), 7.45-7.36 (6H, m, Ph), 5.83-5.76 (2H, m, H-5, H-6), 3.79

(1H, dd, J=4.0, 11.0, Ha-1’), 3.75-3.68 (2H, m, Hb-1’, H-3), 3.55-3.51 (1H, d, J=4.0, H-1), 2.59 (1H, d, J=11.5, Ha-3’’), 2.42 (3H, s, H-5’’), 2.37 (1H, d, J=11.5, Hb-3’’), 2.24-2.14 (4H, m, H-3a, H-7a, Ha-4, Ha-7), 1.99-1.85 (2H, m, Hb-4, Hb-7), 1.06 (9H, s, tBu), 0.93 (3H, s, Me), 0.91 (3H, s, Me); ^{13}C NMR (125 MHz, $CDCl_3$) *δ* 135.7 (2 x Ph), 135.6 (2 x Ph), 133.8 (iPh), 133.6 (iPh), 129.6 (Ph), 129.5 (Ph), 127.6 (2 x Ph), 127.6 (2 x Ph), 127.0 (C5 or C6), 126.8 (C5 or C6), 91.4 (C1), 83.3 (C3), 65.2 (C1’), 61.9 (C3’’), 38.2 (C3a or C7a), 37.6 (C2’’), 37.6 (C5’’), 36.9 (C3a or C7a), 28.1 (C4 or C7), 26.8 (tBu), 24.0 (C4 or C7), 23.1 (Me), 21.1 (Me), 19.2 (Si-C); LC-MS (ESI$^+$) *m/z* 478 [M+H$^+$], R_t 2.28 min [method D]; HRMS [M+H$^+$] calcd for $C_{30}H_{44}NO_2$ 478.3135 found 478.3114.

{2-[(1R,3S,3aS,7aR)-3-{[(*tert*-Butyldiphenylsilyl)oxy]methyl}-1,3,3a,4,7,7a-hexahydro-2-benzofuran-1-yl]-2-methylpropyl}(propyl)amine 53

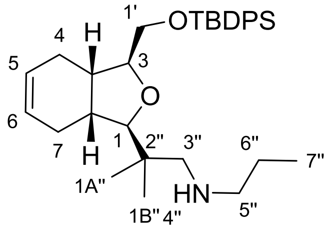

53

2-[(1R,3S,3aS,7aR)-3-{[(*tert*-Butyldiphenylsilyl)oxy]methyl}-1,3,3a,4,7,7a-hexahydro-2-benzofuran-1-yl]-2-methylpropanal **44** (145 mg, 0.315 mmol) and n-propylamine (25 µM, 0.315 mmol) was dissolved in 1,2-dichloroethane (3.0 mL). Sodium triacetoxyborohydride (86 mg, 0.441 mmol) was added and the solution stirred at RT for 3 h. The reaction mixture was quenched by adding aqueous sat. $NaHCO_3$ (10 mL), and the solution was extracted with EtOAc. The EtOAc extract was dried ($MgSO_4$), and the crude residue was purified by ionic exchange chromatography on acidic resin (2 g) to yield **53** as a clear oil (92 mg, 62%). $[\alpha]^{24.4}_D$ -21.3 (c=0.5 $CHCl_3$); IR (film) 2956, 2857, 1472 cm^{-1}; ^{1}H NMR (500 MHz, $CDCl_3$) *δ* 7.75-7.68 (4H, m, Ph), 7.46-7.36 (6H, m, Ph), 5.82-5.74 (2H, m, H-5, H-6), 3.82-3.74 (3H, m, H-1’, H-3), 3.56 (1H, d, J=4.0, H-1), 2.63-2.50 (3H, m, H-5’’, Ha-3’’), 2.42 (1H, d, J=11.5, Hb-3’’), 2.25-2.13 (4H, m, H-3a, H-7a, Ha-4, Ha-7), 2.00-1.84 (2H, m, Hb-4, Hb-7), 1.50 (2H, ddddd, J=7.0, 7.0, 7.0, 7.0, 7.0, H-6’’), 1.07 (9H, s, tBu), 0.91-0.89 (9H, m, Me, Me, H-7’’); ^{13}C NMR (125 MHz, $CDCl_3$) *δ* 135.6 (4 x Ph), 133.7 (iPh), 133.6 (iPh), 129.6 (Ph), 129.5 (Ph), 127.6 (4 x Ph), 127.0 (C5 or C6), 126.8 (C5 or C6), 91.5 (C1), 83.4 (C3), 65.2 (C1’), 59.0 (C3’’), 52.8 (C5’’), 38.3 (C3a or C4a), 37.6 (C2), 36.9 (C3a or C4a), 28.0 (C4 or C7), 26.0 (tBu), 24.0 (C4 or C7), 22.9 (C6’’), 22.9 (C1A’’ or C1B’’), 21.1 (C1A’’ or C1B’’), 19.2 (Si-C), 11.7 (C7’’); LC-MS (ESI$^+$) *m/z* 506 [M+H$^+$], R_t 2.30 min [method D]; HRMS [M+H$^+$] calcd for $C_{32}H_{48}NO_2Si$ Na 506.3449 found 506.3464.

{2-[(1R,3S,3aS,7aR)-3-{[(*tert*-Butyldiphenylsilyl)oxy]methyl}-1,3,3a,4,7,7a-hexahydro-2-benzofuran-1-yl]-2-methylpropyl}(2-methoxyethyl)amine 54

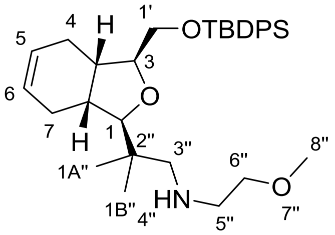

54

2-[(1R,3S,3aS,7aR)-3-{[(*tert*-Butyldiphenylsilyl)oxy]methyl}-1,3,3a,4,7,7a-hexahydro-2-benzofuran-1-yl]-2-methylpropanal **44** (145 mg, 0.315 mmol) and methoxyethyl amine (27 µM, 0.315 mmol) was dissolved in 1,2-dichloroethane (3.0 mL). Sodium triacetoxyborohydride (86 mg, 0.441 mmol) was added and the solution stirred at RT for 3 h. The reaction mixture was quenched by adding aqueous sat. $NaHCO_3$ (10 mL), and the solution was extracted with EtOAc. The EtOAc extract was dried ($MgSO_4$), and the crude residue was purified by ionic exchange chromatography on acidic resin (2 g) to yield **54** as a clear oil (92 mg, 56%). $[\alpha]^{24.3}_D$ -28.6 (c=1.0 $CHCl_3$); IR (film) 2930, 2857, 1472, 1428 cm^{-1}; ^1H NMR (500 MHz, $CDCl_3$) δ 7.74-7.65 (4H, m, Ph), 7.44-7.36 (6H, m, Ph), 5.82-5.74 (2H, m, H-5, H-6), 3.80-3.66 (3H, m, H-1', H-3), 3.56 (1H, d, J=4.0, H-1), 3.48 (2H, dd, J=5.5, 5.5, H-6''), 3.30 (3H, s, H-8''), 2.81-2.75 (2H, m, H-5''), 2.60 (1H, d J=11.5, Ha-3''), 2.42 (1H, d, J=11.5, Hb-3''), 2.25-2.09 (4H, m, H-3a, H-7a, Ha-4, Ha-7), 1.99-1.79 (2H, m, Hb-4, Hb-7), 1.05 (9H, s, tBu), 0.91 (3H, s, Me), 0.90 (3H, s, Me); ^{13}C NMR (125 MHz, $CDCl_3$) δ 135.6 (4 x Ph), 133.7 (iPh), 133.6 (iPh), 129.6 (Ph), 129.5 (Ph), 127.6 (4 x Ph), 127.0 (C5 or C6), 126.8 (C5 or C6), 91.2 (C1), 83.2 (C3), 72.0 (C6''), 65.2 (C1'), 58.9 (C3'' or C8''), 58.7 (C3'' or C8''), 50.4 (C5''), 38.3 (C3a or C7a), 37.8 (C2''), 36.8 (C3a or C7a), 28.0 (C4 or C7), 26.8 (tBu), 24.1 (C4 or C7), 22.3 (Me), 21.2 (Me), 19.2 (Si-C); LC-MS (ESI$^+$) *m/z* 522 [M+H$^+$], R_t 2.30 min [method D]; HRMS [M+H$^+$] calcd for $C_{32}H_{48}NO_3Si$ 522.3398 found 522.3409; Found: C, 73.75; H, 9.00%; $C_{32}H_{47}NO_3Si$ requires: C, 73.66; H, 9.08%.

[(1S,3R,3aR,7aS)-3-[1-(benzylamino)-2-methylpropan-2-yl]-1,3,3a,4,7,7a-hexahydro-2-benzofuran-1-yl]methanol 55

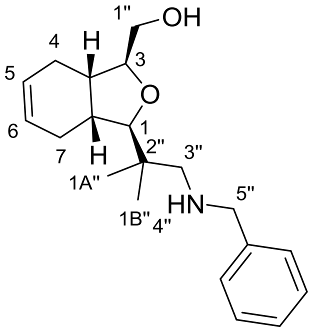

55

{2-[(1R,3S,3aS,7aR)-3-{[(*tert*-*b*utyldiphenylsilyl)oxy]methyl}-1,3,3a,4,7,7a-hexahydro-2-benzofuran-1-yl]-2-methylpropyl}(benzyl)amine **50** (50 mg, 0.090 mmol) was dissolved in THF (1 mL). TBAF (1.0 M in THF, 0.27 mL, 0.27 mmol) was added and the reaction stirred at 0°C for 6 h. The solution was diluted with Et_2O (20 mL) and washed with H_2O (2 x 20 mL), brine (20 mL) and the organic layer was dried with $MgSO_4$. The crude residue was purified by ionic exchange chromatography on acidic resin (2 g) to yield **55** as a clear oil (18 mg, 63%). $[\alpha]^{21}_D$ -10.5 (c=1.0 $CHCl_3$); IR (film) 3319, 2930, 2839, 1495, 1453 cm^{-1}; ^1H NMR (500 MHz, $CDCl_3$) δ 7.35-7.34 (4H, m, Ph), 7.29-7.24 (1H, m, Ph), 5.81-5.74 (2H, m, H-5, H-6), 3.86-3.81 (2H, m, Ha-1', Ha-5''), 3.73 (1H, d, J=13.0, Hb-5''), 3.68 (1H, ddd, J=3.0, 3.0, 8.5, H-3), 3.56-3.52 (2H, m, Hb-1', H-1), 2.61 (1H, d, J=12.0, Ha-3''), 2.38 (1H, d, J=12.0, Hb-3''), 2.34-2.15 (4H, m, H-7a, H-3a, Ha-4, Ha-7), 1.97-1.83 (2H, m, Hb-4, Hb-7), 1.00 (3H, s, Me), 0.83 (3H, s, Me); ^{13}C NMR

(125 MHz, $CDCl_3$) δ 128.4 (4x Ph), 128.3 (Ph), 127.2 (Ph), 126.5 (C5 or C6), 126.4 (C5 or C6), 92.7 (C1), 83.6 (C3), 62.5 (C1′), 57.3 (C3′′), 24.5 (C5′′), 37.5 (C2′′), 37.0 (C3a or C7a), 36.2 (C3a or C7a), 27.9 (C4 or C7), 23.9 (C4 or C7), 23.8 (Me), 23.2 (Me); LC-MS (ESI^+) *m/z* 316 [$M+H^+$], R_t 1.73 min [method D]; HRMS [$M+H^+$] calcd for $C_{20}H_{30}NO_2$ 316.2271 found 316.2278.

[(1S,3R,3aR,7aS)-3-(1-{[(3-Methoxyphenyl)methyl]amino}-2-methylpropan-2-yl)-1,3,3a,4,7,7a-hexahydro-2-benzofuran-1-yl]methanol 56

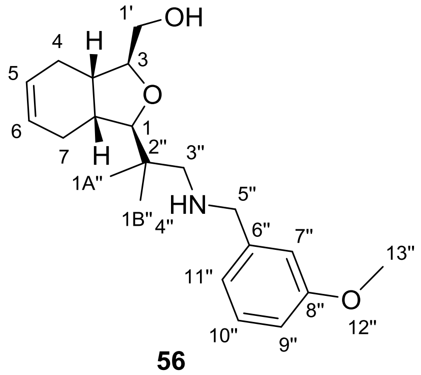

56

{2-[(1R,3S,3aS,7aR)-3-{[(*tert*-Butyldiphenylsilyl)oxy]methyl}-1,3,3a,4,7,7a-hexahydro-2-benzofuran-1-yl]-2-methylpropyl}[(3-methoxyphenyl)methyl]amine **51** (69 mg, 0.118 mmol), was dissolved in THF (1.3 mL). TBAF (1.0 M in THF, 0.35 mL, 0.35 mmol) was added and the reaction stirred at 0 °C for 6 h. The solution was diluted with Et_2O (20 mL) and washed with H_2O (2 x 20 mL), brine (20 mL) and the organic layer was dried with $MgSO_4$. The crude residue was purified by ionic exchange chromatography on acidic resin (2 g) to yield **56** as a clear oil (26 mg, 63%). $[\alpha]^{21}_D$ -6.7 (c=2.0 $CHCl_3$); IR (film) 2938, 1600, 1489, 1466, 1252 cm^{-1}; ^1H NMR (500 MHz, $CDCl_3$) δ 7.29-7.24 (1H, m, Ph), 7.00-6.92 (2H, m, Ph), 6.85-6.80 (1H, m, Ph), 5.82-5.77 (2H, m, H-5, H-6), 3.89-3.79 (6H, m, Ha-1′, H-3′′), 3.76 (3H, s, H-13′′), 3.70-3.65 (1H, m, H-3), 3.57-3.52 (2H, m, Hb-1′, H-1), 2.66 (1H, d, J=12.0, Ha-5′′), 2.42 (1H, d, J=12.0, Hb-5′′), 2.32-2.14 (4H, m, H-3a, H-7a, Ha-4, Hb-7), 1.96-1.83 (2H, m, H-4, H-7), 1.01 (3H, s, Me), 0.92 (3H, s, Me); ^{13}C NMR (125 MHz, $CDCl_3$) δ 159.3 (C8′′), 128.9 (Ph), 126.1 (C5 or C6), 125.8 (C5 or C6), 120.8 (Ph), 119.9 (Ph), 113.5 (Ph), 113.1 (Ph), 92.3 (C1), 83.2 (C3), 61.9 (C1′), 56.5 (C5′′), 54.7 (C13′′), 53.6 (C3′′), 36.9 (C2′′), 36.5 (C3a or C7a), 35.8 (C3a or C7a), 27.4 (C4 or C7), 23.3 (C4 or C7), 23.0 (Me), 22.3 (Me); LC-MS (ESI^+) *m/z* 346 [$M+H^+$], R_t 1.53 min [method D]; HRMS [$M+H^+$] calcd for $C_{21}H_{32}NO_3$ 346.2376 found 346.2386.

[(1S,3R,3aR,7aS)-3-[2-Methyl-1-(methylamino)propan-2-yl]-1,3,3a,4,7,7a-hexahydro-2-benzofuran-1-yl]methanol 57

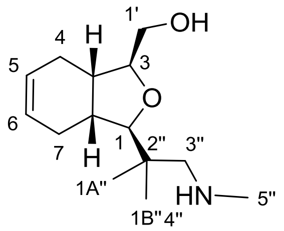

57

{2-[(1R,3S,3aS,7aR)-3-{[(*tert*-Butyldiphenylsilyl)oxy]methyl}-1,3,3a,4,7,7a-hexahydro-2-benzofuran-1-yl]-2-methylpropyl}(methyl)amine **52** (84 mg, 0.176 mmol), was dissolved in THF (2 mL). TBAF (1.0 M in THF, 0.53 mL, 0.53 mmol) was added and the reaction stirred at 0 °C for 4 h. The solution was diluted with

Et_2O (20 mL) and washed with H_2O (2 x 20 mL), brine (20 mL) and the organic layer was dried with $MgSO_4$. The crude residue was purified by ionic exchange chromatography on acidic resin (2 g) to yield **57** as a clear oil (25 mg, 60%). $[\alpha]^{21}_D$ -16.1 (c=1.0 $CHCl_3$); IR (film) 3327, 3027, 2932, 1472, 1443 cm^{-1}; ^1H NMR (500 MHz, $CDCl_3$) δ 5.83-5.72 (2H, m, H-5, H-6), 3.86 (1H, dd, J=2.5, 12.0, Ha-1'), 3.68 (1H, ddd, J=2.5, 2.5, 9.0, H-3), 3.54-3.50 (2H, m, Hb-1', H-1), 2.58 (1H, d, J=12.0, Ha-3''), 2.36 (3H, s, H-5''), 2.35-2.16 (5H, m, Hb-3'', H-3a, H-7a, Ha-4, Ha-7), 1.98-1.82 (2H, m, Hb-4, Hb-7), 1.04 (3H, s, Me, 0.87 (3H, s, Me); ^{13}C NMR (125 MHz, $CDCl_3$) δ 126.4 (C5 or C6), 126.1 (C5 or C6), 93.1 (C1), 83.7 (C3), 62.1 (C1'), 60.5 (C3''), 37.5 (C2''), 27.1 (C7a or C3a), 36.9 (C5''), 35.0 (C3a or C7a), 28.0 (C4 or C7), 24.4 (Me), 23.9 (C4 or C7), 23.8 (Me); LC-MS (ESI$^+$) *m/z* 240 [M+H$^+$], R_t 1.43 min [method D]; HRMS [M+H$^+$] calcd for $C_{14}H_{26}NO_2$ 240.1958 found 240.1967.

[(1S,3R,3aR,7aS)-3-[2-methyl-1-(propylamino)propan-2-yl]-1,3,3a,4,7,7a-hexahydro-2-benzofuran-1-yl]methanol 58

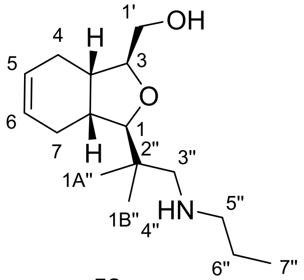

58

{2-[(1R,3S,3aS,7aR)-3-{[(*tert*-Butyldiphenylsilyl)oxy]methyl}-1,3,3a,4,7,7a-hexahydro-2-benzofuran-1-yl]-2-methylpropyl}(propyl)amine **53** (84 mg, 0.144 mmol), was dissolved in THF (2 mL). TBAF (1.0 M in THF, 0.43 mL, 0.43 mmol) was added and the reaction stirred at 0 °C for 4 h. The solution was diluted with Et_2O (20 mL) and washed with H_2O (2 x 20 mL), brine (20 mL) and the organic layer was dried with $MgSO_4$. The crude residue was purified by ionic exchange chromatography on acidic resin (2 g) to yield **58** as a clear oil (36 mg, 93%). $[\alpha]^{21}_D$ -25.6 (c=1.0 $CHCl_3$); IR (film) 3313, 3027, 2931, 2872, 1467 cm^{-1}; ^1H NMR (500 MHz, $CDCl_3$) δ 5.83-5.74 (2H, m, H-5, H-6), 3.88 (1H, dd, J=2.0, 12.0, Ha-1'), 3.68 (1H, ddd, J=2.0, 2.0, 9.0, H-3), 3.57-3.52 (2H, m, Hb-1', H-1), 2.69 (1H, d, J=12.0, Ha-3''), 2.66-2.59 (1H, m, Ha-5''), 2.55-2.49 (1H, m, Hb-5''), 2.40 (1H, d, J=12.0, Hb-3''), 2.32-2.17 (4H, m, Ha-4, Ha-7, H-3a, H-7a), 1.98-1.83 (2H, m, Hb-4, Hb-7), 1.67-1.50 (2H, m, H-6''), 1.06 (3H, s, Me), 0.94 (3H, s, Me), 0.92 (3H, t, J=7.0, J-7''); ^{13}C NMR (125 MHz, $CDCl_3$) δ 126.7 (C5 or C6), 126.0 (C5 or C6), 93.7 (C1), 84.1 (C3), 61.2 (C1'), 58.8 (C3''), 51.7 (C5''), 37.2 (C3a or C7a), 36.9 (C2''), 35.5 (C3a or C7a), 28.0 (C4 or C7), 24.5 (Me), 23.6 (C4 or C7), 21.6 (Me), 20.4 (C6''), 11.4 (C7''); LC-MS (ESI$^+$) *m/z* 268 [M+H$^+$], R_t 1.55 min [method D]; HRMS [M+H$^+$] calcd for $C_{16}H_{31}NO_2$ 268.2271 found 268.2273.

[(1S,3R,3aR,7aS)-3-{1-[(2-Methoxyethyl)amino]-2-methylpropan-2-yl}-1,3,3a,4,7,7a-hexahydro-2-benzofuran-1-yl]methanol 59

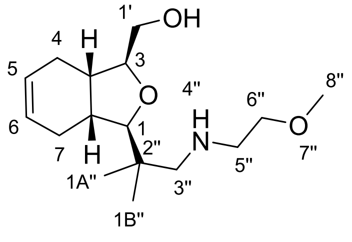

59

{2-[(1R,3S,3aS,7aR)-3-{[(tert-Butyldiphenylsilyl)oxy]methyl}-1,3,3a,4,7,7a-hexahydro-2-benzofuran-1-yl]-2-methylpropyl}(2-methoxyethyl)amine **54** (94 mg, 0.180 mmol), was dissolved in THF (2 mL). TBAF (1.0 M in THF, 0.54 mL, 0.54 mmol) was added and the reaction stirred at 0 °C for 6 h. The solution was diluted with Et_2O (20 mL) and washed with H_2O (2 x 20 mL), brine (20 mL) and the organic layer was dried with $MgSO_4$. The crude residue was purified by ionic exchange chromatography on acidic resin (2 g) to yield **59** as a clear oil (25 mg, 49%). $[\alpha]^{21}_D$ -21.2 (c=1.0 $CHCl_3$); IR (film) 3331, 2875, 2839, 1457, 1097 cm^{-1}; ^1H NMR (500 MHz, $CDCl_3$) δ 5.81-5.76 (2H, m, H-5, H-6), 3.87 (1H, dd, J=2.0, 12.0, Ha-1') 3.69 (1H, ddd, J=3.0, 3.0, 9.0, H-3), 3.57-3.51 (4H, Hb-1', H-1, H-6''), 3.38 (3H, s, H-8''), 2.77-2.72 (2H, m, H-5''), 2.66 (1H, d, J=12.0, Ha-3''), 2.43 (1H, d, J=12.0, Hb-3''), 2.35-2.31 (1H, m, H-3a), 2.30-2.17 (3H, m, Ha-4, Ha-7, H-7a), 1.98-1.84 (2H, m, Hb-4, Hb-7), 1.06 (3H, s, Me), 0.92 (3H, s, Me); ^{13}C NMR (125 MHz, $CDCl_3$) δ 126.0 (C5 or C6), 125.7 (C5 or C6), 92.9 (C3), 83.3 (C1), 70.6 (C6''), 61.8 (C1'), 58.3 (C8''), 57.9 (C3''), 49.6 (C5''), 36.9 (C2''), 36.6 (C7a), 35.4 (C3a), 27.5 (C4 or C7), 23.6 (Me), 23.4 (C4 or C7), 23.2 (Me); LC-MS (ESI^+) *m/z* 284 [$M+H^+$], R_t 1.52 min [method D]; HRMS [$M+H^+$] calcd for $C_{16}H_{30}NO_3$ 284.2220 found 284.2230; Found: C, 67.90; H, 10.36%; $C_{16}H_{29}NO_3$ requires: C, 67.81; H, 10.31%.

[(1S,3S,3aR,7aS)-3-(2-methoxy-3-phenylpropyl)-octahydro-2-benzofuran-1-yl]methanol 60

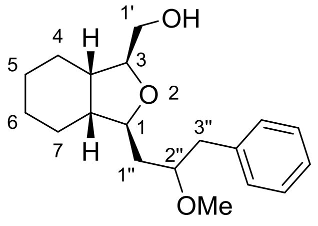

60

Trimethyl(phenyl)silane (0.155 mL, 0.903 mmol), Ph_3PAuCl (11 mg, 0.023 mmol) and Selectfluor (346 mg, 0.903 mmol) was added to a solution of ((1*S*,3*S*,3a*R*,7a*S*)-3-allyloctahydro-isobenzofuran-1-yl)methanol **25** (196 mg, 1.000 mmol) in MeOH:MeCN (1:5, 4.8 mL) and heated to 70 °C for 5 h. The reaction was the cooled to RT and TBAF (1.0 M in THF, 1.35 mL, 1.35 mmol) was added. The resultant solution was stirred at RT for 16 h. The reaction mixture was then diluted with Et_2O (10 mL) and washed with H_2O (10 mL x 2). Solvents were removed *in vacuo* and the crude residue was purified by column chromatography on silica gel (15% EtOAc:hexane) to yield **60** as a mixture of diastereoisomers (39 mg, 29%). IR (film) 3406, 3028, 2926, 1451 cm^{-1}; ^1H NMR (500 MHz, $CDCl_3$) δ 7.34-7.27 (2H, m, Ph), 7.25-7.20 (3H, m, Ph), 4.00 (0.8H, ddd, J=4.0, 4.0, 8.5, H-1), 3.93 (0.2H, ddd, J=3.5, 3.5, 8.5, H-3), 3.88-3.84 (0.8H, m, H-3), 3.80 (0.2H, ddd, J=4.0, 4.0, 10.0, H-1), 3.78 (0.2H, dd, J=3.0, 11.5, Ha-1'), 3.66-3.63 (0.8H, m, H-2''), 3.60-3.54 (1H, m, Ha-1', H-2''), 3.48-3.41 (0.2H, m, Hb-1'), 3.42 (2.4H, s, OMe), 3.36 (0.6H, s, OMe), 3.30-3.23 (0.8H, m, Hb-1'), 2.99 (0.8H, dd, J=5.5, 13.5, Ha-3''), 2.91 (0.2H, dd, J=5.5, 13.5, Ha-3''), 2.75 (0.2H, dd, J=7.0, 13.5, Hb-3''), 2.69 (0.8H, dd, J=7.5, 14.0, Hb-3''), 2.35-2.28 (0.2H, m, H-3a),

2.08-1.98 (0.8H, m, H-3a), 1.85-1.77 (1H m, H-7a), 1.68-1.24 (10H, m, H-4, H-5. H-6, H-7, H-1''); ^{13}C NMR (125 MHz, $CDCl_3$) δ **MAJOR**: 139.3 (iPh), 130.3 (2 x Ph), 129.1 (2 x Ph), 127.0 (Ph), 82.7 (C3), 80.6 (C1), 80.5 (C2''), 65.0 (C1'), 58.1 (OMe), 44.4 (C7a), 41.5 (C1''), 40.8 (C3''), 38.3 (C3a), 27.2 (CH_2), 26.1 (CH_2), 24.3 (CH_2), 23.4 (CH_2), **MINOR**: 139.0 (iPh), 130.1 (2 x Ph), 129.0 (2 x Ph), 126.9 (Ph), 83.1 (C3), 82.1 (C2''), 82.5 (C1), 63.6 (C1'), 57.5 (OMe), 44.6 (C7a), 41.1 (C3''), 40.4 (C1''), 36.8 (C3a), 27.7 (CH_2), 25.5 (CH_2), 24.8 (CH_2), 22.6 (CH_2); LC-MS (ESI^+) *m/z* 327 [$M+Na^+$], R_t 1.52 min [method E].

Ethyl (2E)-4-[(1R,3S,3aS,7aR)-3-{[(*tert*-Butyldiphenylsilyl)oxy]methyl}-1,3,3a,4,7,7a-hexahydro-2-benzofuran-1-yl]-4-methylpent-2-enoate 61

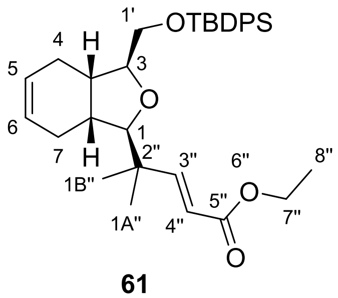

61

NaH (60 mg, 60% w/w mineral oil, 1.519 mmol) was added to THF (5 mL) to form a suspension. Triethylphosphonate (370 µL, 1.870 mmol) was added dropwise followed by a solution of 2-[(1R,3S,3aS,7aR)-3-[(*tert*-butyldiphenylsilyl)oxy]methyl}-1,3,3a,4,7,7a-hexahydro-2-benzofuran-1-yl]-2-methylpropanal **44** (469 mg, 1.167 mmol) in THF (7 mL). The reaction was stirred at RT for 16 h. The reaction was then diluted in Et_2O (50 mL), washed with H_2O (50 mL x 2) and brine (50 mL). The combined aqueous layers were extracted into CH_2Cl_2, and the organic layers dried over $MgSO_4$. Solvents were removed *in vacuo* to yield the crude product as a clear oil (660 mg). The product was purified by column chromatography on silica gel (5% EtOAc:hexane) to yield **61** as a clear oil (361 mg, 58%). $[\alpha]^{24.7}_D$ -22.9 (c=1.0 $CHCl_3$); IR (film) 2931, 2858, 1716 cm^{-1}; ^1H NMR (500 MHz, $CDCl_3$) δ 7.76-7.69 (4H, m, Ph), 7.47-7.37 (6H, m, Ph), 7.03 (1H, d, J=16.0, H-3''), 5.81 (1H, d, J=16.0, H-4''), 5.80-5.77 (2H, m, H-5, H-6), 4.19 (2H, dq, J= 1.5, 7.0, H-7''), 3.80-3.72 (3H, m, H-1', H-3), 3.51 (1H, J=5.0, H-1), 2.24-2.07 (4H, m, H-3a, H-7a, Ha-4, Hb-7), 1.95-1.88 (2H, m, Hb-4, Hb-7), 1.28 (3H, t, J=7.0, H-8''), 1.11 (3H, s, H-1A'' or H-1B''), 1.10 (3H, s, H-1A'' or H-1B''), 1.08 (9H, s, ^{t}Bu); ^{13}C NMR (125 MHz, $CDCl_3$) δ 166.9 (C5''), 155.4 (C3''), 135.7 (2 x Ph), 135.6 (2 x Ph), 133.7 (iPh), 133.6 (iPh), 129.6 (Ph), 129.6 (Ph), 127.8 (2 x Ph), 127.7 (2 x Ph), 126.9 (C5 or C6), 126.4 (C5 or C6), 119.0 (C4''), 91.6 (C1), 83.7 (C3), 65.3 (C1'), 60.2 (C7''), 40.8 (C2''), 38.1 (C3a or C7a), 37.2 (C3a or C7a), 27.6 (C4 or C7), 26.8 (^{t}Bu), 24.1 (C4 or C7), 23.4 (Me), 22.6 (Me), 19.2 (Si-C), 14.3 (C8''); LC-MS (ESI^+) *m/z* 555 [$M+Na^+$], R_t 3.15 min [method E]; HRMS [$M+H^+$] calcd for $C_{33}H_{44}O_4SiNa$ 555.2901 found 555.2917.

Ethyl 4-[(1R,3S,3aS,7aR)-3-{[(*tert*-butyldiphenylsilyl)oxy]methyl} octahydro-2-benzofuran-1-yl]-4-methylpentanoate 62

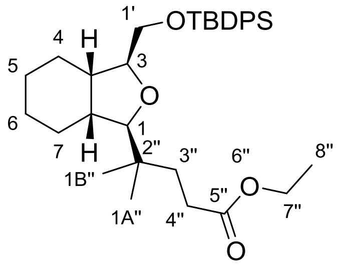

62

Ethyl (2E)-4-[(1R,3S,3aS,7aR)-3-{[(*tert*-*b*utyldiphenylsilyl)oxy]methyl}-1,3,3a,4,7,7a-hexahydro-2-benzofuran-1-yl]-4-methylpent-2-enoate **61** (587 mg, 1.105 mmol) was dissolved in EtOAc (11 mL). Palladium on activated carbon (30 mg, 10% w/w Pd) was added. The flask was evacuated and flushed with H_2, and left to stir under an atmosphere of H_2 for 5 d. The suspension was filtered through celite and the solvents removed *in vacuo* to yield **62** as a clear oil (592 mg, 97%). $[\alpha]^{24.7}_{D}$ -7.0 (c=1.0 $CHCl_3$); IR (film) 2930, 2858, 1733, 1472 cm^{-1}; ^{1}H NMR (500 MHz, $CDCl_3$) δ 7.66-7.60 (4H, m, Ph), 7.36-7.27 (6H, m, Ph), 4.03 (2H, q, J=7.5, H-7''), 3.84 (1H, ddd, J=4.5, 9.0, 9.0, H-3), 3.71 (1H, dd, J=4.0, 11.0, Ha-1'), 3.59 (1H, dd, J=4.5, 11.0, Hb-1'), 3.27 (1H, d, J=2.5, H-1), 2.29 (1H, dd, J=5.0, 10.5, Ha-4''), 2.28 (1H, J=6.0, 10.5, Hb-4''), 1.99-1.91 (2H, m, H-3a, H-7a), 1.64-1.45 (8H, m, 8 x CH_2), 1.49-1.20 (2H, m, 2 x CH_2), 1.16 (3H, t, J=7.5, H-8''), 1.01 (9H, s, tBu), 0.77 (3H, s, Me), 0.75 (3H, s, Me); ^{13}C NMR (125 MHz, $CDCl_3$) δ 174.5 (C5''), 135.7 (2 x Ph), 135.6 (2 x Ph), 133.8 (iPh), 133.6 (iPh), 129.5 (Ph), 129.5 (Ph), 127.6 (4 x Ph), 91.9 (C1), 80.1 (C3), 65.4 (C1'), 60.2 (C7''), 38.8 (C3a, C7a), 36.6 (C2''), 34.0 (CH_2), 30.3 (CH_2), 29.5 (CH_2), 26.8 (tBu), 24.6 (2 × CH_2), 24.0 (CH_2), 22.6 (Me), 22.4 (Me), 22.1 (CH_2), 19.21 (C-Si), 14.2 (C8''); LC-MS (ESI$^+$) *m/z* 559 [M+Na$^+$], R_t 3.50 min [method E]; HRMS [M+Na$^+$] calcd for $C_{33}H_{48}O_4SiNa$ 559.3214 found 559.3224; Found: C, 74.02; H, 8.91%; $C_{33}H_{48}O_4Si$ requires: C, 73.83; H, 9.01%.

Ethyl 4-[(1R,3S)-3-(hydroxymethyl)octahydro-2-benzofuran-1-yl]-4-methylpentanoate 63

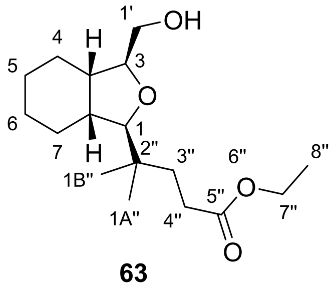

63

Ethyl 4-[(1R,3S,3aS,7aR)-3-{[(*tert*-butyldiphenylsilyl)oxy]methyl}octahydro-2-benzofuran-1-yl]-4-methylpentanoate **62** (221 mg, 0.412 mmol) was dissolved in THF (4 mL). TBAF (1.0 Mol in THF, 1.23 mL, 1.23 mmol) was added and the reaction stirred at 0 °C for 4 h. The solution was diluted with Et_2O (20 mL) and washed with H_2O (2 x 20 mL), brine (20 mL) and the organic layer was dried with $MgSO_4$. The product was purified by column chromatography on silica gel (15% EtOAc:hexane) to yield **63** as a clear oil (89 mg, 72%). $[\alpha]^{24.6}_{D}$ +7.3 (c=3.0 $CHCl_3$); IR (film) 2931, 1723, 1450 cm^{-1}; ^{1}H NMR (500 MHz, $CDCl_3$) δ 4.11 (2H, dq, J= 1.5, 7.0, H-7''), 3.89 (1H, ddd, J=3.0, 6.0, 10.5, H-3), 3.76 (1H, br d, J=11.5, Ha-1'), 3.51 (1H, dd, J=6.0, 11.5, Hb-1'), 3.35 (1H, d, J=3.0, H-1), 2.32 (1H, ddd, J=6.5, 10.5, 15.5, Ha-4''), 2.27 (1H, ddd, J=6.5, 10.5, 15.5, Hb-4''), 2.07-1.99 (1H, m, H-7a), 1.92-1.86 (1H, m, H-3a), 1.67-1.52 (6H, m, CH_2), 1.50-1.44 (1H, m, CH_2), 1.41-1.20 (3H, m, CH_2), 1.24 (3H, t, J=7.0, H-8''), 0.83 (3H, s, Me), 0.81 (3H, s, Me); ^{13}C NMR (125 MHz, $CDCl_3$) δ 174.4 (C5''), 92.3 (C1), 79.9 (C3), 64.2 (C1'), 60.3 (C4'), 39.1 (C7a), 38.1 (C3a), 36.5 (C2''), 34.0 (CH_2), 30.3 (CH_2), 29.5 (CH_2), 24.5 (CH_2), 23.8 (CH_2), 22.9 (Me), 22.3 (Me), 21.9

(CH_2), 14.2 (C8''); LC-MS (ESI^+) *m/z* 321.24 [$M+Na^+$], R_t 1.72 min [method E]; HRMS [$M+H^+$] calcd for $C_{17}H_{30}O_4H$ 298.2144 found 298.2151;

4-[(1R,3S,3aS,7aR)-3-(hydroxymethyl)octahydro-2-benzofuran-1-yl]-4-methylpentanoic acid 64

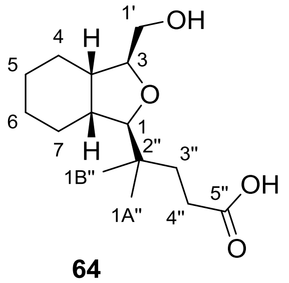

64

Ethyl 4-[(1R,3S)-3-(hydroxymethyl)octahydro-2-benzofuran-1-yl]-4-methylpentanoate **63** (95 mg, 0.319 mmol) was dissolved in THF:H_2O (3.0 mL, 2:1 ratio) and LiOH.H_2O (23 mg, 7.9 mmol) was added. The solution was stirred at RT for 16 h. The reaction mixture was acidified to pH 4 with 1 M HCl. The solution was extracted with CH_2Cl_2 (4 x 5 mL). The combined organic layers were dried over $MgSO_4$ and the solvents removed *in vacuo* to yield **64** as a clear oil (81 mg, 99%). $[\alpha]^{24.2}_D$ +17.8 (c=1.0 $CHCl_3$); IR (film) 2928, 2858, 1708, 1451 cm^{-1}; ^1H NMR (500 MHz, $CDCl_3$) δ 3.96 (1H, ddd, J=2.5, 6.0, 10.0, H-3), 3.77 (1H, dd, H=2.5, 12.0, Ha-1'), 3.55 (1H, dd, J=6.0, 12.0, Hb-1'), 3.40 (1H, d, J=3.0, H-1), 2.37 (1H, ddd, J=6.0, 12.0, 16.0, Ha-4''), 2.30 (1H, ddd, J=6.0, 12.0, 16.0, Hb-4''), 2.09-2.02 (1H, m, H-7a), 1.95-1.88 (1H, m, H-3a), 1.71-1.46 (7H, m, H-3'', 3 x CH_2), 1.42-1.21 (3H, m, CH_2), 0.85 (3H, s, H-1A''), 0.84 (3H, s, H-1B''); ^{13}C NMR (125 MHz, $CDCl_3$) δ 178.5 (C5''), 92.3 (C1), 80.5 (C3), 64.2 (C1'), 39.1 (C7a), 38.0 (C3a), 36.4 (C2''), 33.9 (C3''), 30.3 (CH_2), 29.0 (C4''), 24.5 (CH_2), 23.7 (CH_2), 23.1 (C1A''), 22.1 (C1B''), 21.8 (CH_2); LC-MS (ESI^+) *m/z* 293 [$M+Na^+$], R_t 1.58 min [method D lipoII]; HRMS [$M+Na^+$] calcd for $C_{15}H_{26}O_4Na$ 293.1723 found 293.1726; Found: C, 66.59 H, 9.59%; $C_{15}H_{26}O_4$ requires: C, 66.64; H, 9.69%.

(1R,2R,7S,8S)-14,14-dimethyl-10,15-dioxatricyclo[6.6.1.0$^{\{2,7\}}$]pentadecan-11-one 65

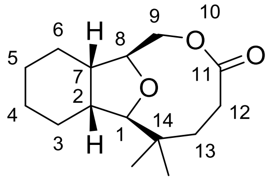

65

2-Methyl-6-nitrobenzoic anhydride (87 mg, 0.253 mmol) and DMAP (61 mg, 0.506 mmol) was dissolved in CH_2Cl_2 (90 mL). A solution of 4-[(1R,3S,3aS,7aR)-3-(hydroxymethyl)octahydro-2-benzofuran-1-yl]-4-methylpentanoic acid **64** (57 mg, 0.211 mmol) in CH_2Cl_2 (20 mL, 0.01 M) was added by syringe pump over 16 h at RT. Approximately 50% of the solvent was removed *in vacuo* and the remaining solution was washed with ice cold sat. aqueous $NaHCO_3$. The aqueous layer was extracted with CH_2Cl_2 (2 x 30 mL), and the combined organic layers were washed with brine (30 mL). The organic layer was dried with $MgSO_4$ and the solvents were removed *in vacuo*. The crude residue was purified by column chromatography on silica gel (5% MeOH:CH_2Cl_2) to yield the product as a white crystalline solid (18 mg, 30%). mp (56.6-57.1 ˚C); $[\alpha]^{22}_D$ +14.0 (c=2.0 $CHCl_3$); IR (film) 2923, 2860, 1730, 1447 cm^{-1}; ^1H NMR (500 MHz, $CDCl_3$) δ 5.12 (1H, dd, J=4.0, 11.5, Ha-9), 3.90 (1H, dd, J=4.0, 8.0, H-8), 3.70 (1H, d, J=11.5, Hb-9), 3.50 (1H, d, J=3.0, H-1), 2.55-2.48

(1H, m, H-7), 2.48-2.44 (2H, m, H-12), 2.27-2.20 (1H, m, H-2), 2.05 (1H, ddd, J=6.5, 10.5, 15.0, Ha-13), 1.69-1.60 (3H, m, CH_2), 1.62-1.47 (2H, m, CH_2), 1.40-1.09 (4H, m, CH_2), 1.09 (3H, s, Me), 0.86 (3H, s, Me); ^{13}C NMR (125 MHz, $CDCl_3$) δ 175.7 (C11), 95.7 (C1), 79.5 (C8), 65.4 (C9), 39.0 (C2 or C7), 38.8 (C2 or C7), 37.3 (C14), 33.9 (C12), 32.5 (C13), 30.1 (CH_2), 29.1 (Me), 25.4 (Me), 24.3 (CH_2), 24.0 (CH_2), 22.1 (CH_2); LC-MS (ESI$^+$) *m/z* 253 [M+H$^+$], R_t 1.63 min [method D]; HRMS [M+H$^+$] calcd for $C_{15}H_{25}O_3$ 253.1798 found 253.1799; Found: C, 71.45; H, 9.42%; $C_{15}H_{24}O_3$ requires: C, 71.39; H, 9.59%.

2-[(1R,3S,3aS,7aR)-3-{[(*tert-b*utyldiphenylsilyl)oxy]methyl}-octahydro-2-benzofuran-1-yl]-2-methylpropanal 66

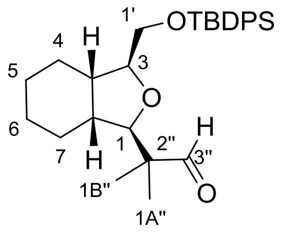

66

(3S,3aS,7aR)-3-{[(*tert*-Butyldiphenylsilyl)oxy]methyl}-octahydro-2-benzofuran-1-ol **23** (841 mg, 2.05 mmol) was dissolved in CH_2Cl_2 (20 mL) and cooled to -78 °C. $BF_3.OEt_2$ (0.78 mL, 6.15 mmol) was added and the solution was stirred for 5 min. Trimethyl(2-methylprop-1-enyloxy)silane (0.41 mL, 2.25 mmol) was then added and the reaction was warmed to RT and stirred for 3 h. The reaction was diluted with CH_2Cl_2 (20 mL), and washed with H_2O (2 x 50 mL), followed by brine (50 mL). The organic layer was dried with $MgSO_4$. Solvent was removed *in vacuo* to yield **66** as a pale brown oil (950 mg, >98%). This compound was found to be unstable so was used immediately. ^1H NMR (500 MHz, $CDCl_3$) δ 9.63 (1H, s, H-3''), 7.75-7.64 (4H, m, Ph), 7.46-4.36 (6H, m, Ph), 3.96-3.89 (1H, m, H-3), 3.79-3.75 (2H, m, Ha-1', H-1), 3.65 (1H, dd, J=5.0, 11.5, Hb-1'), 2.06-2.01 (2H, m, H-3a, H-7a), 1.70-1.56 (4H, 4 x CH_2), 1.49-1.37 (2H, m, 2 x CH_2), 1.37-1.21 (2H, m, 2 x CH_2), 1.07 (3H, s, Me), 1.06 (9H, s, tBu), 1.06 (3H, s, Me); ^{13}C NMR (125 MHz, $CDCl_3$) δ 206.1 (C3'') 135.7 (2 x Ph), 135.6 (2 x Ph), 133.6 (iPh), 133.4 (iPh), 129.6 (Ph), 129.6 (Ph), 127.7 (4 x Ph), 88.3 (C1), 80.9 (C3), 64.9 (C1'), 49.9 (C2''), 39.0 (C3a or C7a), 38.6 (C3a or C7a), 29.4 (CH_2), 26.8 (tBu), 24.2 (CH_2), 24.0 (CH_2), 22.2 (CH_2), 19.2 (Me) 18.8 (C-Si), 17.6 (Me); LC-MS (ESI$^+$) *m/z* 487 [M+Na$^+$], R_t 1.92 min [method E]; HRMS [M+Na$^+$] calcd for $C_{29}H_{40}O_3SiNa$ 487.2644 found 487.2639.

[(1S,3R,3aR,7aS)-3-(2-methyl-1-oxopropan-2-yl)octahydro-2-benzofuran-1-yl]methyl 2-bromoacetate 68

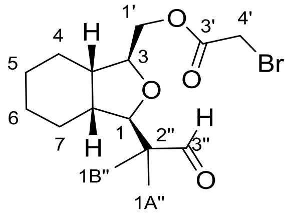

68

2-[(1R,3S,3aS,7aR)-3-{[(*tert*-Butyldiphenylsilyl)oxy]methyl}-octahydro-2-benzofuran-1-yl]-2-methylpropanal **66** (950 mg, 2.05 mmol) was dissolved in THF (20 mL). TBAF (1.0 M in THF, 6.2 mL, 6.2 mmol) was added and the reaction stirred at RT for 8 h. The solution was diluted with Et_2O (30 mL) and washed with H_2O (2 x 30 mL) and

the organic layer was dried with $MgSO_4$. The product was purified by column chromatography on silica gel (33% EtOAc:hexane) to yield the alcohol **67** as a clear oil (200 mg, 43%). This unstable product was taken immediately into the next step. The alcohol **67** (200 mg, 0.89 mmol) was dissolved in CH_2Cl_2 (9 mL) and Et_3N (257 µL, 1.78 mmol) was added. 2-Bromoacetyl bromide (154 µL, 1.78 mmol) was added and the solution was stirred at RT for 5 h. The solution was diluted with CH_2Cl_2 (20 mL), washed with H_2O (10 mL), brine (10 mL) and then the aqueous layers where extracted with CH_2Cl_2 (2 x10 mL). The organic layer was dried with $MgSO_4$. The solvents were removed in vacuo and the crude mixture was flushed through silica (4 cm x Ø 20 mm) with (33% EtOAc:hexane) to yield **68** as a pale brown oil (250 mg, 34% over 2 steps). This compound was unstable so limited characterisation was possible. ^{1}H NMR (500 MHz, $CDCl_3$) δ 9.61 (1H, s, H-3''), 4.31 (1H, dd, J=3.0, 11.5, Ha-1'), 4.17-4.10 (1H, m, Hb-1'), 4.04 (1H, ddd, J=3.0, 6.0, 9.0, H-3), 3.88 (2H, s, H-4'), 3.81 (1H, d, J=4.0, H-1), 2.14-2.08 (1H, m, H-7a), 1.96-1.89 (1H, m, H-3a), 1.75-1.58 (4H, m, 2 x CH_2), 1.46-1.25 (4H, m, 2x CH_2), 1.08 (3H, s, Me), 1.07 (3H, s, Me); ^{13}C NMR (125 MHz, $CDCl_3$) δ 205.7 (C3''), 166.7 (C3'), 88.3 (C1), 77.4 (C3), 66.5 (C1'), 49.3 (C2''), 38.6 (C7a or C3a), 38.4 (C7a or C3a), 28.7 (CH_2), 25.1 (C4'), 23.5 (CH_2), 23.4 (CH_2), 21.3 (CH_2), 18.3 (C1A''), 17.3 (C1B''); LC-MS (ESI$^+$) *m/z* 369 [M+Na$^+$], R_t 2.25 min [method D].

(1R,2R,7S,8S,13R)-13-hydroxy-14,14-dimethyl-10,15-dioxatricyclo[6.6.1.0$^{\{2,7\}}$]pentadecan-11-one 69

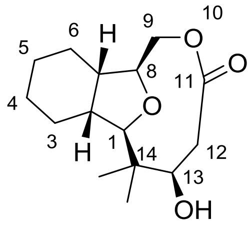

69

[(1S,3R,3aR,7aS)-3-(2-Methyl-1-oxopropan-2-yl)octahydro-2-benzofuran-1-yl]methyl 2-bromoacetate **68** (240 mg, 0.581 mmol) was dissolved in THF (200 mL), and SmI_2 (0.1 M in THF) was added dropwise (~25 mL) until the colour change of the SmI_2 from blue to yellow on addition slowed and the reaction mixture remained green. The solution was then diluted with Et_2O (20 mL), and washed with H_2O (20 mL) and brine (20 mL). The organic layer was dried with $MgSO_4$. The crude yellow oil was purified by column chromatography on silica gel (5% MeOH:CH_2Cl_2) to yield **69** as a white crystalline solid (70 mg, 45%). mp (107.8-108.3 ˚C); $[\alpha]^{21}_D$ +14.3 (c=2.0 $CHCl_3$); IR (film) 3518, 1724, 1448 cm^{-1}; ^{1}H NMR (500 MHz, $CDCl_3$) δ 5.41 (1H, dd, J=4.0, 11.0, H-9A), 3.93 (1H, dd, J=4.0, 8.0, H-8), 3.82-3.78 (1H, m, H-13), 3.74 (1H, d, J=11.0, H-9B), 3.62 (1H, d, J=1.5, H-1), 2.86-2.77 (2H, m, H-12), 2.49-2.43 (3H, m, H-2, H-7, OH), 1.72-1.60 (3H, m, CH_2), 1.57-1.49 (2H, m, CH_2), 1.38-1.24 (3H, m, CH_2), 1.08 (3H, s, CH_3), 1.01 (3H, s, CH_3); ^{13}C NMR (125 MHz, $CDCl_3$) δ 172.2 (C11), 94.7 (C1), 78.1 (C8), 75.4 (C13), 65.6 (C9), 41.9 (C14), 40.8 (C12), 38.8 (C2 or C7), 38.7 (C2 or C7), 30.1 (CH_2), 26.2 (Me), 23.6 (CH_2), 23.4 (CH_2), 23.1 (Me), 21.2 (CH_2); LC-MS (ESI$^+$) *m/z* 269 [M+H$^+$], R_t 1.28 min [method E]; HRMS [M+Na$^+$] calcd for $C_{15}H_{24}O_4Na$ 291.1566 found 291.1572; Found: C, 67.26; H, 9.01%; $C_{15}H_{24}O_4$ requires: C, 67.14; H, 9.01%.

(1R,2R,7S,8S,13R)-14,14-dimethyl-11-oxo-10,15-dioxatricyclo[6.6.1.0{2,7}]pentadecan-13-yl acetate 70

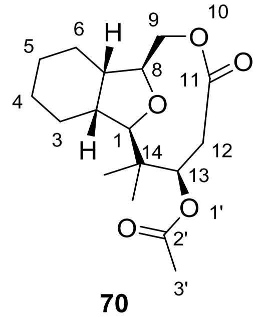

70

(1R,2R,7S,8S,13R)-13-Hydroxy-14,14-dimethyl-10,15-dioxatricyclo[6.6.1.0{2,7}]pentadecan-11-one **69** (8 mg, 0.030 mmol) was dissolved in CH_2Cl_2 (1.0 mL) and Et_3N (8.3 µL, 0.060 mmol) was added. Acetic anhydride (6.6 µL, 0.070 mmol) was added and the reaction mixture was stirred for 18 h. The solution was diluted with CH_2Cl_2 (5 mL) and washed with H_2O (10 mL x 2). The combined aqueous layers were extracted in CH_2Cl_2 (10 mL) and the combined organic layers dried over $MgSO_4$. Solvent was removed *in vacuo* to yield **70** as a clear oil (9 mg, quant.). $[\alpha]^{24}_D$ +42.3 (c=0.1 $CHCl_3$); IR (film) 2921, 2852, 1735, 1450 cm^{-1}; ^1H NMR (500 MHz, $CDCl_3$) δ 5.18 (1H, dd, J=4.0, 11.0, Ha-9), 4.95 (1H, dd, J=4.5, 4.5, H-13), 3.96 (1H, dd, J=4.0, 9.0, H-8), 3.74 (1H, d, J=11.5, Hb-9), 3.58 (1H, d, J=1.5, H-1), 2.83 (1H, obs dd, J=4.5, 15.0, Ha-12), 2.82-2.76 (1H, m, H-7), 2.68 (1H, dd, J=4.5, 15.0, Hb-12), 2.39-2.32 (1H, m, H-2), 2.11 (3H, s, H-3'), 1.72-1.50 (5H, m, CH_2), 1.37-1.21 (3H, m, CH_2), 1.07 (3H, s, Me), 0.98 (3H, s, Me); ^{13}C NMR (125 MHz, $CDCl_3$) δ 172.4 (C11), 169.9 (C2'), 95.2 (C1), 78.9 (C8), 76.1 (C13), 65.9 (C9), 41.6 (C14), 39.8 (C2), 38.7 (C12), 37.4 (C7), 30.9 (CH_2), 25.9 (Me), 24.4 (CH_2), 24.4 (Me), 23.7 (CH_2), 21.7 (CH_2), 21.0 (C3'); LC-MS (ESI^+) *m/z* 311 [$M+Na^+$], R_t 1.57 min [method D]; HRMS [$M+Na^+$] calcd for $C_{17}H_{26}O_5Na$ 333.1673 found 333.1691.

(1S,3S,3aR,7aS)-3-(Prop-2-en-1-yl)-octahydro-2-benzofuran-1-carbaldehyde 71

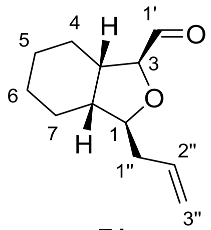

71

[(1S,3S,3aR,7aS)-3-(Prop-2-en-1-yl)-octahydro-2-benzofuran-1-yl]methanol **25** (146 mg, 0.753 mmol) was dissolved in CH_2Cl_2 (3 mL). Dess Martin reagent (3.0 mL, 0.3 M in CH_2Cl_2, 0.90 mmol) was added and the reaction was stirred at RT for 3 h. An aqueous quenching solution (10 mL, 1.0 g $NaHCO_3$ and 13 g $Na_2S_2O_3$ in 100 mL H_2O) was added and the mixture was stirred for 3 h. The organic layer was separated and washed with the quenching solution (5 mL), dried over $MgSO_4$ and flushed through silica gel (CH_2Cl_2). The solvent was removed *in vacuo* to yield **71** as a clear oil (146 mg, 100%). This compound was unstable so limited characterisation was possible and the compound was used immediately. ^1H NMR (500 MHz, $CDCl_3$) δ 9.69 (1H, d, J=2.0, H-1'), 5.90 (1H, dddd, J=7.0, 7.0, 10.0, 17.0, H-2''), 5.20-5.06 (2H, m, H-3''), 4.09-3.99 (2H, m, H-1, H-3), 2.45-2.25 (3H, m, H-1'', H-7a or H-3a), 1.98-1.90 (1H, m, H-7a or H-3a), 1.81-1.32 (8H, m, CH_2); ^{13}C NMR (125 MHz, $CDCl_3$) δ 203.2 (C1'), 134.2 (C2''), 116.8 (C3''), 85.7 (C1 or C3), 82.8 (C1 or C3), 41.6 (C3a or C7a), 39.7 (C3a or C7a), 39.2 (C1''), 25.2 (CH_2), 24.9 (CH_2), 22.5 (CH_2), 22.2 (CH_2).

(R)-1-((1S,3S,3aR,7aS)-3-allyl-1-methyloctahydroisobenzofuran-1-yl)pent-4-en-1-ol 72 and (S)-1-((1S,3S,3aR,7aS)-3-allyl-1-methyloctahydroisobenzofuran-1-yl)pent-4-en-1-ol 73

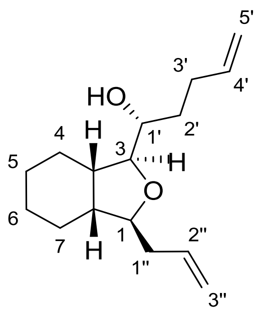

72

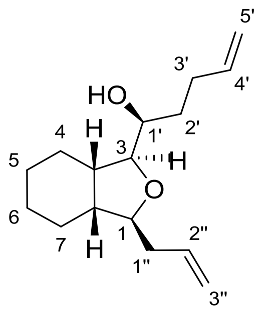

73

A solution of (1S,3S,3aR,7aS)-3-(Prop-2-en-1-yl)-octahydro-2-benzofuran-1-carbaldehyde **71** (160 mg, 0.824 mmol) in THF (8.2 mL) was added dropwise by syringe to a freshly prepared solution of but-3-enylmagnesium bromide (0.9 mL, 4.0 M in Et_2O) at 0 °C under nitrogen. The reaction was warmed to RT, quenched with sat. aqueous NH_4Cl (30 mL) and extracted with Et_2O (3 × 20 mL). The combined organic layers were dried over $NaSO_4$ and the solvents were removed *in vacuo*. The crude residue was purified by column chromatography (20% ethyl acetate in hexane) to give major isomer **72** (106 mg, 51%) and minor isomer **73** (59 mg, 29%) as clear oils. Major isomer $[\alpha]^{24.6}_D$ -35.1 (c=0.1 $CHCl_3$); IR (film) 3408, 2925, 2856, 1640 cm^{-1}; ^1H NMR (500 MHz, $CDCl_3$) δ 5.94-5.77 (2H, m, H-2'', H-4'), 5.22-4.96 (4H, m, H-3'', H-5'), 3.88 (1H, ddd, J=4.5, 7.0, 7.0, H-1), 3.70-3.62 (2H, m, H-1', H-3), 2.40-2.10 (5H, m, H-3', H-1'', H-3a), 2.00-1.88 (2H, m, H-7a, OH), 1.69-1.27 (10H, m, CH_2); ^{13}C NMR (125 MHz, $CDCl_3$) δ 138.6 (C4' or C2''), 135.2 (C4' or C2''), 117.3 (C3'' or C5'), 115.0 (C3'' or C5'), 86.3 (C3), 80.6 (C1), 72.6 (C1'), 42.5 (C7a), 39.1 (C1'' or C3'), 37.7 (C3a), 31.9 (CH_2), 30.5 (C1'' or C3'), 28.0 (CH_2), 25.3 (CH_2), 23.8 (CH_2), 22.8 (CH_2); LC-MS (ESI^+) *m/z* 273 [$M+Na^+$], R_t 1.45 min [method D]; HRMS [$M+H^+$] calcd for $C_{16}H_{27}O_2$ 251.2006 found 251.2013. Minor Isomer- ^1H NMR (500 MHz, $CDCl_3$) δ 5.90-5.77 (m, 2H, H-2'', H-4'), 5.14-4.93 (m, 4H, H-3'', H-5'), 3.89 (ddd, J = 7.0, 6.0, 5.2 Hz, 1H, H-1), 3.56 (t, J = 5.4 Hz, 1H, H-3), 3.35 (dq, J = 8.1, 5.2 Hz, 1H, H-1'), 2.38-2.05 (m, 6H, H-1'', H-3', H-3a, OH), 1.98-1.89 (m, 1H, H-7a), 1.69-1.30 (m, 10H); ^{13}C NMR (126 MHz, $CDCl_3$) δ 138.7 (C4' or C2''), 135.2 (C4' or C2''), 117.3 (C5' or C3''), 114.8 (C5' or C3''), 85.4 (C3), 81.5 (C1), 71.8 (C1'), 42.2 (C7a), 40.0 (C1'' or C3'), 39.0 (C3a), 34.0 (CH_2), 30.2 (C1'' or C3'), 26.5 (CH_2), 25.9 (CH_2), 23.4 (CH_2), 23.2 (CH_2); LC-MS (ESI^+) *m/z* 273 [$M+Na^+$], R_t 1.53 min [method D]; HRMS [$M+H^+$] calcd for $C_{16}H_{27}O_2$ 251.2006 found 251.2003.

(((R)-1-((1S,3S,3aR,7aS)-3-allyl-1-methyloctahydroisobenzofuran-1-yl)pent-4-en-1-yl)oxy)(tert-butyl)dimethylsilane 74

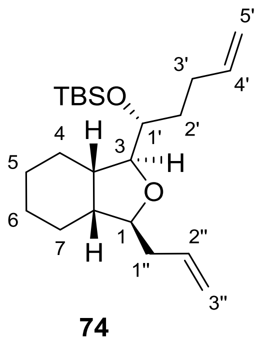

74

1-[(1S,3S,3aR,7aS)-3-(Prop-2-en-1-yl)-octahydro-2-benzofuran-1-yl]pent-4-en-1-ol **72** (70 mg, 0.283 mmol) was dissolved in CH_2Cl_2 (2.8 mL) and DIPEA (195 µL, 1.12 mmol) was added. TBSOTf (117 µL, 0.67 mmol) was added and the reaction was stirred at RT for 30 min. The reaction was quenched with sat. aqueous NH_4Cl (10 mL) and extracted in CH_2Cl_2 (3 × 10 mL). The combined organic layers were dried over

$MgSO_4$ and the solvent was removed *in vacuo* to yield **74** as a clear oil (102 mg, 100%). $[\alpha]^{24.3}{}_D$ -33.5 (c=0.25 $CHCl_3$); IR (film) 3407, 2927, 2857, 1463 cm^{-1}; ^1H NMR (500 MHz, $CDCl_3$) *δ* 5.95-5.81 (2H, m, H-2'', H-4'), 5.14-4.92 (4H, m, H-5', H-3''), 3.87-3.85 (1H, m, H-1), 3.58-3.52 (2H, m, H-3, H-1'), 2.37-2.24 (1H, m, Ha-1''), 2.20-2.12 (2H, m, Hb-1'', H-3a or H-7a), 1.94-1.84 (1H, m, H-3a or H-7a), 1.74-1.26 (12H, m, CH_2), 0.91 (9H, s, ^tBu), 0.08 (6H, s, Si-CH_3); ^{13}C NMR (125 MHz, $CDCl_3$) *δ* 139.2 (C2'' or C4'), 135.7 (C2'' or C4'), 116.5 (C3'' or C5'), 114.1 (C3'' or C5'), 85.8 (C3), 80.2 (C1), 73.6 (C1'), 41.6 (C3a or C7a), 39.7 (C3a or C7a), 39.6 (C1''), 33.4 (CH_2), 28.6 (CH_2), 28.2 (CH_2), 26.0 (tBu), 24.7 (CH_2), 24.3 (CH_2), 22.3 (CH_2), 18.1 (Si-C), -4.0 (Si-Me), -4.3 (Si-Me); HRMS [M+H$^+$] calcd for $C_{22}H_{41}O_2Si$ 365.2870 found 365.2875.

tert-butyl(((4aS,5S,6R,12S,12aR,Z)-5,12-dimethyl-1,2,3,4,4a,5,6,7,8,11,12,12a-dodecahydro-5,12-epoxybenzo[10]annulen-6-yl)oxy)dimethylsilane 75

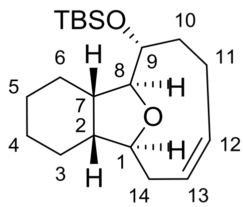

75

({1-[(1S,3S,3aR,7aS)-3-(Prop-2-en-1-yl)-octahydro-2-benzofuran-1-yl]pent-4-en-1-yl}oxy)(*tert*-butyl)dimethylsilane **74** (42 mg, 0.115 mmol) was dissolved in CH_2Cl_2 (230 mL) and Grubbs II catalyst (9.7 mg, 0.01 mmol) was added. The solution was stirred at RT for 16 h. DMSO (10 µL) was added and the solution was stirred for 24 h, before pre-absorbing on to silica (~200 mg). The product was purified by column chromatography to yield **75** as a clear oil (36 mg, 97%). $[\alpha]^{24.6}{}_D$ -31.1 (c=0.75 $CHCl_3$); IR (film) 2930, 2857 cm^{-1}; ^1H NMR (Acetone-*d*$_6$, 500 MHz) δ 5.75 (1H, tdd, *J*=11.6, 5.6, 2.0 Hz, H-12), 5.62 (1H, ddd, *J*=11.0, 8.7, 6.3 Hz, H-13), 4.09 (1H, dt, *J*=9.9, 3.1 Hz, H-1), 3.45 (1H, ddd, *J*=9.6, 8.8, 4.9 Hz, H-9), 3.28 (1H, d, *J*=8.8 Hz, H-8), 3.01 (1H, qd, *J*=11.7, 8.7 Hz, H-11a), 2.56 (1H, ddt, *J*=14.6, 6.3, 3.1, 2.0 Hz, H-14a), 2.18 (1H, dtd, *J*=10.3, 6.0, 4.8, 1.7 Hz, H-2), 2.07 (2H, ddd, *J*=14.8, 8.7, 2.7 Hz, H-14b), 1.96 (1H, dt, *J*=11.6, 5.9 Hz, H-7), 1.92 (1H, ddt, *J*=11.6, 4.8, 2.5, 1.2 Hz, H-11b), 1.73 – 1.66 (3H, m, H-5a, H-10), 1.66 – 1.54 (3H, m, H-6a, H-3), 1.54 – 1.48 (1H, m, H-4a), 1.40 – 1.29 (1H, m, H-4b), 1.29 – 1.14 (2H, m, H-5b, H-6b), 0.89 (9H, s, ^tBu), 0.08 (3H, s, Si-Me), 0.06 (3H, s, Si-Me); ^{13}C NMR (126 MHz, Acetone) δ 133.2 (C12), 125.5 (C13), 89.9 (C9), 79.5 (C1), 72.1 (C8), 42.8 (C7), 36.4 (C2), 33.6 (C10), 28.5 (C14), 27.9 (C6), 25.5 (C11), 25.3 (^tBu), 24.9 (C5), 23.5 (C3), 21.1 (4), 17.6 (^tBu), -4.4 (Si-Me), -5.2 (Si-Me); LC-MS (ESI$^+$) *m/z* 359 [M+Na$^+$], R_t 2.00 min [method E]; HRMS [M+H$^+$] calcd for $C_{20}H_{37}O_2Si$ 337.2557 found 337.2549

(4aS,5S,6R,12S,12aR,Z)-5,12-dimethyl-1,2,3,4,4a,5,6,7,8,11,12,12a-dodecahydro-5,12-epoxybenzo[10]annulen-6-ol 76

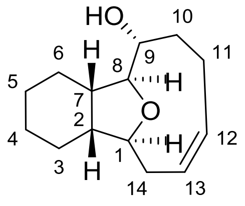

76

tert-Butyldimethyl[(1S,2R,7S,8S,12Z)-15-oxatricyclo[6.6.1.0$^{\{2,7\}}$]pentadec-12-en-9- yloxy]silane **75** (42 mg, 0.113 mmol), was dissolved in 1M HCl:EtOH:Et_2O (5 mL, 0.05:1:1) and stirred

at RT for 3 h. The solution was then basified with sat. aqueous $NaHCO_3$ (dropwise to neutral pH) and extracted into Et_2O (3 × 10 mL). The combined organic layers were washed in brine (10 mL) and dried over $MgSO_4$. The solvents were removed *in vacuo* to yield **76** as white crystals (14 mg, 55%). mp (102.3-103.2 ˚C); $[\alpha]^{22}_D$ +23.0 (c=0.1 $CHCl_3$); IR (film) 3014, 2923, 2851, 1449 cm^{-1}; ^1H NMR (500 MHz, $CDCl_3$) δ 5.73 (1H, dddd, J=2.5, 5.5, 11.0, 12.0, H-12), 5.59 (1H, ddd, J=6.5, 8.5, 11.0, H-13), 4.12 (1H, ddd, H-1), 3.40-3.35 (2H, m, H-8, H-9), 2.98 (1H, dddd, J=7.5, 12.0, 12.0, 12.0, Ha-11), 2.67-2.62 (1H, m, Ha-14), 2.20-2.16 (1H, m, H-2), 2.05-1.97 (3H, m, H-7, Hb-11, Hb-14), 1.82-1.75 (2H, m, H-10), 1.74-1.66 (2H, m, CH_2), 1.62-1.49 (3H, m, CH_2), 1.36-1.15 (3H, m, CH_2); ^{13}C NMR (125 MHz, $CDCl_3$) δ 133.6 (C12), 125.5 (C13), 89.7 (C8), 80.0 (C1), 71.6 (C9), 42.9 (C7), 36.7 (C2), 33.9 (C10), 28.8 (C14), 27.8 (CH_2), 25.4 (C11), 24.9 (CH_2), 23.9 (C3), 21.4 (CH_2); LC-MS (ESI^+) *m/z* 245 [$M+Na^+$], R_t 1.47 min [method E]; HRMS [$M+H^+$] calcd for $C_{14}H_{23}O_2$ 223.1693 found 223.1695.

((1R,2S,3S,5S,6R,7S)-5-Allyl-4-oxatricyclo[5.2.1.0^{2,6}]dec-8-en-3-yl)-methanol 79

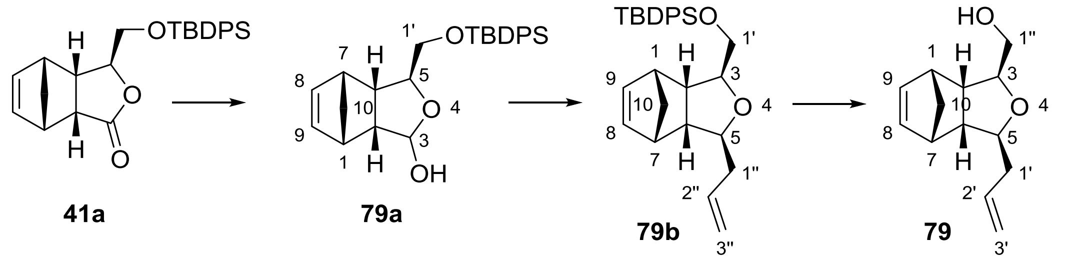

(1*S*,2*R*,5*S*,6*S*,7*R*)-5-(*tert*-Butyldiphenylsilanoxymethyl)-4-oxatricyclo[5.2.1.0.^{2,6}]dec-8-en-3-one **41a** (292 mg, 0.699 mmol) was dissolved in anhydrous CH_2Cl_2 (1.9 mL) and cooled to -78 °C under N_2. DIBAL-H (1.0 M in toluene, 0.77 mL, 0.77 mmol) was added dropwise. The reaction was stirred at 78 °C for 100 min then quenched with EtOAc (10 mL). The solution was warmed to RT and a sat. aq. solution of Rochelles salt (6 mL) was added and the mixture was stirred for 3 h. The aqueous layer was separated and extracted with CH_2Cl_2 (3 x 20 mL). The organic layers were combined, dried over $MgSO_4$ and the solvent was removed *in vacuo* to yield **79a** as a clear oil (286 mg, 98%). $[\alpha]^{24}_D$ +2.8 (c=0.34 CH_2Cl_2); IR (film) 3423, 2931, 2858, 1428 cm^{-1}; ^1H NMR (500 MHz, $CDCl_3$) δ 7.73-7.67 (4H, m, Ph), 7.49-7.41 (6H, m, Ph), 6.19-6.17 (2H, m, H-8, H-9), 4.87 (1H, d, *J*=9.5, H-3), 3.99 (1H, d, *J*=9.5, OH), 3.76 (1H, dd, *J*=3.0, 11.0, Ha-1'), 3.67-3.65 (1H, m, H-5), 3.55 (1H, dd, *J*=3.0, 11.0, Hb-1'), 3.08-3.05 (1H, m, H-7), 2.92-2.89 (1H, m, H-1), 2.88-2.84 (2H, m, H-2, 6), 1.48 (1H, d, *J*=8.0, Ha-10), 1.35 (1H, d, *J*=8.0, Hb-10), 1.09 (9H, s, ^tBu); ^{13}C NMR (125 MHz, $CDCl_3$) δ 135.8 (2 x Ph), 135.7 (2 x Ph), 135.6 (Ph), 134.9 (C9), 134.6 (C8), 132.5 (Ph), 132.4 (Ph), 130.1 (Ph), 129.9 (Ph), 129.4 (Ph), 127.9 (Ph), 127.7 (Ph), 101.3 (C3), 83.6 (C5), 67.6 (C1'), 58.1 (C1), 51.6 (C10), 48.3 (C6), 46.0 (C2), 45.2 (C7), 27.0 (^tBu), 19.2 (Si-C); LC-MS (ESI^+) m/z 443 [$M+Na^+$], Rt 3.22 min, [method C]; HRMS [$M+Na^+$] calcd for $C_{26}H_{32}O_3SiNa$ 443.2013; found 443.2002.

(1*S*,2*R*,5*S*,6*S*,7*R*)-5-(*tert*-Butyldiphenylsilanoxymethyl)-4-oxatricyclo[5.2.1.0.^{2,6}]dec-8-en-3-ol **79a** (286 mg, 0.90 mmol) was dissolved in CH_2Cl_2 (3 mL) and cooled to -78 °C. $BF_3.OEt_2$ (0.26 mL, 2.04 mmol) was added by syringe and the solution stirred for 5 min. Allyltrimethylsilane (0.32 mL, 2.04 mmol) was added by syringe and the reaction then warmed to RT over 18 h. The reaction was then quenched with H_2O (6 mL) and extracted into CH_2Cl_2 (3 x 6 mL). The combined organic layers were dried over $MgSO_4$. The solvent was removed *in vacuo* to yield **79b** as a clear oil (289 mg, 96%). $[\alpha]^{22}_D$ 67.7 (c=0.10 CH_2Cl_2); IR (film) 2930, 2857, 1471, 1427 cm^{-1}; ^1H NMR (500 MHz, $CDCl_3$) δ 7.72-7.68 (4H, m, Ph), 7.45-7.39 (6H, m, Ph), 6.22-6.19 (2H, m, H-8, H-9), 5.85 (1H, dddd, *J*=7.0, 7.0, 10.0, 17.0, H-2'), 5.09-5.02 (2 H, m, H-3'), 3.72 (1H, dd, *J*=5.0, 10.0 H-1''), 3.59 (1H, dd, *J*=6.0, 10.0, H-1''), 3.54 (1H, ddd, *J*=6.0, 6.0, 6.0, H-3), 3.48 (1H, ddd, *J*=6.5, 6.0, 6.5, H-5), 2.89 (1H, ddd, *J*=4.0, 6.0, 10.0, H-2), 2.85-2.82 (1H, m, H-1), 2.80-2.78 (1H, m, H-7), 2.66 (1H, ddd, *J*=4.0, 6.0, 10.5, H-6), 2.38-2.32 (1H, m, H-1'), 2.20-2.15 (1H, m, H-1'), 1.71 (1H, dt, *J*=2.0, 8.0, H-10), 1.59-1.56 (1H, m, H-10), 1.09 (9H, s, ^{t}Bu); ^{13}C NMR (125 MHz, $CDCl_3$) δ 136.7 (C9), 136.6 (C8), 135.7 (4 x Ph), 135.1 (C1'), 133.8 (^{i}Ph), 133.7 (^{i}Ph), 129.6 (Ph), 129.6 (Ph), 127.6 (4 x Ph), 116.6 (C3'), 82.1 (C3), 81.5 (C5), 66.9 (C1''), 55.0 (C2), 53.8 (C10), 53.1 (C6), 45.0 (C1), 44.9 (C7), 41.0 (C1'), 26.9 (^{t}Bu), 19.3 (Si-C).

((1*R*,2*S*,3*S*,5*S*,6*R*,7*S*)-5-Allyl-4-oxatricyclo[5.2.1.0^{2,6}]dec-8-en-3-ylmethoxy)-*tert*-butyldiphenyl silane **79b** (178 mg, 0.403 mmol) was dissolved in THF (2.2 mL). The solution was cooled to 0 °C under N_2 and TBAF (1.20 mL, 1.20 mmol, 1.0 M in THF) was added by syringe. The reaction was stirred for 1 h at 0 °C before quenching with Et_2O (20 mL). The reaction mixture was washed with H_2O (3 x 30 mL) and extracted into Et_2O (2 x 30 mL). The combined organic layers were dried over Na_2SO_4 and the solvent was removed *in vacuo* to give the crude product. This was purified by column chromatography on silica gel (15% EtOAc:hexane) to yield **79** as a clear oil (49 mg, 59%). $[\alpha]^{25}_D$ -5.7 (c=0.51 CH_2Cl_2); IR (film) 3420, 2965, 1653, 1558 cm^{-1}; ^1H NMR (500 MHz, d5-Pyr) δ 6.23-6.11 (2H, m, H-8, H-9), 6.11-6.02 (1H, m, OH), 5.91 (1H, ddt, *J*=7.0, 10.5, 17.0, H-2'), 5.12 (1H, dd, *J*=1.5, 17.0, Ha-3'), 5.09-5.09 (1H, m, Hb-3'), 3.90 (1H, dd, J=5.0, 11.0, Ha-1''), 3.84 (1H, dd, J=6.0, 11.0, Hb-1''), 3.75 (1H, ddd, *J*=5.5, 6.0, 6.0, H-3), 3.57 (1H, ddd, *J*=6.5, 6.5, 6.5, H-5), 2.93 (1H, ddd, *J*=4.0, 6.5, 10.5, H-2), 2.80-2.75 (1H, m, H-1) 2.70-2.64 (1H, m, H-7), 2.64 (1H, ddd, *J*=4.0, 6.5, 10.0, H-6), 2.43 (1H, m, H-1'), 2.29 (1H, ddd, *J*=7.0, 7.0, 13.5, H-1'), 1.63 (1H, d, *J*=8.0, Ha-10), 1.48 (1H, d, *J*=8.0, Hb-10); ^{13}C NMR (125 MHz, $CDCl_3$) δ 137.6 (C8 or C9), 137.4 (C8 or C9), 136.4 (C2'), 117.1 (C3'), 83.9 (C3), 81.9 (C5), 66.1 (C1''), 56.0 (C2), 54.7 (C10), 53.5 (C6), 45.6 (C1 or C7), 45.5 (C1 or C7), 41.7 (C2'); LC-MS (ESI$^+$) *m/z* 229 [M+Na$^+$], R_t 2.12 min [method D]; HRMS [M+H$^+$] calcd for $C_{13}H_{19}O_2$ 207.1380; found 207.1377.

(1R,2S,3S,5S,6R,7S)-5-allyl-4-oxa-tricyclo[5.2.1.0^{2,6}]dec-8-en-3-ylmethyl but-3-enoate 80

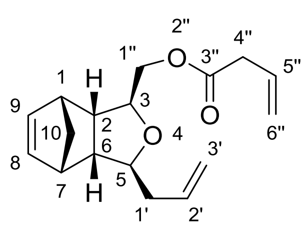

80

((1R,2S,3S,5S,6R,7S)-5-Allyl-4-oxatricyclo[5.2.1.0^{2,6}]dec-8-en-3-yl)methanol **79** (50 mg, 0.243 mmol) was dissolved in CH_2Cl_2 (0.5

mL). *Trans*-crotonyl chloride (30 µL, 0.316 mmol) and triethylamine (105 µL, 0.725 mmol) were added and the reaction mixture was stirred at RT for 18 h. The reaction mixture was diluted with CH_2Cl_2 (10 mL) and washed with H_2O (10 mL) and brine (10 mL). The aqueous layers were combined and extracted with CH_2Cl_2 (10 mL x 2). The combined organic layers were dried with $MgSO_4$ and the solvents removed *in vacuo*. The crude reaction mixture was purified by column chromatography on silica gel (20% EtOAc:hexane) to yield **80** as a clear oil (44 mg, 66%). $[\alpha]^{25}_{D}$ -19.0 (c=1.0 $CHCl_3$); IR (film) 3076, 2968, 1752 cm^{-1}; ^1H NMR (500 MHz, $CDCl_3$) δ 6.23-6.19 (2H, m, H-9, H-8), 5.93 (1H, tdd, *J*=6.5, 10.0, 17.0, H-5''), 5.78 (1H, tdd, *J*= 7.0, 10.0, 17.0, H-2'), 5.19-5.14 (2H, m, H-6''), 5.11-5.03 (2H, m, H-3'), 4.15 (1H, dd, *J*=4.0, 11.0, HA-1''), 4.02 (1H, dd, *J*=7.0, 11.0, HB-1''), 3.64 (1H, dd, *J*=6.0, 11.0, H-3), 3.51 (1H, dd, *J*=6.0, 12.5, H-5), 3.13 (2H, d, *J*=7.0, H-4''), 2.84-2.77 (2H, m, H-1, H-7), 2.76-2.67 (2H, m, H-2, H-6), 2.41-2.34 (1H, m, HA-1'), 2.24-2.18 (1H, m, HB-1'), 1.71 (1H, d, *J*=8.0, HA-10), 1.56 (1H, d, *J*=8.0, HB-10); ^{13}C NMR (125 MHz, $CDCl_3$) δ 171.4 (C3''), 136.8 (C8 or C9), 136.3 (C8 or C9), 134.7 (C2'), 130.2 (C5''), 118.6 (C6''), 116.9 (C3'), 81.8 (C5), 79.2 (C3), 67.1 (C1''), 54.9 (C2 or C6), 53.7 (C10), 52.9 (C2 or C6), 44.9 (C1 or C7), 44.5 (C1 or C7), 40.8 (C1'), 38.7 (C4''); LC-MS (ESI^+) *m/z* 297 $[M+Na^+]$, R_t 2.37 min [method D]; HRMS $[M+Na^+]$ calcd for $C_{17}H_{22}O_3$ H 275.1643 found 275.1642.

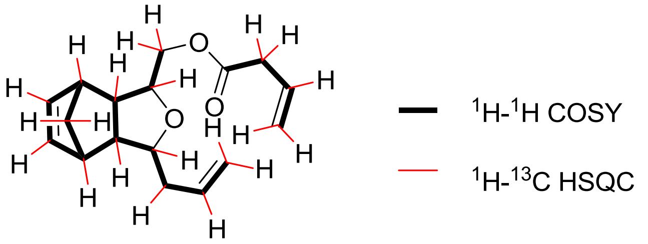

(1R,2S,3S,5S,6R,7S)-5-Allyl-4-oxatricyclo[5.2.1.0^{2,6}]dec-8-en-3-ylmethyl pent-4-enoate 81

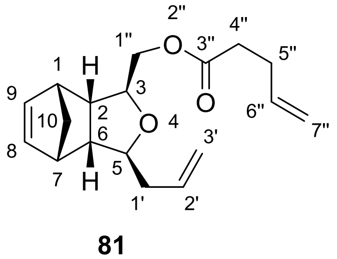

81

((1R,2S,3S,5S,6R,7S)-5-Allyl-4-oxatricyclo[5.2.1.0^{2,6}]dec-8-en-3-yl)-methanol **79** (50 mg, 0.243 mmol) was dissolved in pyridine (1.2 mL). Pent-4-enoyl chloride (35 µL, 0.316 mmol) was added and the reaction mixture was stirred at RT for 18 h. The reaction mixture was diluted with CH_2Cl_2 (10 mL) and washed with H_2O (10 mL) and brine (10 mL). The aqueous layers were combined and extracted with CH_2Cl_2 (10 mL x 2). The combined organic layers were dried with $MgSO_4$ and the solvents removed *in vacuo*. The crude reaction mixture was purified by column chromatography on silica gel (20% EtOAc:hexane) to yield **81** as a clear oil (49 mg, 70%). $[\alpha]^{25}_{D}$ +15.5 (c=2.0, $CHCl_3$); IR (film) 3075, 2965, 1741 cm^{-1}; ^1H NMR (500 MHz, $CDCl_3$) δ 6.22-6.21 (2H, m, H-9, H-8), 5.88-5.74 (2H, m, H-2', H-6''), 5.12-4.99 (4H, m, H-3', H-7''), 4.15 (1H, dd, *J*=4.5, 11.5, HA-1''), 4.02 (1H, dd, *J*=6.5, 11.5, HB-1''), 3.64 (1H, ddd, *J*=6.5, 6.5, 4.5, H-3), 3.15 (1H, ddd, *J*=5.5, 5.5, 7.5, H-5), 2.83-2.79 (2H, m, H-1, H-7), 2.76-2.69 (2H, m, H-2, H-6), 2.47-2.35 (5H, m, H-4'', H-5'', HA-1'), 2.20 (1H, ddd, *J*=7.5, 7.5,

14.5, HB-1'), 1.71 (1H, ddd, *J*=1.5, 1.5, 8.5, HA-10), 1.57 (1H, br d, *J*=8.5, HB-10); ^{13}C NMR (125 MHz, $CDCl_3$) δ 172.9 (C3''), 136.9 (C8 or C9), 136.7 (C2' or C6''), 136.4 (C8 or C9), 134.6 (C2' or C6''), 117.0 (C3' or C7''), 115.5 (C3' or C7''), 81.8 (C5), 79.3 (C3), 66.9 (C1''), 54.9 (C2 or C6), 53.9 (C10), 52.7 (C2 or C6), 44.9 (C1 or C7), 44.5 (C1 or C7), 40.8 (C1'), 33.5 (C4'' or C5''), 28.8 (C4'' or C5''); LC-MS (ESI+) m/z 311 [M+Na+], Rt 2.43 min [method D]; HRMS [M+H$^+$] calcd for $C_{18}H_{24}O_3$ H 289.1798 found 289.1798.

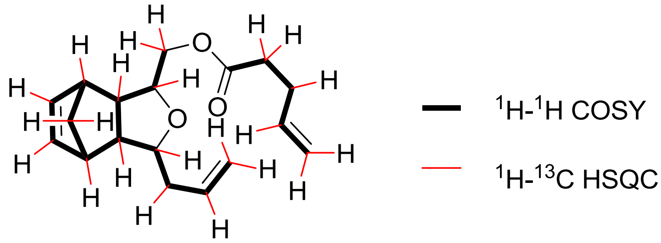

(1R,2S,3S,5S,6R,7S)-5-allyl-4-oxatricyclo[5.2.1.0^{2,6}]dec-8-en-3-ylmethyl hex-5-enoate 82

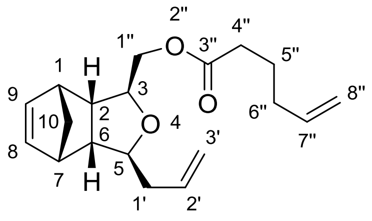

82

((1R,2S,3S,5S,6R,7S)-5-Allyl-4-oxatricyclo[5.2.1.0^{2,6}]dec-8-en-3-yl) methanol **79** (50 mg, 0.243 mmol) was dissolved in pyridine (1.2 mL). Hex-5-enoyl chloride (42 mg, 0.317 mmol) was added and the reaction mixture was stirred at RT for 18 h. The reaction mixture was diluted with CH_2Cl_2 (10 mL) and washed with H_2O (10 mL) and brine (10 mL). The aqueous layers were combined and extracted with CH_2Cl_2 (10 mL x 2). The combined organic layers were dried over $MgSO_4$ and the solvents removed *in vacuo*. The crude reaction mixture was purified by column chromatography on silica gel (20% EtOAc:hexane) to yield **82** as a clear oil (33 mg, 46%). $[\alpha]^{25}_D$ +19.6 (c=1.0 $CHCl_3$); IR (film) 3075, 2965, 1738 cm^{-1}; ^1H NMR (500 MHz, $CDCl_3$) *δ* 6.25-6.22 (2H, m, H-9, H-8), 5.83-5.74 (2H, m, H-2', H-7'', 5.13-5.05 (2H, m, H-3'), 5.06-4.98 (2H, m, H-8''), 4.18 (1H, dd, *J*=4.5, 11.0, Ha-1''), 4.02 (1H, dd, *J*=7.0, 11.0, Hb-1''), 3.63 (1H, ddd, *J*=4.5, 6.0, 6.5, H-3), 3.51 (1H, ddd, *J*=6.0, 7.0, 7.5, H-5), 2.84-2.79 (2H, m, H-1, H-7), 2.77-2.69 (2H, m, H-2, H-6), 2.42-2.35 (1H, m, Ha-1'), 2.38 (2H, t, J=7.5, H-4''), 2.26-2.19 (2H, m, H-1'), 2.14-2.08 (1H, m, H-5''), 1.78-112 (3H, m, H-6'', Ha-10), 1.61-1.56 (1H, m, Hb-10), ^{13}C NMR (125 MHz, $CDCl_3$) *δ* 173.5 (C3''), 137.6 (C7''), 136.9 (C8 or C9), 136.3 (C8 or C9), 134.6 (C2'), 116.2 (C3'), 115.3 (C8''), 81.7 (C5), 79.3 (C3), 66.8 (C1''), 54.9 (C2 or C6), 53.8 (C10), 52.7 (C2 or C6), 44.9 (C1 or C7), 44.5 (C1 or C7), 40.8 (C1'), 33.7 (C4''), 33.0 (C5''), 24.0 (C6''); LC-MS (ESI$^+$) *m/z* 325 [M+Na$^+$], R_t 2.48 min [method D]; HRMS [M+H$^+$] calcd for $C_{19}H_{27}O_3$ 303.1955 found 303.1940.

[(1S,3R,3aR,7aS)-3-(2-methyl-3-oxobutan-2-yl)-octahydro-2-benzofuran-1-yl]methyl acetate 89

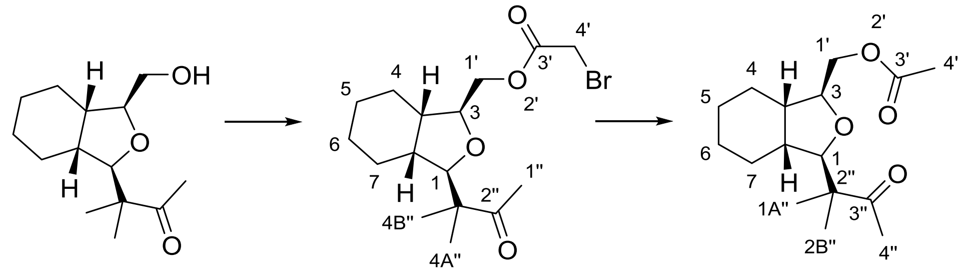

46 **89a** **89**

3-[(1R,3S,3aS,7aR)-3-(Hydroxymethyl)octahydro-2-benzofuran-1-yl]-3-methylbutan-2-one **46** (67 mg, 0.279) was dissolved in CH_2Cl_2 (3 mL) and Et_3N (80 µL, 0.558 mmol) was added. 2-Bromoacetyl bromide (260 µL, 0.558 mmol) was added and the solution was stirred at RT for 5 h. The solution was diluted with CH_2Cl_2 (20 mL), washed with H_2O (10 mL), brine (10 mL), and then the aqueous layers where extracted with CH_2Cl_2 (2 x10 mL). The combined organic layers were dried with $MgSO_4$. The solvent was removed *in vacuo* and the crude residue purified by column chromatography on silica gel (33% EtOAc:hexane) to yield the product as a pale brown oil (67 mg, 67%). This compound was unstable so limited characterisation was possible. ^{1}H NMR (500 MHz, $CDCl_3$) δ 4.29 (1H, dd, J=3.0, 12.0, Ha-1'), 4.16 (1H, dd, J=6.0, 12.0, Hb-1'), 4.03 (1H, ddd, J=3.0, 6.0, 9.0, H-3), 3.88 (2H, s, H-4'), 3.88-3.80 (1H, m, H-1), 2.18 (3H, s, H-1''), 2.07-2.01 (1H, m, H-7a), 1.95-1.88 (1H, m, H-3a), 1.75-1.68 (1H, m, CH_2), 1.68-1.57 (3H, m, CH_2), 1.45-1.23 (4H, m, CH_2), 1.13 (3H, s, Me), 1.09 (3H, s, Me); LC-MS (ESI^+) *m/z* 361 [$M+H^+$], R_t 1.50 min [method E]; HRMS [$M+Na^+$] calcd for $C_{16}H_{25}BrO_4Na$ 361.1009 found 361.1013.

[(1S,3R,3aR,7aS)-3-(2-Methyl-3-oxobutan-2-yl)-octahydro-2-benzofuran-1-yl]methyl 2-bromoacetate **89a** (67 mg, 0.186 mmol) was dissolved in THF (37 mL) and SmI_2 (0.1 M in THF) was added dropwise (~9 mL) until the colour change of the SmI_2 from blue to yellow on addition slowed and the reaction mixture remained green for ~10 seconds before returning to a pale yellow. The solution was then diluted with Et_2O (20 mL) and washed with H_2O (20 mL) and brine (20 mL). The organic layer was dried with $MgSO_4$. The crude yellow oil was purified by column chromatography on silica gel (5% MeOH:CH_2Cl_2) to yield **89** as a clear oil (26 mg, 49%). $[\alpha]^{24.3}_D$ +25.4 (c=1.0 $CHCl_3$); IR (film) 2929, 2858, 1739, 1701 cm^{-1}; ^{1}H NMR (500 MHz, $CDCl_3$) δ 4.25-4.19 (1H, m, Ha-1'), 4.06-3.97 (2H, m, Hb-1', H-3), 3.86 (1H, d, J=3.5, H-1), 2.17 (3H, s, H-4''), 2.08 (3H, s, H-4'), 2.06-1.98 (1H, m, H-7a), 1.83-1.76 (1H, m, H-3a), 1.75-1.57 (4H, m, CH_2), 1.46-1.22 (4H, m, CH_2), 1.12 (3H, s, Me), 1.09 (3H, s, Me), ^{13}C NMR (125 MHz, $CDCl_3$) δ 213.2 (C3''), 171.1 (C3'), 89.7 (C1), 78.0 (C3), 65.7 (C1'), 51.6 (C2''), 39.4 (C3a or C7a), 39.4 (C3a or C7a), 29.9 (CH_2), 27.0 (C4''), 24.2 (CH_2), 24.1 (CH_2), 22.1 (CH_2), 21.0 (C4'' or Me), 21.0 (C4'' or Me), 20.6 (C4'' or Me); LC-MS (ESI^+) *m/z* 305 [$M+Na^+$], R_t 1.48 min [method E]; HRMS [$M+H^+$] calcd for $C_{16}H_{27}O_4$ 283.1904 found 283.1914; Found: C, 66.09; H, 8.81%; $C_{16}H_{26}O_4$ requires: C, 66.12; H, 8.72%.

Assay methods

SRB assay

Cells were seeded in 100µl medium into 96-well plates. After 36 hours, serial doubling dilutions of drug (or vehicle) were added to give the required dose range in sextuplicate across the plate. Cells were incubated for a further 96 hours. The medium was then removed and cells were fixed by adding 50µl of 10% (w/v) trichloroacetic acid (TCA) for one hour at room temperature. The plates were gently washed by immersion in tap water three times then air dried. 50µl of 0.4% (w/v) SRB in 1% (v/v) acetic acid was added to each well for one hour. Plates were then washed three times with 1% acetic acid and air dried. SRB was solubilised with 100ul/well 10mM Tris

pH10.5. Absorbance at 570nm was measured using a SPECTRAmax 340PC plate reader.

Migration assay

Cells in standard tissue culture flasks were fluorescently labelled by incubation with 1umol/L CellTracker Green 5-chloromethylfluorescein diacetate (Invitrogen, Paisley) concomitant with serum starvation for two hours. Following trypsinisation cells were counted and added to the upper wells of 8um pore FluoroblokTM membrane inserts in 24-well companion plates (BD Biosciences, Franklin Lakes, NJ). The lower chambers were filled with 800uL medium containing 5% heat- inactivated FCS (to measure chemotaxis) or serum-free medium (to measure unstimulated random motility). The assay plates were incubated in a humidified atmosphere at 37°C in 5% CO_2 in air for 16 hours. Cells which successfully migrated to the lower surface of the filter were visualised using an inverted fluorescence microscope.

PCA methodology

Principal Components Analysis (PCA) was conducted using SIMCA-P+[4], and the scatter plot was created using Spotfire[5]. The descriptors used for the calculation of the PCA models are given in Table S1.

Table S1. Molecular descriptors calculated for the generation of the PCA model.

Descriptor ID	Descriptor Definition
Molecular Weight	Molecular weight
No. Bonds	Number of bonds
No. Rings	Number of rings
AlogP	Calculated octanol/water partition coefficient
No. Rotatable Bonds	Number of rotatable bonds
No. H acceptors	Number of hydrogen-bond acceptors
No. H donors	Number of hydrogen-bond donors
O count	Number of oxygen atoms
H count	Number of hydrogen atoms
No. Stereocentres	Number of stereocentres
N count	Number of nitrogen atoms
No. Aromatic Rings	Number of aromatic rings
No. Aromatic Bonds	Number of aromatic bonds

The first two principal components of the PCA model explain 69% of the variance in the data which indicates that visualisation in a two-dimensional scatterplot is reasonable for interpretation of the chemistry spaces covered by the libraries.

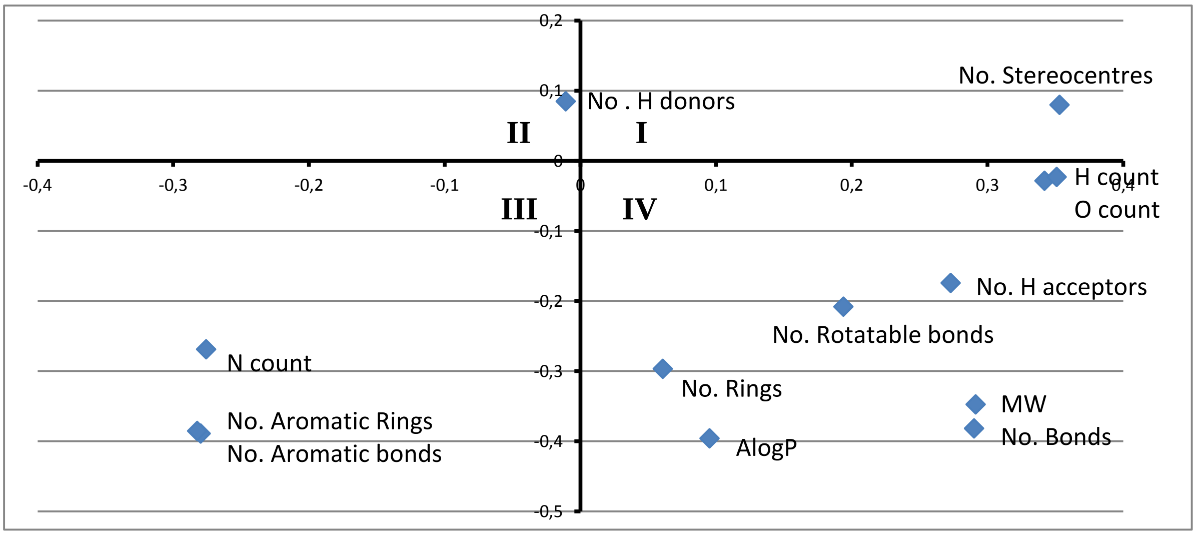

Figure S1. The loading scores of the Principal Components Analysis.

The loadings plot is provided in Figure S1 and can assist in interpreting the PCA scores plot of Figure 5 in the main paper. The loadings plot illustrates those molecular descriptors that are most important for explaining the variance of the molecules in those regions of the scores plot when overlaid. Descriptors that lie proximate to each other on the loadings plot are correlated, such as aromatic rings and bonds in quadrant III. Furthermore, the magnitude of deviation from the origin indicates the importance of each descriptor in that region of the plot, as seen in those molecules in quadrant III being more nitrogen-rich in their composition. Therefore, it can be readily observed that the screening collection is enriched for nitrogen-containing compounds and aromaticity, as opposed to the cembranoid-like and cembranoid libraries.

Crystallography data for compound 26

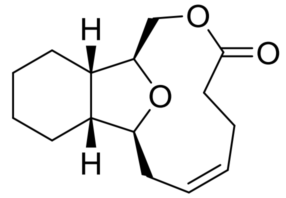

26

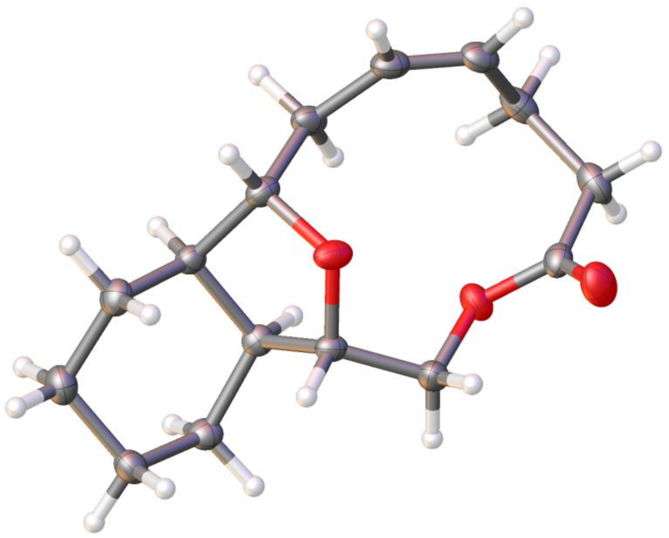

Formula: $C_{15}H_{22}O_3$; M_r = 250.32; crystal dimensions: 0.62 x 0.22 x 0.06 mm; crystal system: orthorhombic; space group: $P2_12_12_1$; a = 5.25570(10) Å, b = 10.3241(4) Å, c = 24.6007(9) Å, α = 90°, β = 90°, γ = 90°; V = 1334.84(8) Å^3; Z = 4; ρ_{calcd} = 1.246 Mg/m^3; μ = 0.085 mm^{-1}; Mo $K\alpha$ radiation, λ = 0.71073 Å; T = 120(2) K; $2\Theta_{max}$ = 54.9°; 10722/3007 measured/independent reflections; R_{int}: 0.0442; R = 0.0418, wR = 0.0935; $\Delta\rho_{max}$ = 0.206 eÅ^{-3}, $\Delta\rho_{min}$ = −0.208 eÅ^{-3}. Colourless blade crystals gave good diffraction. The data were collected on a Nonius-Kappa CCD area detector mounted at the window of an FR591 rotating anode generator with a Mo anode and equipped with an Oxford Cryosystems cryostream device. Nonius COLLECT[6] was used to record images and HKL (Denzo and Scalepack)[7] was used for data integration. The structure was solved by direct methods using SHELXT[8] and refined on F_o^2 by full-matrix least squares refinement using SHELXL.[9] All non-hydrogen atoms were refined with anisotropic displacement parameters. Hydrogen atoms were added at calculated positions and refined using a riding model with isotropic displacement parameters based on the equivalent isotropic displacement parameter (Ueq) of the parent atom. The structure was deposited on the Cambridge Structural Database with the deposition number CCDC 1010197.

Crystallography data for compound 40

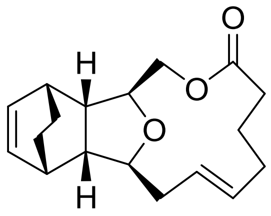

40

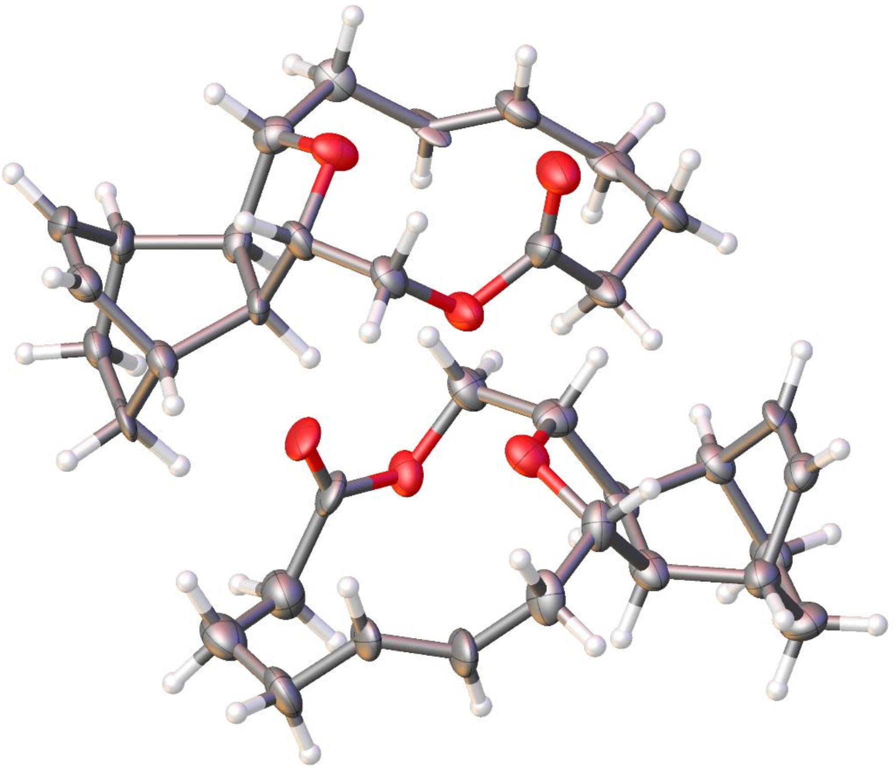

Formula: $C_{18}H_{24}O_3$; M_r = 288.37; crystal dimensions: 0.3 x 0.2 x 0.19 mm; crystal system: monoclinic; space group: $P2_1$; a = 10.801(3) Å, b = 11.564(3) Å, c = 12.083(4) Å, α = 90°, β = 90.15(3)°, γ = 90°; V = 1509.2(8) Å^3; Z = 4; ρ_{calcd} = 1.269 Mg/m^3; μ = 0.085 mm^{-1}; Mo $K\alpha$ radiation, λ = 0.71073 Å; T = 120(2) K; $2\Theta_{max}$ = 50.2°; 9752/9752 measured/independent reflections; R_{int}: N/A; R = 0.0899, wR = 0.2274; $\Delta\rho_{max}$ = 0.348 eÅ^{-3}, $\Delta\rho_{min}$ = −0.308 eÅ^{-3}. Colourless block crystals were poorly diffracting with no significant data beyond 0.84Å resolution and also non-merohedrally twinned, with a twinning ratio of 0.537:0.463. The twinned components were related by 179.8° rotation about reciprocal lattice vector 001. The data were collected on a Nonius-Kappa CCD area detector mounted at the window of an FR591 rotating anode generator with a Mo anode and equipped with an Oxford Cryosystems cryostream device. Nonius COLLECT[6] was used to record images and RigakuOD CrysAlisPro[10] was used for data integration. The structure was solved by direct methods using SHELXT[8] and refined on F_o^2 by full-matrix least squares refinement using SHELXL.[9] All non-hydrogen atoms were refined with anisotropic displacement parameters. Hydrogen atoms were added at calculated positions and refined using a riding model with isotropic displacement parameters based on the equivalent isotropic displacement parameter (Ueq) of the parent atom. The structure was deposited on the Cambridge Structural Database with the deposition number CCDC 1011320.

Crystallography data for compound 42

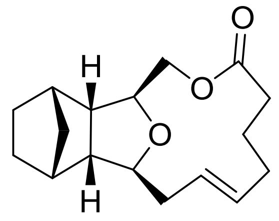

42

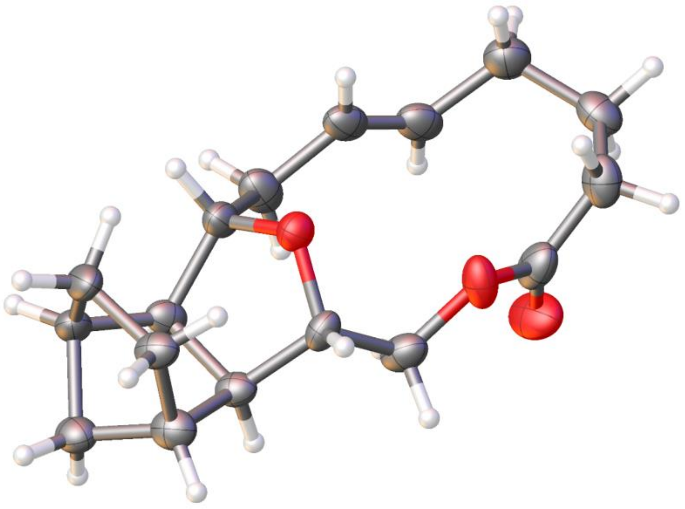

Formula: $C_{17}H_{24}O_3$; M_r = 276.36; crystal dimensions: 0.4 x 0.2 x 0.03 mm; crystal system: orthorhombic; space group: $P2_12_12_1$; a = 5.7992(6) Å, b = 12.0419(10) Å, c = 20.795(2) Å, α = 90°, β = 90°, γ = 90°; V = 1452.2(2) Å^3; Z = 4; ρ_{calcd} = 1.264 Mg/m^3; μ = 0.085 mm^{-1}; Mo $K\alpha$ radiation, λ = 0.71073 Å; T = 120(2) K; $2\Theta_{max}$ = 54.9°; 17321/3252 measured/independent reflections; R_{int}: 0.1130; R = 0.1002, wR = 0.2476; $\Delta\rho_{max}$ = 0.534 eÅ^{-3}, $\Delta\rho_{min}$ = −0.400 eÅ^{-3}. Colourless plate crystals were poorly diffracting. The data were collected on a Nonius-Kappa CCD area detector mounted at the window of an FR591 rotating anode generator with a Mo anode and equipped with an Oxford Cryosystems cryostream device. Nonius COLLECT[6] was used to record images and RigakuOD CrysAlisPro[10] was used for data integration. The structure was solved by direct methods using SHELXT[8] and refined on F_o^2 by full-matrix least squares refinement using SHELXL.[9] All non-hydrogen atoms were refined with anisotropic displacement parameters. Hydrogen atoms were added at calculated positions and refined using a riding model with isotropic displacement parameters based on the equivalent isotropic displacement parameter (Ueq) of the parent atom. The structure was deposited on the Cambridge Structural Database with the deposition number CCDC 1435146.

Crystallography data for compound 69

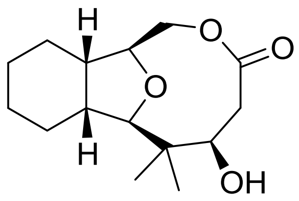

69

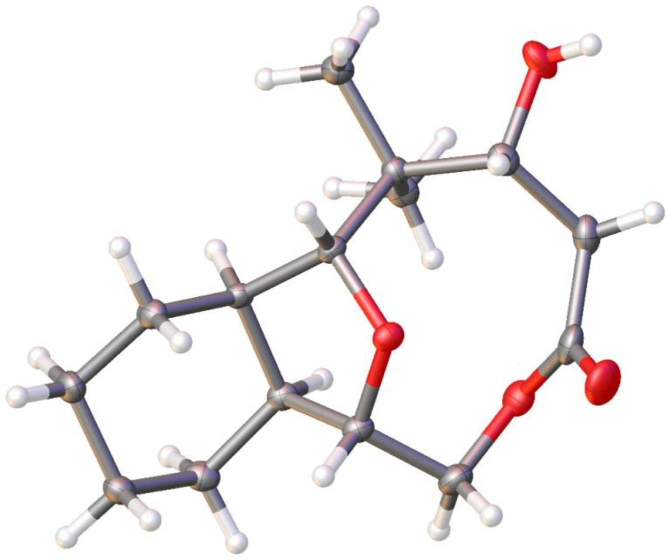

Formula: $C_{15}H_{24}O_4$; M_r = 268.34; crystal dimensions: 0.45 x 0.4 x 0.38 mm; crystal system: orthorhombic; space group: *P2₁2₁2₁*; a = 9.16700(10) Å, b = 10.5275(2) Å, c = 14.3960(3) Å, α = 90°, β = 90°, γ = 90°; V = 1389.29(4) $Å^3$; Z = 4 ρ_{calcd} = 1.283 Mg/m^3; μ = 0.091 mm^{-1}; Mo $K\alpha$ radiation, λ = 0.71073 Å; T = 120(2) K; $2\Theta_{max}$ = 55.4°; 14418/3181 measured/independent reflections; R_{int}: 0.0361; R = 0.0324, wR = 0.0756; $\Delta\rho_{max}$ = 0.178 $eÅ^{-3}$, $\Delta\rho_{min}$ = −0.174 $eÅ^{-3}$. Colourless block crystals diffracted well. The data were collected on a Nonius-Kappa CCD area detector mounted at the window of an FR591 rotating anode generator with a Mo anode and equipped with an Oxford Cryosystems cryostream device. Nonius COLLECT[6] was used to record images and HKL (Denzo and Scalepack)[7] was used for for data integration. The structure was solved by direct methods using SHELXS[11] and refined on F_o^2 by full-matrix least squares refinement using SHELXL.[9] All non-hydrogen atoms were refined with anisotropic displacement parameters. Hydrogen atoms were added at calculated positions and refined using a riding model with isotropic displacement parameters based on the equivalent isotropic displacement parameter (Ueq) of the parent atom. The structure was deposited on the Cambridge Structural Database with the deposition number CCDC 1435148.

NCI cytotoxicity screening compounds

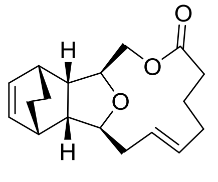

40

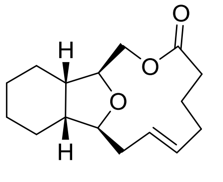

28

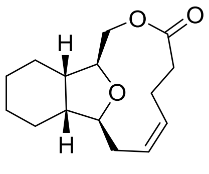

21

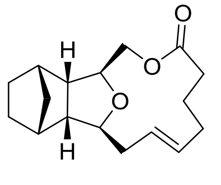

42

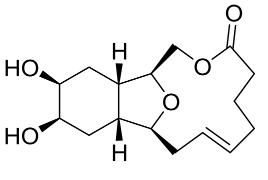

36

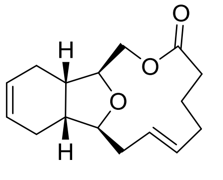

22

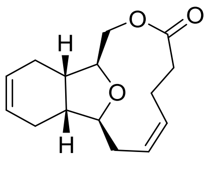

26

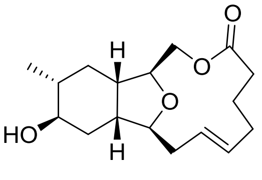

39

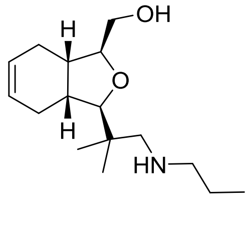

58

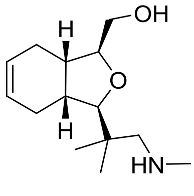

57

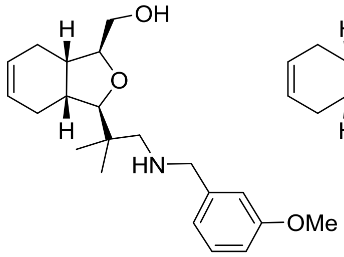

56

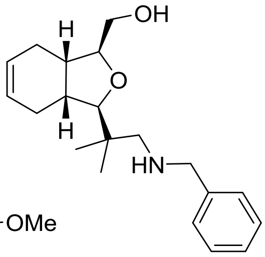

55

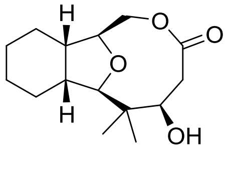

69

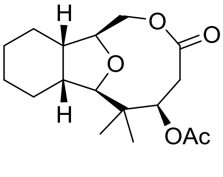

70

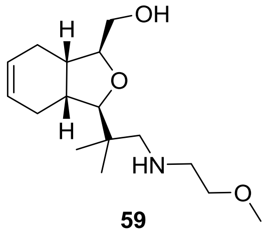

59

Figure S2: Compounds screened at the NCI for cytotoxicity in a 60 cell line panel

References

1. J. Mann, A. Thomas, *Chem. Commun*. **1985**, 737-738.
2. S. Hanessian, P. J. Murray, *J Org Chem*, **1987**, *52*, 1170-1172.
3. J. Mann, A. Thomas, *J Chem Soc., Perkin Trans 1*, **1986**, 2279-2285.
4. Simca-P+ 12.0, Umertrics, Umeâ, Sweden, http://www.umetrics.com/
5. Spotfile Decision Site, Tibco, Palo Alto, CA, USA, http://spotfire.tibco.com/
6. R. W. W. Hooft, COLLECT data collection software, Nonius B.V., 1998.
7. Z. Otwinowski, W. Minor, Macromolecular Crystallography, Pt A. 1997; 276, 307-326.
8. G. M. Sheldrick, Acta Cryst., 2015, A71, 3-8.
9. G. M. Sheldrick, Acta Cryst., 2015, C71, 3-8.
10. CrysAlisPro Software System, Rigaku Oxford Diffraction, Yarnton, Oxford, UK, 2015.
11. G. M. Sheldrick, Acta Cryst., 2008, A64, 112-122.

(S)-5-((*tert*-Butyldiphenylsilyloxy)methyl)furan-2-(5*H*)one **8**

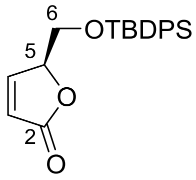

8

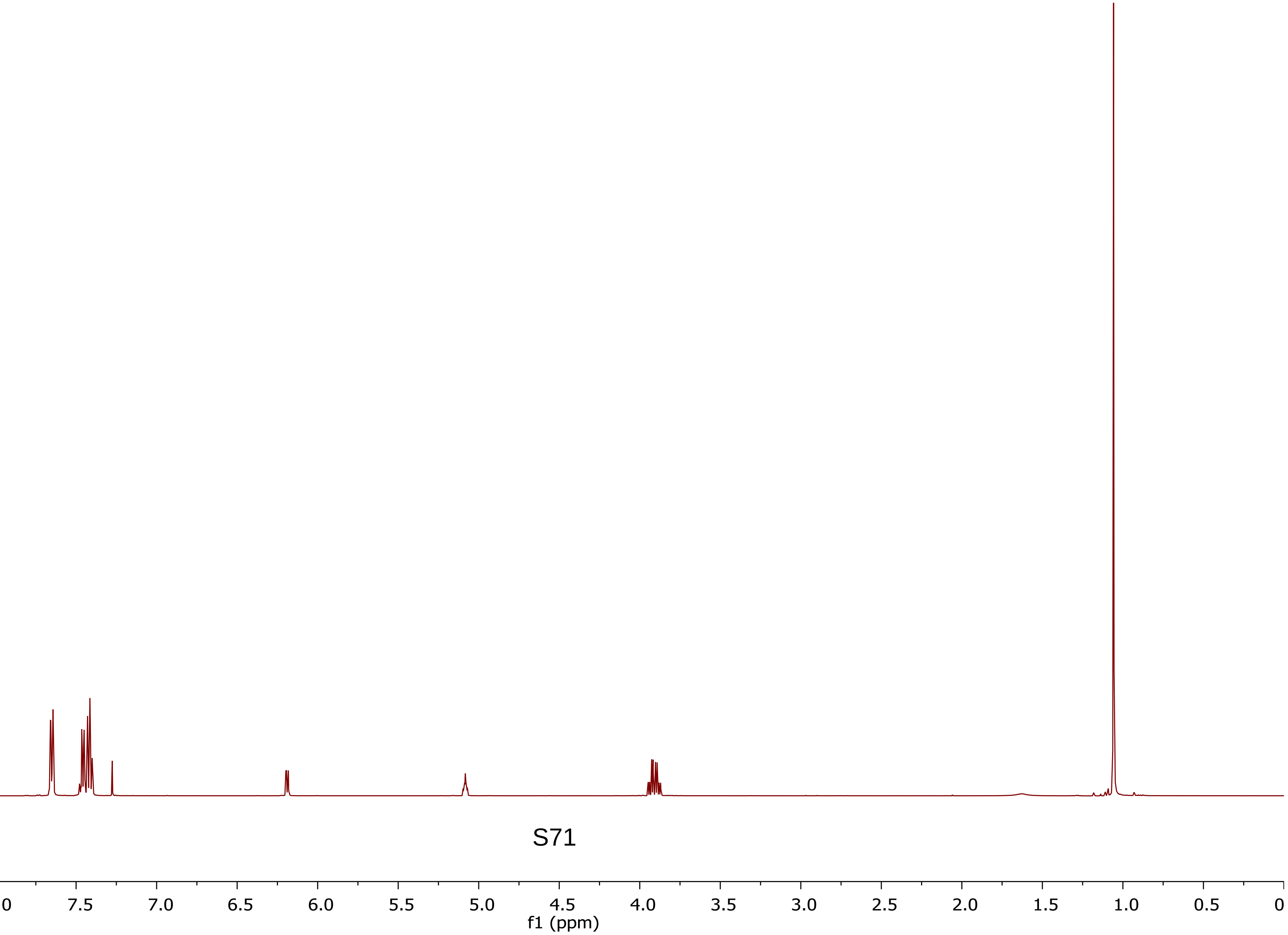

(S)-5-((*tert*-Butyldiphenylsilyloxy)methyl)furan-2-(5*H*)one **8**

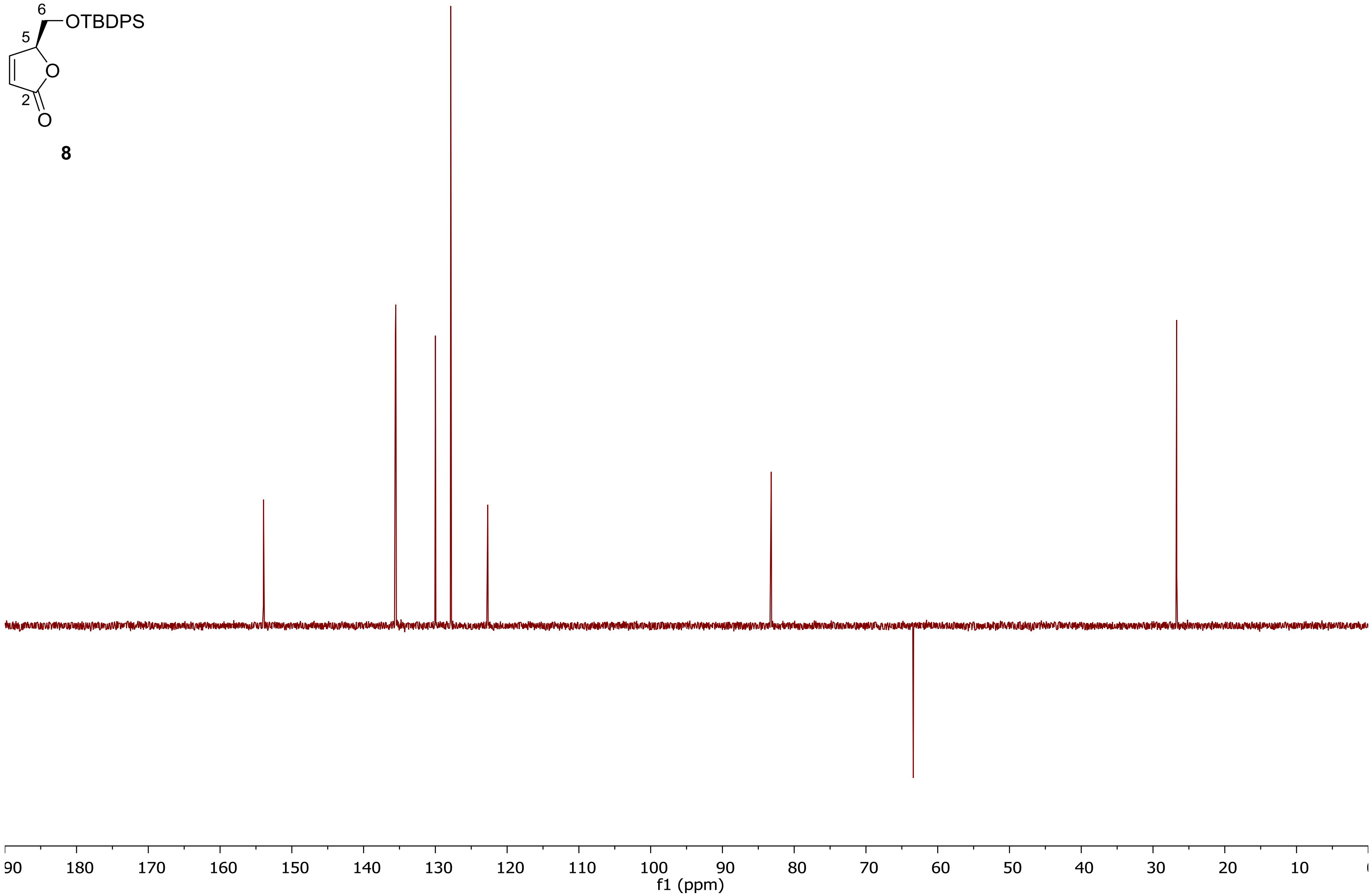

(3*S*)-3-((*tert*-Butyldiphenylsilyloxy)methyl)-3a,4,7,7a-tetrahydroisobenzofuran-1-(3*H*)-one **10**

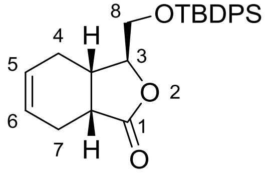

10

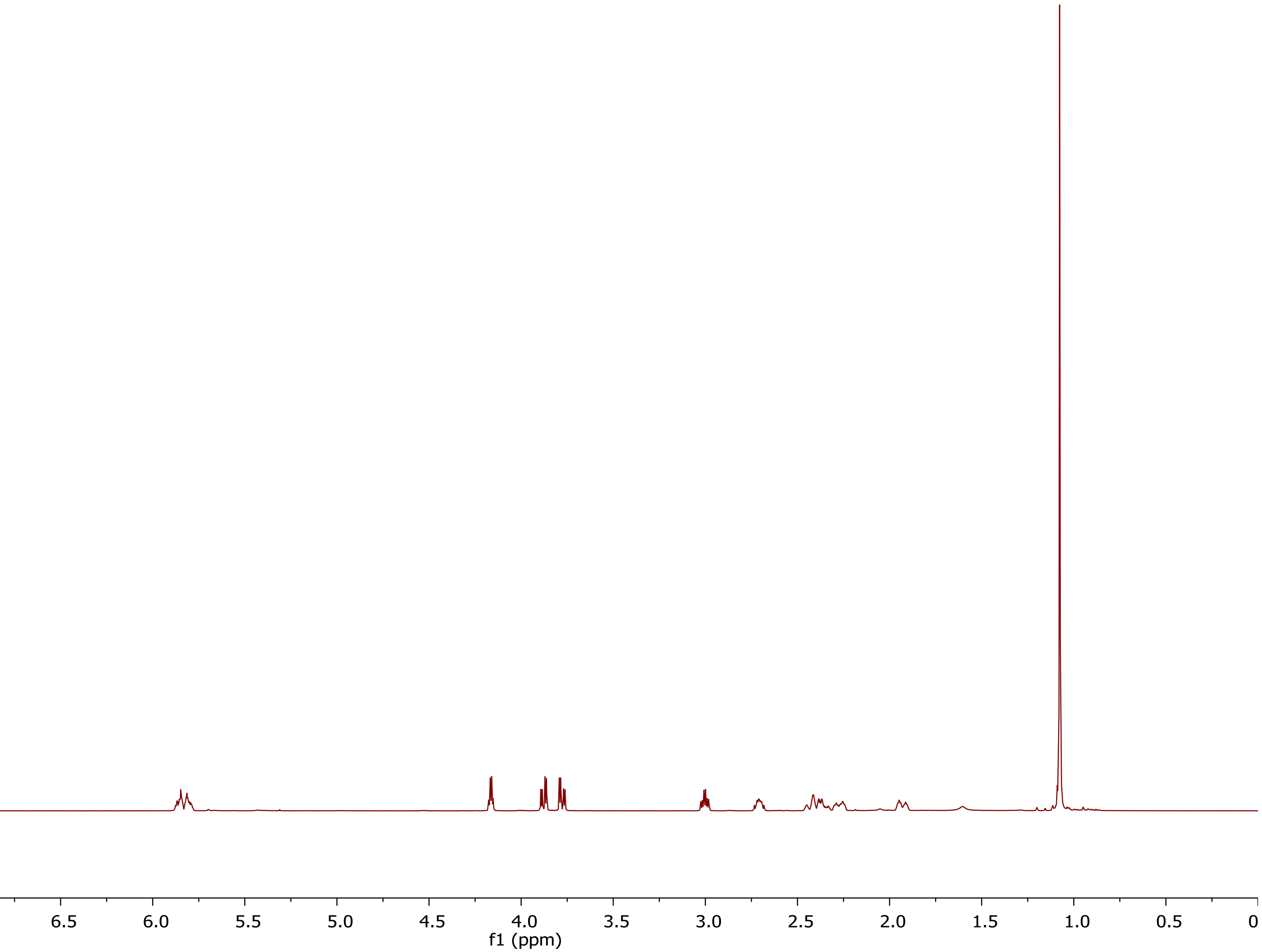

(3*S*)-3-((*tert*-Butyldiphenylsilyloxy)methyl)-3a,4,7,7a-tetrahydroisobenzofuran-1-(3*H*)-one **10**

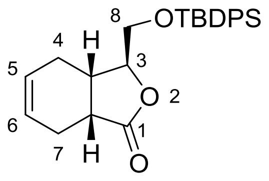

10

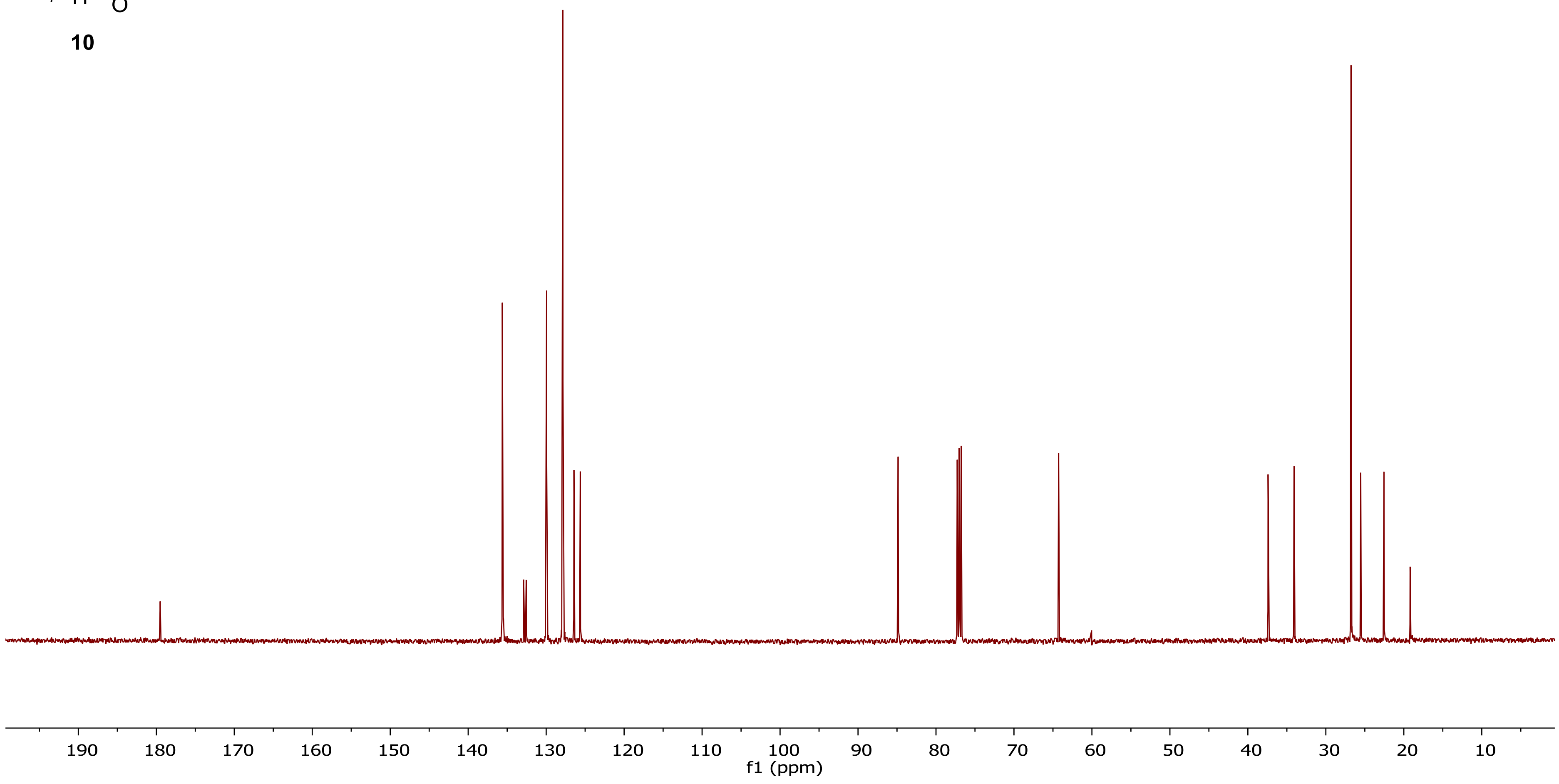

(3*S*,3a*S*,7a*R*)-3-((*tert*-Butyldiphenylsilyloxy)methyl)-1,3,3a,4,7,7a-hexahydroisobenzofuran-1-ol **11**

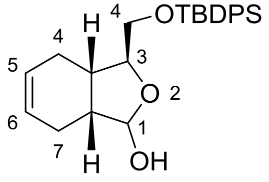

11

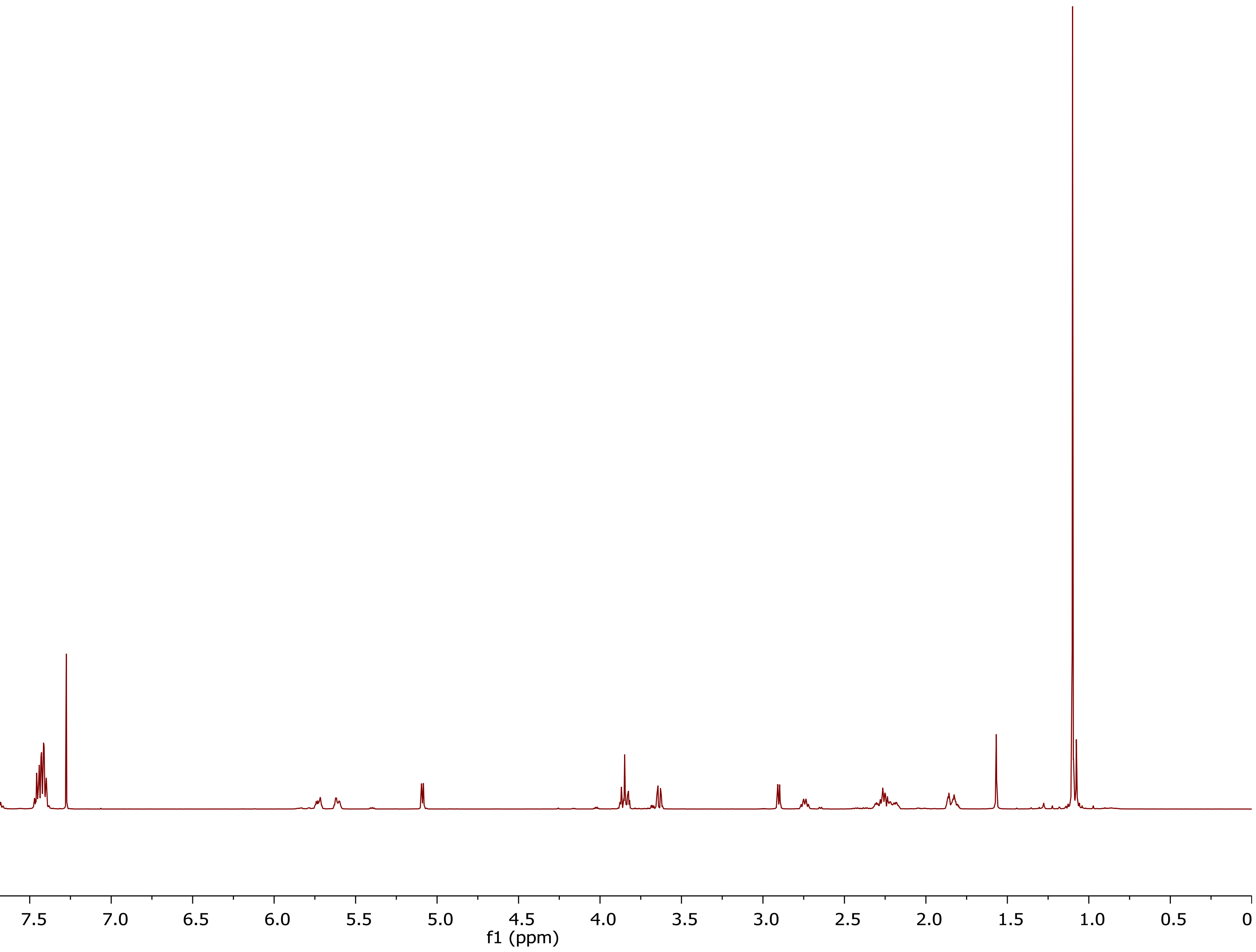

(3*S*,3a*S*,7a*R*)-3-((*tert*-Butyldiphenylsilyloxy)methyl)-1,3,3a,4,7,7a-hexahydroisobenzofuran-1-ol **11**

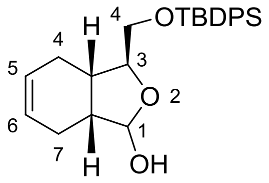

11

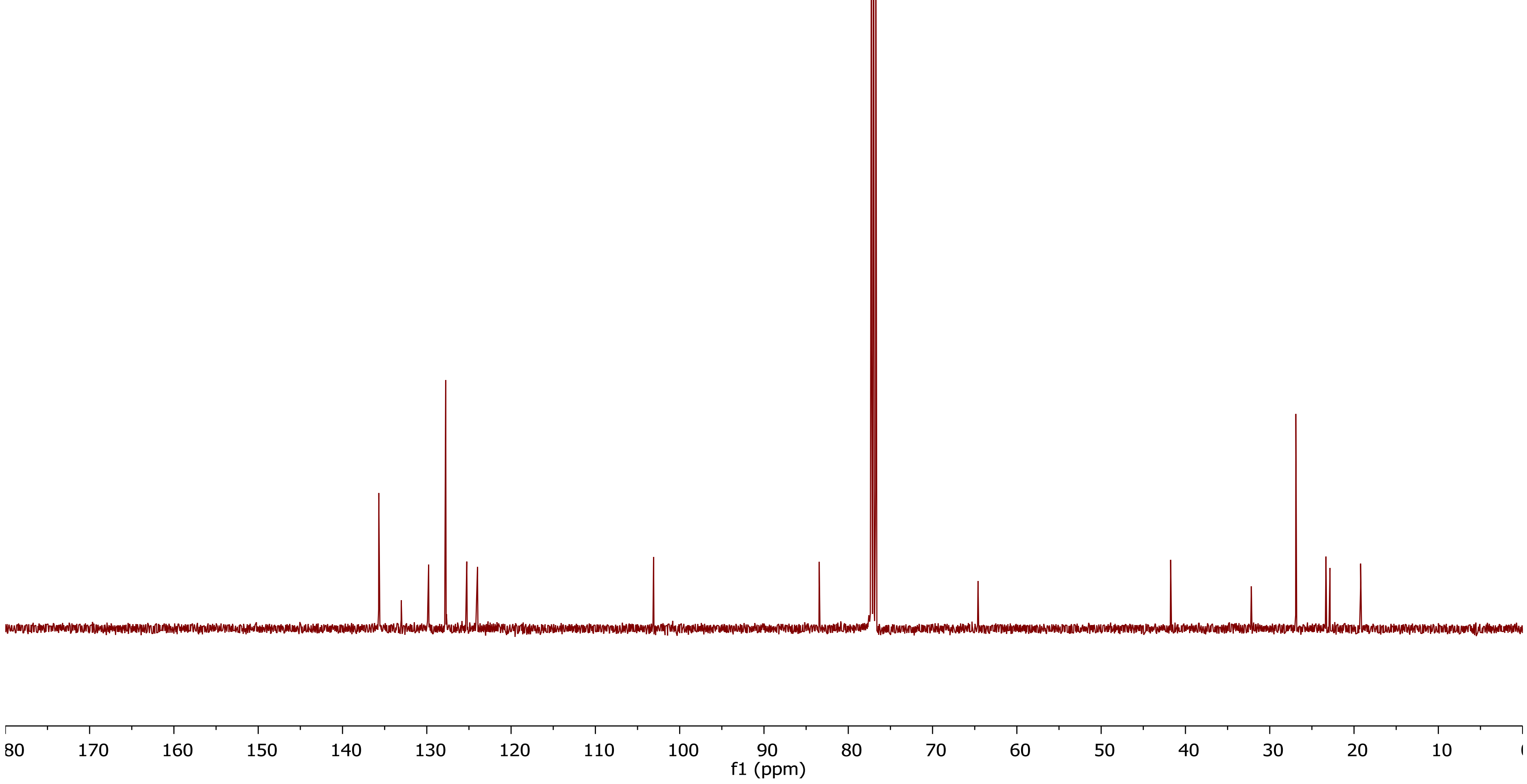

(((1*S*,3*S*,3a*R*,7a*S*)-3-Allyl-1,3,3a,4,7,7a-hexahydroisobenzofuran-1-yl)methoxy)(*tert*-butyl)diphenylsilane **12**

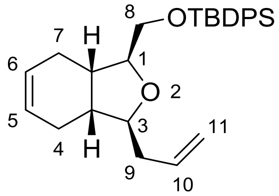

12

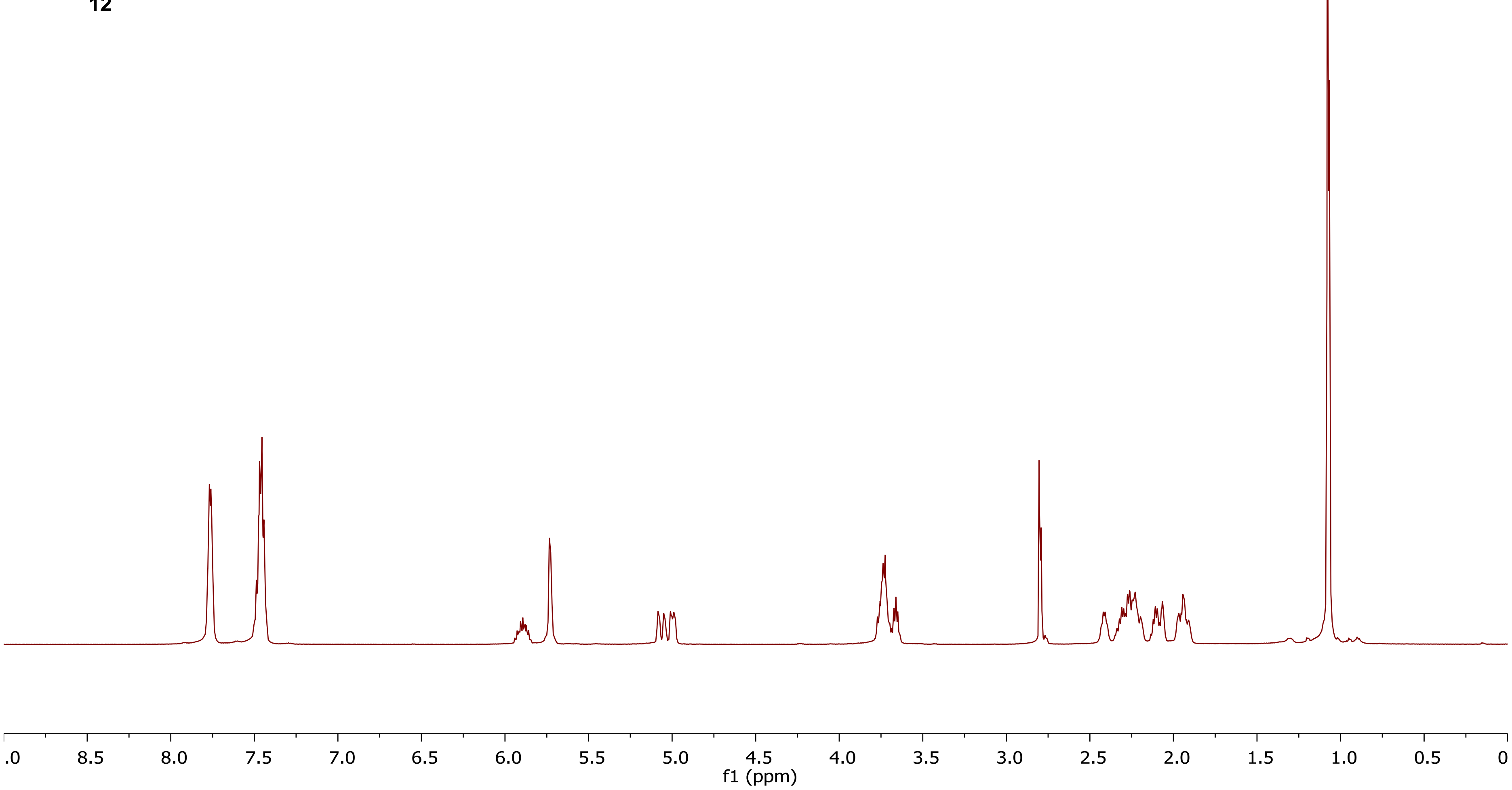

(((1*S*,3*S*,3a*R*,7a*S*)-3-Allyl-1,3,3a,4,7,7a-hexahydroisobenzofuran-1-yl)methoxy)(*tert*-butyl)diphenylsilane **12**

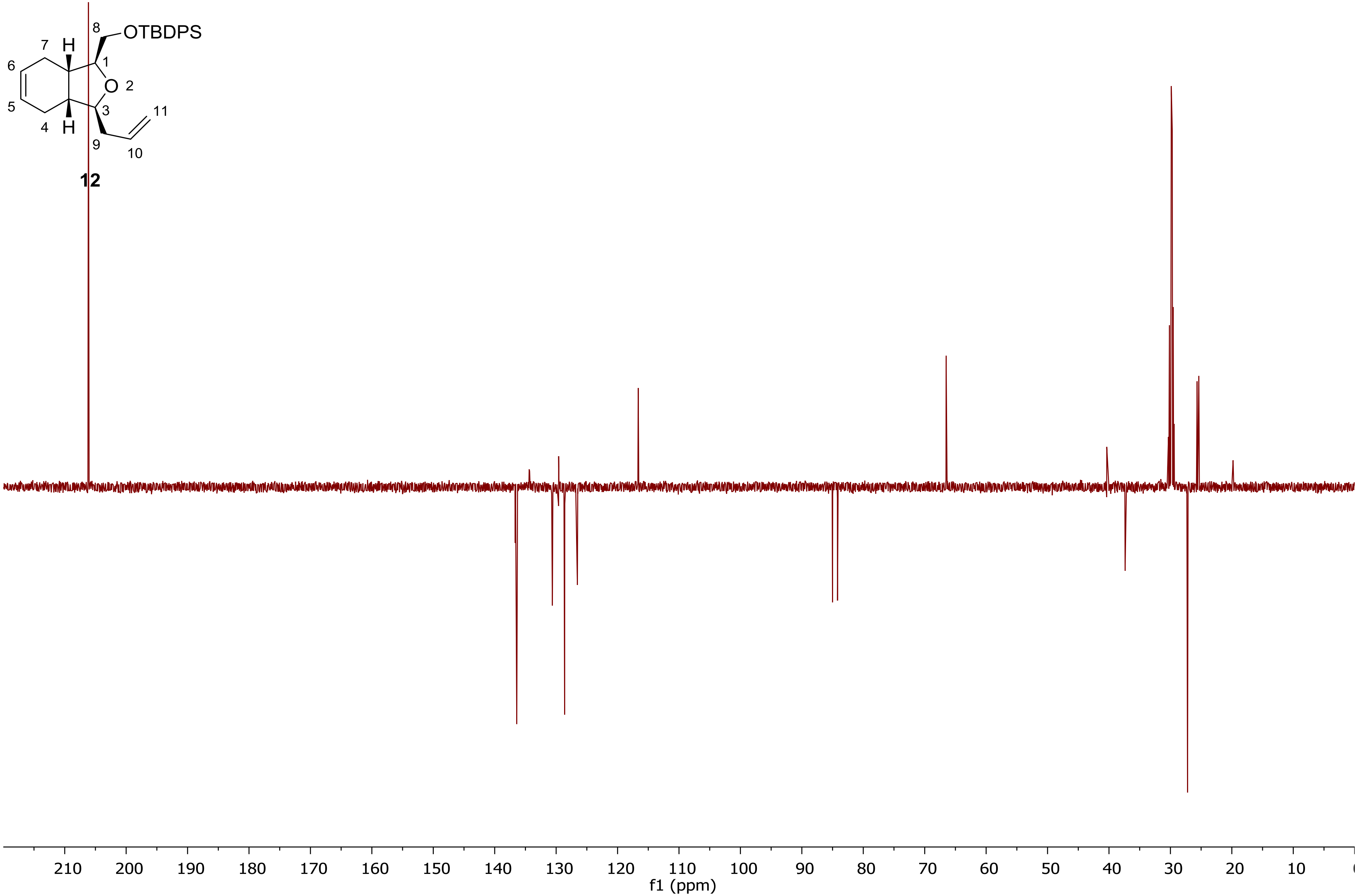

((1*S*,3*S*,3a*R*,7a*S*)-3-Allyl-1,3,3a,4,7,7a-hexahydroisobenzofuran-1-yl)methanol **14**

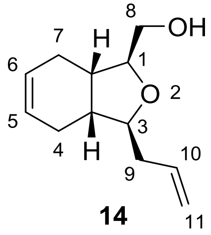

14

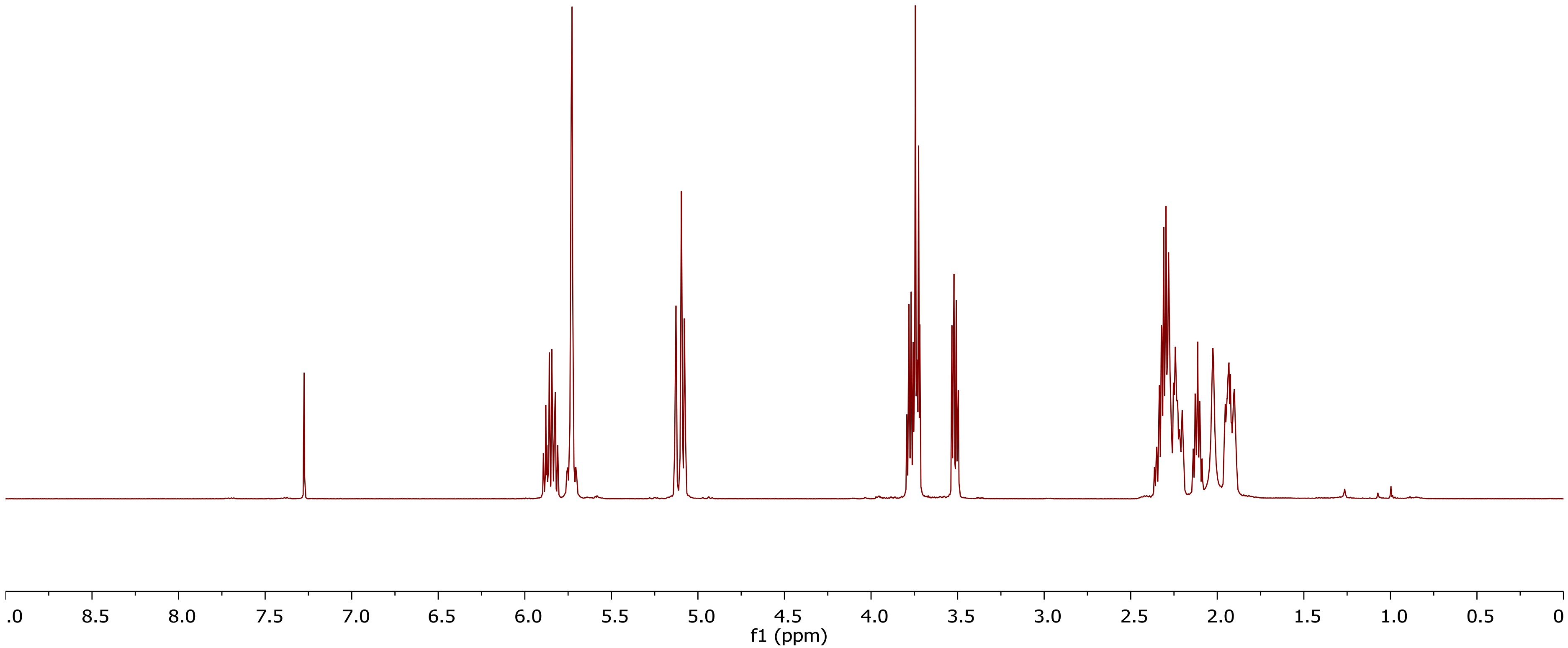

((1*S*,3*S*,3a*R*,7a*S*)-3-Allyl-1,3,3a,4,7,7a-hexahydroisobenzofuran-1-yl)methanol **14**

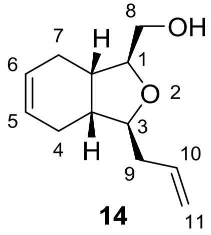

14

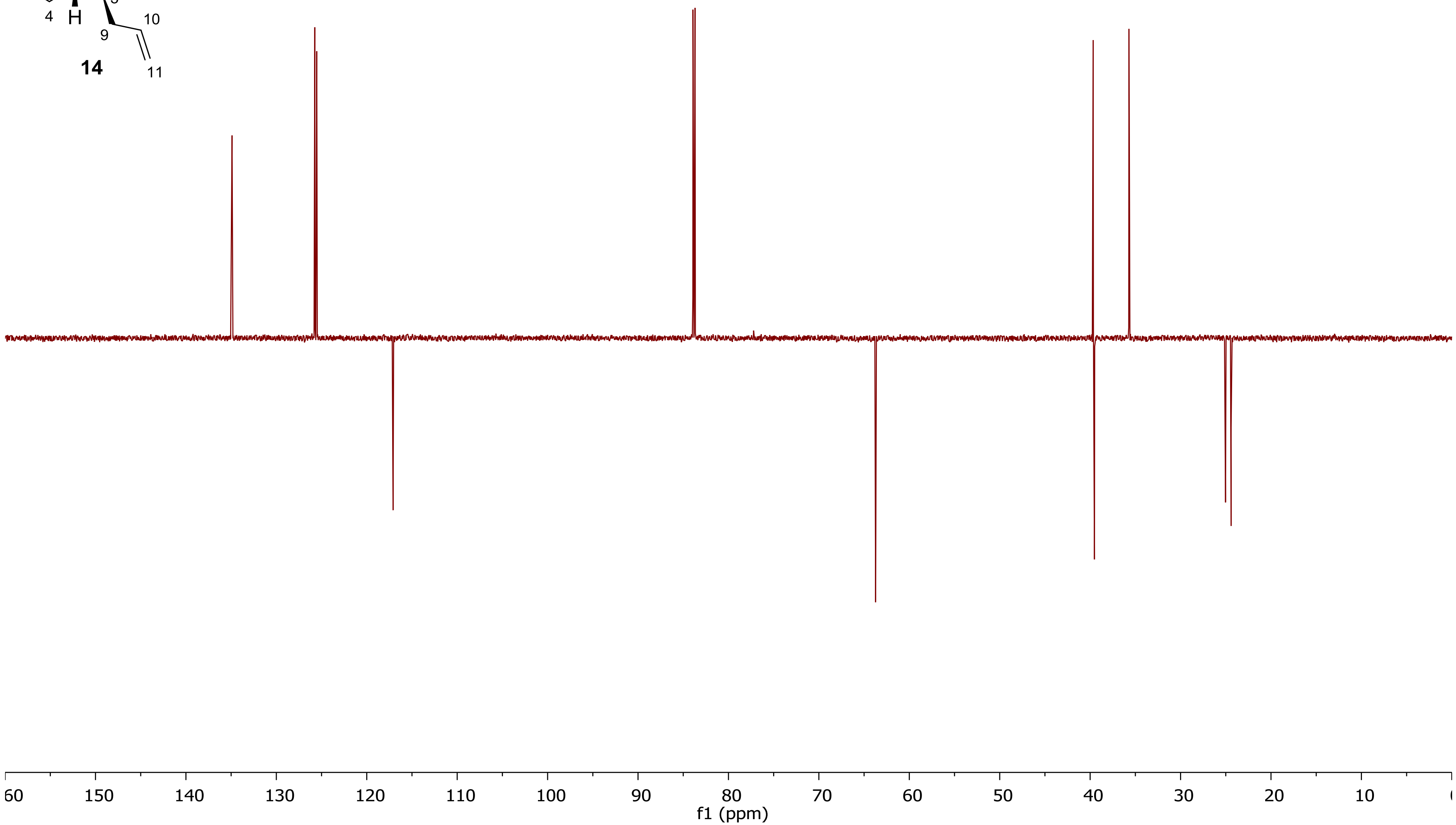

But-2'-enoic acid (1*S*, 3*S*, 3a*R*, 7a*S*)-allyl-1, 3, 3a, 4, 7, 7a-hexahydro-isobenzofuran-8-ylmethyl ester **15**

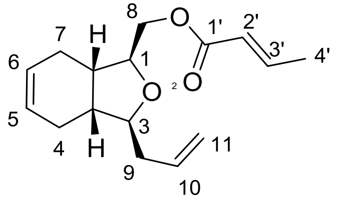

15

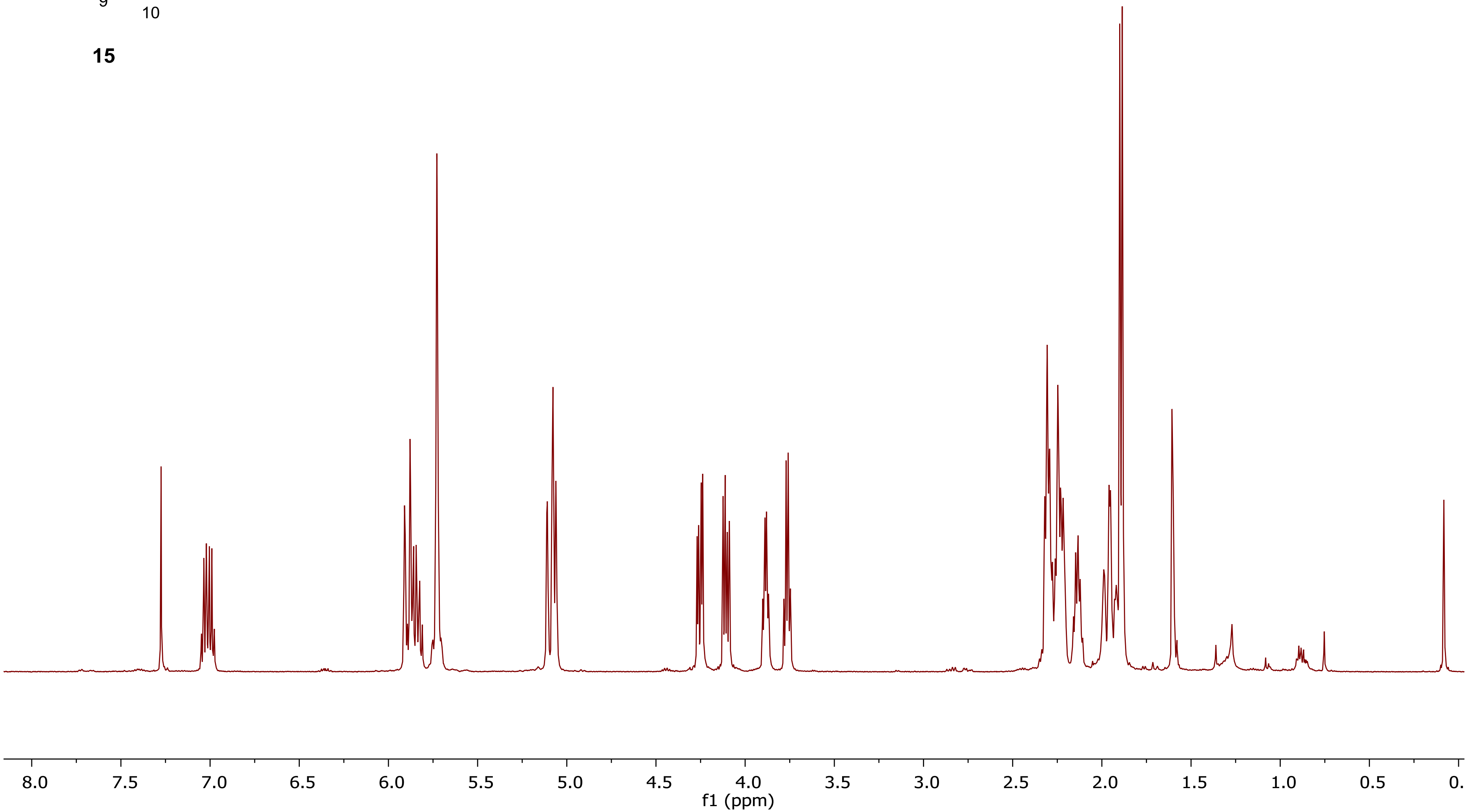

But-2’-enoic acid (1*S*, 3*S*, 3a*R*, 7a*S*)-allyl-1, 3, 3a, 4, 7, 7a-hexahydro-isobenzofuran-8-ylmethyl ester **15**

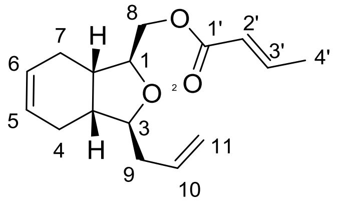

15

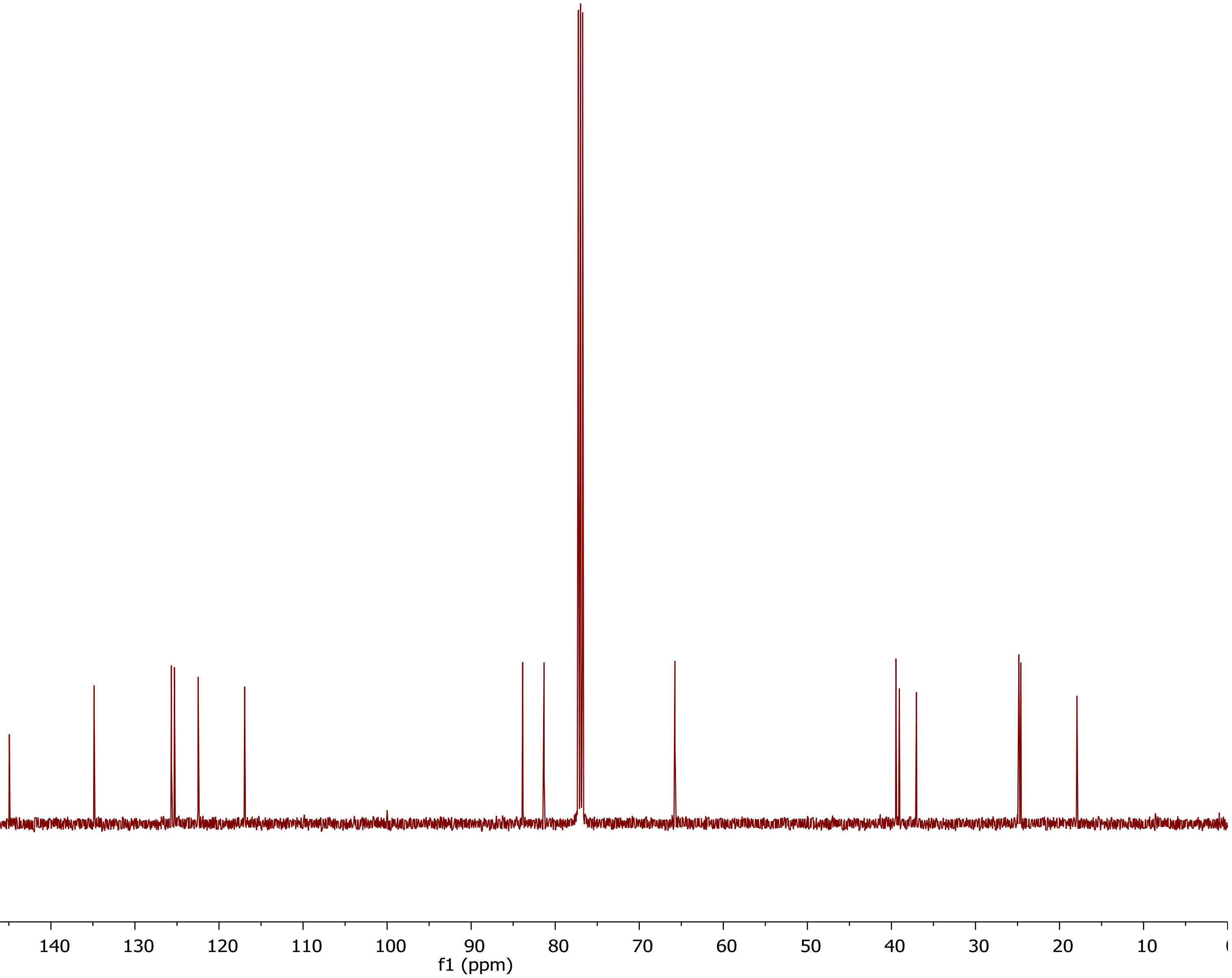

((1*S*,3*S*,3a*R*,7a*S*)-3-Allyl-1,3,3a,4,7,7a-hexahydroisobenzofuran-1-yl)methyl but-3’-enoate **16**

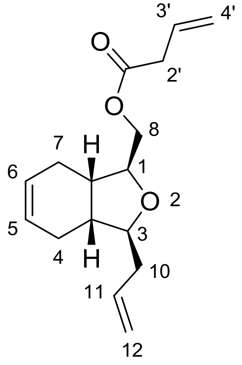

16

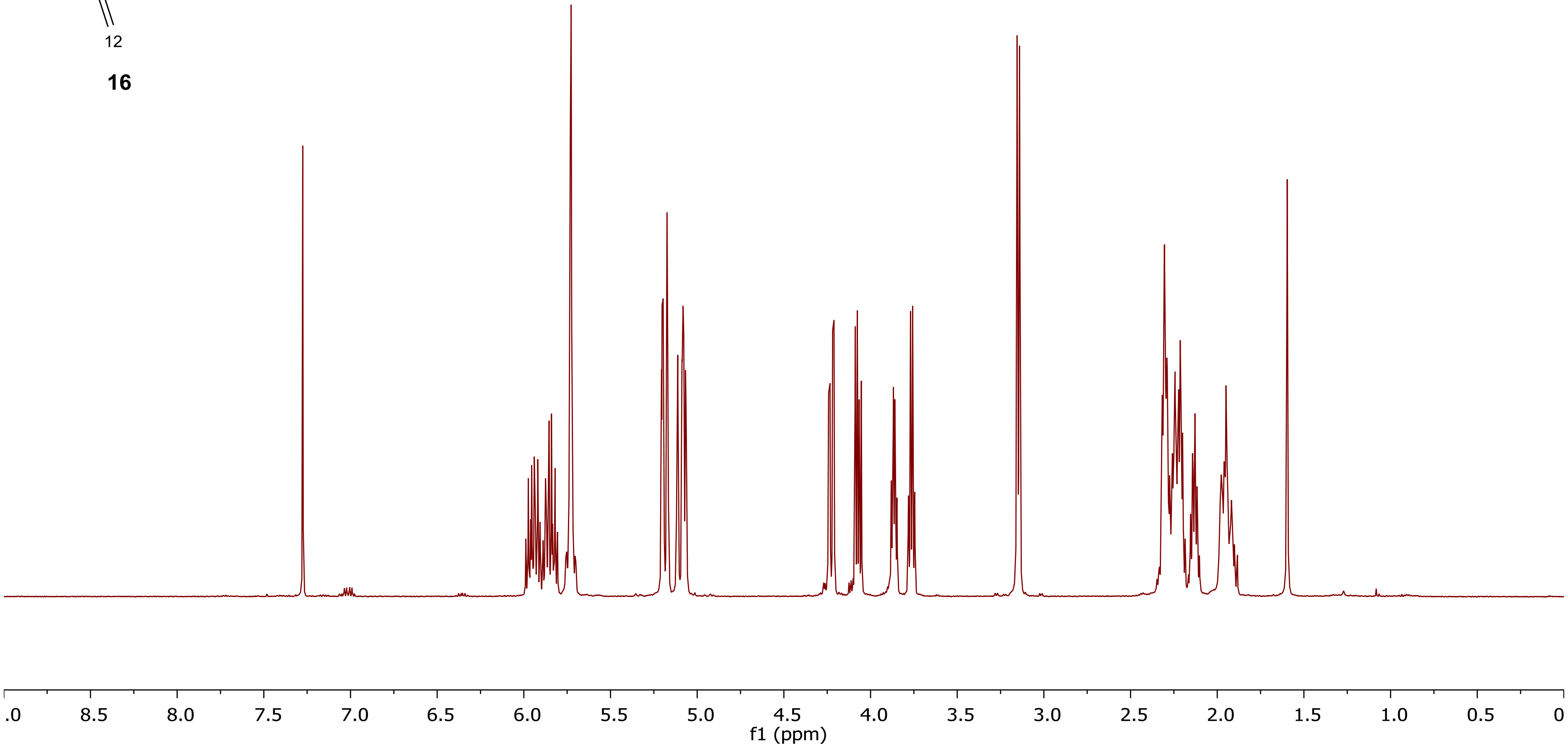

((1*S*,3*S*,3a*R*,7a*S*)-3-Allyl-1,3,3a,4,7,7a-hexahydroisobenzofuran-1-yl)methyl but-3'-enoate **16**

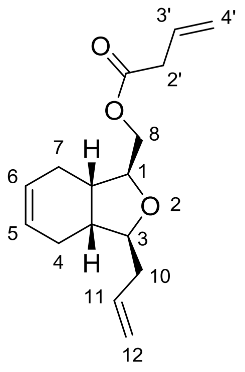

16

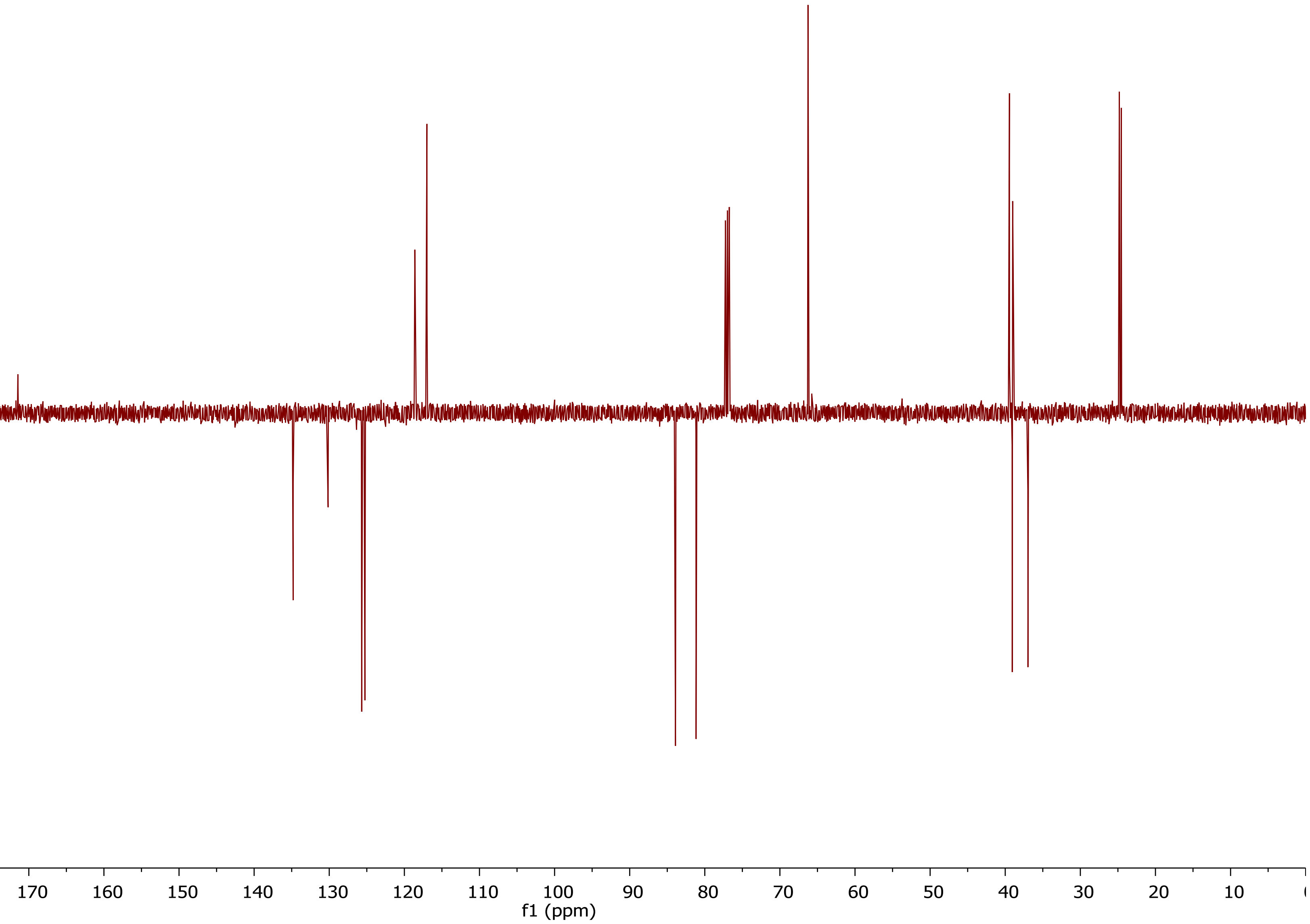

Pent-4’enoic acid (1*S*, 3*S*, 3a*R*, 7a*S*)-allyl-1, 3, 3a, 4, 7, 7a-hexahydro-isobenzofuran-8-ylmethyl ester **17**

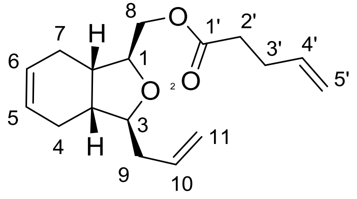

17

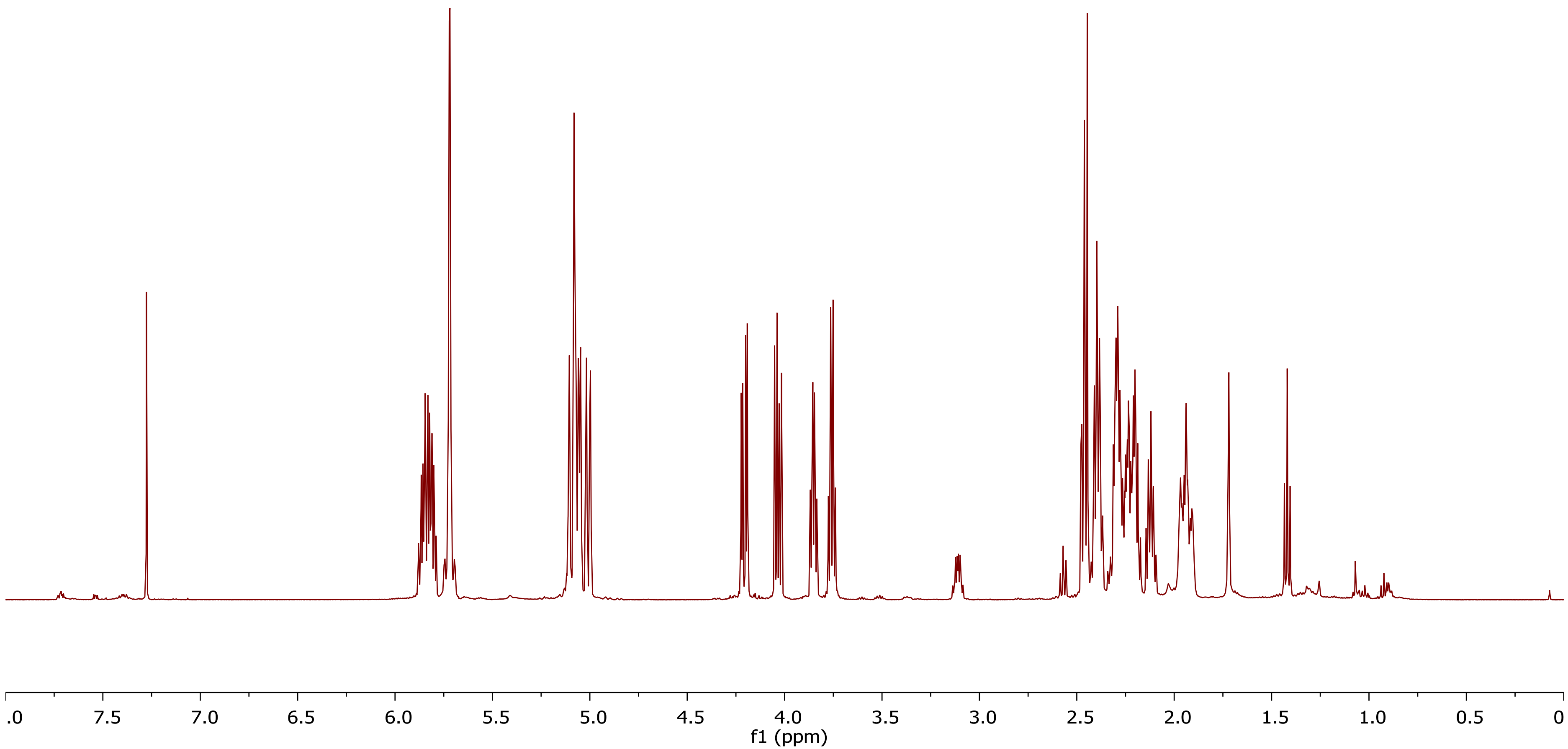

Pent-4’enoic acid (1*S*, 3*S*, 3a*R*, 7a*S*)-allyl-1, 3, 3a, 4, 7, 7a-hexahydro-isobenzofuran-8-ylmethyl ester **17**

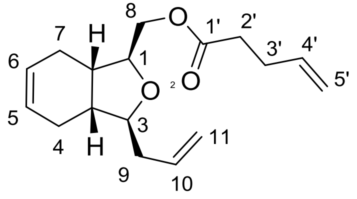

17

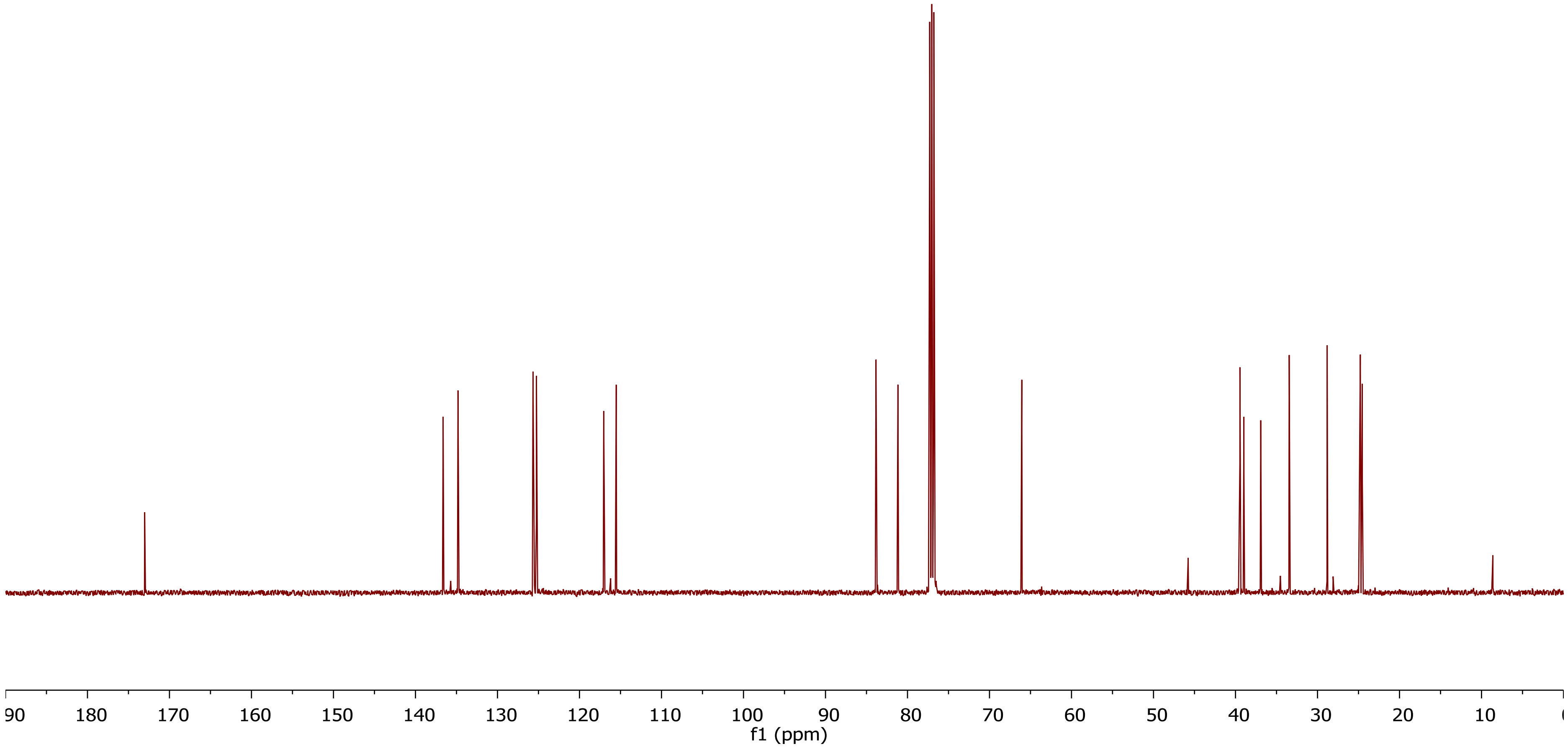

((1S,3S,3aR,7aS)-3-Allyl-1,3,3a,4,7,7a-hexahydroisobenzofuran-1-yl)methyl hex-5-enoate **18**

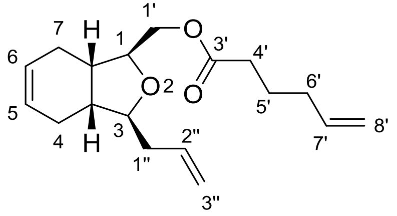

18

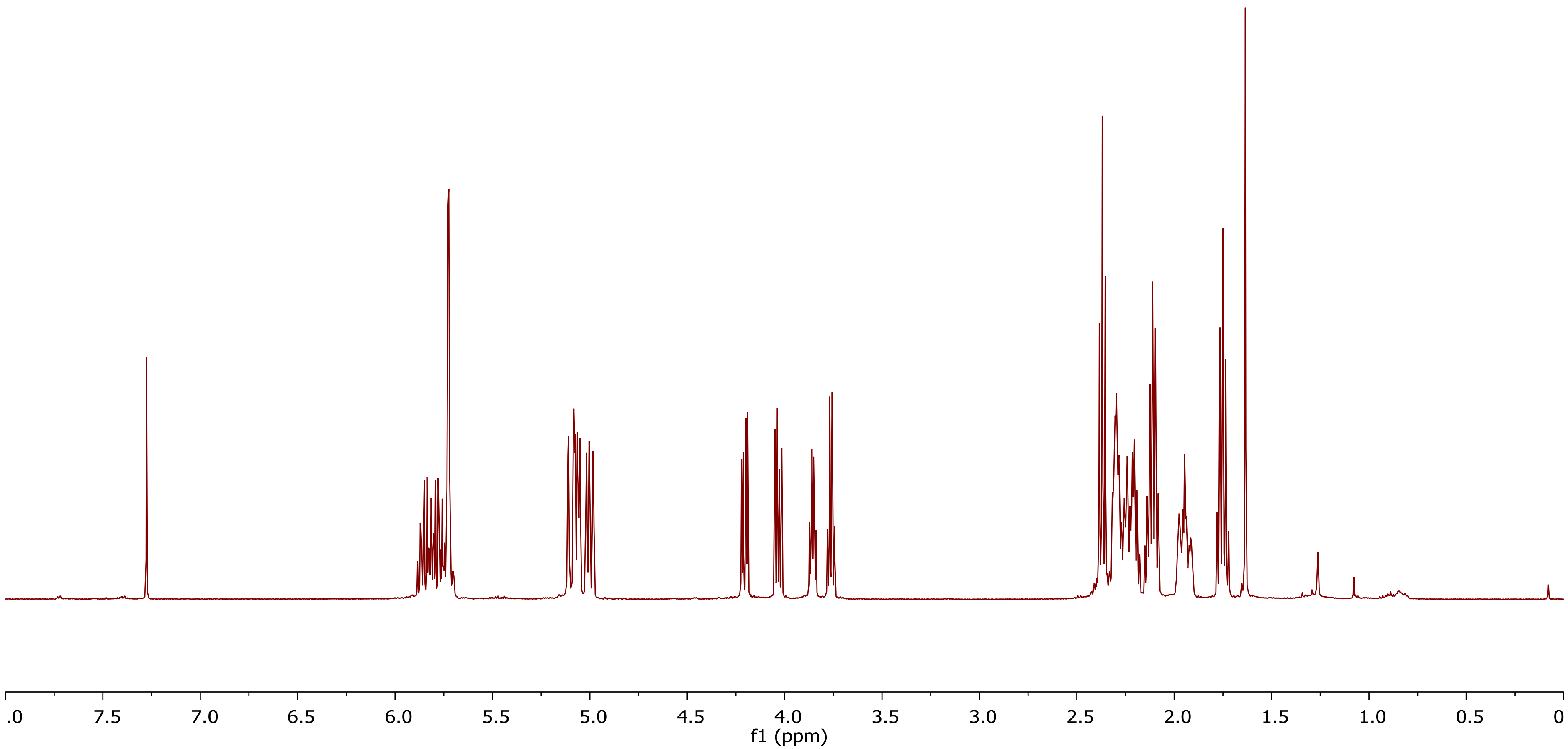

((1S,3S,3aR,7aS)-3-Allyl-1,3,3a,4,7,7a-hexahydroisobenzofuran-1-yl)methyl hex-5-enoate **18**

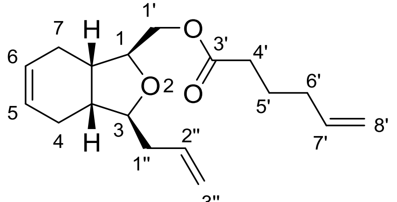

18

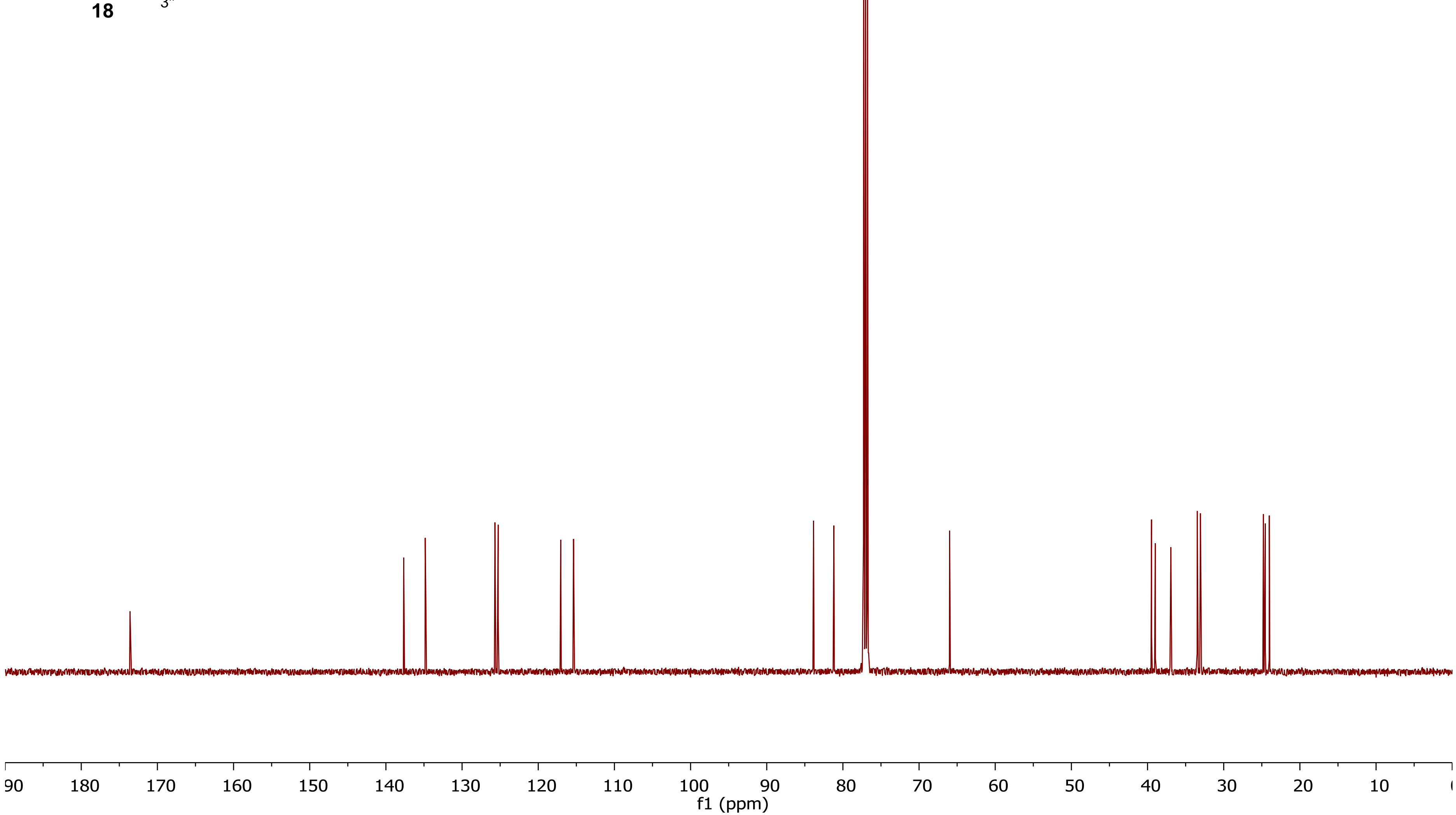

(*Z*)-(1*S*, 9*S*, 10*R*, 15*S*)-3, 16-Dioxa-tricyclo[7.6.1.0 ^{10,15}]hexadeca-6,12-diene-4-one **20**

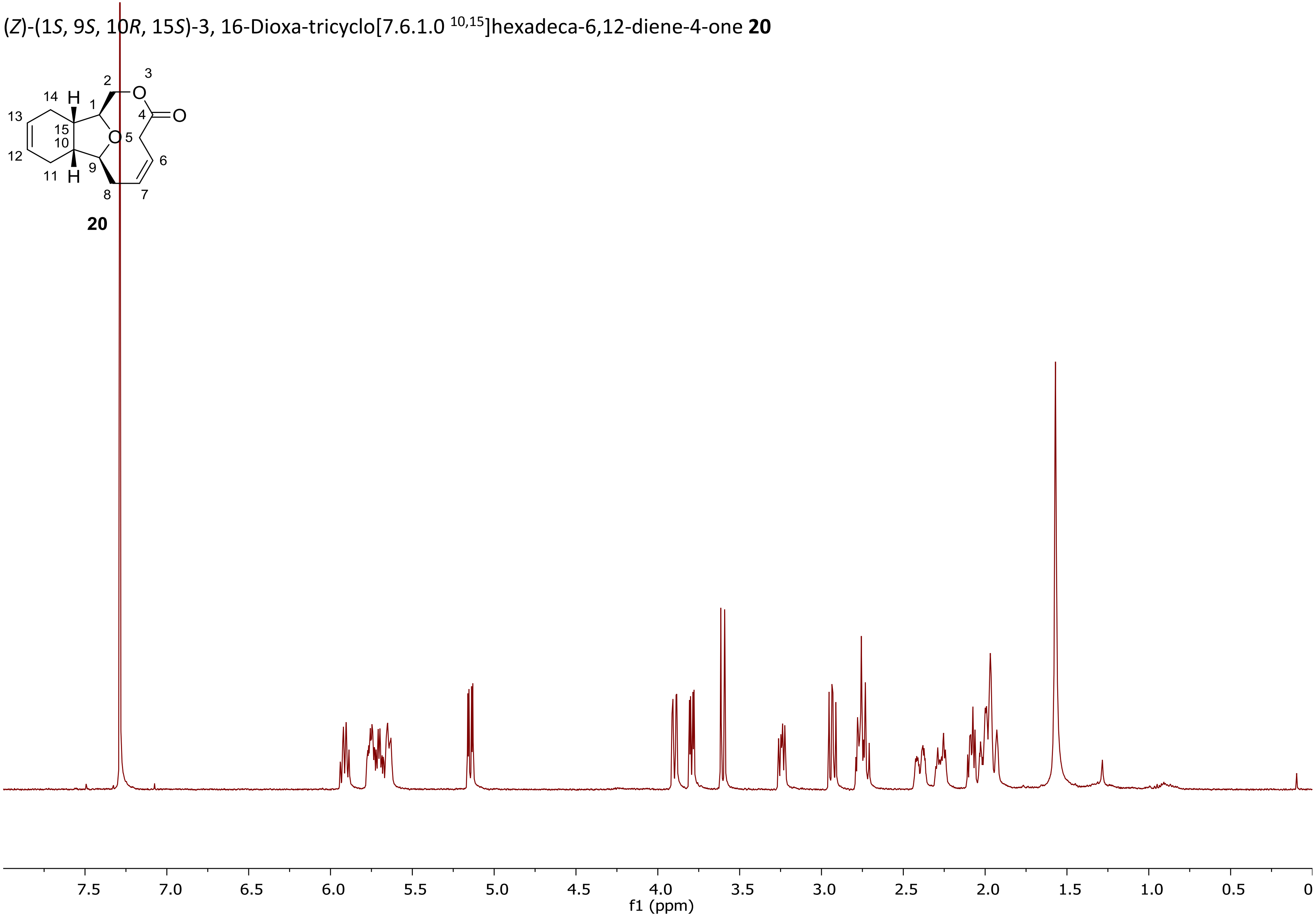

(*Z*)-(1*S*, 9*S*, 10*R*, 15*S*)-3, 16-Dioxa-tricyclo[7.6.1.0 ^{10,15}]hexadeca-6,12-diene-4-one **20**

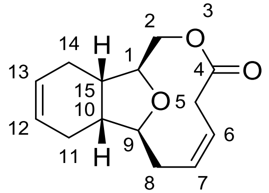

20

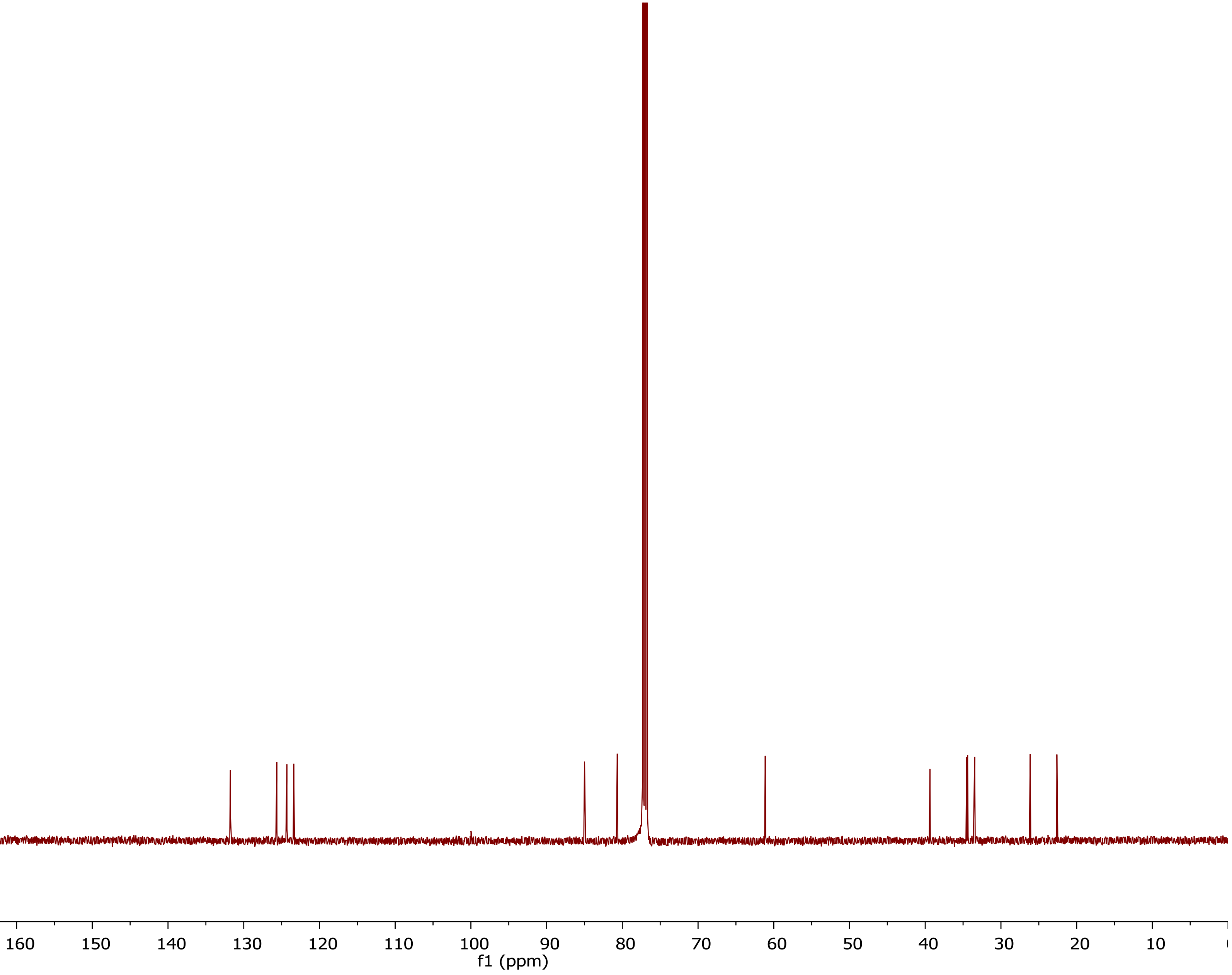

(*Z*)-(1*S*,10*S*,11*R*,16*S*)-3,17-Dioxa-tricyclo[8.6.1.0^{11,16}]heptadeca-7,13-diene-4-one **21**

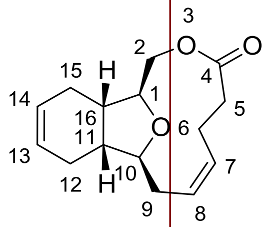

21

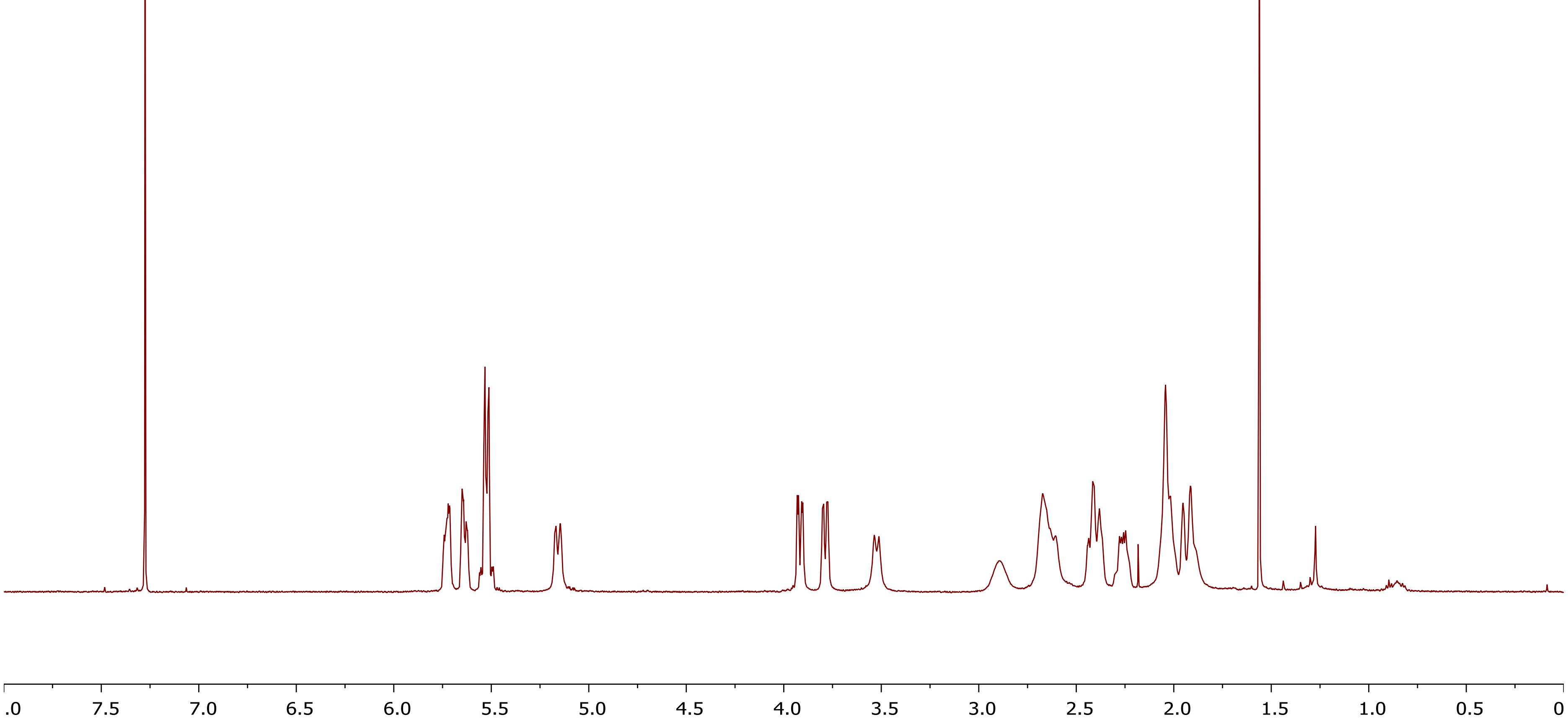

(*Z*)-(1*S*,10*S*,11*R*,16*S*)-3,17-Dioxa-tricyclo[8.6.1.0^{11,16}]heptadeca-7,13-diene-4-one **21** acetone contaminant

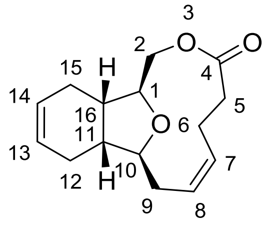

21

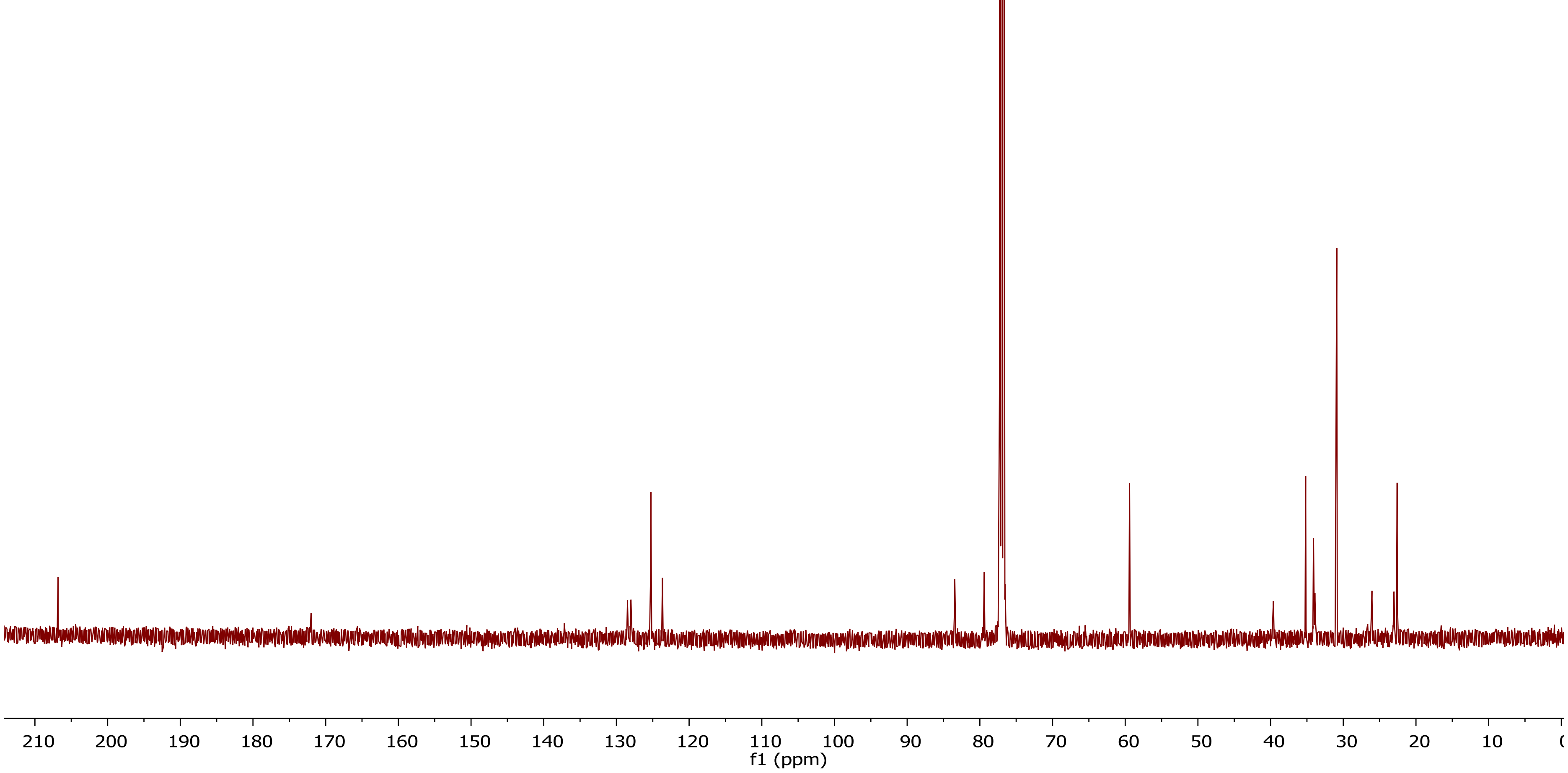

(Z)-(1*S*,11*S*,12*R*,17*S*)-3,18-Dioxatricyclo[9.6.1.0^{12,17}]octadeca-8,14-dien-4-one **22**

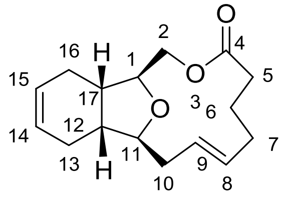

22

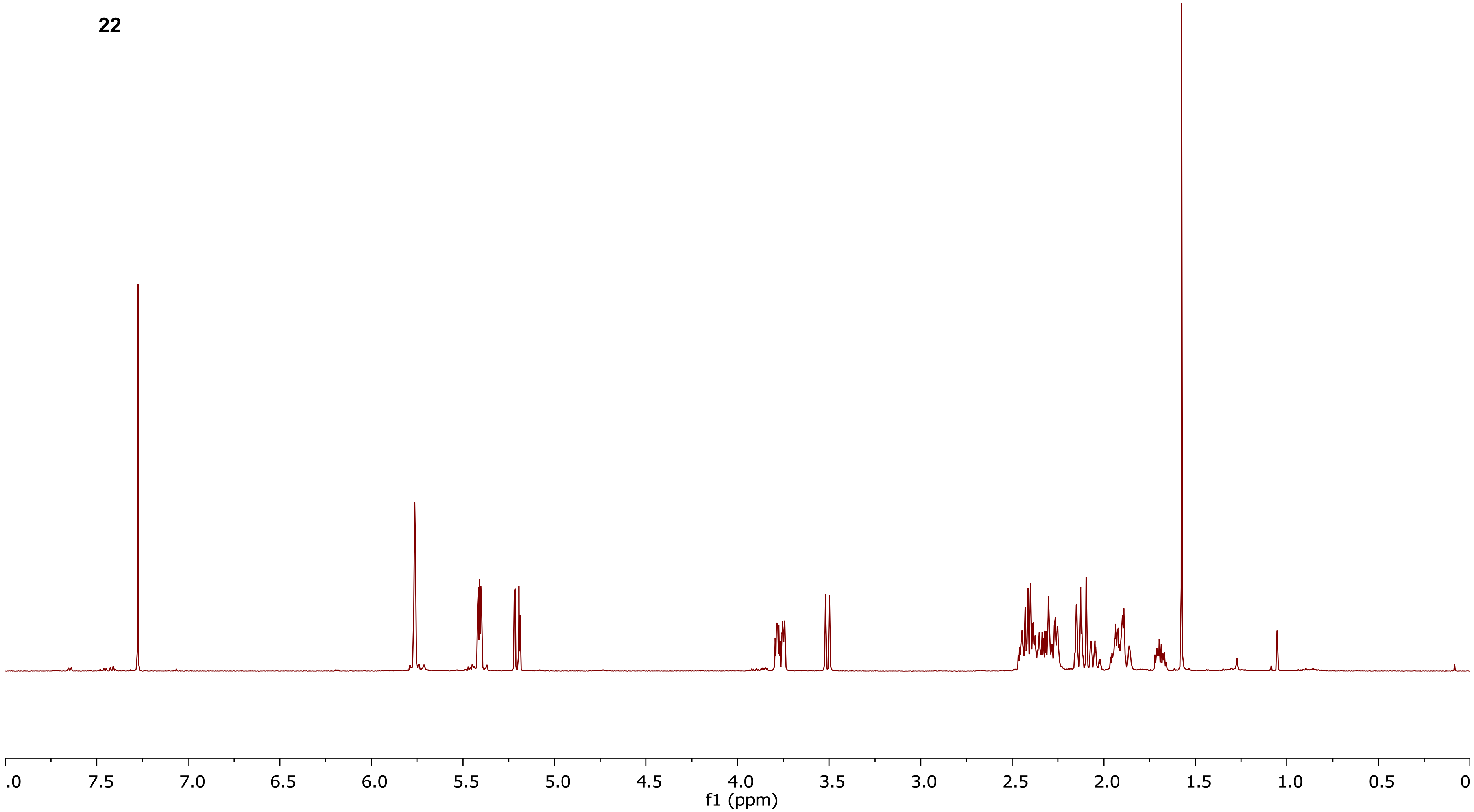

(Z)-(1*S*,11*S*,12*R*,17*S*)-3,18-Dioxatricyclo[9.6.1.0^{12,17}]octadeca-8,14-dien-4-one **22**

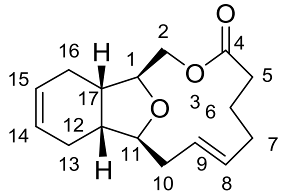

22

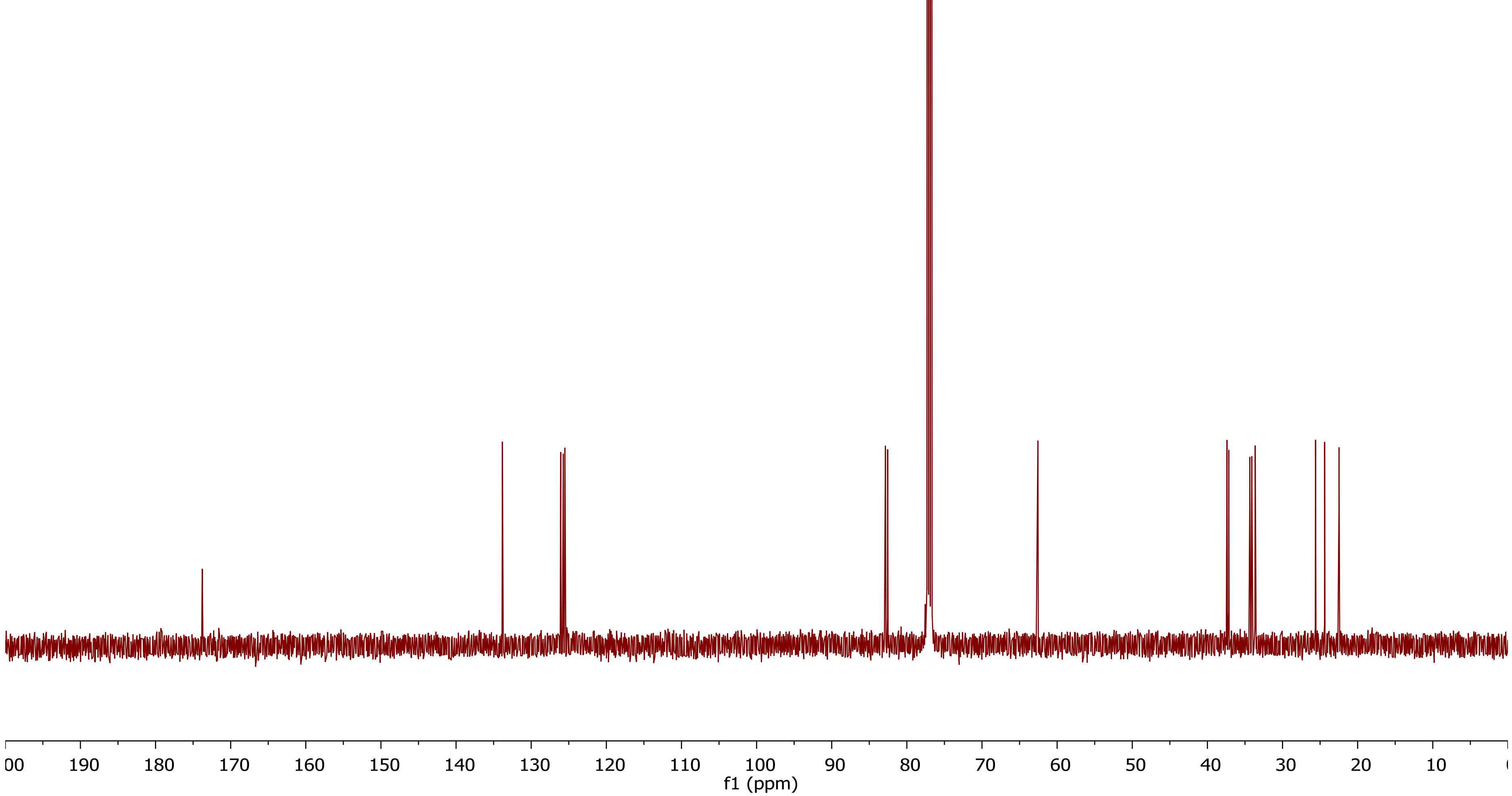

(3*S*,3a*S*,7a*R*)-3-((*tert*-Butyldiphenylsilyloxy)methyl)hexahydroisobenzofuran-1(3*H*)-one **23a**

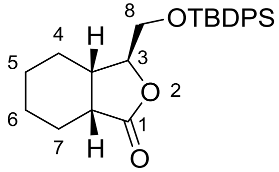

23a

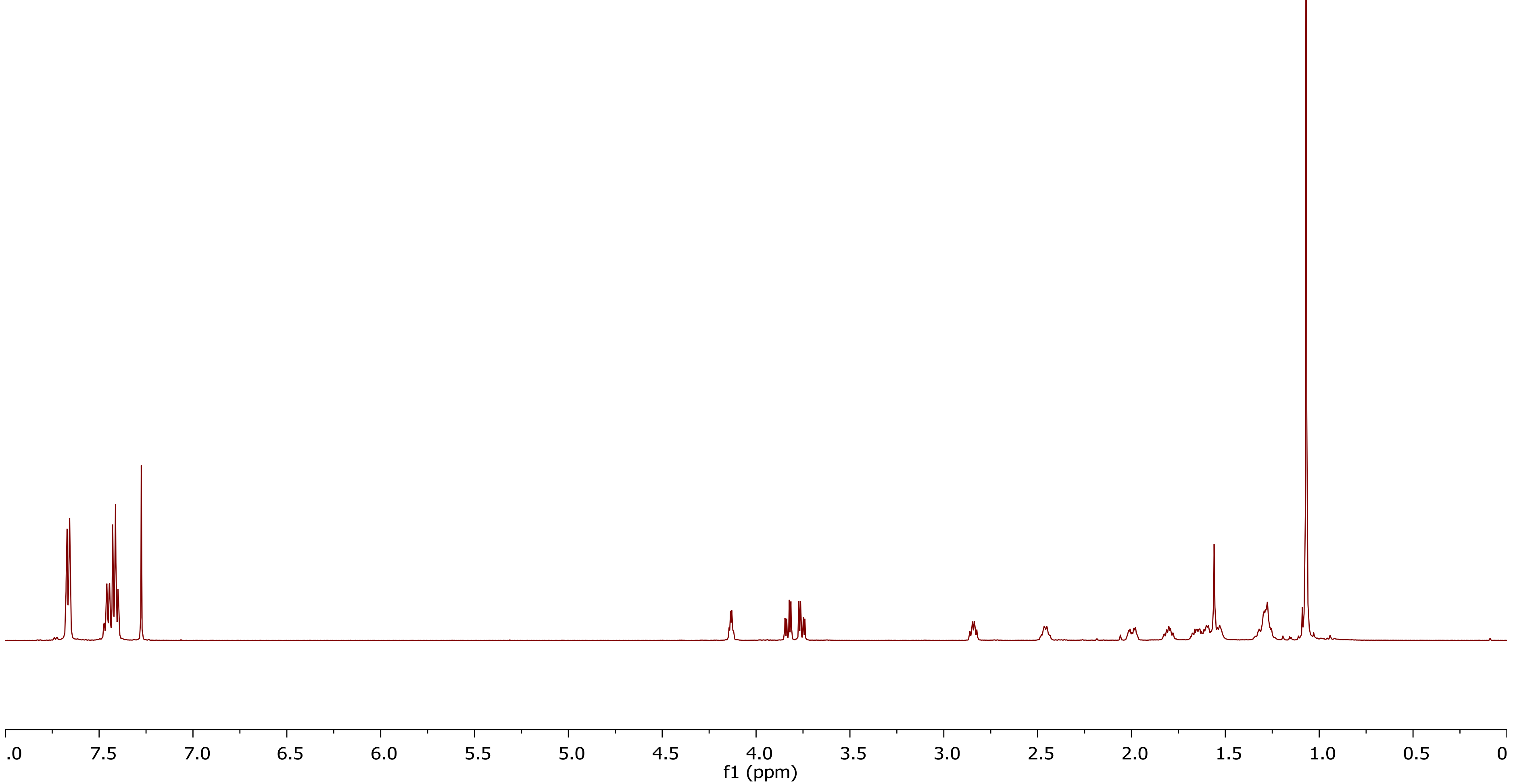

(3*S*,3a*S*,7a*R*)-3-((*tert*-Butyldiphenylsilyloxy)methyl)hexahydroisobenzofuran-1(3*H*)-one **23a**

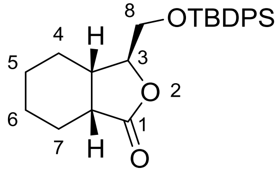

23a

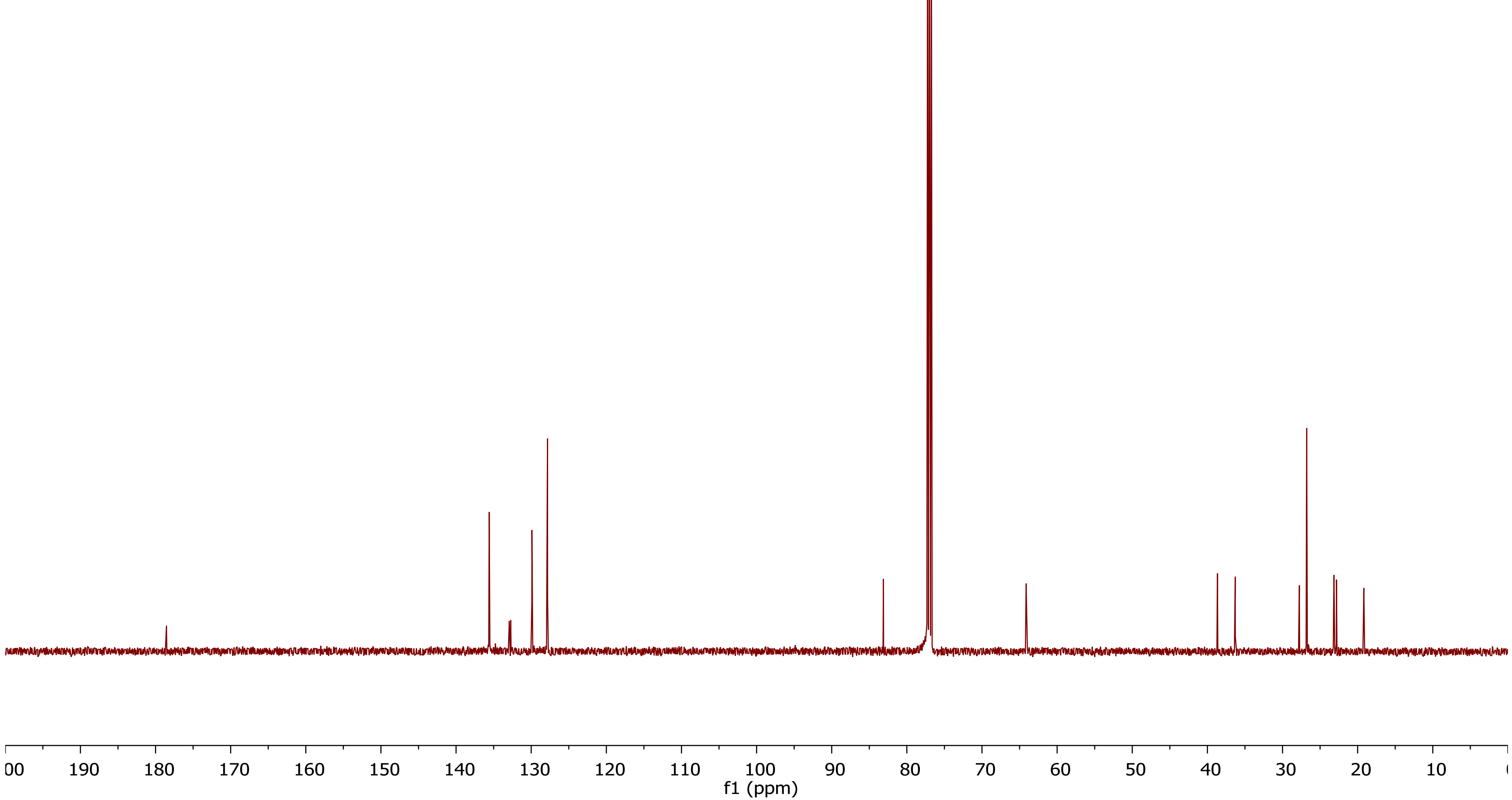

(3*S*,3a*S*,7a*R*)-3-((*tert*-Butyldiphenylsilyloxy)methyl)octahydroisobenzofuran-1-ol **23**

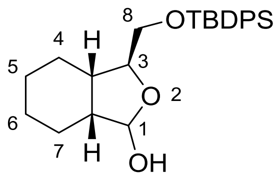

23

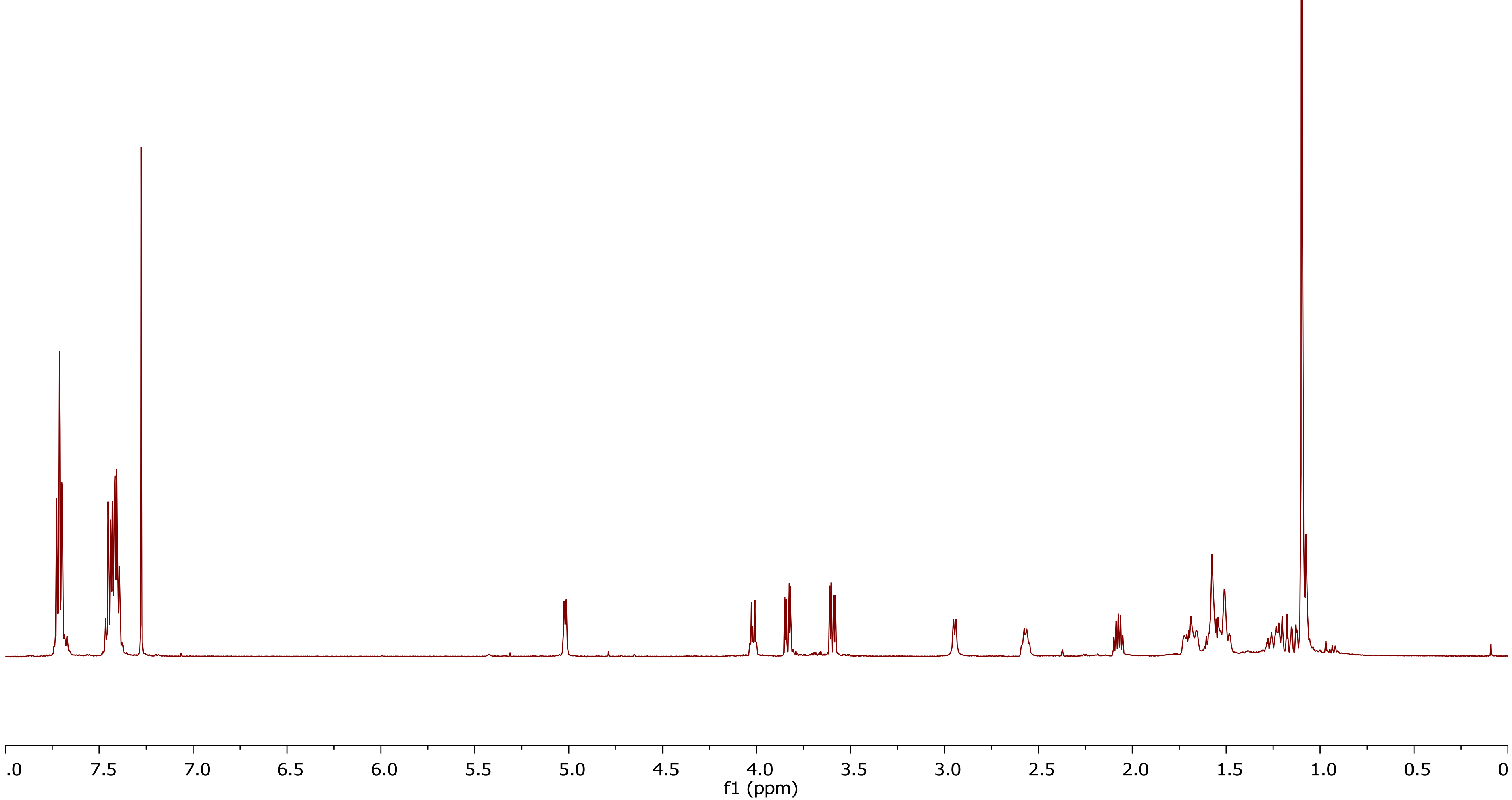

(3*S*,3a*S*,7a*R*)-3-((*tert*-Butyldiphenylsilyloxy)methyl)octahydroisobenzofuran-1-ol **23**

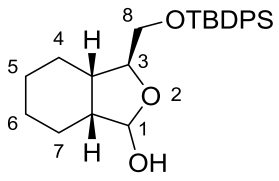

23

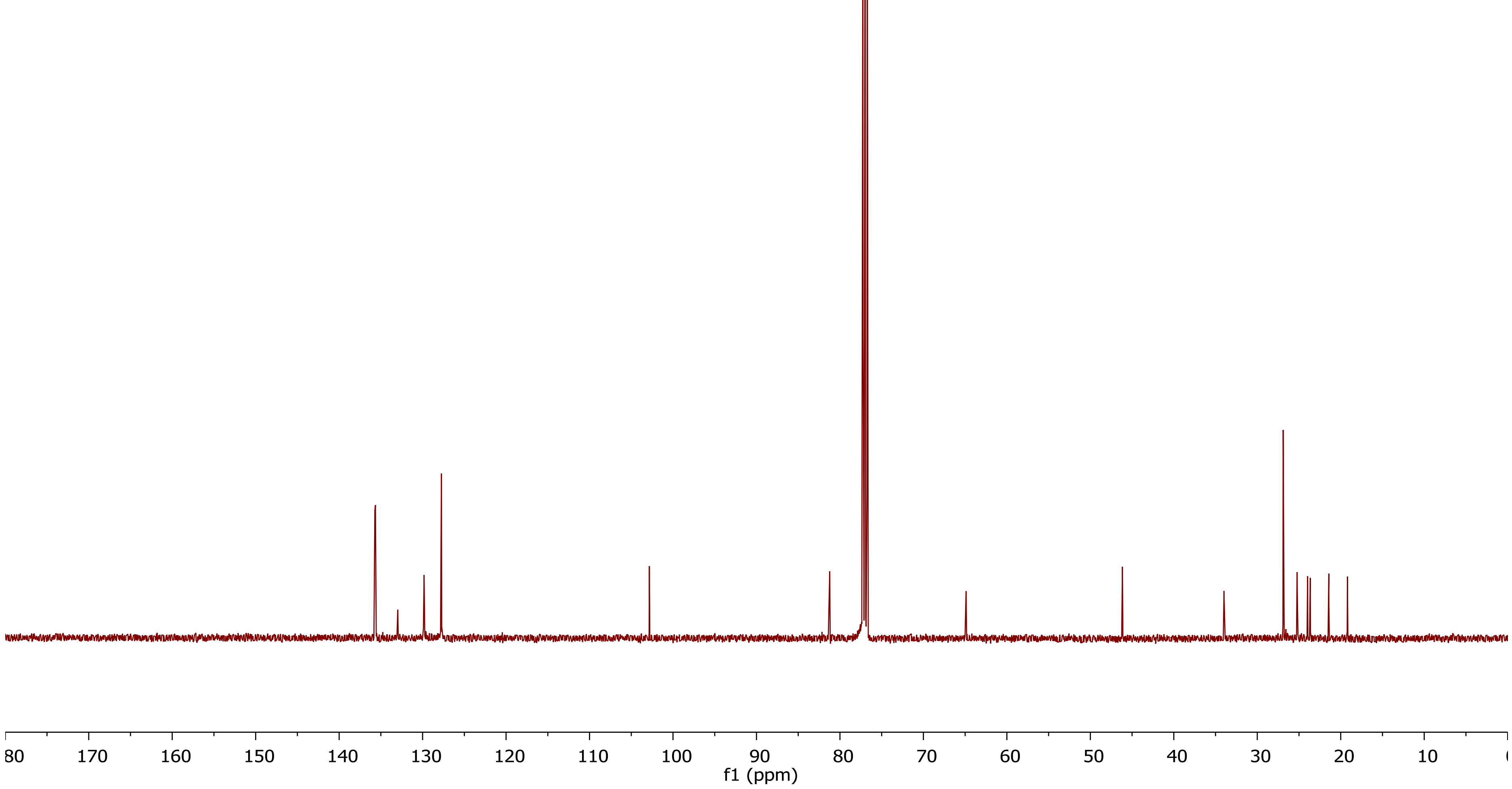

((1*S*,3*S*,3a*R*,7a*S*)-3-Allyl-octahydro-isobenzofuran-1-ylmethoxy)-*tert*-butyldiphenylsilane **24**

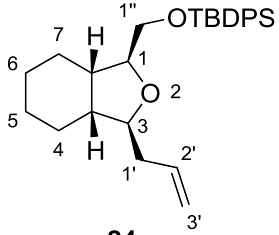

24

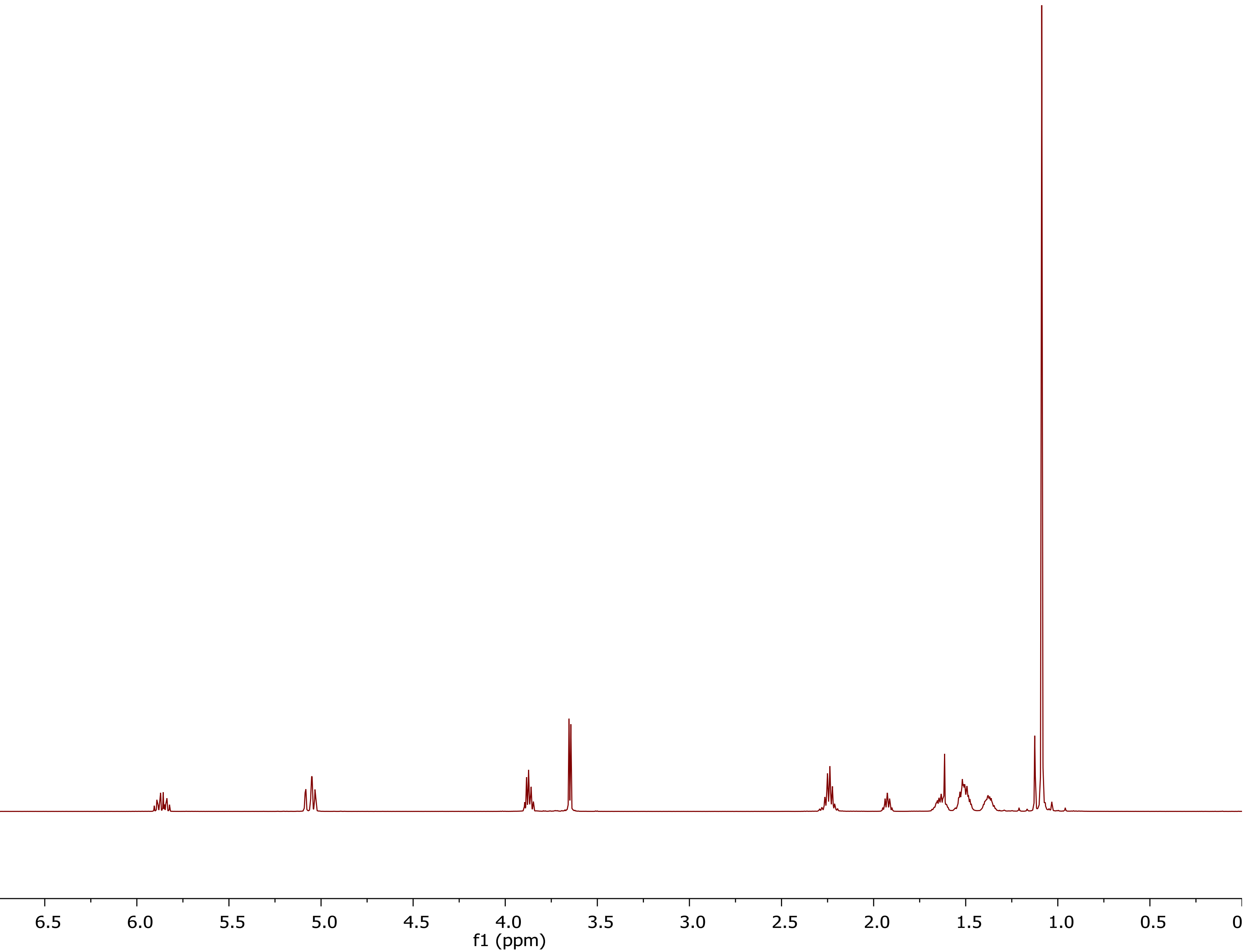

((1*S*,3*S*,3a*R*,7a*S*)-3-Allyl-octahydro-isobenzofuran-1-ylmethoxy)-*tert*-butyldiphenylsilane **24**

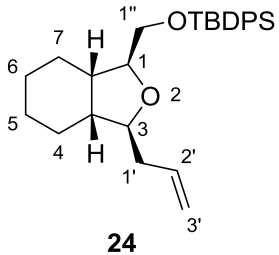

24

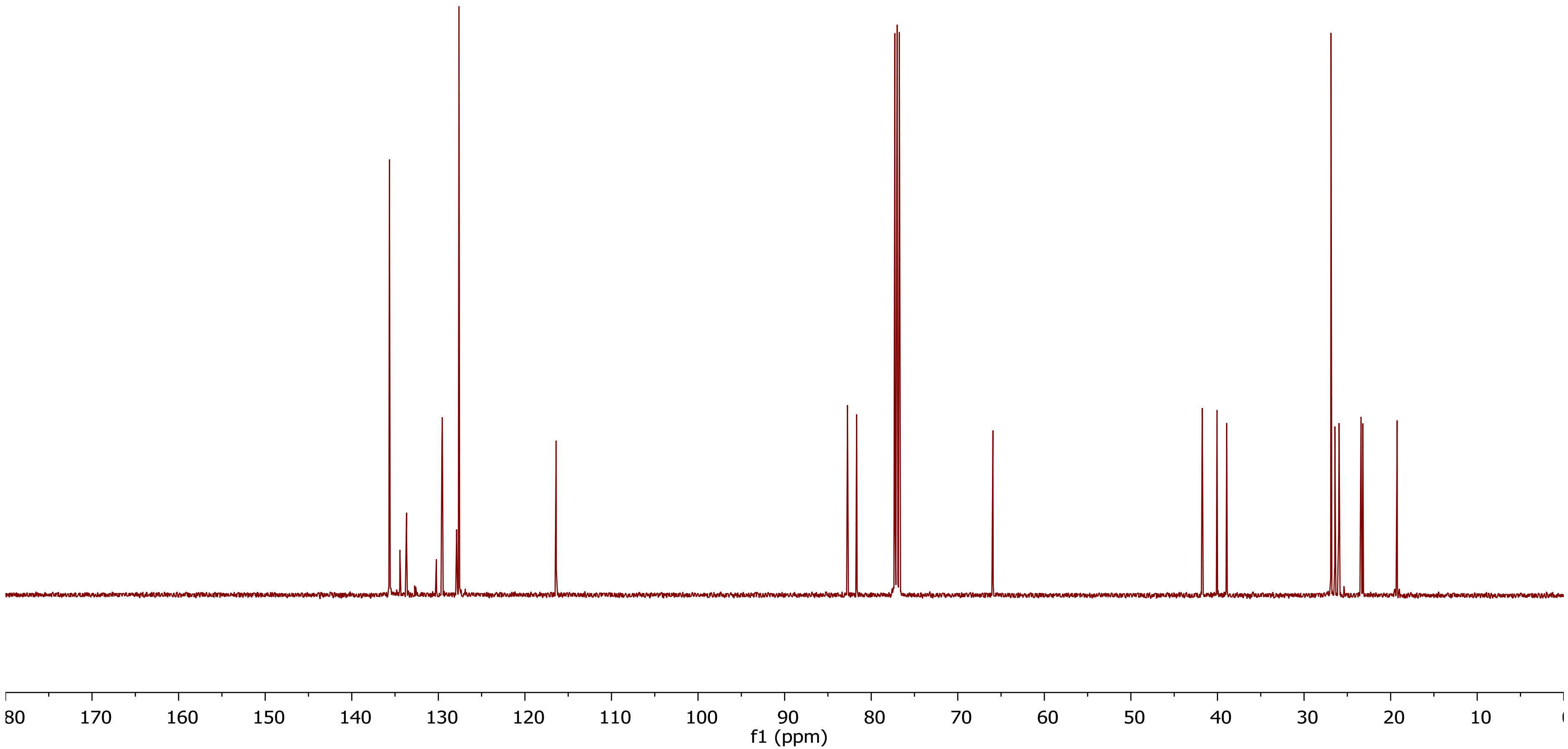

((1*S*,3*S*,3a*R*,7a*S*)-3-Allyl-octahydro-isobenzofuran-1-yl)-methanol **25**

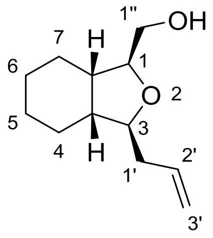

25

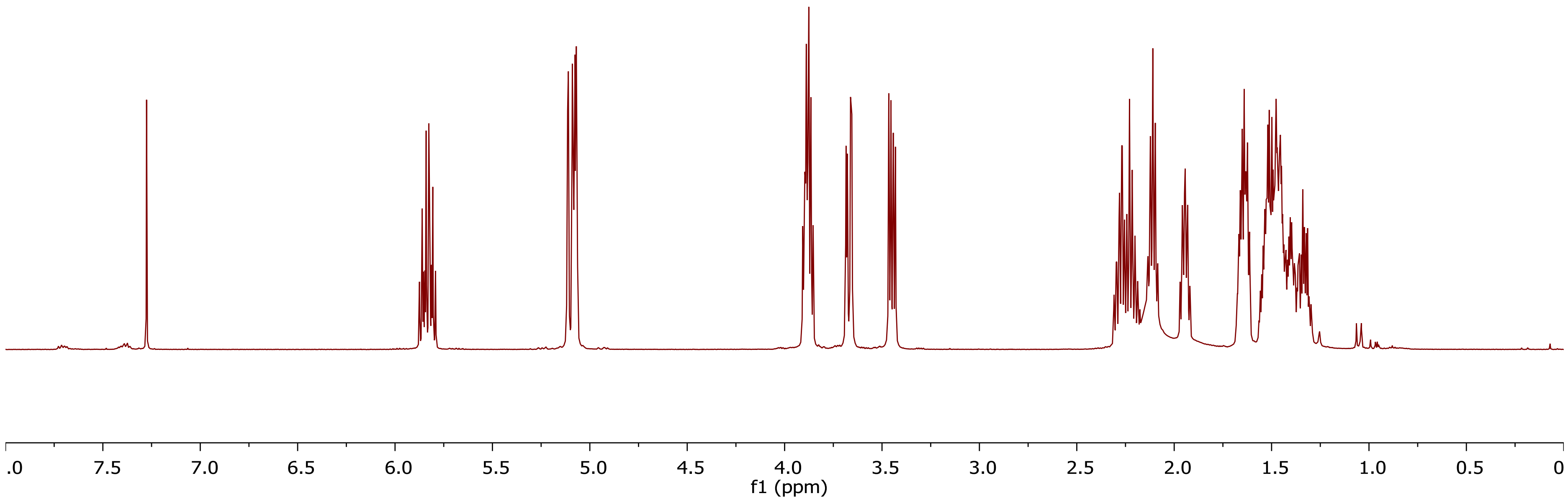

((1*S*,3*S*,3a*R*,7a*S*)-3-Allyl-octahydro-isobenzofuran-1-yl)-methanol **25**

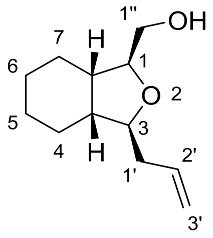

25

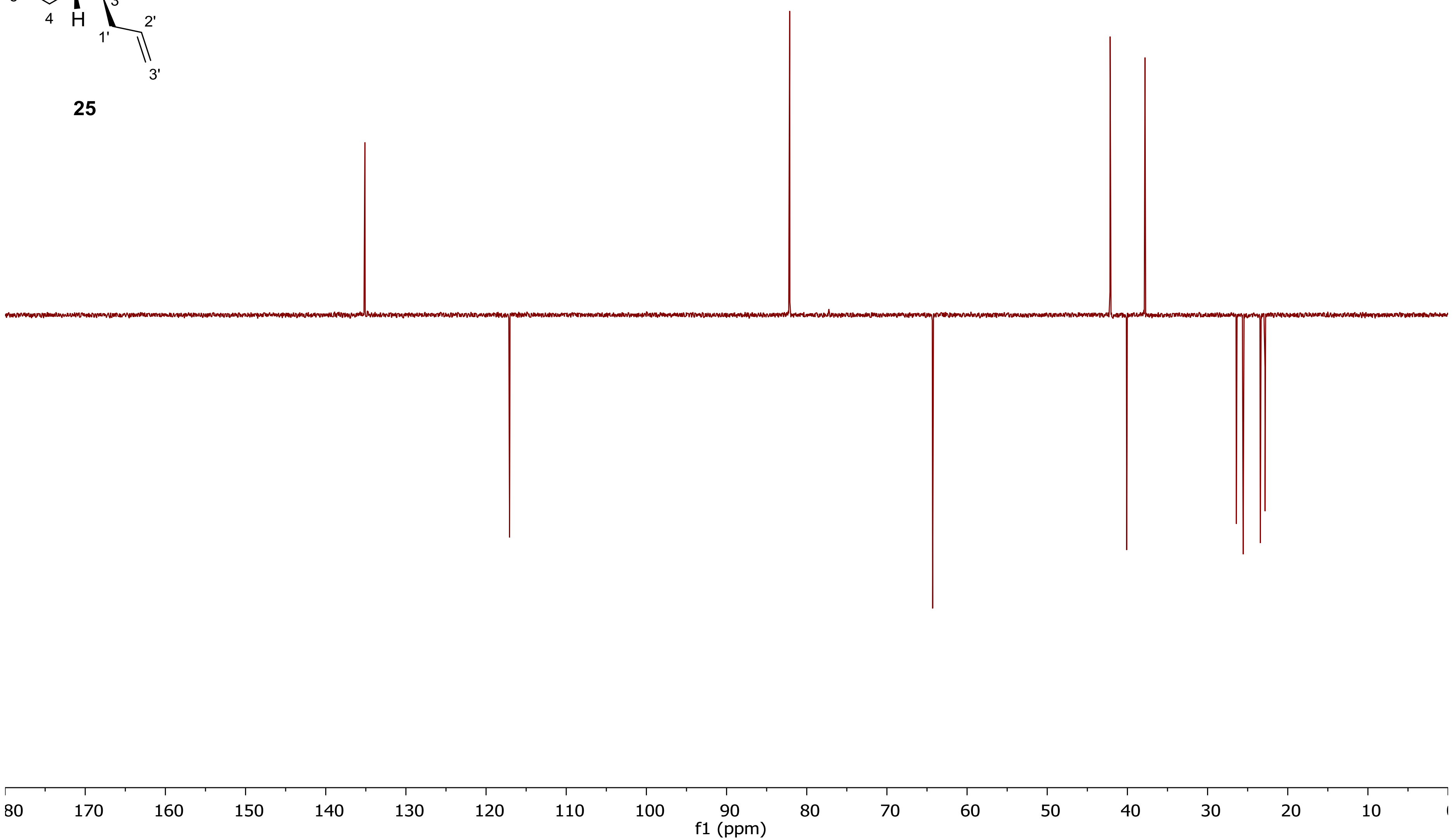

((1S,3S,3aR,7aS)-3-allyloctahydroisobenzofuran-1-yl)methyl pent-4-enoate **77**

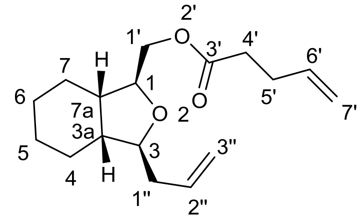

77

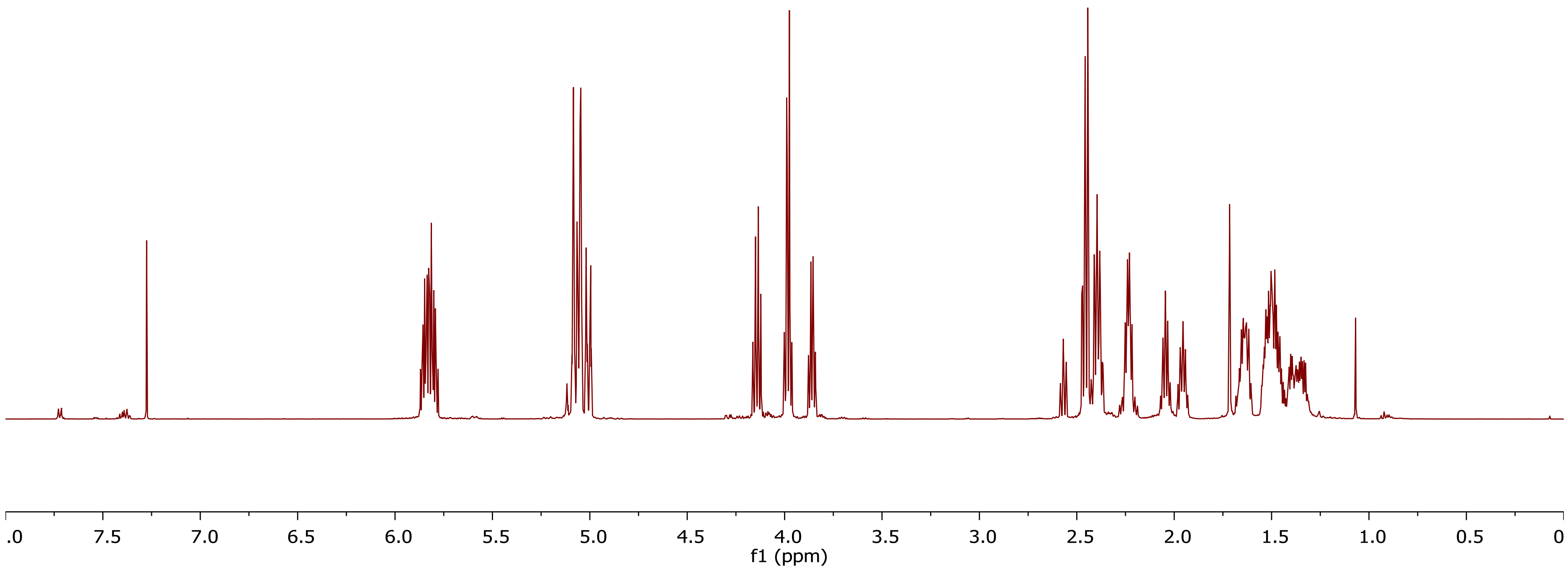

((1S,3S,3aR,7aS)-3-allyloctahydroisobenzofuran-1-yl)methyl pent-4-enoate **77**

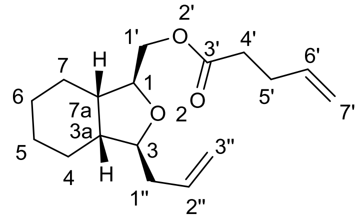

77

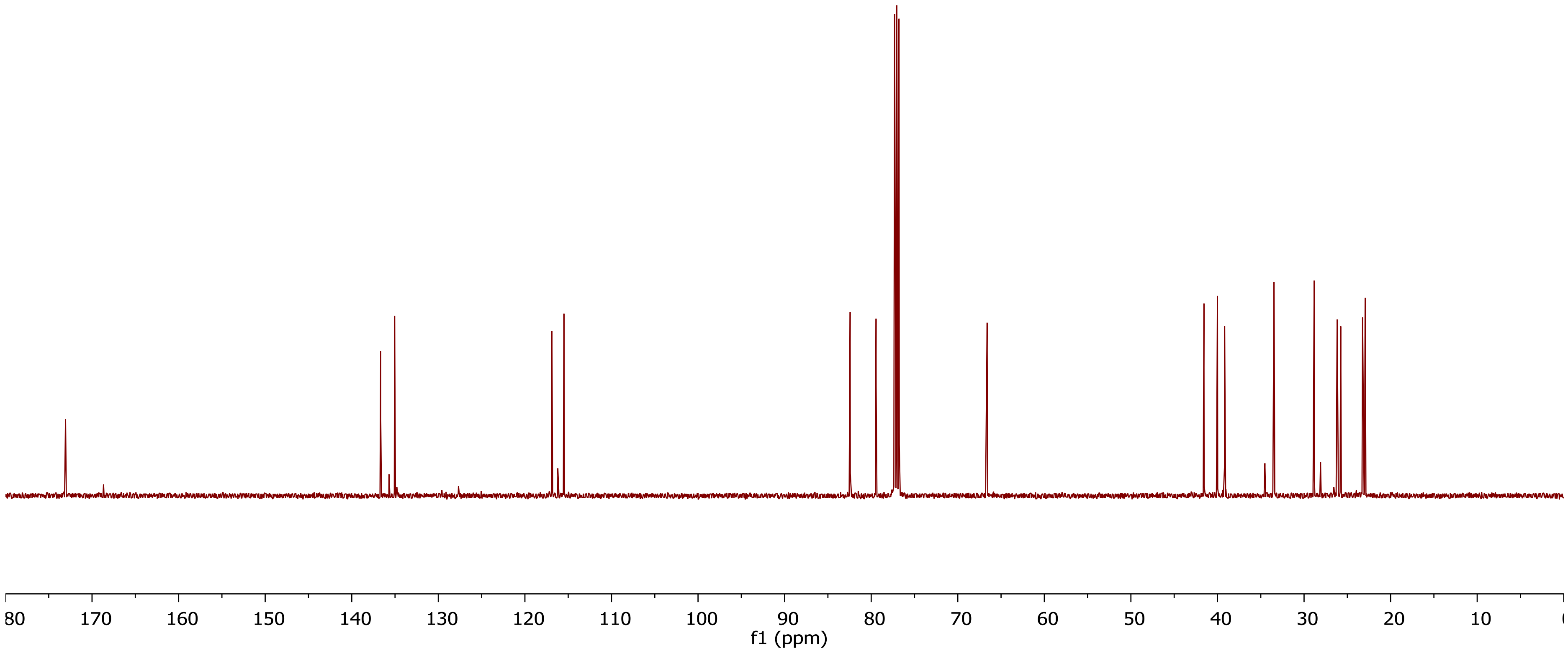

(Z)-(1*S*,10*S*,11*R*,16*S*)-3,17-Dioxatricyclo[8.6.1.0^{11,16}]heptadeca-7-en-4-one **26**

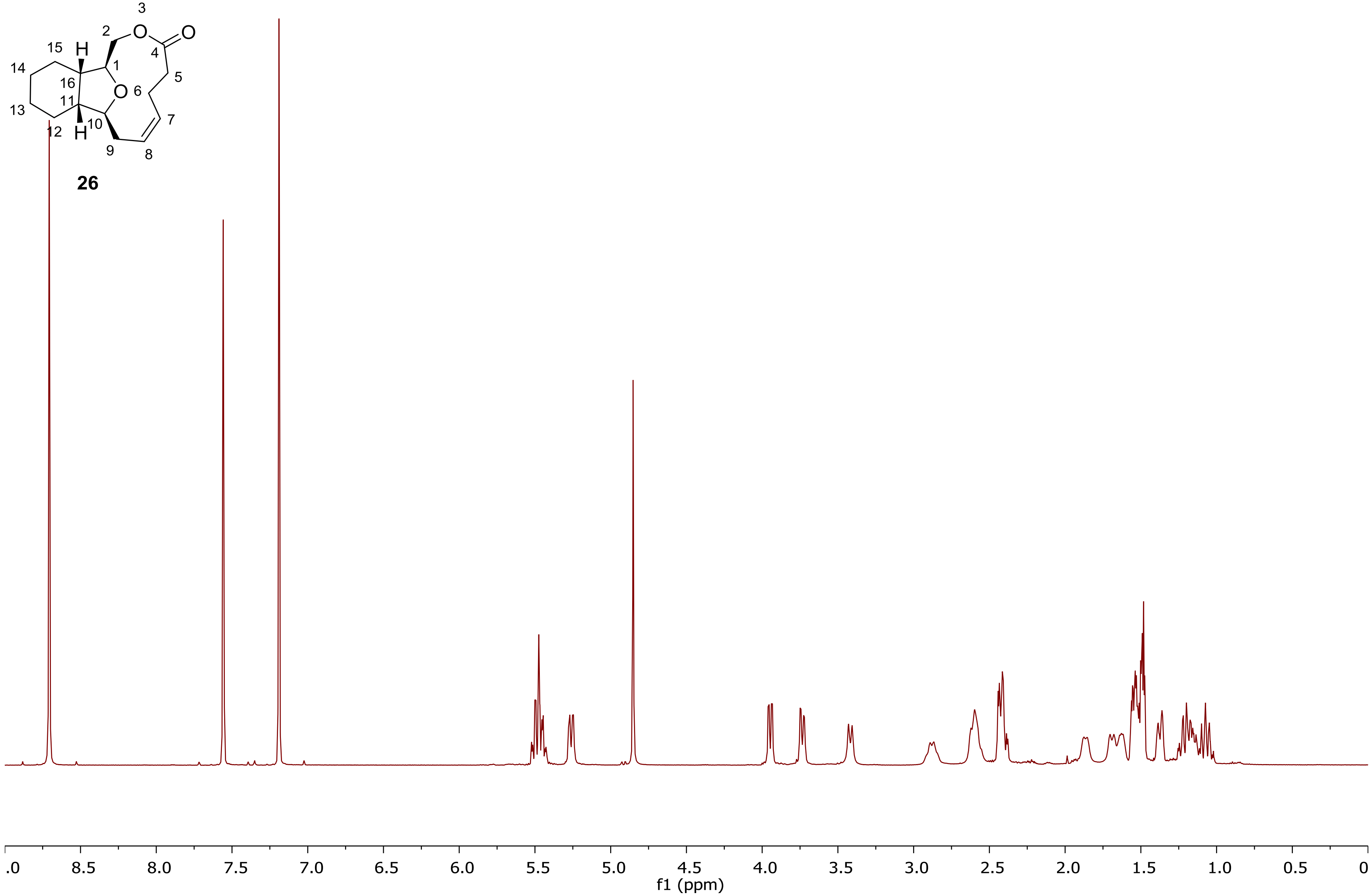

(Z)-(1*S*,10*S*,11*R*,16*S*)-3,17-Dioxatricyclo[8.6.1.0^{11,16}]heptadeca-7-en-4-one **26**

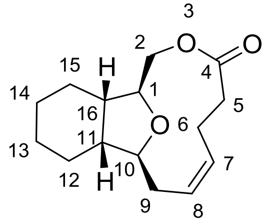

26

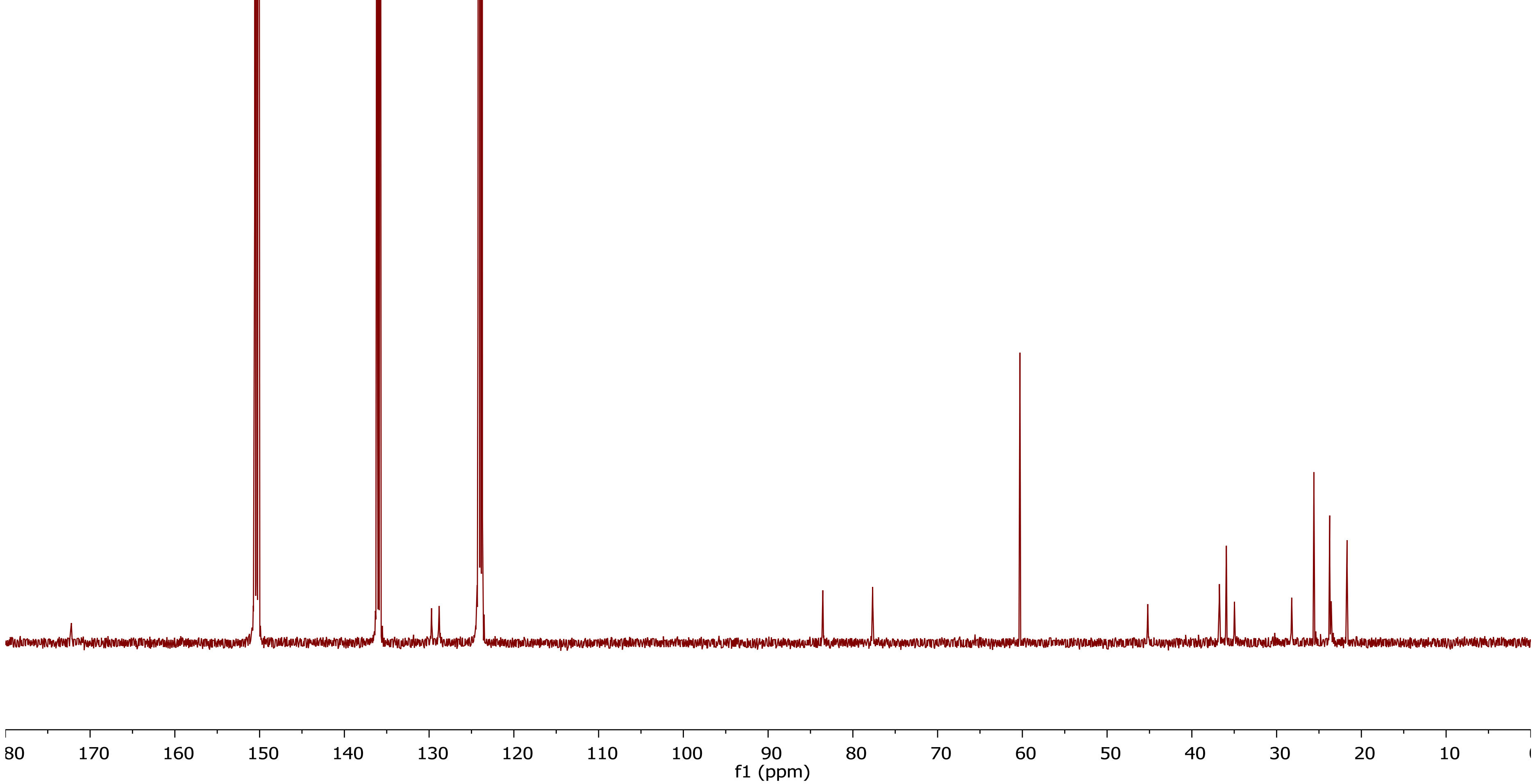

(E)-(1*S*,10*S*,11*R*,16*S*)-3,17-Dioxatricyclo[8.6.1.0^{11,16}]heptadeca-7-en-4-one **27**

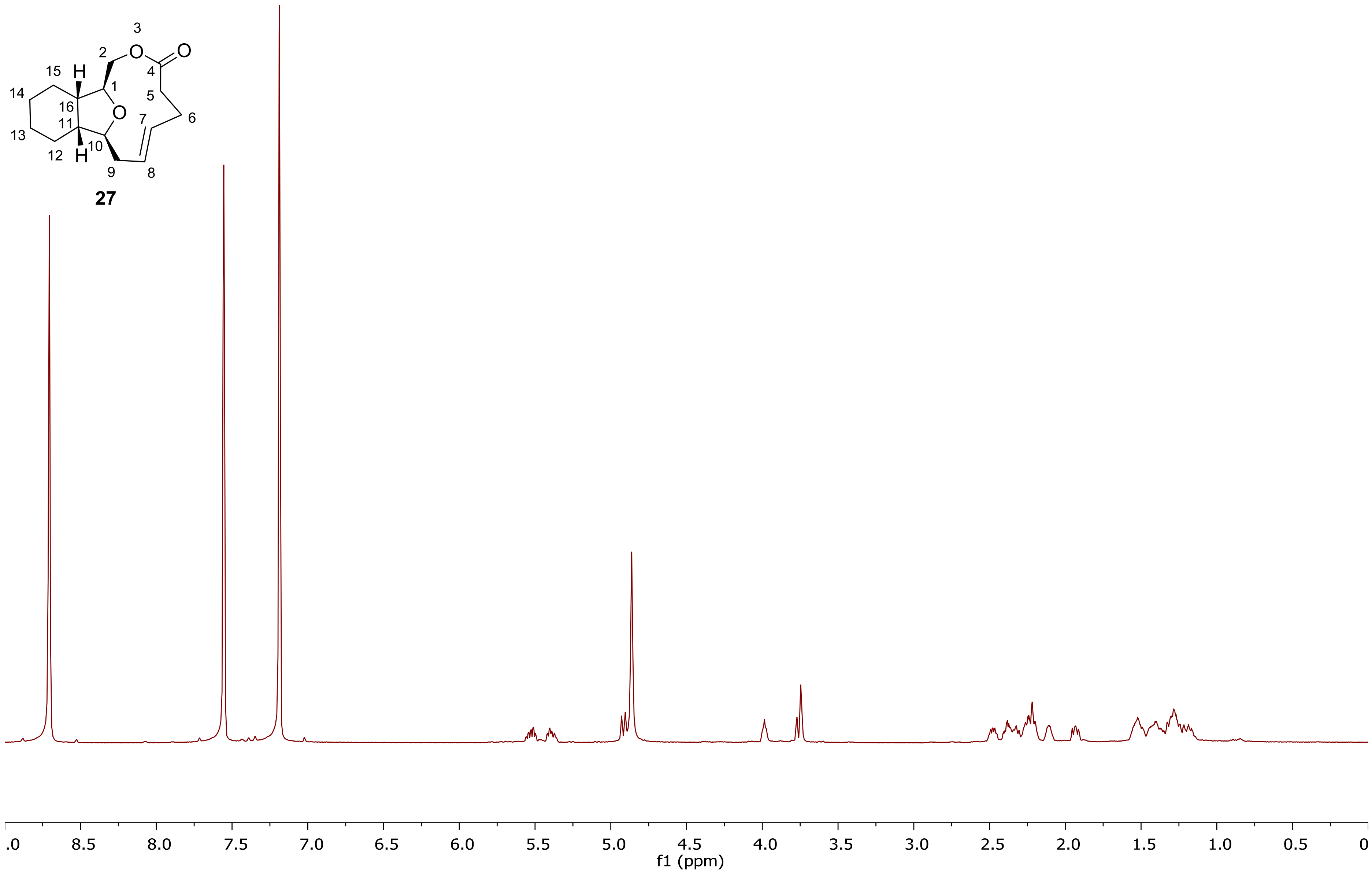

(E)-(1*S*,10*S*,11*R*,16*S*)-3,17-Dioxatricyclo[8.6.1.$0^{11,16}$]heptadeca-7-en-4-one **27**

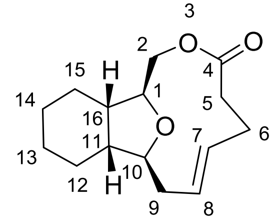

27

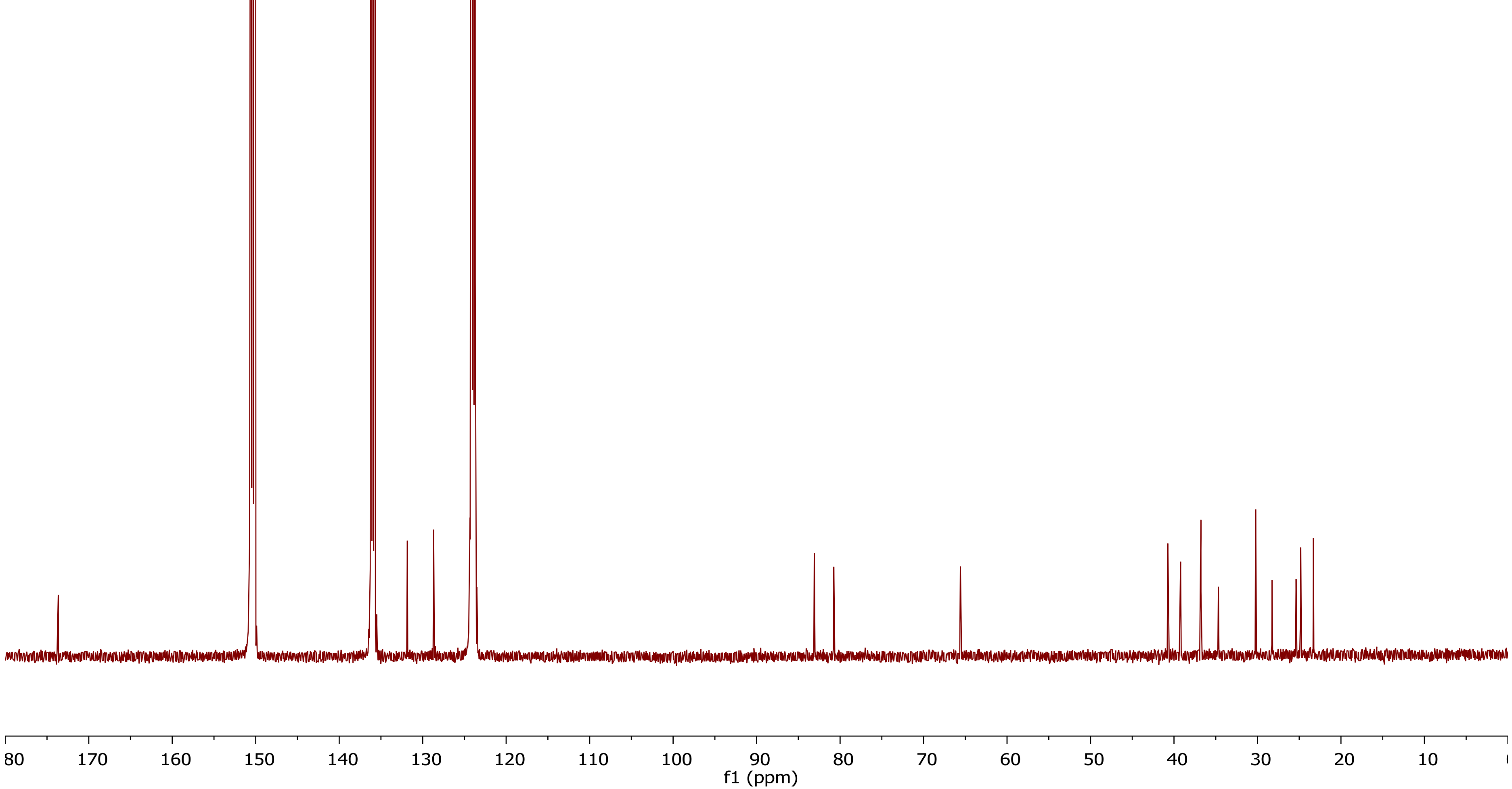

((1S,3S,3aR,7aS)-3-allyloctahydroisobenzofuran-1-yl)methyl hex-5-enoate **78**

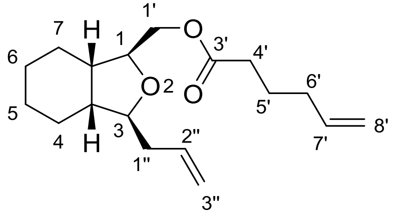

78

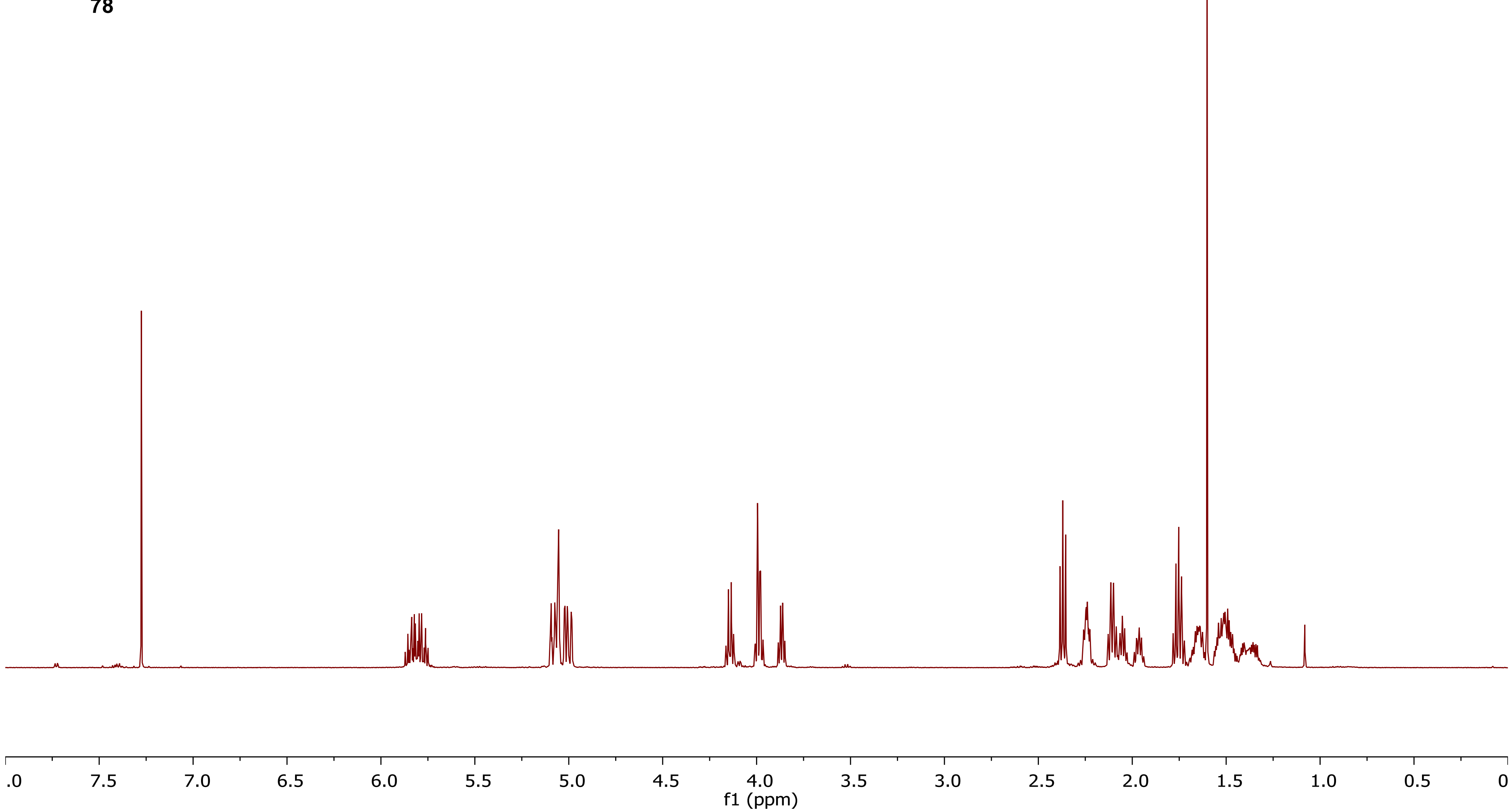

((1S,3S,3aR,7aS)-3-allyloctahydroisobenzofuran-1-yl)methyl hex-5-enoate **78**

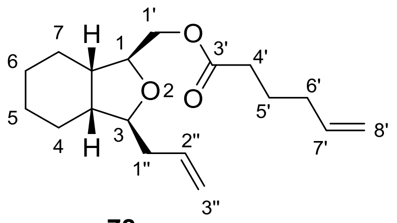

78

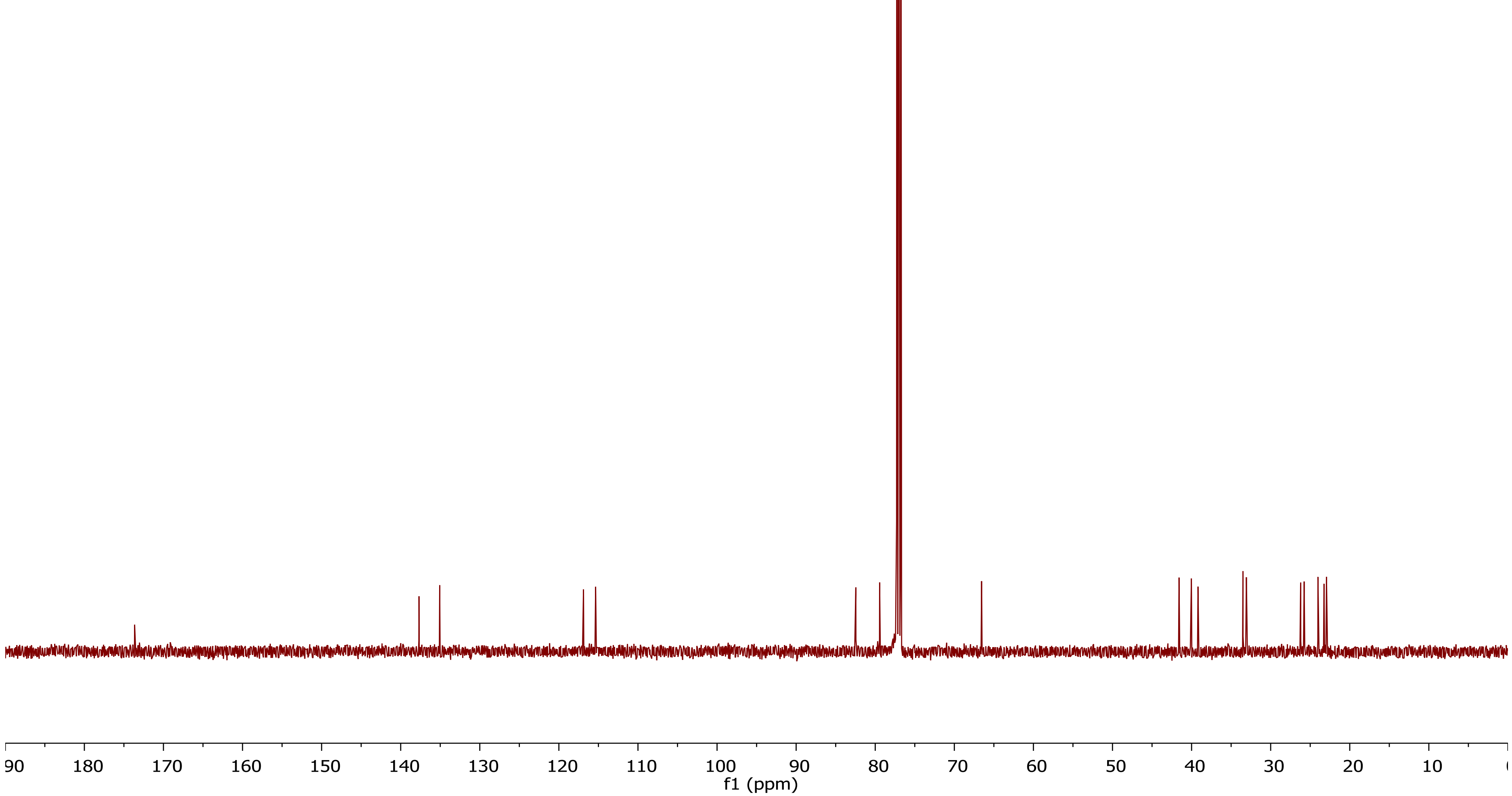

(Z)-(1S,11S,12R,17S)-3,18-Dioxatricyclo[9.6.1.0^{12,17}]octadec-8-en-4-one **28**

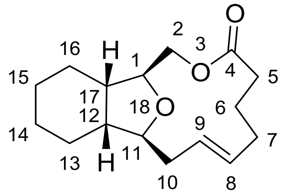

28

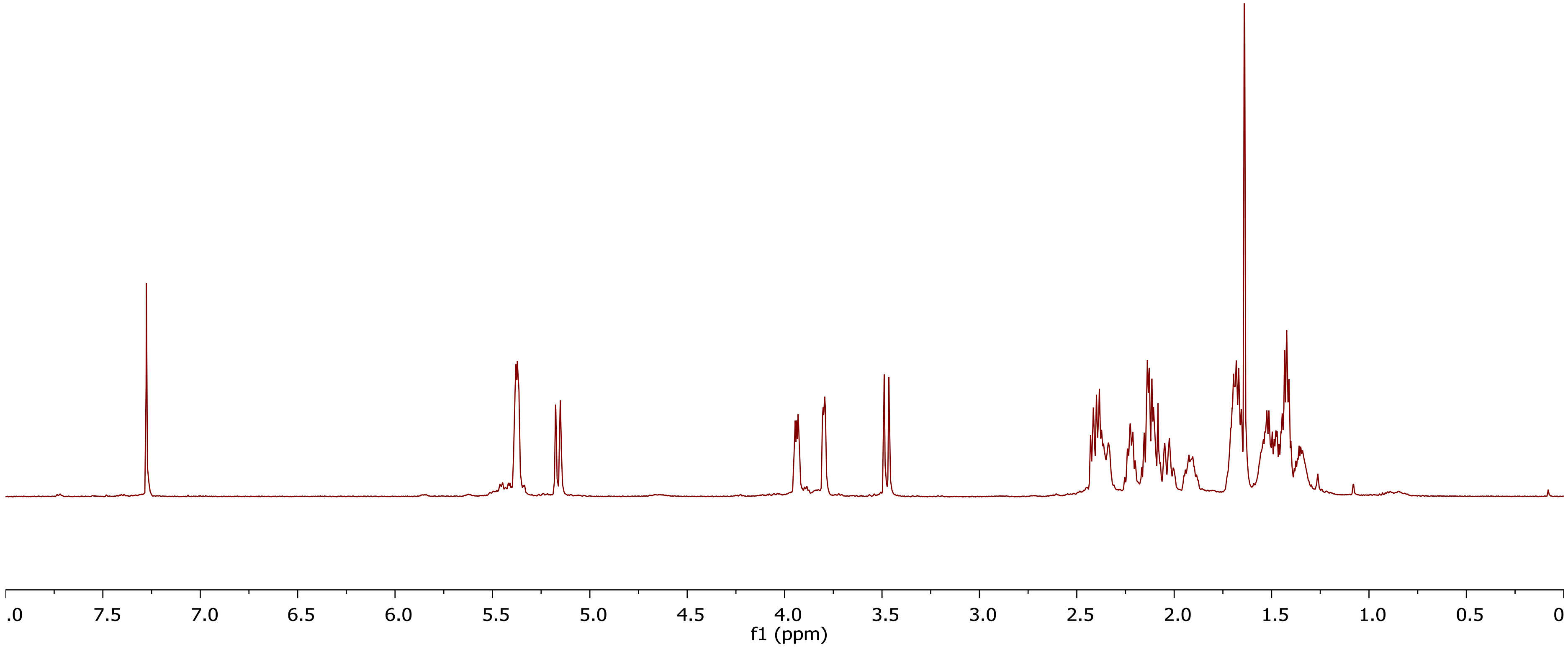

(Z)-(1S,11S,12R,17S)-3,18-Dioxatricyclo[9.6.1.0^{12,17}]octadec-8-en-4-one **28**

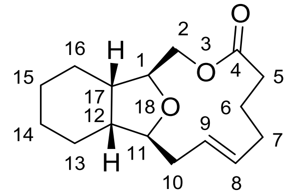

28

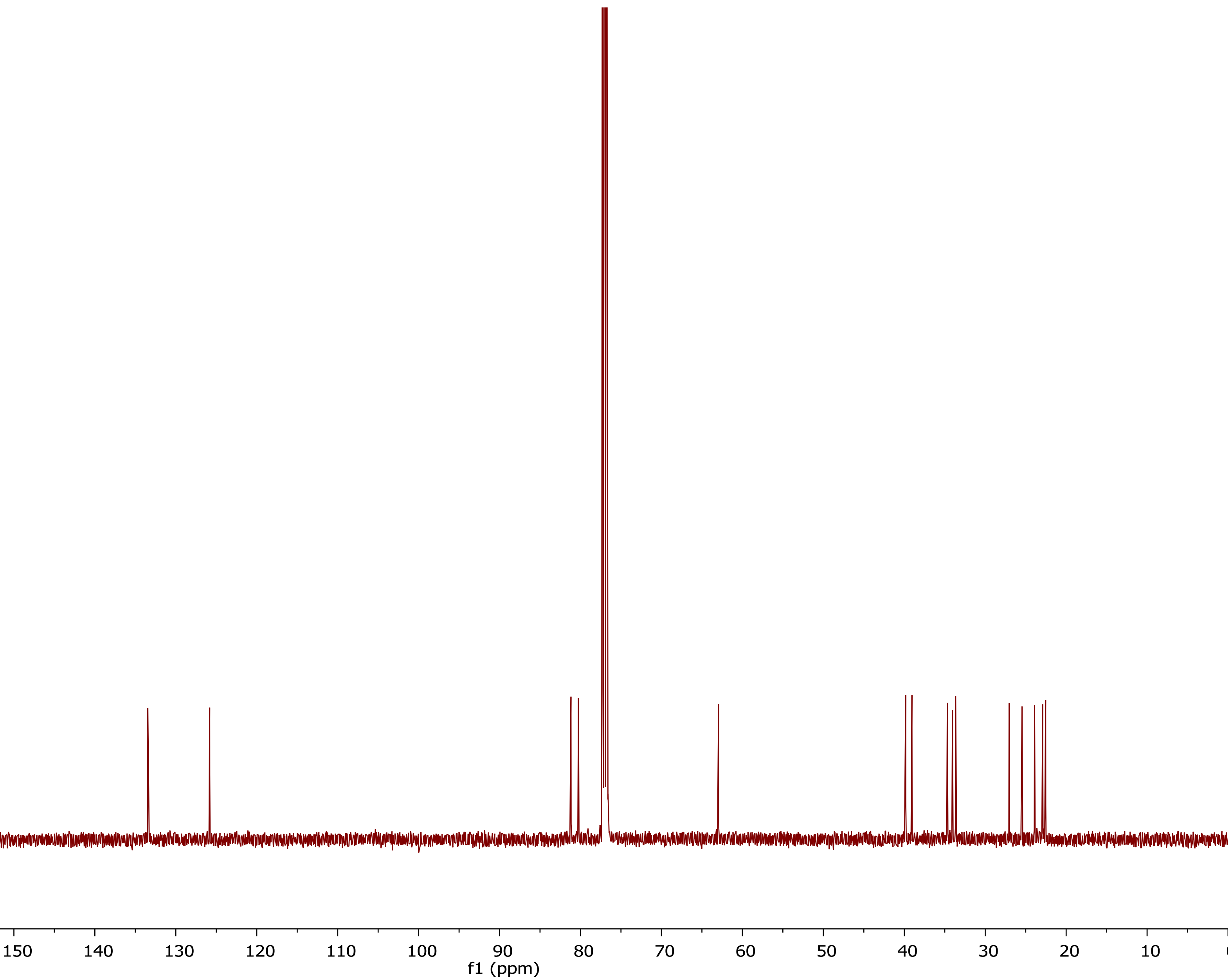

(1*S*,3*S*,3a*S*,7a*R*)-1-Allyl-3-(allyloxymethyl)-1,3,3a,4,7,7a-hexahydroisobenzofuran **29**

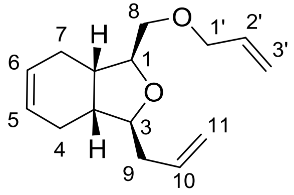

29

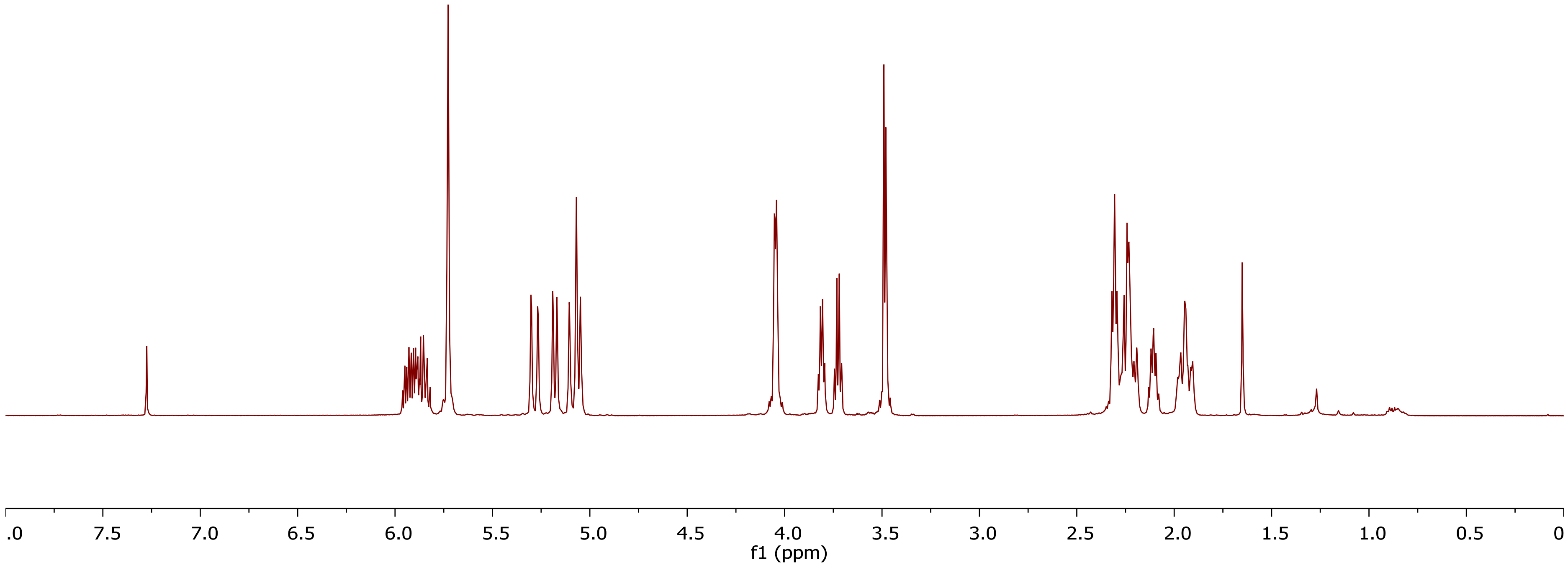

(1*S*,3*S*,3a*S*,7a*R*)-1-Allyl-3-(allyloxymethyl)-1,3,3a,4,7,7a-hexahydroisobenzofuran **29**

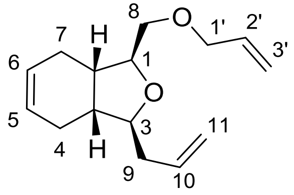

29

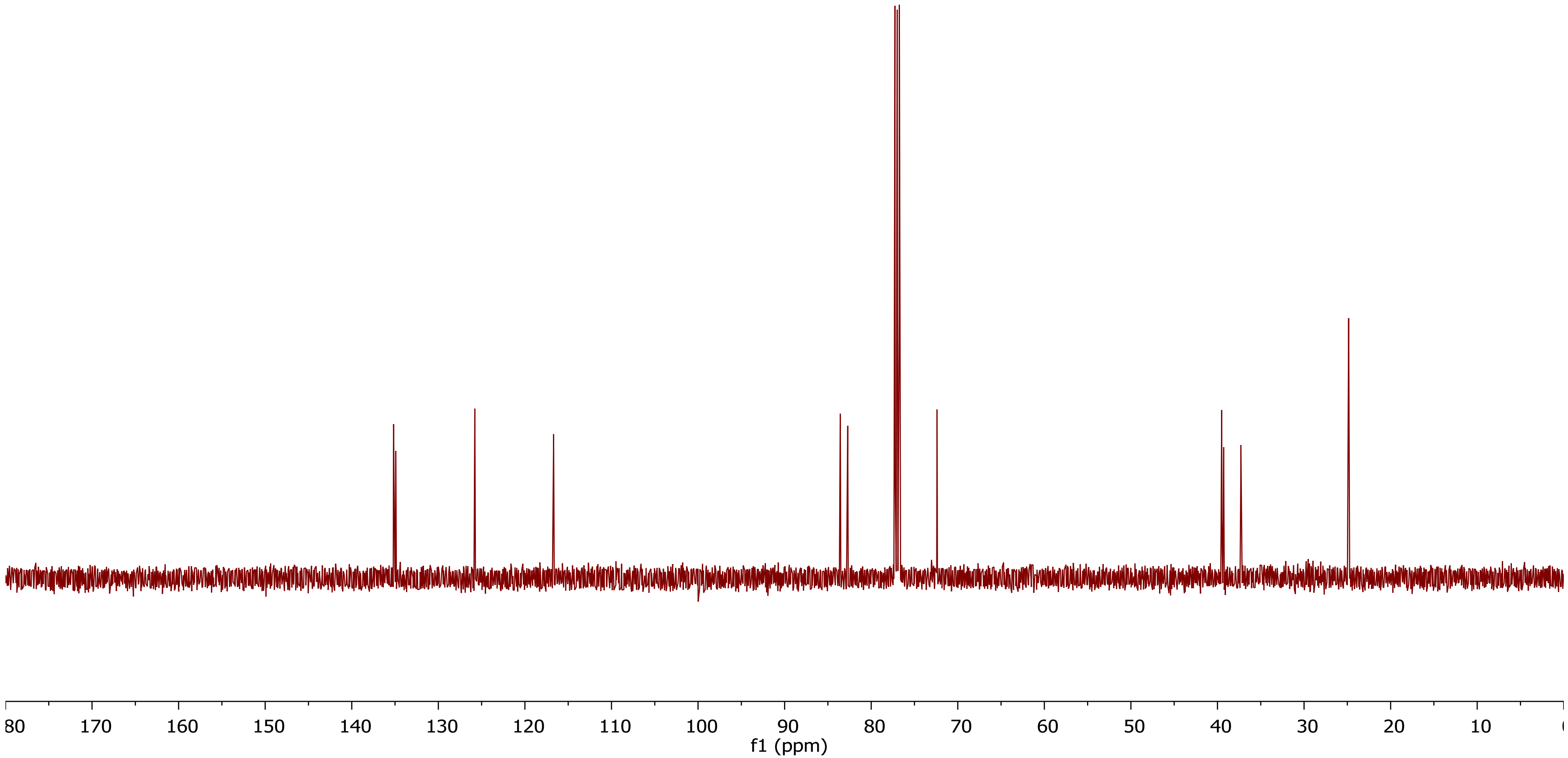

(3aR,4aR,7S,7aS,8aS)-7-(*tert*-Butyldiphenylsilanyloxymethyl)-2,2-dimethyl-hexahydro-furo[3',4':4,5]benzo[1,2-d][1,3]dioxol-5-one **31**

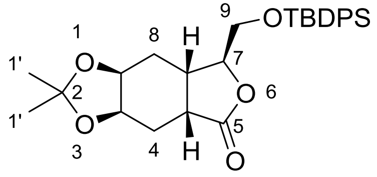

31

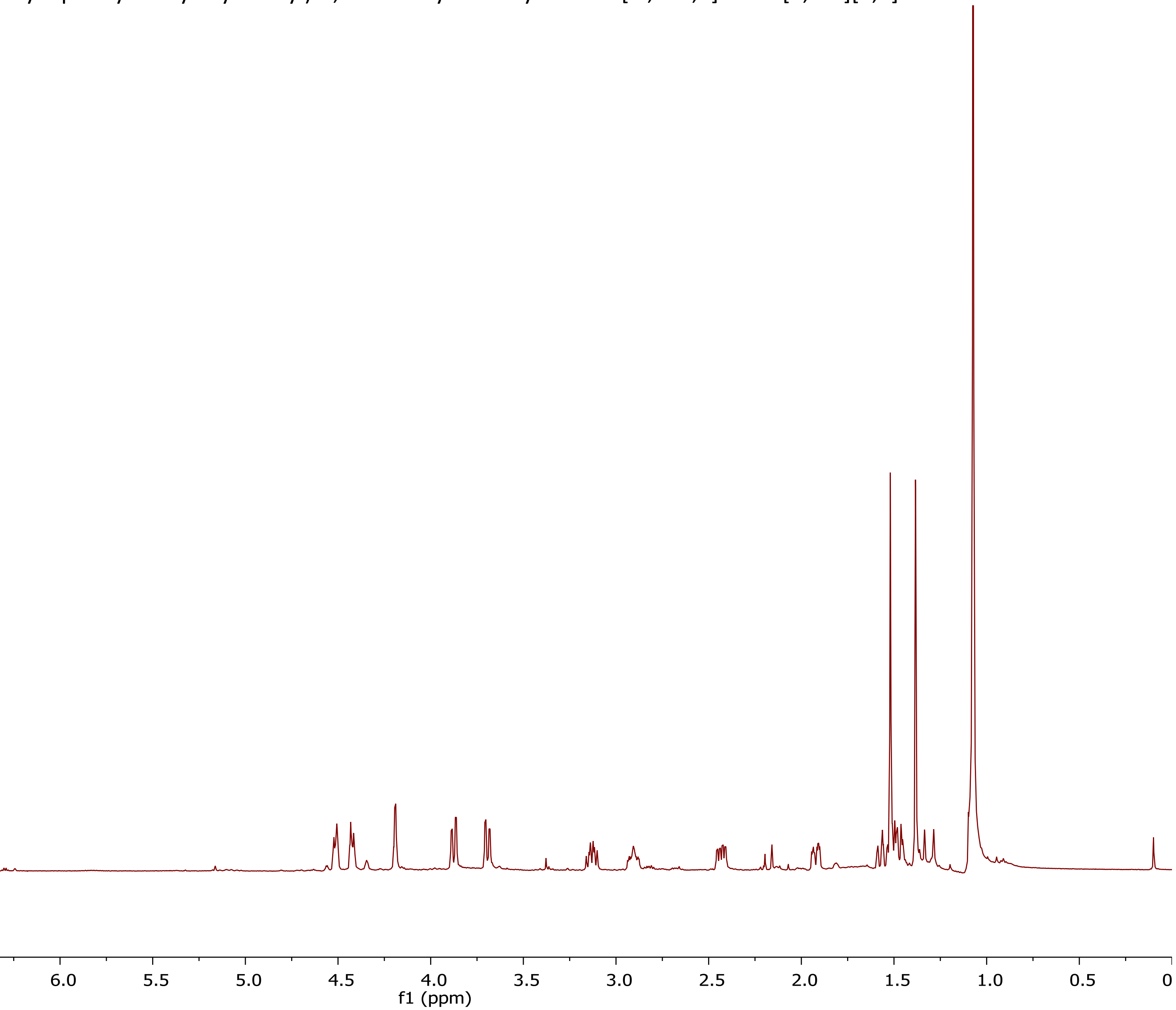

(3aR,4aR,7S,7aS,8aS)-7-(*tert*-Butyldiphenylsilanyloxymethyl)-2,2-dimethyl-hexahydro-furo[3',4':4,5]benzo[1,2-d][1,3]dioxol-5-one **31**

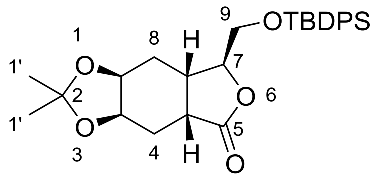

31

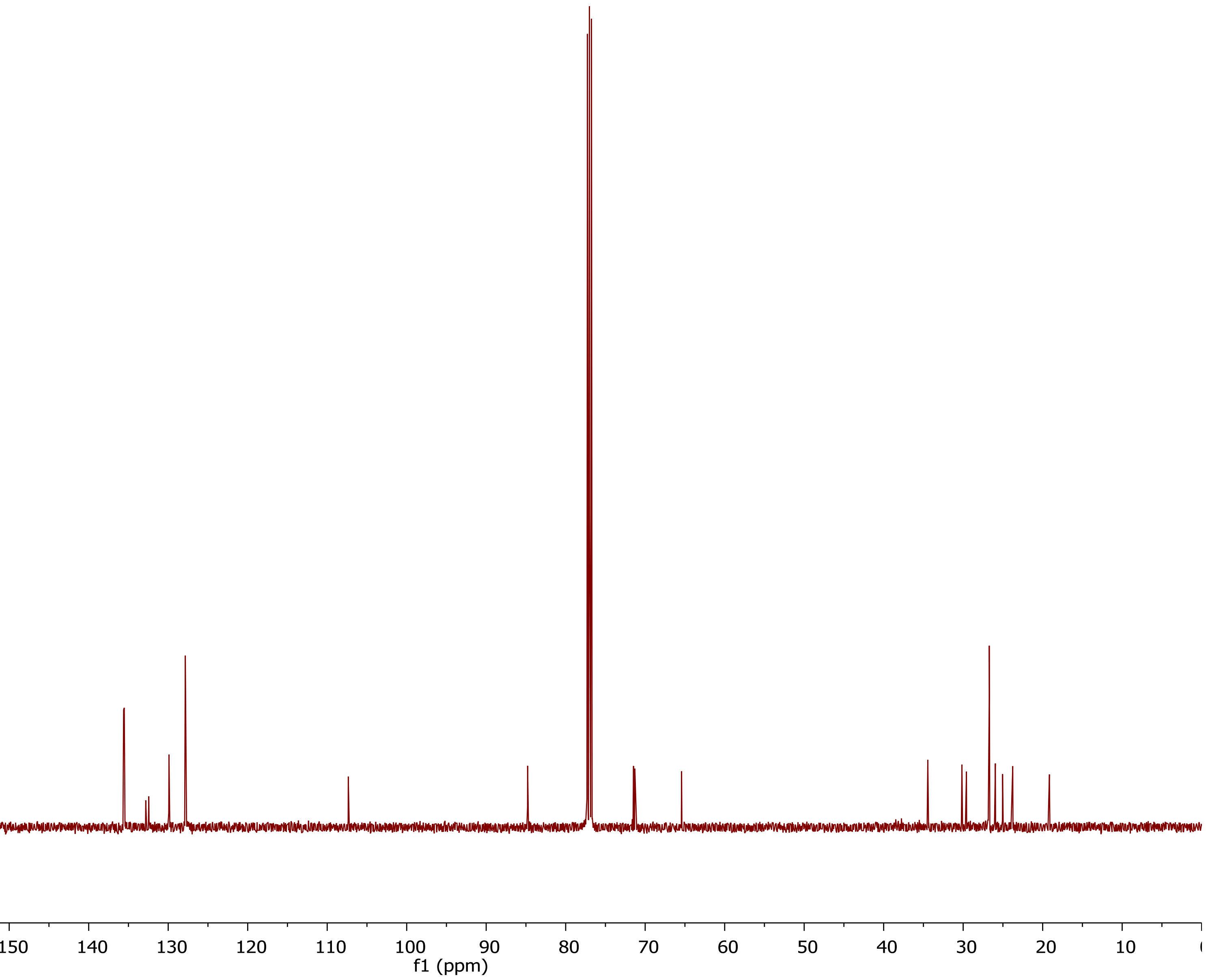

(3aR,4aR,7S,7aS,8aS)-7-(*tert*-Butyldiphenylsilanyloxymethyl)-2,2-dimethyl-hexahydro-furo[3',4':4,5]benzo[1,2-d][1,3]dioxol-5-one **31**

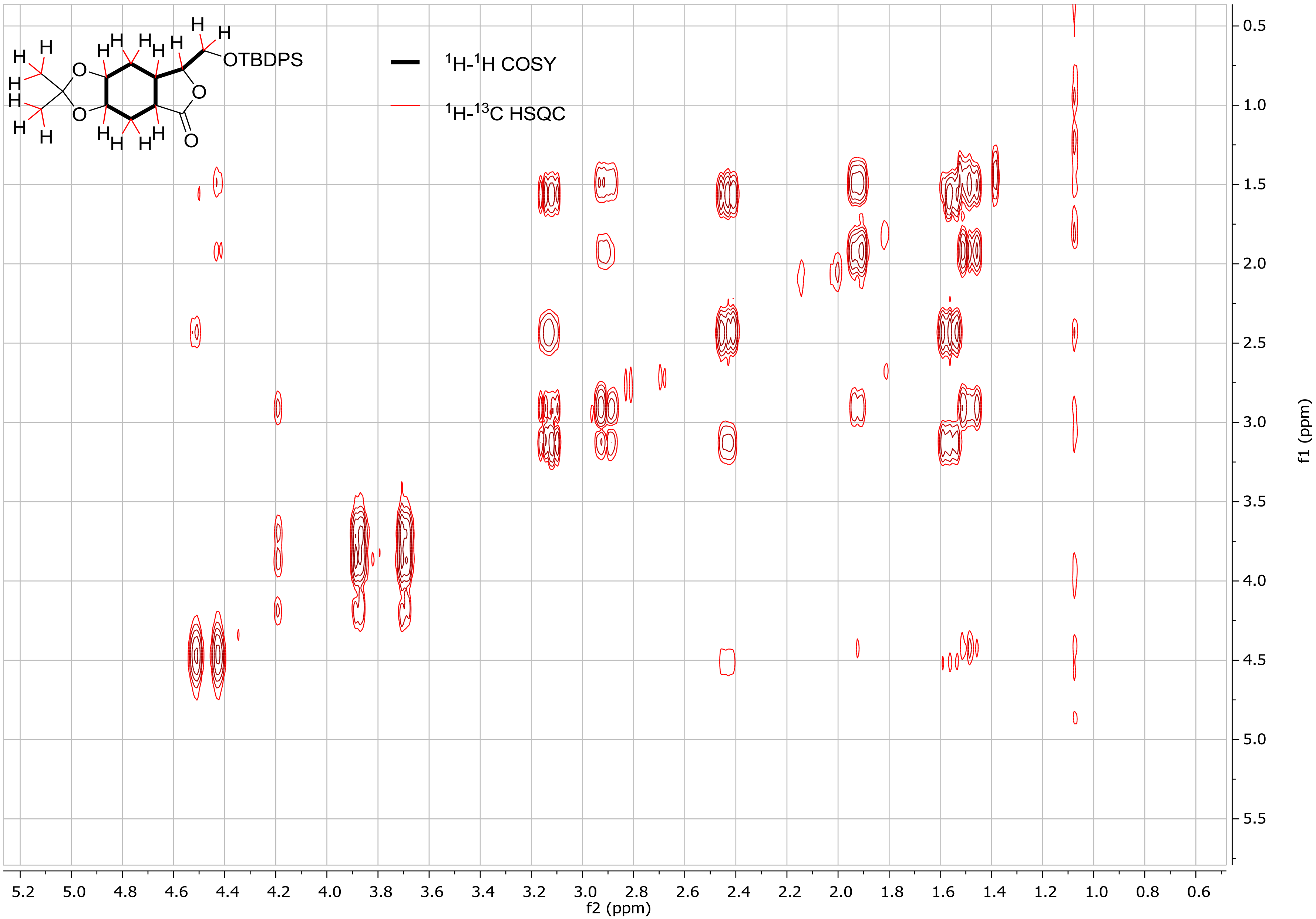

(3aR,4aR,7S,7aS,8aS)-7-(*tert*-Butyldiphenylsilanyloxymethyl)-2,2-dimethyl-hexahydro-furo[3',4':4,5]benzo[1,2-d][1,3]dioxol-5-one **31**

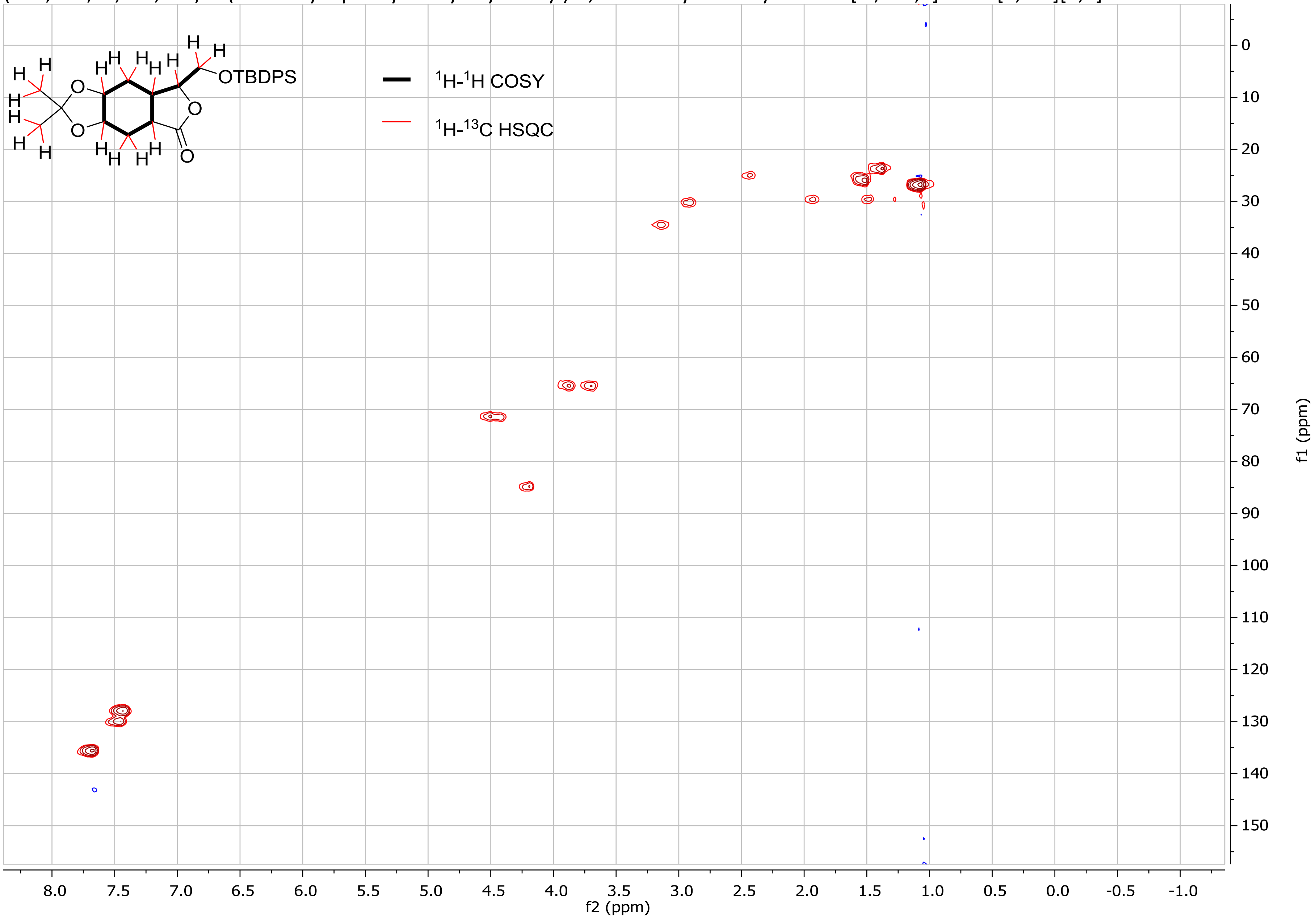

(3aR,4aR,7S,7aS,8aS)-7-(*tert*-Butyldiphenylsilanyloxymethyl)-2,2-dimethyloctahydrofuro [3',4':4,5]benzo[1,2-*d*][1,3]dioxol-5-ol **32**

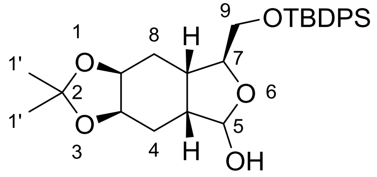

32

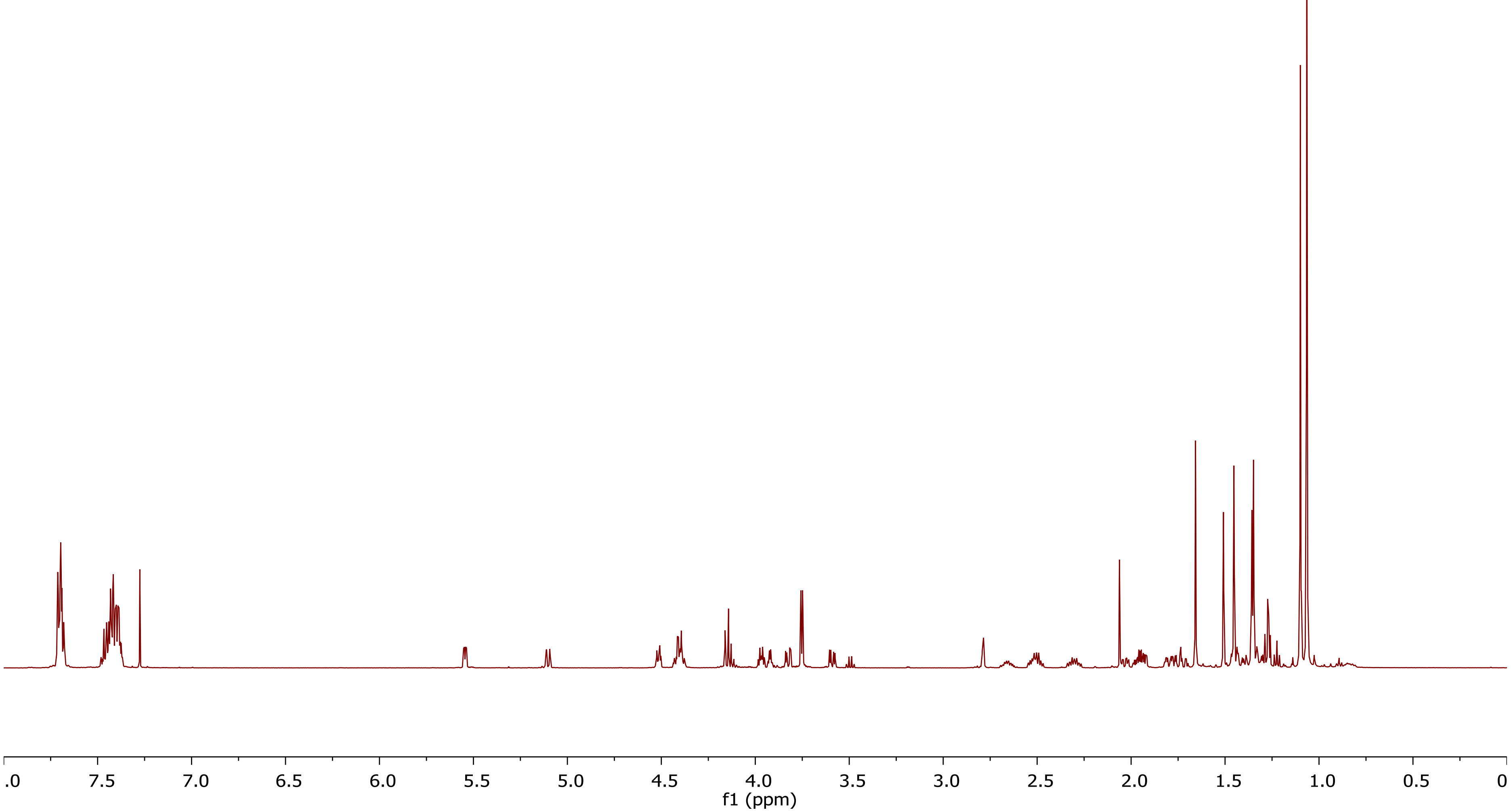

(3aR,4aR,7S,7aS,8aS)-7-(*tert*-Butyldiphenylsilanyloxymethyl)-2,2-dimethyloctahydrofuro [3',4':4,5]benzo[1,2-*d*][1,3]dioxol-5-ol **32**

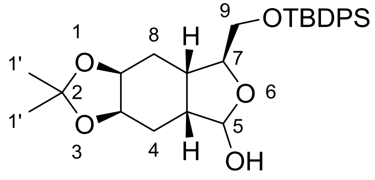

32

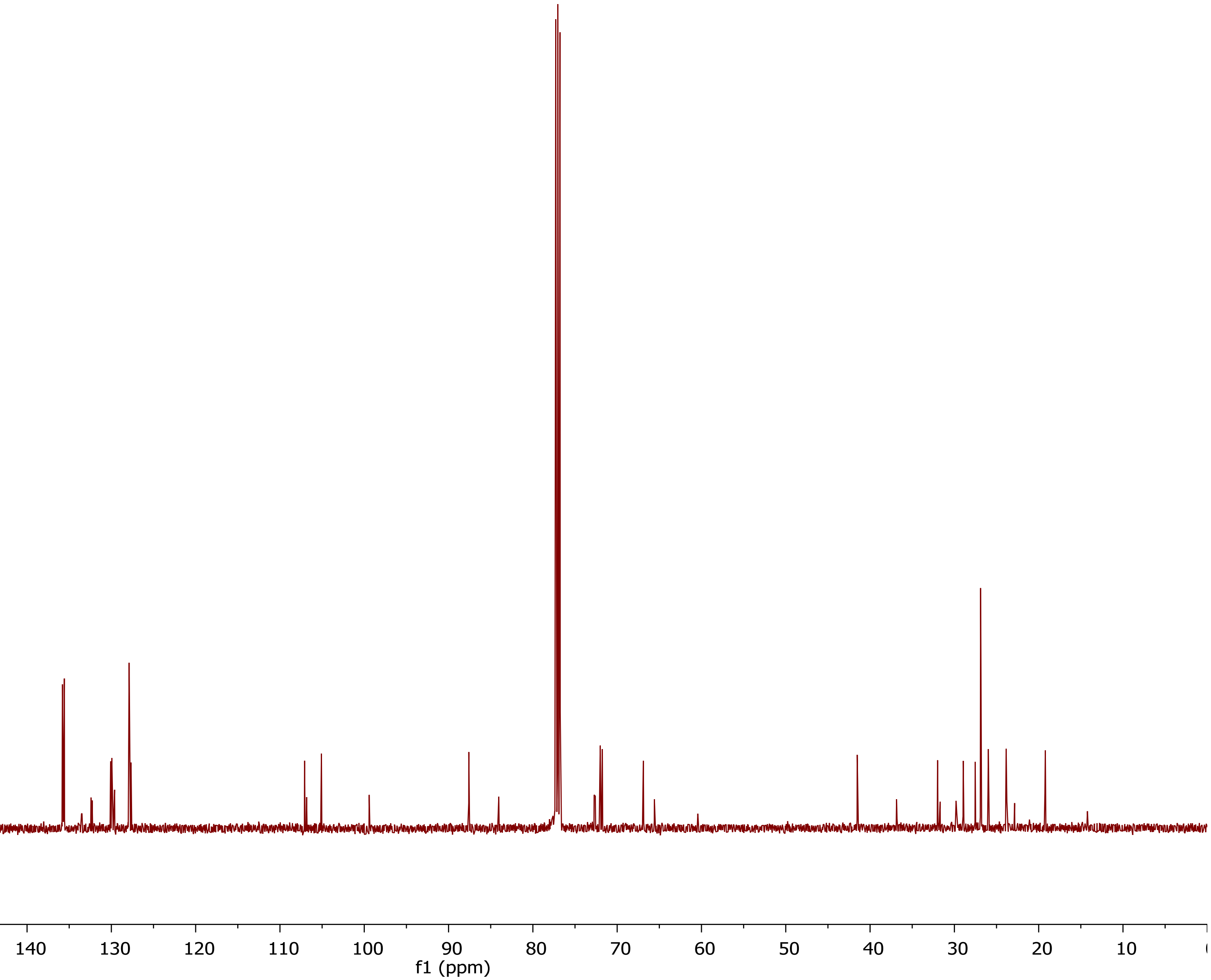

(((3aS,4aS,5S,7S,7aR,8aR)-7-allyl-2,2-dimethyloctahydroisobenzofuro[5,6-d][1,3]dioxol-5-yl)methoxy)(tert-butyl)diphenylsilane **33a**

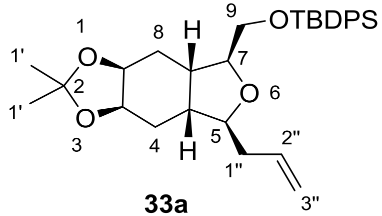

33a

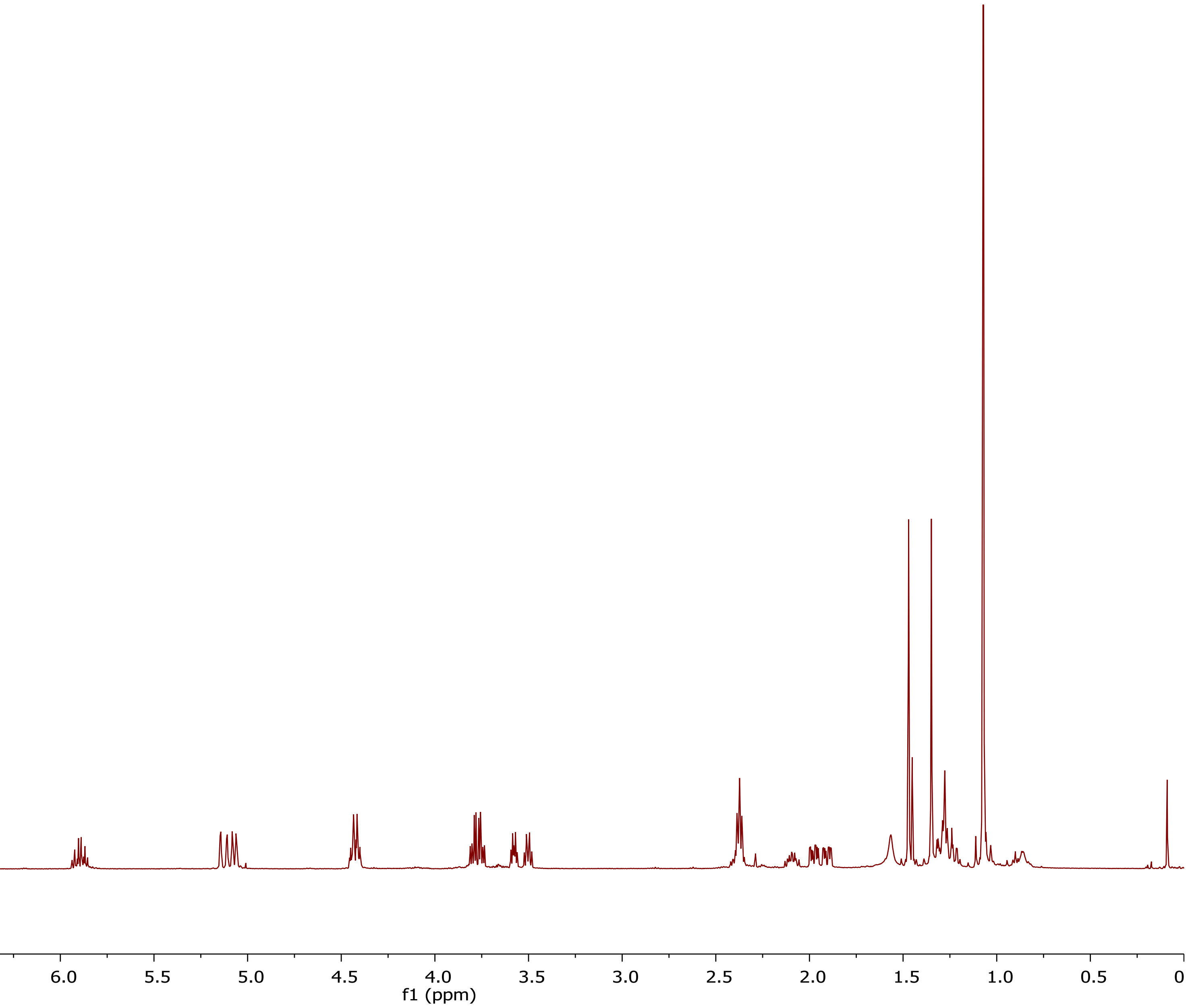

(((3aS,4aS,5S,7S,7aR,8aR)-7-allyl-2,2-dimethyloctahydroisobenzofuro[5,6-d][1,3]dioxol-5-yl)methoxy)(tert-butyl)diphenylsilane **33a**

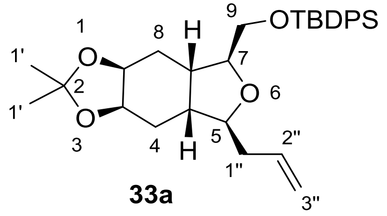

33a

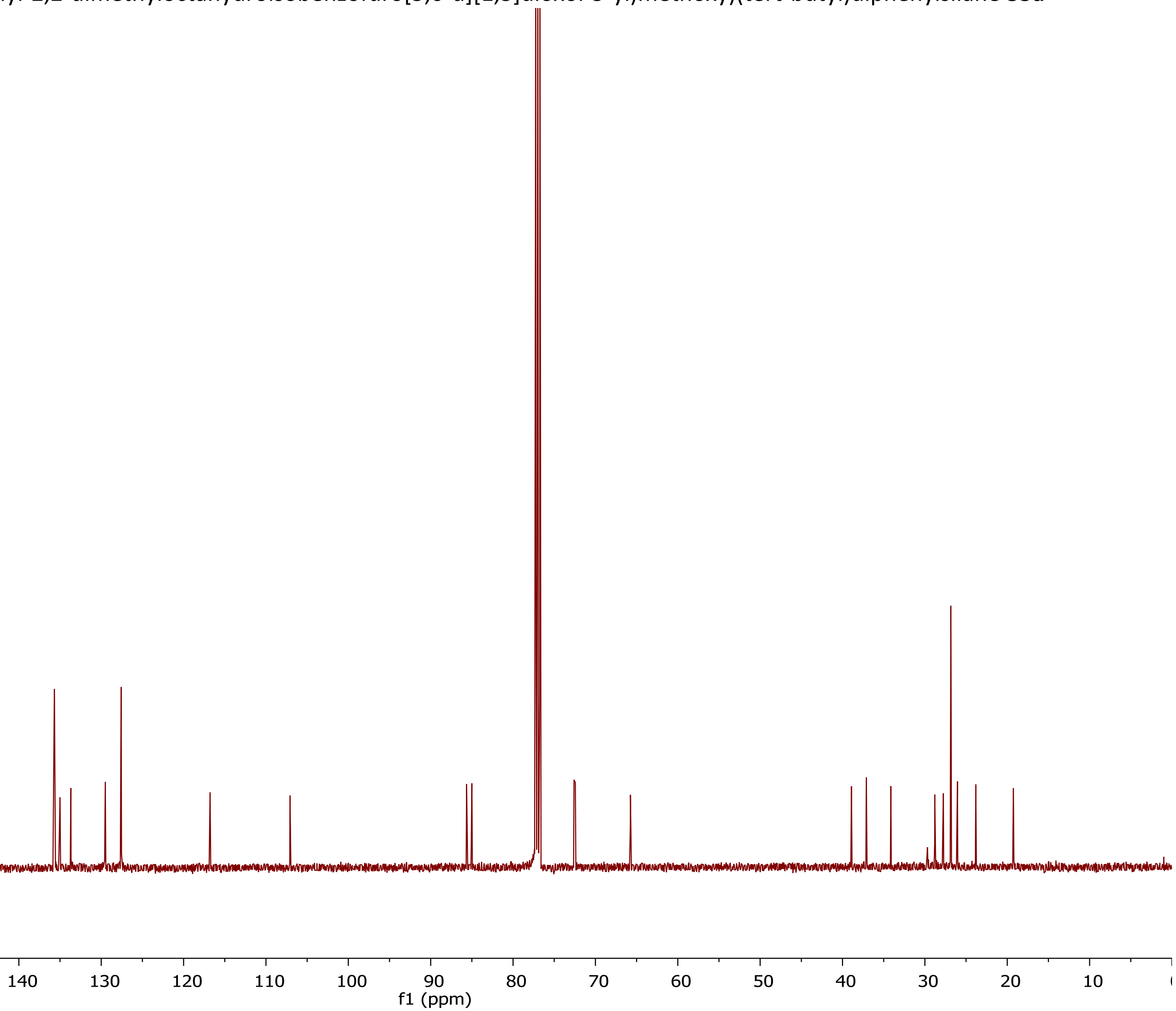

((3aS,4aS,5S,7S,7aR,8aR)-7-allyl-2,2-dimethyloctahydroisobenzofuro[5,6-d][1,3]dioxol-5-yl)methanol **33**

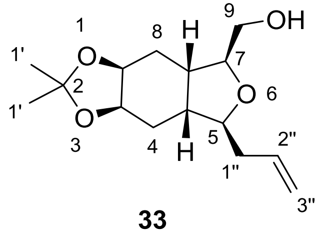

33

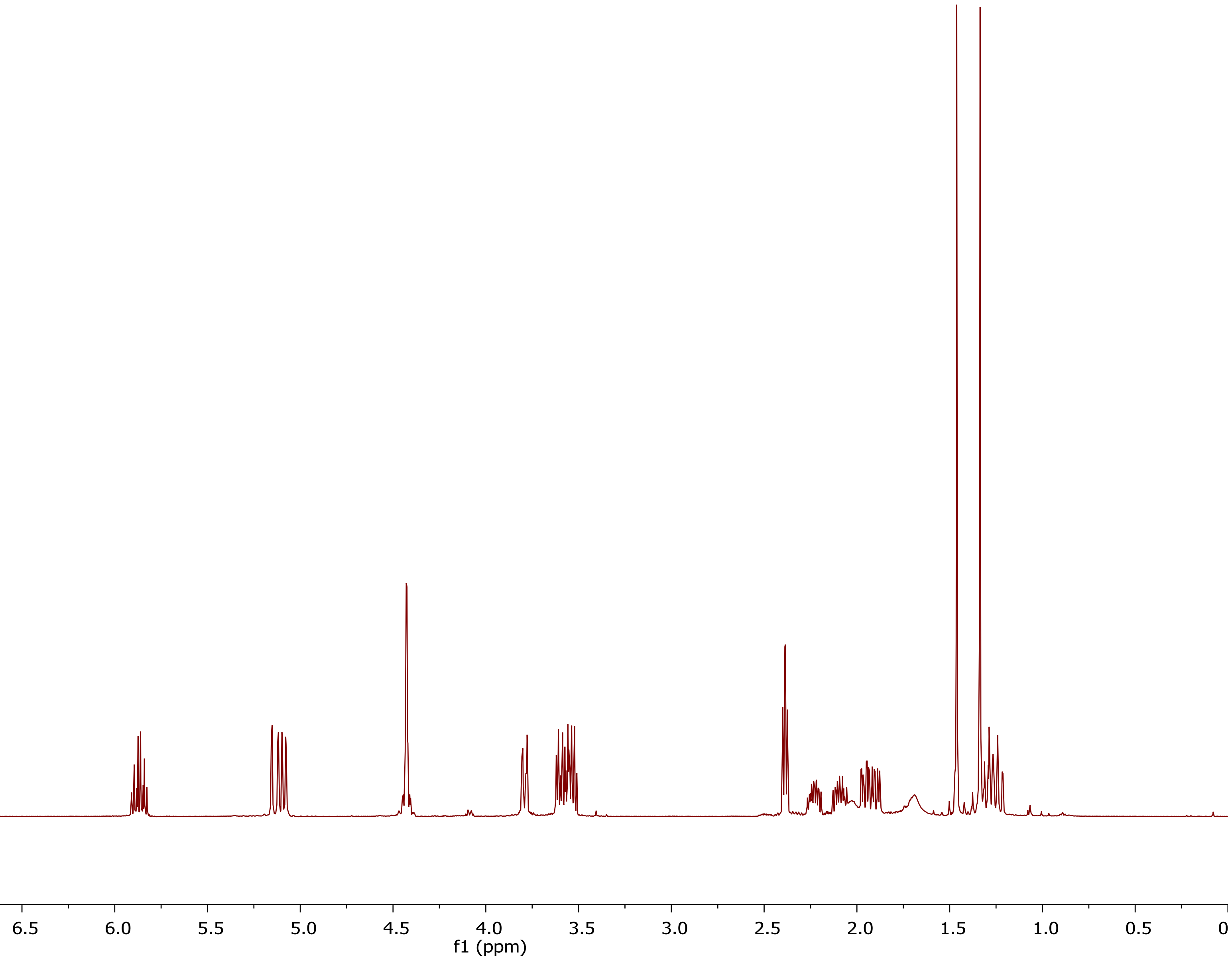

((3aS,4aS,5S,7S,7aR,8aR)-7-allyl-2,2-dimethyloctahydroisobenzofuro[5,6-d][1,3]dioxol-5-yl)methanol **33**

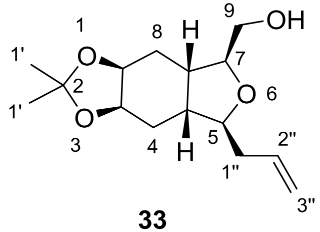

33

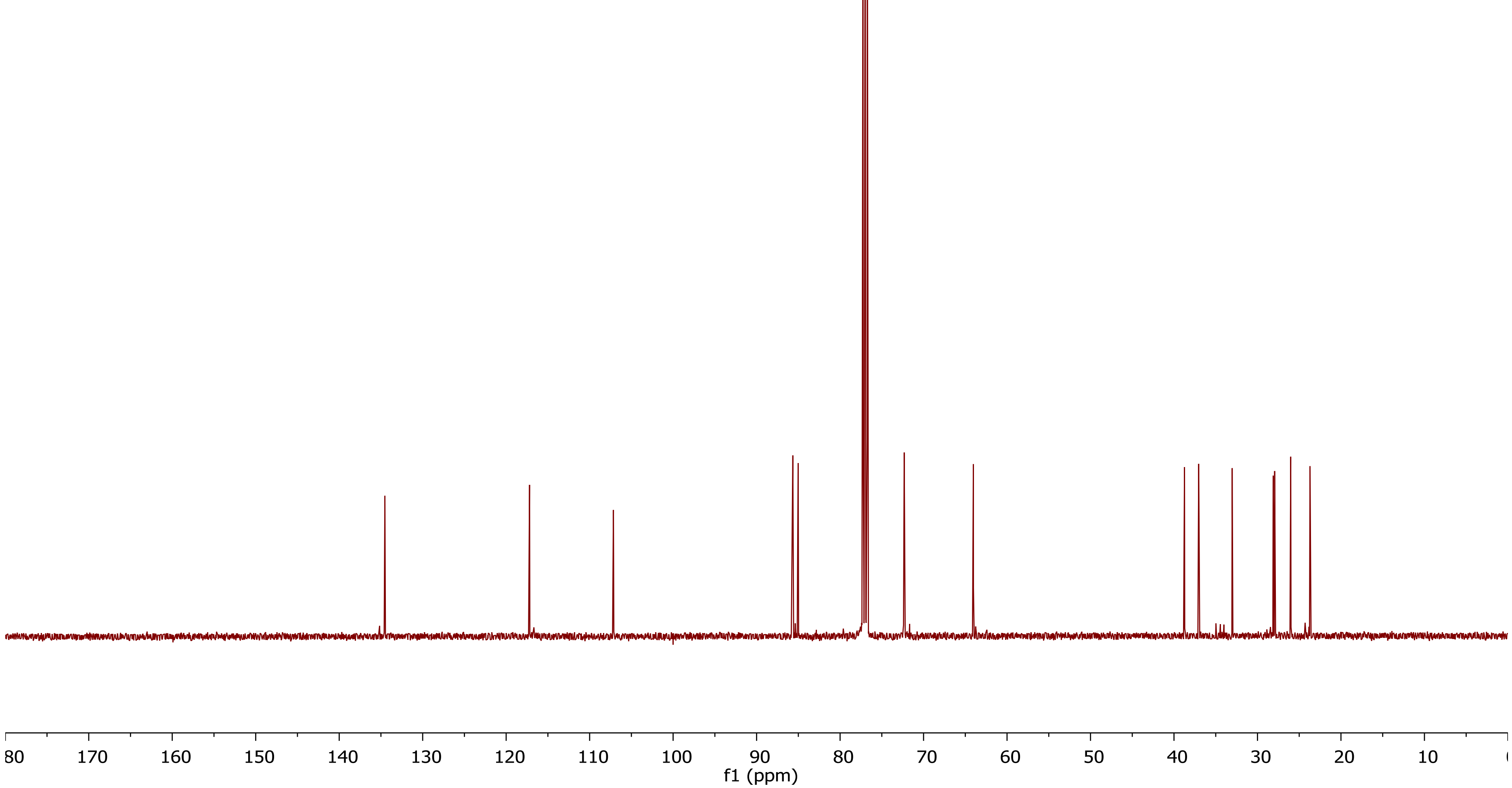

((3aS,4aS,5S,7S,7aR,8aR)-7-allyl-2,2-dimethyloctahydroisobenzofuro[5,6-d][1,3]dioxol-5-yl)methyl hex-5-enoate **34**

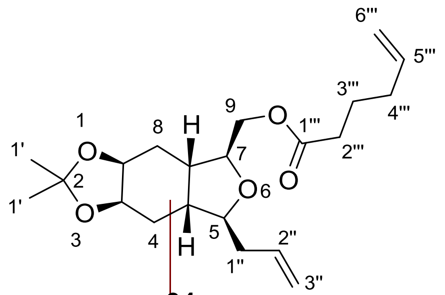

34

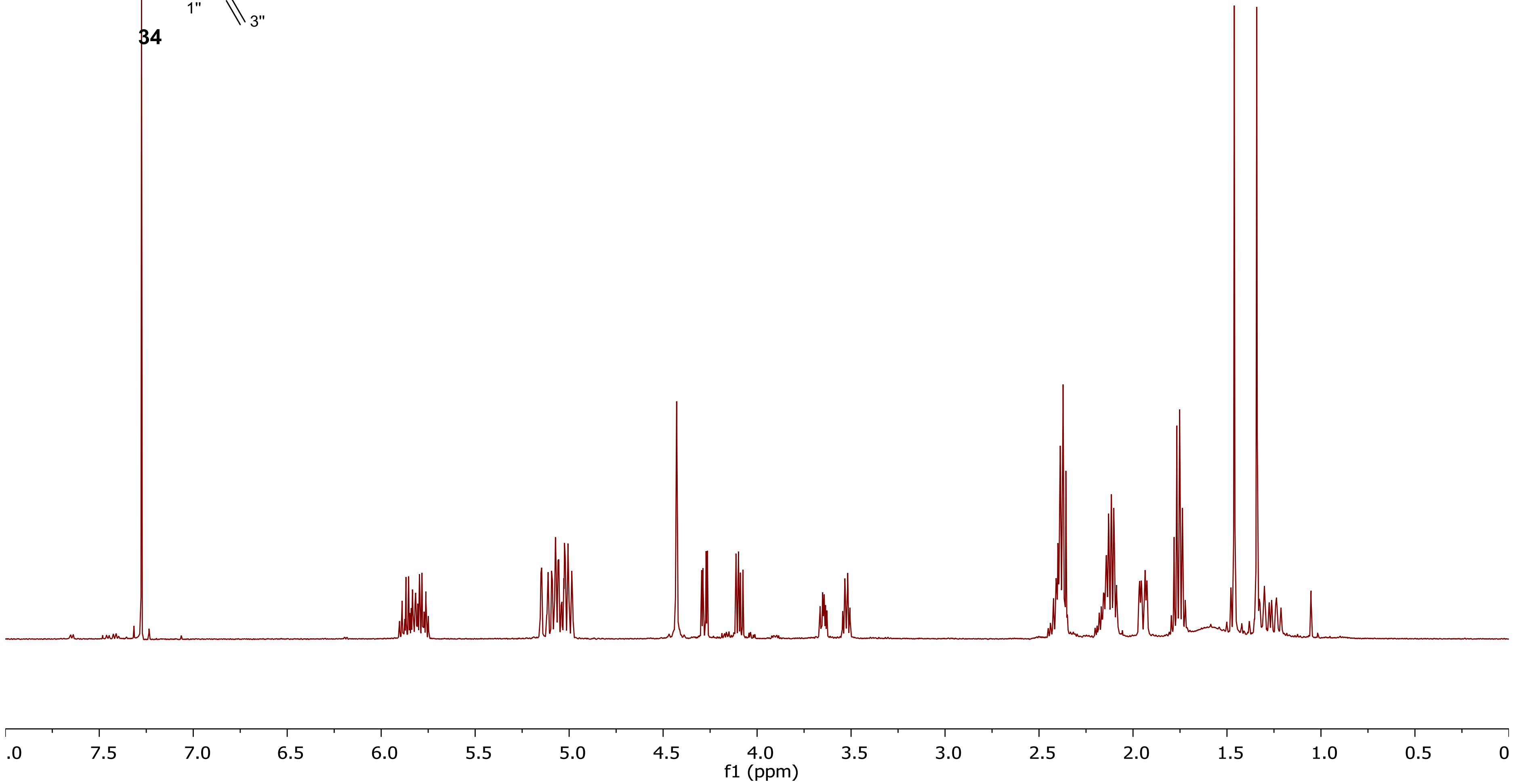

((3aS,4aS,5S,7S,7aR,8aR)-7-allyl-2,2-dimethyloctahydroisobenzofuro[5,6-d][1,3]dioxol-5-yl)methyl hex-5-enoate **34**

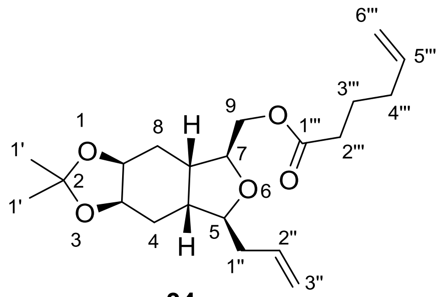

34

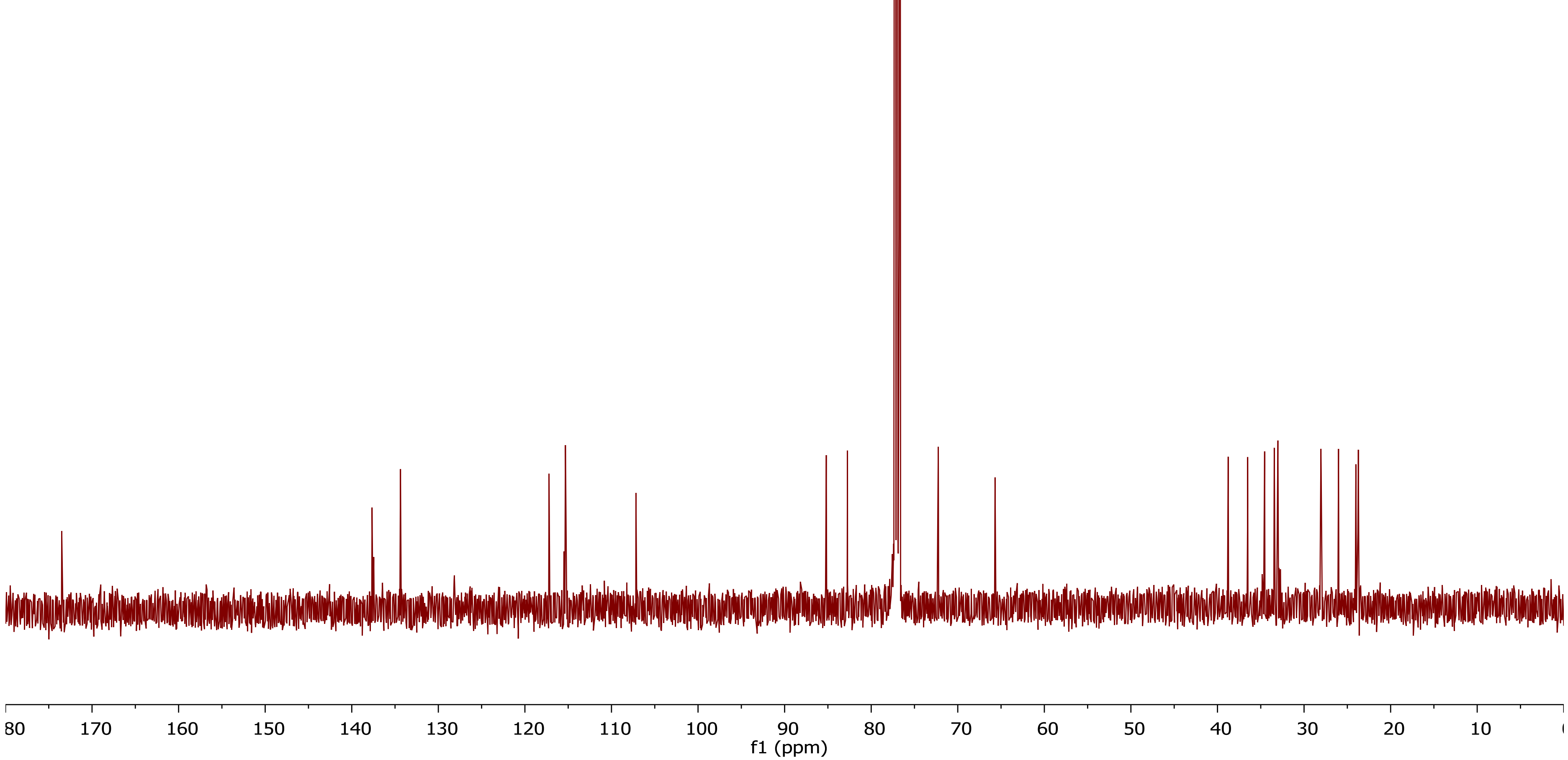

(1S,2R,4R,8S,10S,11S,18E)-6,6-dimethyl-5,7,13,21- tetraoxatetracyclo[9.9.1.0^{2,10}.0^{4,8}]henicos-18-en-14-one **35**

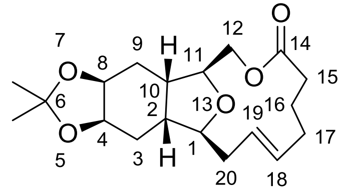

35

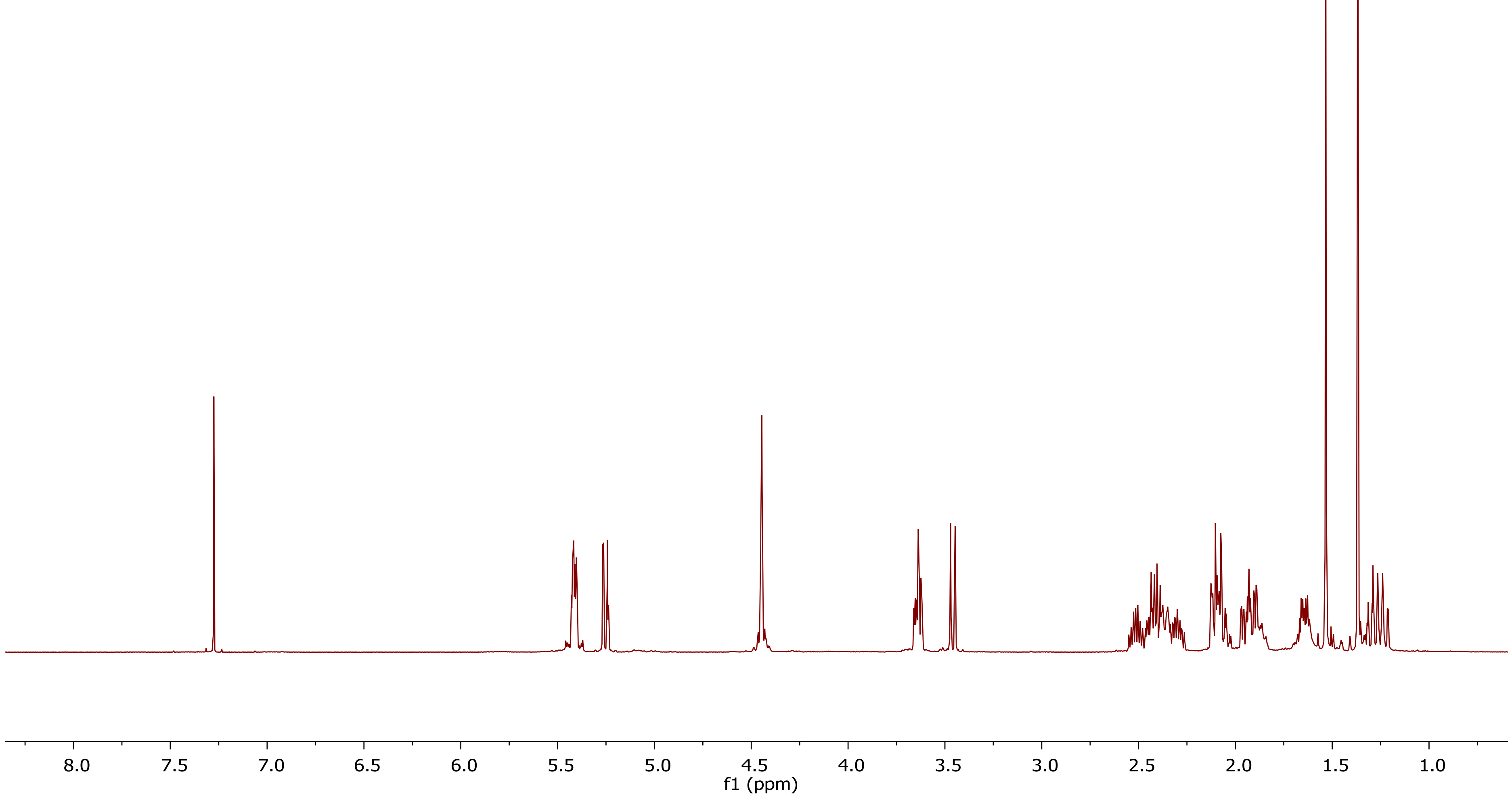

(1S,2R,4R,8S,10S,11S,18E)-6,6-dimethyl-5,7,13,21- tetraoxatetracyclo[9.9.1.0^{2,10}.0^{4,8}]henicos-18-en-14-one **35**

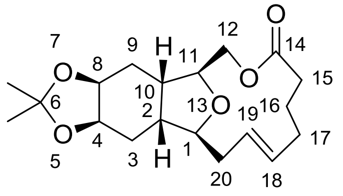

35

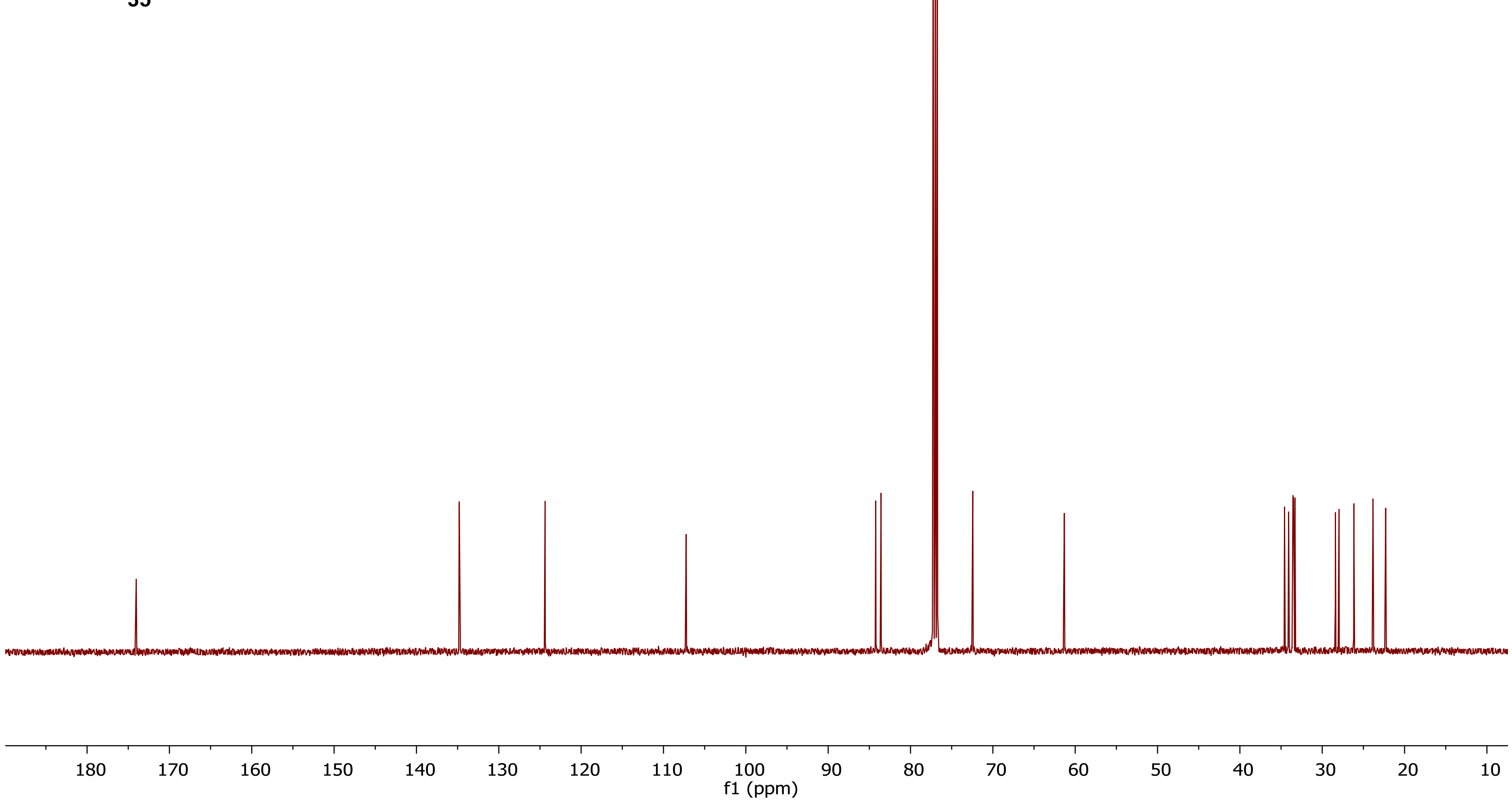

(E)-(1S,11S,12R,14R,15S,17S)-14,15-Dihydroxy-3,18-dioxa-tricyclo[9.6.1.012,17]octadec-8-en-4-one **36**

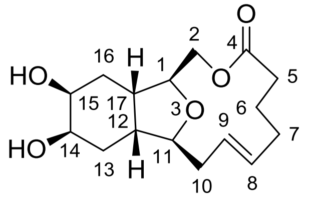

36

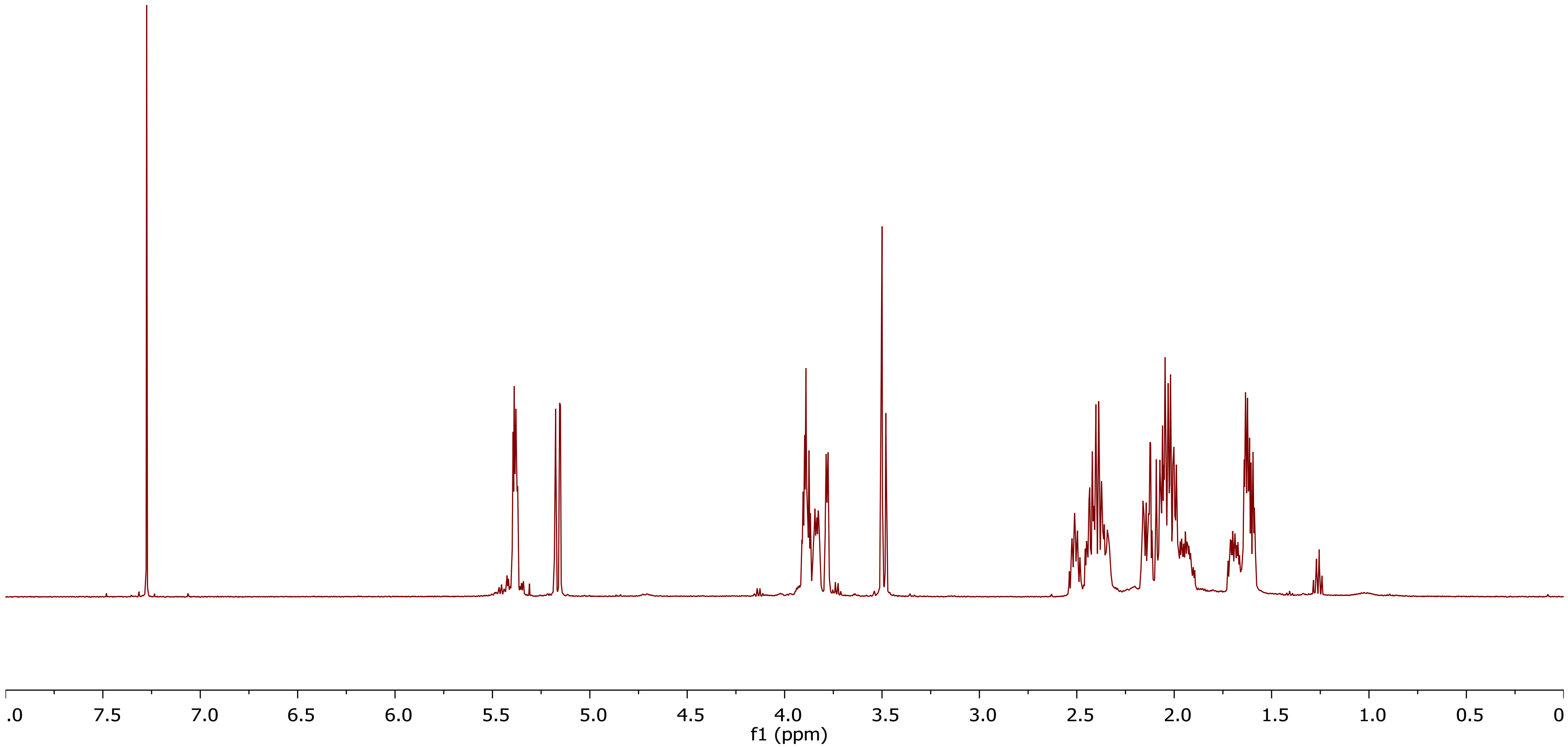

(E)-(1S,11S,12R,14R,15S,17S)-14,15-Dihydroxy-3,18-dioxa-tricyclo[9.6.1.012,17]octadec-8-en-4-one **36**

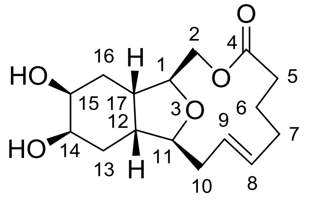

36

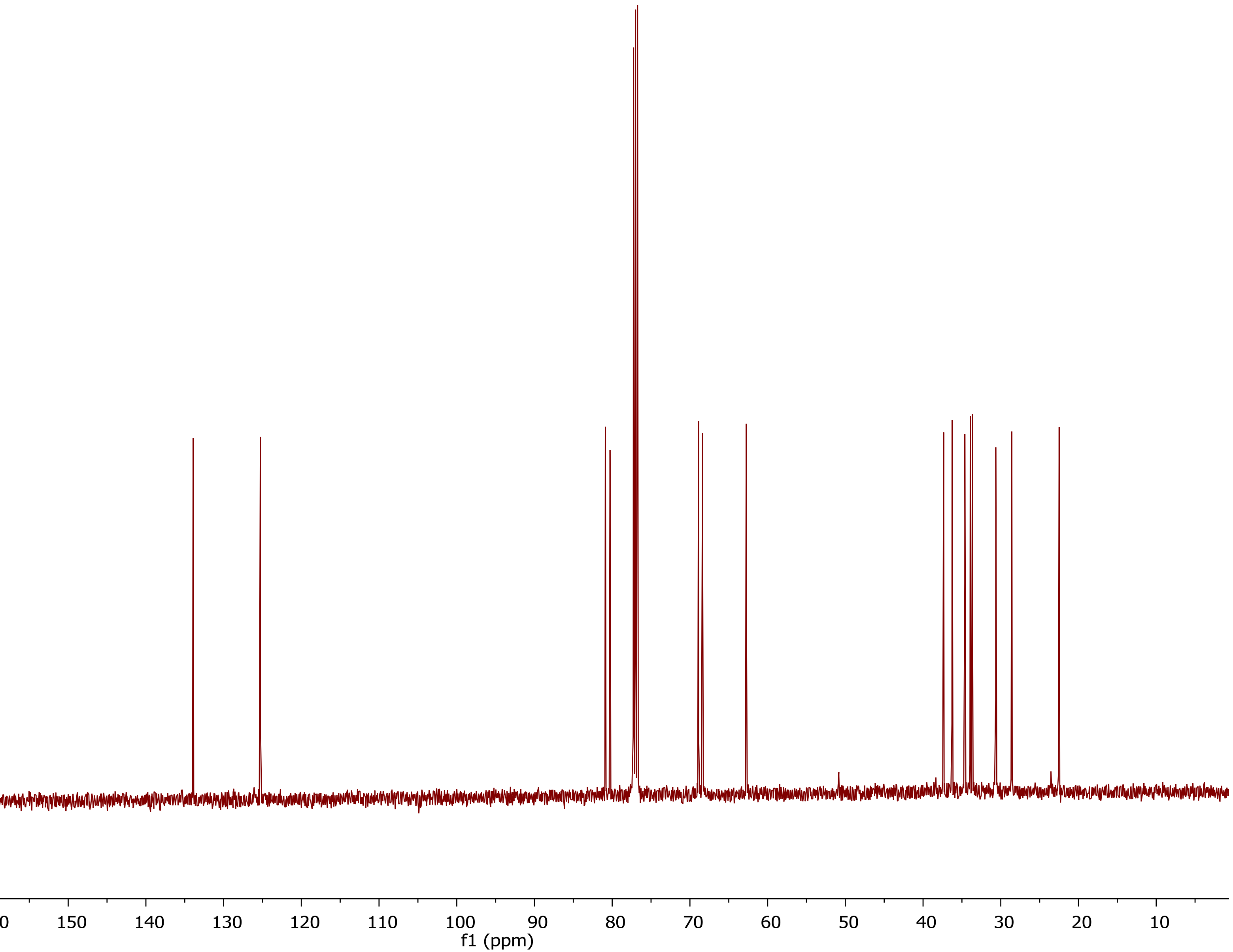

(3S,3aS,7aR)-3-(((tert-butyldiphenylsilyl)oxy)methyl)-5-methyl-3a,4,7,7a-tetrahydroisobenzofuran-1(3H)-one **37**

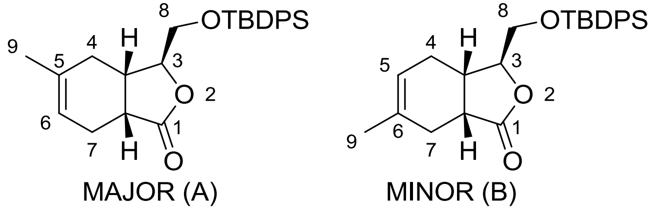

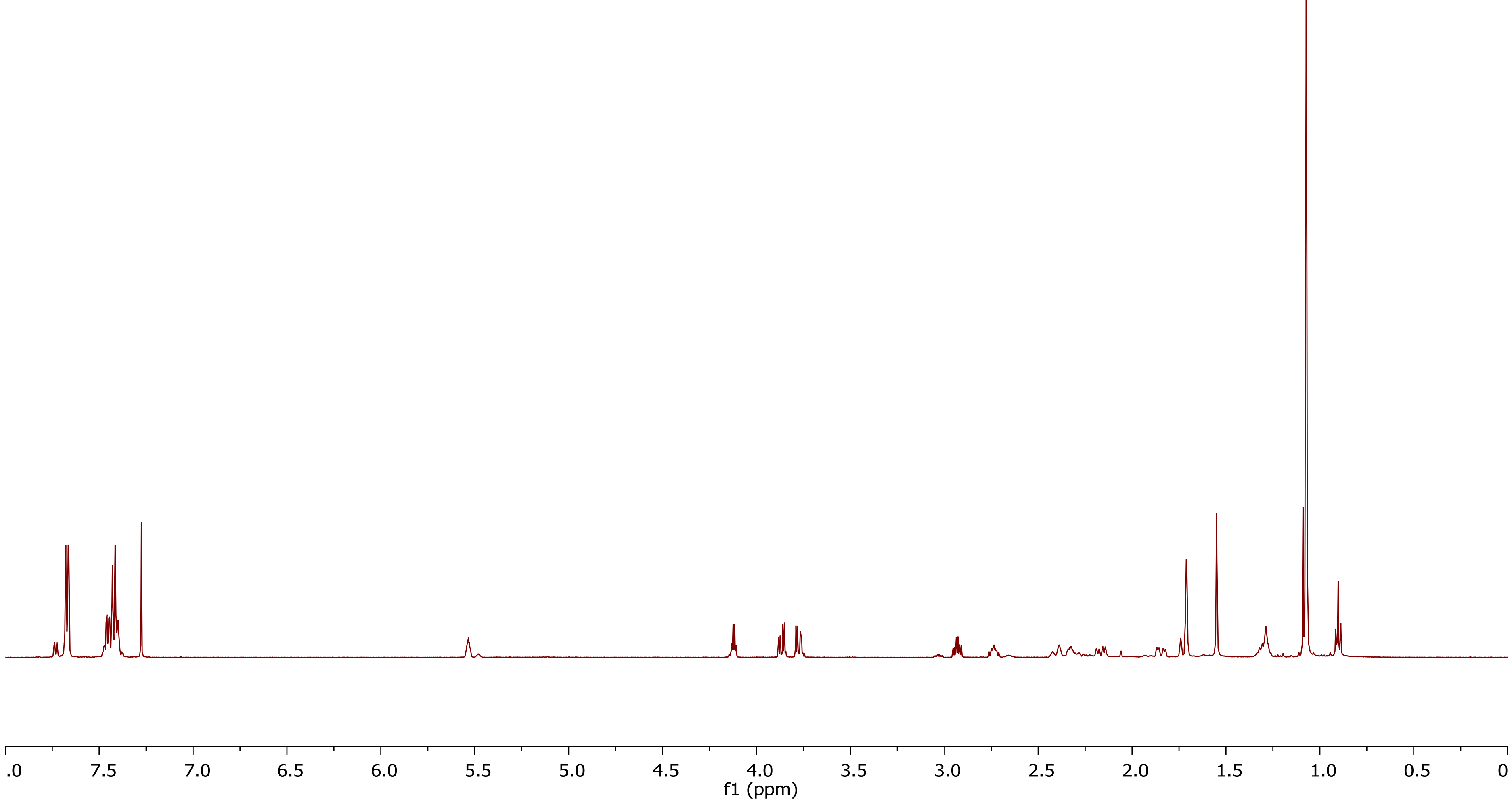

(3S,3aS,7aR)-3-(((tert-butyldiphenylsilyl)oxy)methyl)-5-methyl-3a,4,7,7a-tetrahydroisobenzofuran-1(3H)-one **37**

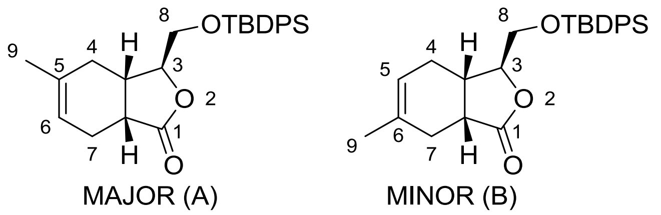

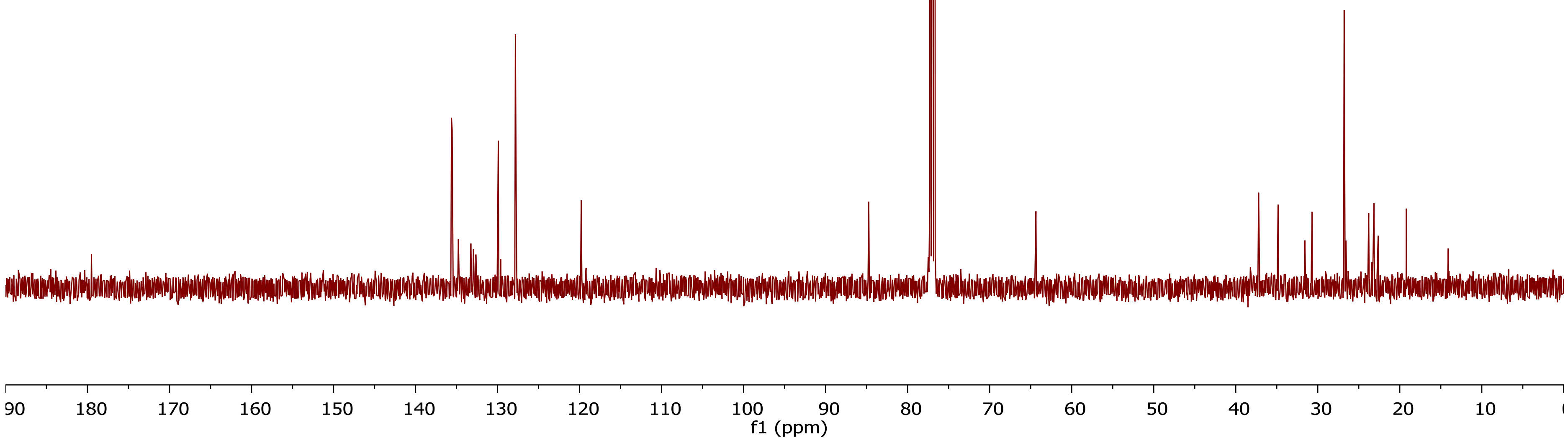

(3S, 3aS, 5R, 6R, 7aR)-3-(*tert*-Butyldiphenylsilanyloxymethyl)-6-hydroxy-5-methylhexahydroisobenzofuran-1-one **38**

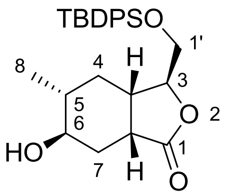

217

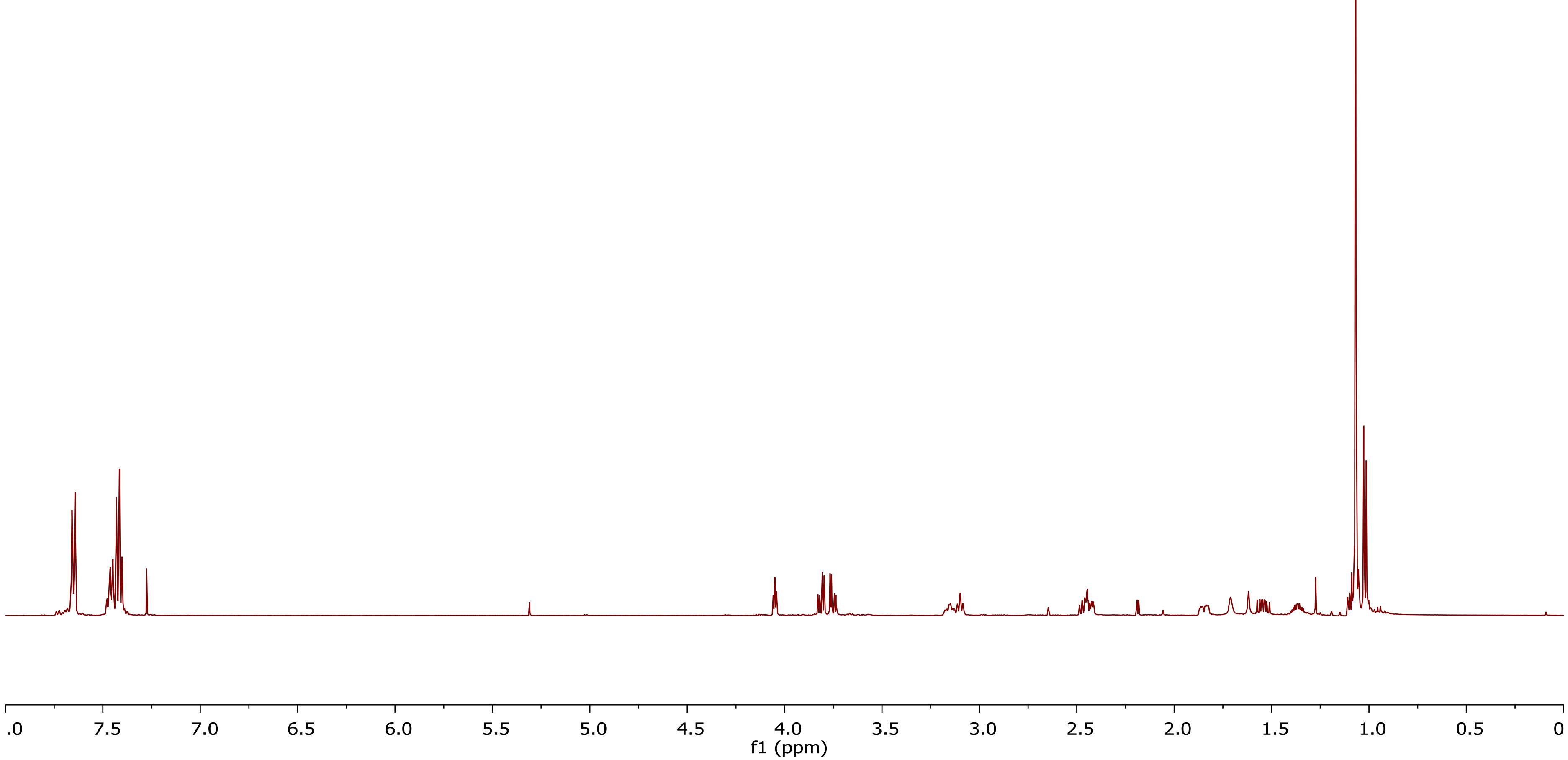

(3S, 3aS, 5R, 6R, 7aR)-3-(*tert*-Butyldiphenylsilanyloxymethyl)-6-hydroxy-5-methylhexahydroisobenzofuran-1-one **38**

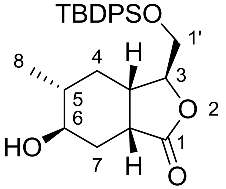

217

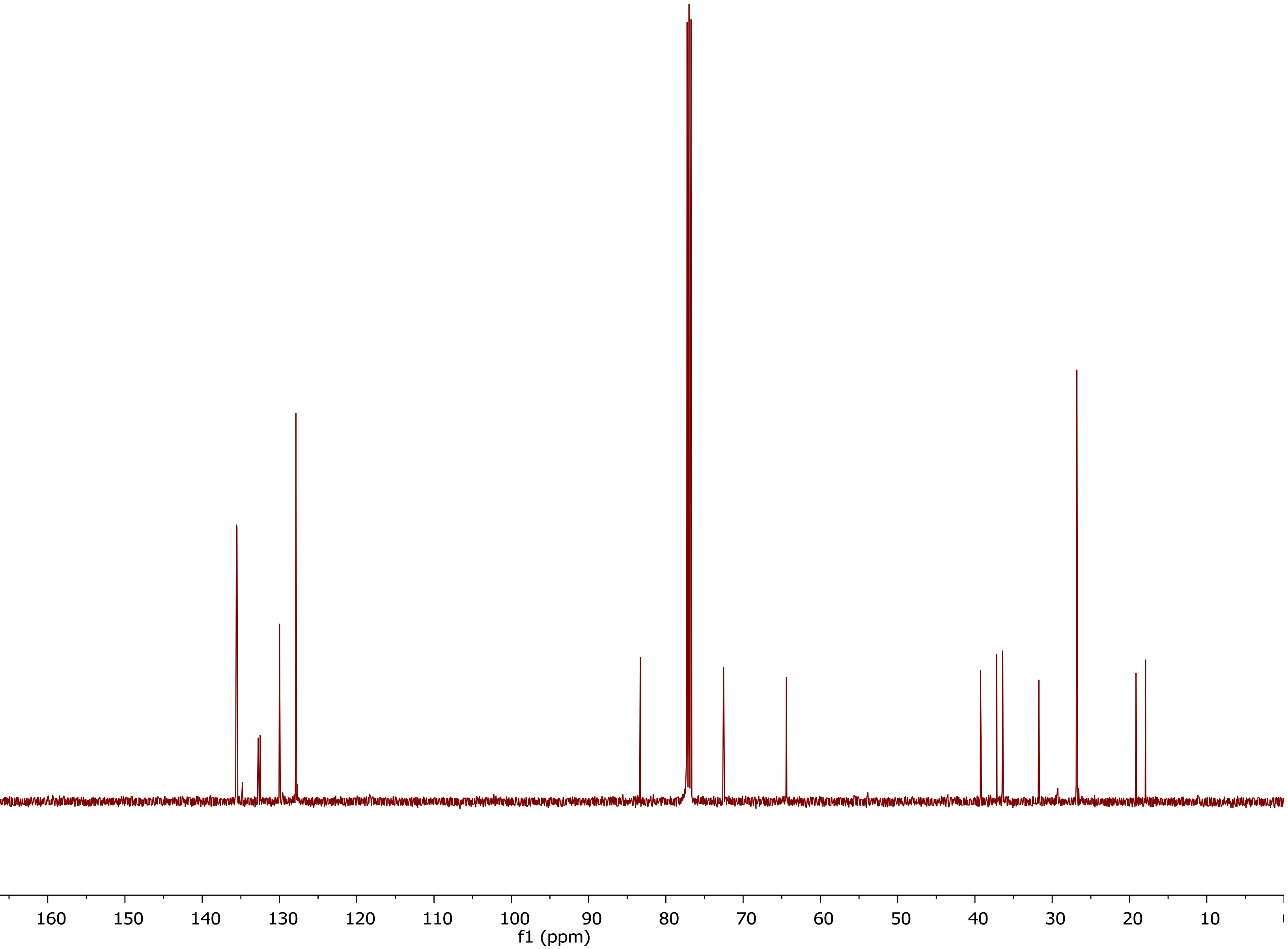

(3S,3aS,5R,6R,7aR)-3-((*tert*-butyldiphenylsilyloxy)methyl)-5-methyloctahydroisobenzofuran-1,6-diol **39a**

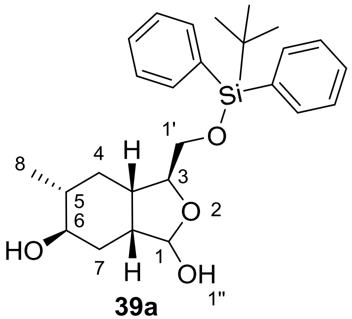

39a

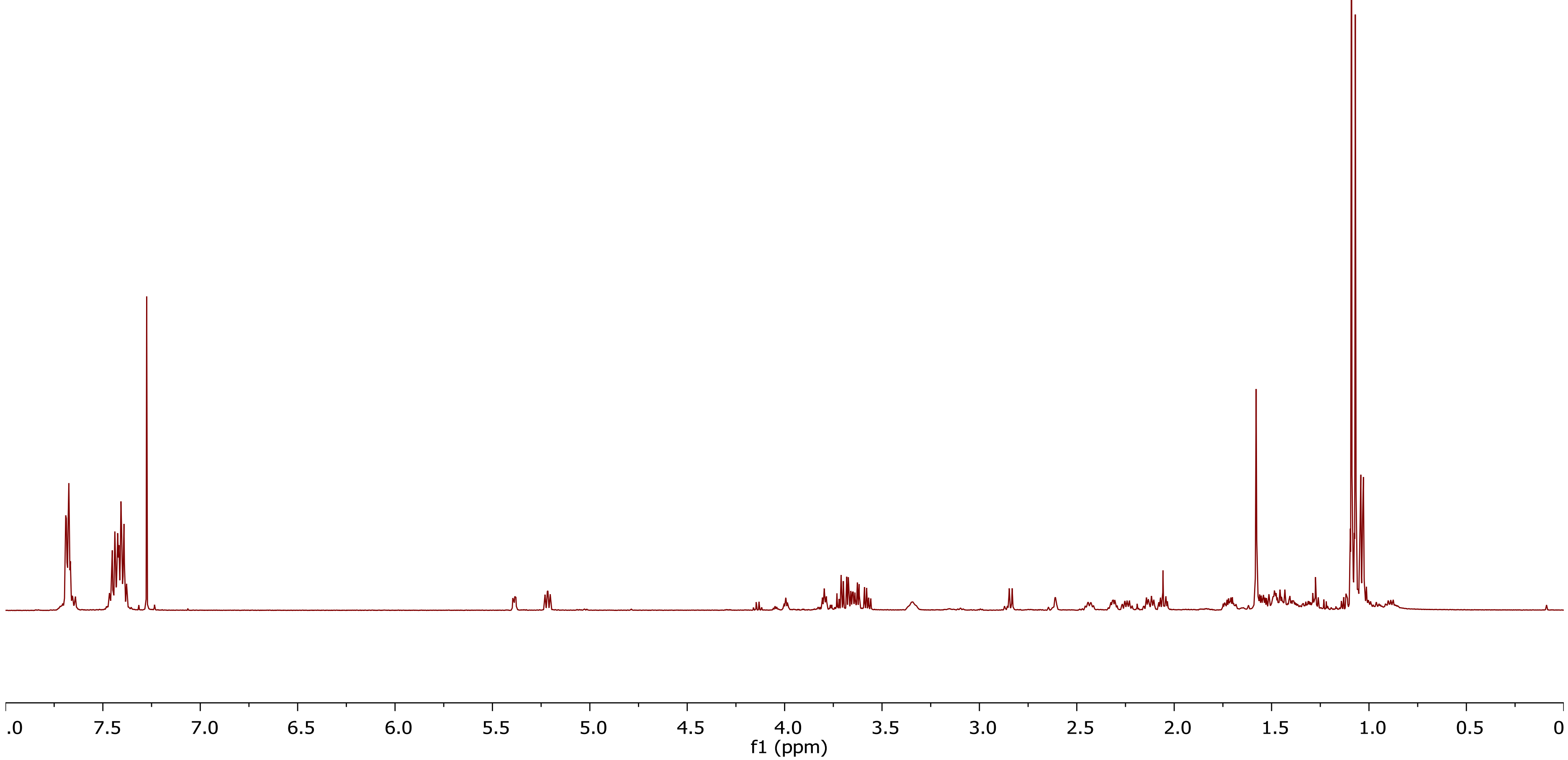

(3S,3aS,5R,6R,7aR)-3-((*tert*-butyldiphenylsilyloxy)methyl)-5-methyloctahydroisobenzofuran-1,6-diol **39a**

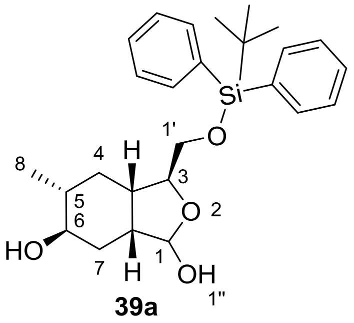

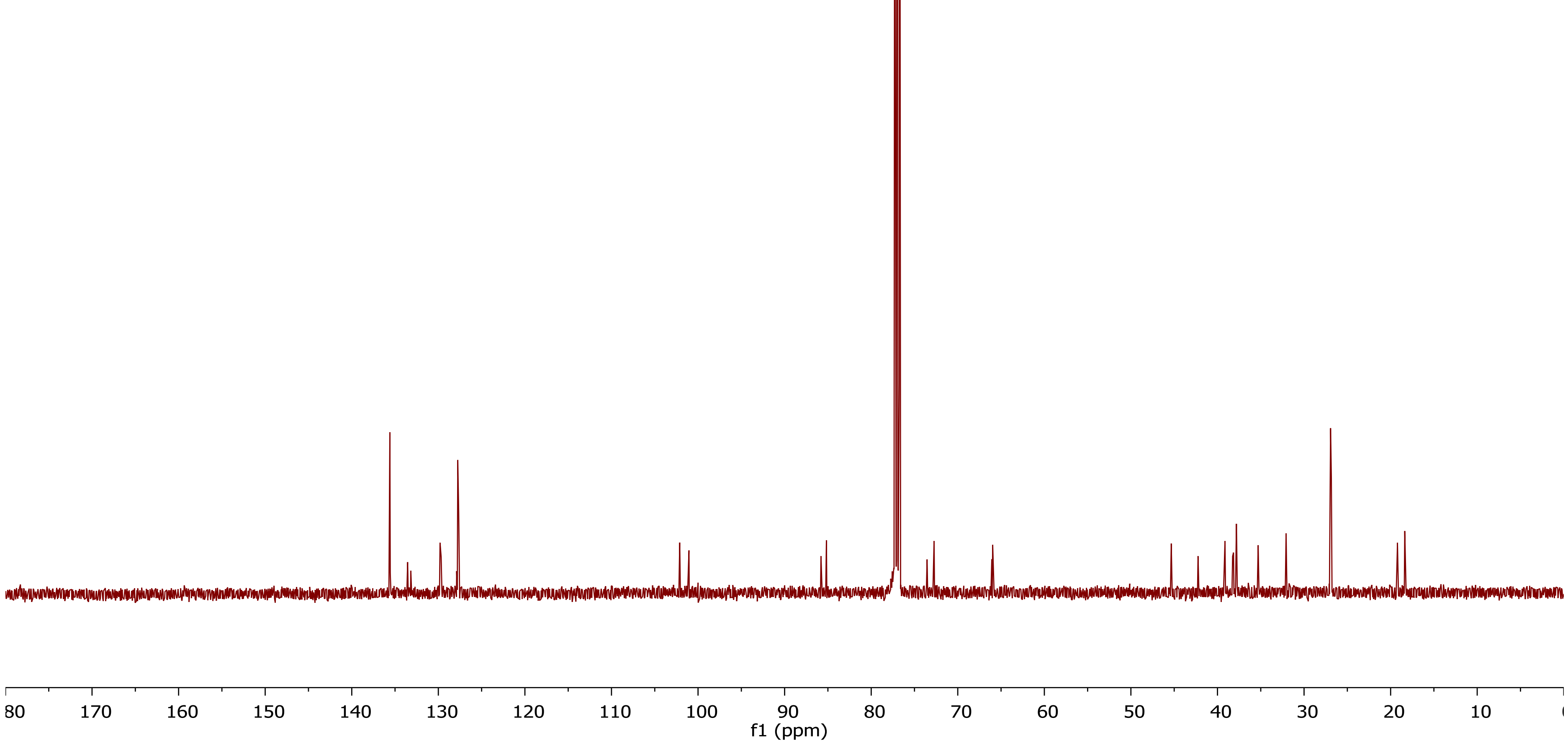

(1S,3S,3aR,5R,6R,7aS)-3-allyl-1-((tert-butyldiphenylsilyloxy)methyl)-6-methyloctahydroisobenzofuran-5-ol **39b**

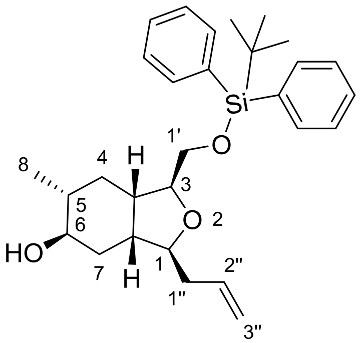

39b

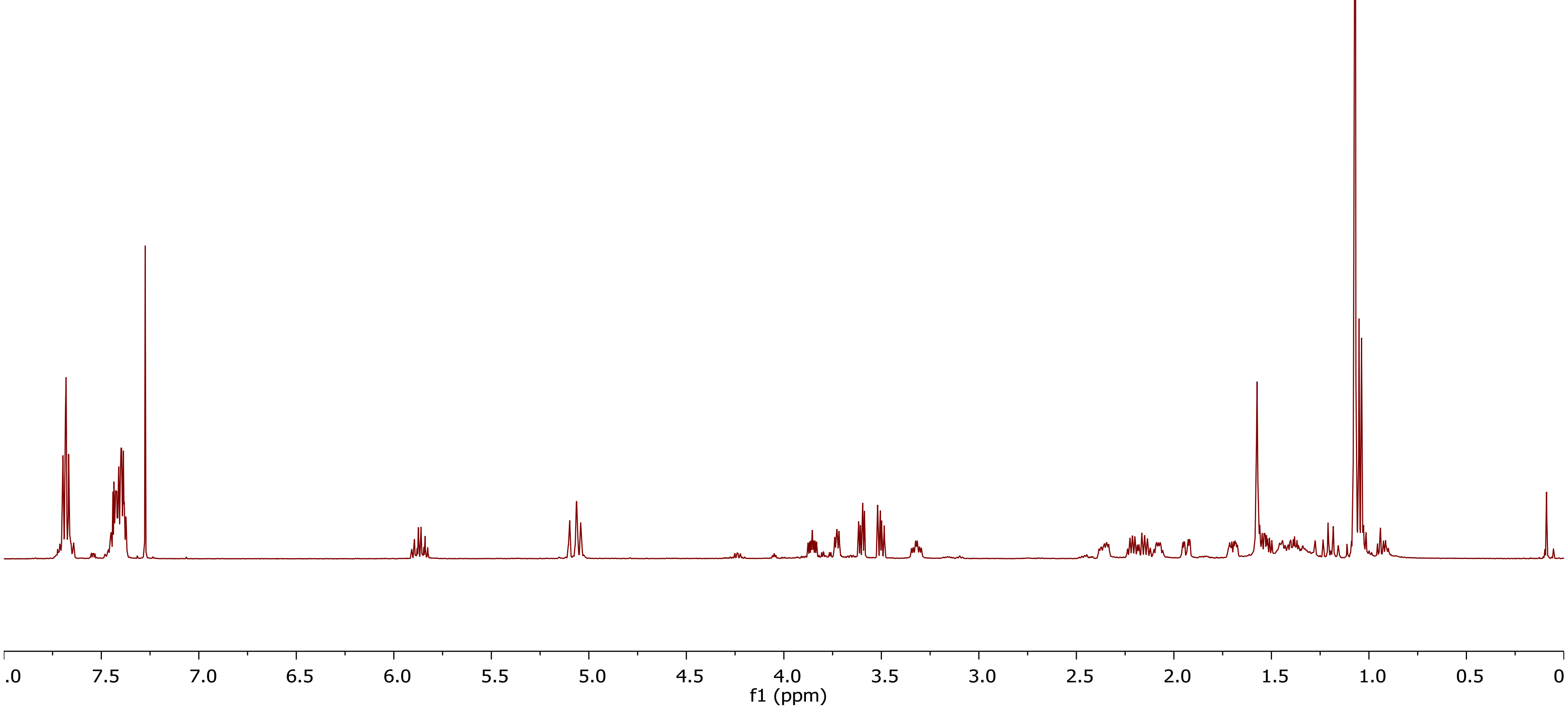

(1S,3S,3aR,5R,6R,7aS)-3-allyl-1-((tert-butyldiphenylsilyloxy)methyl)-6-methyloctahydroisobenzofuran-5-ol **39b**

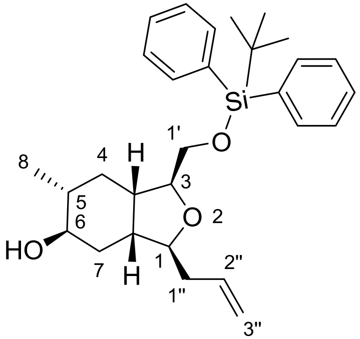

39b

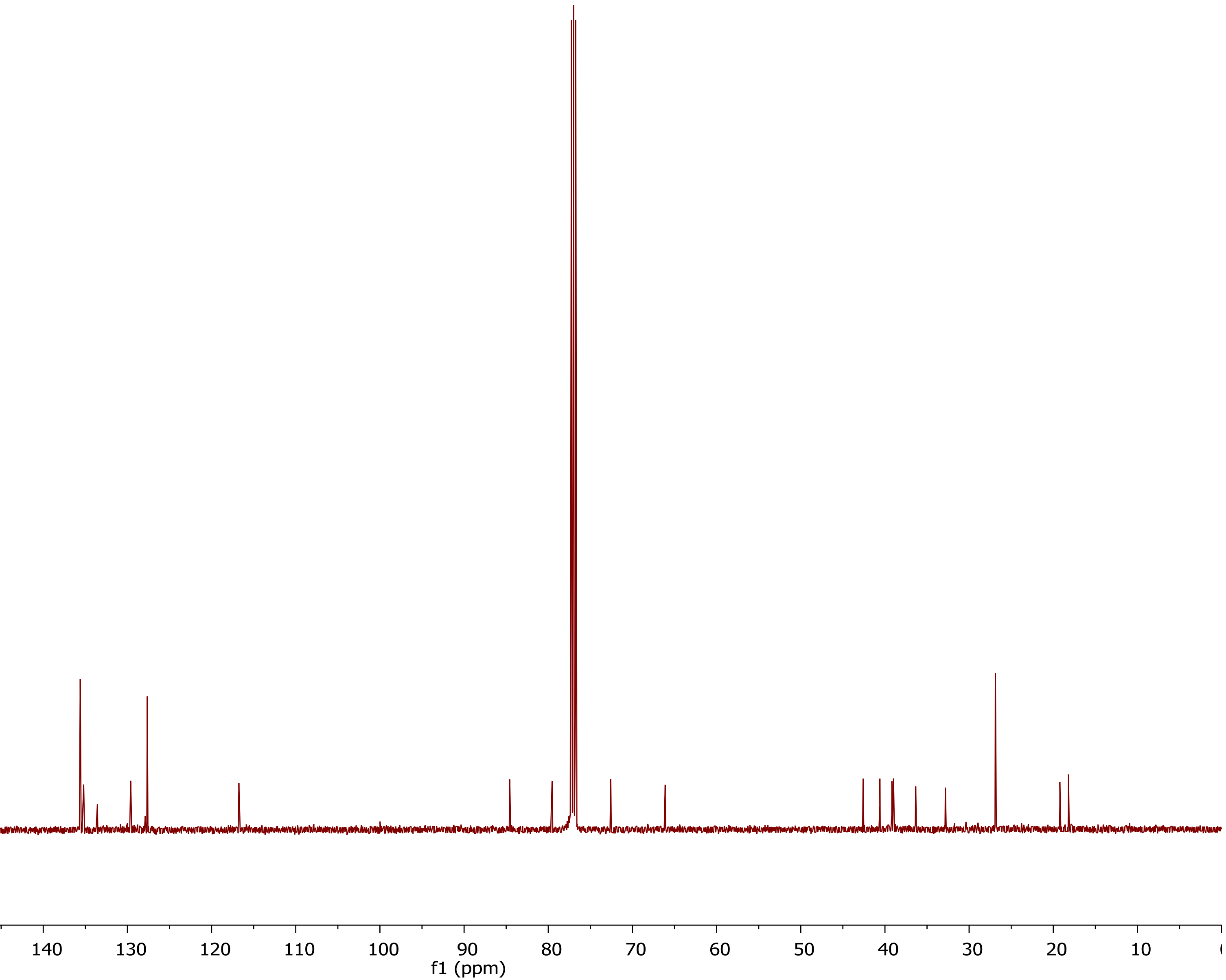

(1S,3S,3aR,5R,6R,7aS)-3-allyl-1-((tert-butyldiphenylsilyloxy)methyl)-6-methyloctahydroisobenzofuran-5-ol **39b**

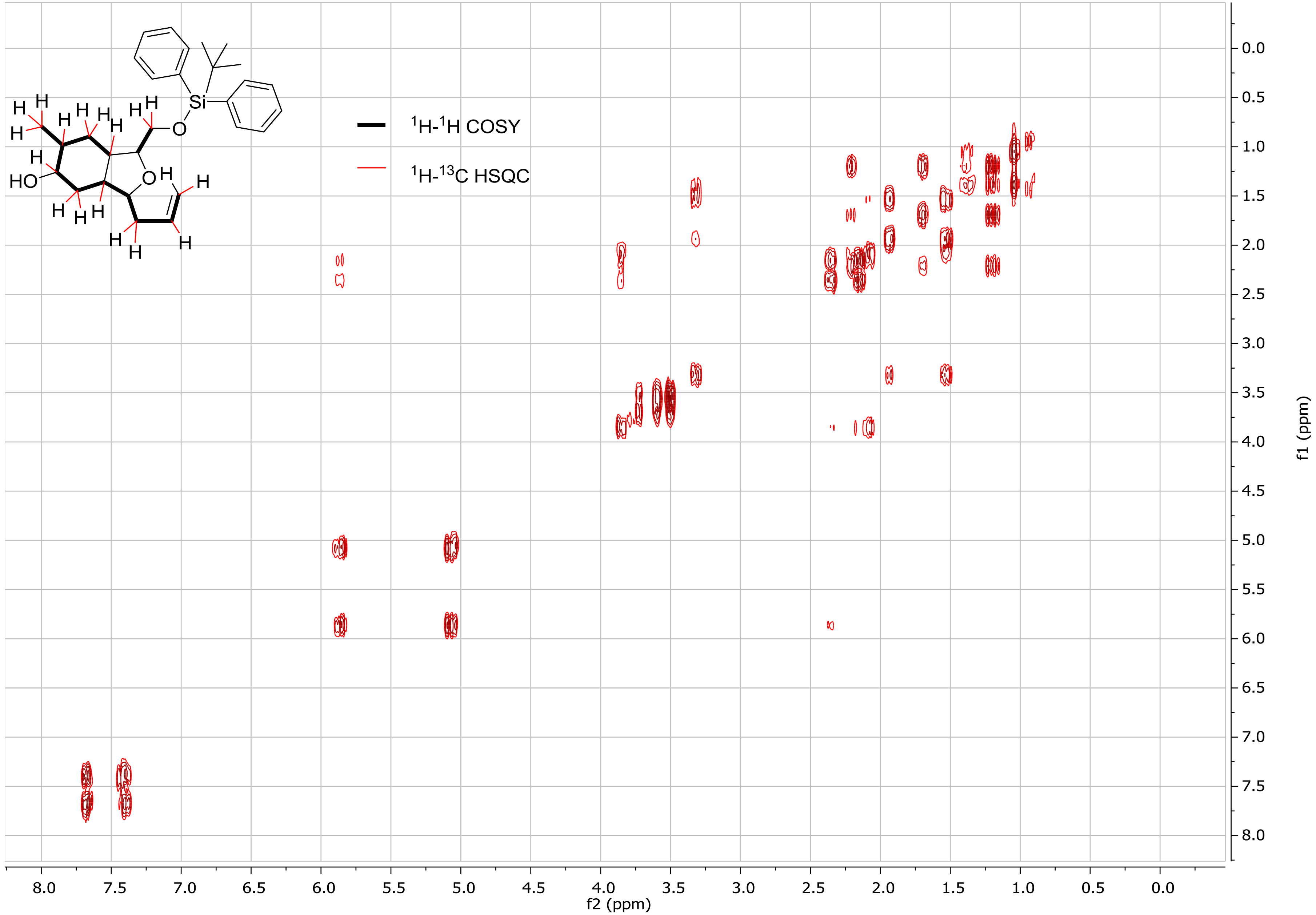

(1S,3S,3aR,5R,6R,7aS)-3-allyl-1-((tert-butyldiphenylsilyloxy)methyl)-6-methyloctahydroisobenzofuran-5-ol **39b**

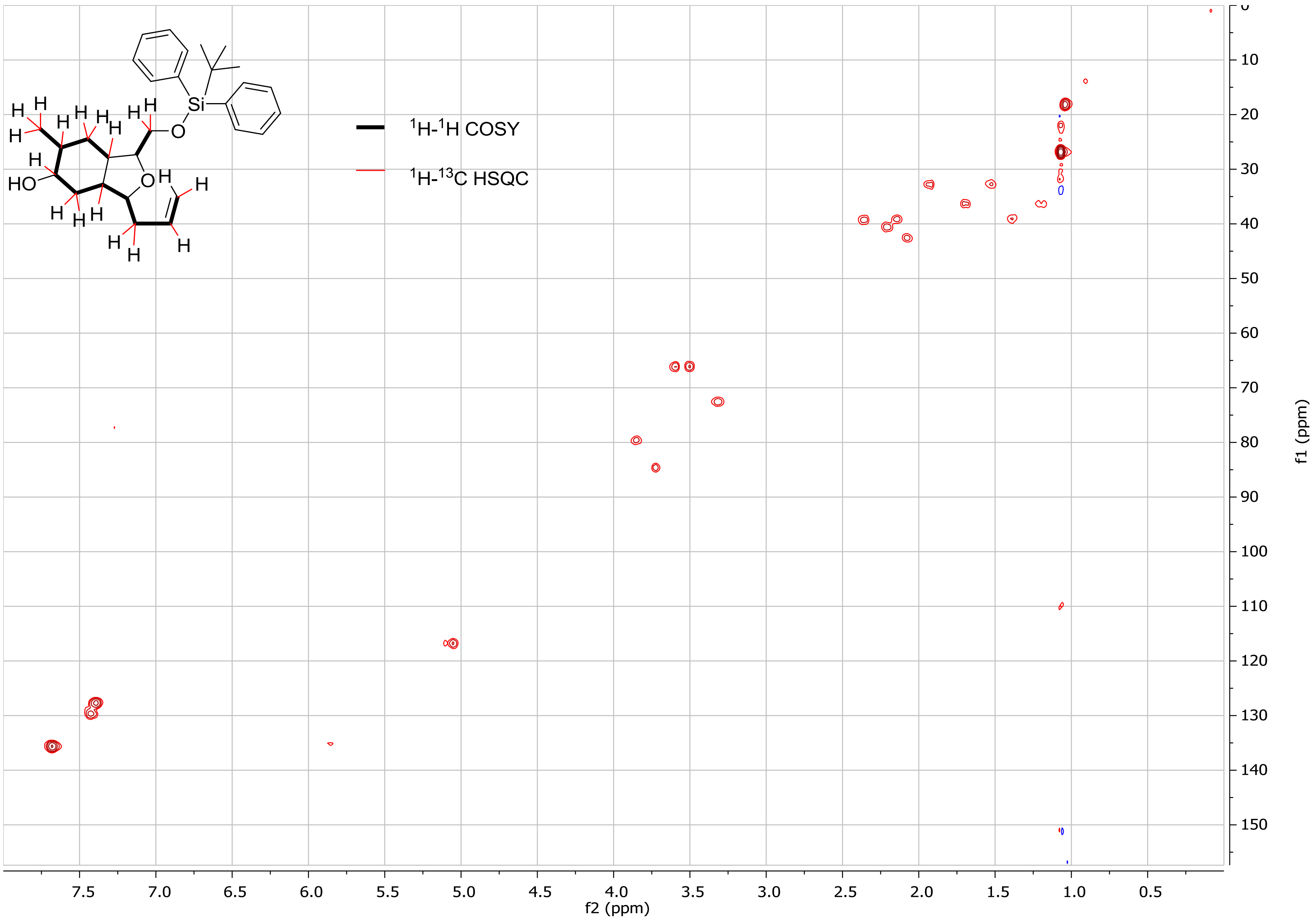

(1S,3S,3aR,5R,6R,7aS)-3-allyl-1-(hydroxymethyl)-6-methyloctahydroisobenzofuran-5-ol **86**

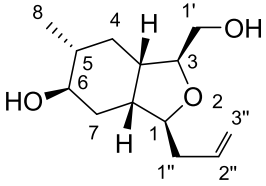

86

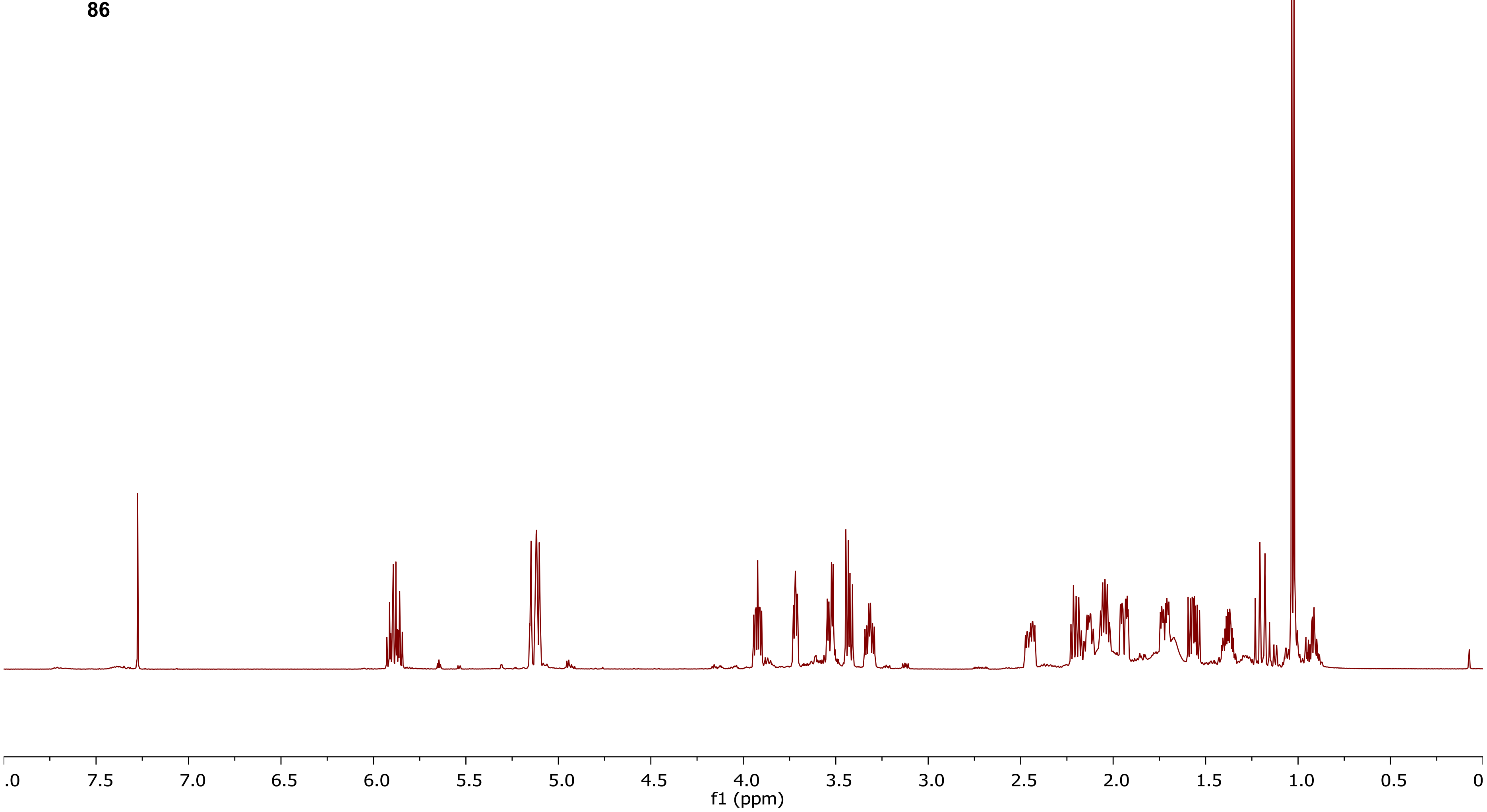

(1S,3S,3aR,5R,6R,7aS)-3-allyl-1-(hydroxymethyl)-6-methyloctahydroisobenzofuran-5-ol **86**

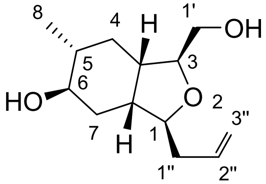

86

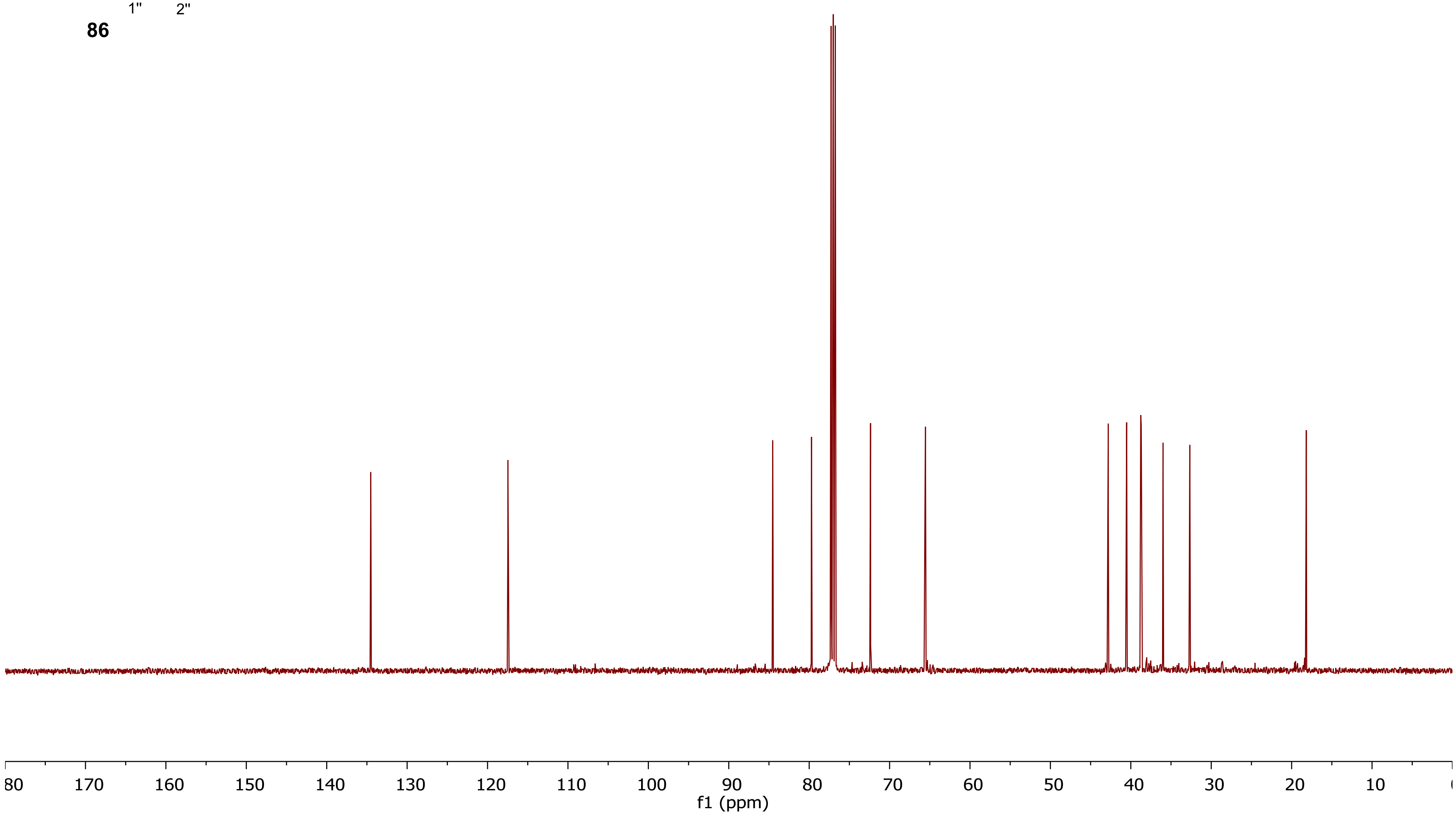

((1S,3S,3aR,5R,6R,7aS)-3-Allyl-5-hydroxy-6-methyloctahydroisobenzofuran-1-yl)methyl hex-5-enoate **87**

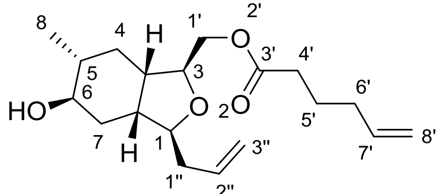

87

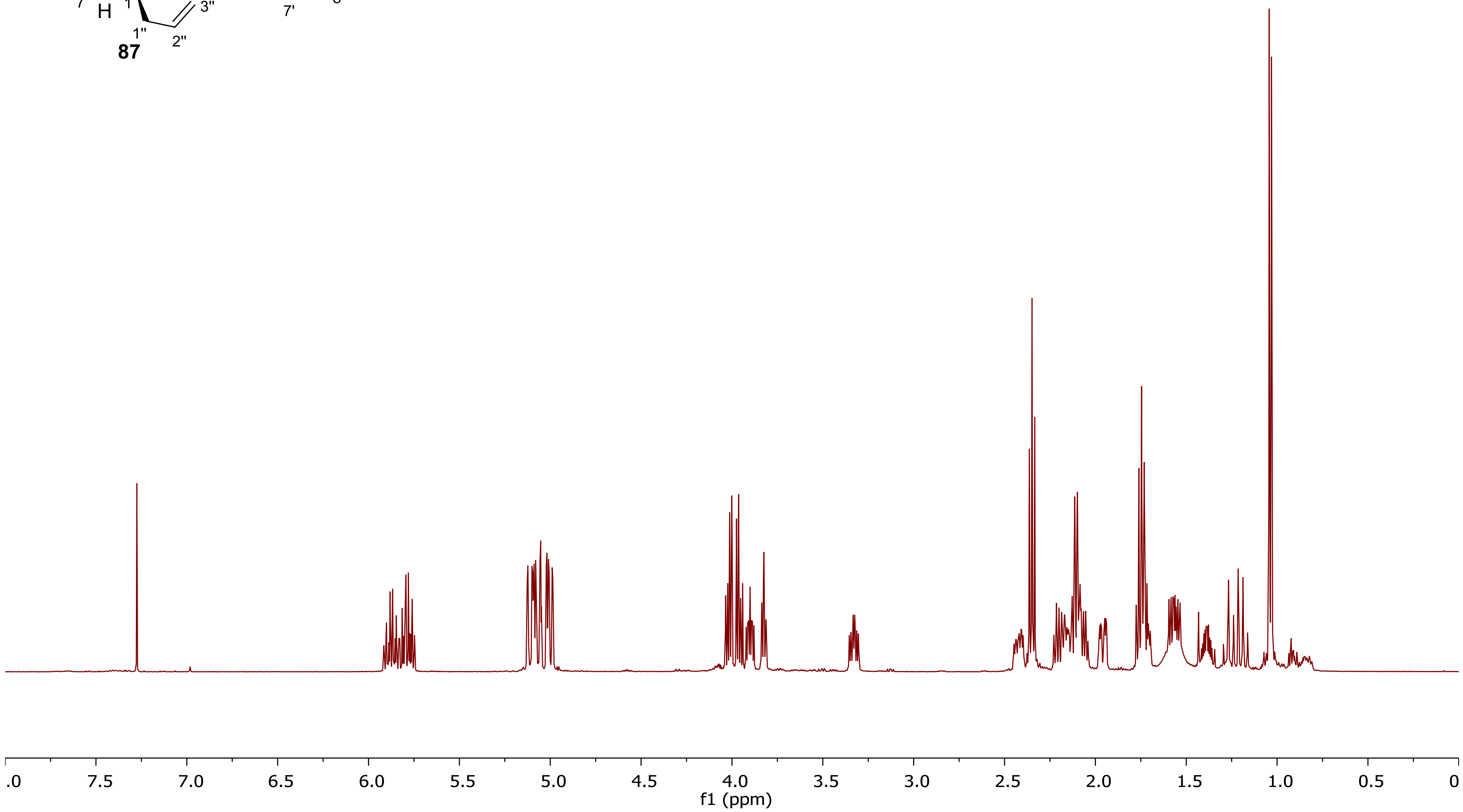

((1S,3S,3aR,5R,6R,7aS)-3-Allyl-5-hydroxy-6-methyloctahydroisobenzofuran-1-yl)methyl hex-5-enoate **87**

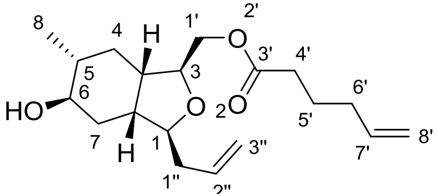

87

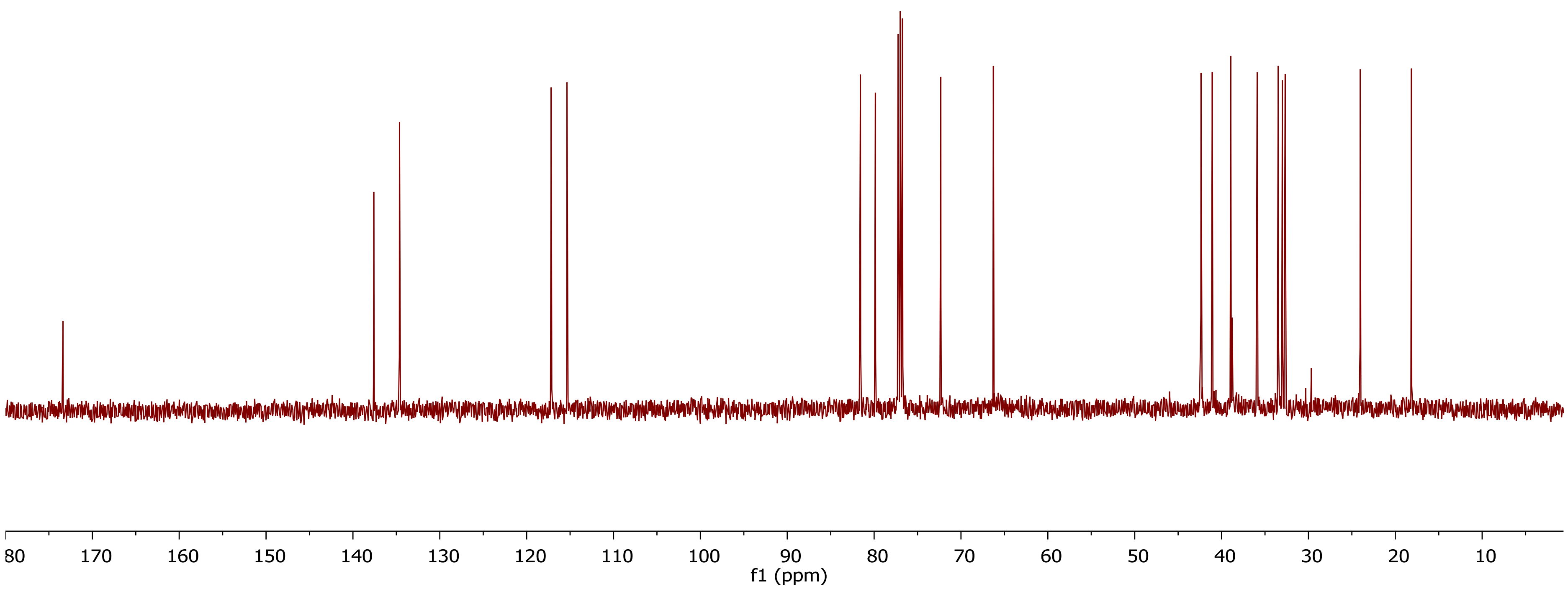

(1S,8E,11S,12R,14R,15R,17S)-14-hydroxy-15-methyl- 3,18-dioxatricyclo[9.6.1.0^{12,17}]octadec-8-en-4- one **39**

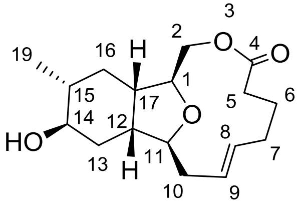

39

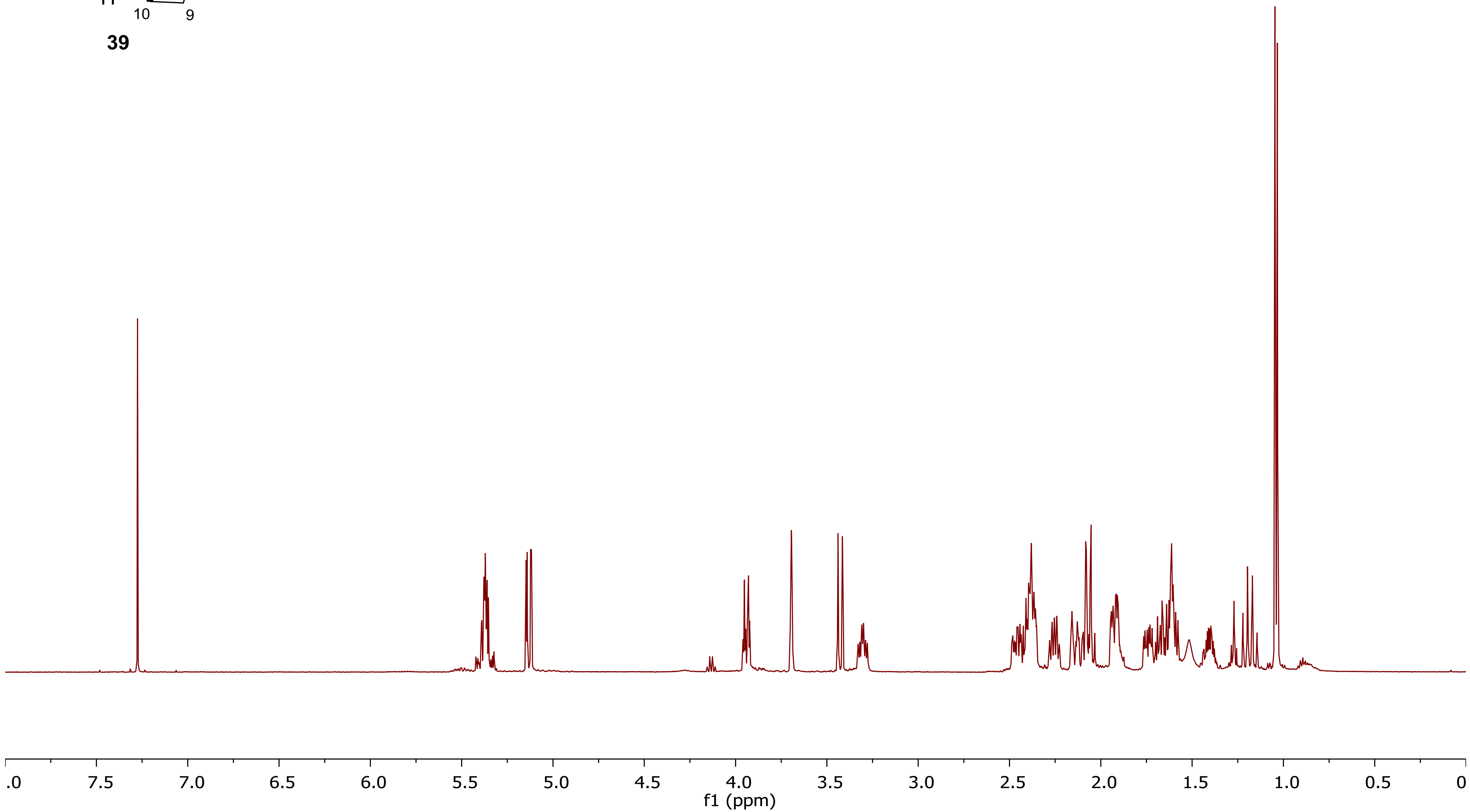

(1S,8E,11S,12R,14R,15R,17S)-14-hydroxy-15-methyl- 3,18-dioxatricyclo[9.6.1.0^{12,17}]octadec-8-en-4- one **39**

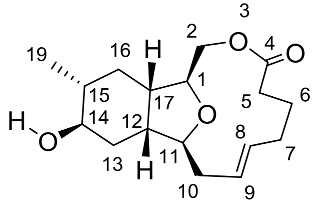

39

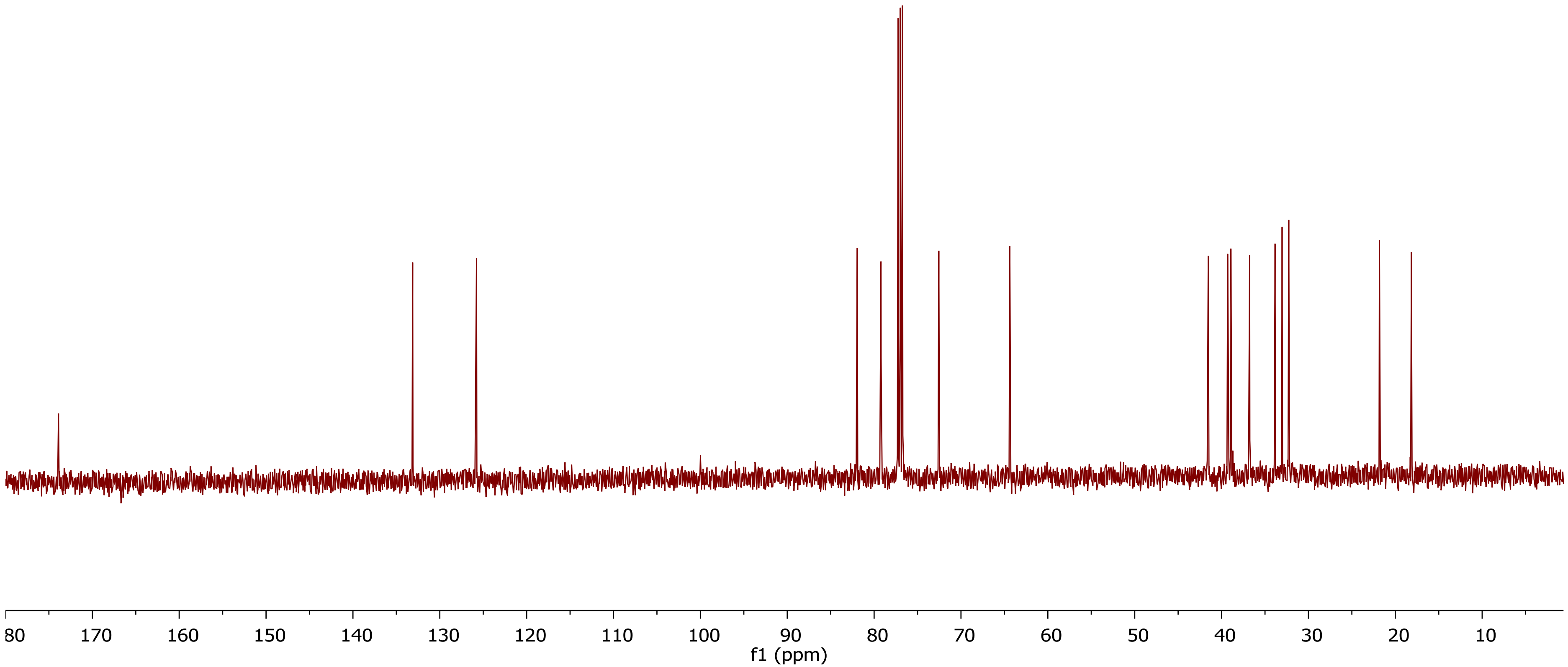

(1S,2R,5S,6S,7R)-5-(*tert*-Butyldiphenylsilanyloxymethyl)-4-oxa-tricyclo[5.2.2.0^{2,6}]undec-8-en-3-one **40a**

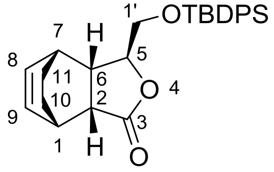

40a

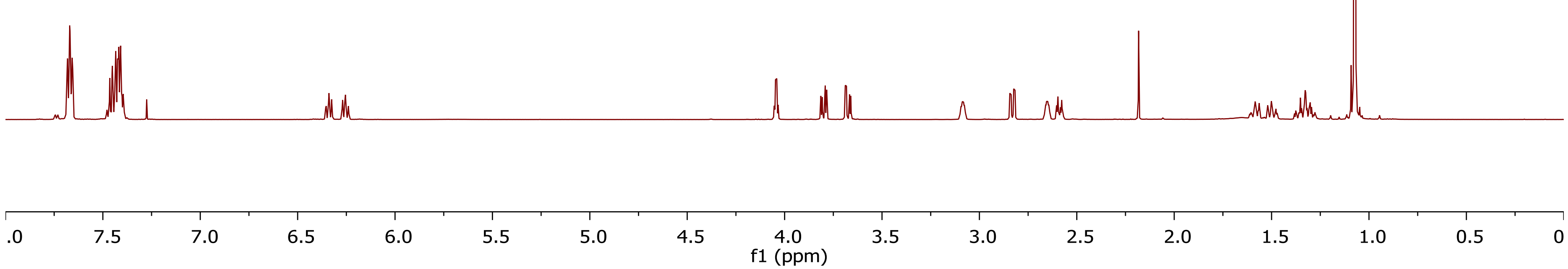

(1S,2R,5S,6S,7R)-5-(*tert*-Butyldiphenylsilanyloxymethyl)-4-oxa-tricyclo[5.2.2.0^{2,6}]undec-8-en-3-one **40a**

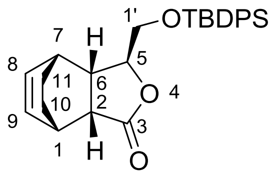

40a

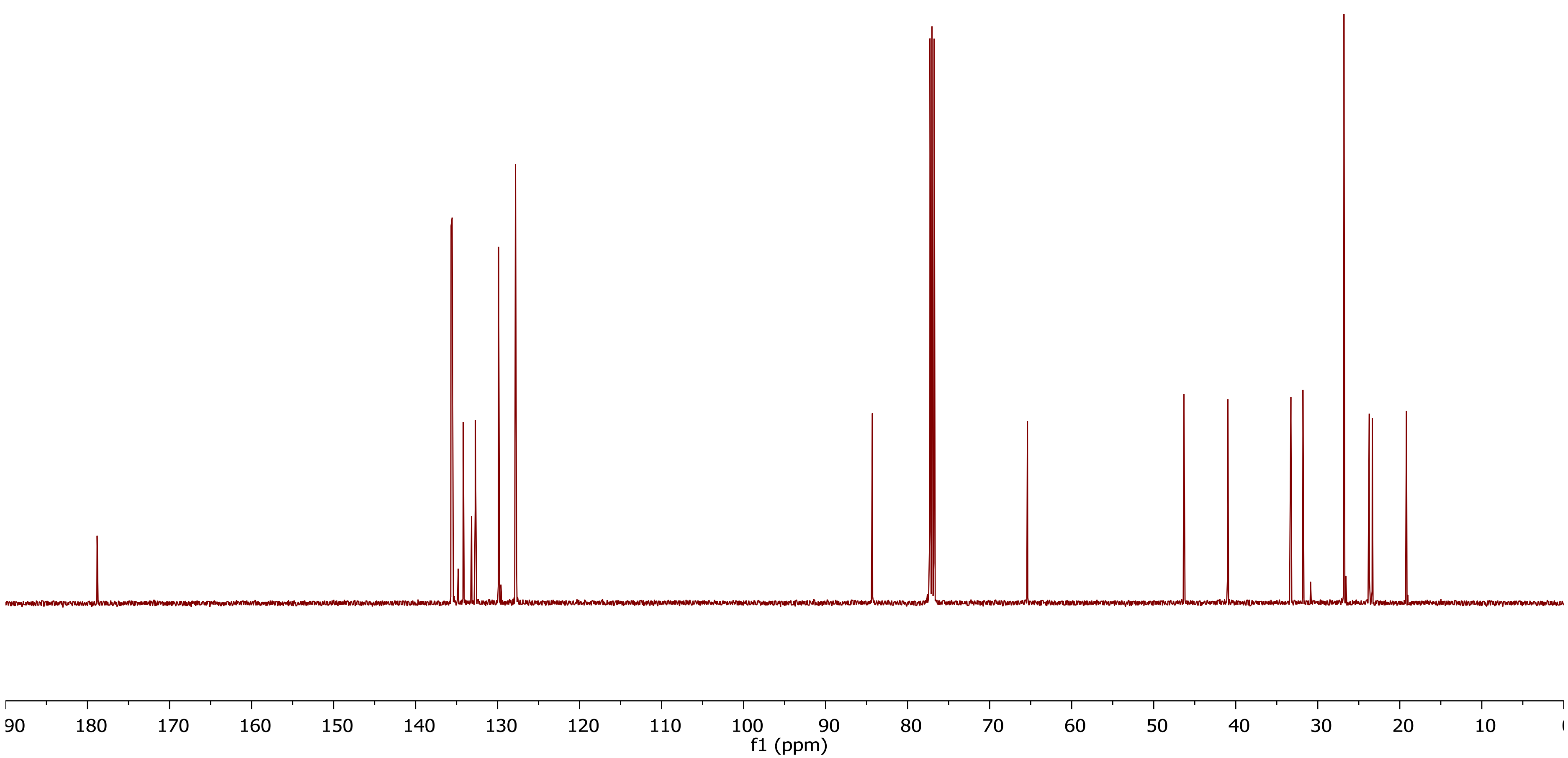

(1S,2R,5S,6S,7R)-5-(*tert*-Butyldiphenylsilanyloxymethyl)-4-oxa-tricyclo[5.2.2.0^{2,6}]undec-8-en-3-ol **40b**

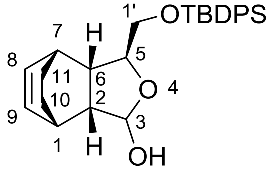

40b

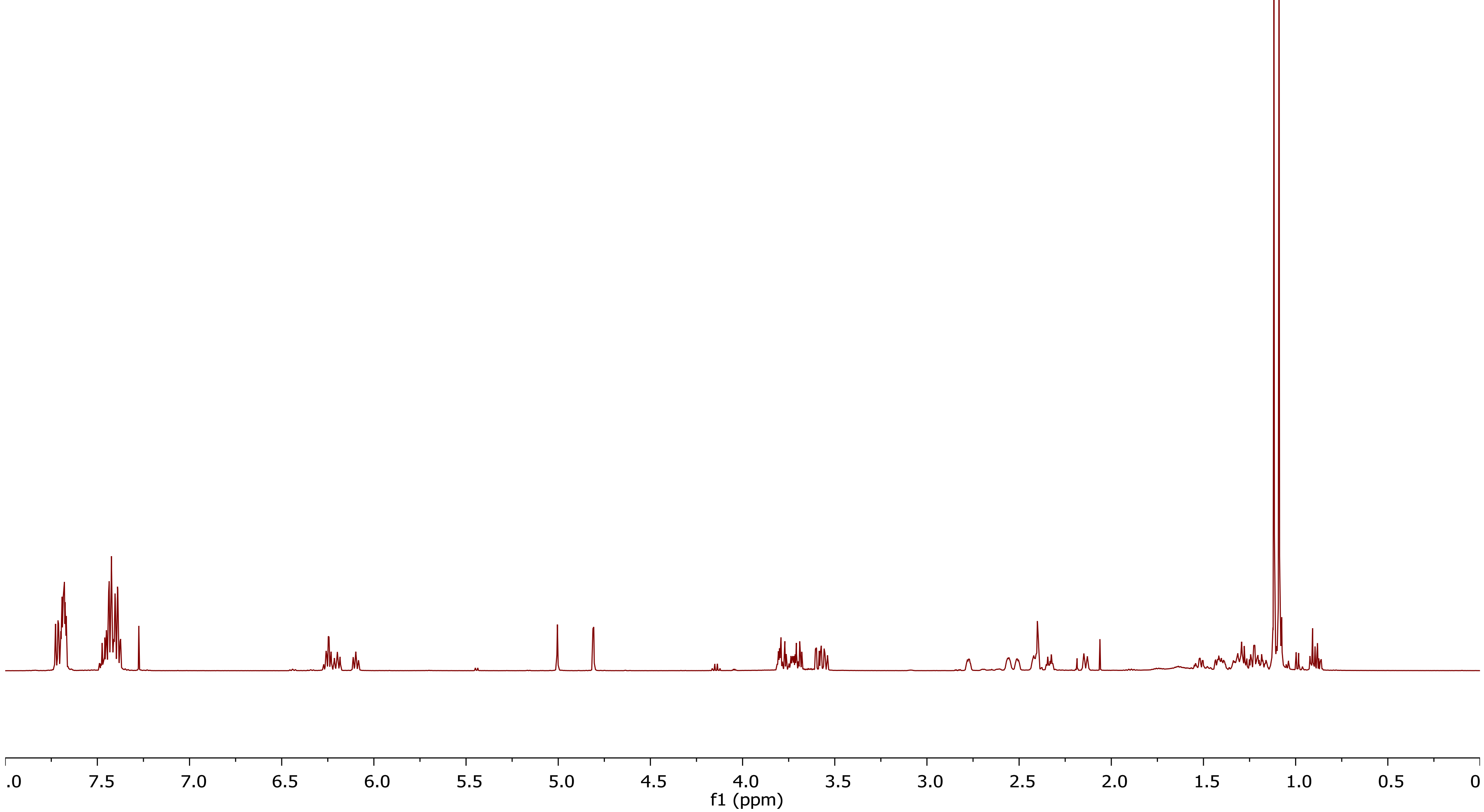

(1S,2R,5S,6S,7R)-5-(*tert*-Butyldiphenylsilanyloxymethyl)-4-oxa-tricyclo[5.2.2.0^{2,6}]undec-8-en-3-ol **40b**

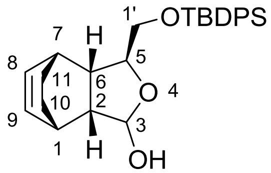

40b

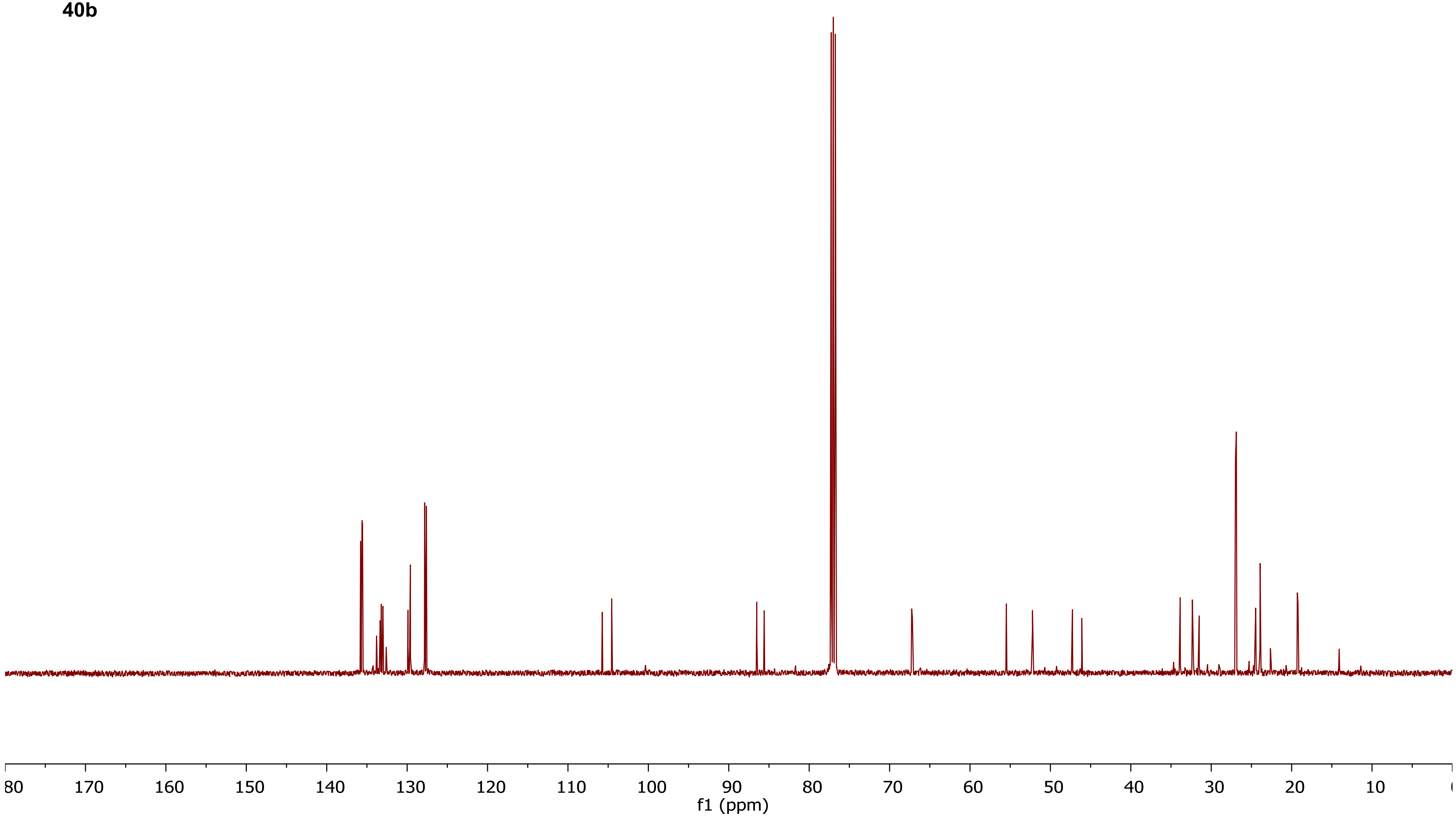

((1R, 2S, 3S, 5S, 6R, 7S)-5-allyl-4-oxatricyclo[5.2.2.0^{2,6}]undec-8-en-3-ylmethoxy)*tert*butyl diphenylsilane **40c**

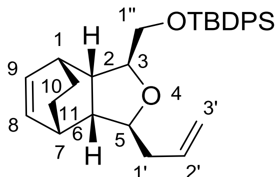

40c

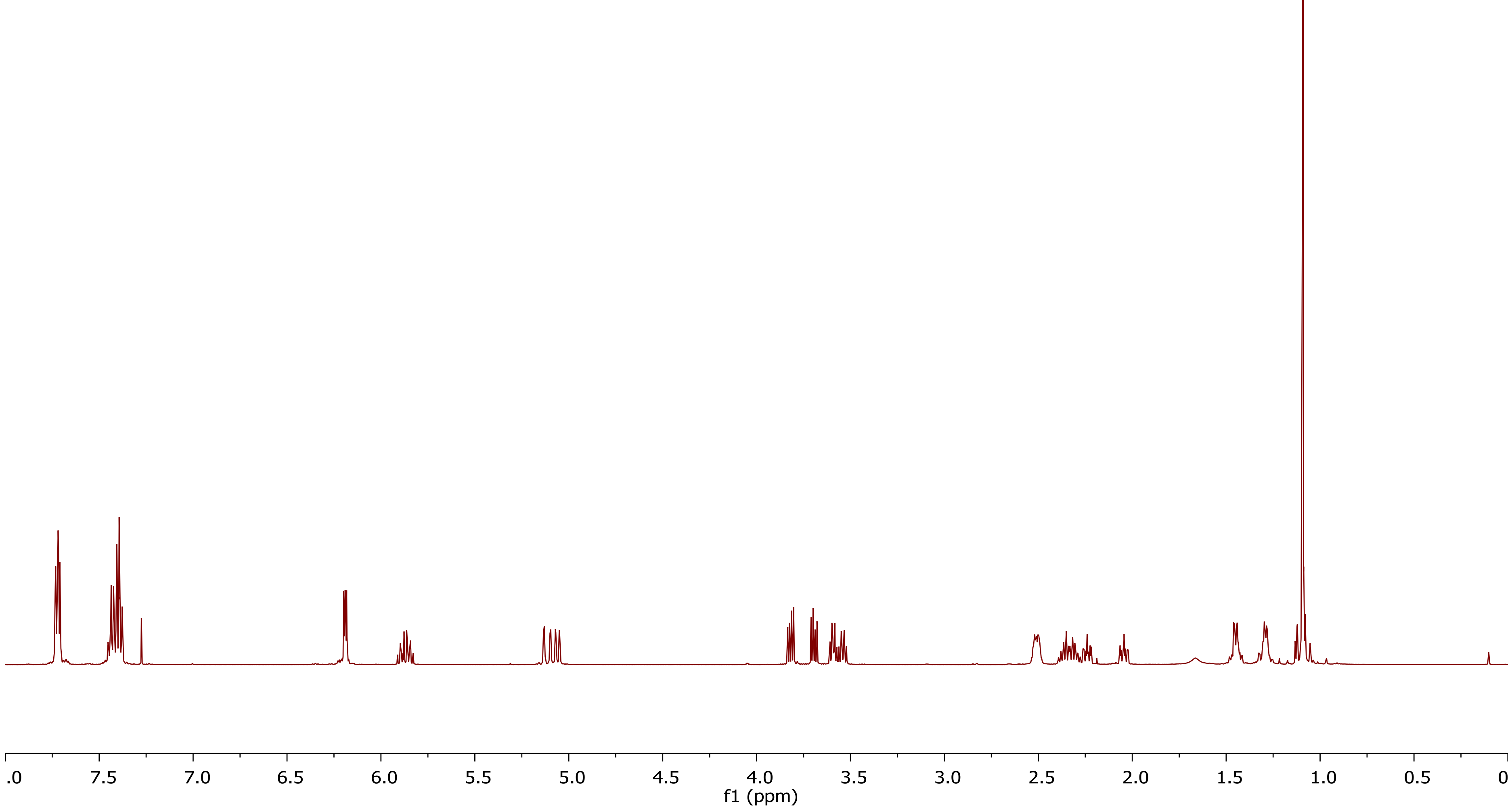

((1R, 2S, 3S, 5S, 6R, 7S)-5-allyl-4-oxatricyclo[5.2.2.0^{2,6}]undec-8-en-3-ylmethoxy)*tert*butyl diphenylsilane **40c**

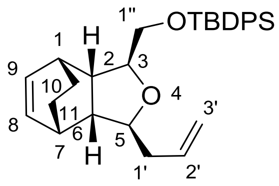

40c

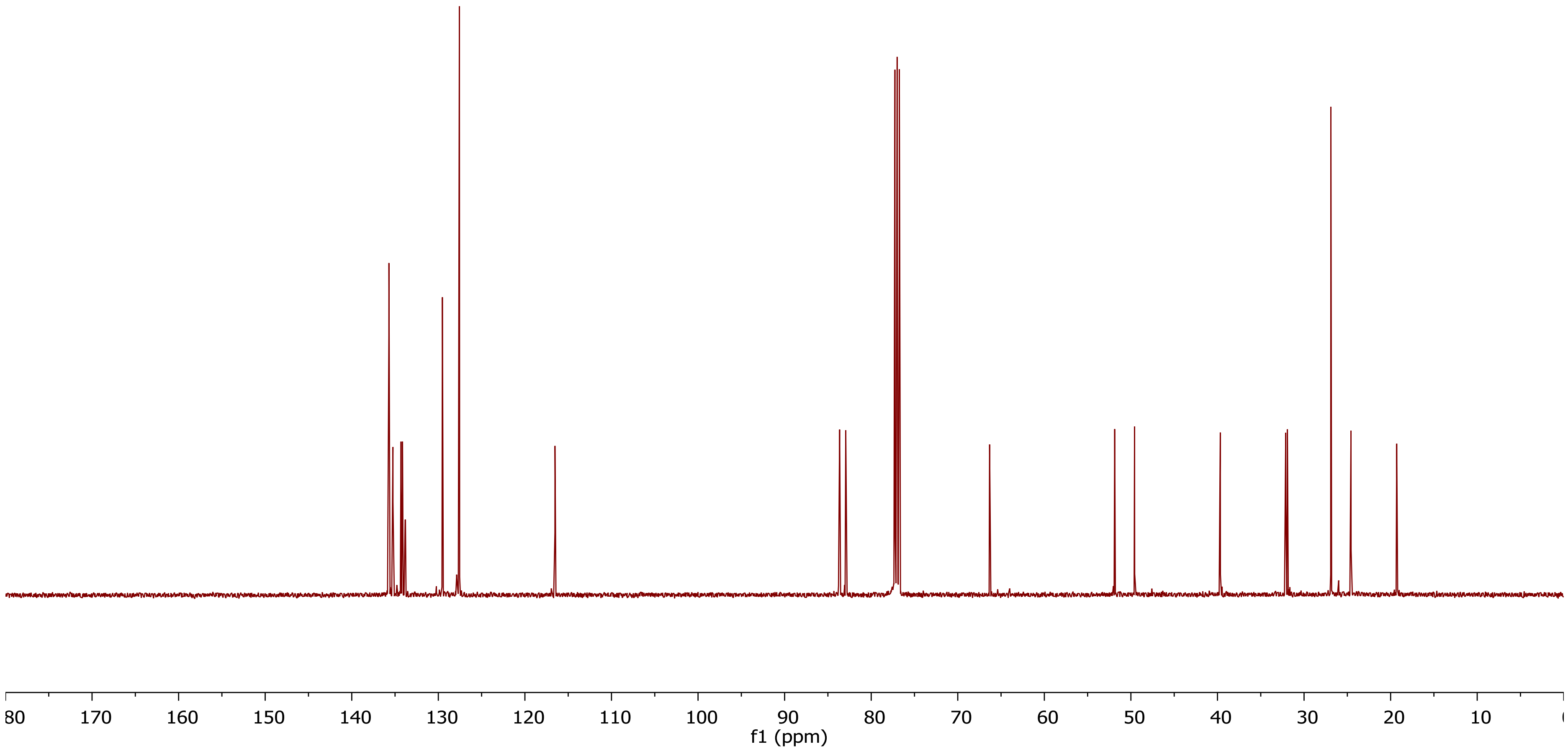

((1R, 2S, 3S, 5S, 6R, 7S)-5-allyl-4-oxatricyclo[5.2.2.0^{2,6}]undec-8-en-3-yl)-methanol **84**

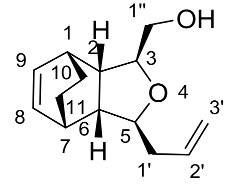

84

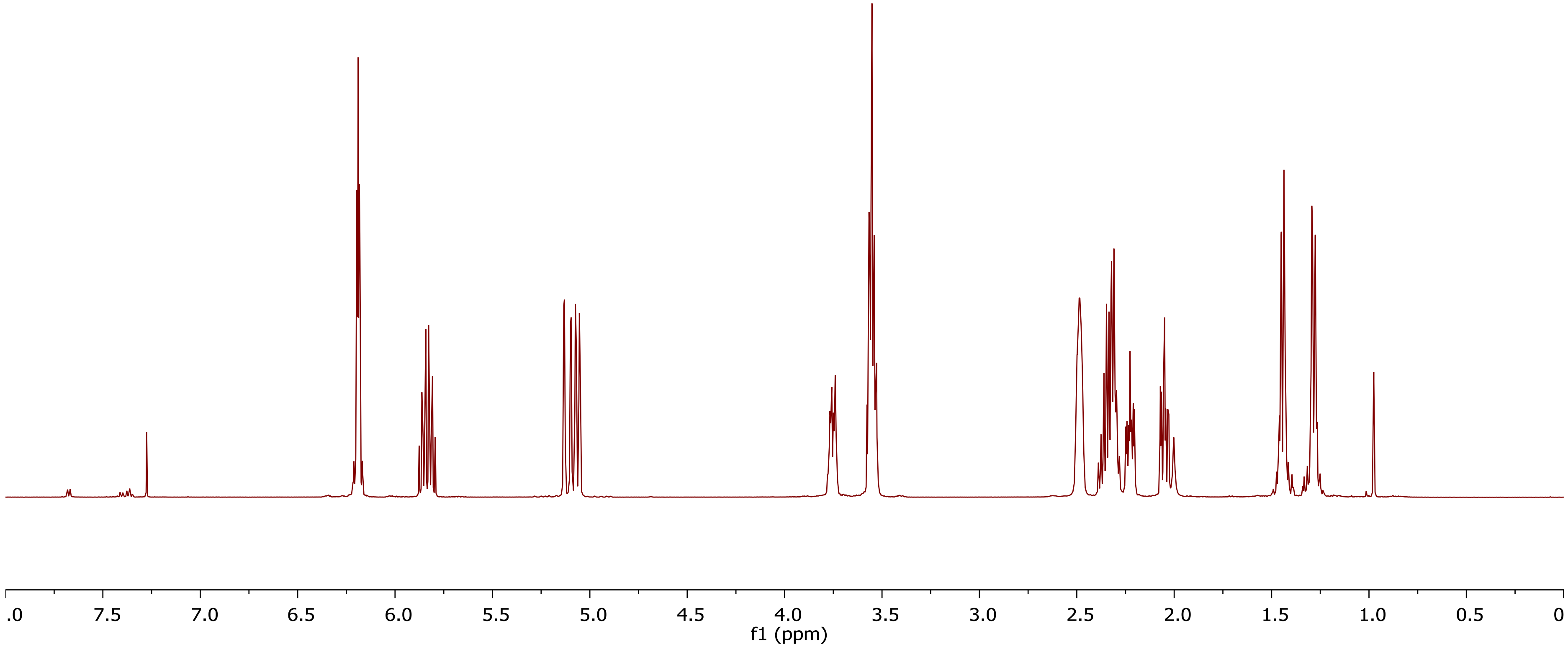

((1R, 2S, 3S, 5S, 6R, 7S)-5-allyl-4-oxatricyclo[5.2.2.0$^{2.6}$]undec-8-en-3-yl)-methanol **84**

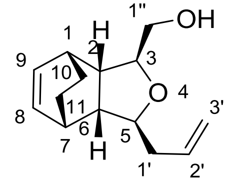

84

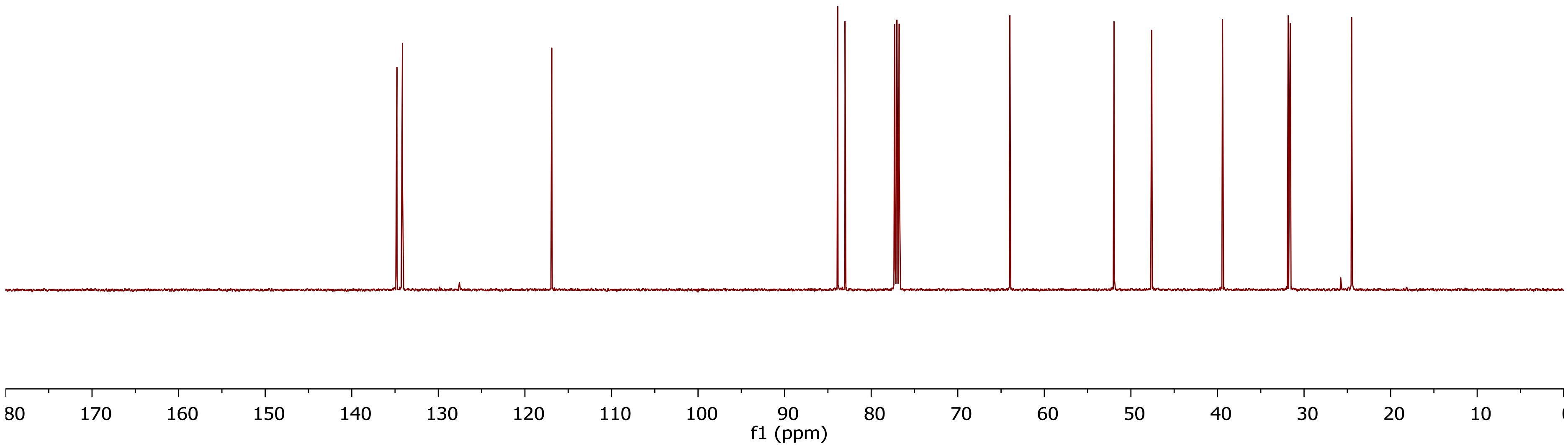

(1R,2S,3S,5S,6R,7S)-5-Allyl-4-oxatricyclo[5.2.2.$0^{2,6}$]undec-8-en-3-ylmethyl hex-5-enoate **85**

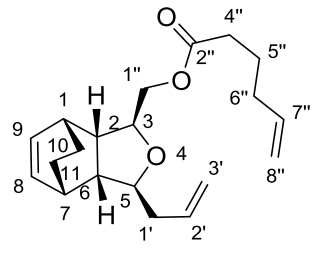

85

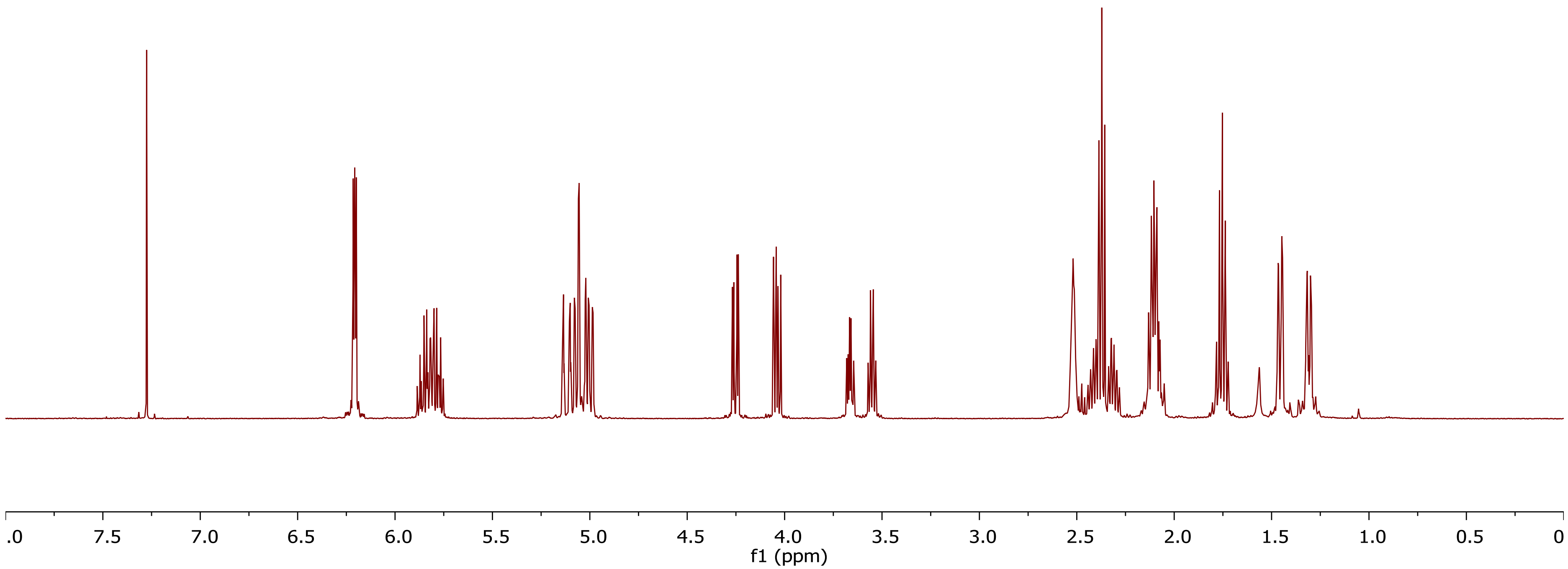

(1R,2S,3S,5S,6R,7S)-5-Allyl-4-oxatricyclo[5.2.2.0^{2,6}]undec-8-en-3-ylmethyl hex-5-enoate **85**

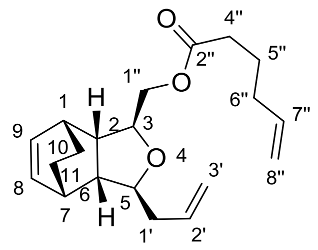

85

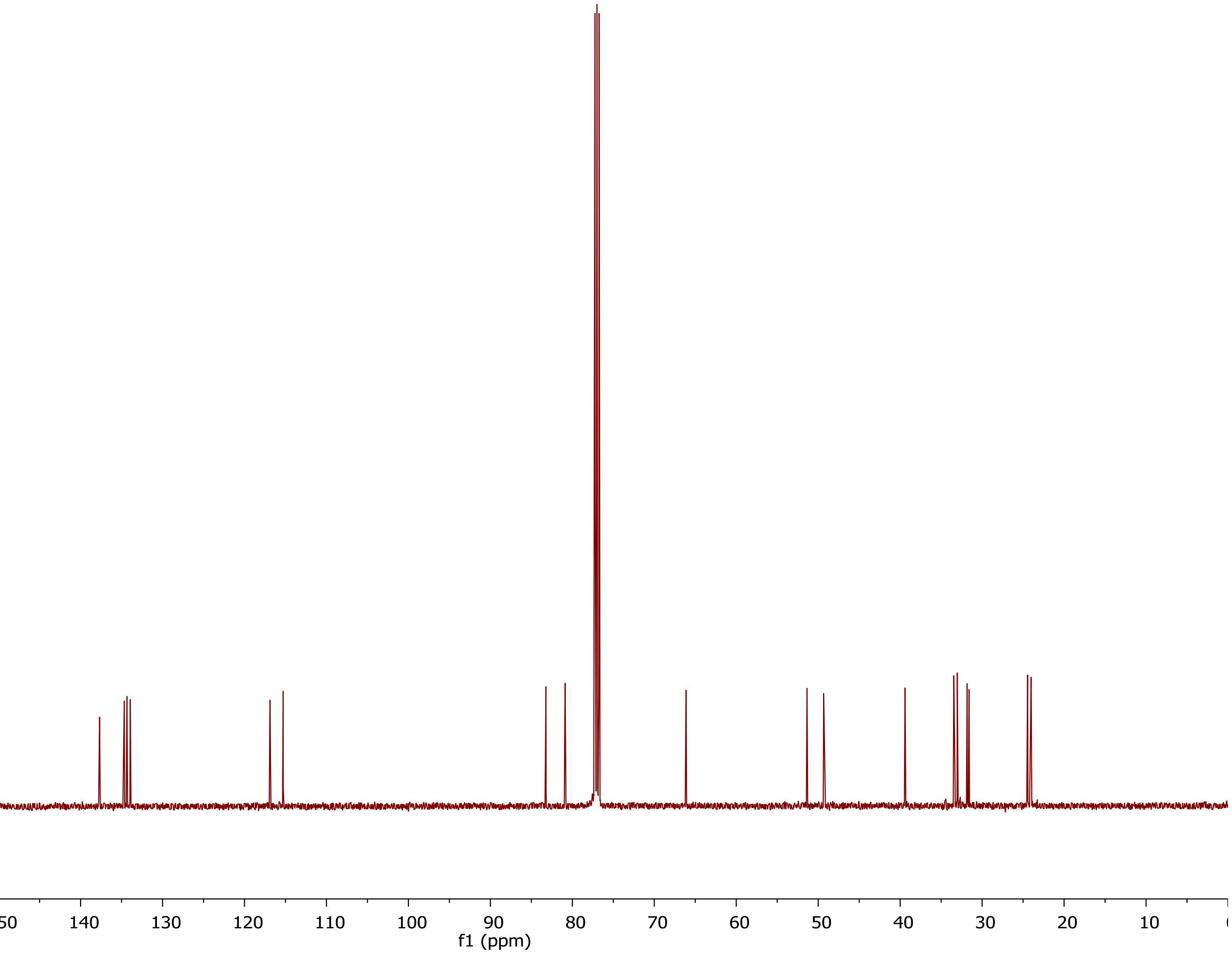

(1R,2S,3S,10E,13S,14R,15S)-5,20- dioxatetracyclo[13.2.2.1^{3,13}.0^{2,14}]icosa- 10,16-dien-6-one **40**

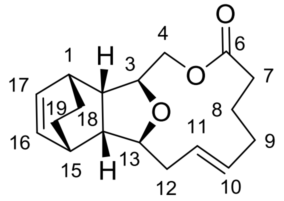

40

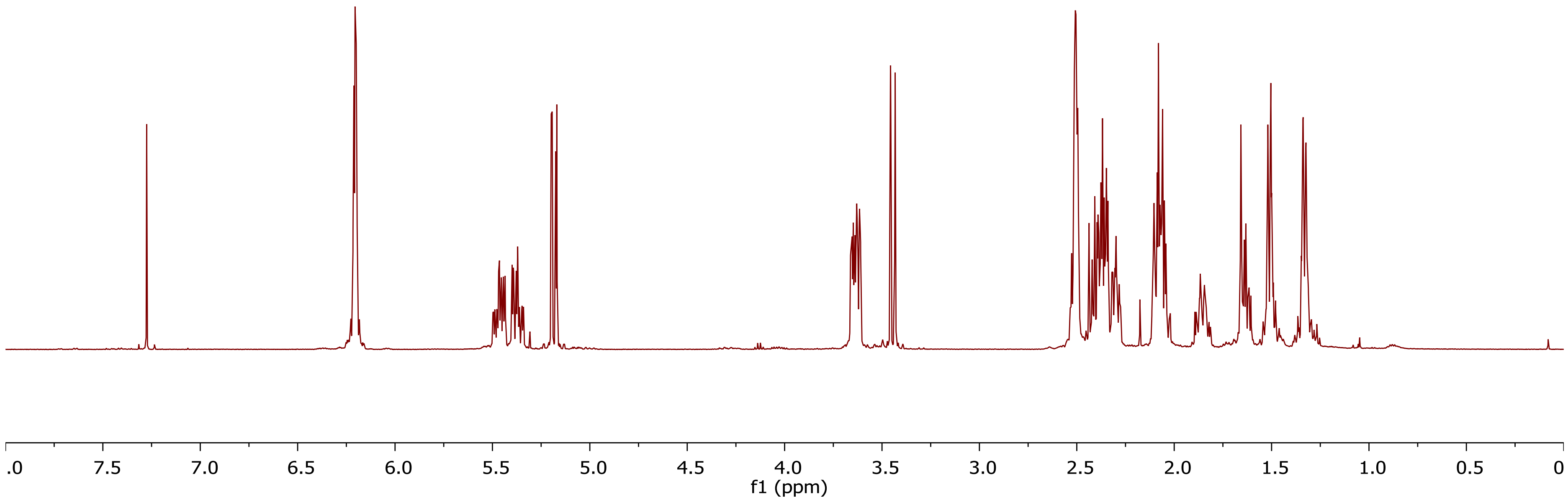

(1R,2S,3S,10E,13S,14R,15S)-5,20- dioxatetracyclo[13.2.2.1^{3,13}.0^{2,14}]icosa- 10,16-dien-6-one **40**

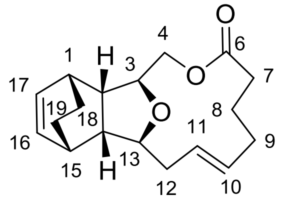

40

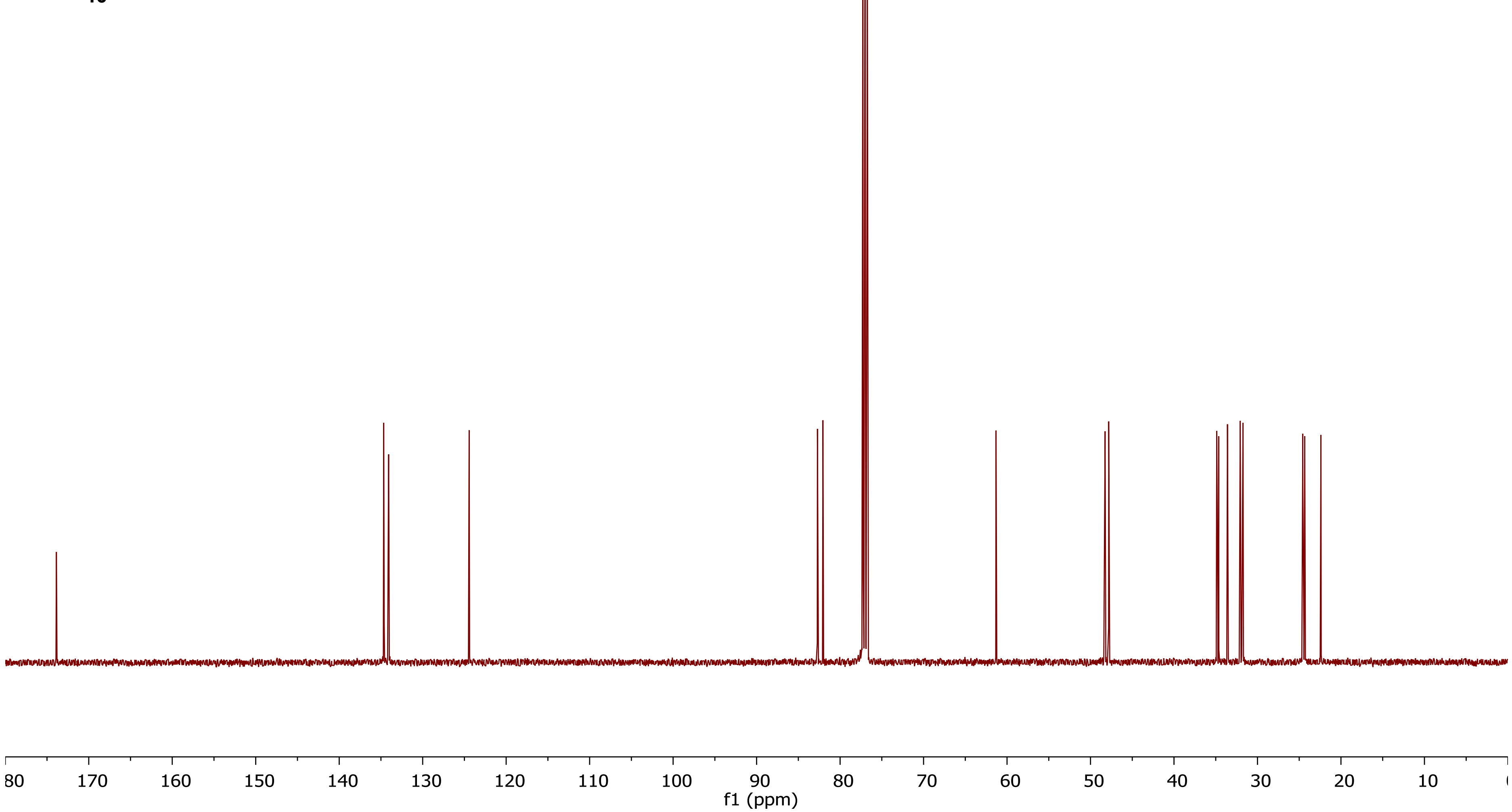

(3S,3aS,4R,7S,7aR)-3-(((tert-butyldiphenylsilyl)oxy)methyl)-3a,4,7,7a-tetrahydro-4,7-methanoisobenzofuran-1(3H)-one **41a**

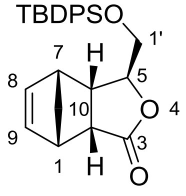

41a

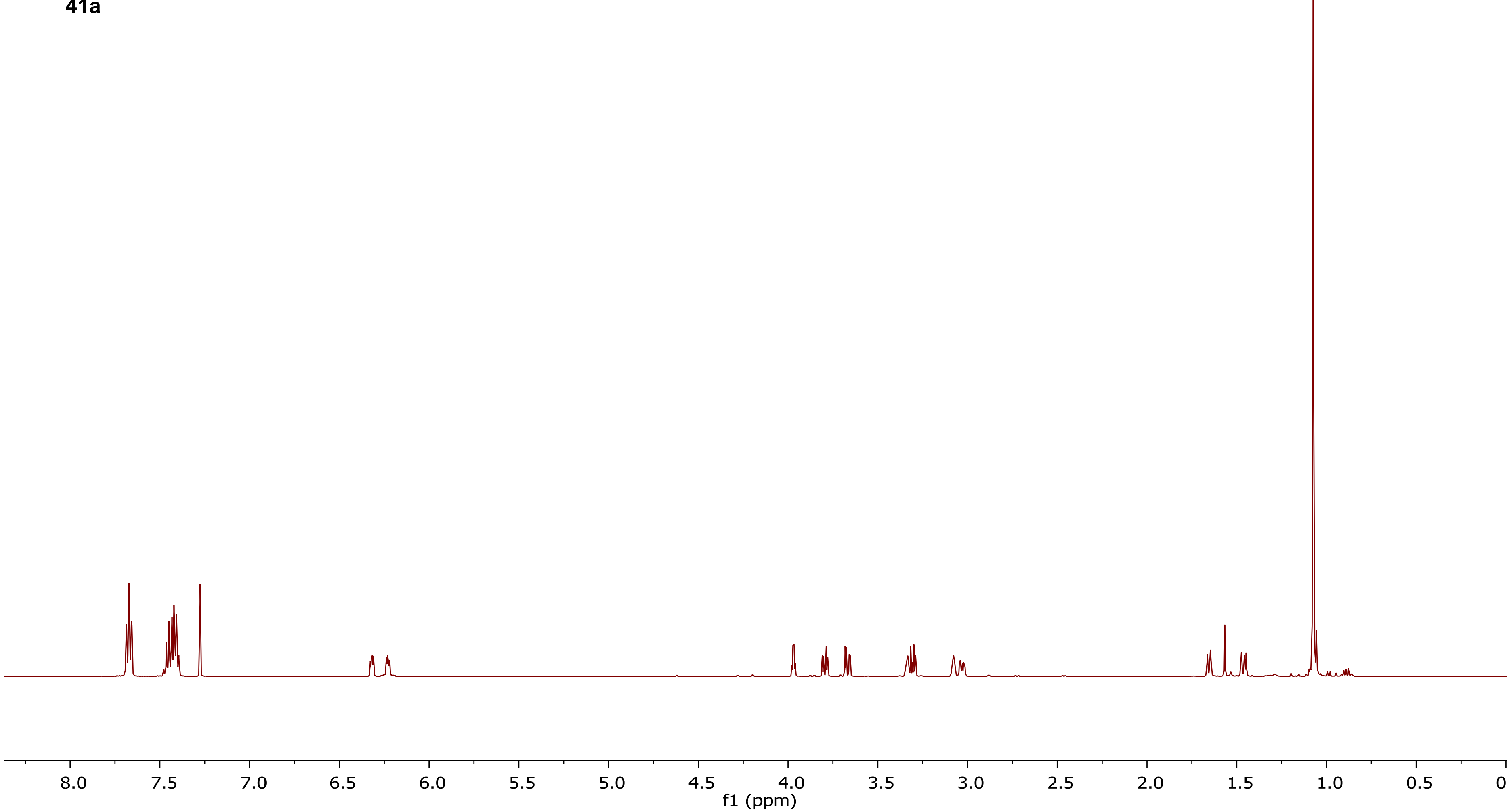

(3S,3aS,4R,7S,7aR)-3-(((tert-butyldiphenylsilyl)oxy)methyl)-3a,4,7,7a-tetrahydro-4,7-methanoisobenzofuran-1(3H)-one **41a**

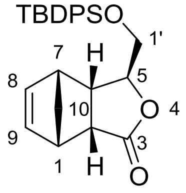

41a

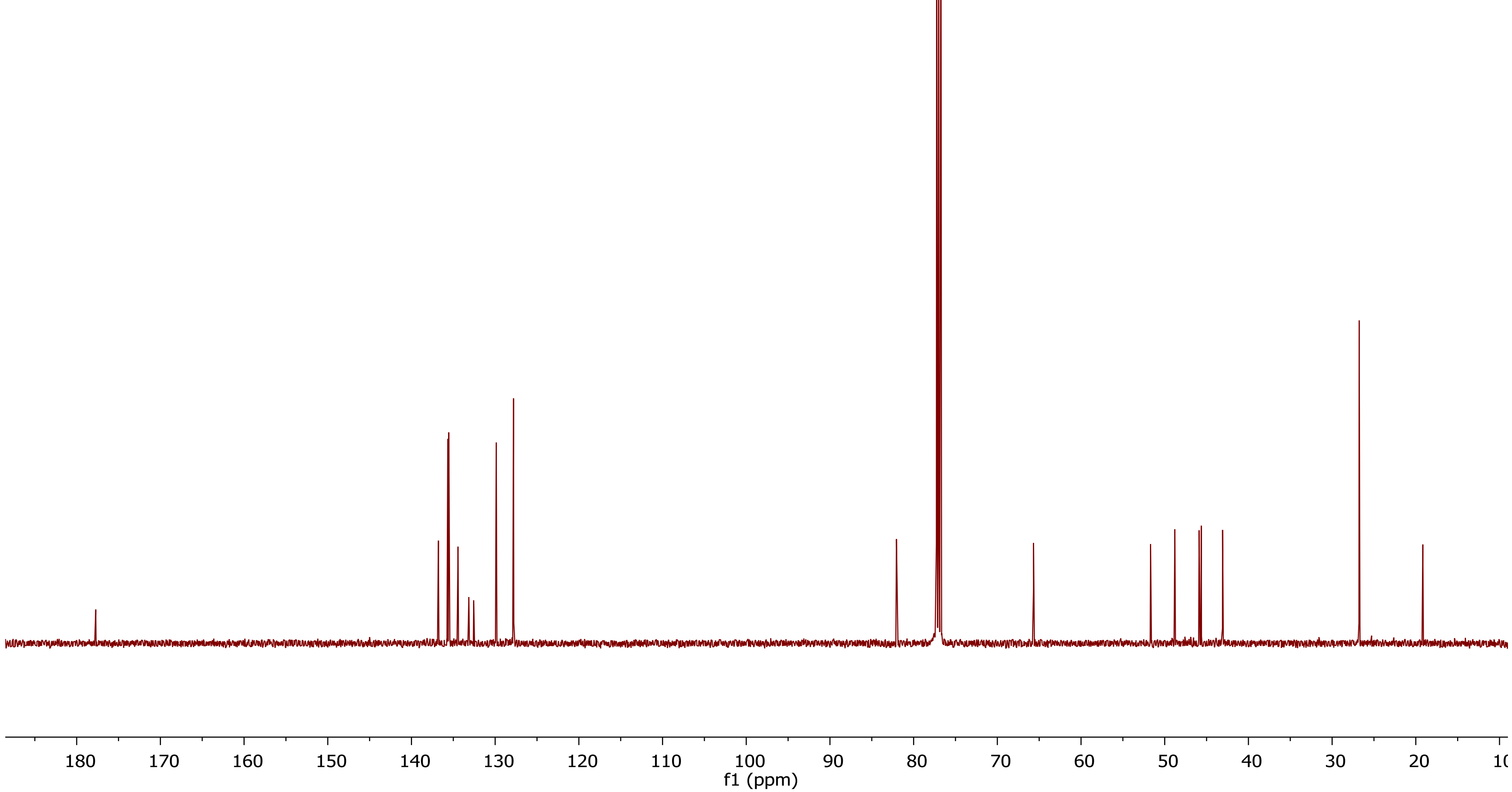

(1*S*,2*R*,5*S*,6*S*,7*R*)-5-(*tert*-Butyldiphenylsilanoxymethyl)-4-oxa-tricyclo[5.2.1.0.[2,6]]decan-3-one **41b**

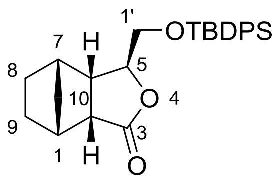

41b

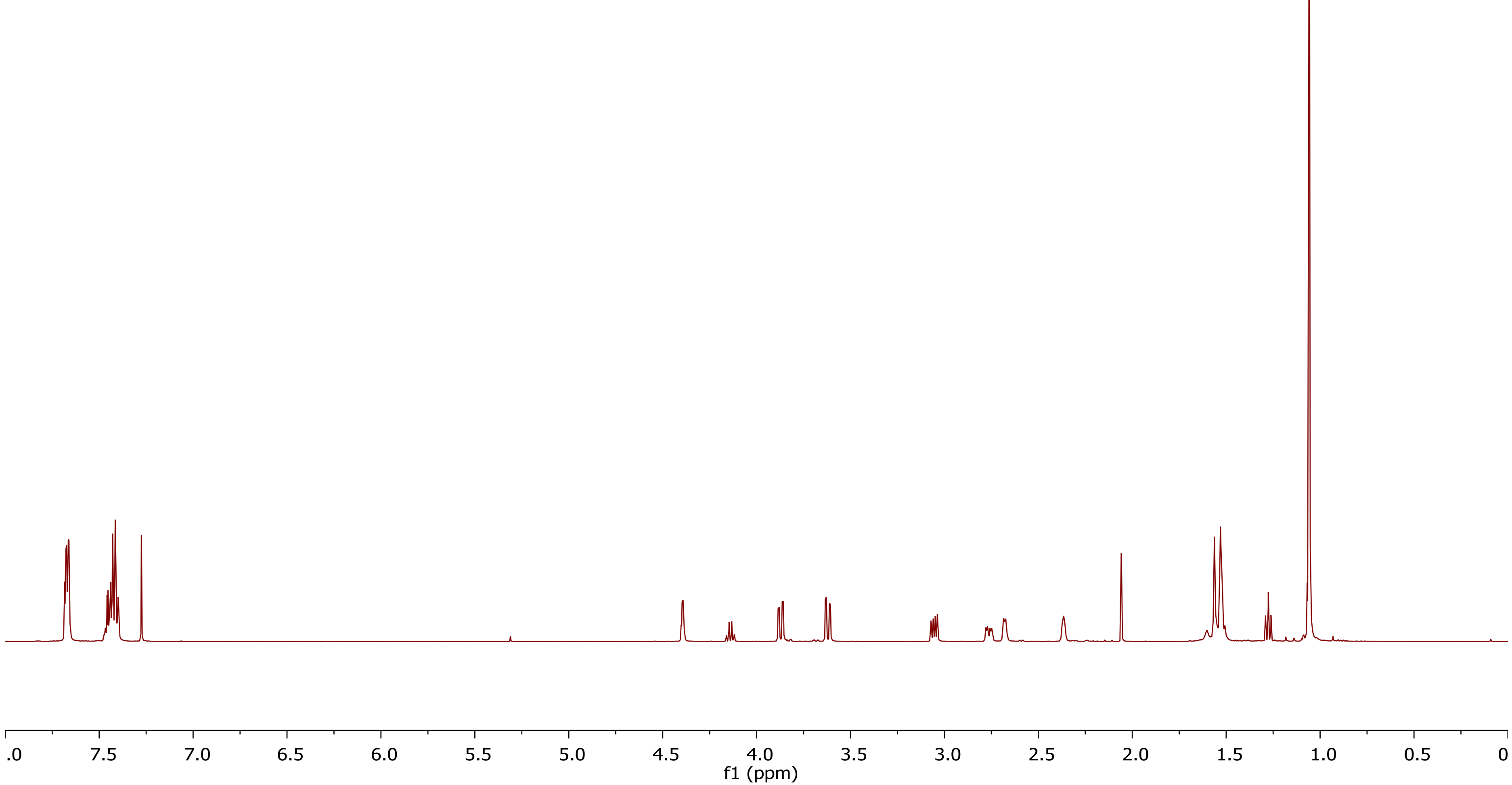

(1*S*,2*R*,5*S*,6*S*,7*R*)-5-(*tert*-Butyldiphenylsilanoxymethyl)-4-oxa-tricyclo[5.2.1.0.[2,6]]decan-3-one **41b**

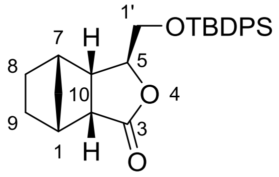

41b

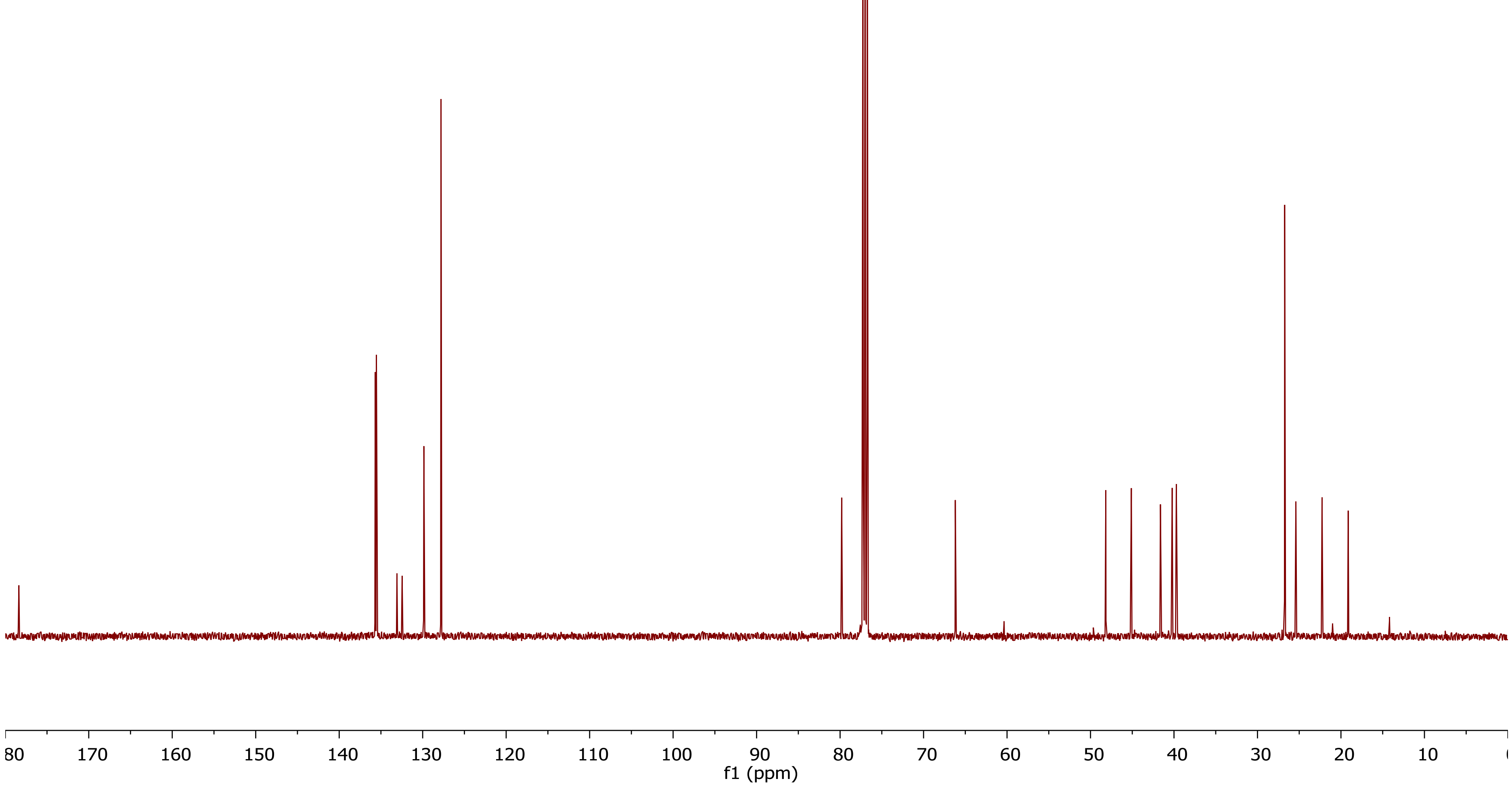

(1*S*,2*R*,5*S*,6*S*,7*R*)-5-(*tert*-Butyldiphenylsilanoxymethyl)-4-oxa-tricyclo[5.2.1.0.[2,6]]decan-3-ol **41c**

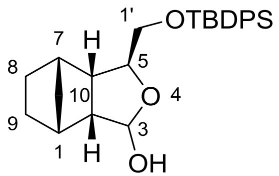

41c

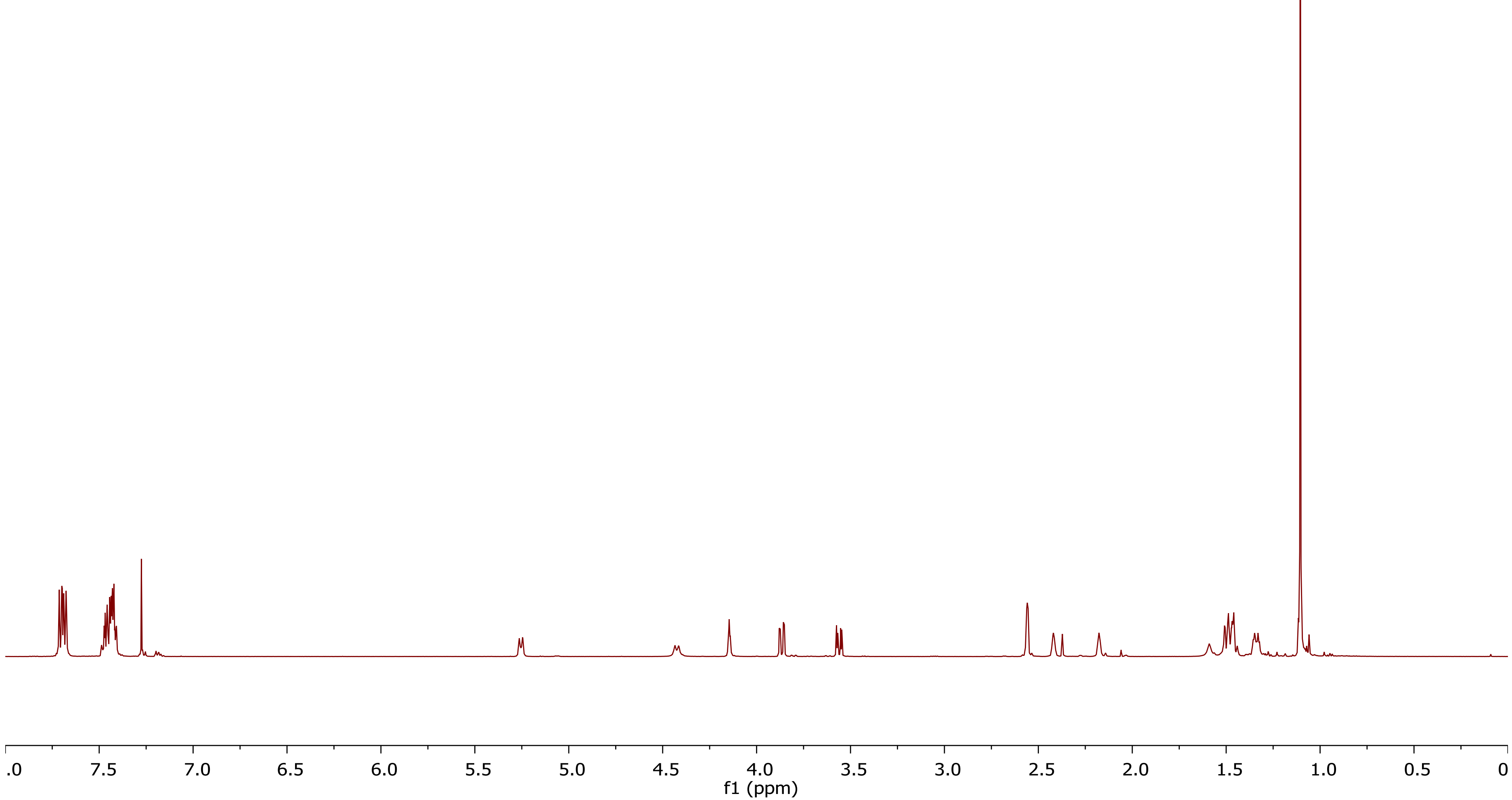

(1*S*,2*R*,5*S*,6*S*,7*R*)-5-(*tert*-Butyldiphenylsilanoxymethyl)-4-oxa-tricyclo[5.2.1.0.^{2,6}]decan-3-ol **41c**

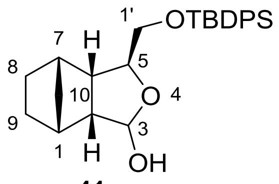

41c

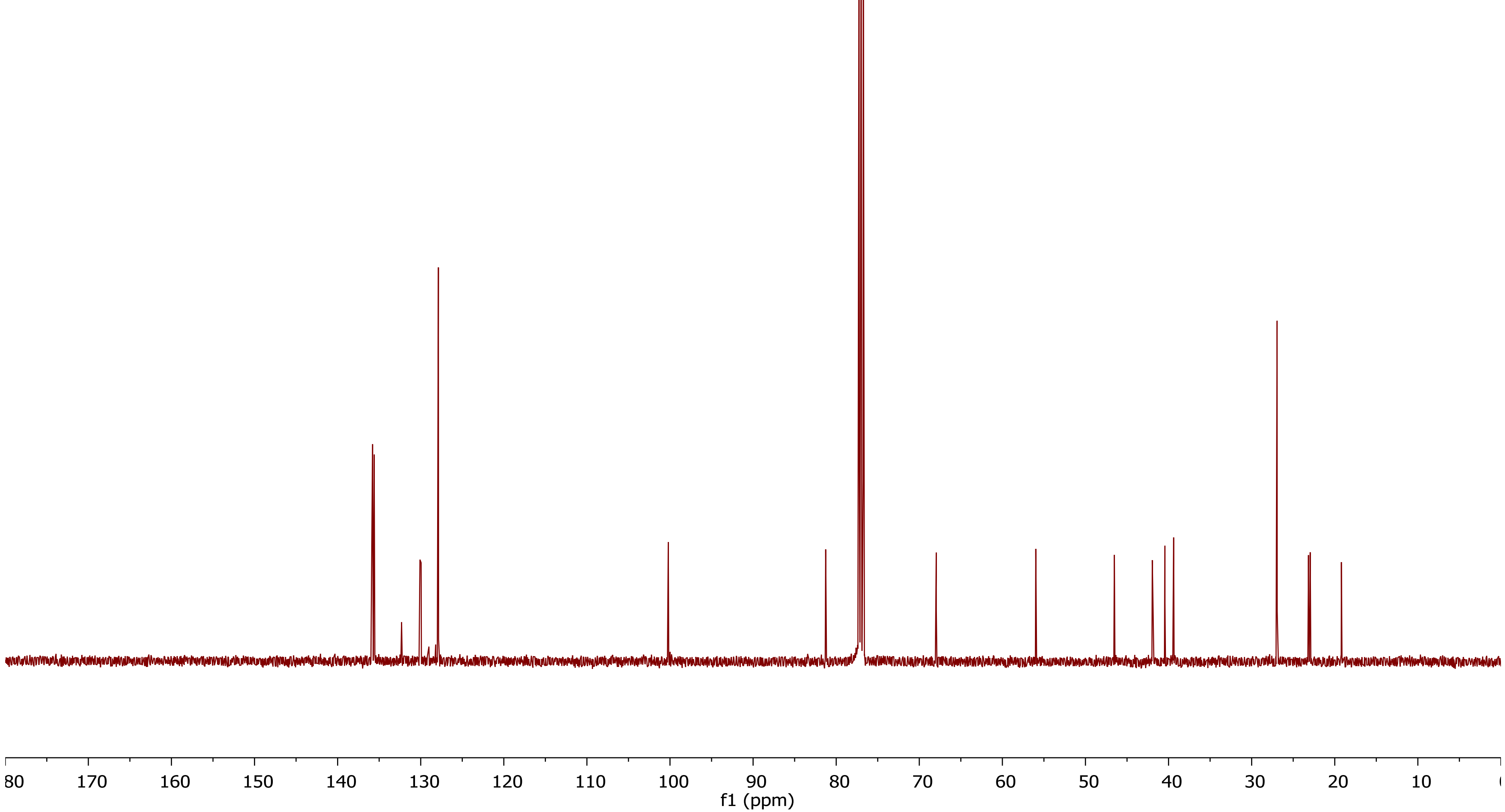

((1*R*,2*S*,3*S*,5*S*,6*R*,7S)-5-Allyl-4-oxa-tricyclo[5.2.1.0^{2,6}]decane-3-ylmethoxy-*tert*-butyldiphenylsilane **41d**

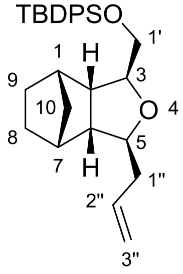

41d

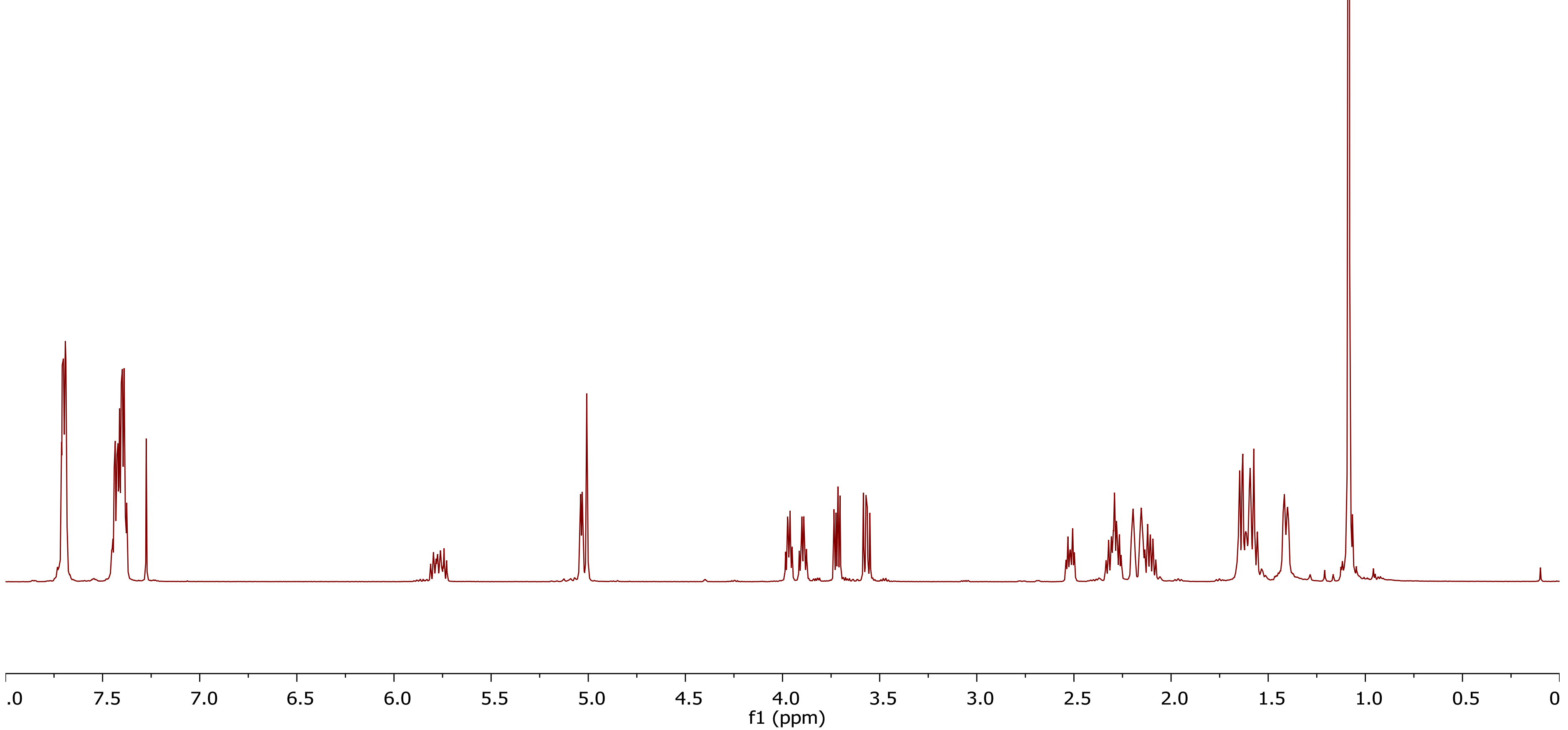

((1*R*,2*S*,3*S*,5*S*,6*R*,7S)-5-Allyl-4-oxa-tricyclo[5.2.1.0^{2,6}]decane-3-ylmethoxy-*tert*-butyldiphenylsilane **41d**

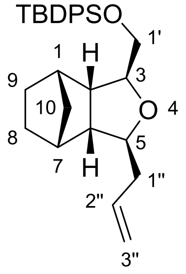

41d

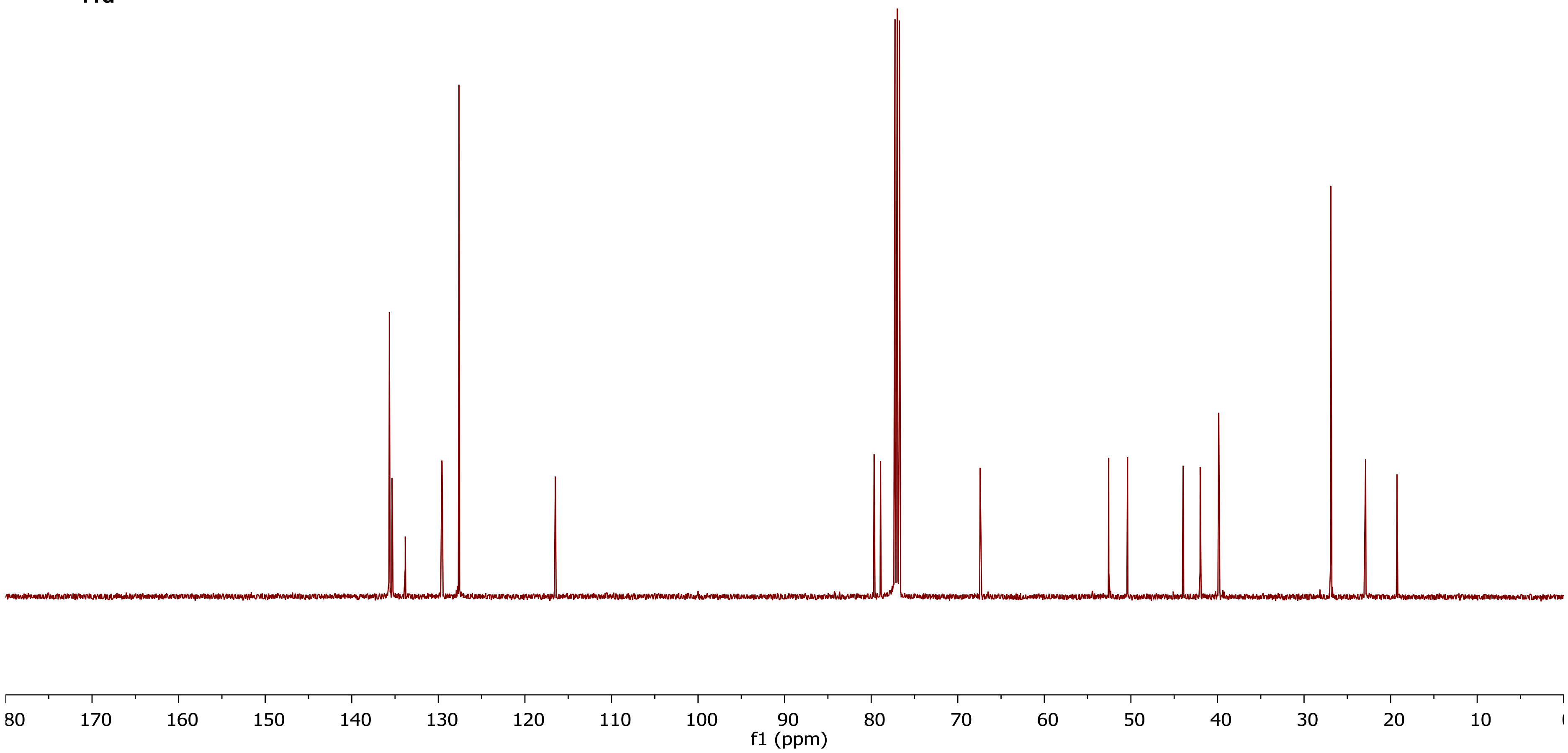

((1R,2S,3S,5S,6R,7S)-5-Allyl-4-oxa-tricyclo[5.2.1.02,6]decan-3-yl)-methanol **41e**

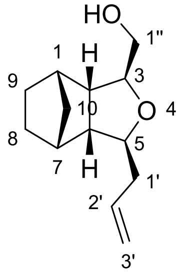

41e

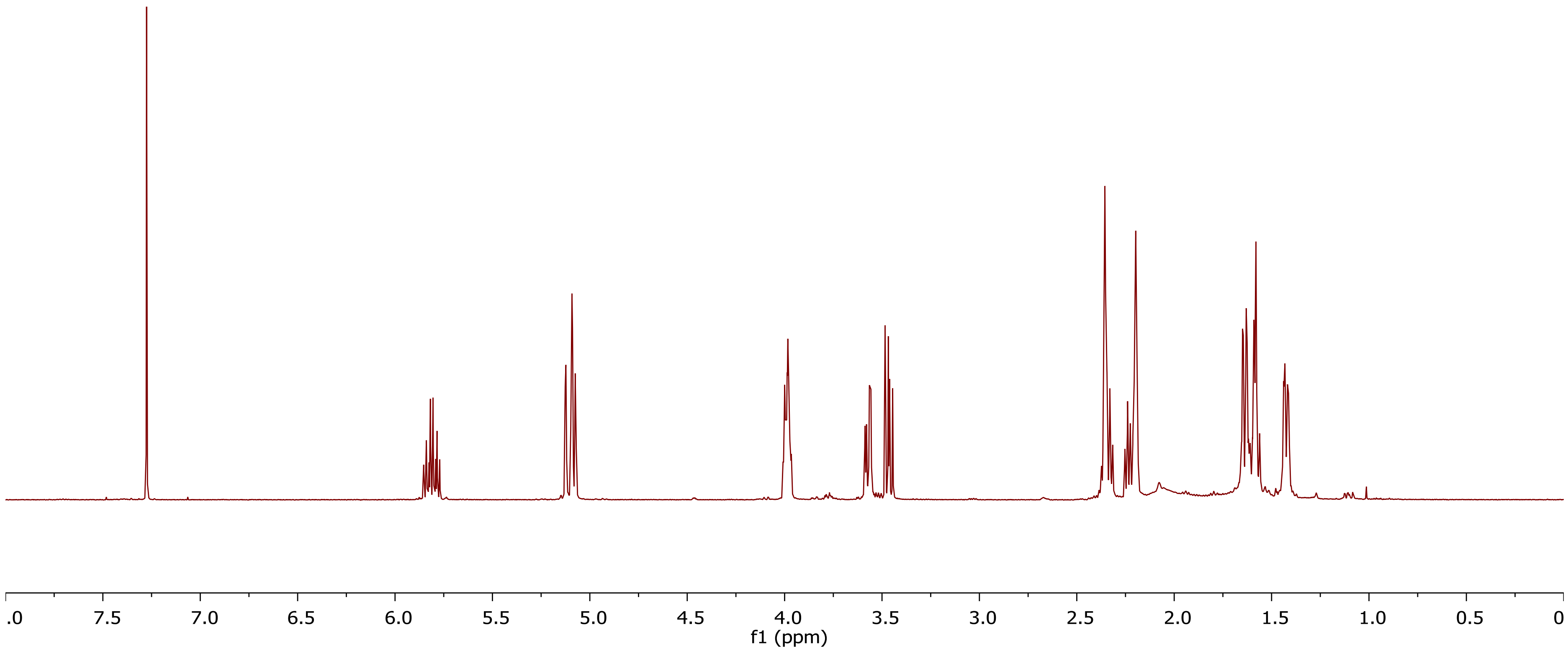

((1R,2S,3S,5S,6R,7S)-5-Allyl-4-oxa-tricyclo[5.2.1.02,6]decan-3-yl)-methanol **41e**

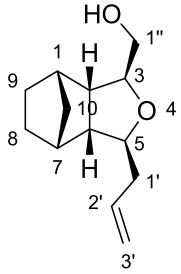

41e

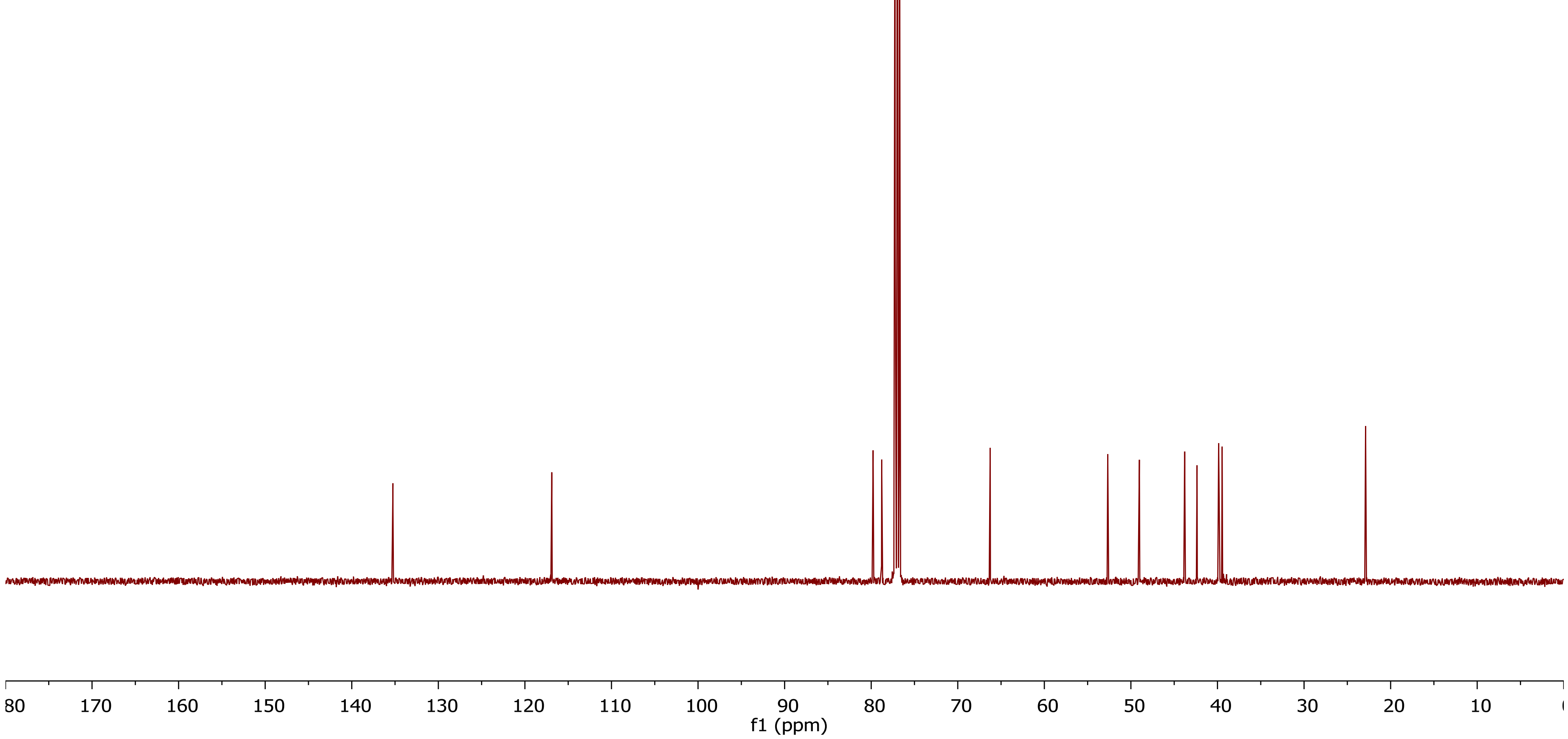

((1S,3S,3aR,4R,7S,7aS)-3-allyloctahydro-4,7-methanoisobenzofuran-1-yl)methyl pent-4-enoate **41f**

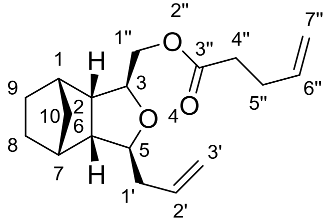

41f

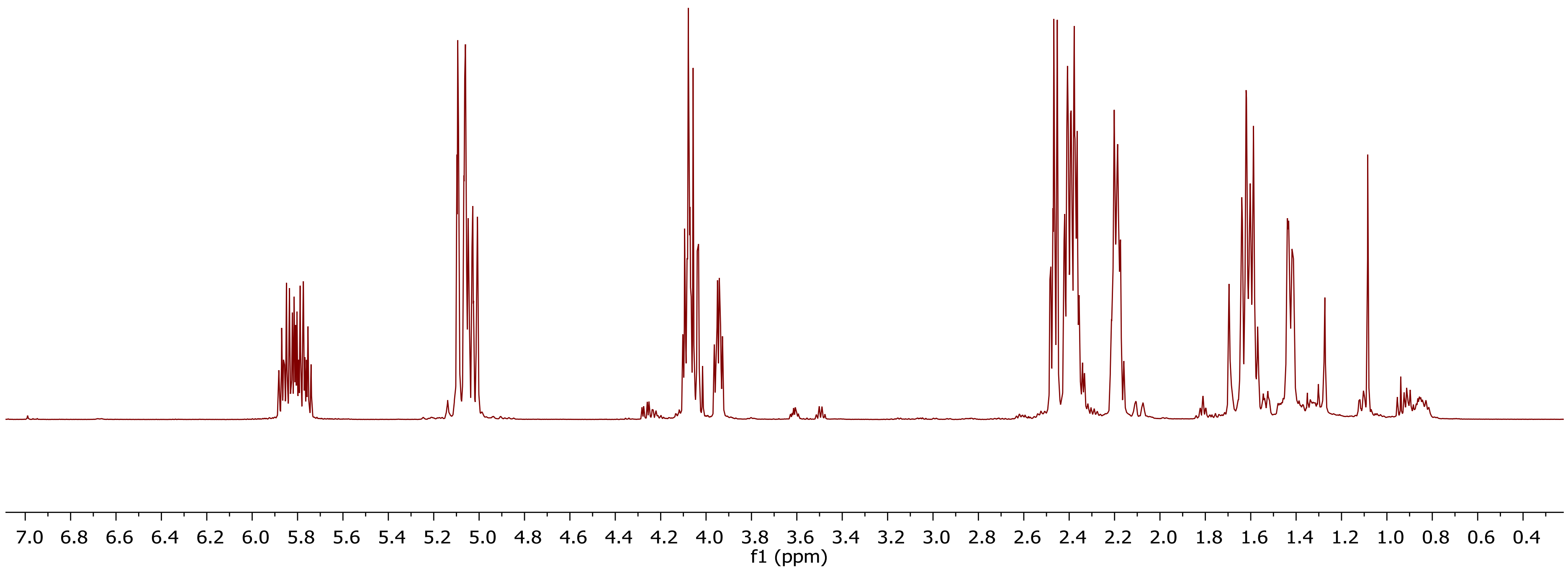

((1S,3S,3aR,4R,7S,7aS)-3-allyloctahydro-4,7-methanoisobenzofuran-1-yl)methyl pent-4-enoate **41f**

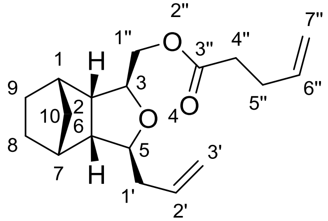

41f

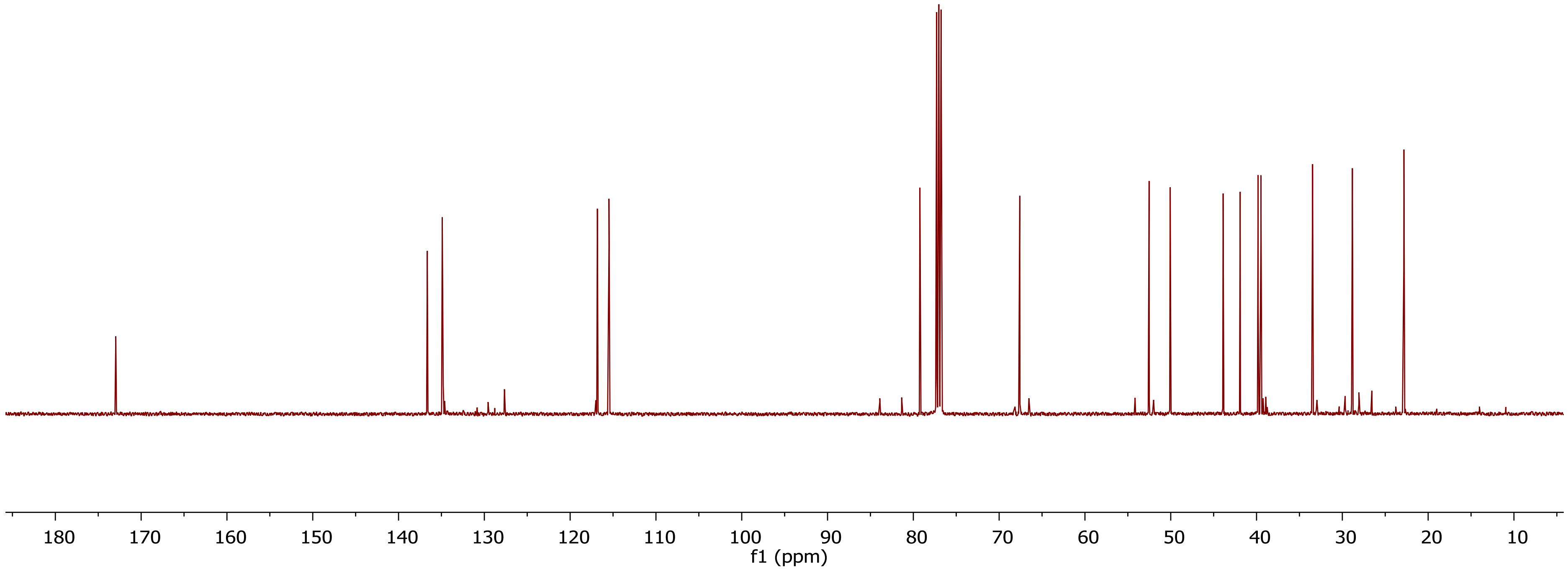

(1S,10S,10aR,11R,14S,14aS)-1,2,5,6,9,10,10a,11,12,13,14,14a-dodecahydro-4H-1,10-epoxy-11,14-methanobenzo[d][1]oxacyclododecin-4-one **41**

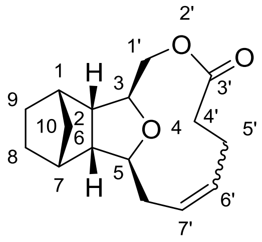

41

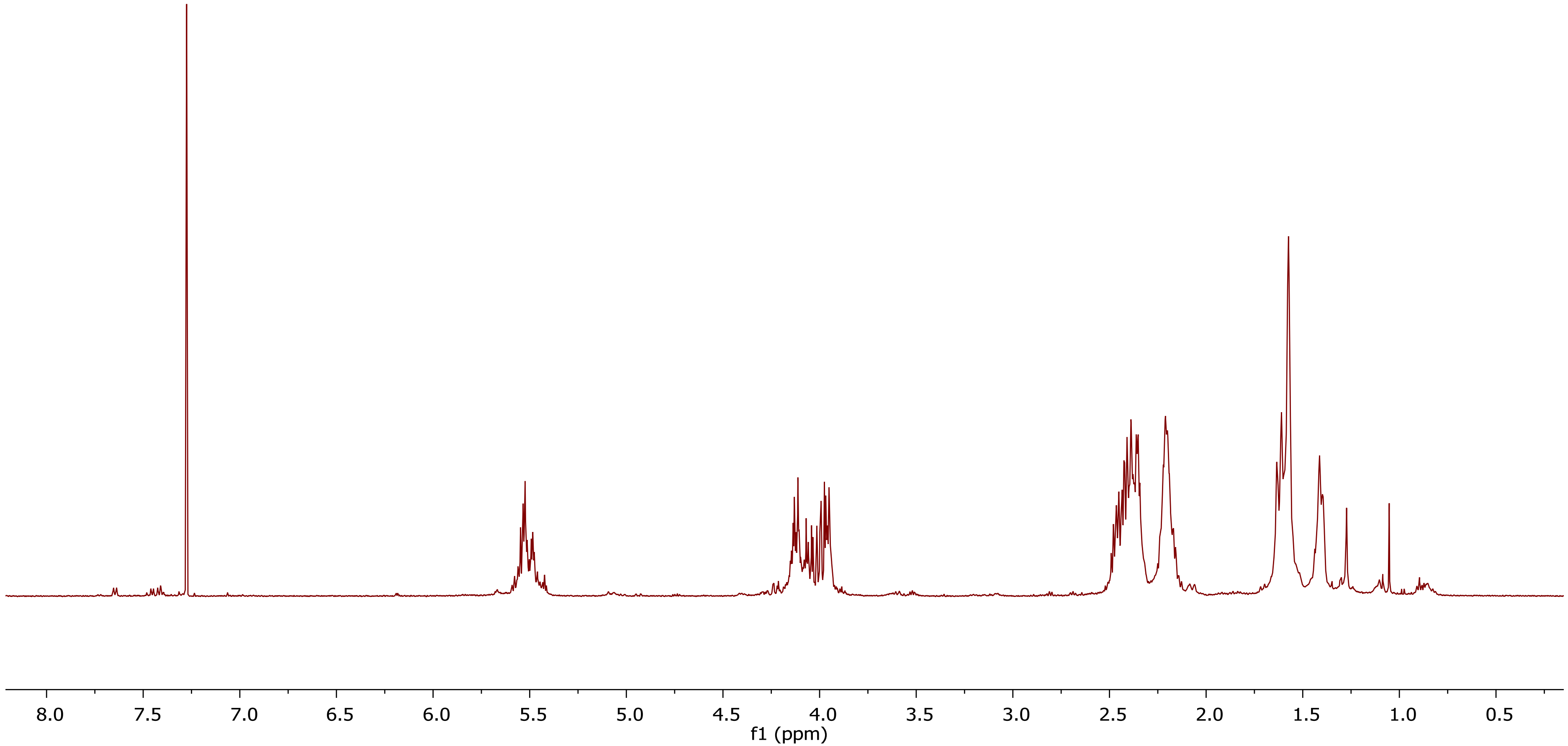

(1S,10S,10aR,11R,14S,14aS)-1,2,5,6,9,10,10a,11,12,13,14,14a-dodecahydro-4H-1,10-epoxy-11,14-methanobenzo[d][1]oxacyclododecin-4-one

41

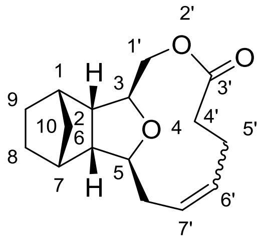

41

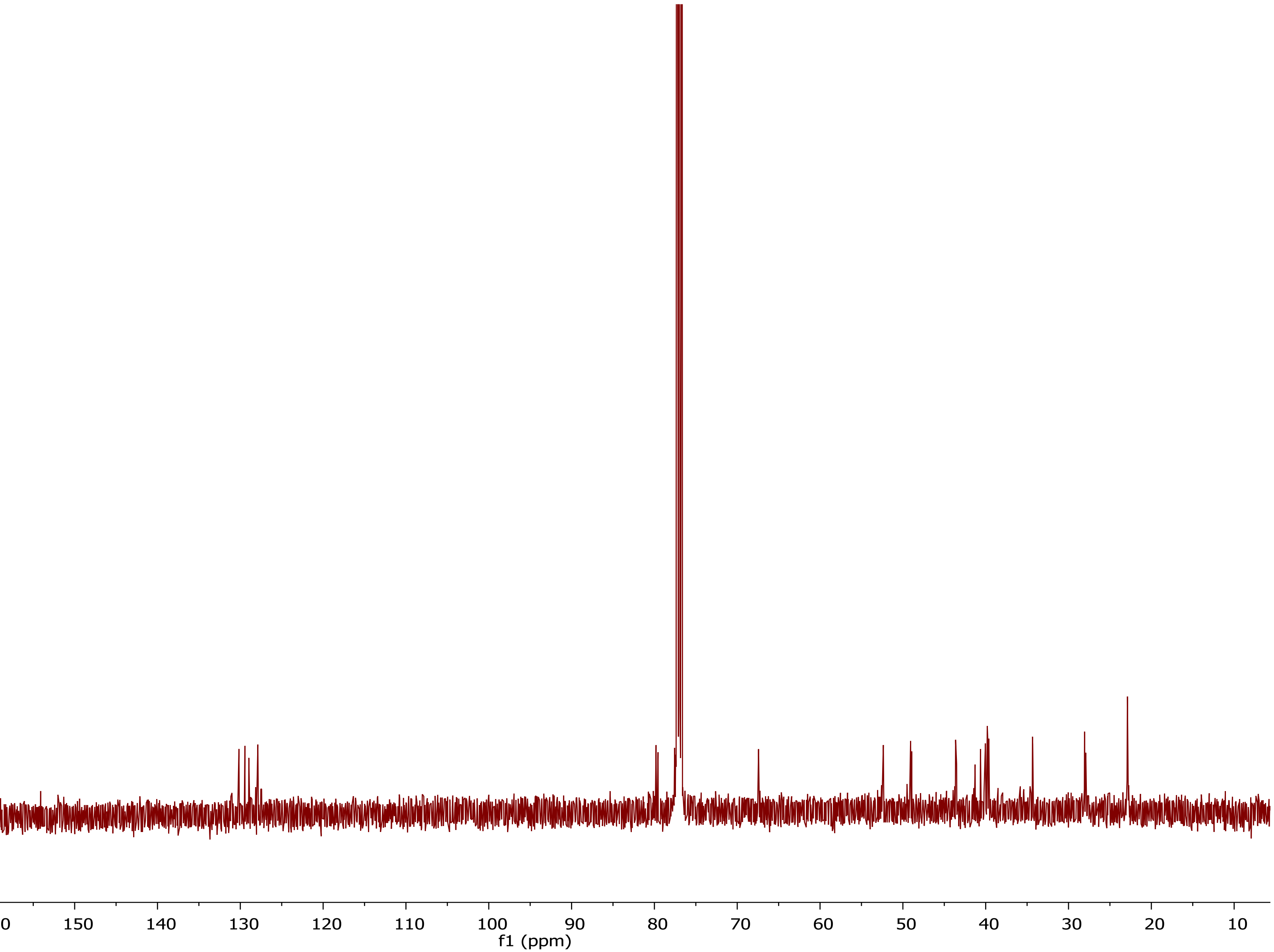

(1S,2S,3S,5S,6R,7R)-5-Allyl-4-oxatricyclo[5.2.1.0^{2,6}]dec-3-ylmethyl hex-5-enoate **83**

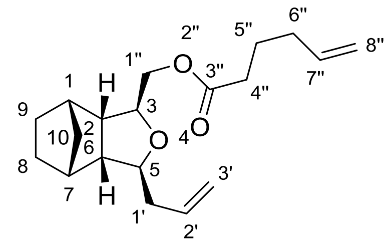

83

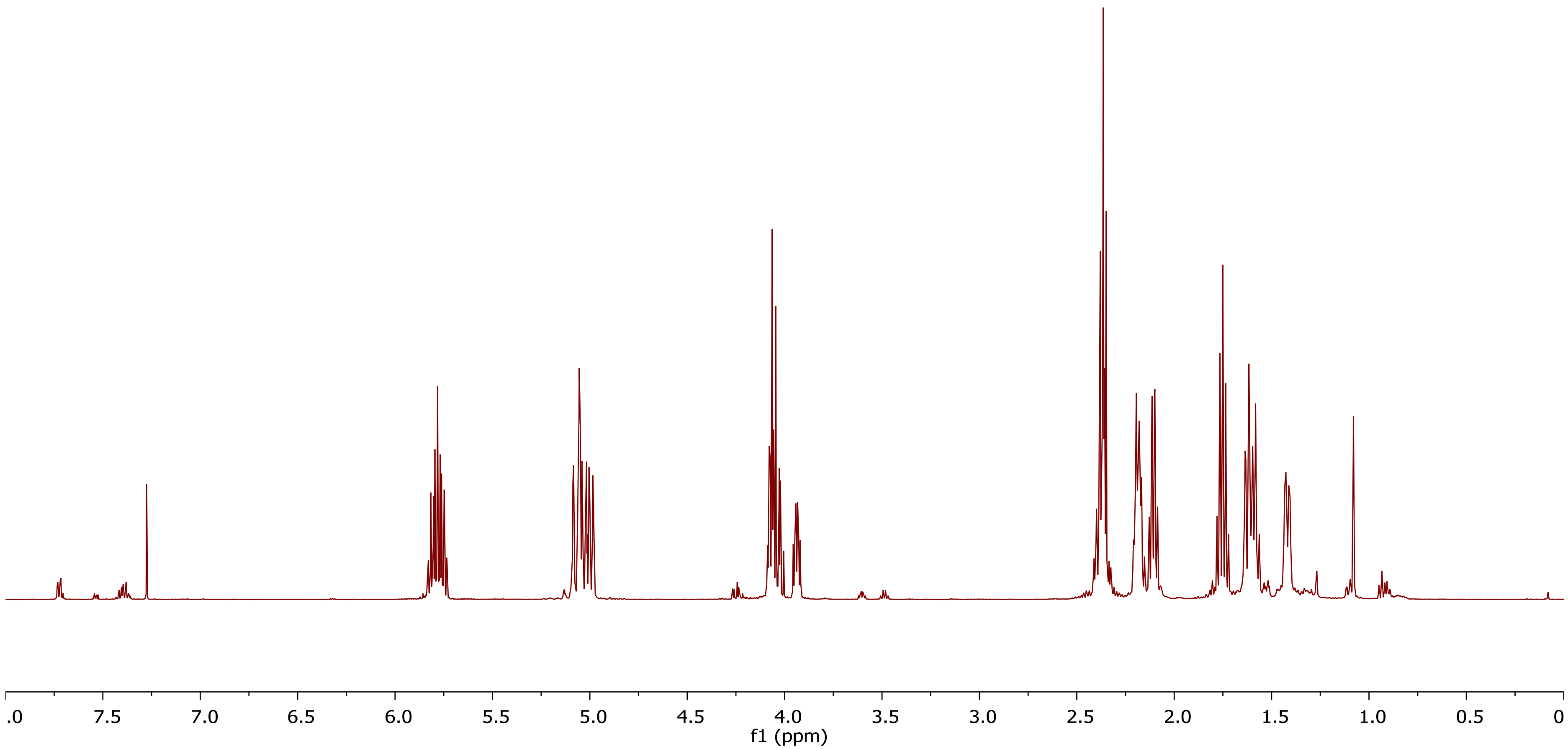

(1S,2S,3S,5S,6R,7R)-5-Allyl-4-oxatricyclo[5.2.1.0^{2,6}]dec-3-ylmethyl hex-5-enoate **83**

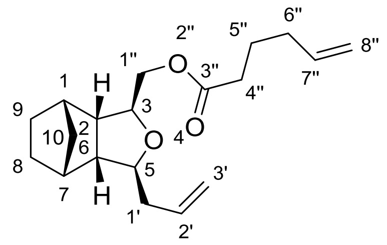

83

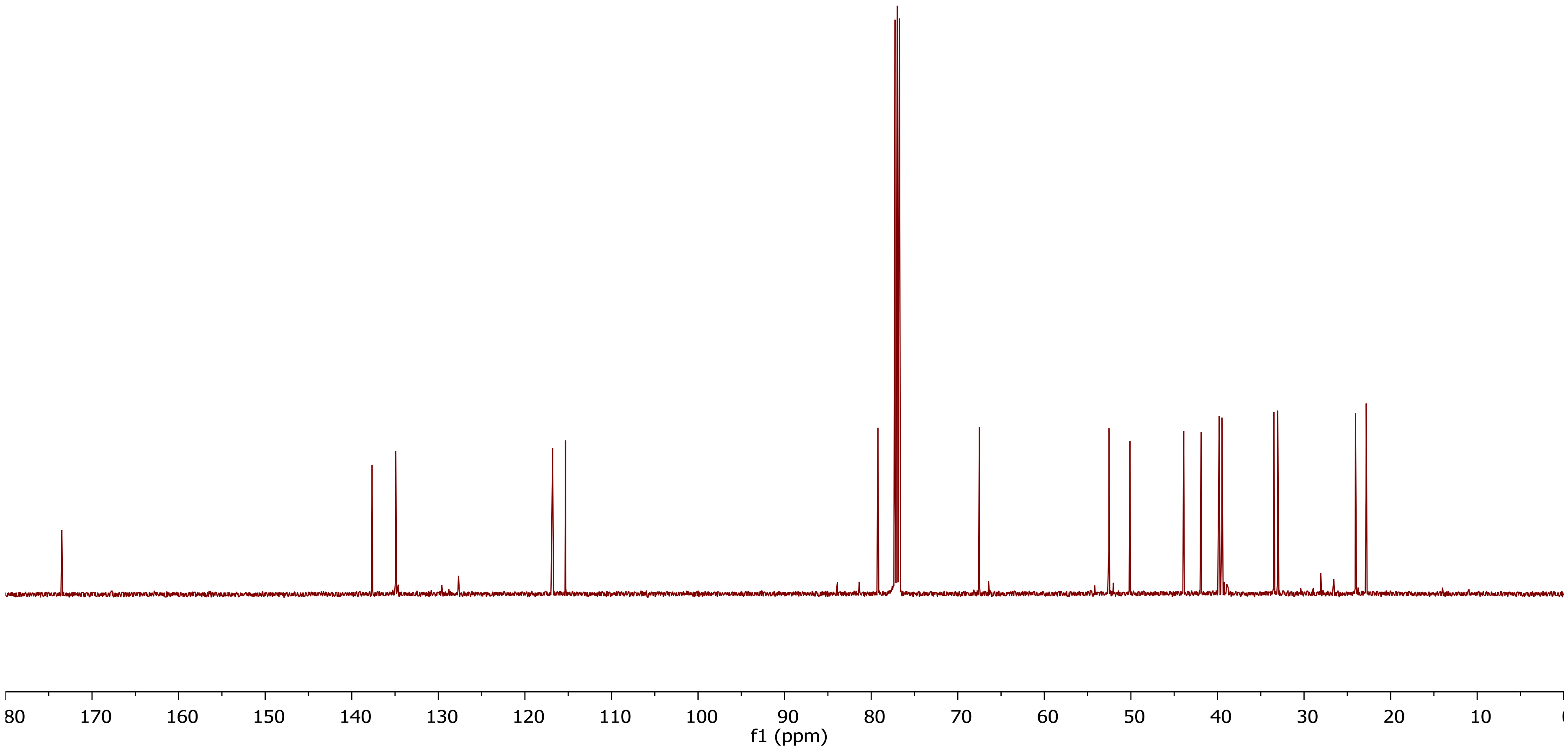

(E)-(1S,11S,12R,17S)-3,18-Dioxatetracyclo[9.6.1.1^{13,16}.0 ^{12,17}]nonadec-8-en-4-one **42**

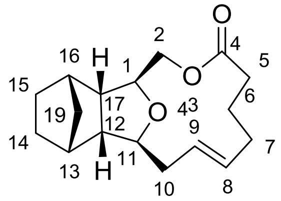

42

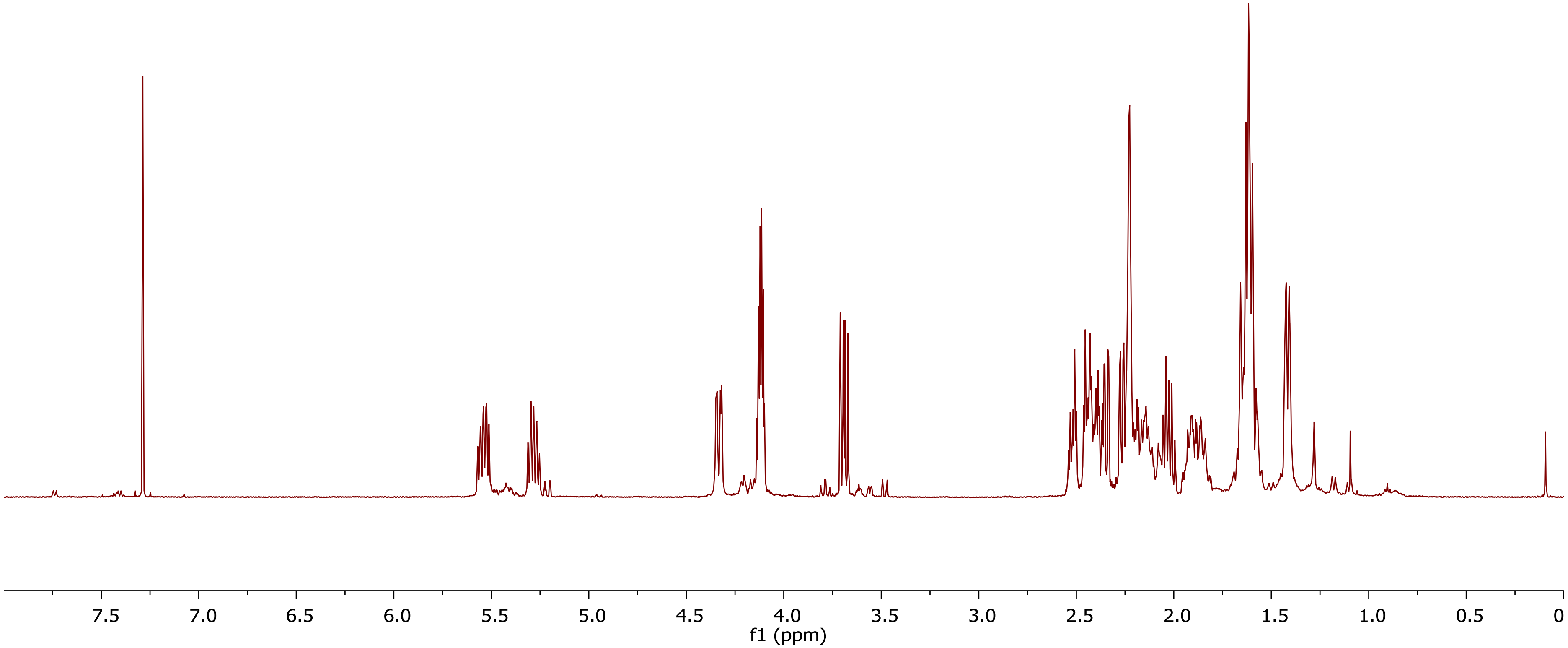

(E)-(1S,11S,12R,17S)-3,18-Dioxatetracyclo[9.6.1.1^{13,16}.0 ^{12,17}]nonadec-8-en-4-one **42**

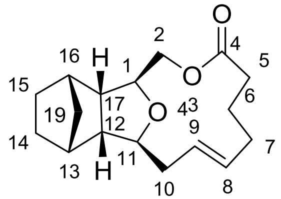

42

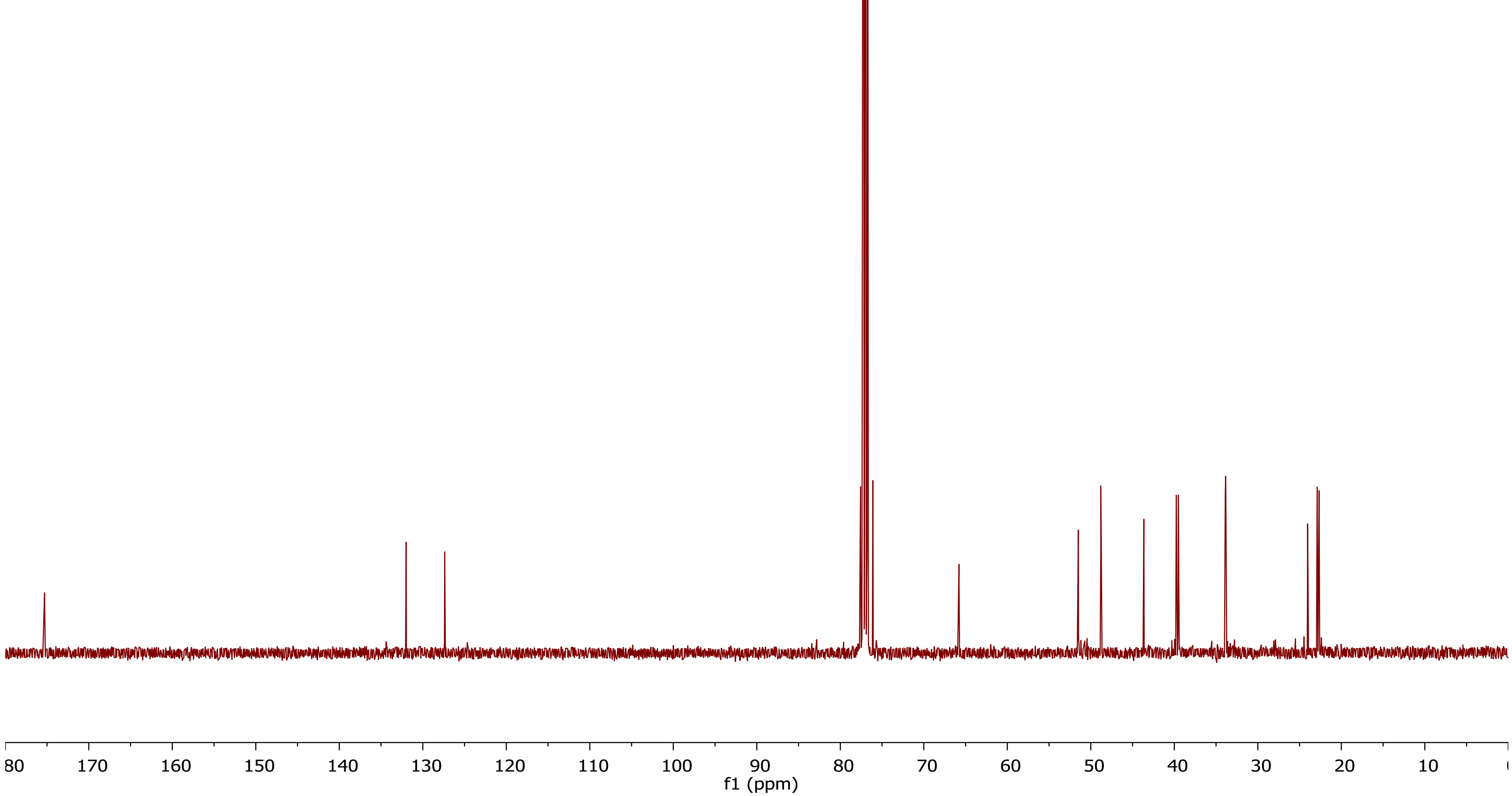

2-[(1R,3S,3aS,7aR)-3-{[(tert-butyldiphenylsilyl)oxy]methyl}-1,3,3a,4,7,7a-hexahydro-2-benzofuran-1-yl]-2-methylpropanal **44**

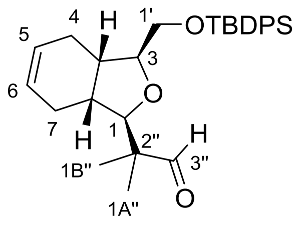

44

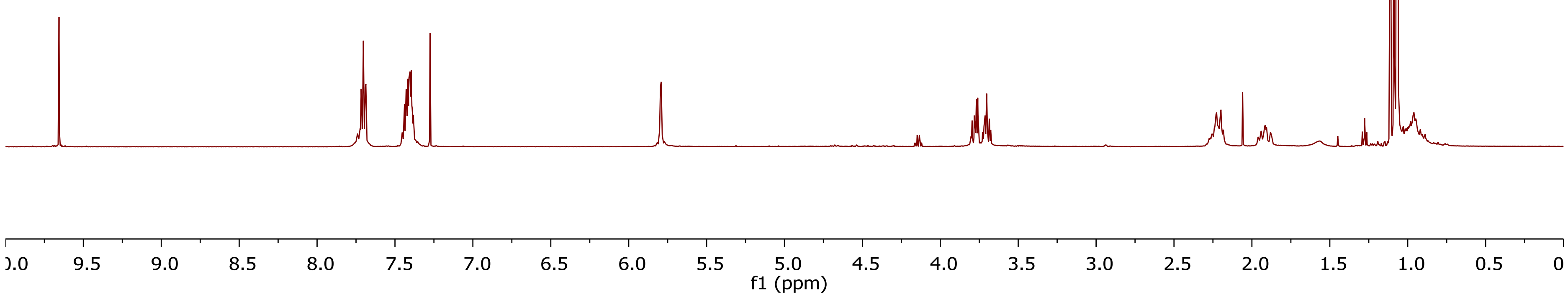

2-[(1R,3S,3aS,7aR)-3-{[(tert-butyldiphenylsilyl)oxy]methyl}-1,3,3a,4,7,7a-hexahydro-2-benzofuran-1-yl]-2-methylpropanal **44**

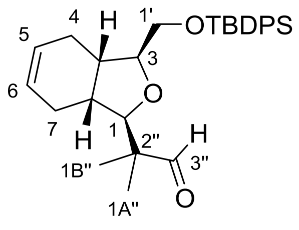

44

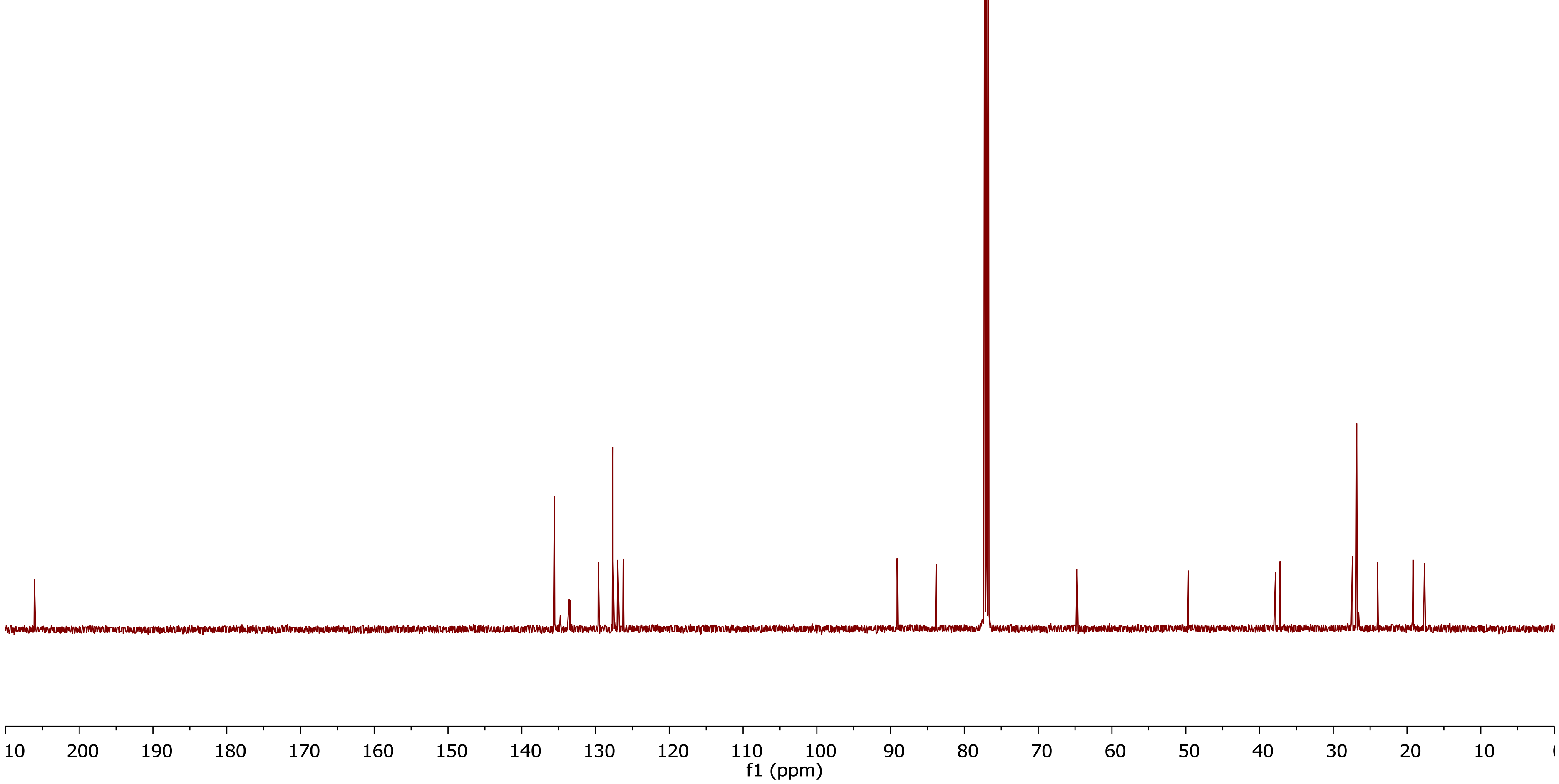

2-((1S,3S,3aS,7aR)-3-(((tert-butyldiphenylsilyl)oxy)methyl)-1,3,3a,4,7,7a-hexahydroisobenzofuran-1-yl)-1-phenylethan-1-one **45**

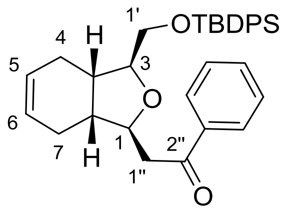

45

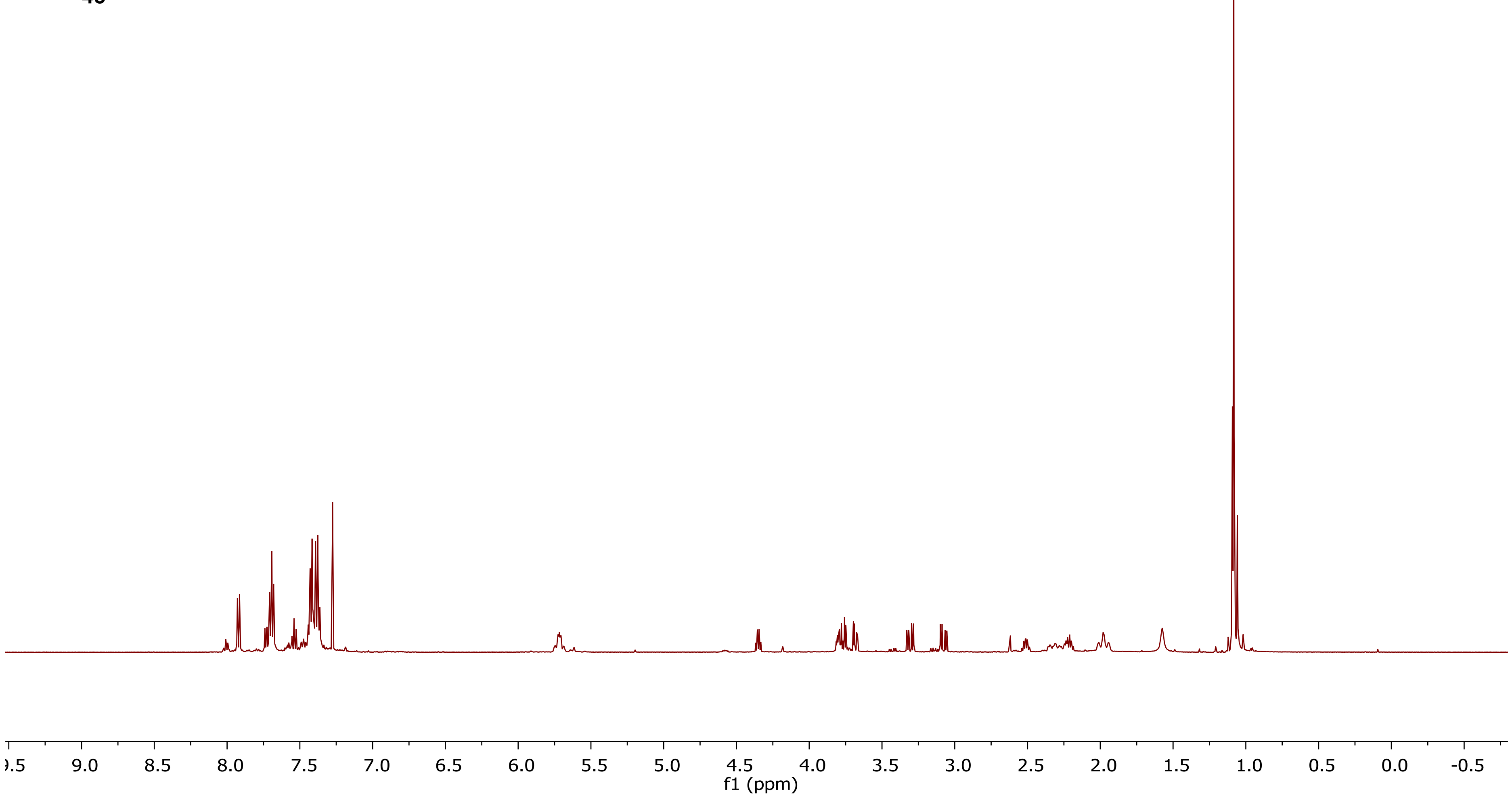

2-((1S,3S,3aS,7aR)-3-(((tert-butyldiphenylsilyl)oxy)methyl)-1,3,3a,4,7,7a-hexahydroisobenzofuran-1-yl)-1-phenylethan-1-one **45**

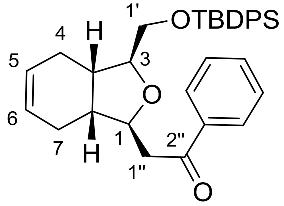

45

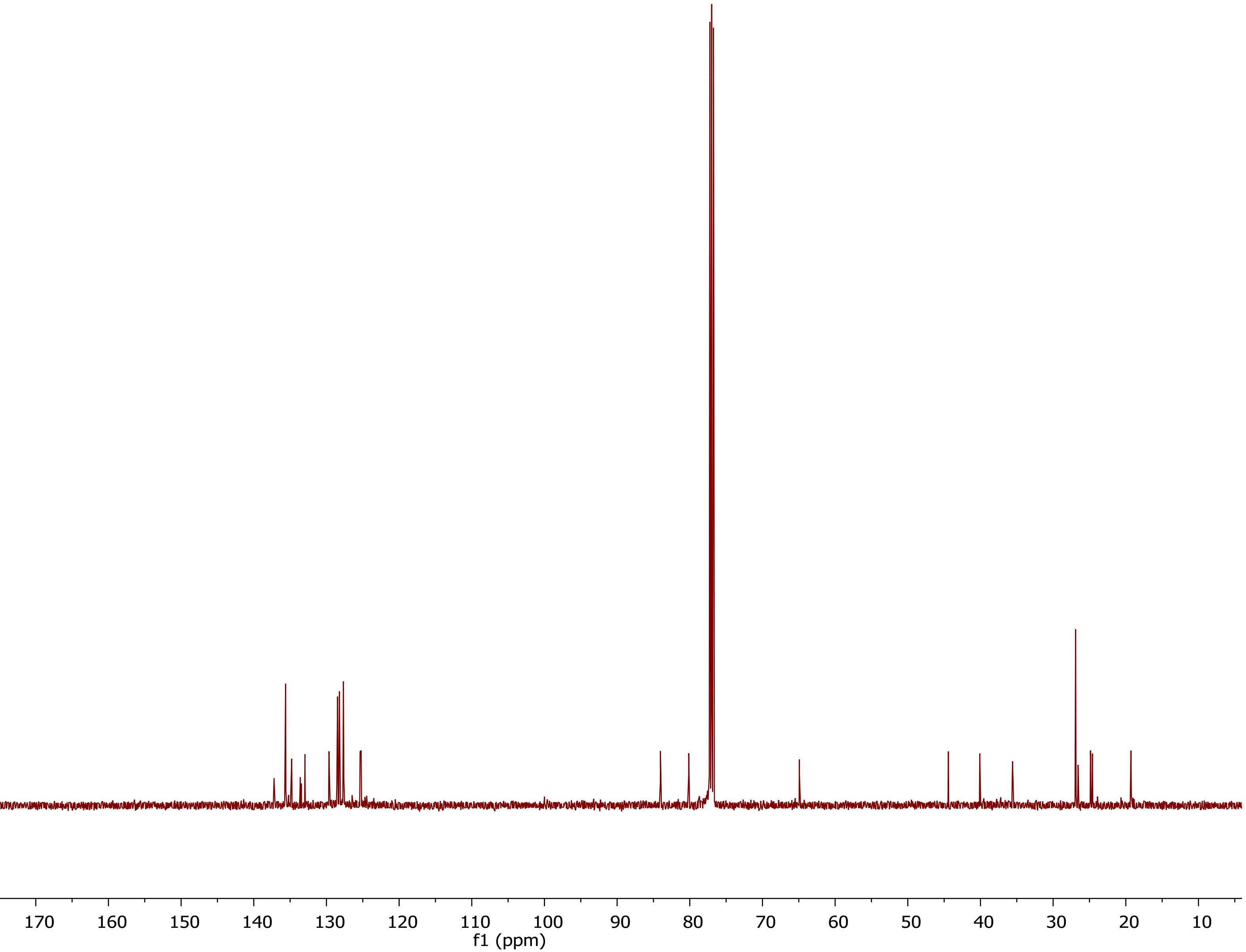

3-[(1R,3S,3aS,7aR)-3-{[(*tert*-Butyldiphenylsilyl)oxy]methyl}-octahydro-2-benzofuran-1-yl]-3-methylbutan-2-one **46a**

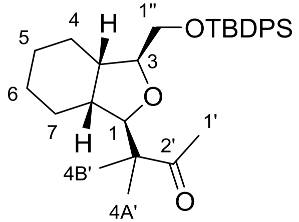

46a

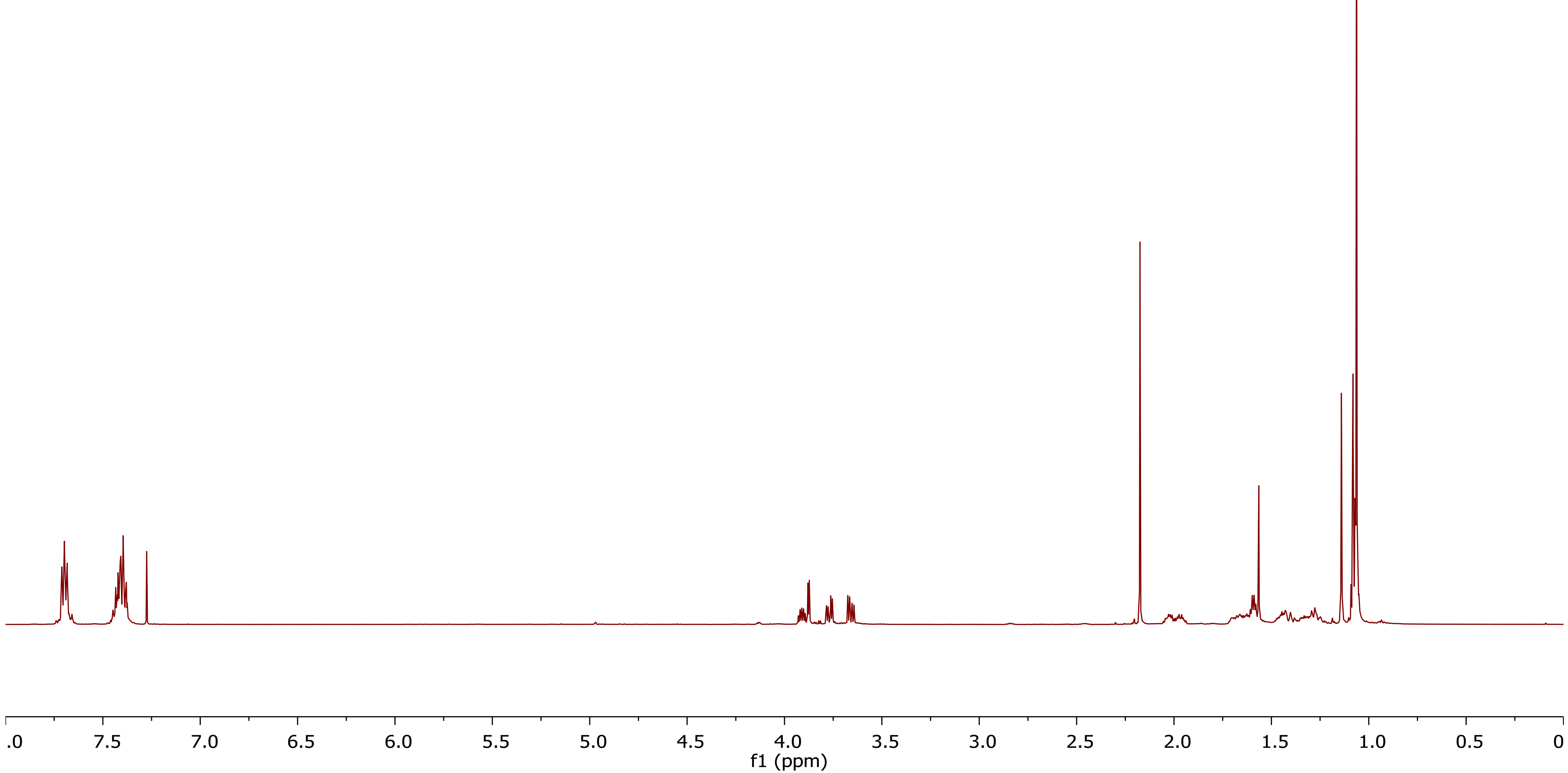

3-[(1R,3S,3aS,7aR)-3-{[(*tert*-Butyldiphenylsilyl)oxy]methyl}-octahydro-2-benzofuran-1-yl]-3-methylbutan-2-one **46a**

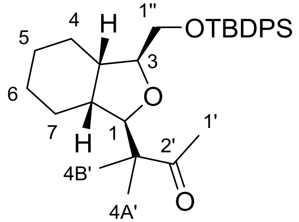

46a

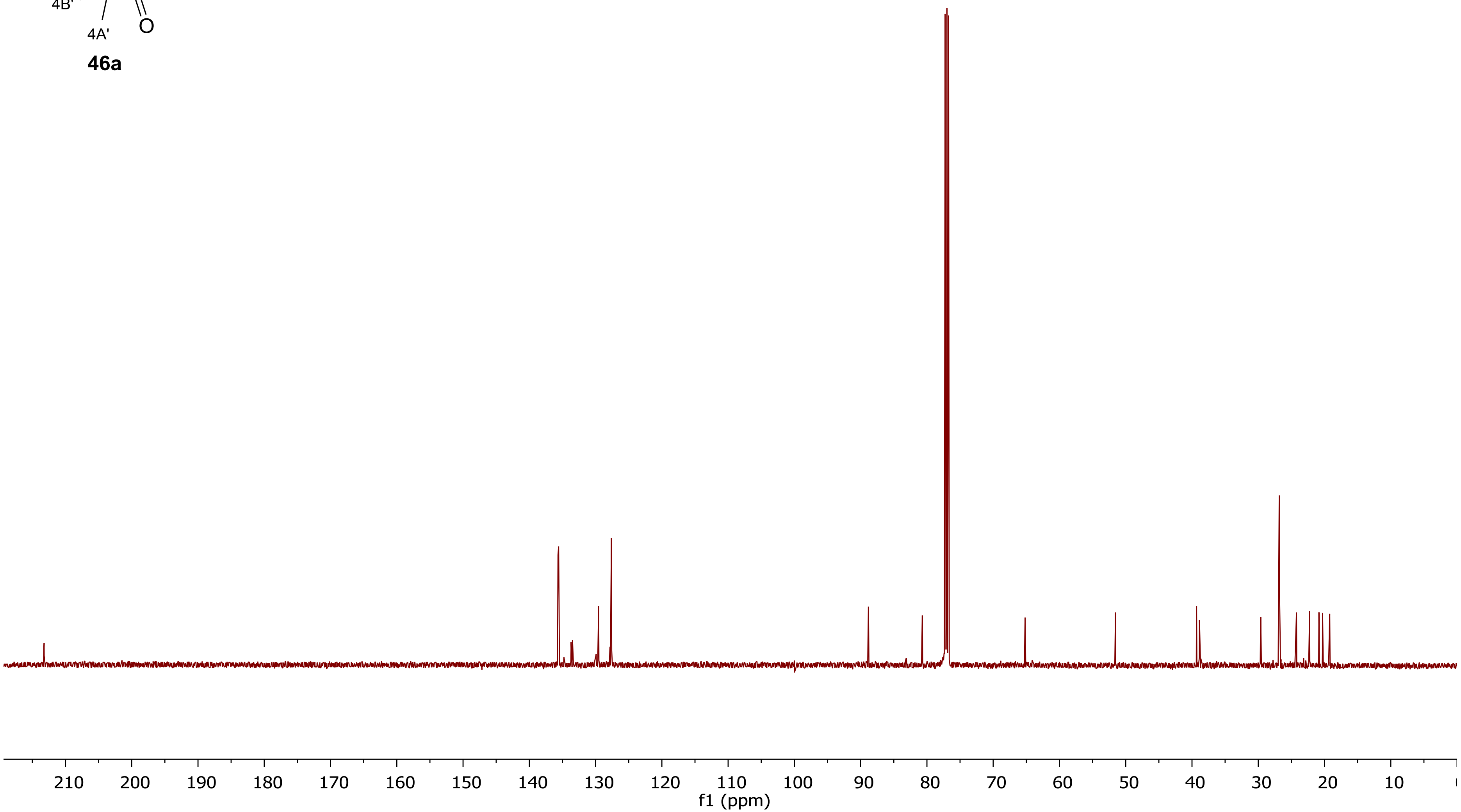

3-[(1R,3S,3aS,7aR)-3-(Hydroxymethyl)-octahydro-2-benzofuran-1-yl]-3-methylbutan-2-one **46**

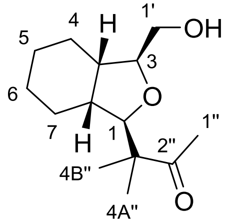

46

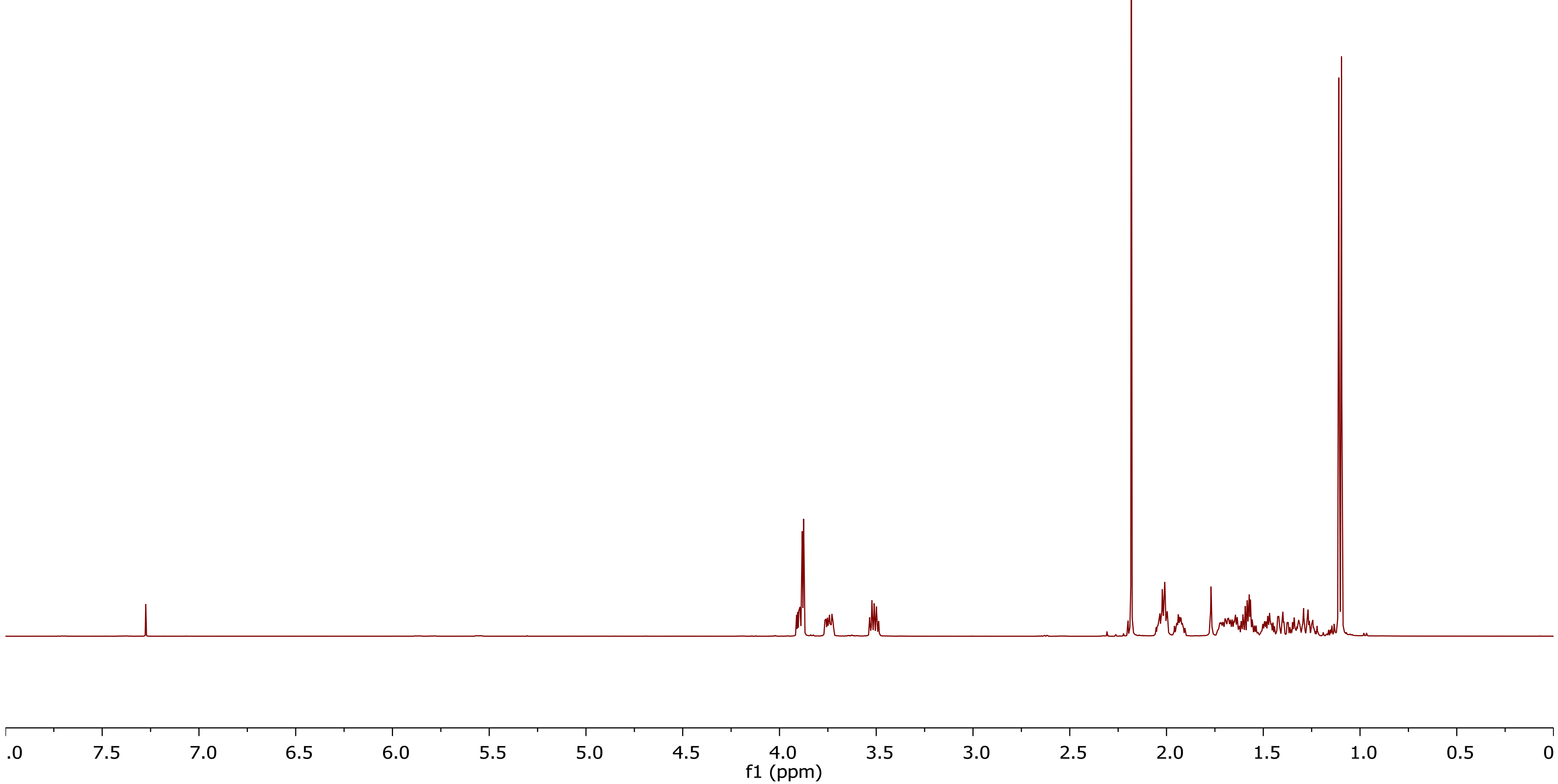

3-[(1R,3S,3aS,7aR)-3-(Hydroxymethyl)-octahydro-2-benzofuran-1-yl]-3-methylbutan-2-one **46**

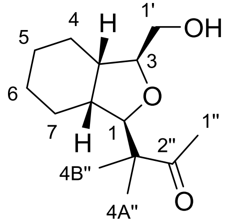

46

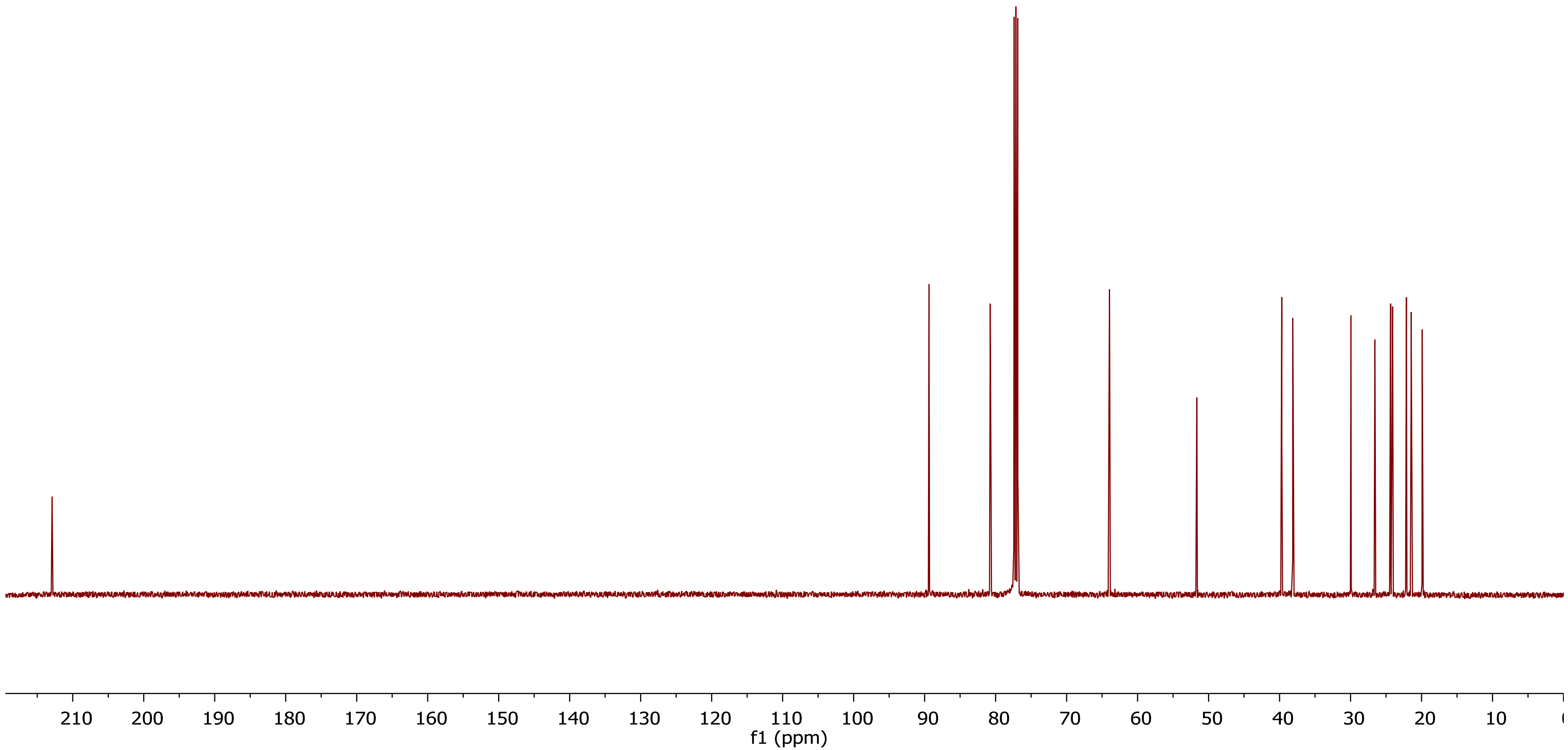

1-((1S,3S,3aS,7aR)-3-(((tert-butyldiphenylsilyl)oxy)methyl)octahydroisobenzofuran-1-yl)-3,3-dimethylbutan-2-one **47**

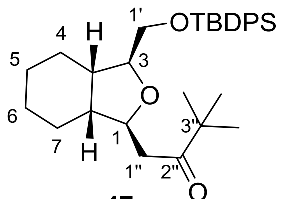

47a

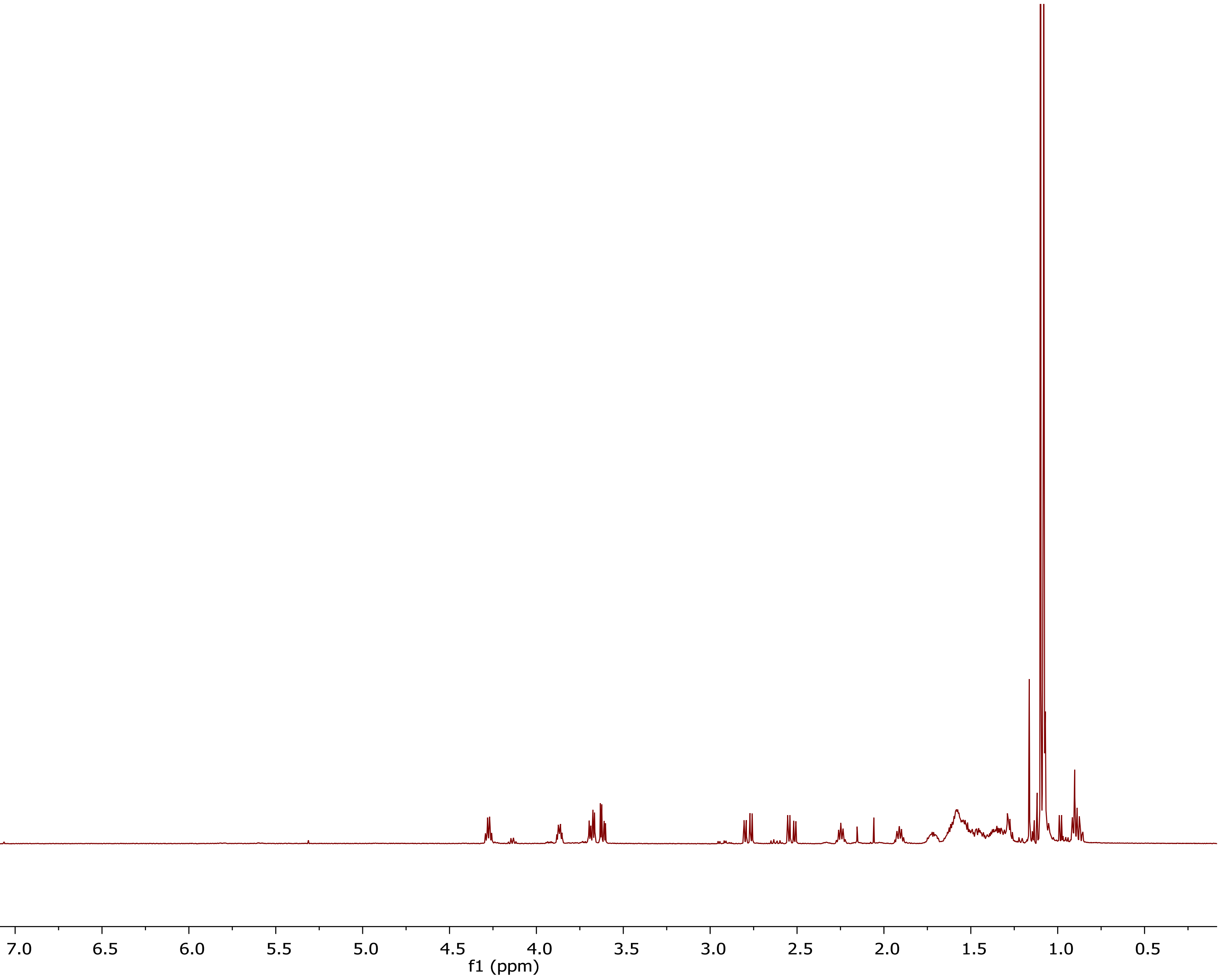

1-((1S,3S,3aS,7aR)-3-(((tert-butyldiphenylsilyl)oxy)methyl)octahydroisobenzofuran-1-yl)-3,3-dimethylbutan-2-one **47**

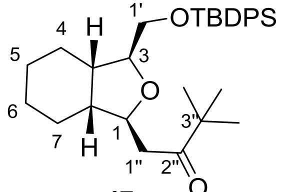

47a

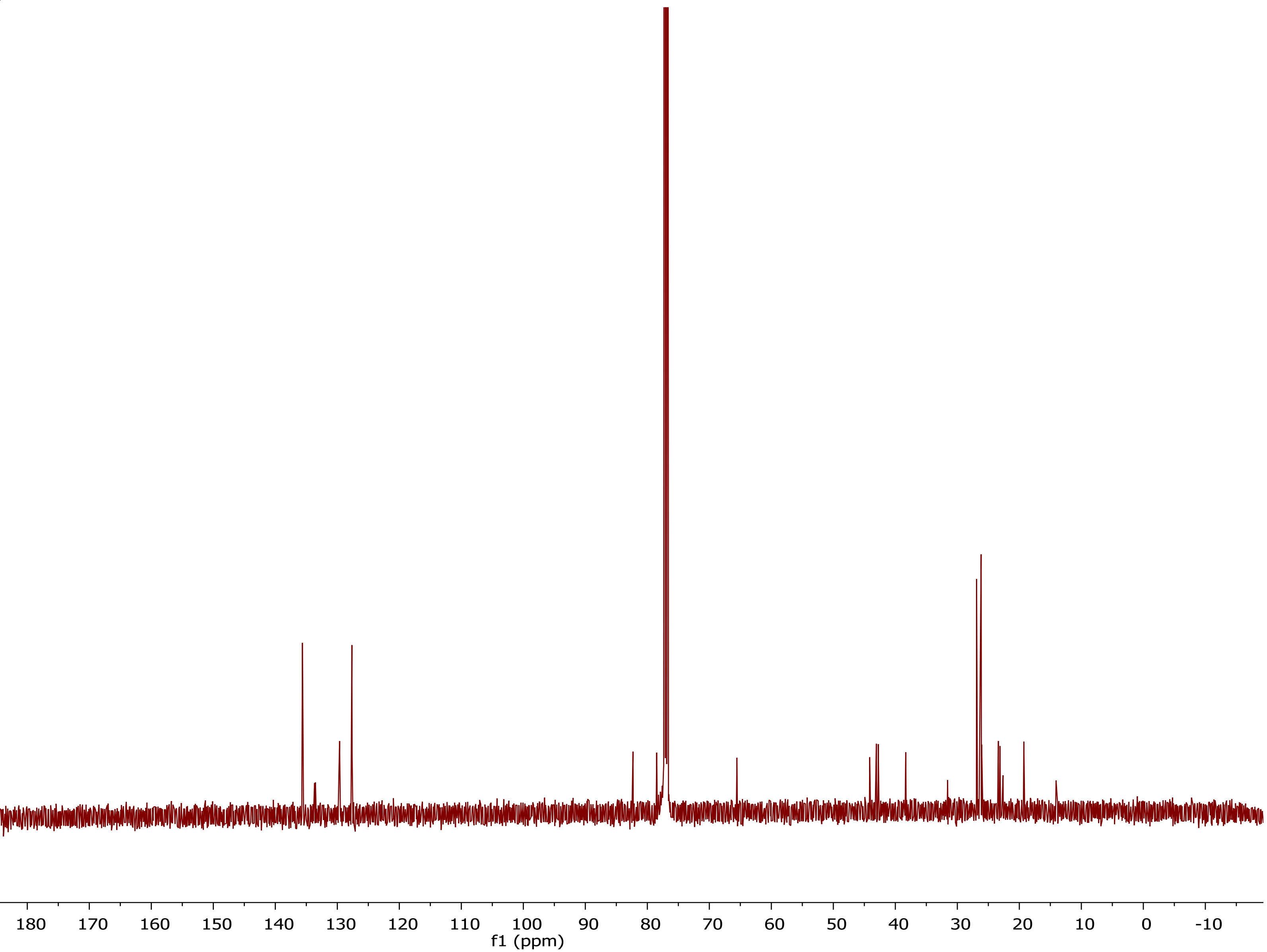

1-((1S,3S,3aS,7aR)-3-(hydroxymethyl)octahydroisobenzofuran-1-yl)-3,3-dimethylbutan-2-one **47**

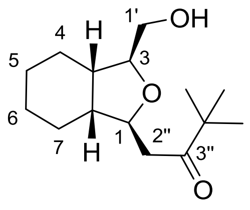

47

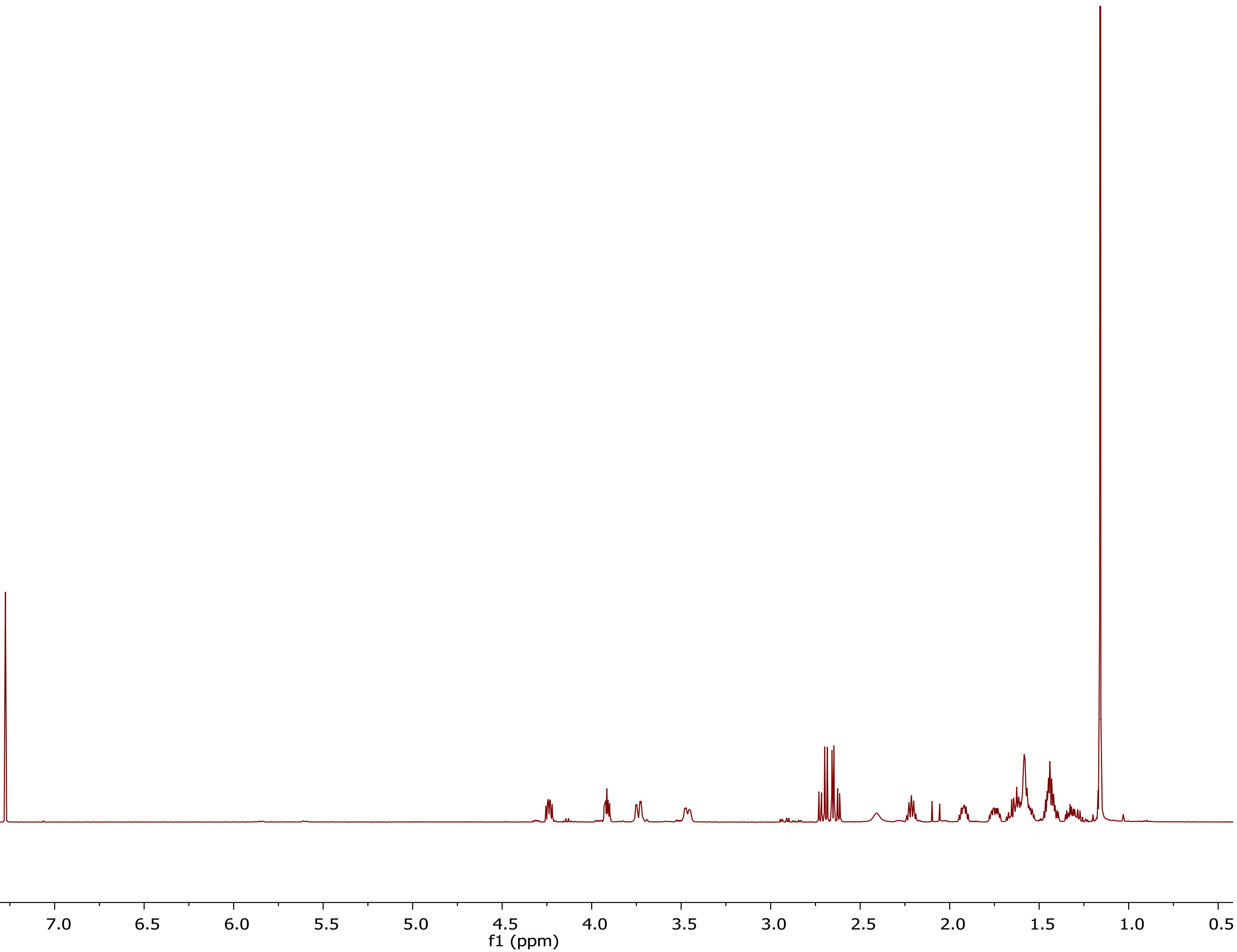

1-((1S,3S,3aS,7aR)-3-(hydroxymethyl)octahydroisobenzofuran-1-yl)-3,3-dimethylbutan-2-one **47**

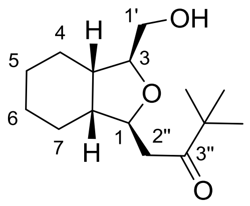

47

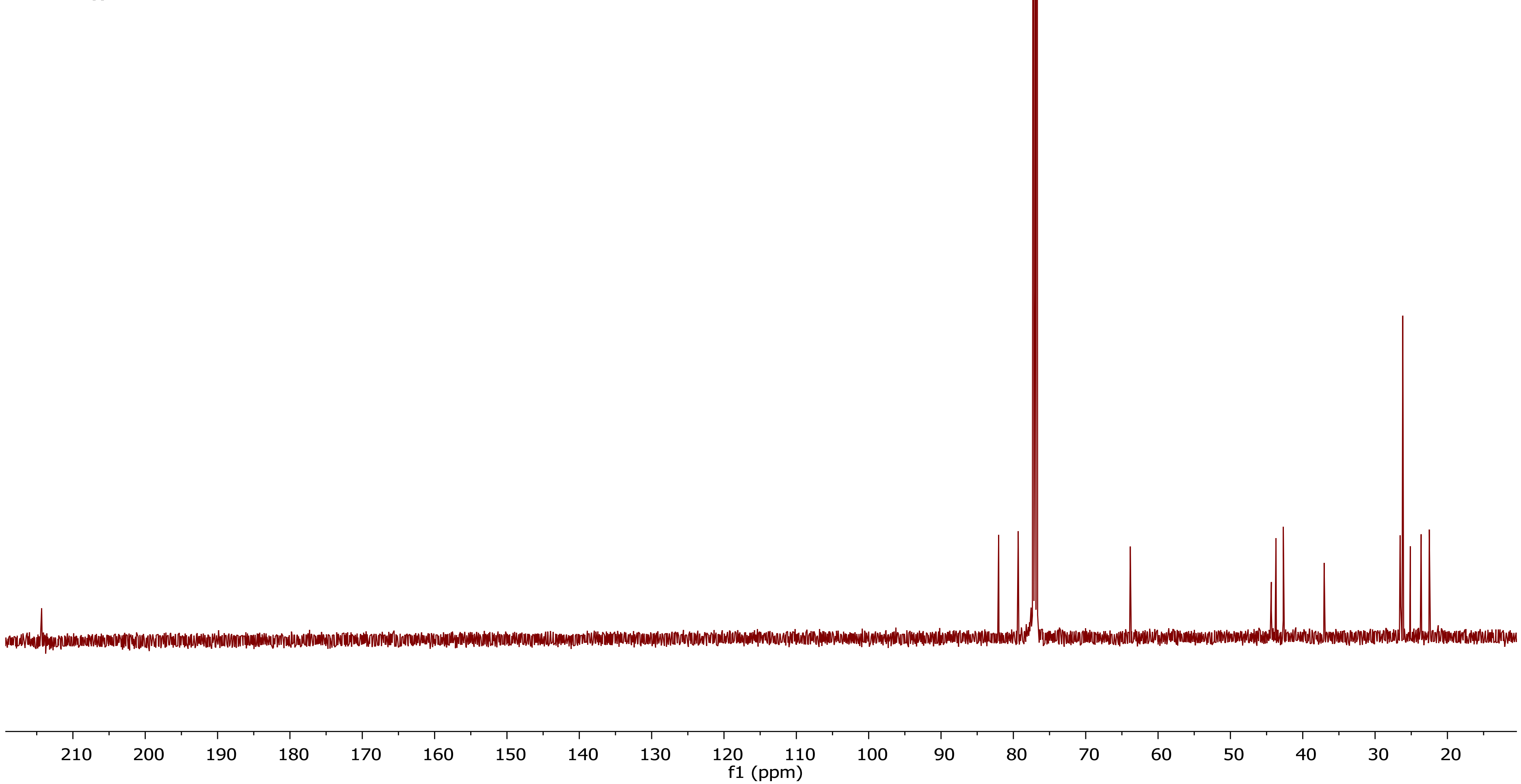

1-((1S,3S,3aS,7aR)-3-((tert-butyldiphenylsilyloxy)methyl)-1,3,3a,4,7,7a-hexahydroisobenzofuran-1-yl)-3,3-dimethylbutan-2-one **48a**

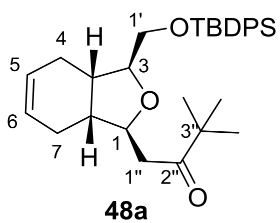

48a

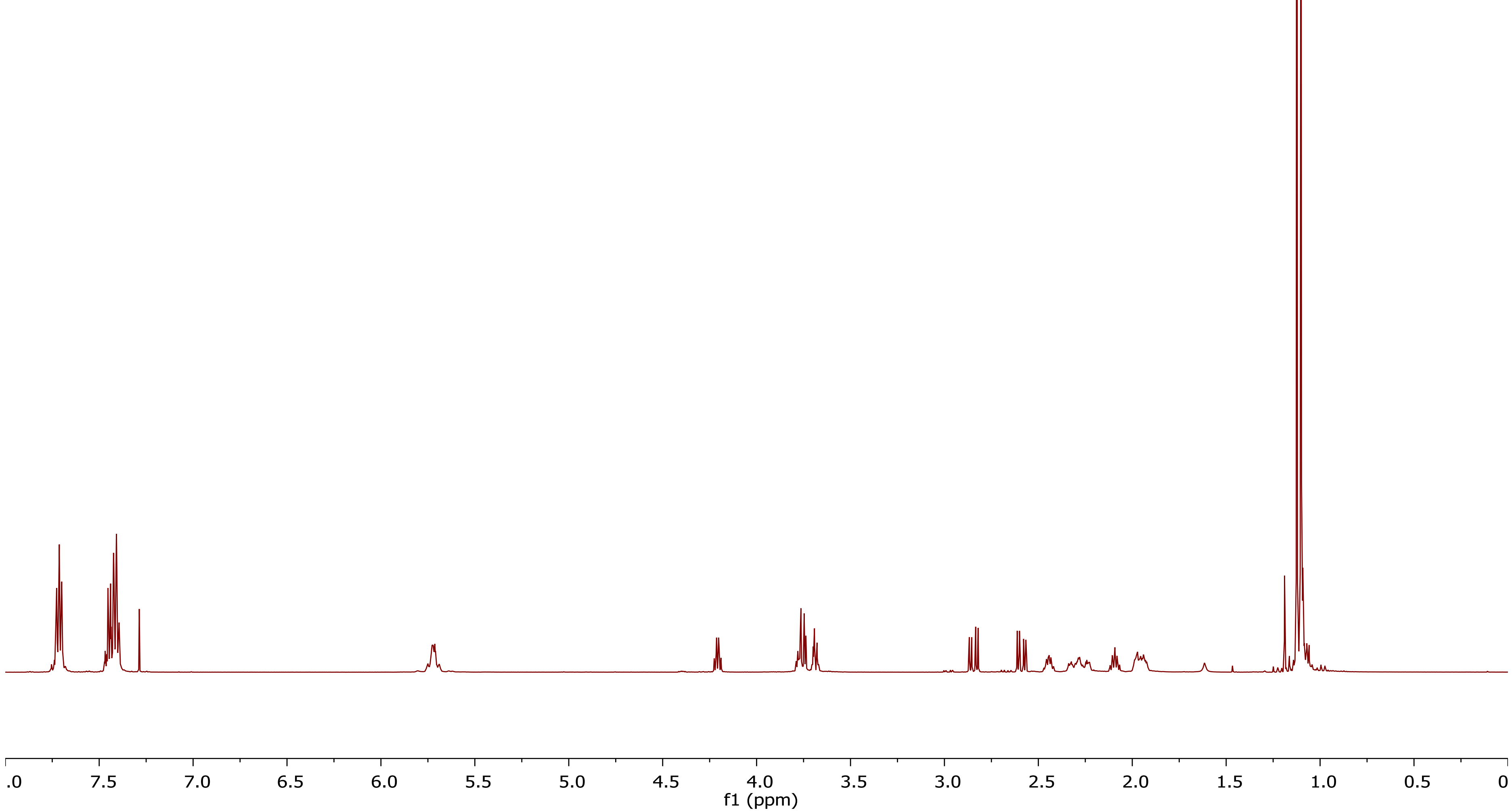

1-((1S,3S,3aS,7aR)-3-((tert-butyldiphenylsilyloxy)methyl)-1,3,3a,4,7,7a-hexahydroisobenzofuran-1-yl)-3,3-dimethylbutan-2-one **48a**

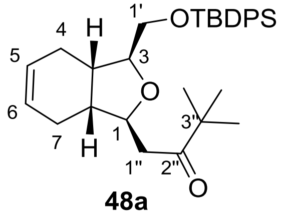

48a

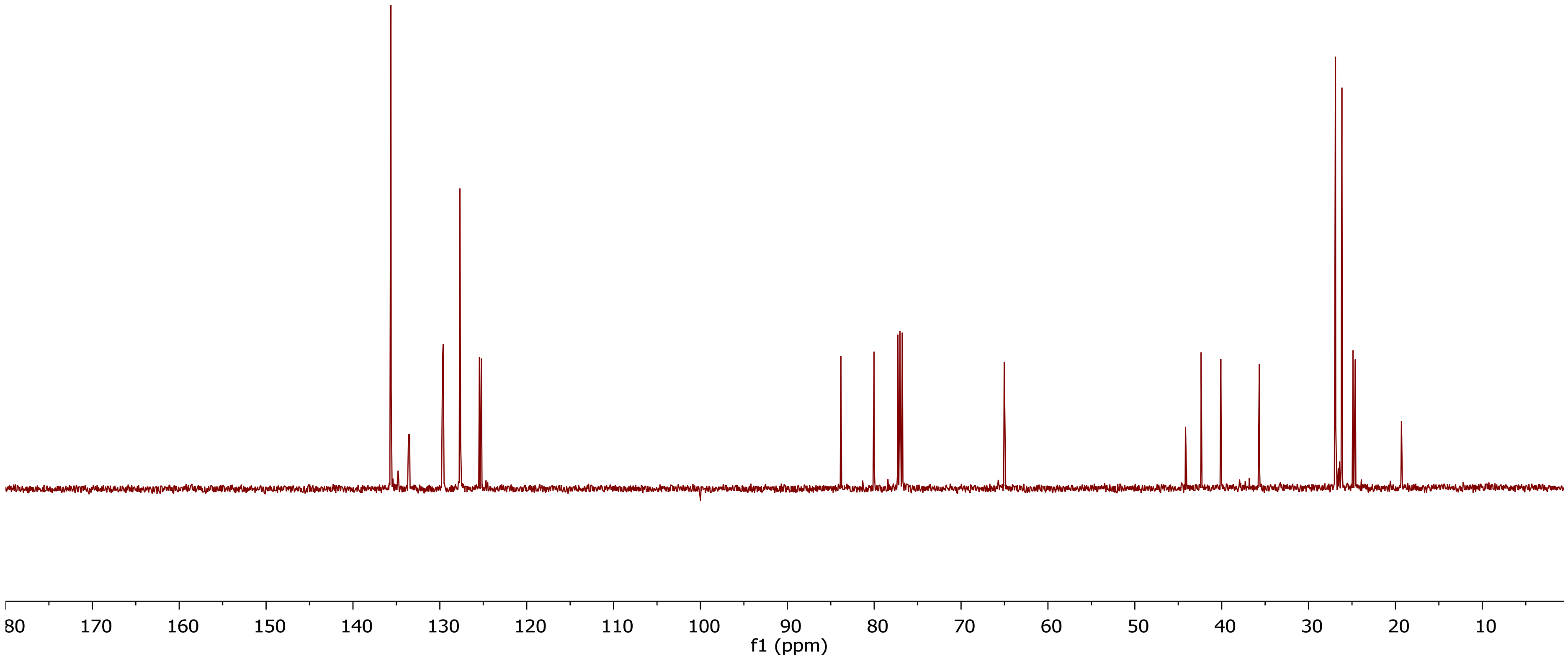

1-[(1S,3S,3aS,7aR)-3-(hydroxymethyl)-1,3,3a,4,7,7a-hexahydro-2-benzofuran-1-yl]-3,3-imethylbutan-2-one **48**

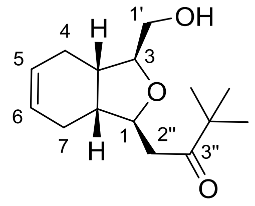

48

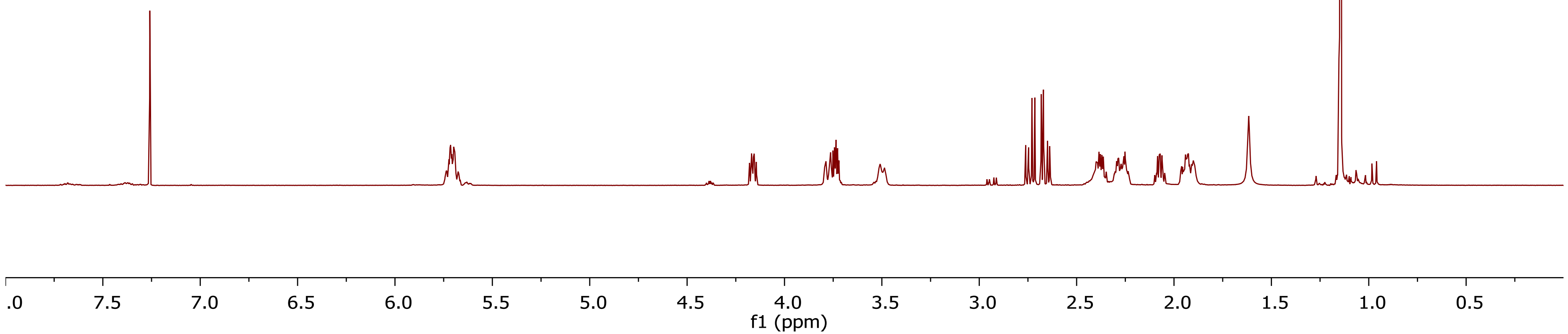

1-[(1S,3S,3aS,7aR)-3-(hydroxymethyl)-1,3,3a,4,7,7a-hexahydro-2-benzofuran-1-yl]-3,3-imethylbutan-2-one **48**

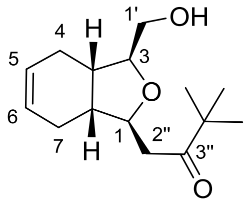

48

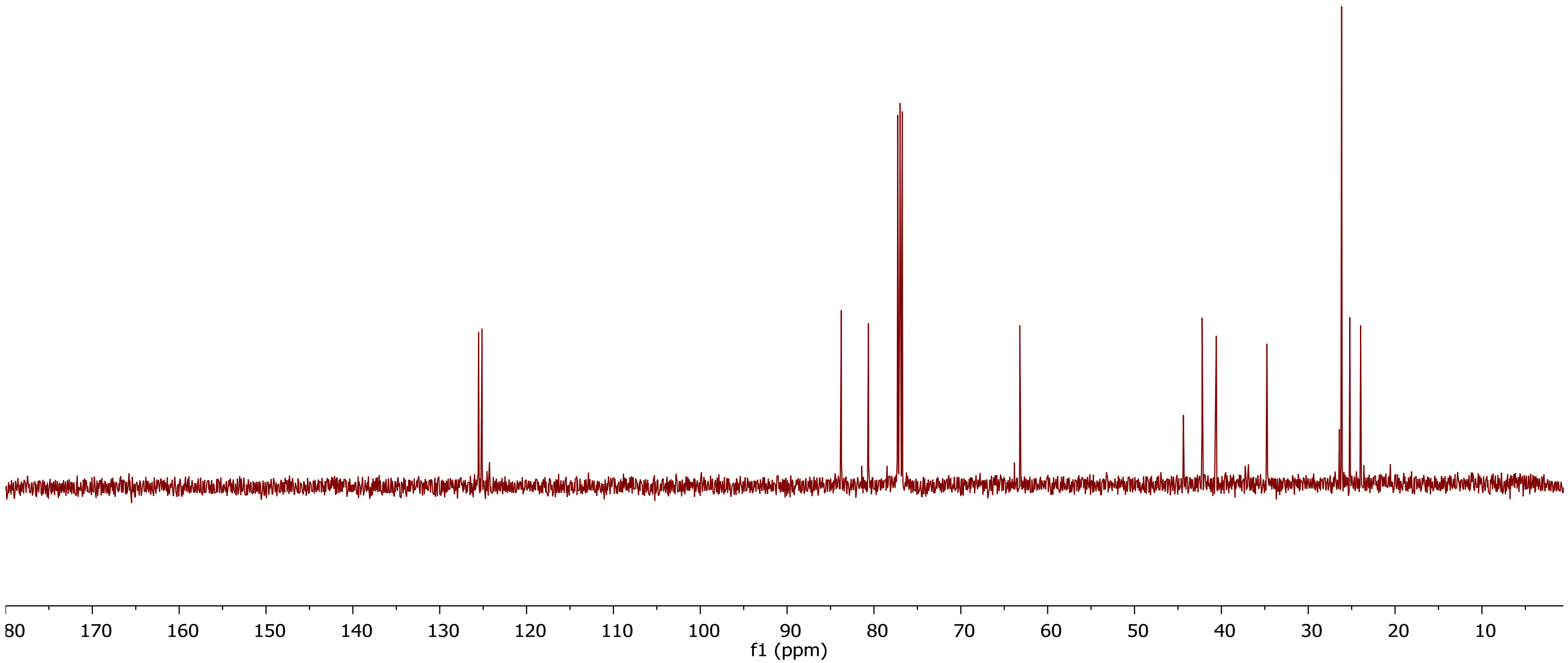

2-[(1R,3S,3aS,7aR)-3-{[(*tert*-butyldiphenylsilyl)oxy]methyl}-1,3,3a,4,7,7a-hexahydro-2-benzofuran-1-yl]-2-methylpropan-1-ol **49a**

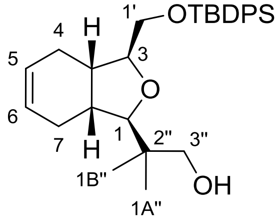

49a

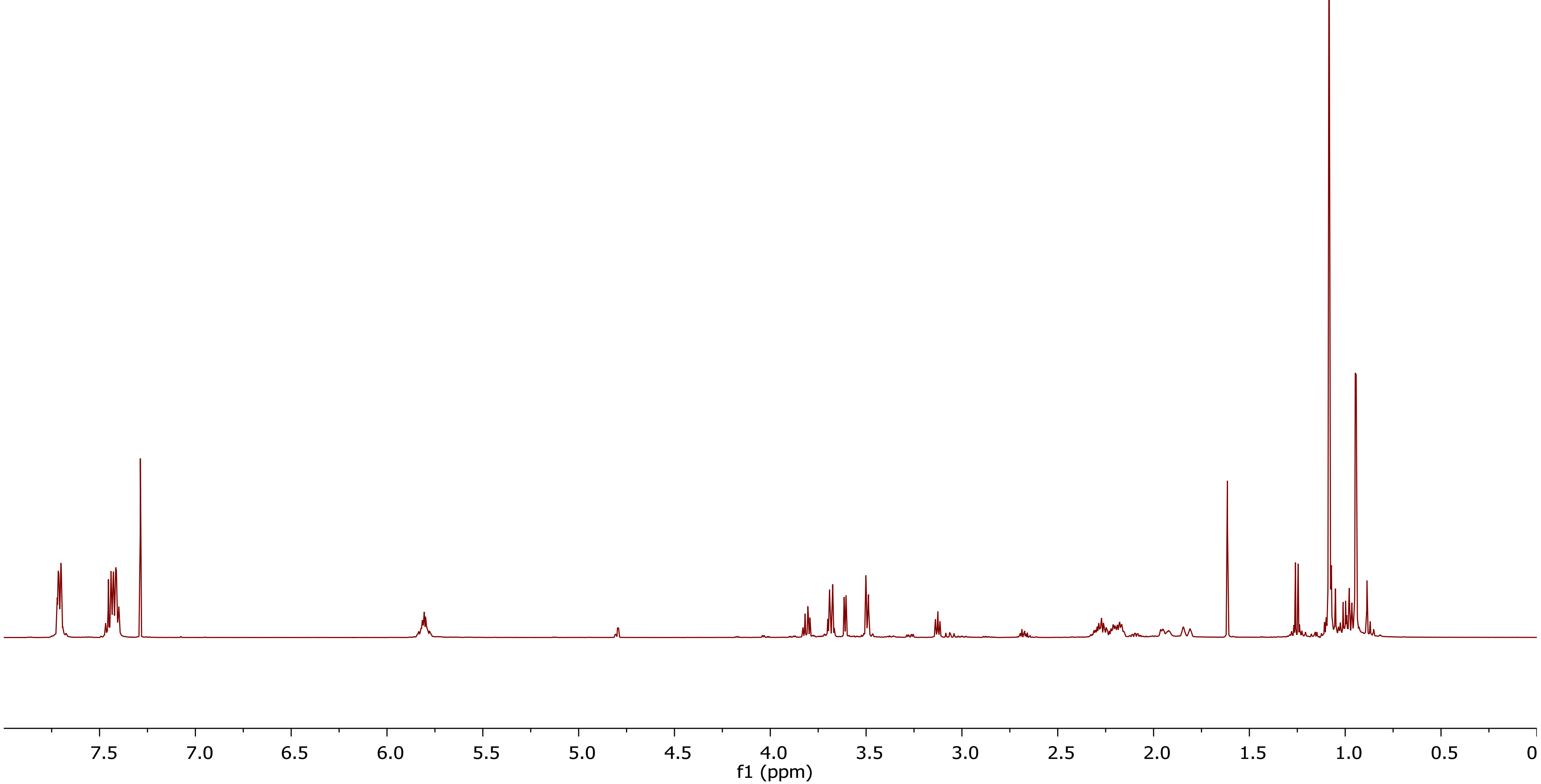

2-[(1R,3S,3aS,7aR)-3-{[(*tert*-butyldiphenylsilyl)oxy]methyl}-1,3,3a,4,7,7a-hexahydro-2-benzofuran-1-yl]-2-methylpropan-1-ol **49a**

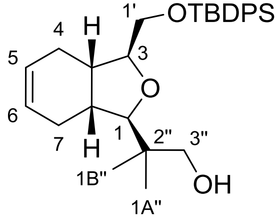

49a

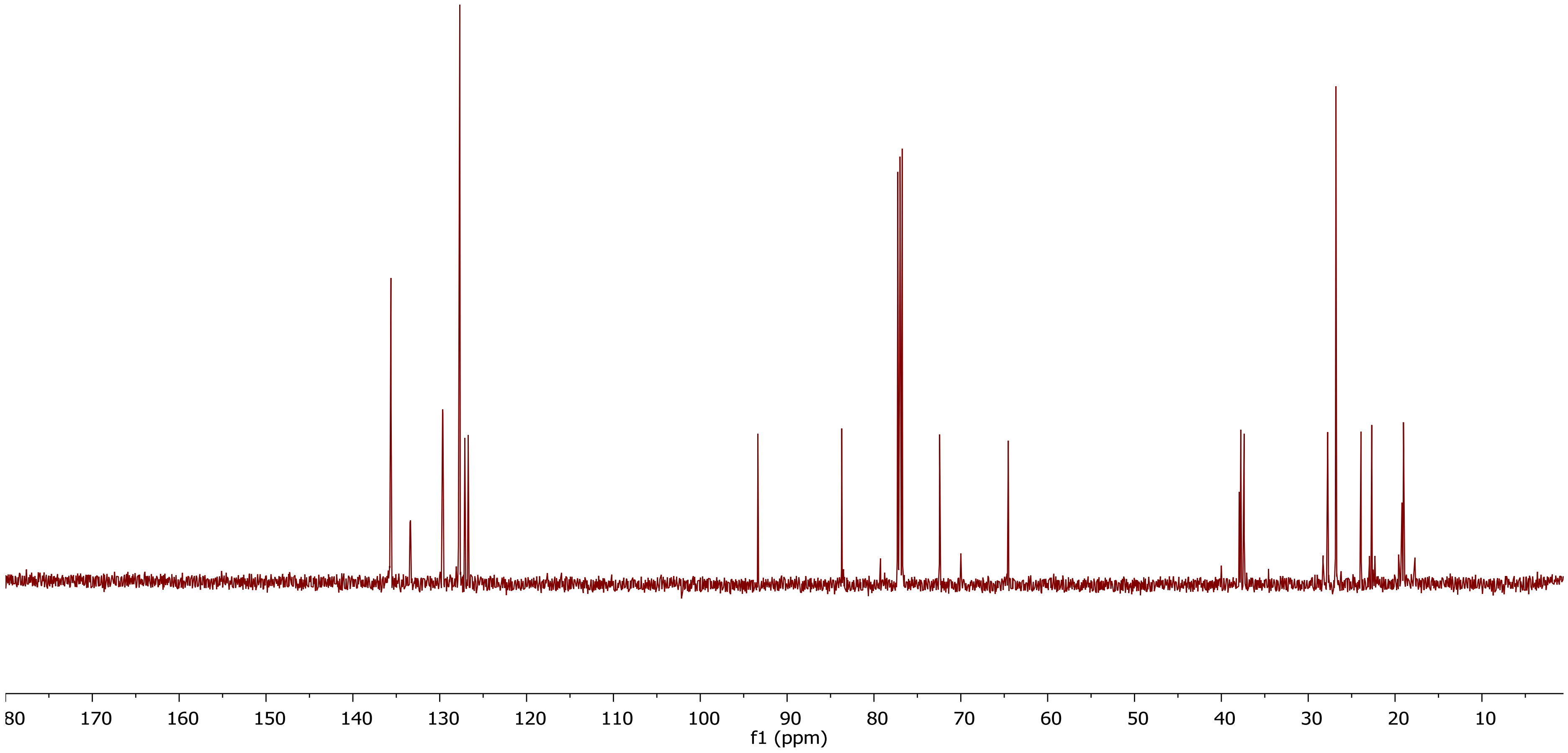

2-[(1R,3S,3aS,7aR)-3-(hydroxymethyl)-1,3,3a,4,7,7a-hexahydro-2-benzofuran-1-yl]-2-methylpropan-1-ol **49**

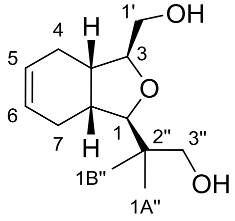

49

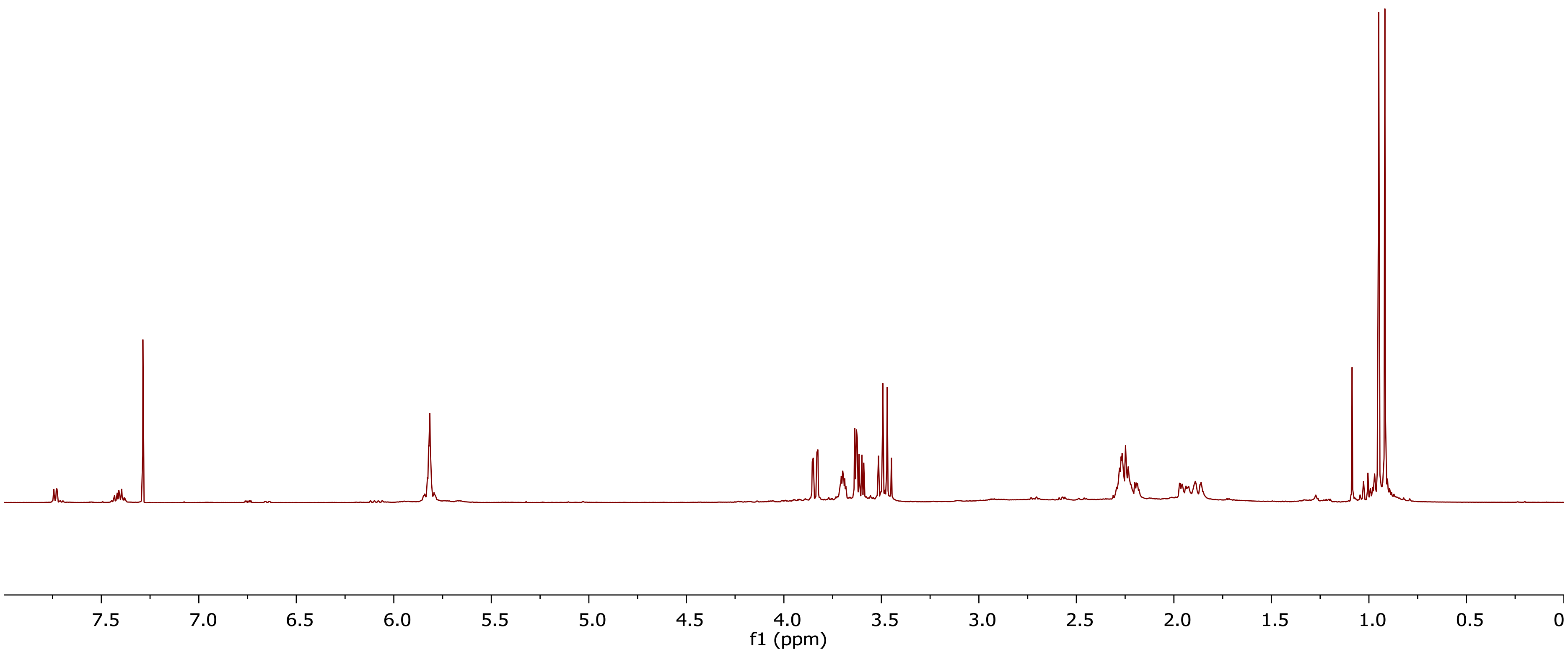

2-[(1R,3S,3aS,7aR)-3-(hydroxymethyl)-1,3,3a,4,7,7a-hexahydro-2-benzofuran-1-yl]-2-methylpropan-1-ol **49**

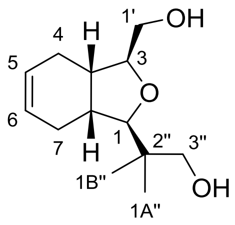

49

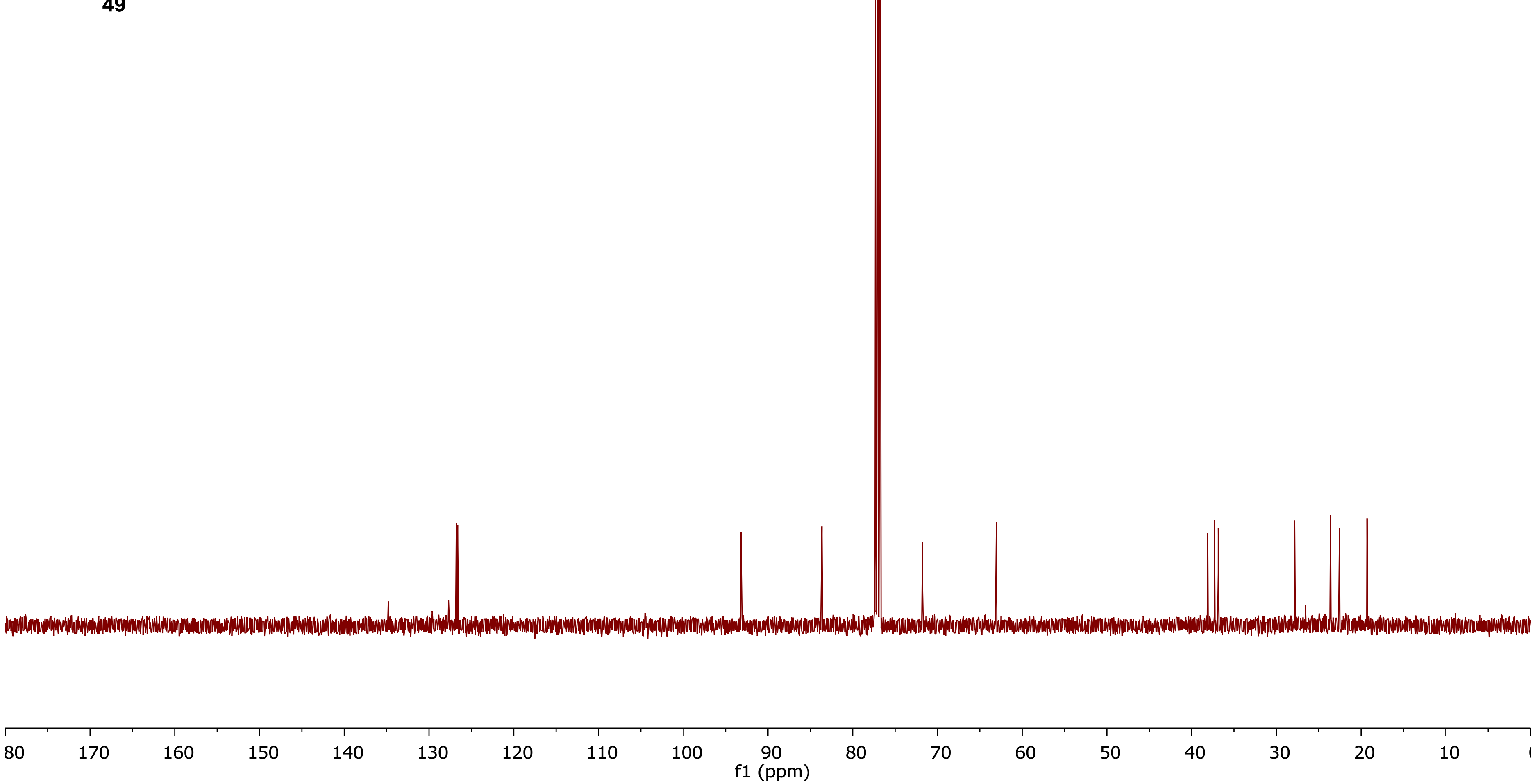

{2-[(1R,3S,3aS,7aR)-3-{[(*tert*-Butyldiphenylsilyl)oxy]methyl}-1,3,3a,4,7,7a-hexahydro-2-benzofuran-1-yl]-2-methylpropyl}(benzyl)amine **50**

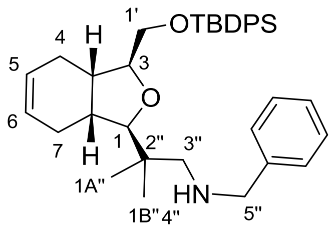

50

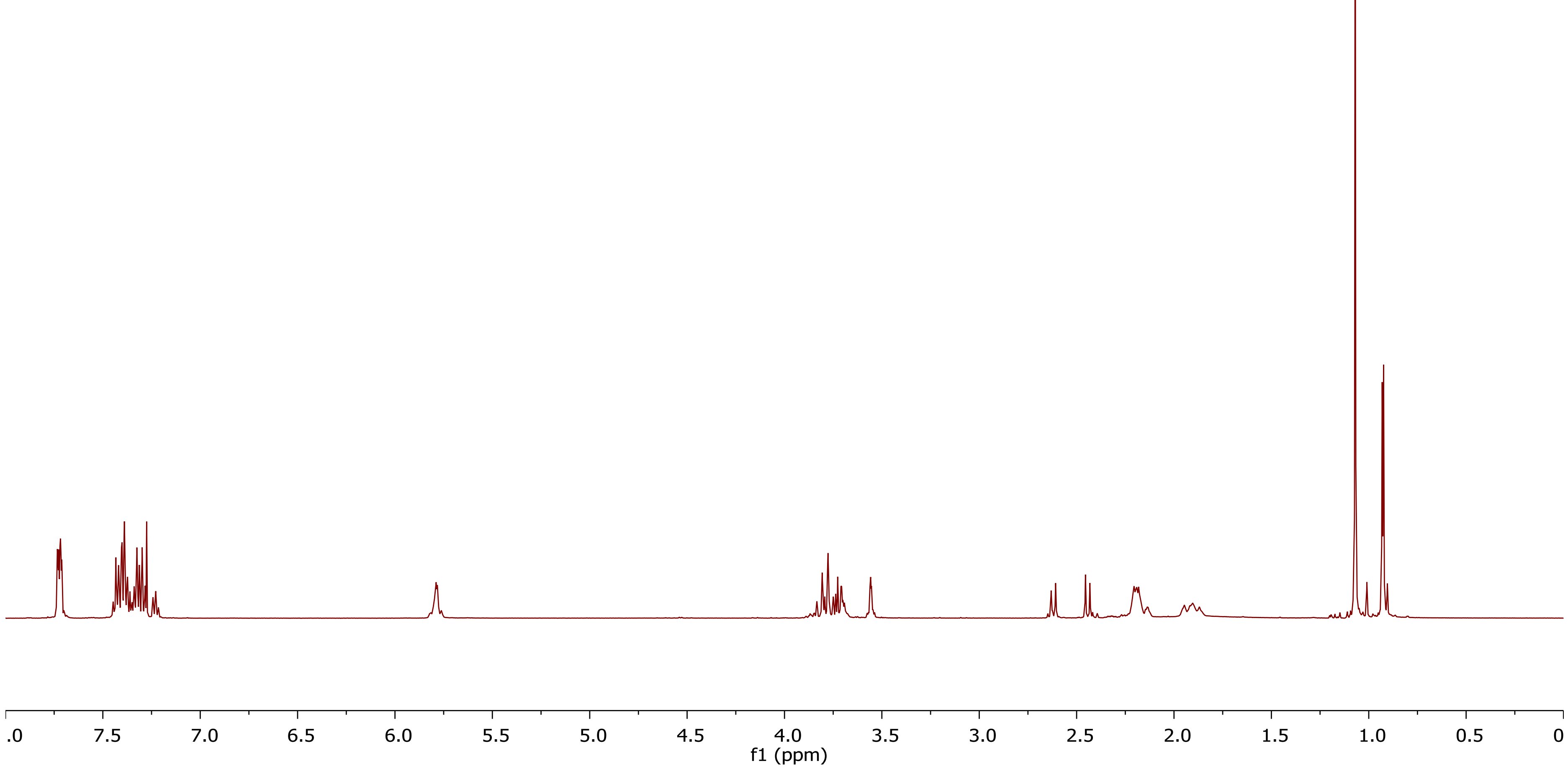

{2-[(1R,3S,3aS,7aR)-3-{[(*tert*-Butyldiphenylsilyl)oxy]methyl}-1,3,3a,4,7,7a-hexahydro-2-benzofuran-1-yl]-2-methylpropyl}(benzyl)amine **50**

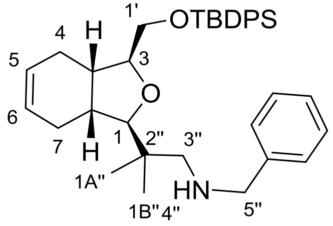

50

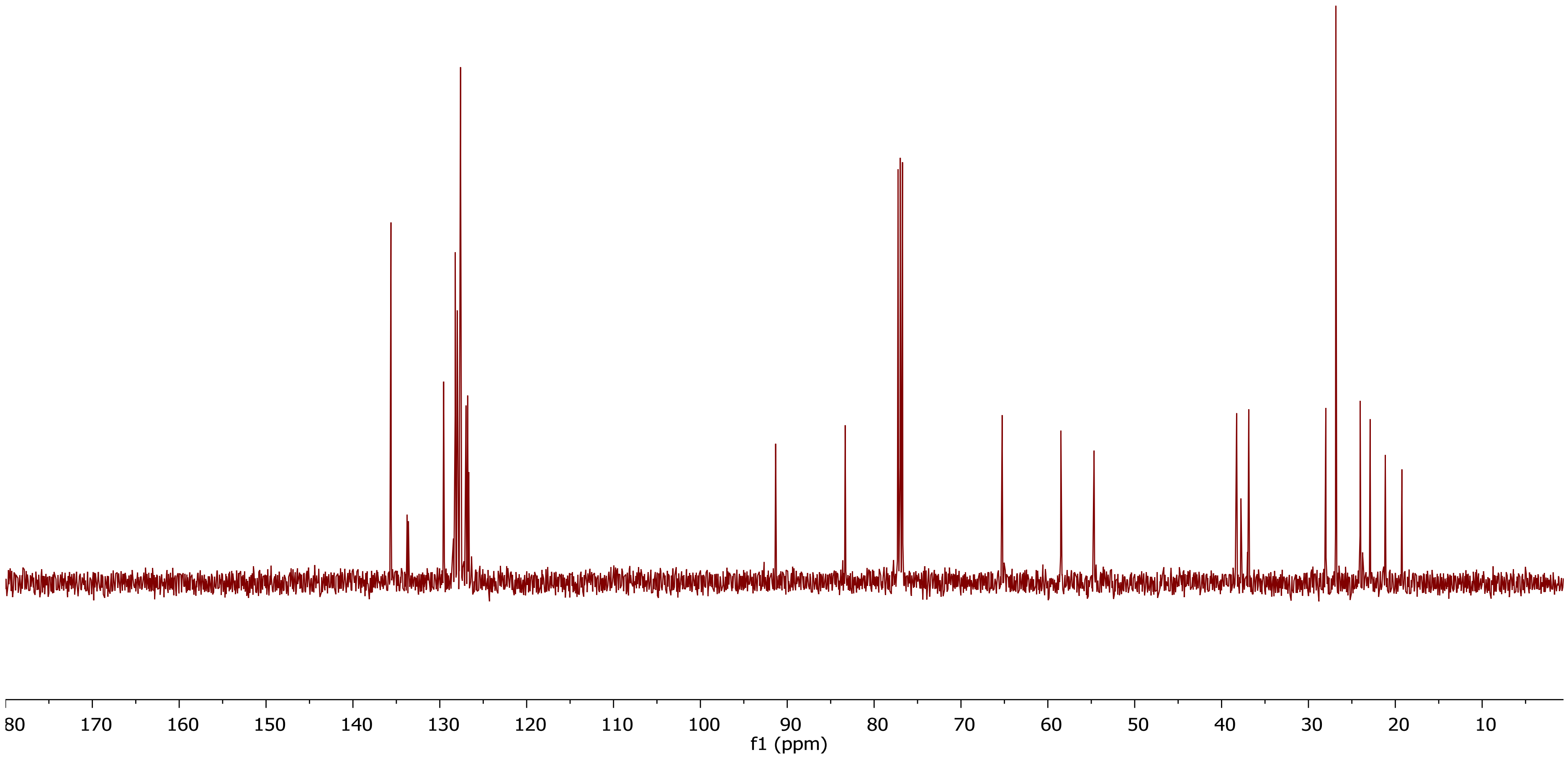

{2-[(1R,3S,3aS,7aR)-3-{[(*tert*-Butyldiphenylsilyl)oxy]methyl}-1,3,3a,4,7,7a-hexahydro-2-benzofuran-1-yl]-2-methylpropyl}[(3-methoxyphenyl)methyl]amine **51**

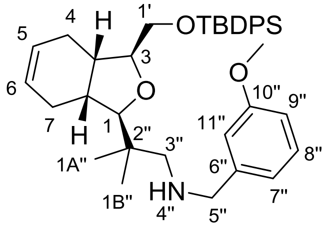

51

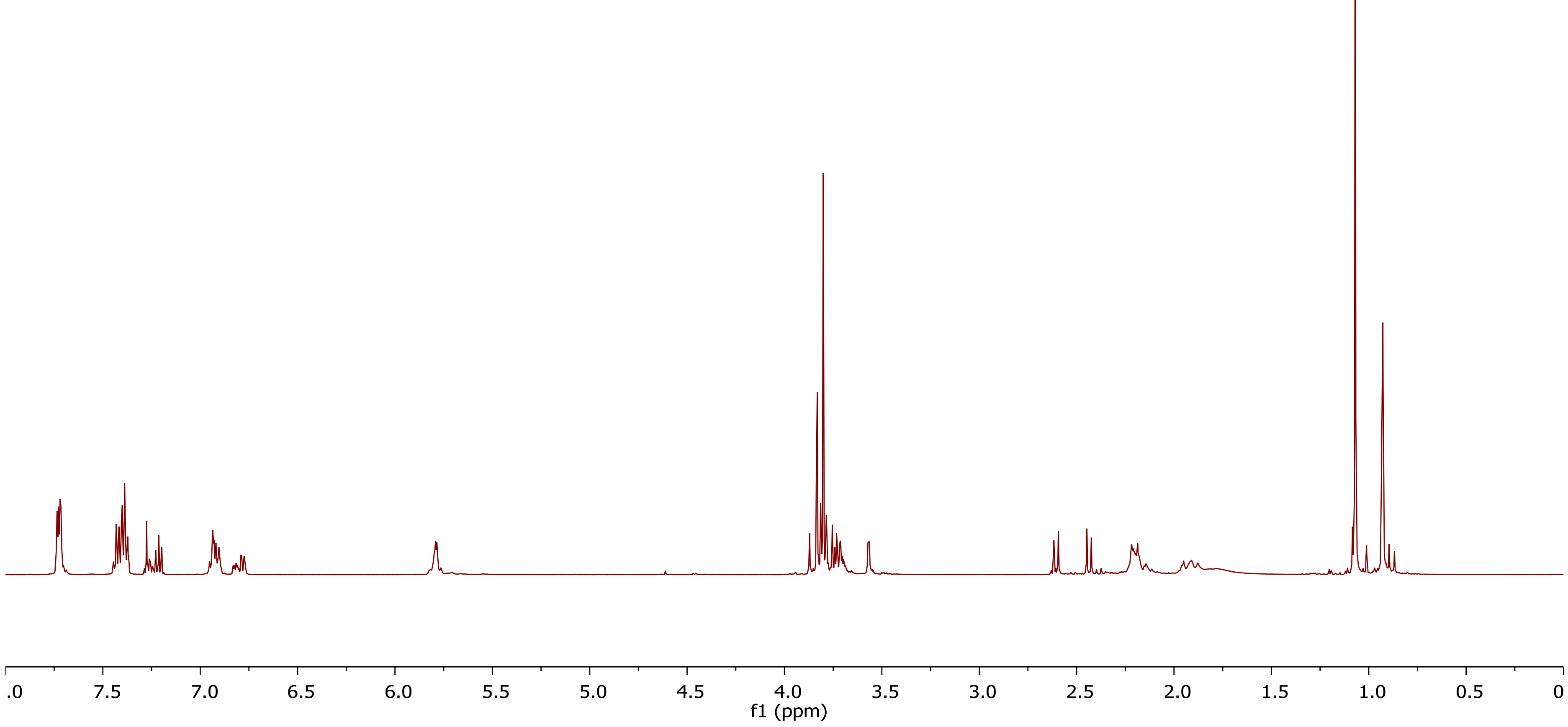

{2-[(1R,3S,3aS,7aR)-3-{[(*tert*-Butyldiphenylsilyl)oxy]methyl}-1,3,3a,4,7,7a-hexahydro-2-benzofuran-1-yl]-2-methylpropyl}[(3-methoxyphenyl)methyl]amine **51**

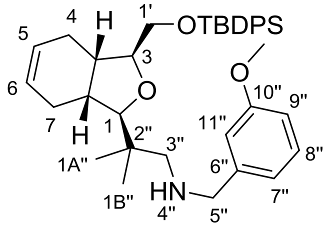

51

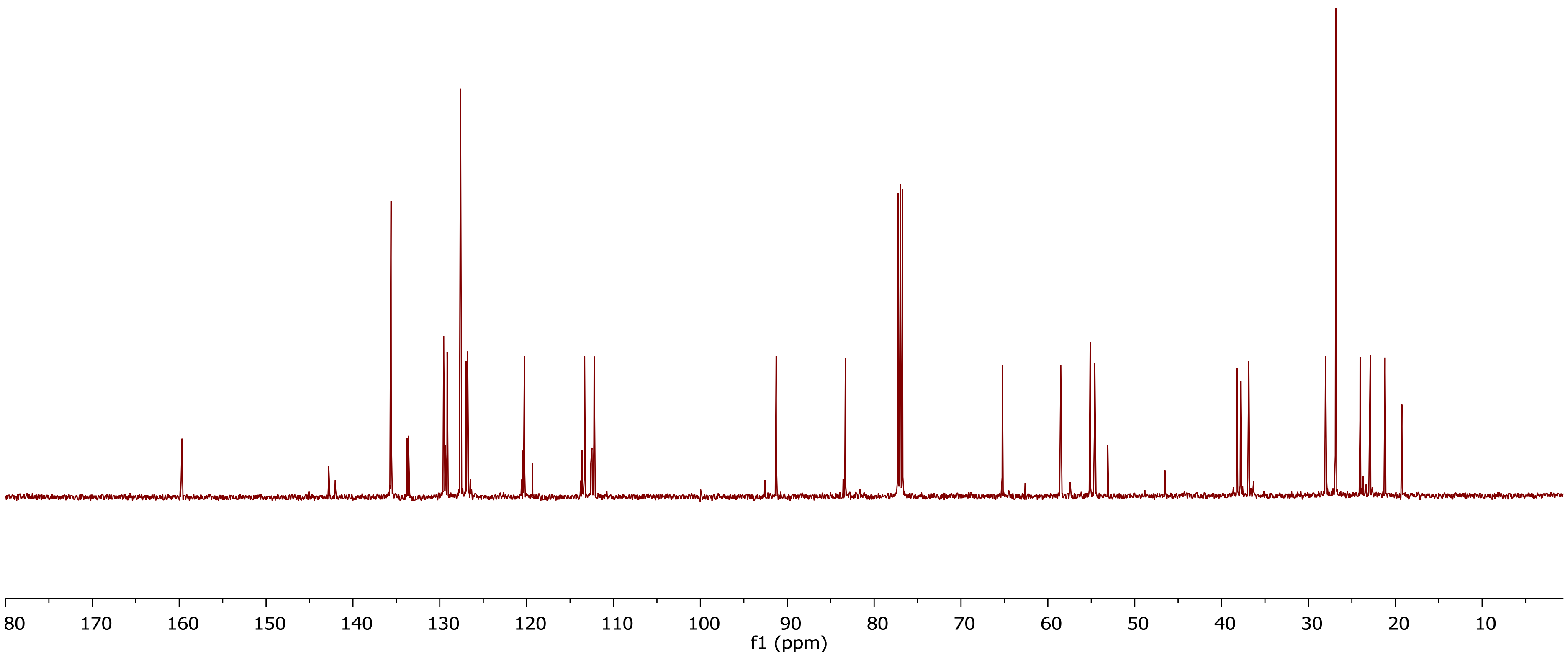

{2-[(1R,3S,3aS,7aR)-3-{[(*tert*-Butyldiphenylsilyl)oxy]methyl}-1,3,3a,4,7,7a-hexahydro-2-benzofuran-1-yl]-2-methylpropyl}(methyl)amine **52**

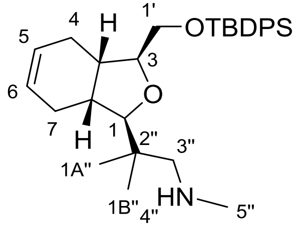

52

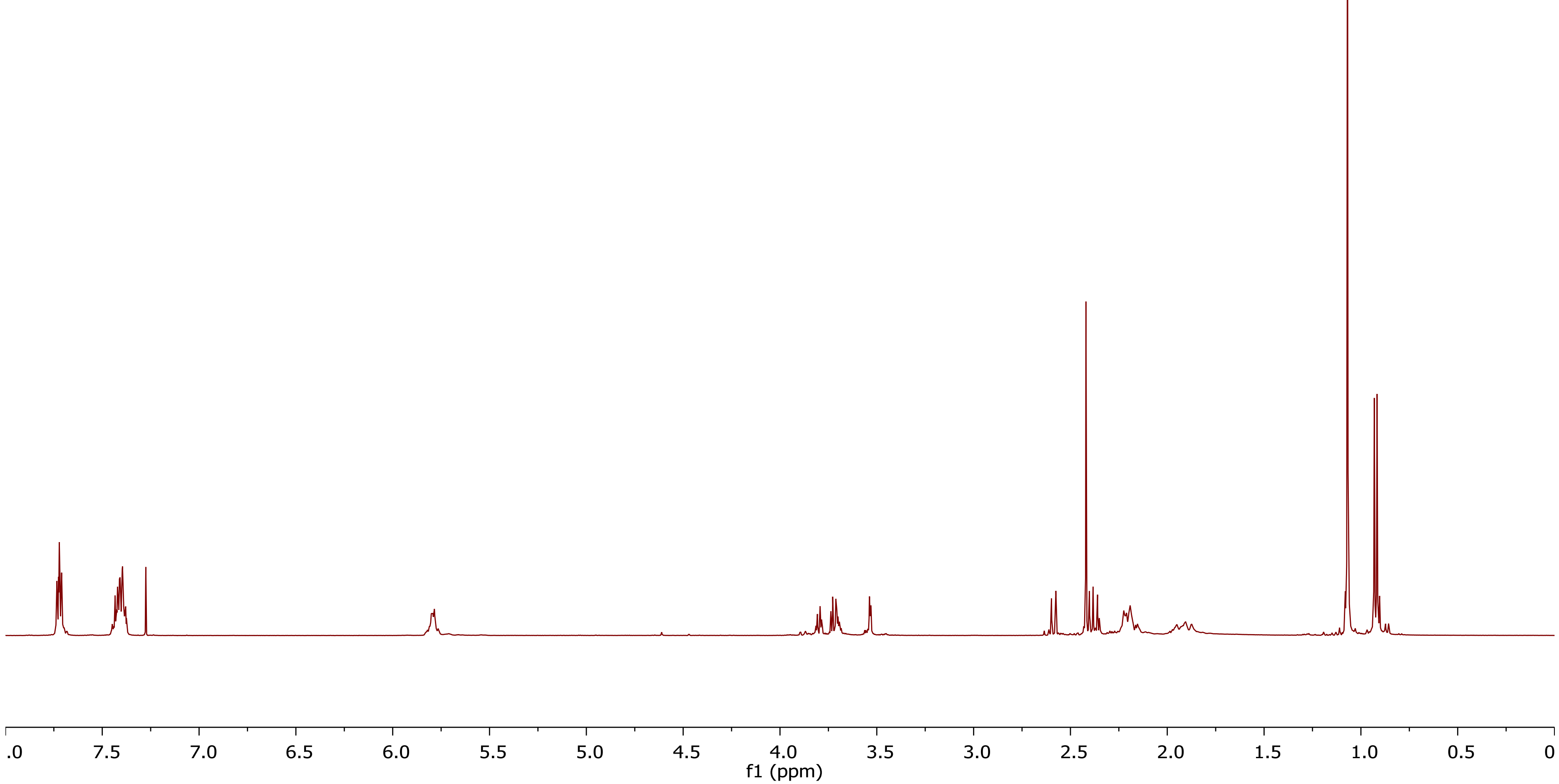

{2-[(1R,3S,3aS,7aR)-3-{[(*tert*-Butyldiphenylsilyl)oxy]methyl}-1,3,3a,4,7,7a-hexahydro-2-benzofuran-1-yl]-2-methylpropyl}(methyl)amine **52**

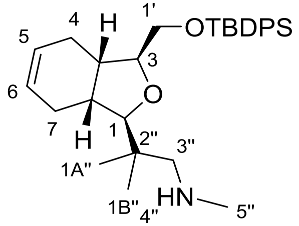

52

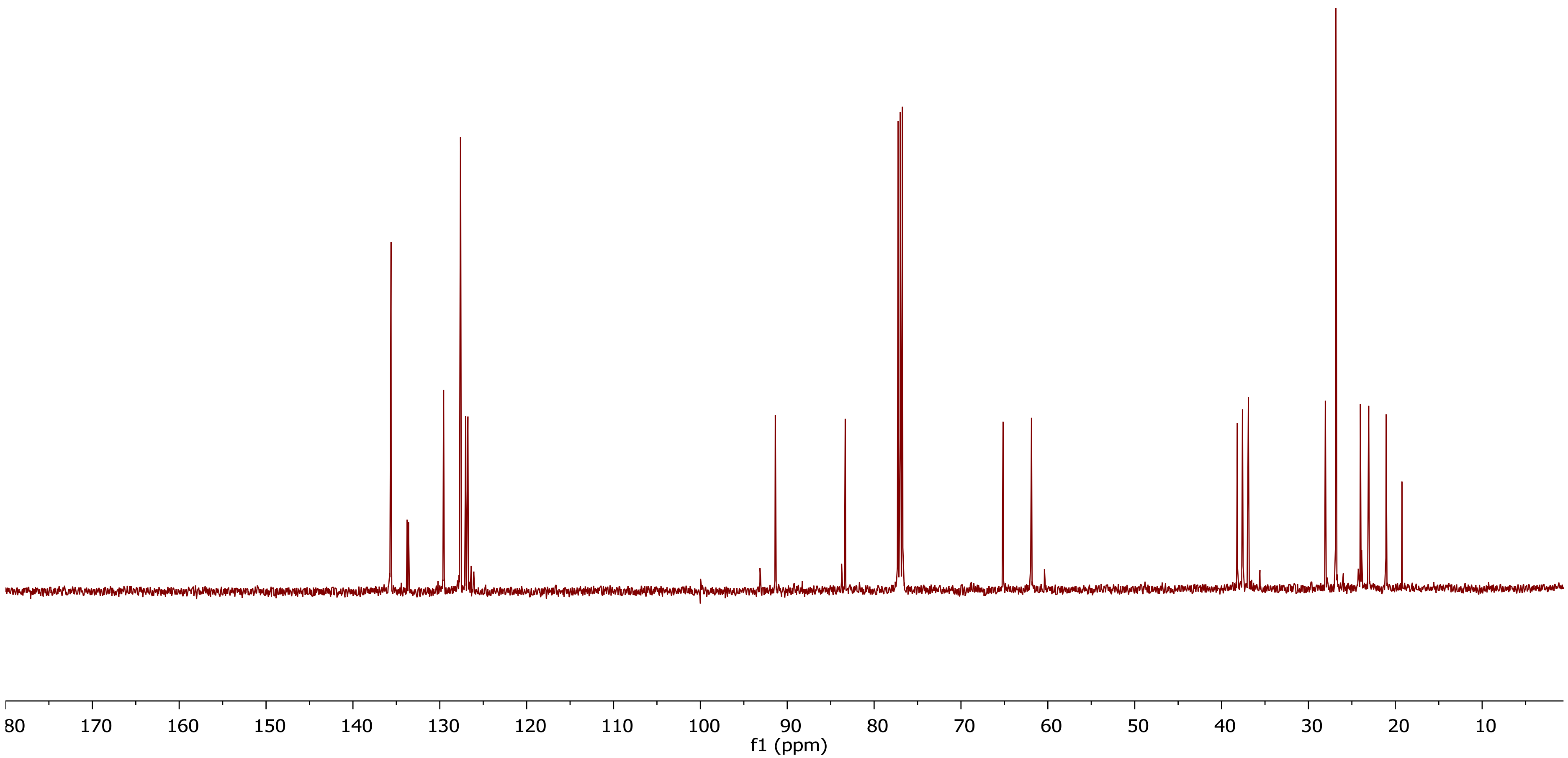

{2-[(1R,3S,3aS,7aR)-3-{[(*tert*-Butyldiphenylsilyl)oxy]methyl}-1,3,3a,4,7,7a-hexahydro-2-benzofuran-1-yl]-2-methylpropyl}(propyl)amine **53**

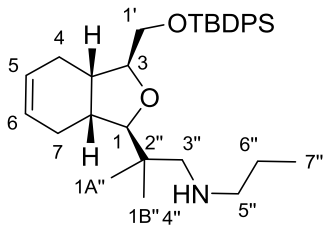

53

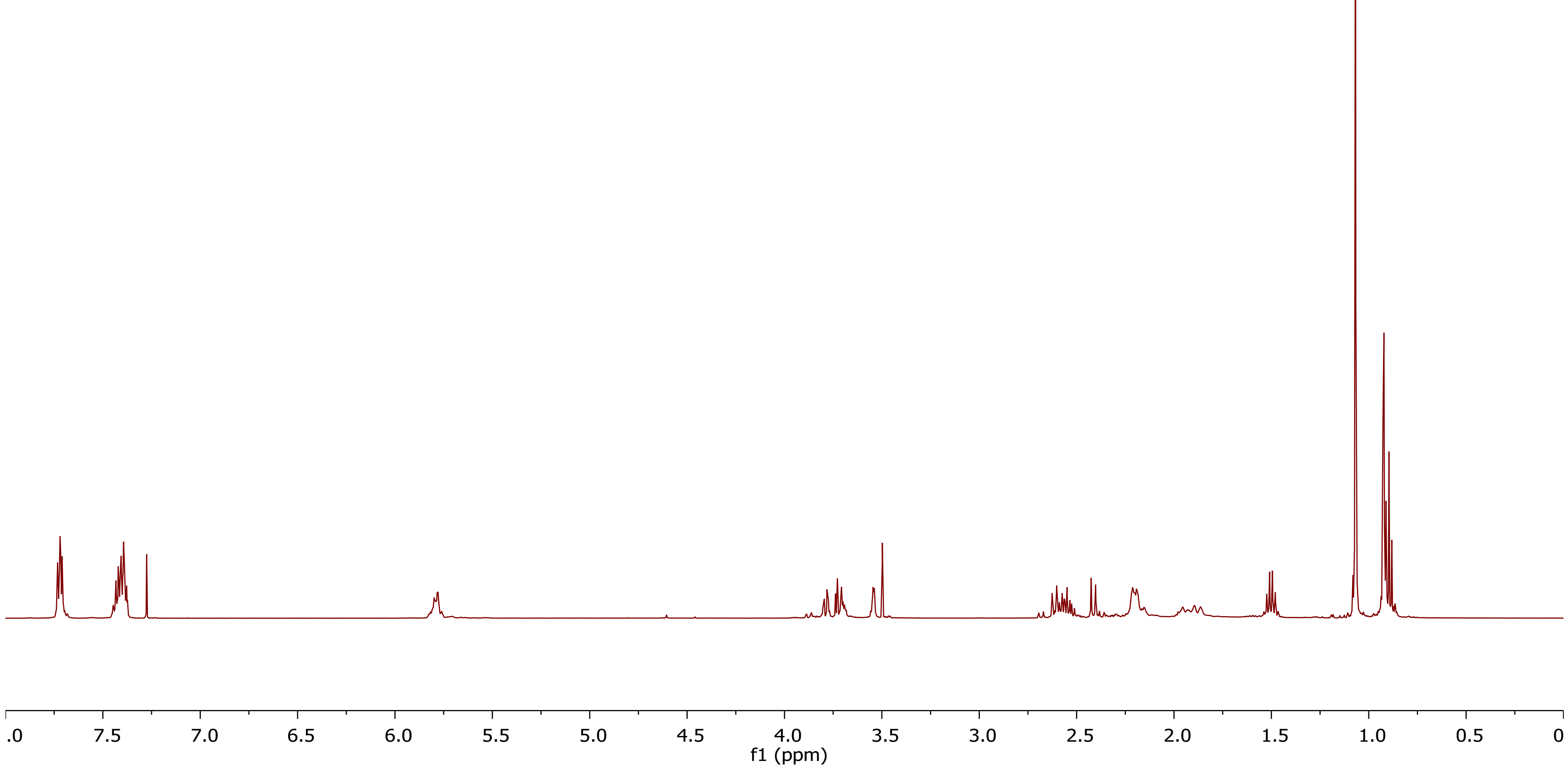

{2-[(1R,3S,3aS,7aR)-3-{[(*tert*-Butyldiphenylsilyl)oxy]methyl}-1,3,3a,4,7,7a-hexahydro-2-benzofuran-1-yl]-2-methylpropyl}(propyl)amine **53**

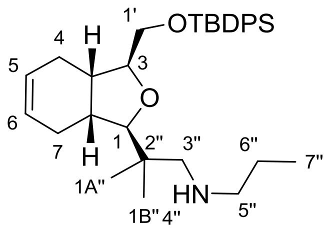

53

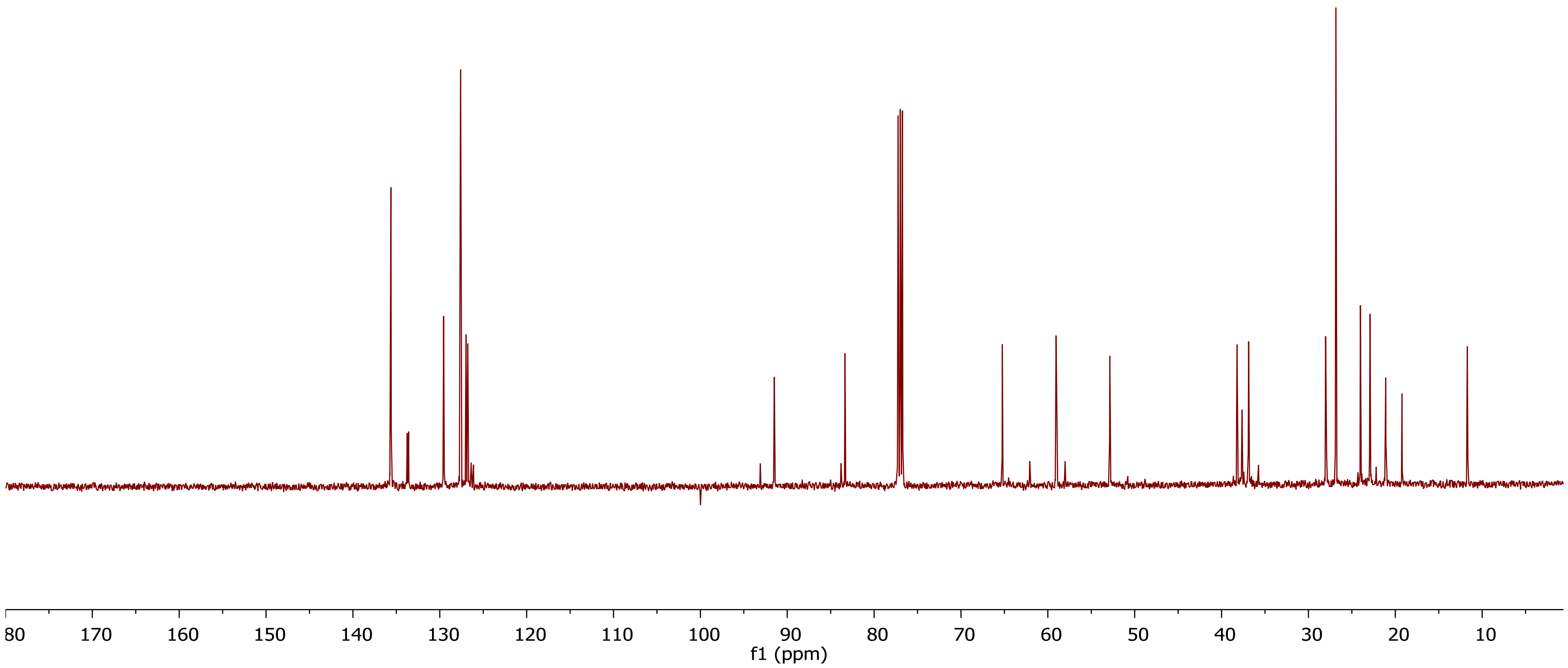

{2-[(1R,3S,3aS,7aR)-3-{[(*tert*-Butyldiphenylsilyl)oxy]methyl}-1,3,3a,4,7,7a-hexahydro-2-benzofuran-1-yl]-2-methylpropyl}(2-methoxyethyl)amine **54**

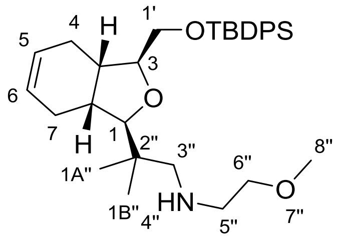

54

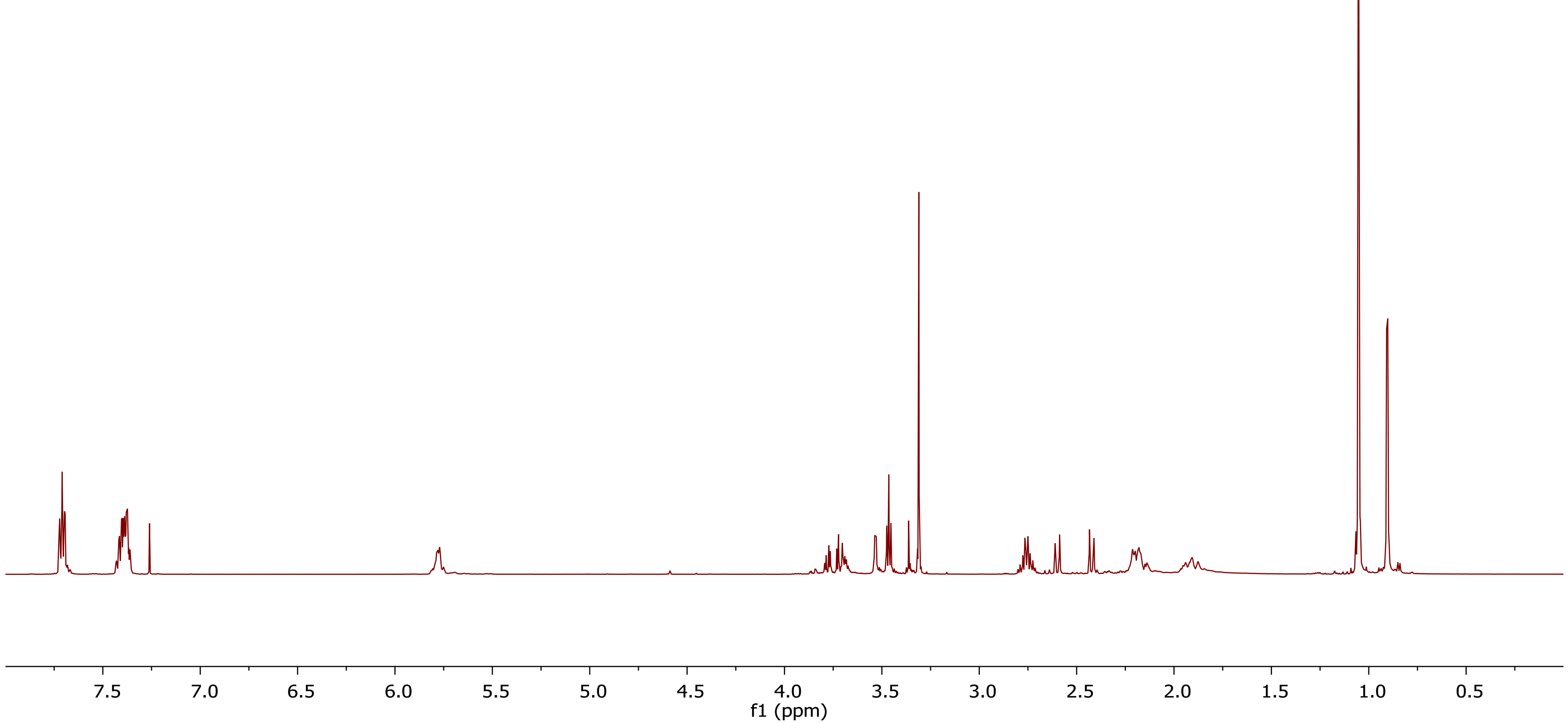

{2-[(1R,3S,3aS,7aR)-3-{[(*tert*-Butyldiphenylsilyl)oxy]methyl}-1,3,3a,4,7,7a-hexahydro-2-benzofuran-1-yl]-2-methylpropyl}(2-methoxyethyl)amine **54**

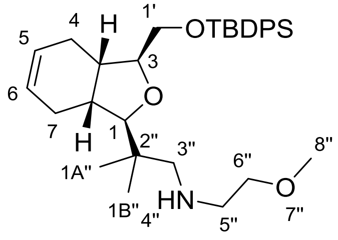

54

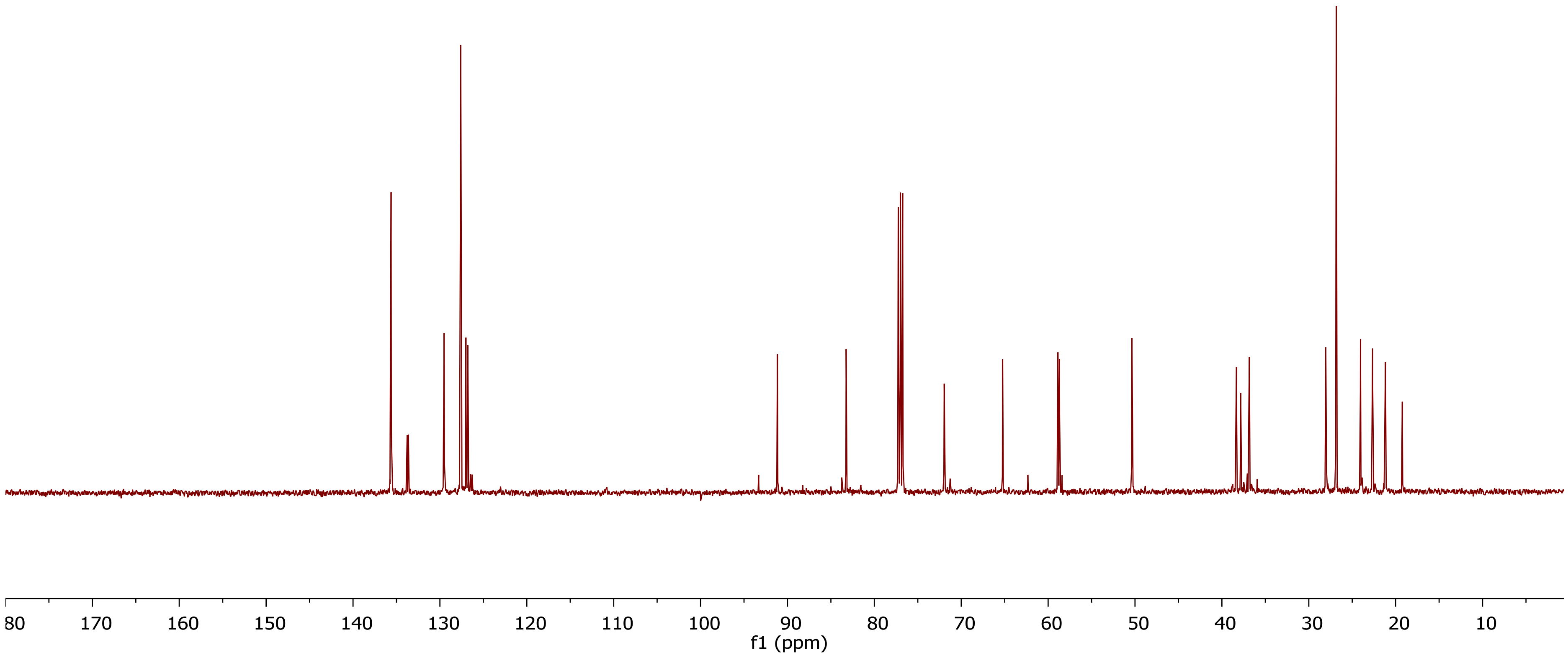

[(1S,3R,3aR,7aS)-3-[1-(benzylamino)-2-methylpropan-2-yl]-1,3,3a,4,7,7a-hexahydro-2-benzofuran-1-yl]methanol **55**

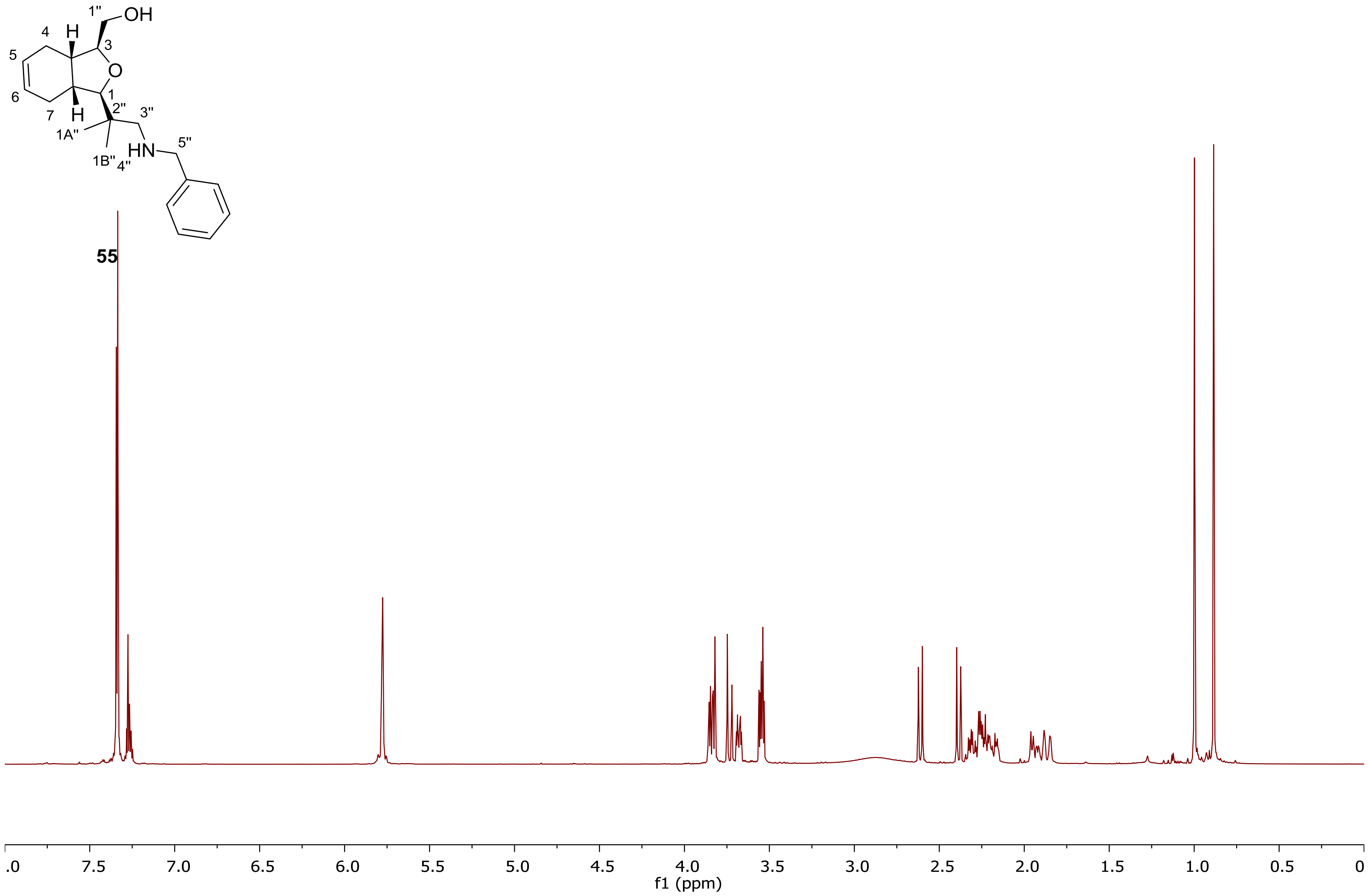

[(1S,3R,3aR,7aS)-3-[1-(benzylamino)-2-methylpropan-2-yl]-1,3,3a,4,7,7a-hexahydro-2-benzofuran-1-yl]methanol **55**

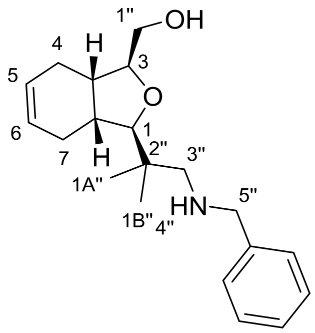

55

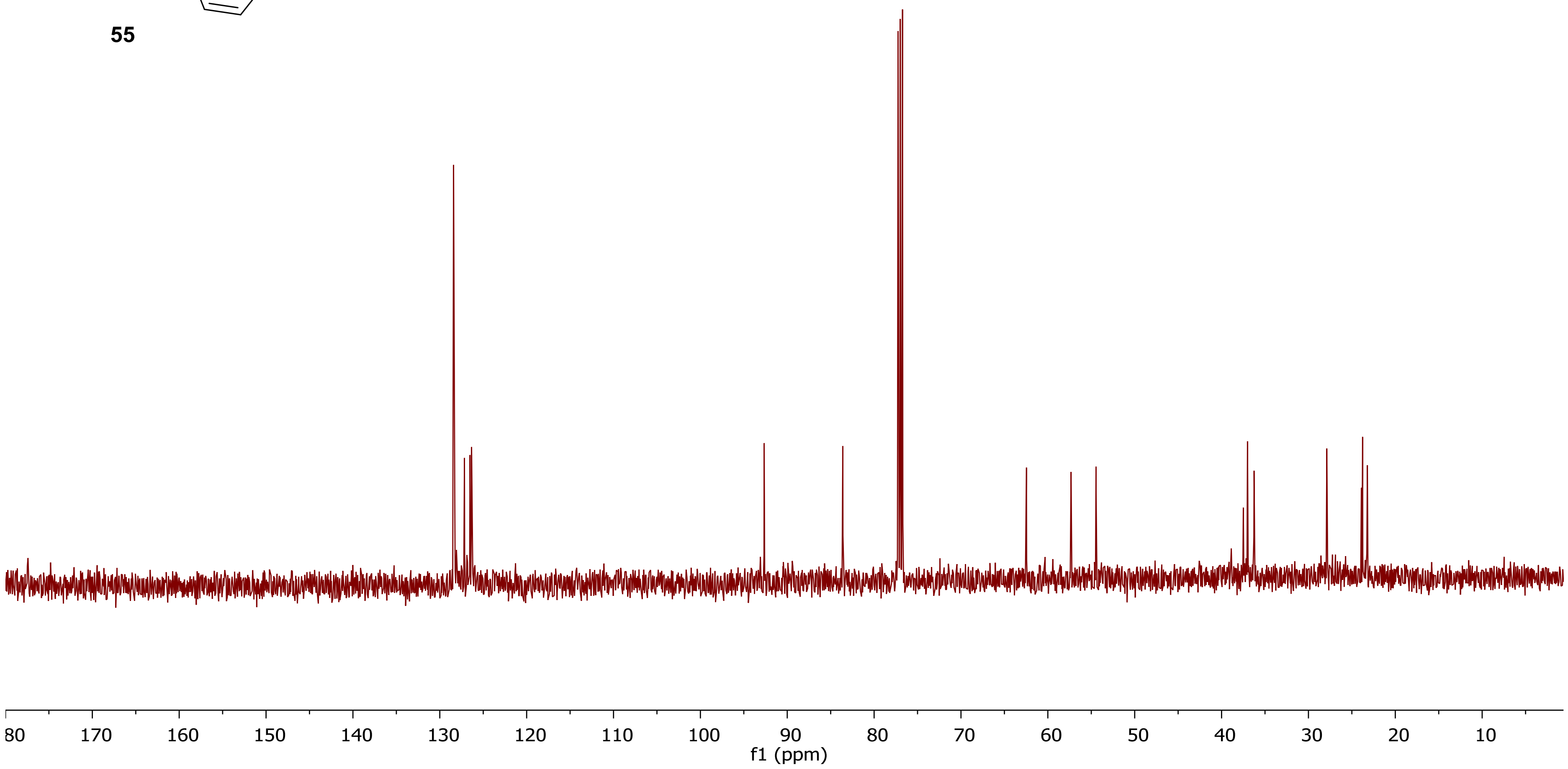

[(1S,3R,3aR,7aS)-3-(1-{[(3-Methoxyphenyl)methyl]amino}-2-methylpropan-2-yl)-1,3,3a,4,7,7a-hexahydro-2-benzofuran-1-yl]methanol **56**

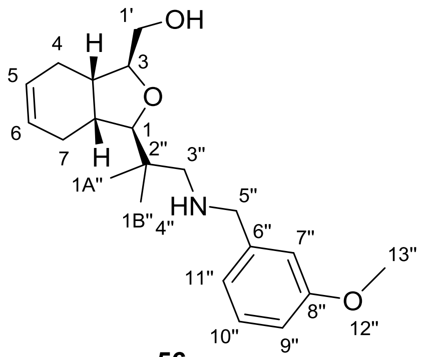

56

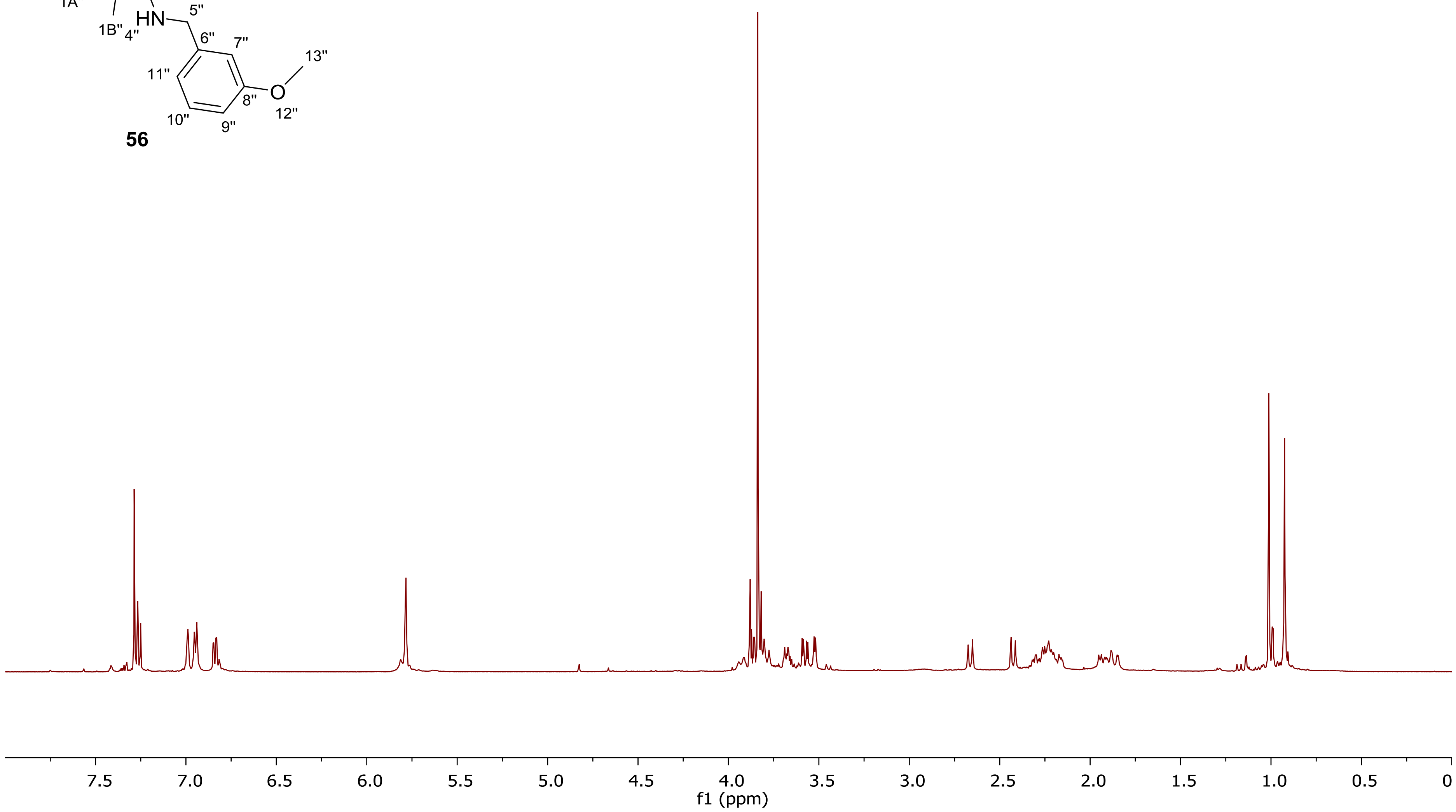

[(1S,3R,3aR,7aS)-3-(1-{[(3-Methoxyphenyl)methyl]amino}-2-methylpropan-2-yl)-1,3,3a,4,7,7a-hexahydro-2-benzofuran-1-yl]methanol **56**

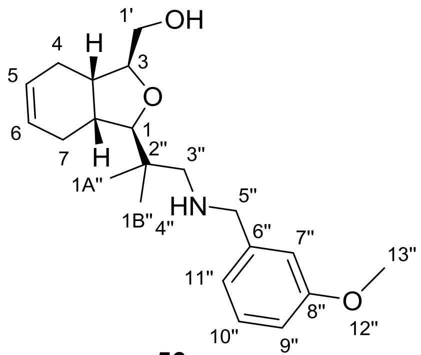

56

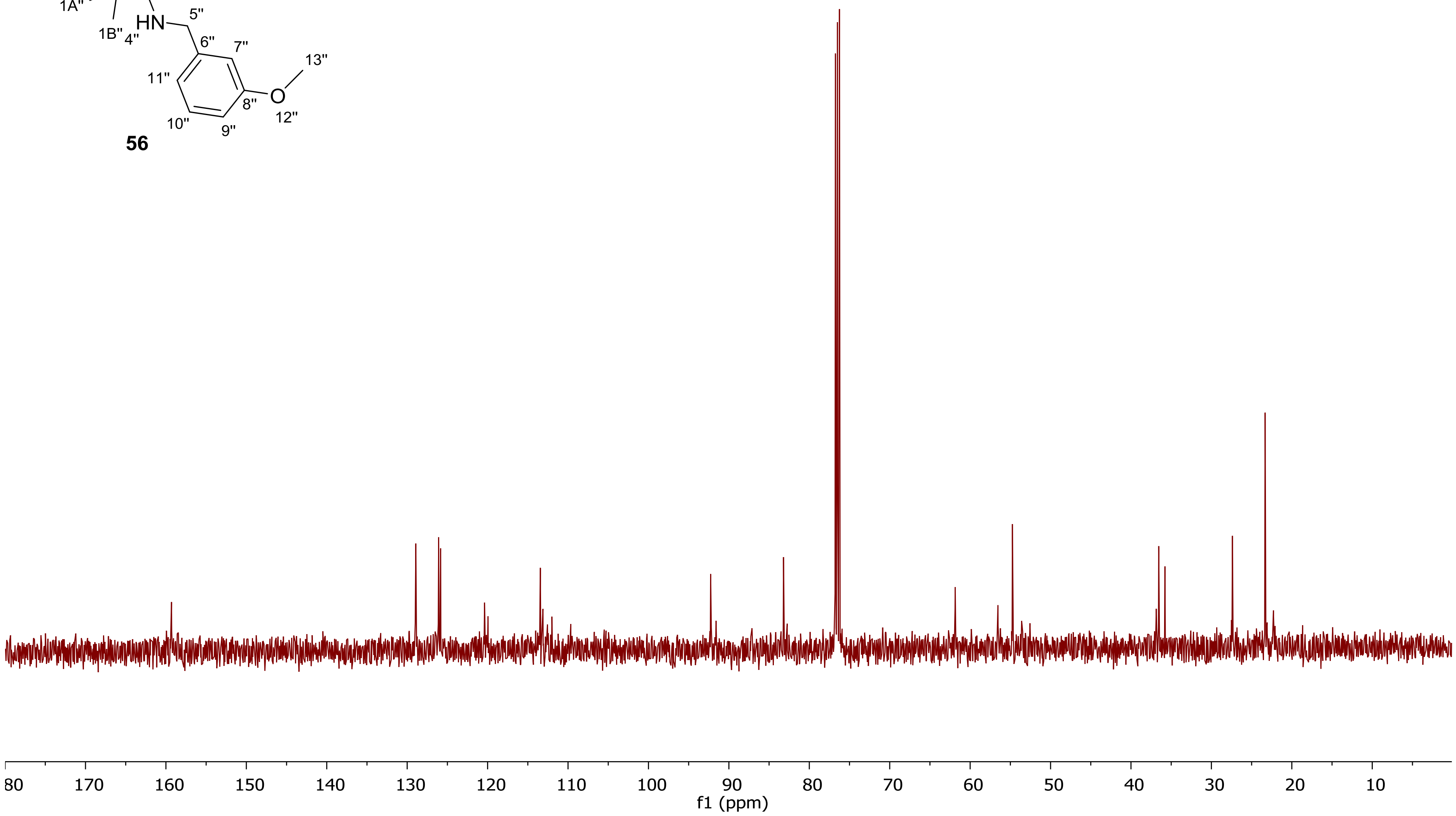

[(1S,3R,3aR,7aS)-3-[2-Methyl-1-(methylamino)propan-2-yl]-1,3,3a,4,7,7a-hexahydro-2-benzofuran-1-yl]methanol **57**

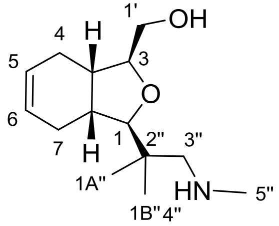

57

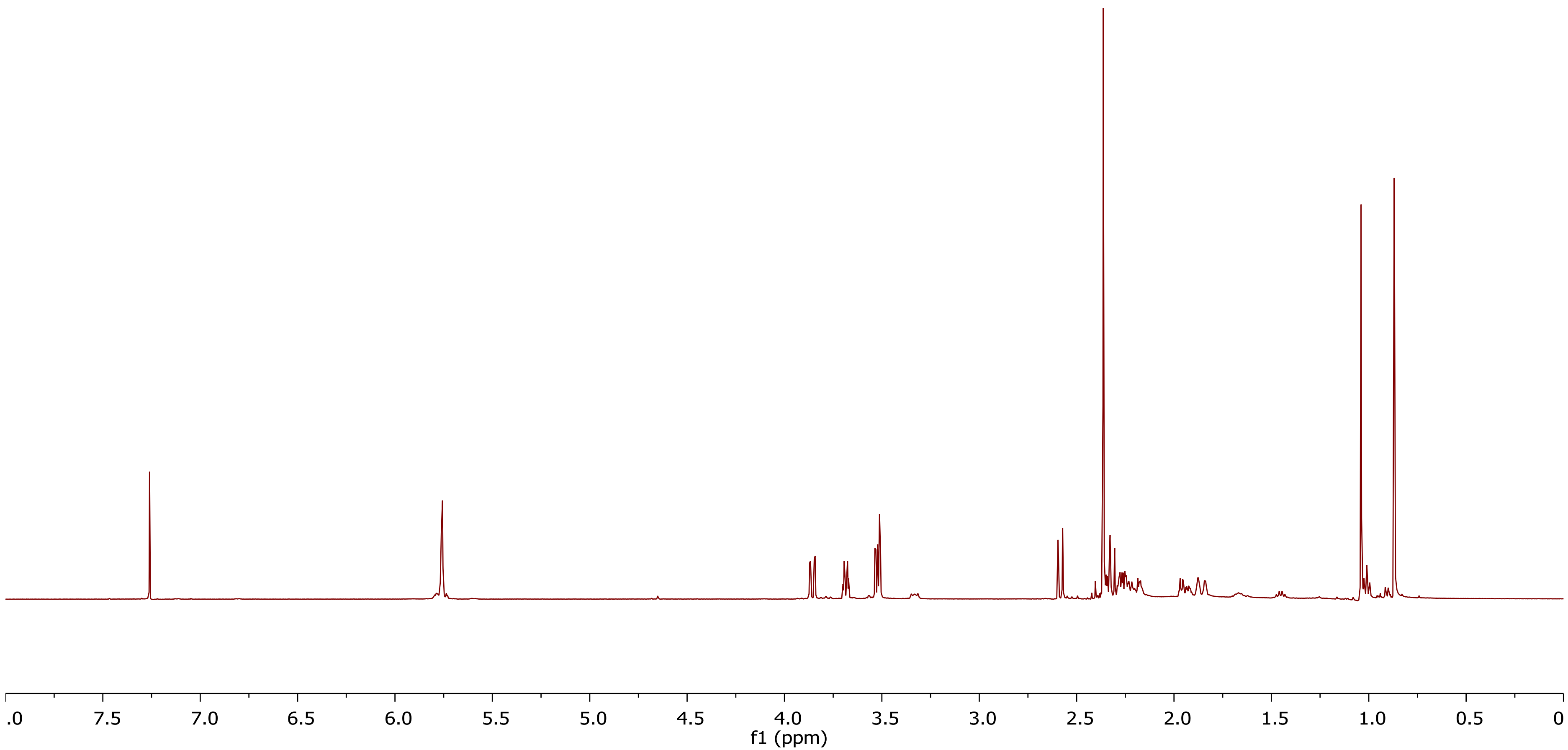

[(1S,3R,3aR,7aS)-3-[2-Methyl-1-(methylamino)propan-2-yl]-1,3,3a,4,7,7a-hexahydro-2-benzofuran-1-yl]methanol **57**

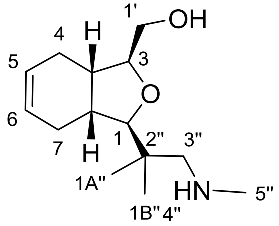

57

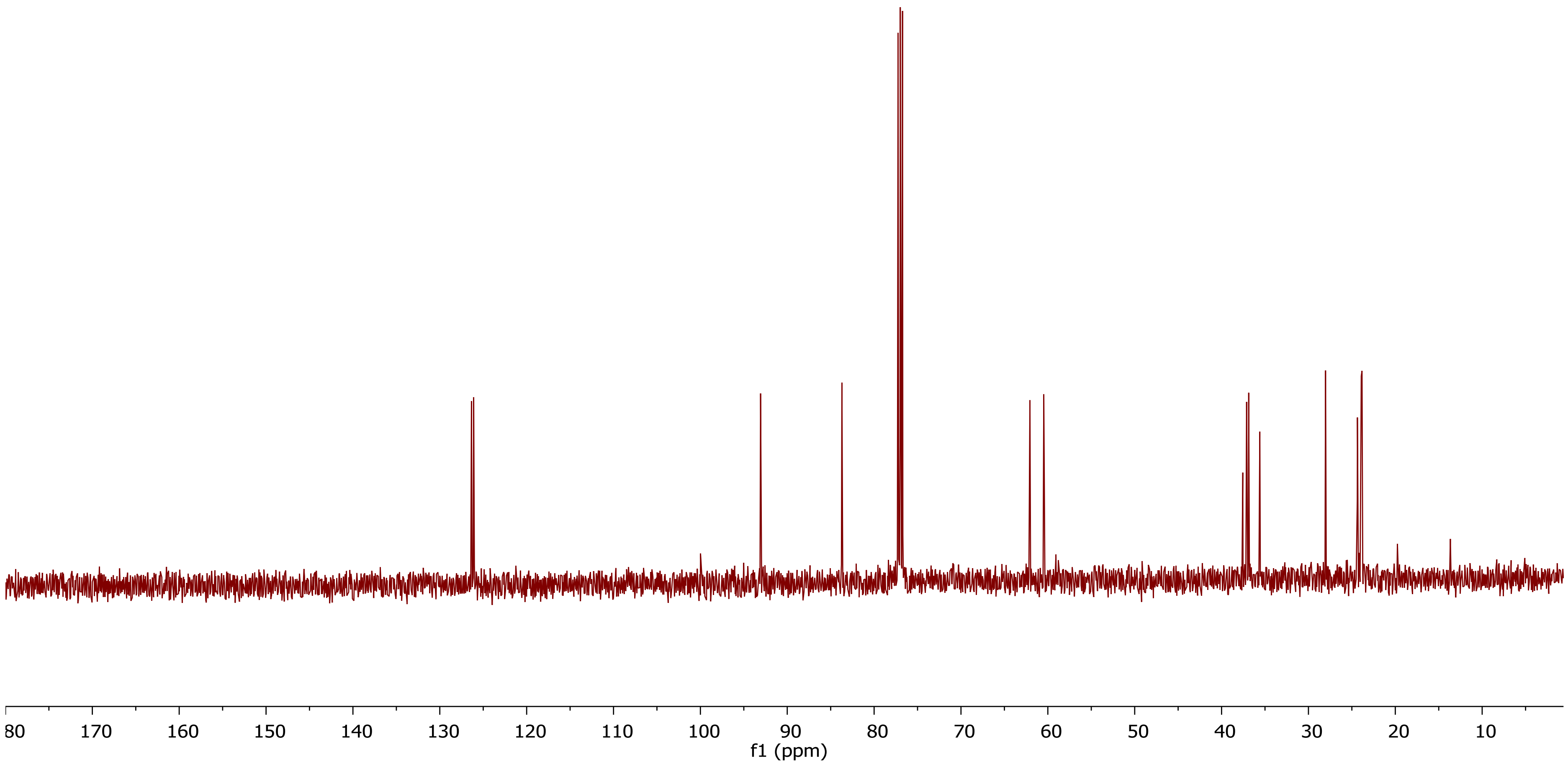

[(1S,3R,3aR,7aS)-3-[2-methyl-1-(propylamino)propan-2-yl]-1,3,3a,4,7,7a-hexahydro-2-benzofuran-1-yl]methanol **58**

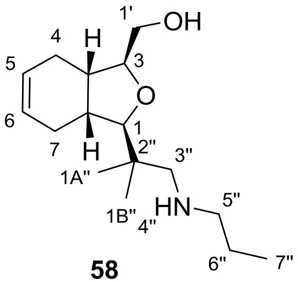

58

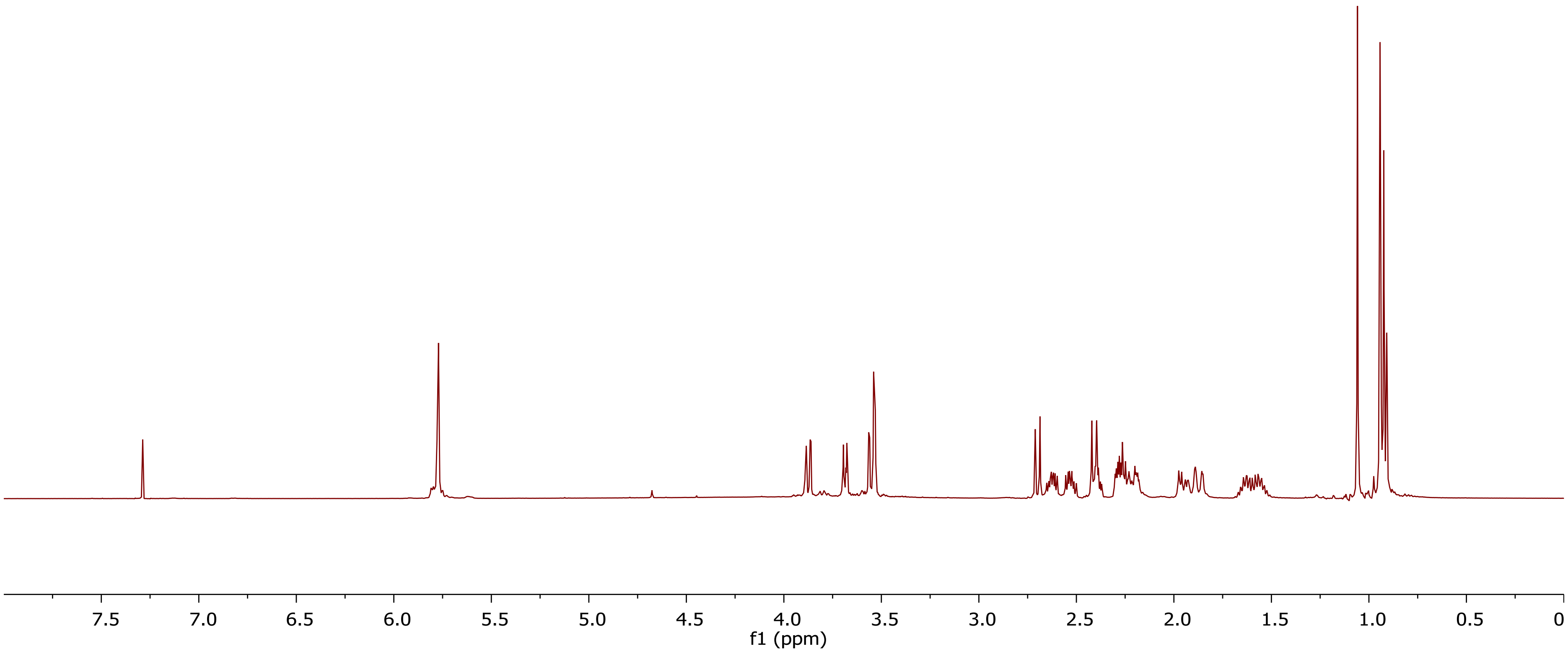

[(1S,3R,3aR,7aS)-3-[2-methyl-1-(propylamino)propan-2-yl]-1,3,3a,4,7,7a-hexahydro-2-benzofuran-1-yl]methanol **58**

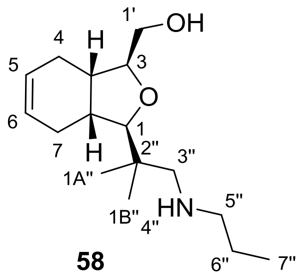

58

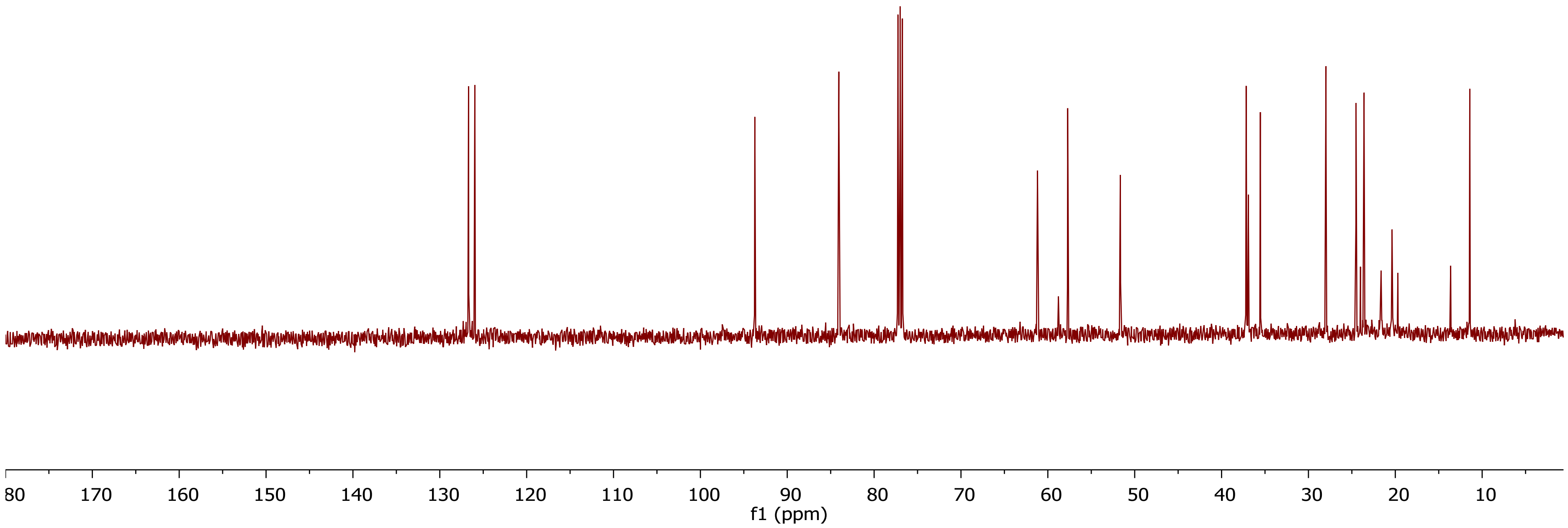

[(1S,3R,3aR,7aS)-3-{1-[(2-Methoxyethyl)amino]-2-methylpropan-2-yl}-1,3,3a,4,7,7a-hexahydro-2-benzofuran-1-yl]methanol **59**

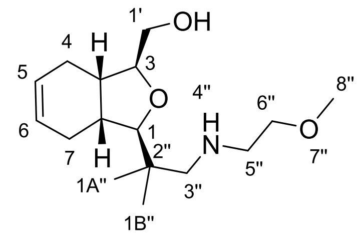

59

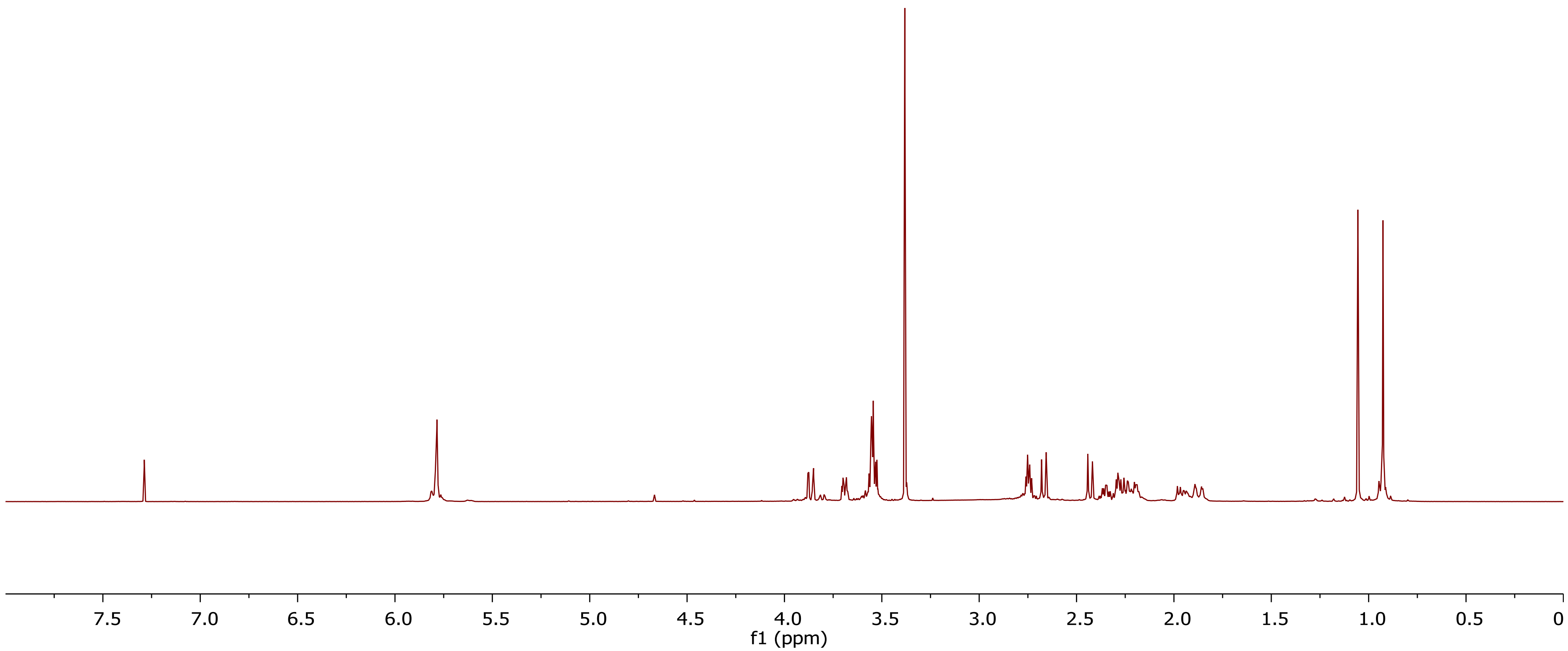

[(1S,3R,3aR,7aS)-3-{1-[(2-Methoxyethyl)amino]-2-methylpropan-2-yl}-1,3,3a,4,7,7a-hexahydro-2-benzofuran-1-yl]methanol **59**

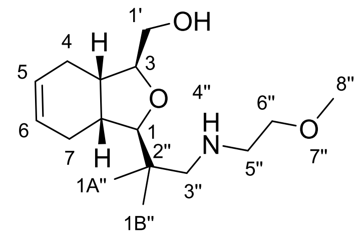

59

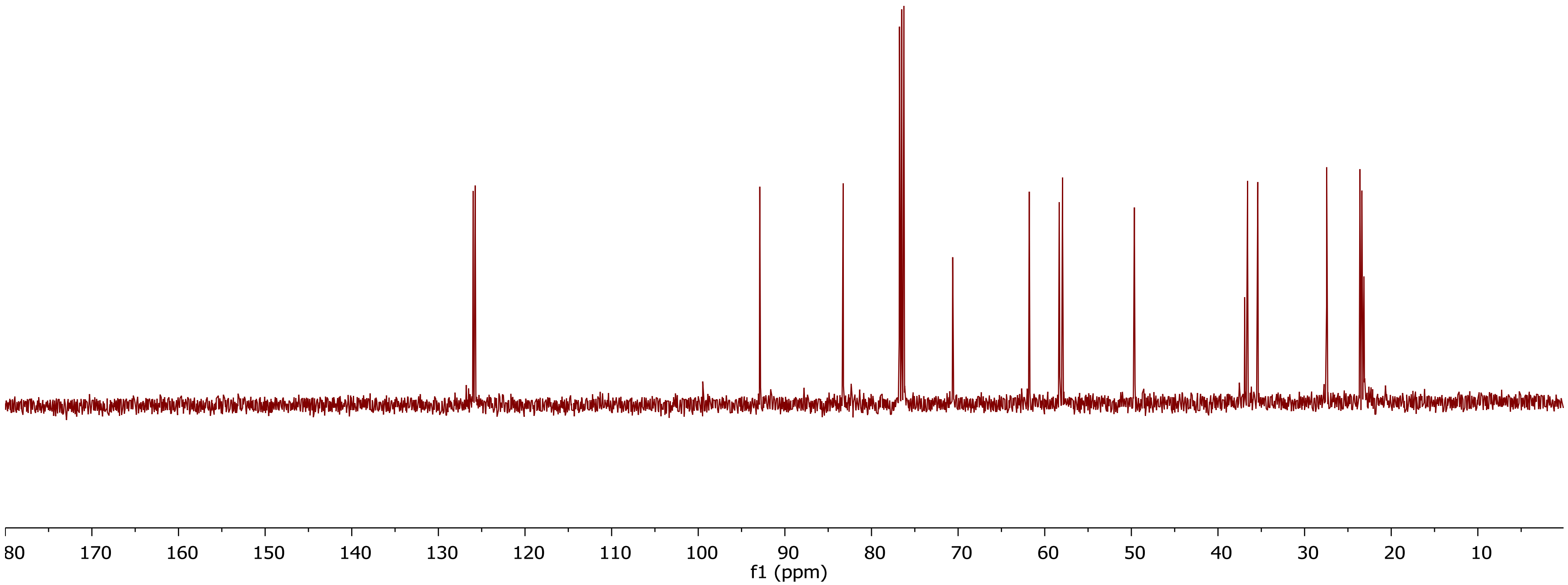

[(1S,3S,3aR,7aS)-3-(2-methoxy-3-phenylpropyl)-octahydro-2-benzofuran-1-yl]methanol **60**

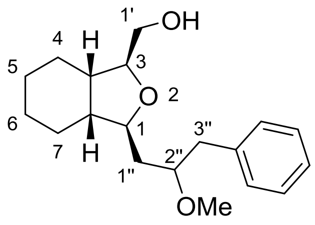

60

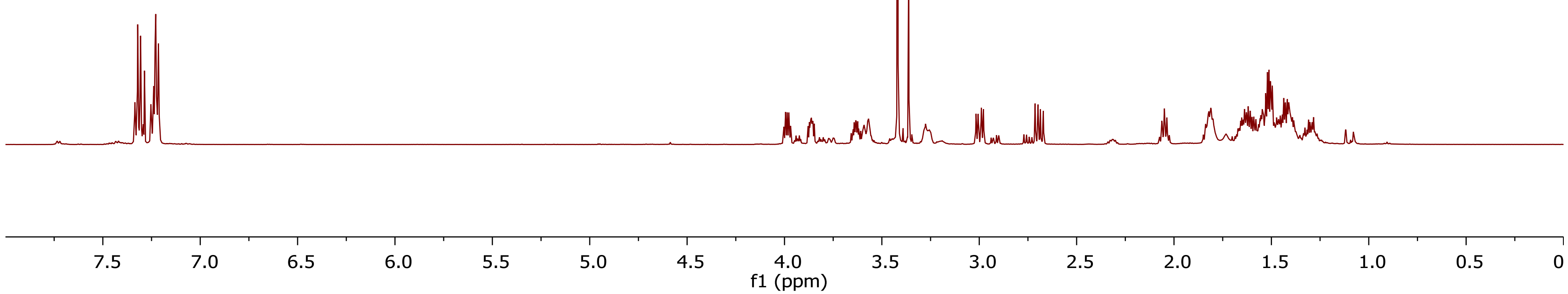

[(1S,3S,3aR,7aS)-3-(2-methoxy-3-phenylpropyl)-octahydro-2-benzofuran-1-yl]methanol **60**

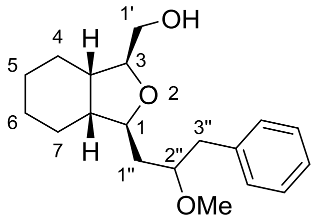

60

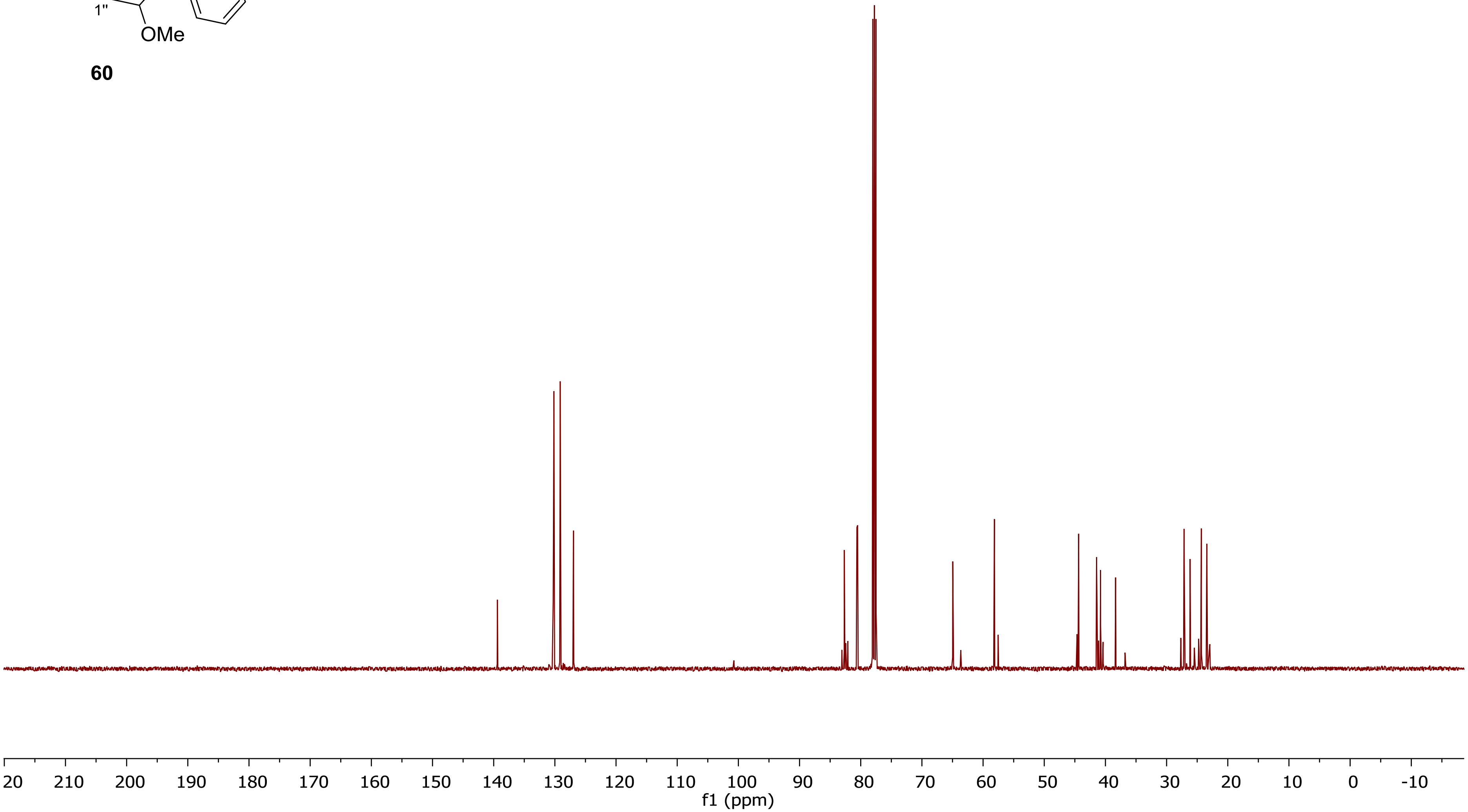

Ethyl (2E)-4-[(1R,3S,3aS,7aR)-3-{[(*tert*-Butyldiphenylsilyl)oxy]methyl}-1,3,3a,4,7,7a-hexahydro-2-benzofuran-1-yl]-4-methylpent-2-enoate **61**

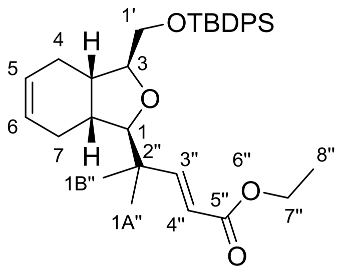

61

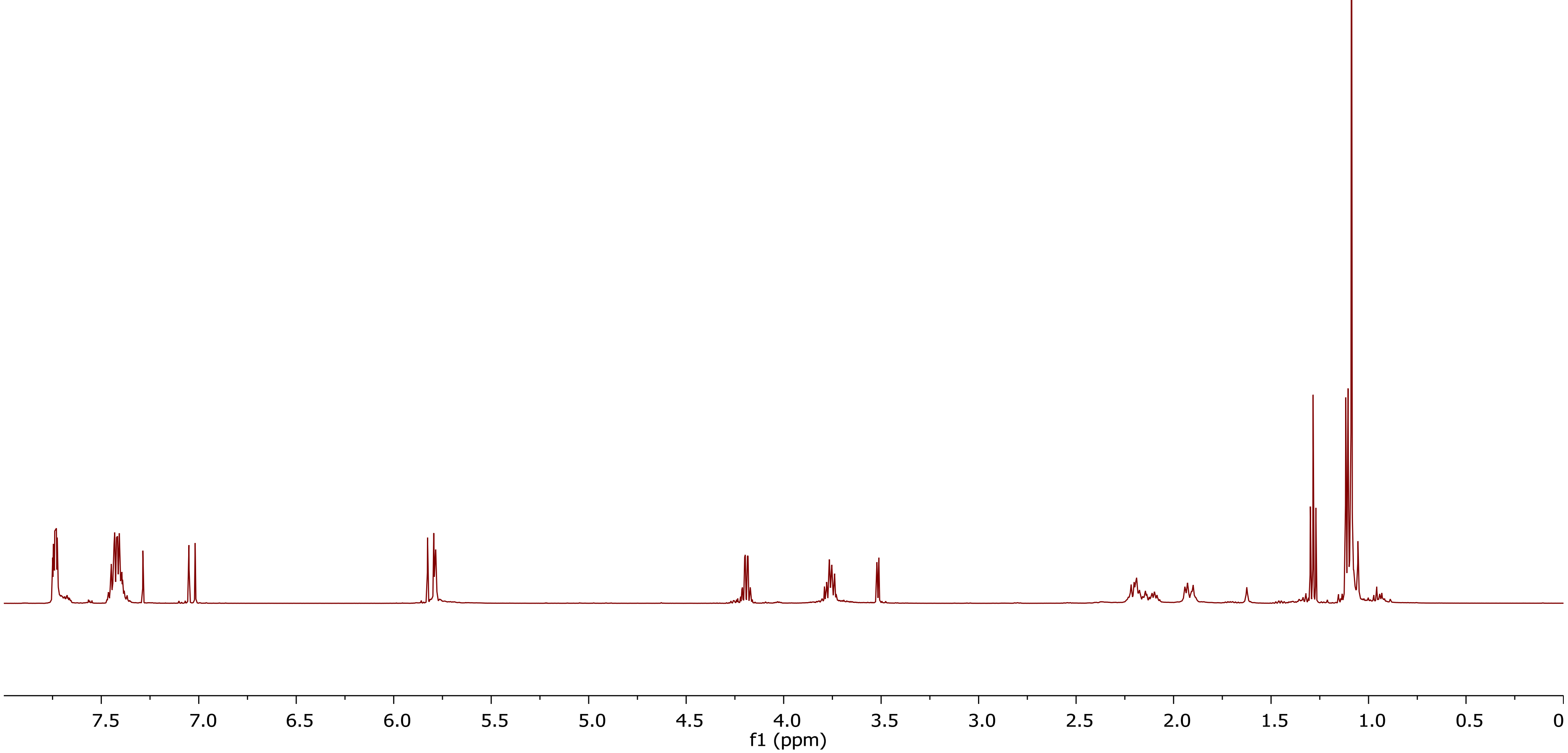

Ethyl (2E)-4-[(1R,3S,3aS,7aR)-3-{[(*tert*-Butyldiphenylsilyl)oxy]methyl}-1,3,3a,4,7,7a-hexahydro-2-benzofuran-1-yl]-4-methylpent-2-enoate **61**

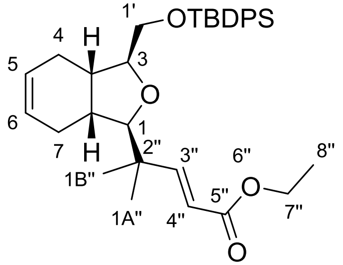

61

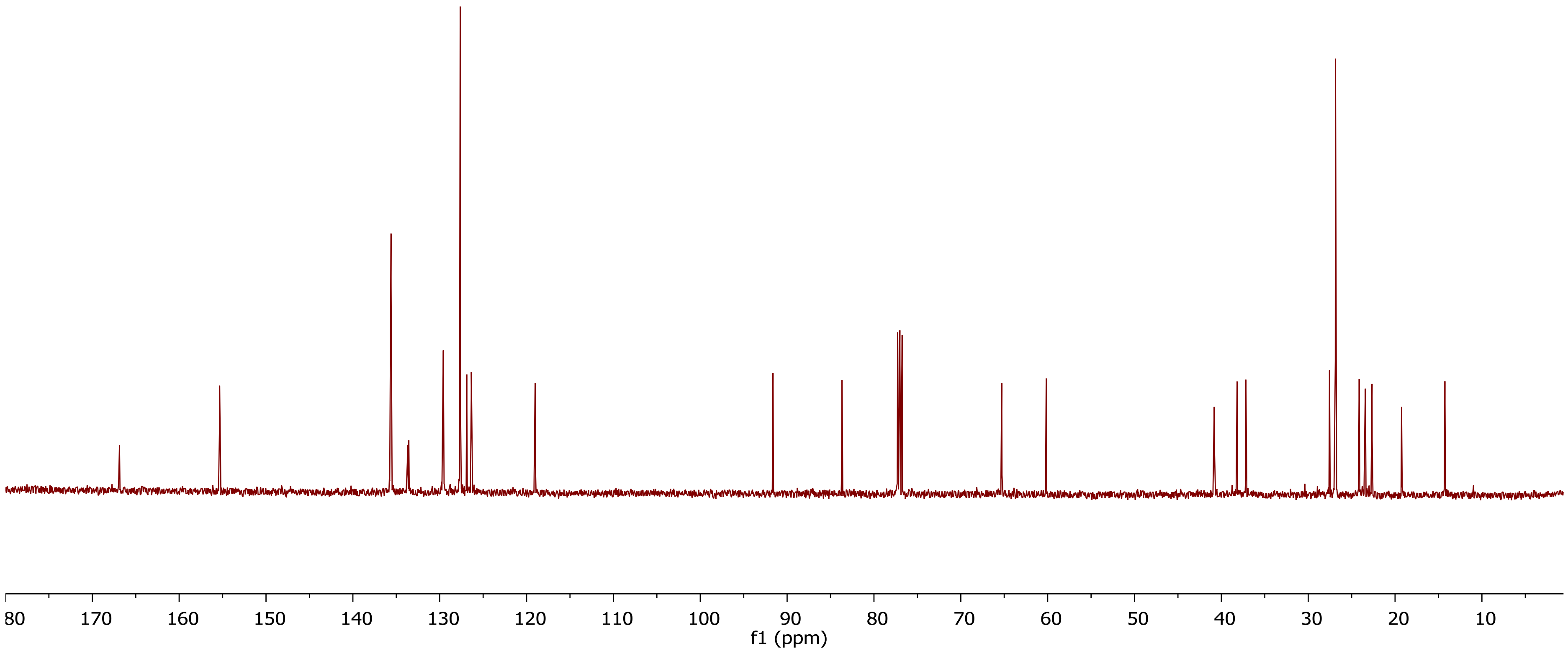

Ethyl 4-[(1R,3S,3aS,7aR)-3-{[(*tert*-butyldiphenylsilyl)oxy]methyl} octahydro-2-benzofuran-1-yl]-4-methylpentanoate **62**

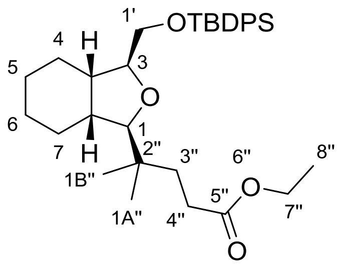

62

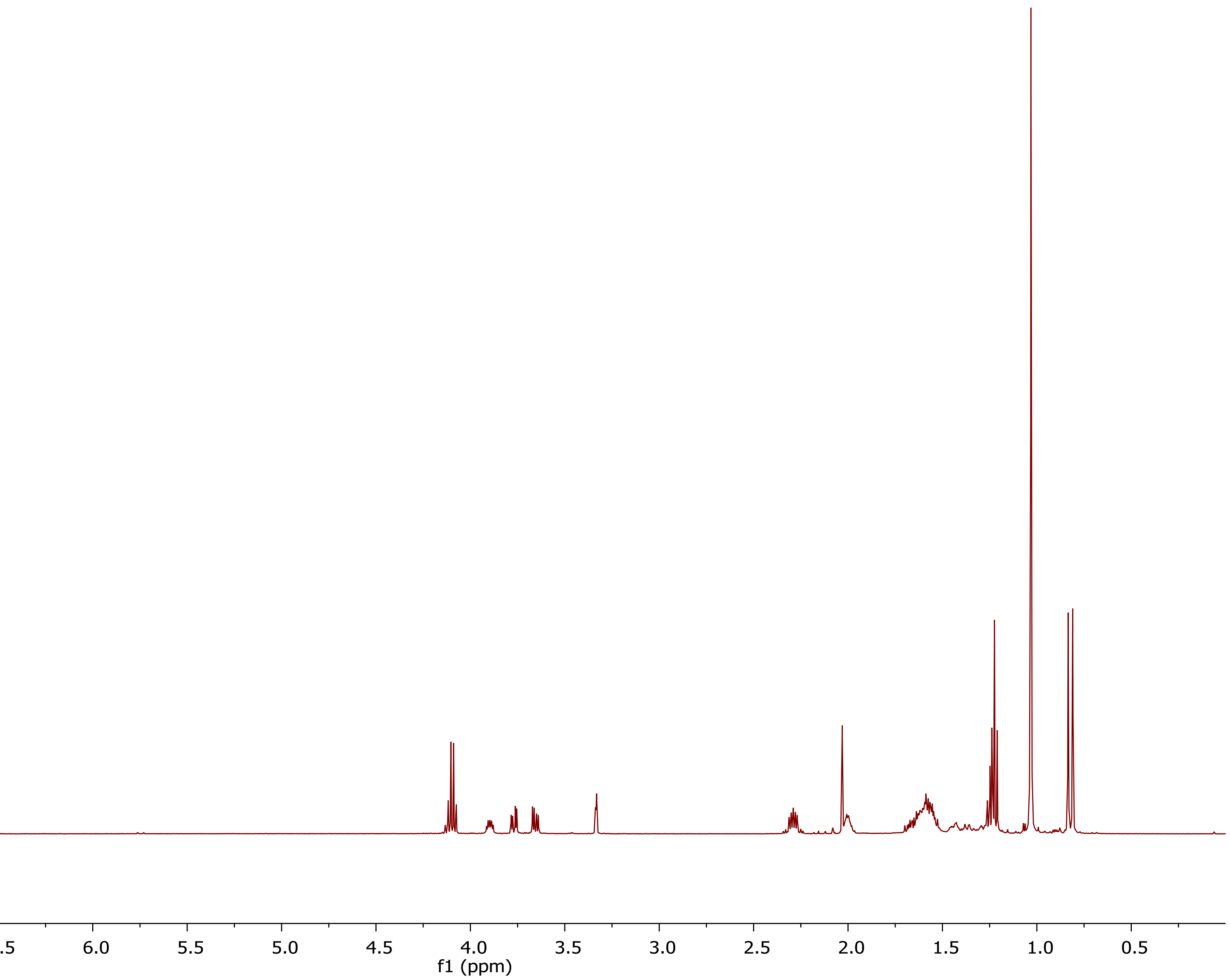

Ethyl 4-[(1R,3S,3aS,7aR)-3-{[(*tert*-butyldiphenylsilyl)oxy]methyl} octahydro-2-benzofuran-1-yl]-4-methylpentanoate **62**

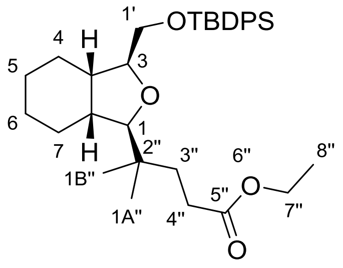

62

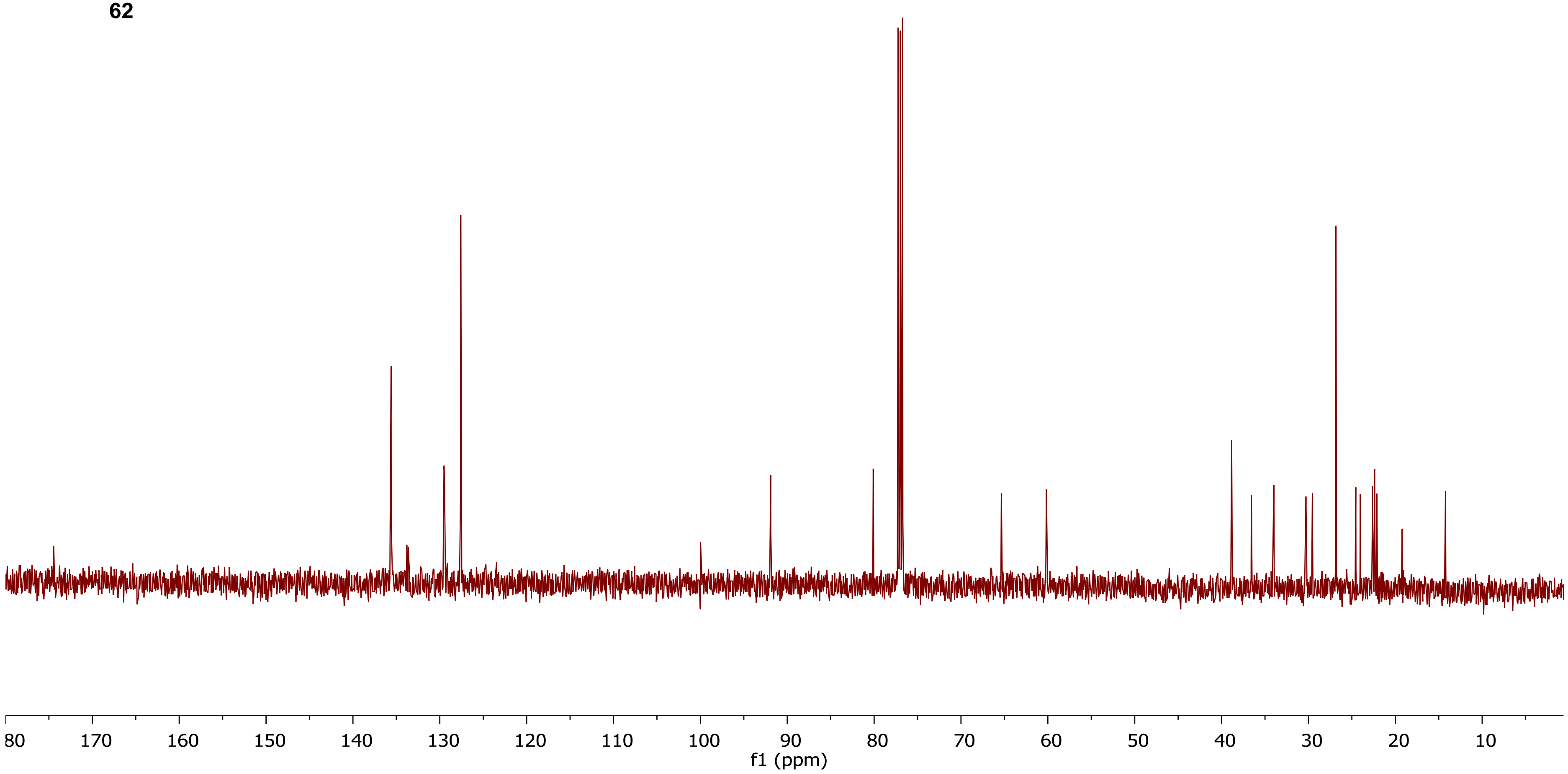

Ethyl 4-[(1R,3S)-3-(hydroxymethyl)octahydro-2-benzofuran-1-yl]-4-methylpentanoate **63**

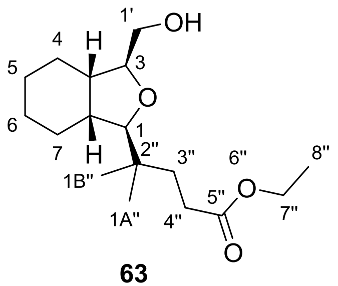

63

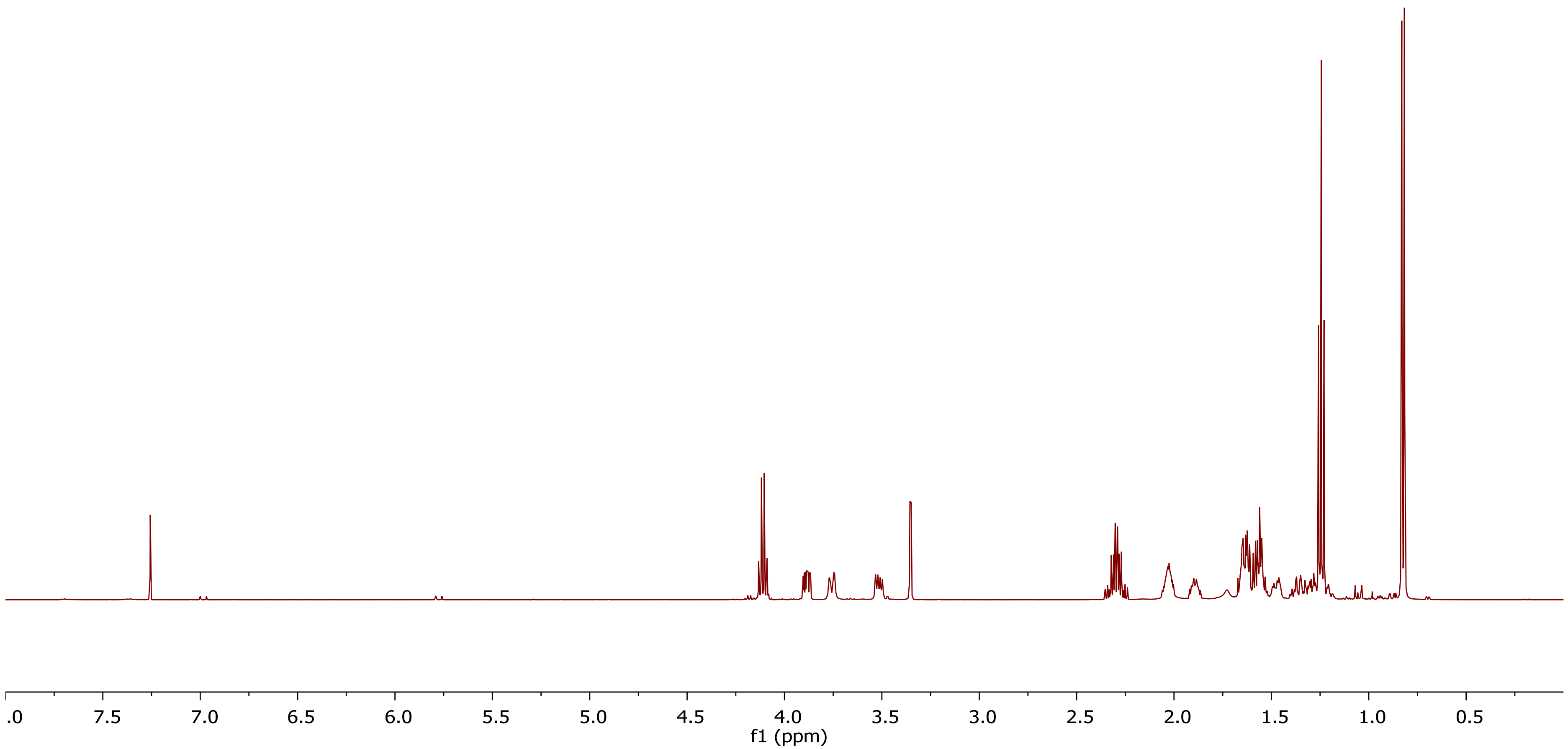

Ethyl 4-[(1R,3S)-3-(hydroxymethyl)octahydro-2-benzofuran-1-yl]-4-methylpentanoate **63**

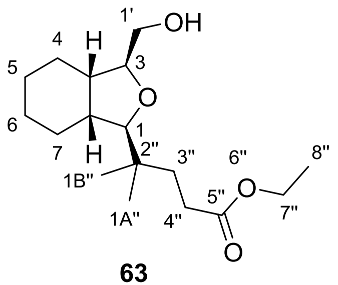

63

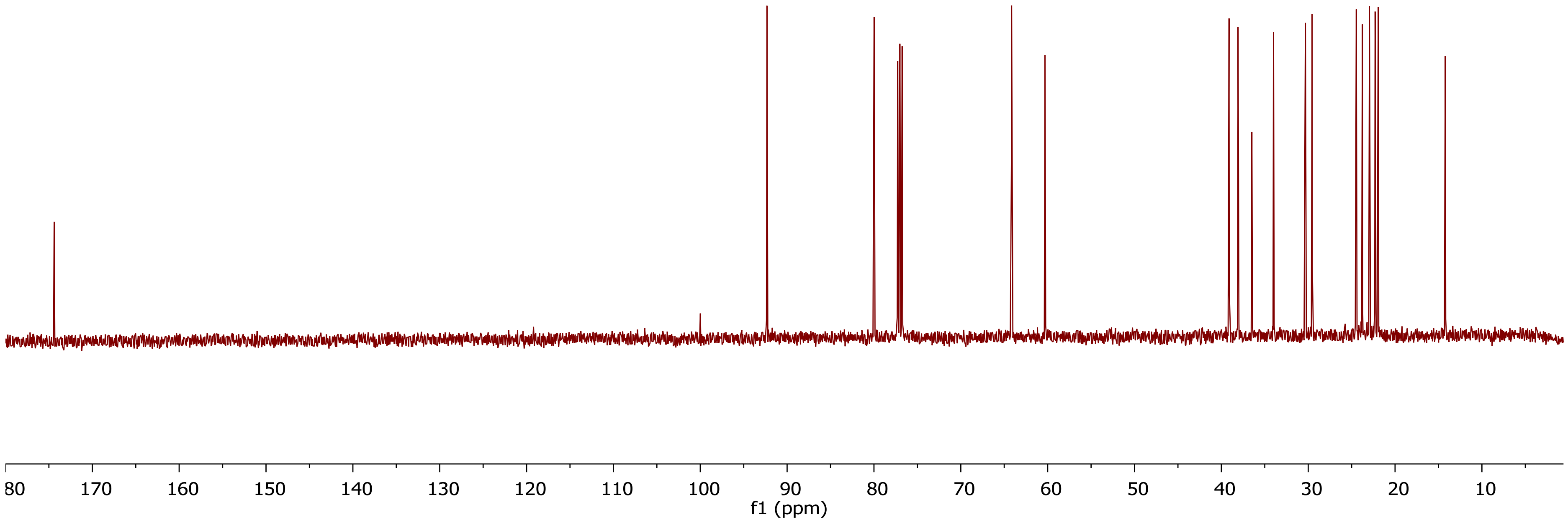

4-[(1R,3S,3aS,7aR)-3-(hydroxymethyl)-octahydro-2-benzofuran-1-yl]-4-methylpentanoic acid **64**

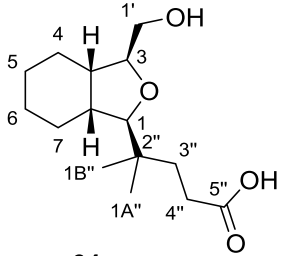

64

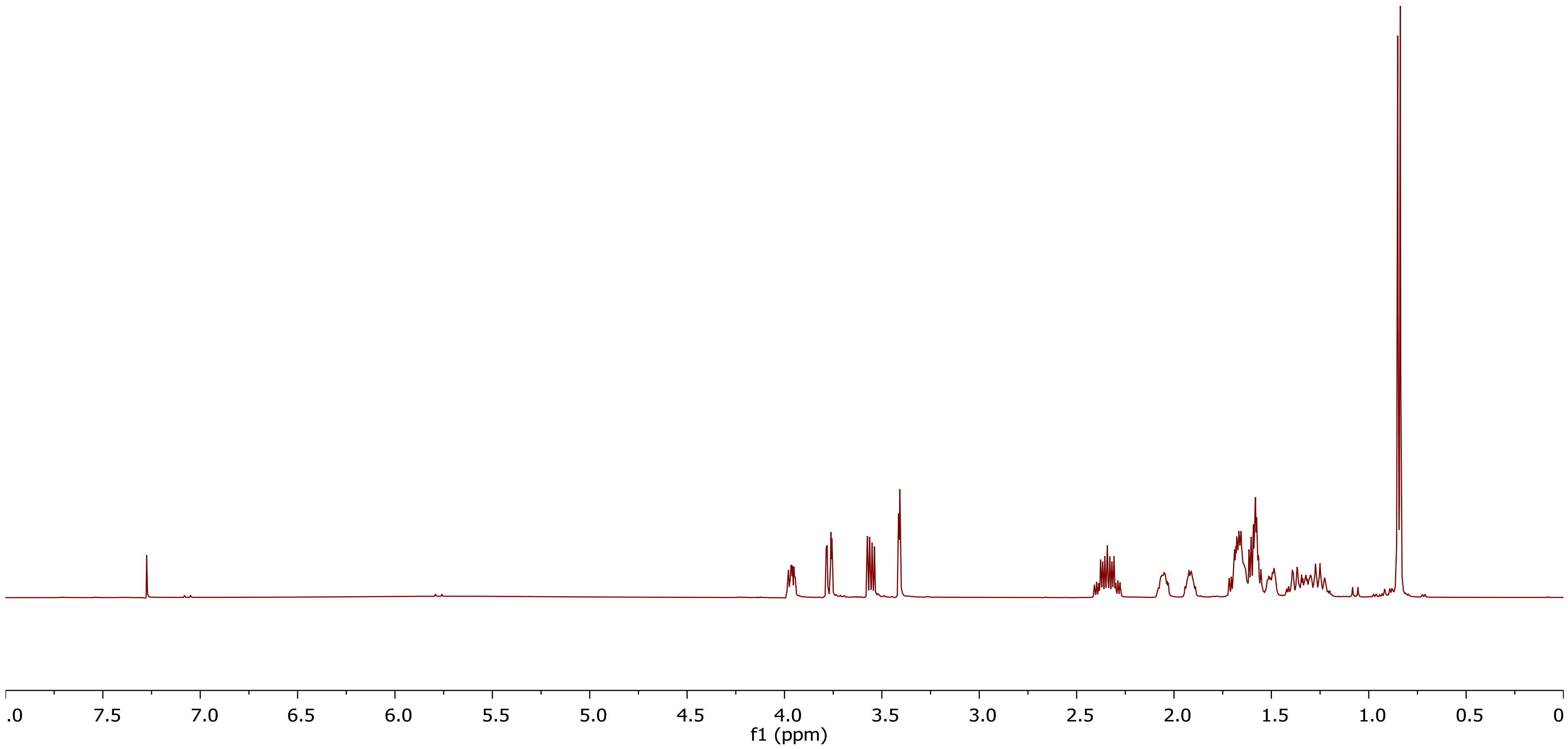

4-[(1R,3S,3aS,7aR)-3-(hydroxymethyl)-octahydro-2-benzofuran-1-yl]-4-methylpentanoic acid **64**

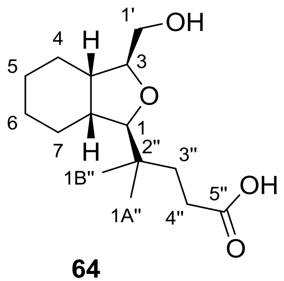

64

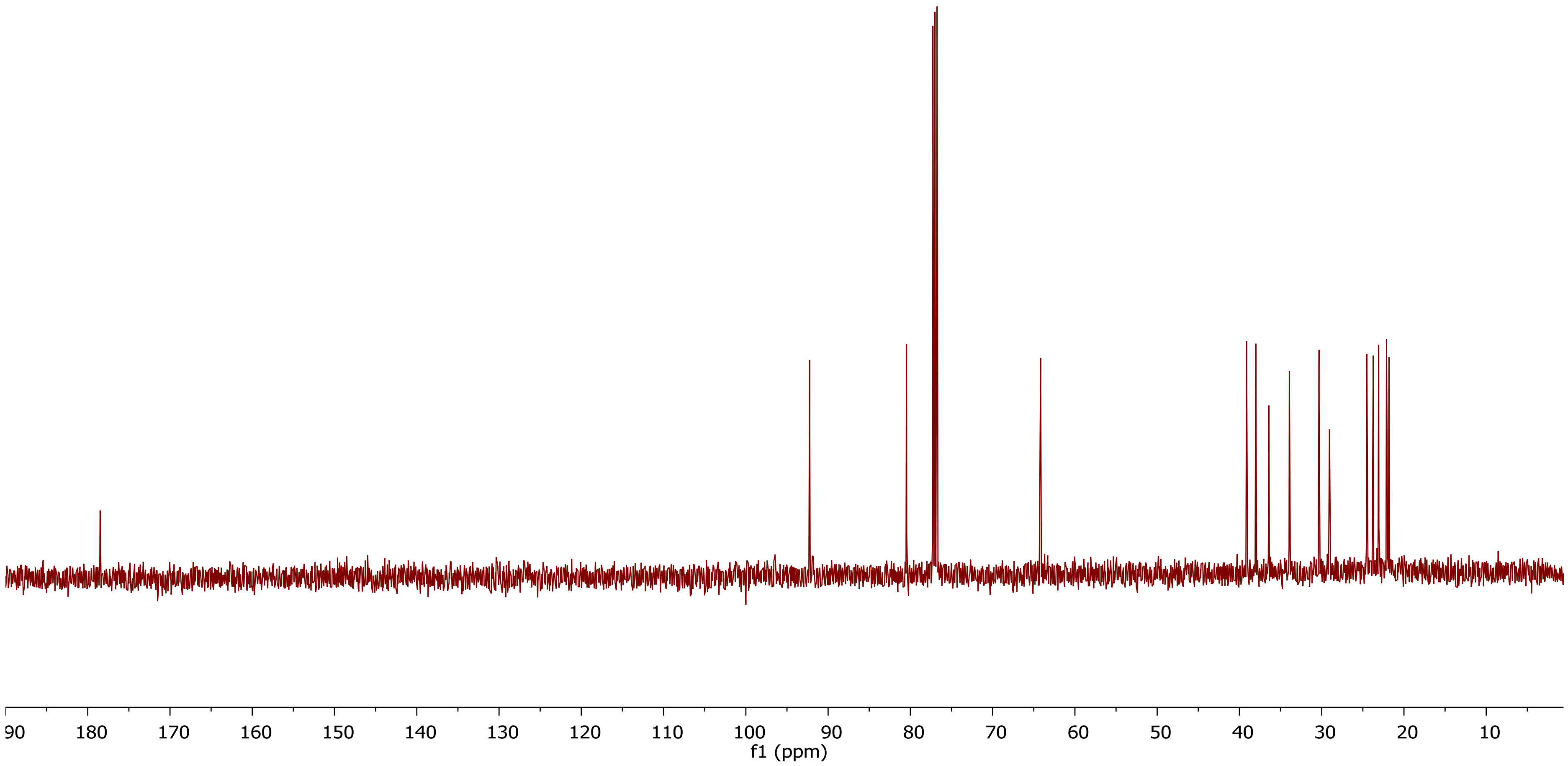

(1R,2R,7S,8S)-14,14-dimethyl-10,15-dioxatricyclo[6.6.1.0{2,7}]pentadecan-11-one **65**

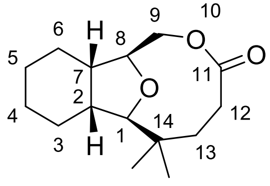

65

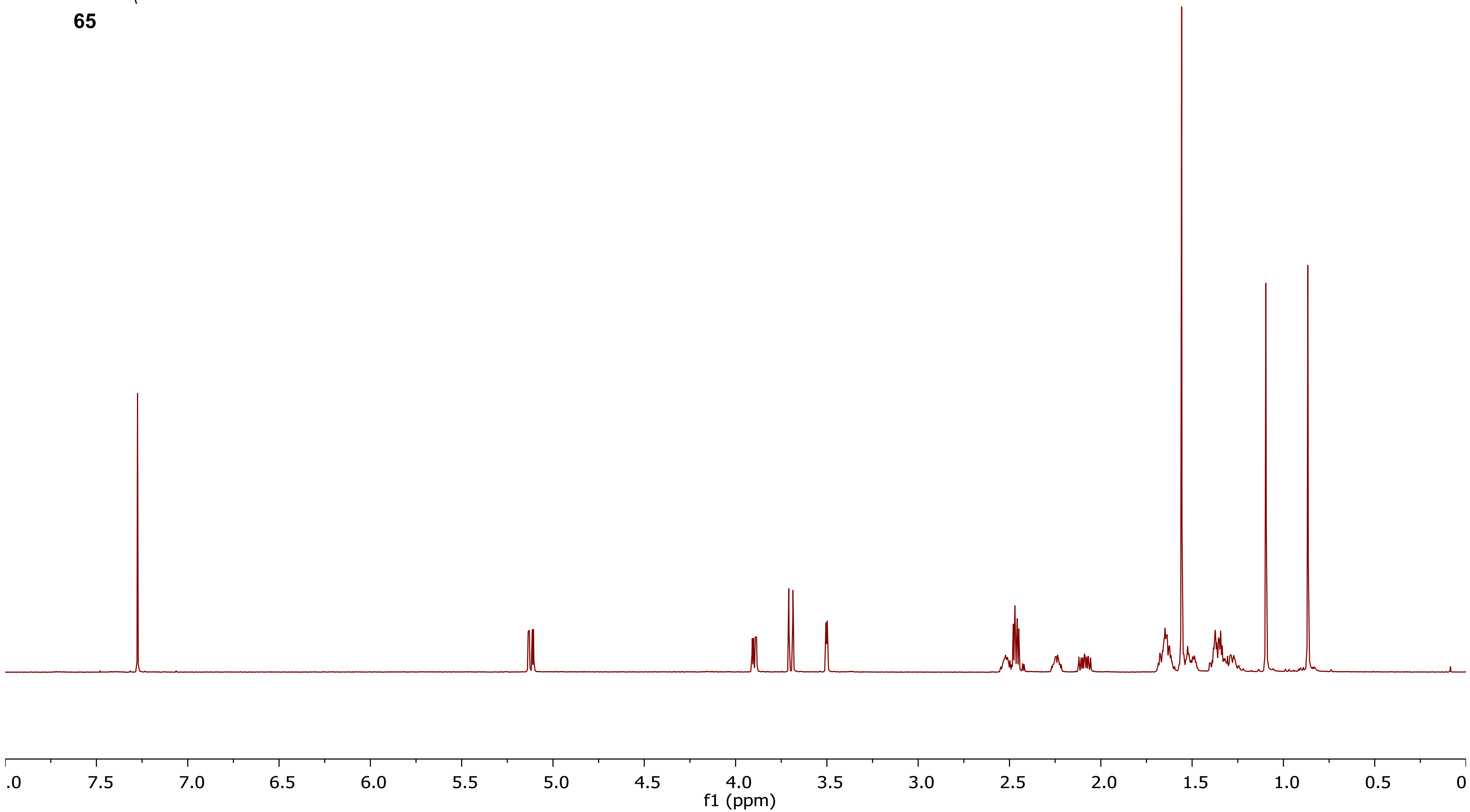

(1R,2R,7S,8S)-14,14-dimethyl-10,15-dioxatricyclo[6.6.1.0$^{\{2,7\}}$]pentadecan-11-one **65**

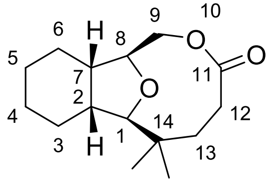

65

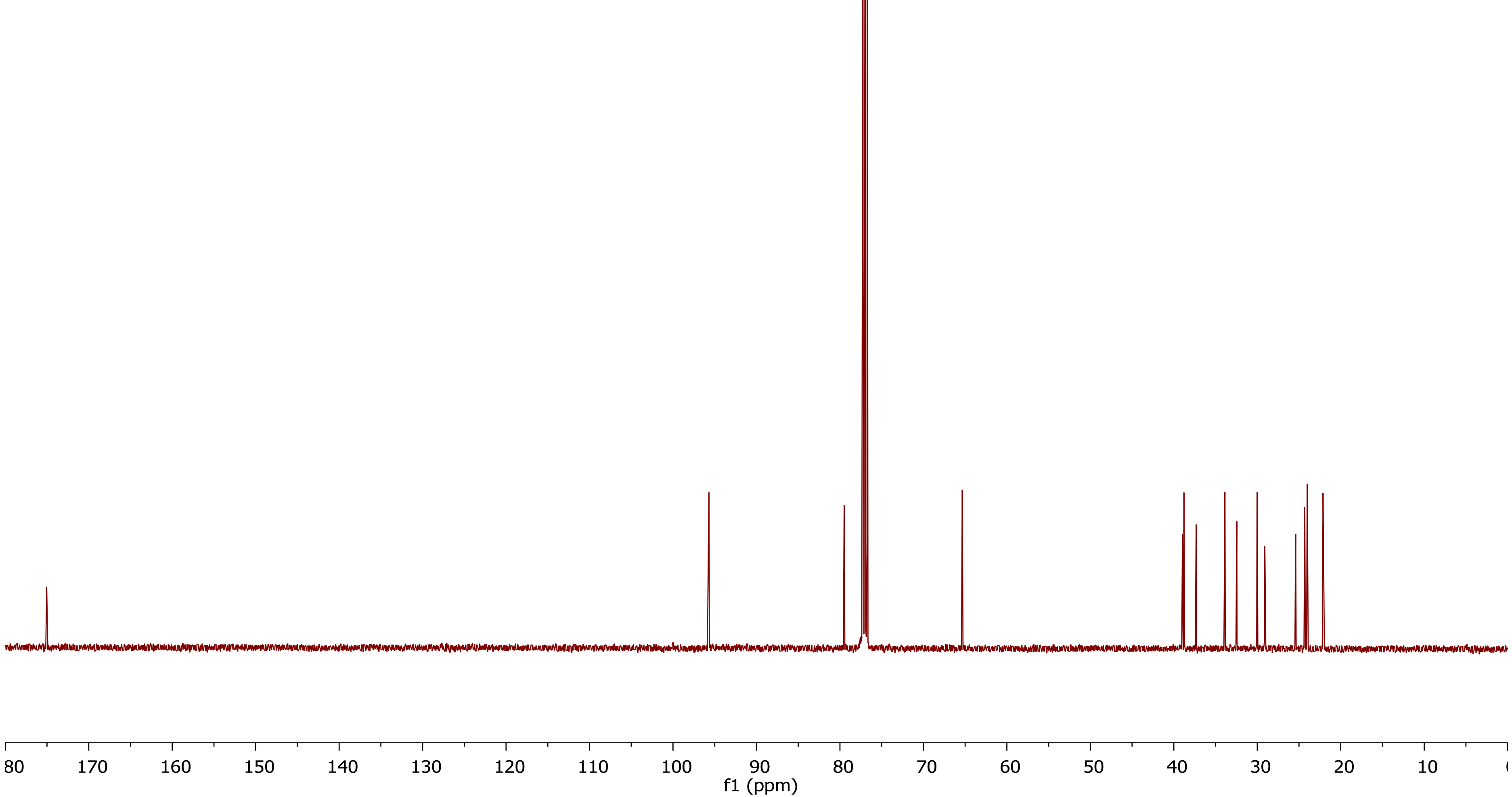

2-[(1R,3S,3aS,7aR)-3-{[(*tert*-*b*utyldiphenylsilyl)oxy]methyl}-octahydro-2-benzofuran-1-yl]-2-methylpropanal **66**

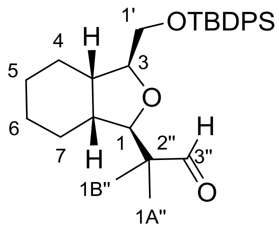

66

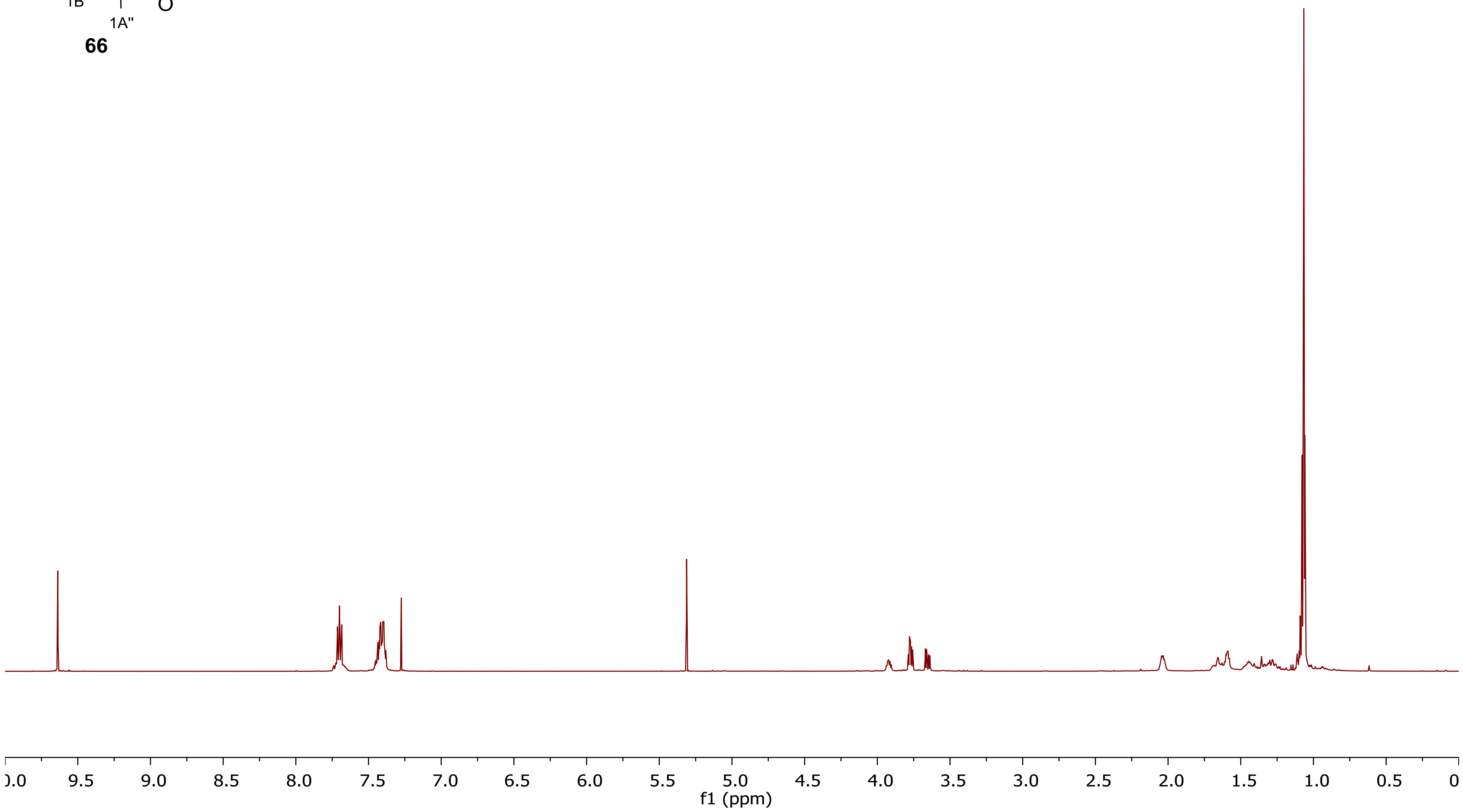

2-[(1R,3S,3aS,7aR)-3-{[(*tert*-*b*utyldiphenylsilyl)oxy]methyl}-octahydro-2-benzofuran-1-yl]-2-methylpropanal **66**

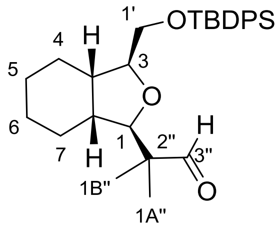

66

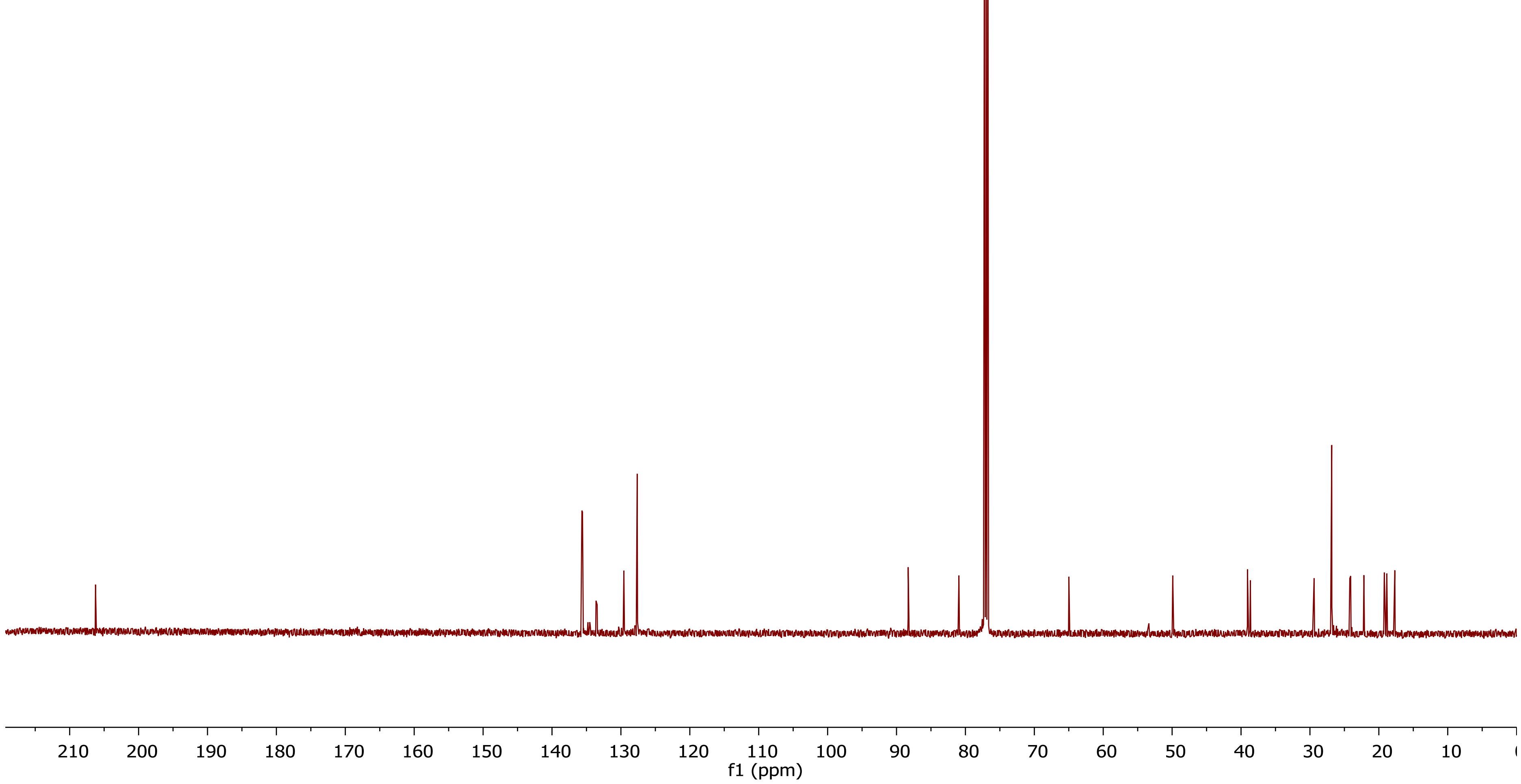

[(1S,3R,3aR,7aS)-3-(2-methyl-1-oxopropan-2-yl)octahydro-2-benzofuran-1-yl]methyl 2-bromoacetate **68**

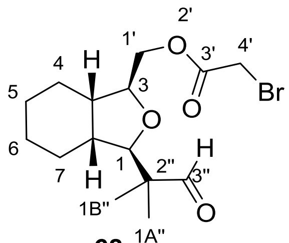

68

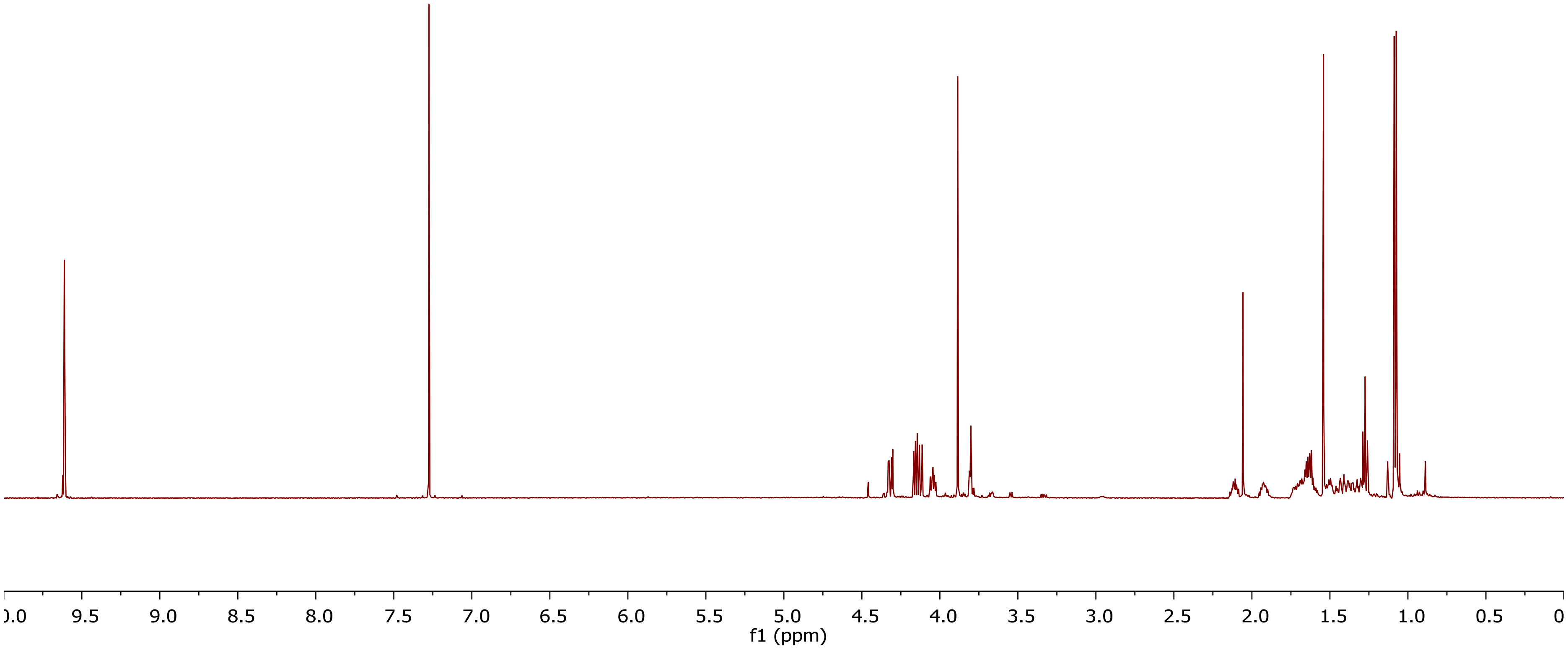

(1R,2R,7S,8S,13R)-13-hydroxy-14,14-dimethyl-10,15-dioxatricyclo[6.6.1.0$^{\{2,7\}}$]pentadecan-11-one **69**

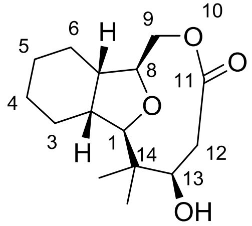

69

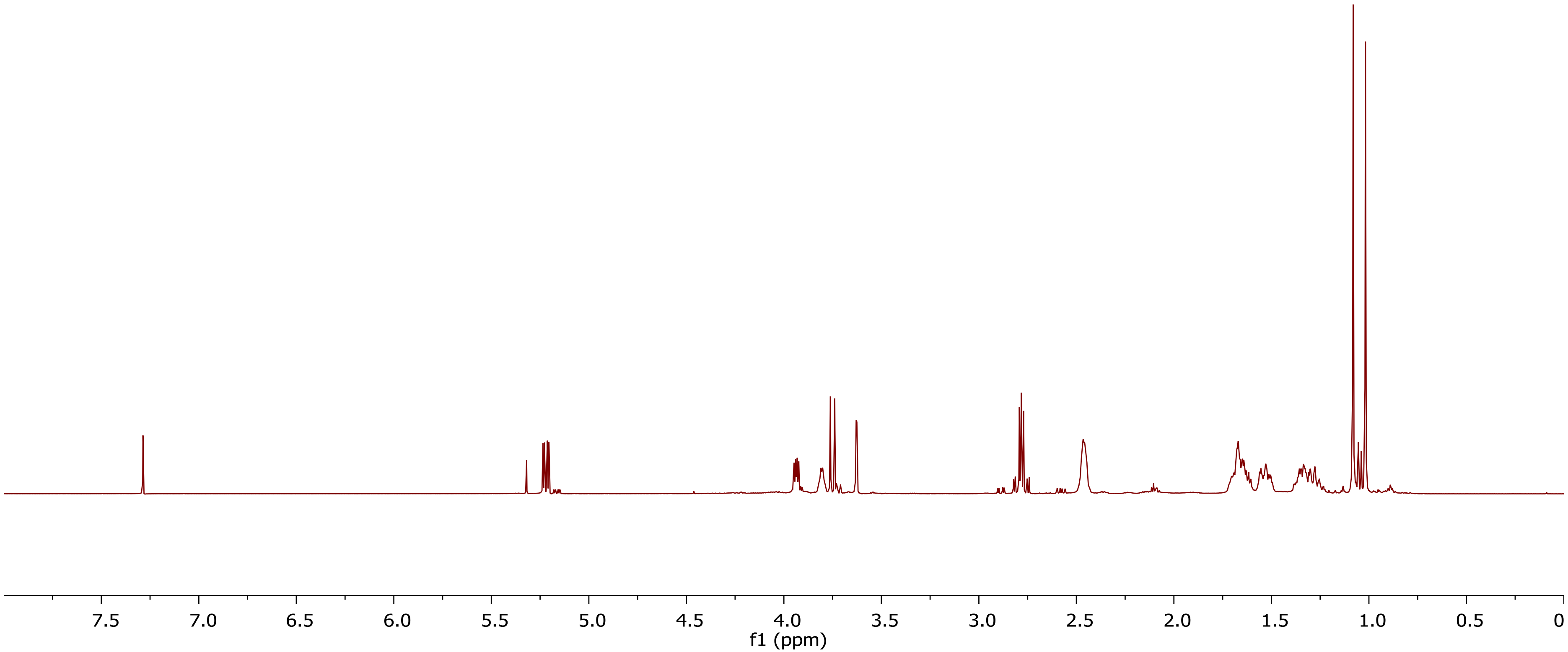

(1R,2R,7S,8S,13R)-13-hydroxy-14,14-dimethyl-10,15-dioxatricyclo[6.6.1.0$^{\{2,7\}}$]pentadecan-11-one **69**

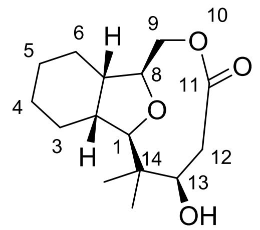

69

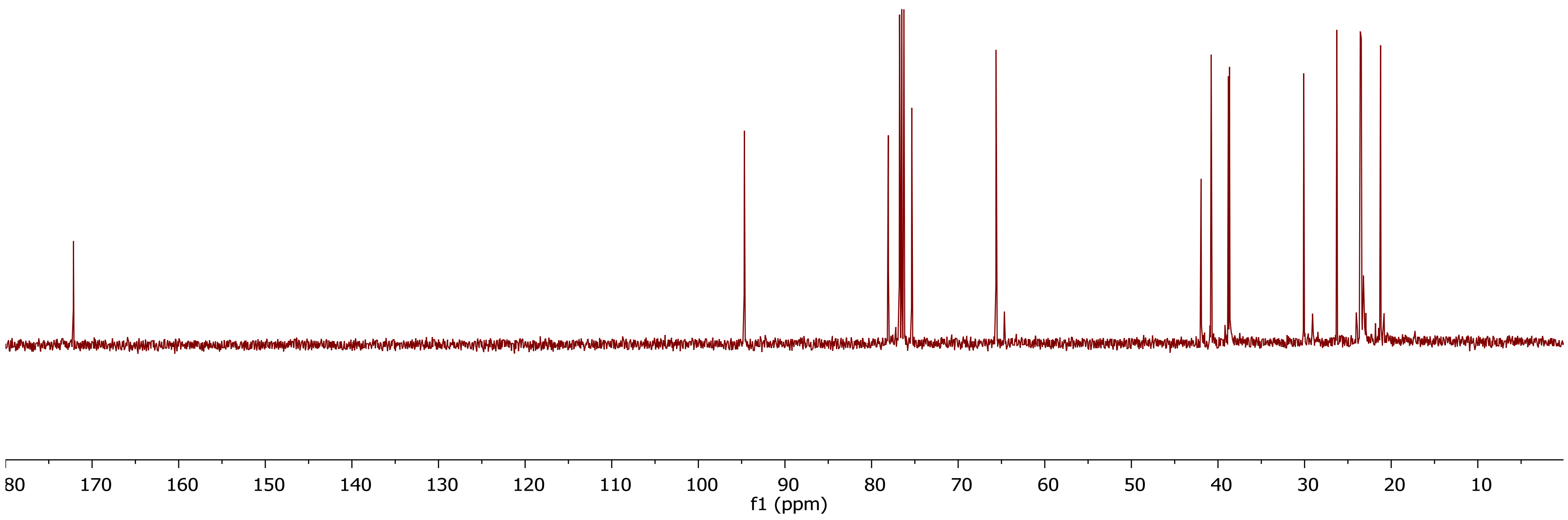

(1R,2R,7S,8S,13R)-14,14-dimethyl-11-oxo-10,15- dioxatricyclo[6.6.1.0{2,7}]pentadecan-13-yl acetate **70**

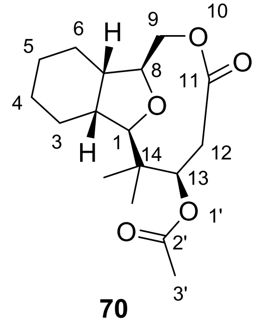

70

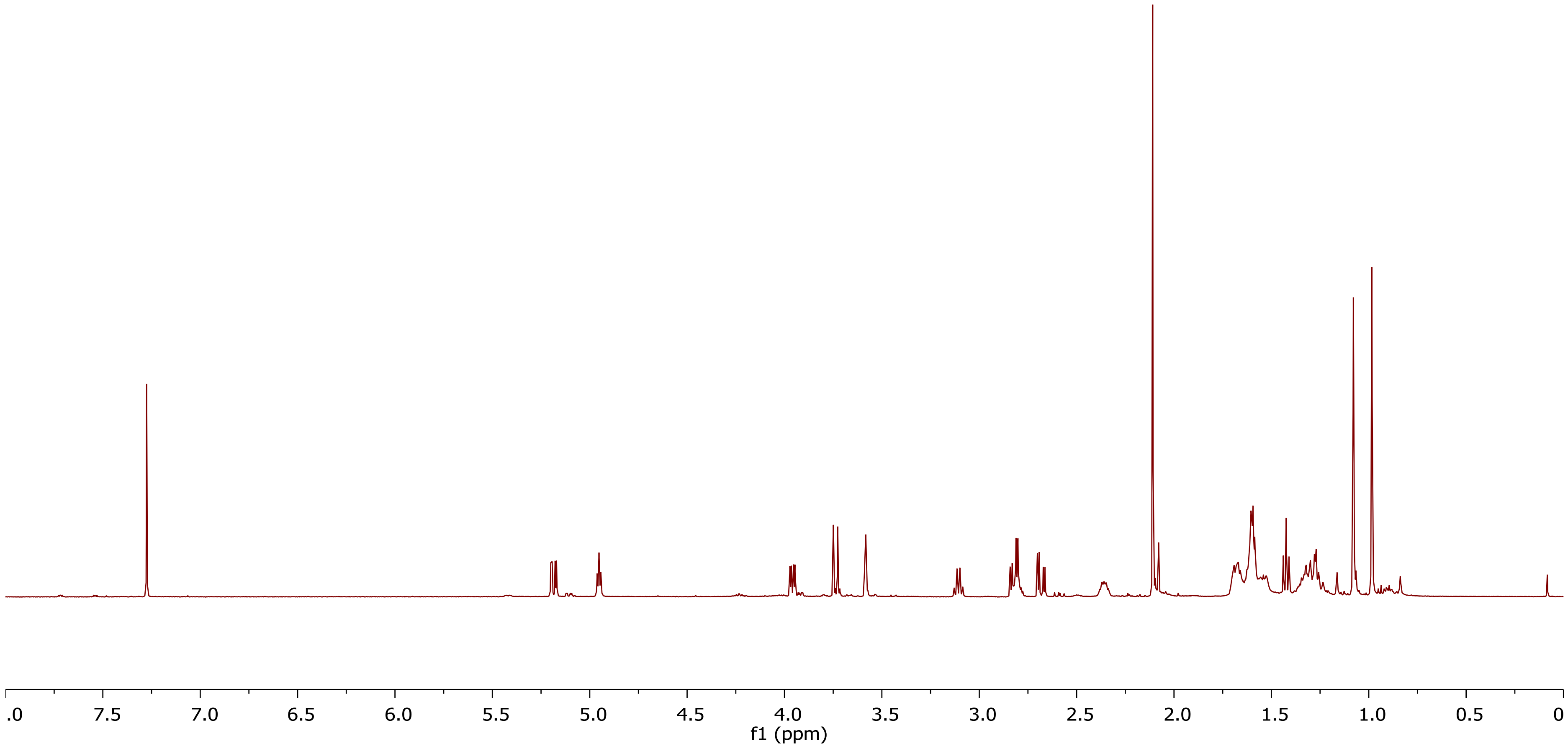

(1R,2R,7S,8S,13R)-14,14-dimethyl-11-oxo-10,15- dioxatricyclo[6.6.1.0{2,7}]pentadecan-13-yl acetate **70**

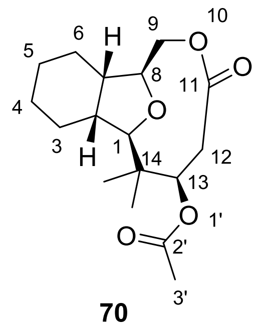

70

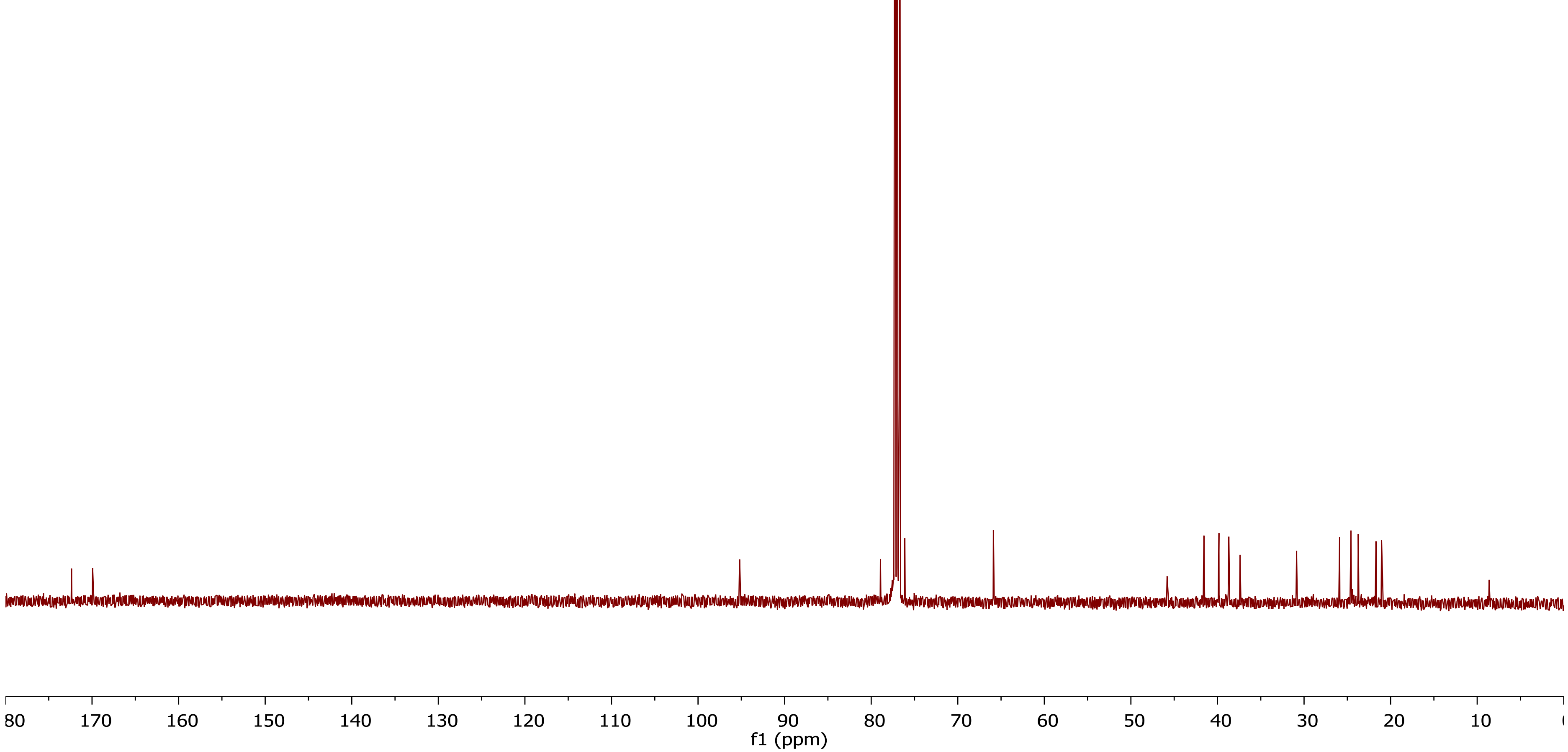

(1S,3S,3aR,7aS)-3-(Prop-2-en-1-yl)-octahydro-2-benzofuran-1-carbaldehyde **71**

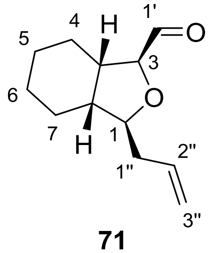

71

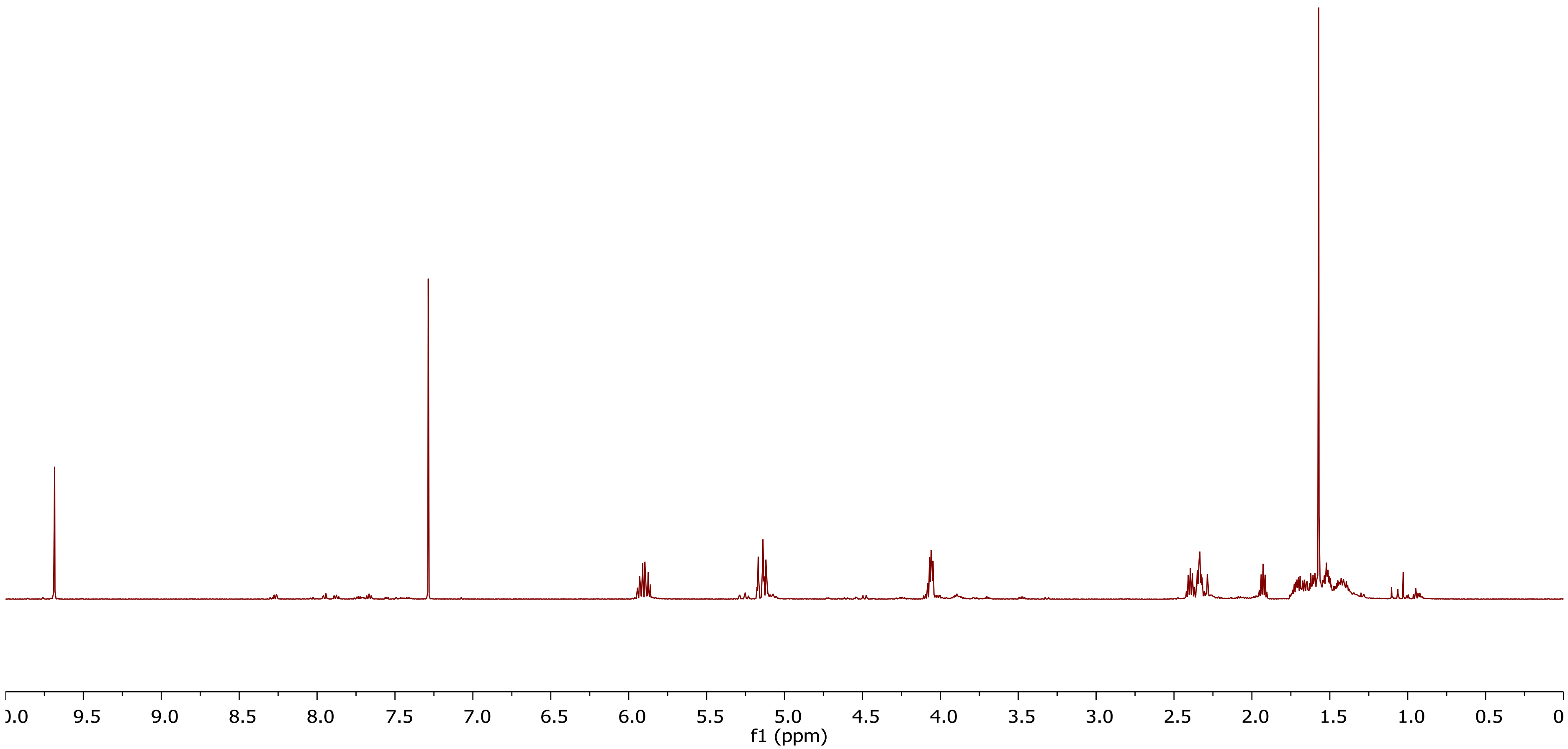

(1S,3S,3aR,7aS)-3-(Prop-2-en-1-yl)-octahydro-2-benzofuran-1-carbaldehyde **71**

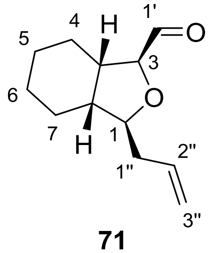

71

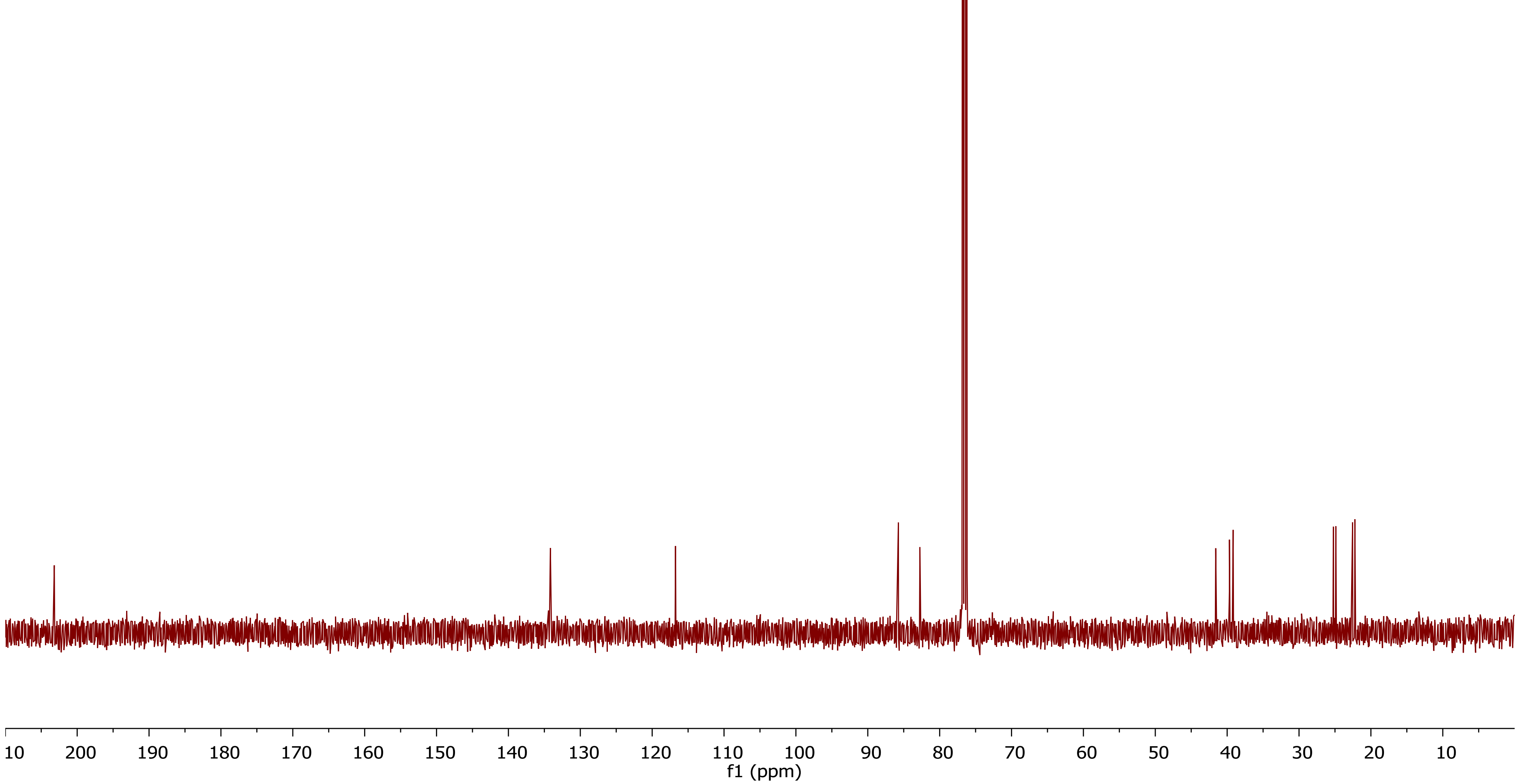

(R)-1-((1S,3S,3aR,7aS)-3-allyl-1-methyloctahydroisobenzofuran-1-yl)pent-4-en-1-ol **72**

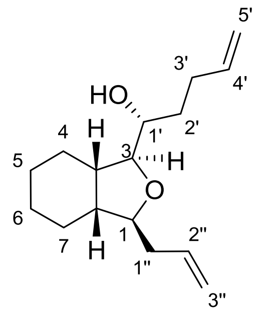

72

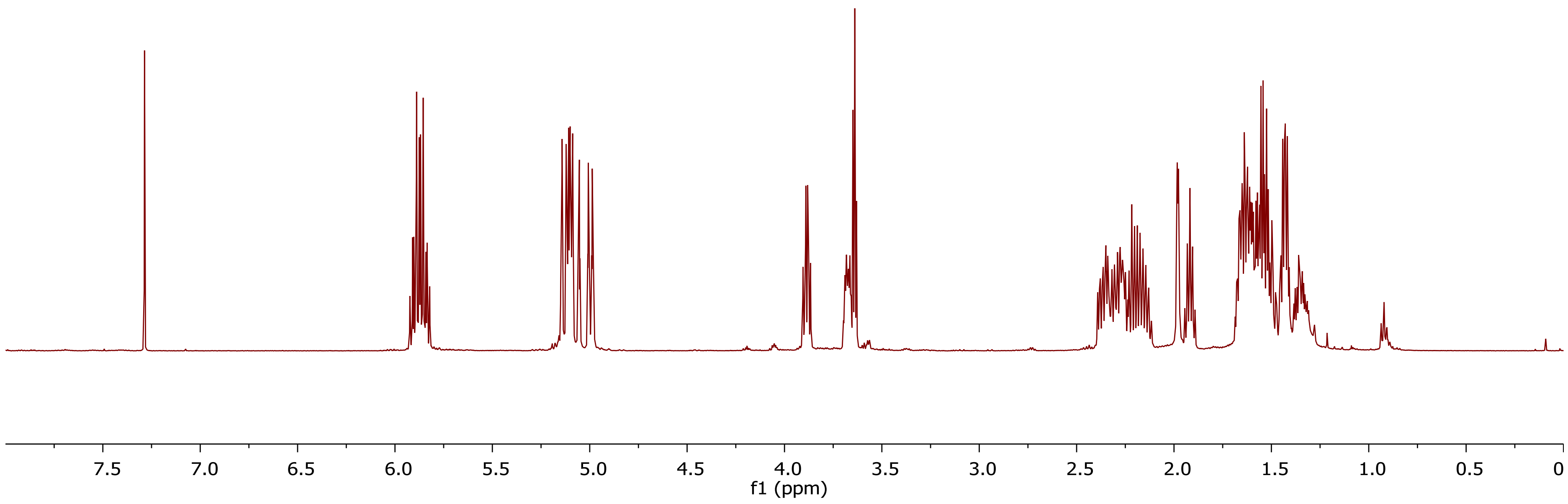

(R)-1-((1S,3S,3aR,7aS)-3-allyl-1-methyloctahydroisobenzofuran-1-yl)pent-4-en-1-ol **72**

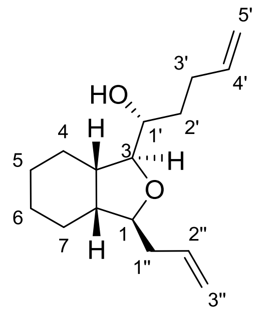

72

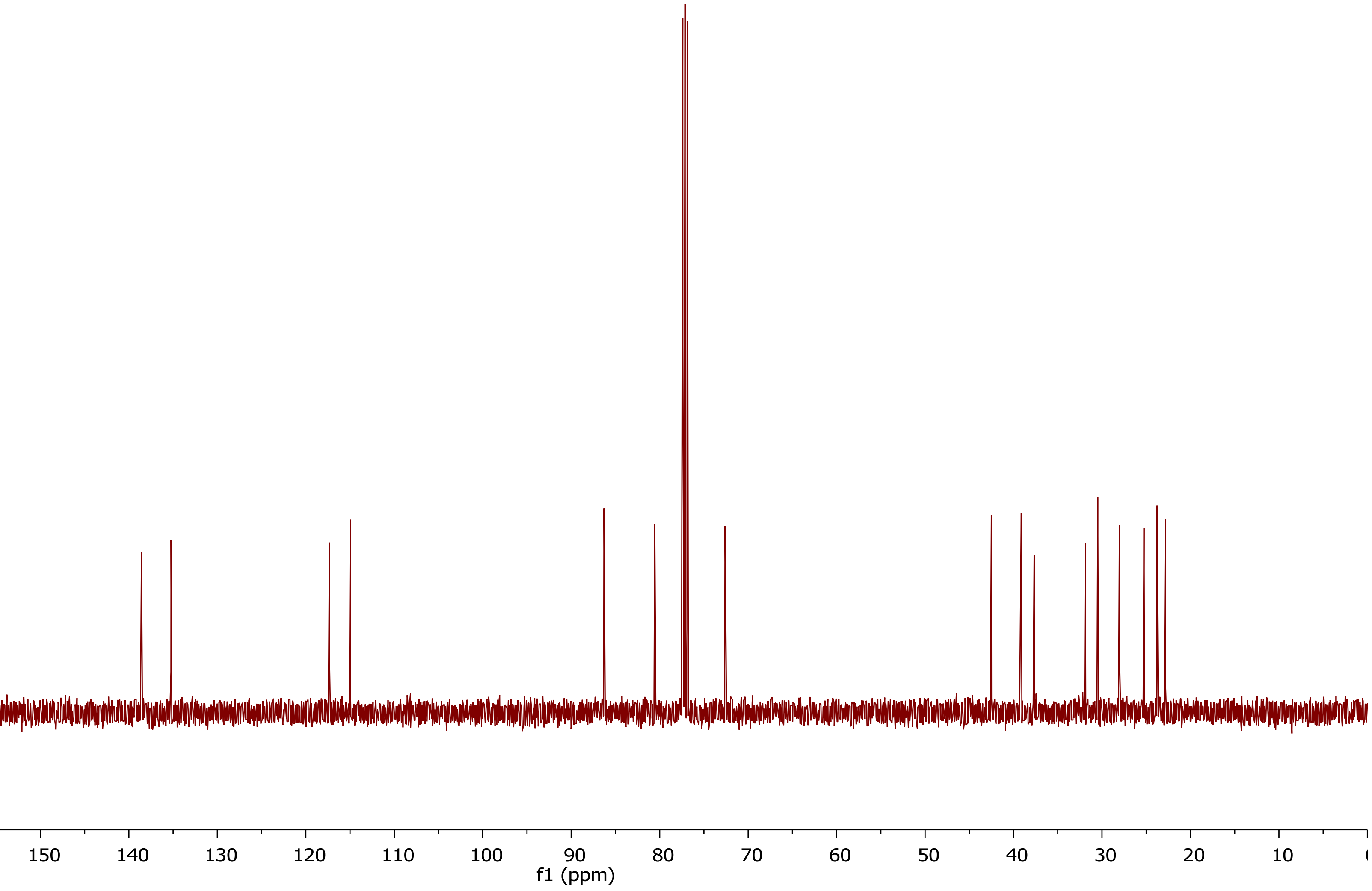

(S)-1-((1S,3S,3aR,7aS)-3-allyl-1-methyloctahydroisobenzofuran-1-yl)pent-4-en-1-ol **73**

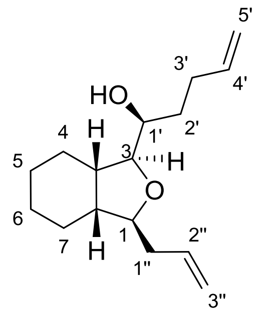

73

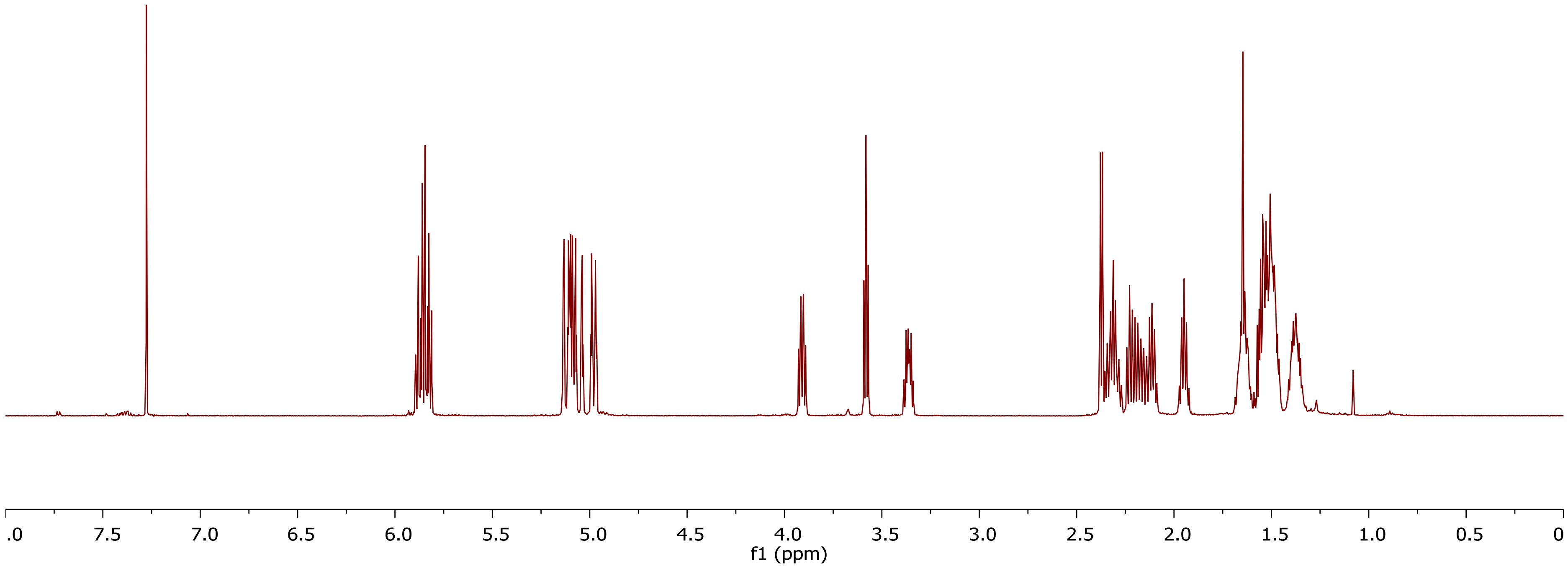

(S)-1-((1S,3S,3aR,7aS)-3-allyl-1-methyloctahydroisobenzofuran-1-yl)pent-4-en-1-ol **73**

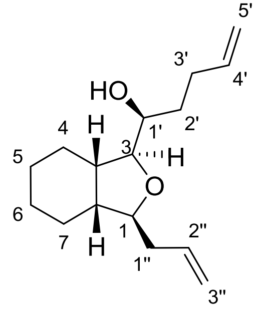

73

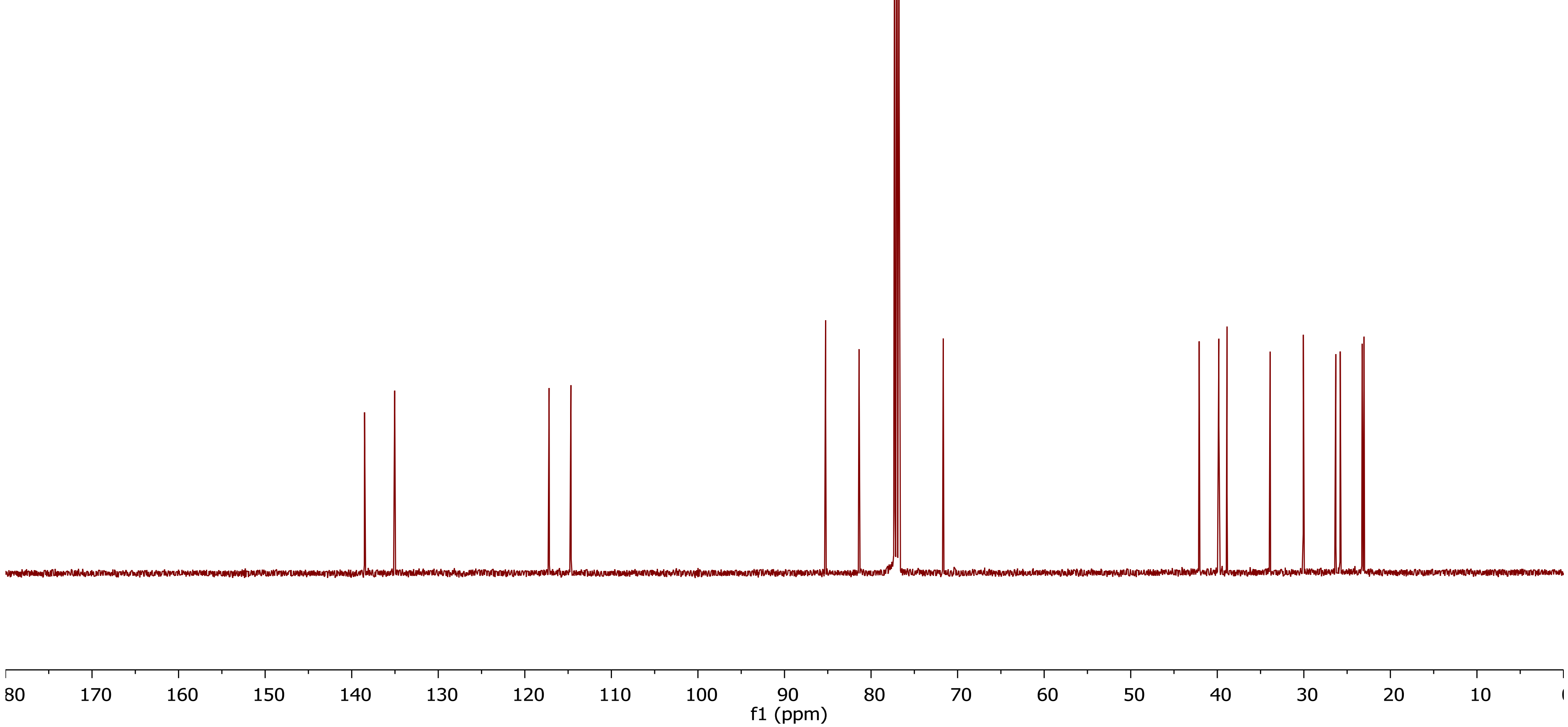

(((R)-1-((1S,3S,3aR,7aS)-3-allyl-1-methyloctahydroisobenzofuran-1-yl)pent-4-en-1-yl)oxy)(tert-butyl)dimethylsilane **74**

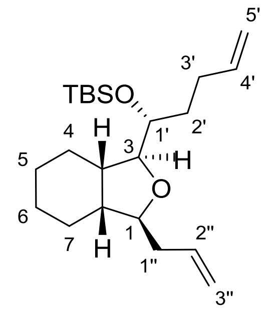

74

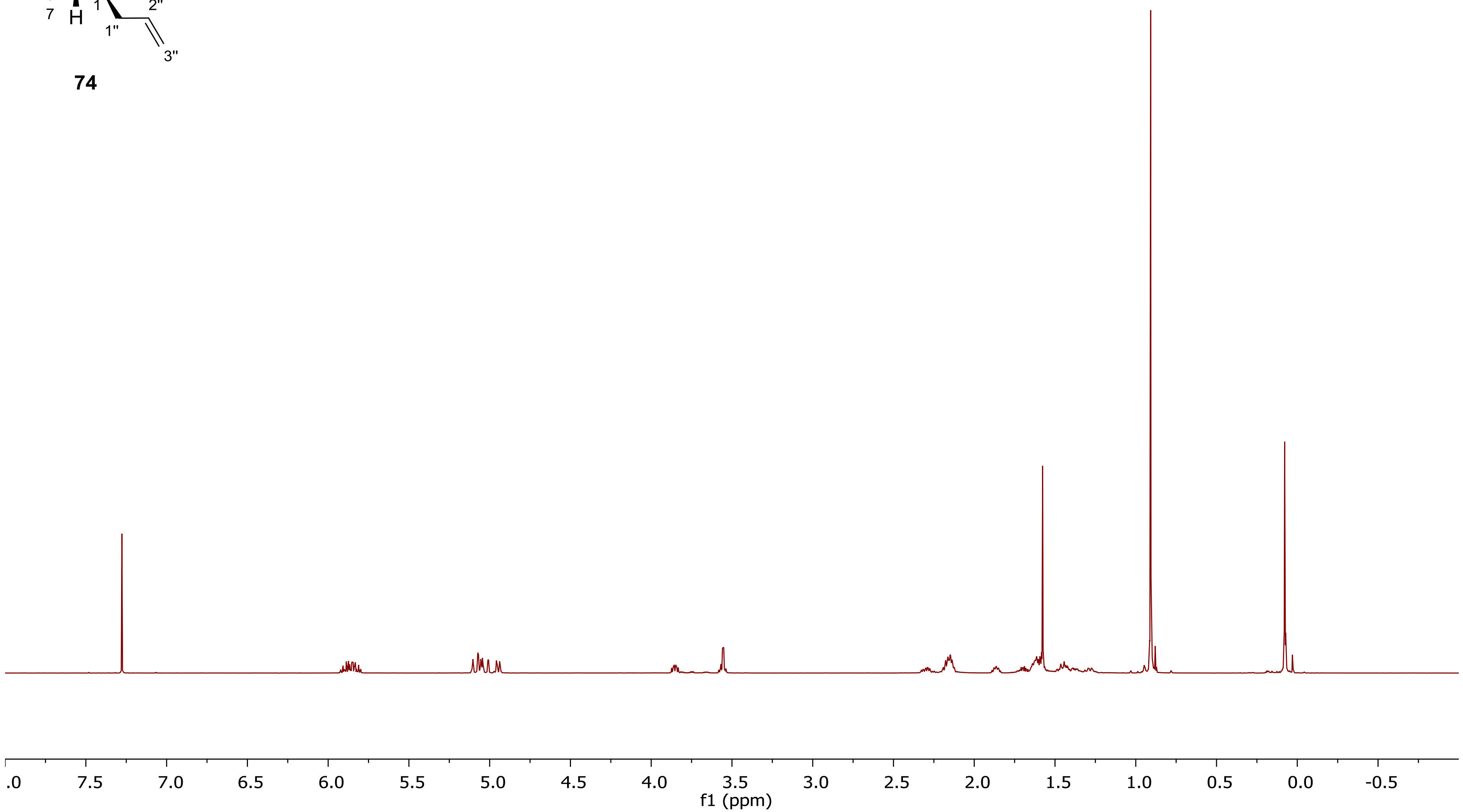

(((R)-1-((1S,3S,3aR,7aS)-3-allyl-1-methyloctahydroisobenzofuran-1-yl)pent-4-en-1-yl)oxy)(tert-butyl)dimethylsilane **74**

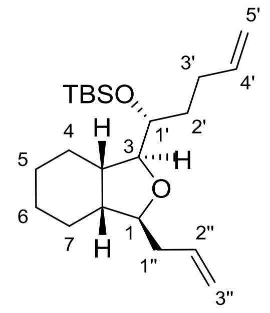

74

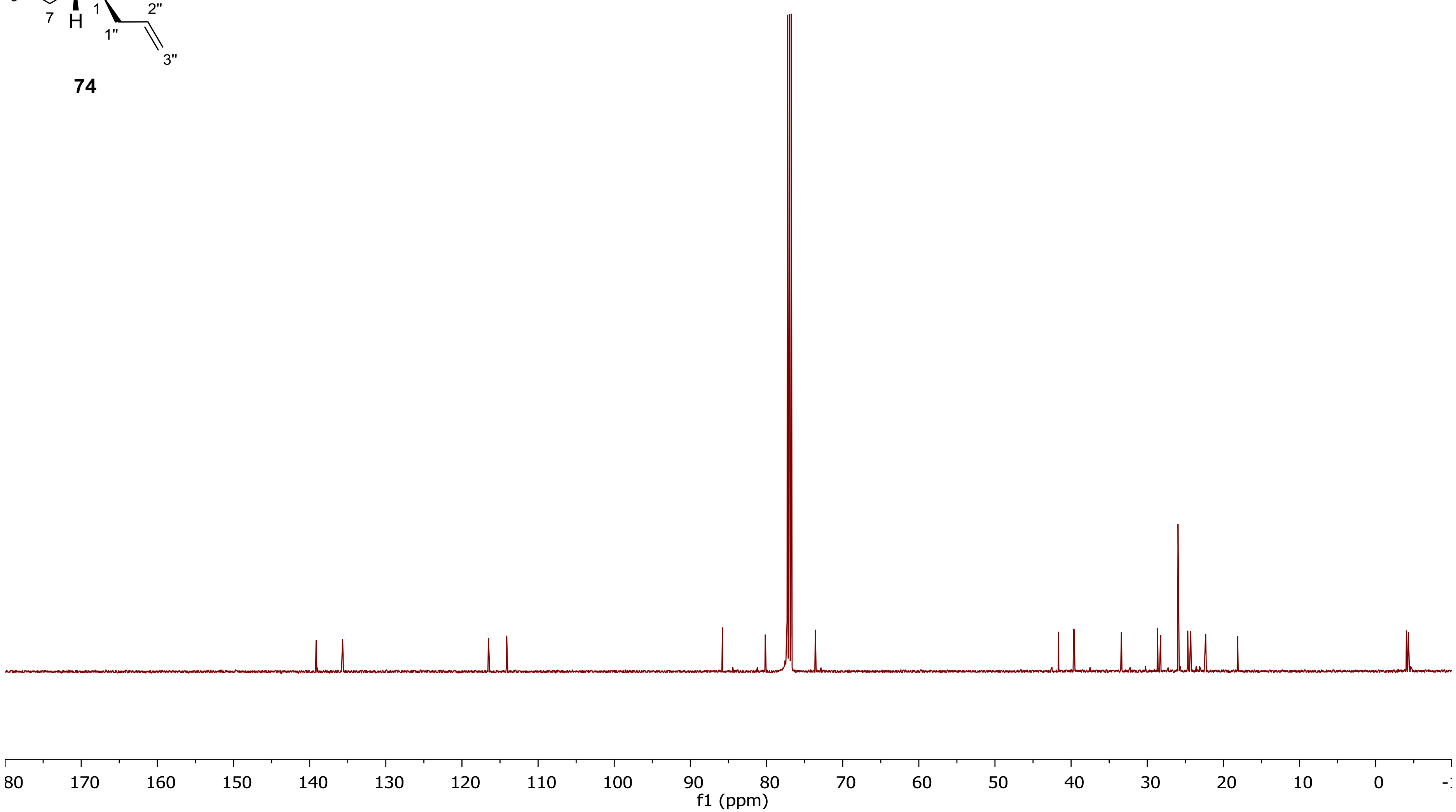

tert-butyl(((4aS,5S,6R,12S,12aR,Z)-1,2,3,4,4a,5,6,7,8,11,12,12a-dodecahydro-5,12-epoxybenzo[10]annulen-6-yl)oxy)dimethylsilane **75**

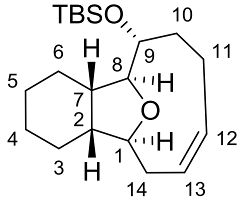

75

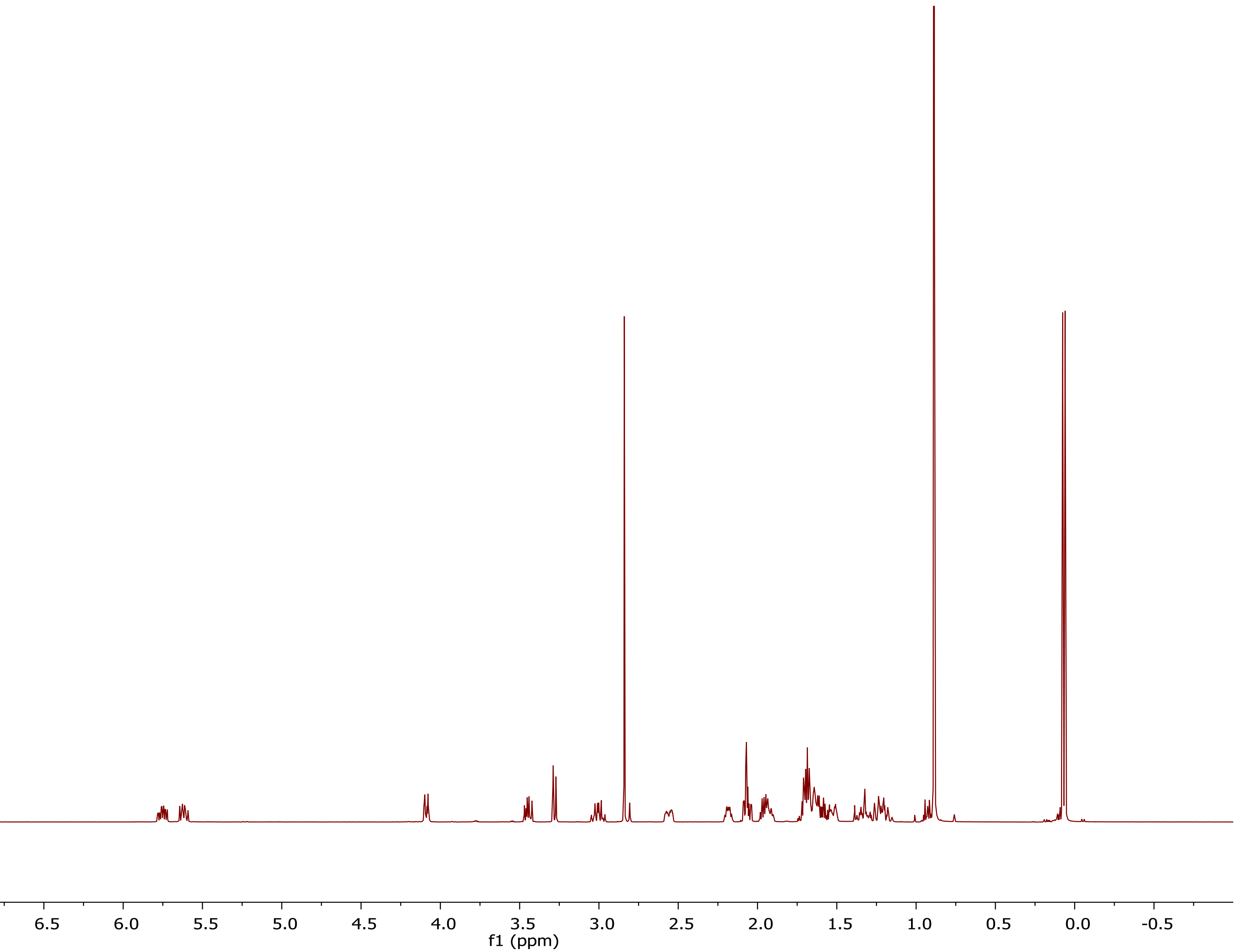

tert-butyl(((4aS,5S,6R,12S,12aR,Z)-1,2,3,4,4a,5,6,7,8,11,12,12a-dodecahydro-5,12-epoxybenzo[10]annulen-6-yl)oxy)dimethylsilane **75**

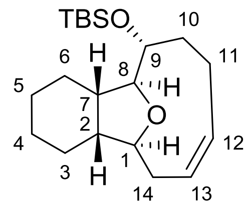

75

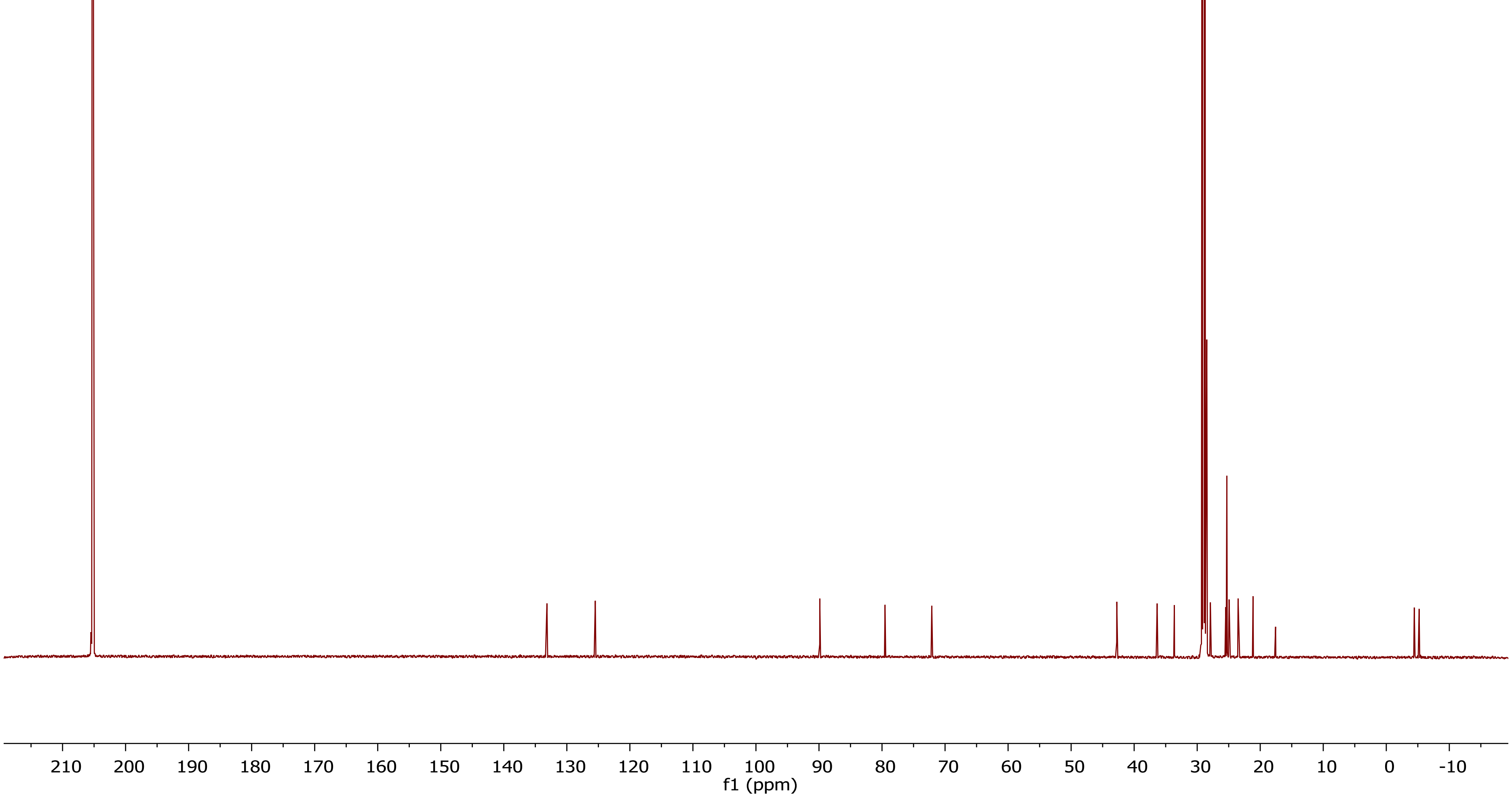

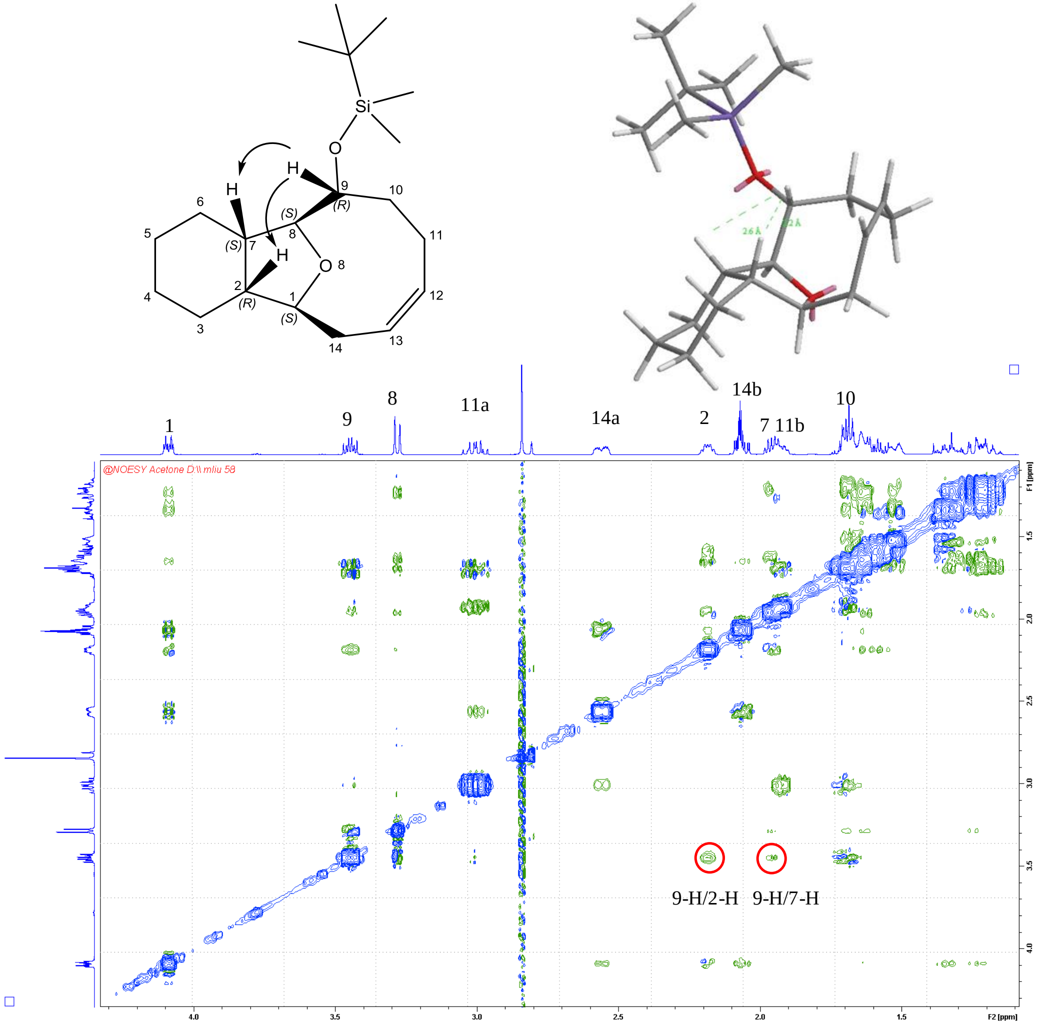

The ^1H-^1H NOESY spectrum for **75**

(4aS,5S,6R,12S,12aR,Z)-5,12-dimethyl-1,2,3,4,4a,5,6,7,8,11,12,12a-dodecahydro-5,12-epoxybenzo[10]annulen-6-ol **76**

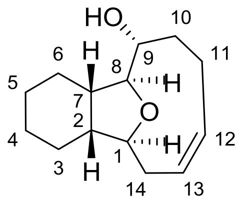

76

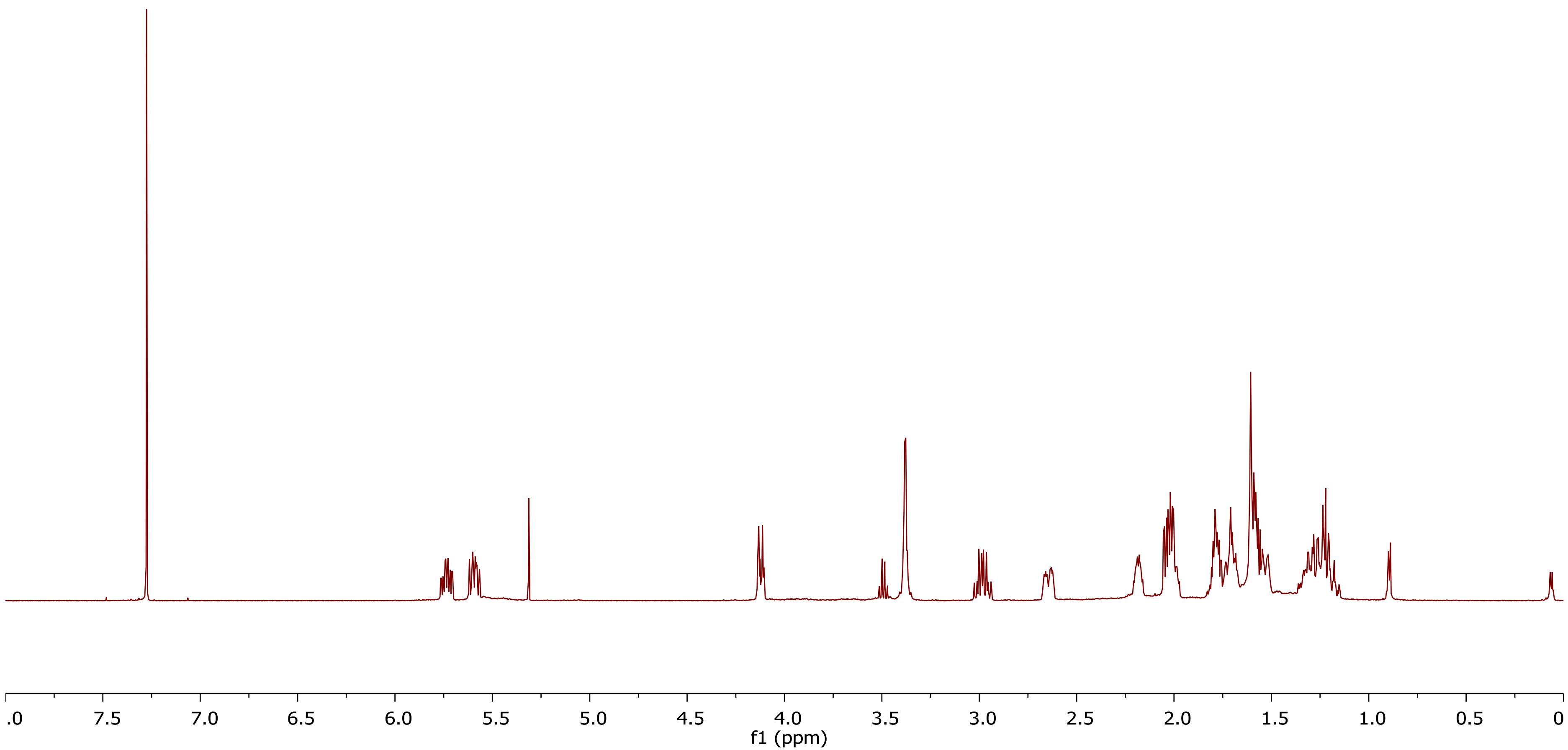

(4aS,5S,6R,12S,12aR,Z)-5,12-dimethyl-1,2,3,4,4a,5,6,7,8,11,12,12a-dodecahydro-5,12-epoxybenzo[10]annulen-6-ol **76**

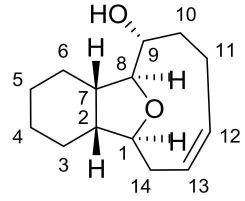

76

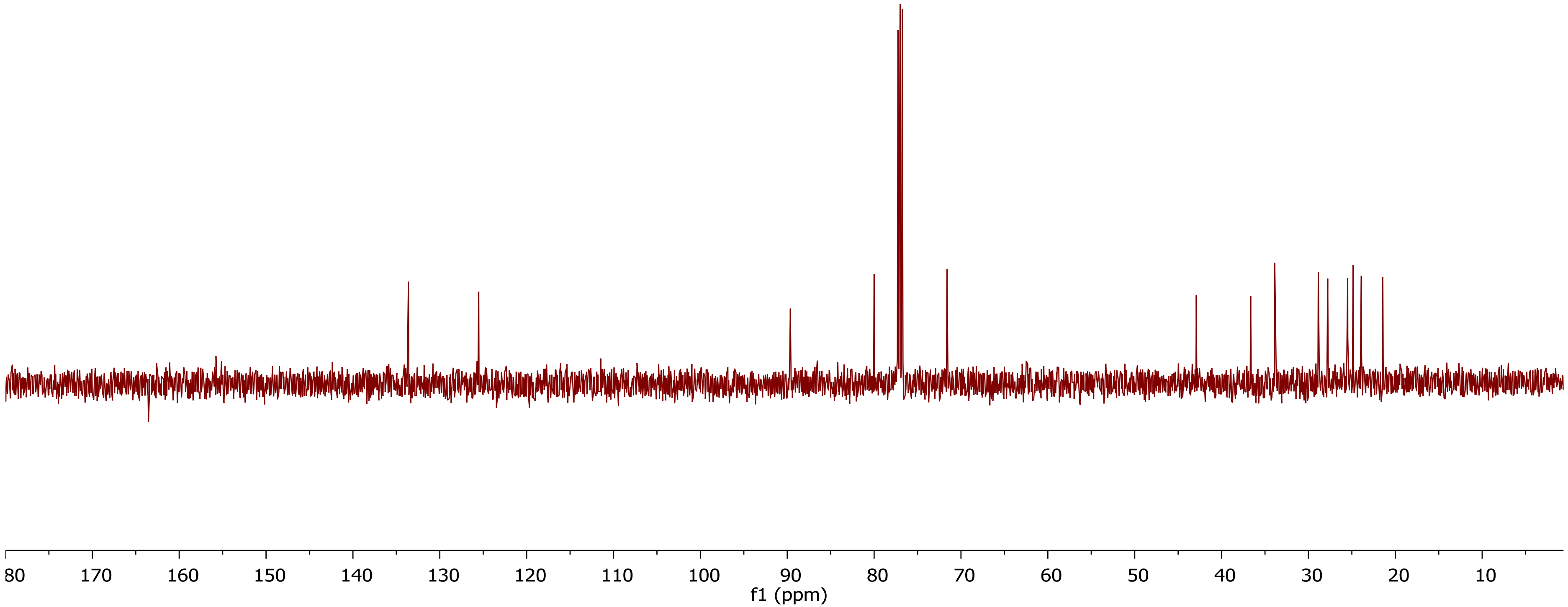

(3S,3aS,4R,7S,7aR)-3-(((tert-butyldiphenylsilyl)oxy)methyl)-1,3,3a,4,7,7a-hexahydro-4,7-methanoisobenzofuran-1-ol **79a**

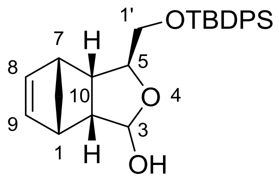

79a

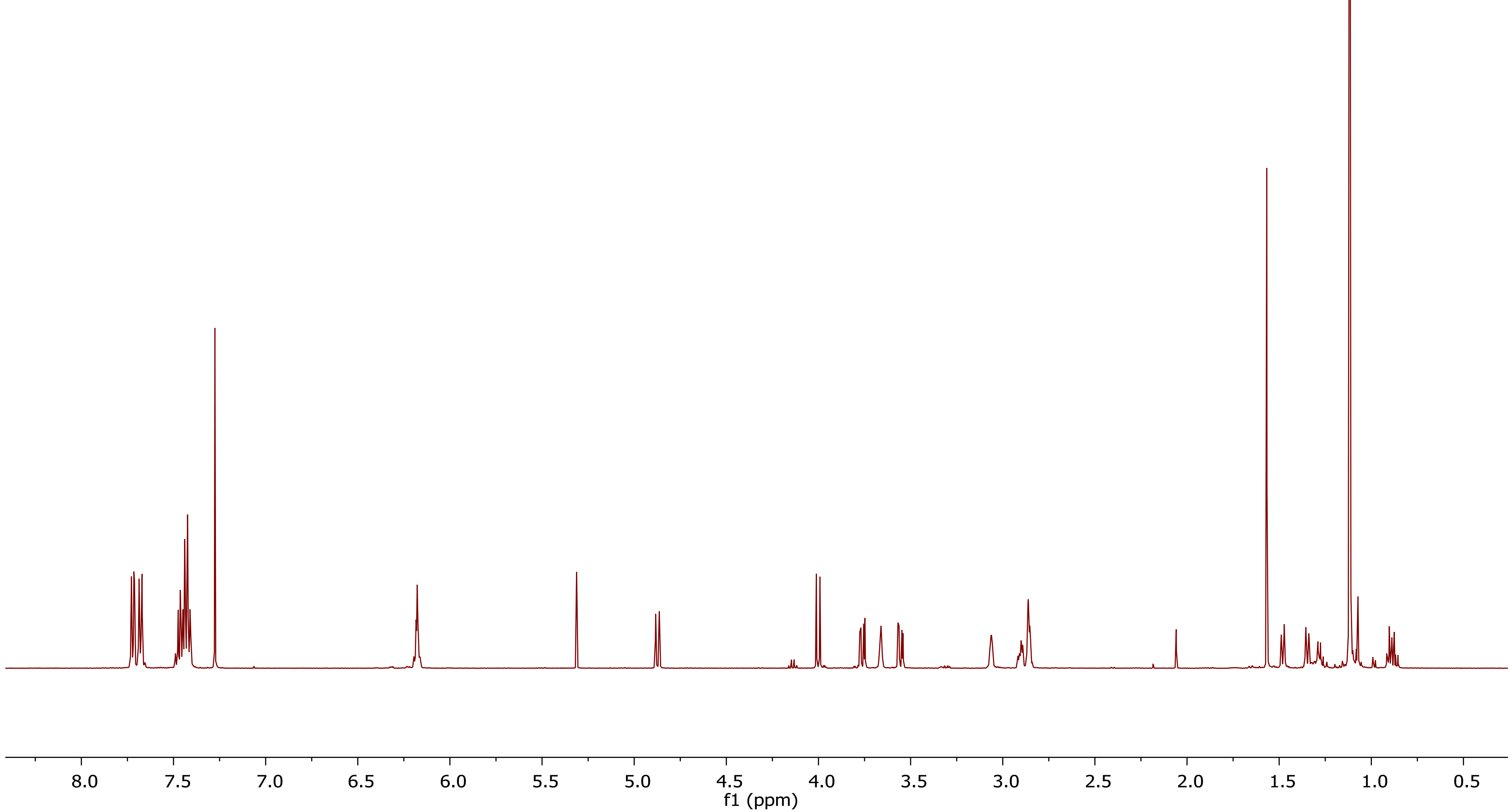

(3S,3aS,4R,7S,7aR)-3-(((tert-butyldiphenylsilyl)oxy)methyl)-1,3,3a,4,7,7a-hexahydro-4,7-methanoisobenzofuran-1-ol **79a**

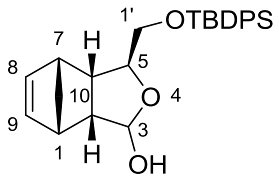

79a

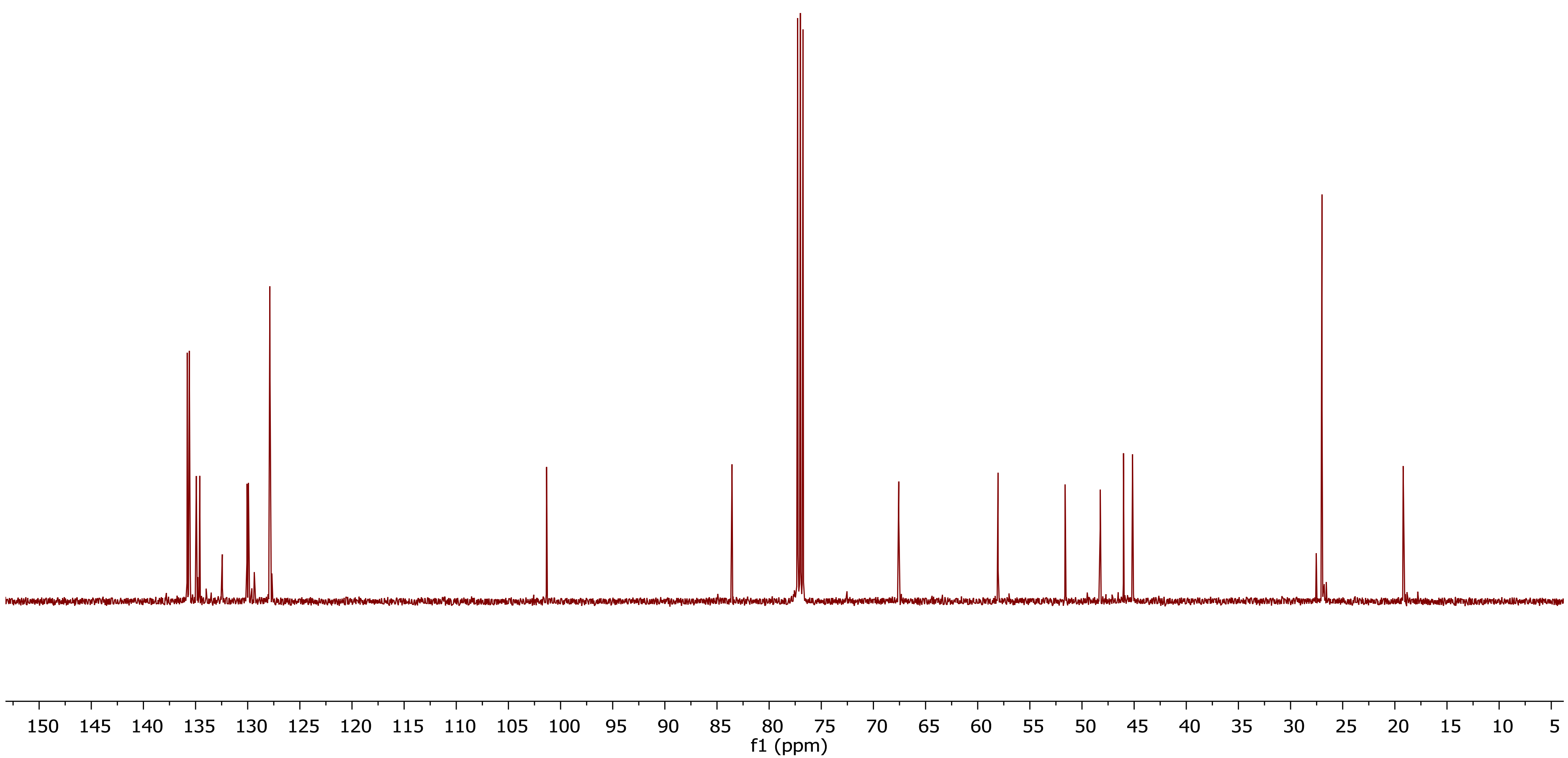

(((1S,3S,3aR,4S,7R,7aS)-3-allyl-1,3,3a,4,7,7a-hexahydro-4,7-methanoisobenzofuran-1-yl)methoxy)(tert-butyl)diphenylsilane **79b**

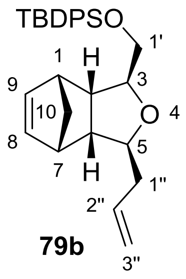

79b

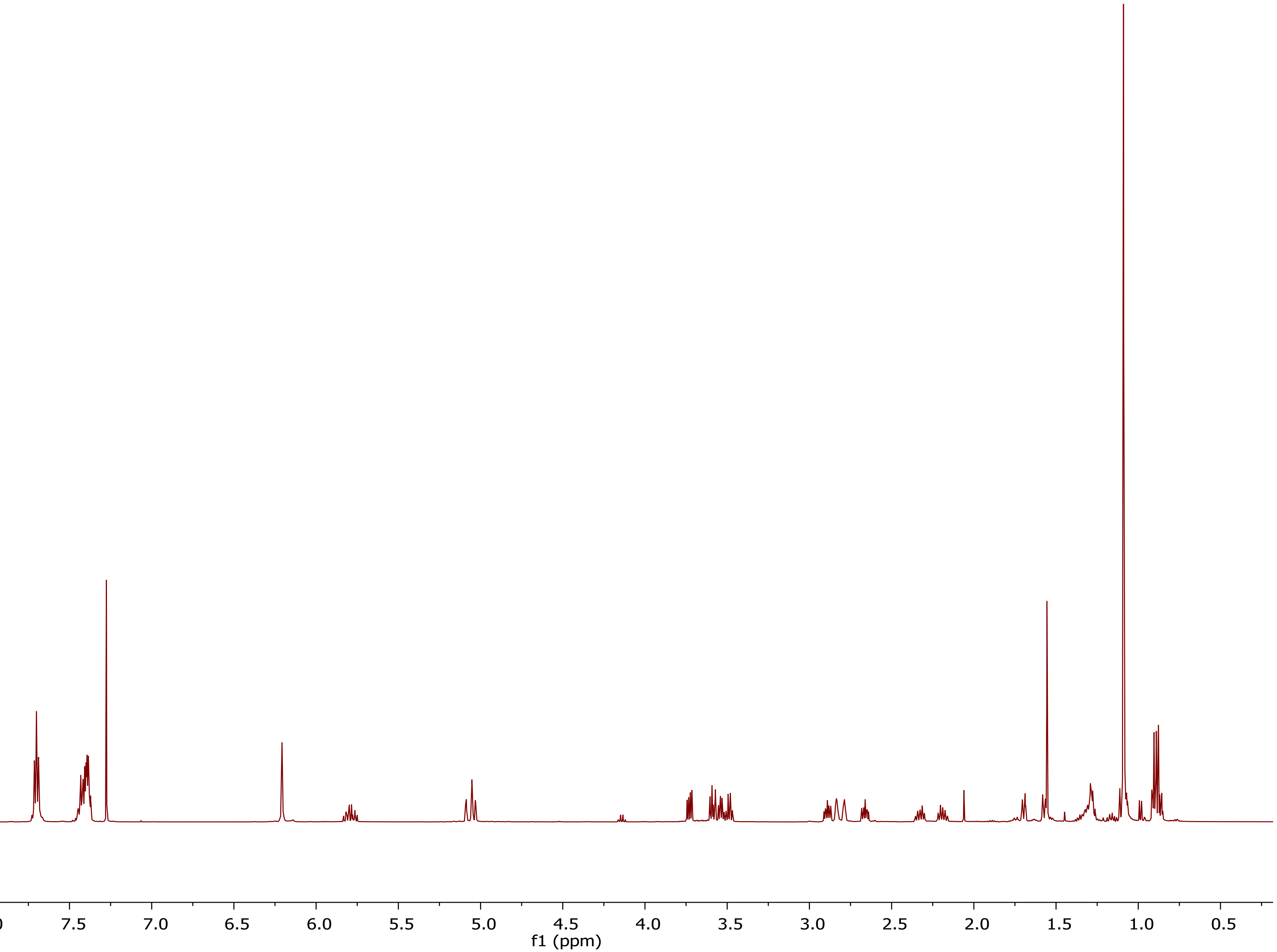

(((1S,3S,3aR,4S,7R,7aS)-3-allyl-1,3,3a,4,7,7a-hexahydro-4,7-methanoisobenzofuran-1-yl)methoxy)(tert-butyl)diphenylsilane **79b**

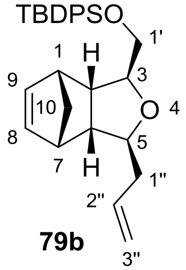

79b

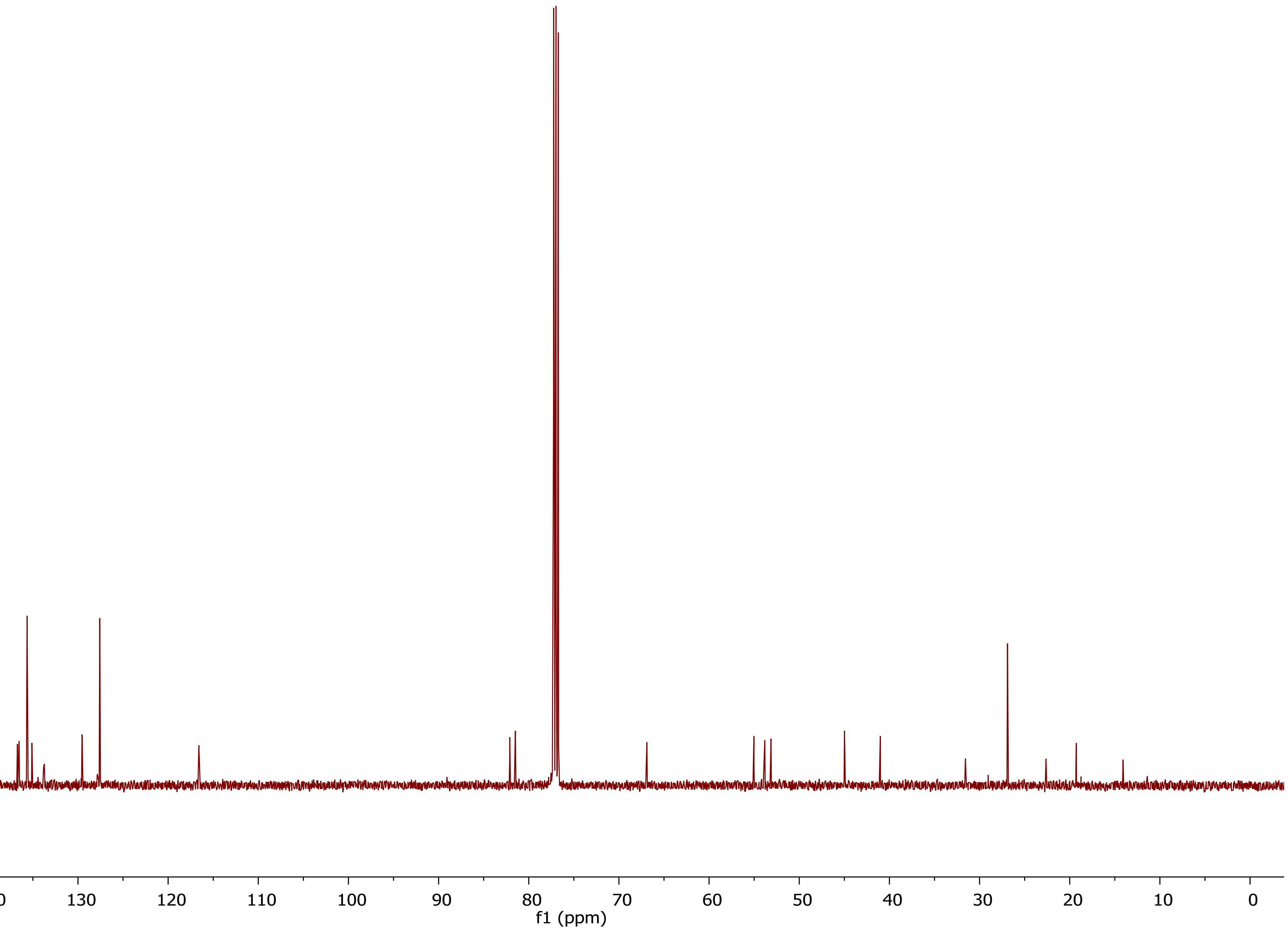

((1S,3S,3aR,4S,7R,7aS)-3-allyl-1,3,3a,4,7,7a-hexahydro-4,7-methanoisobenzofuran-1-yl)methanol **79**

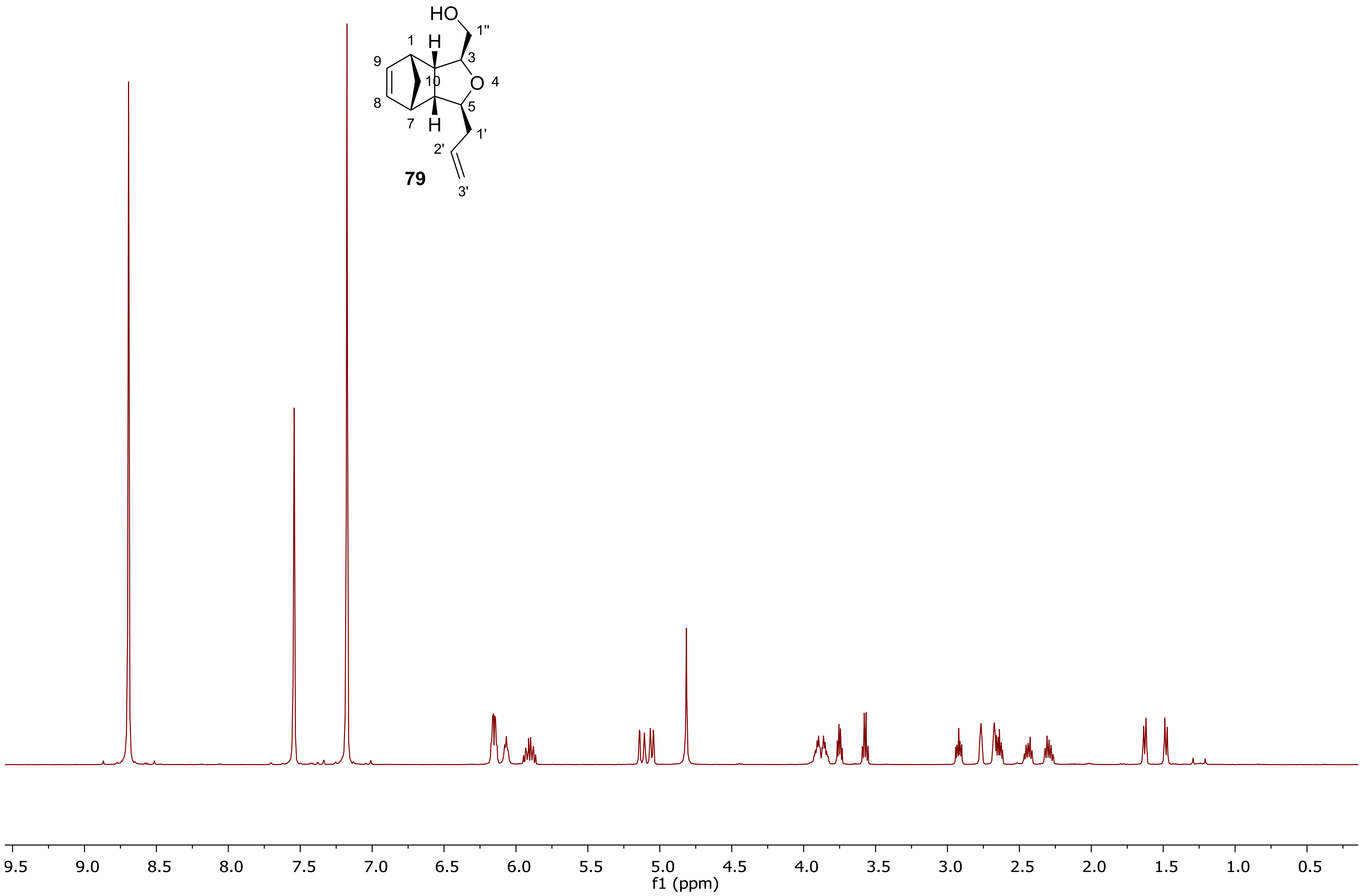

((1S,3S,3aR,4S,7R,7aS)-3-allyl-1,3,3a,4,7,7a-hexahydro-4,7-methanoisobenzofuran-1-yl)methanol **79**

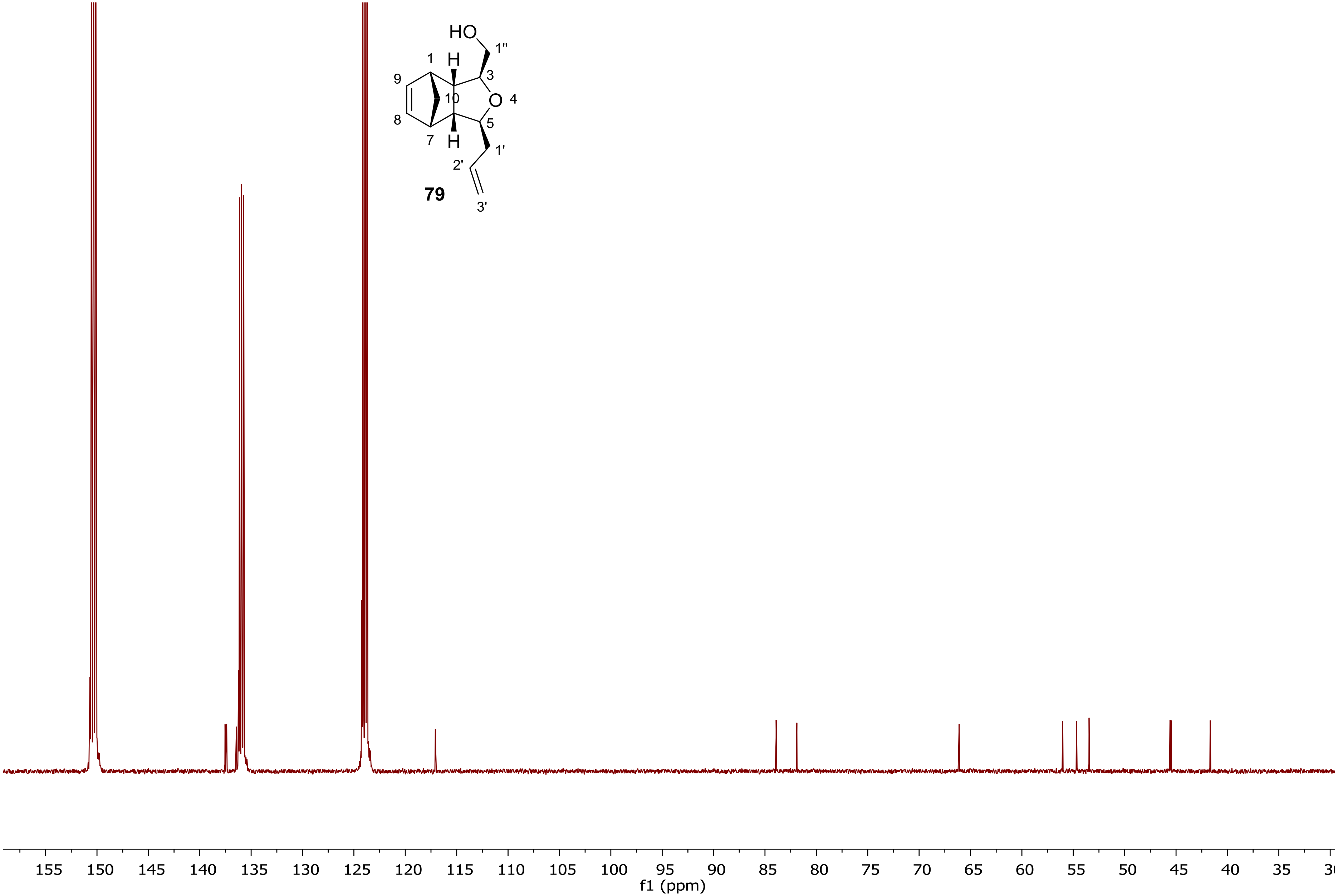

((1S,3S,3aR,4S,7R,7aS)-3-allyl-1,3,3a,4,7,7a-hexahydro-4,7-methanoisobenzofuran-1-yl)methyl but-3-enoate **80**

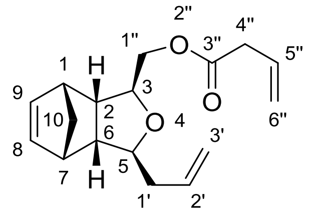

80

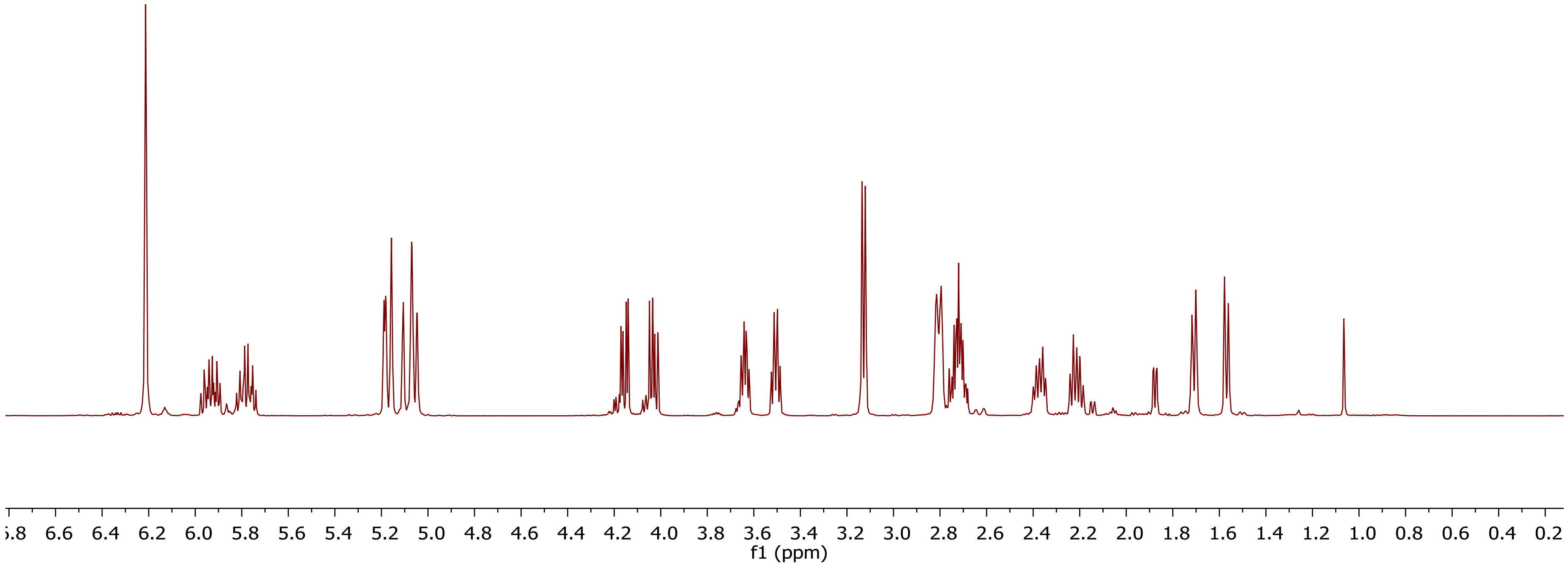

((1S,3S,3aR,4S,7R,7aS)-3-allyl-1,3,3a,4,7,7a-hexahydro-4,7-methanoisobenzofuran-1-yl)methyl but-3-enoate **80**

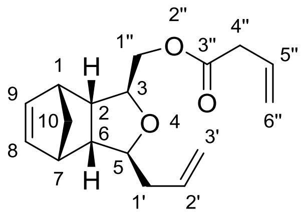

80

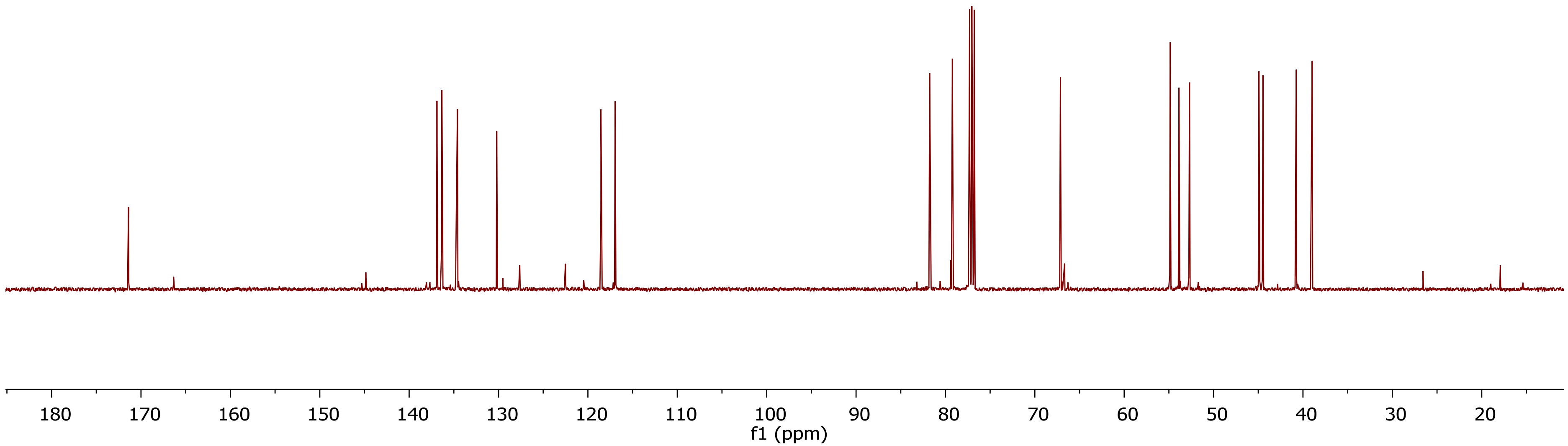

((1S,3S,3aR,4S,7R,7aS)-3-allyl-1,3,3a,4,7,7a-hexahydro-4,7-methanoisobenzofuran-1-yl)methyl pent-4-enoate **81**

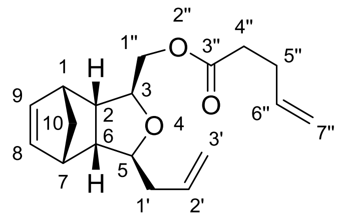

81

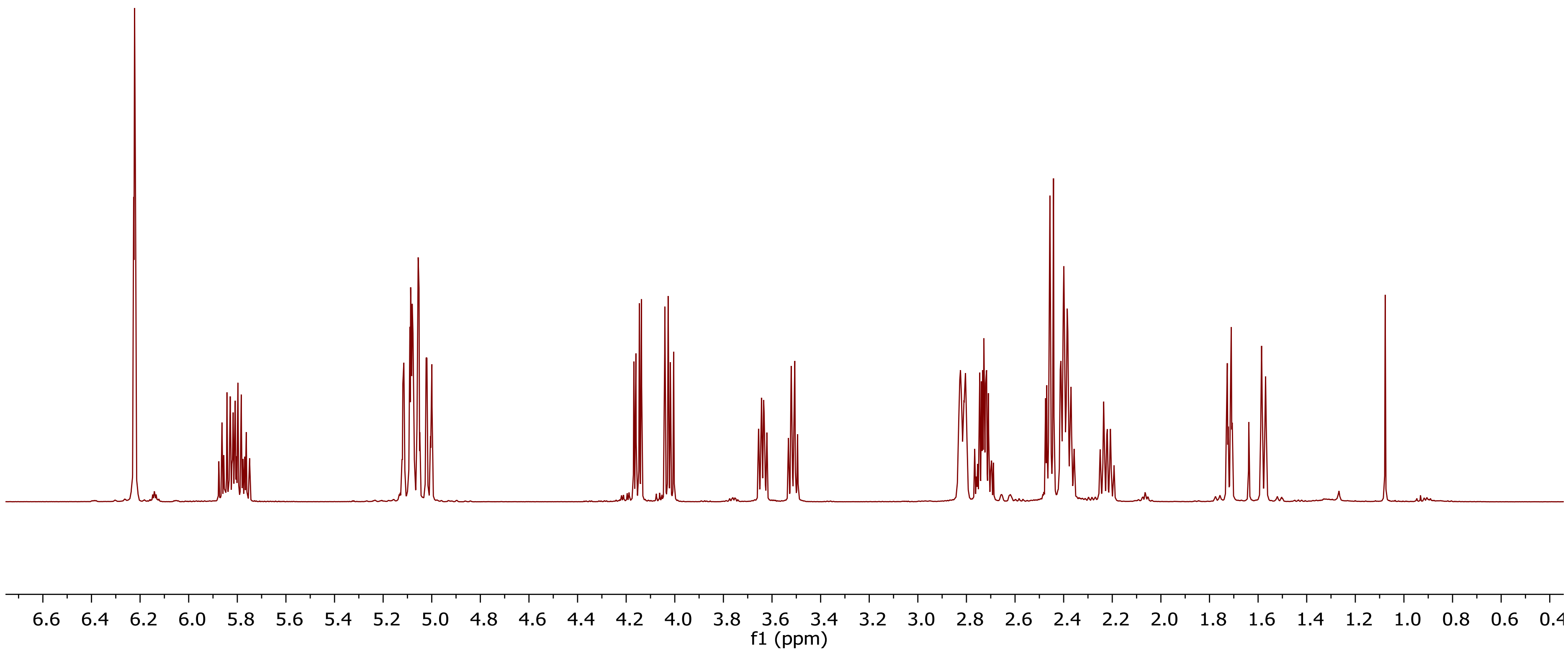

((1S,3S,3aR,4S,7R,7aS)-3-allyl-1,3,3a,4,7,7a-hexahydro-4,7-methanoisobenzofuran-1-yl)methyl pent-4-enoate **81**

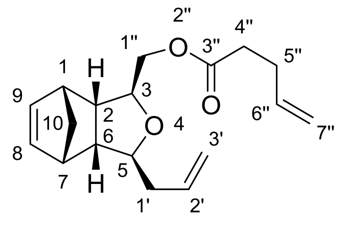

81

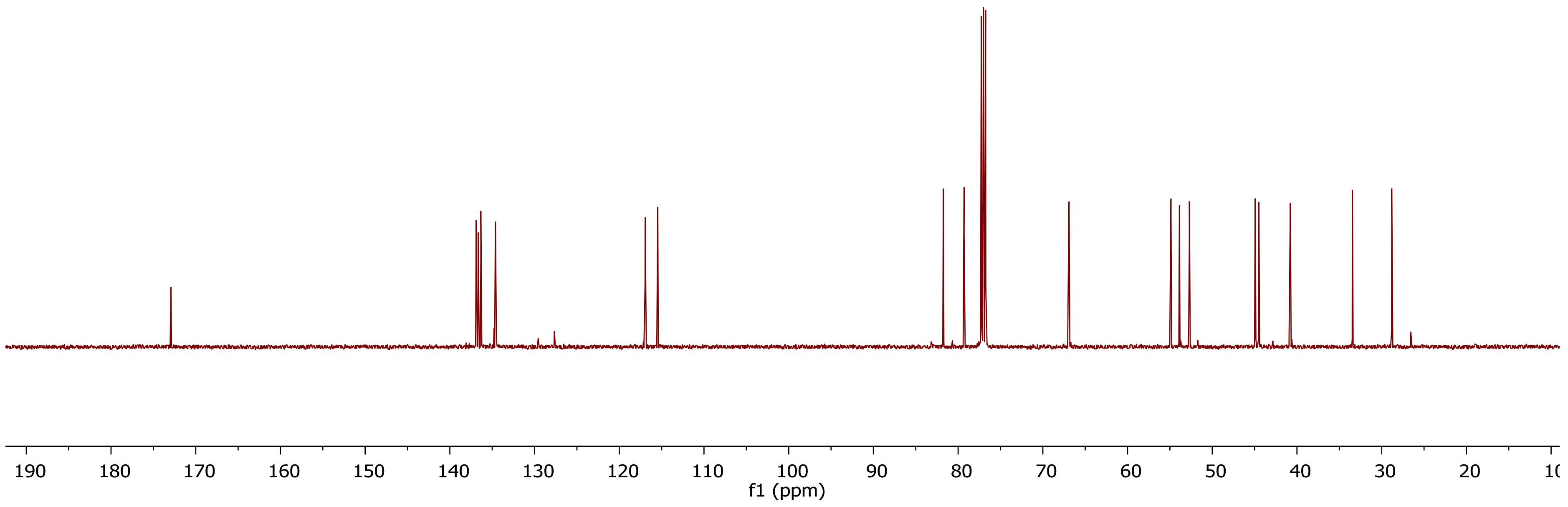

((1S,3S,3aR,4S,7R,7aS)-3-allyl-1,3,3a,4,7,7a-hexahydro-4,7-methanoisobenzofuran-1-yl)methyl hex-5-enoate **82**

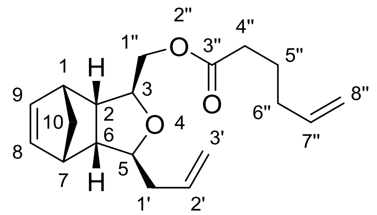

82

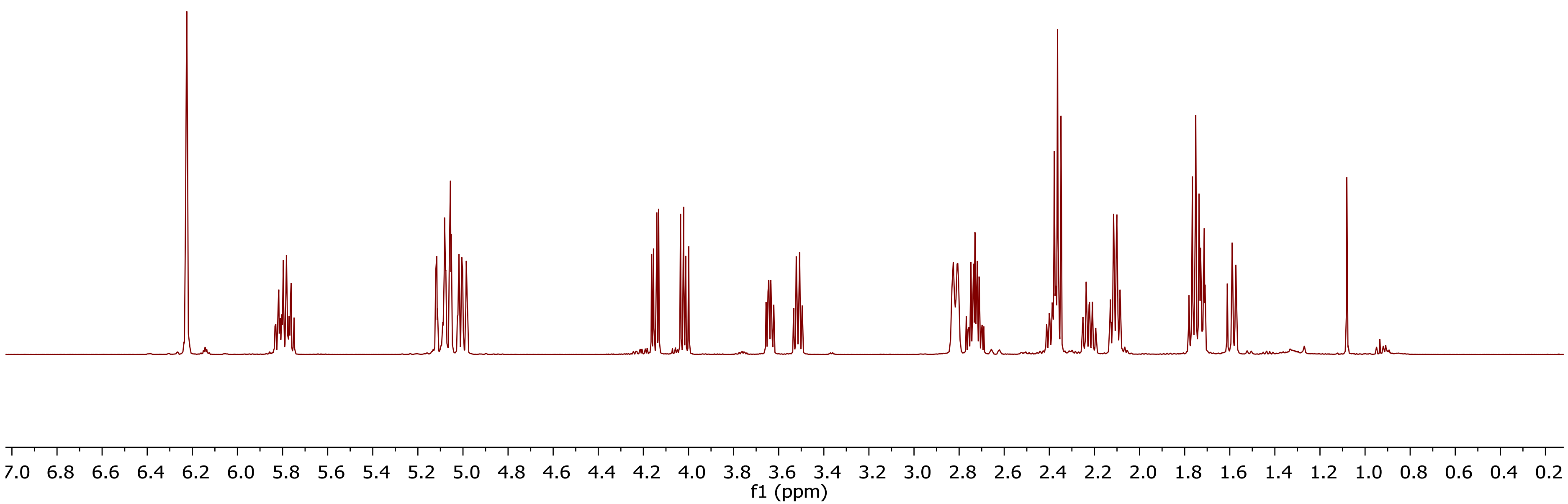

((1S,3S,3aR,4S,7R,7aS)-3-allyl-1,3,3a,4,7,7a-hexahydro-4,7-methanoisobenzofuran-1-yl)methyl hex-5-enoate **82**

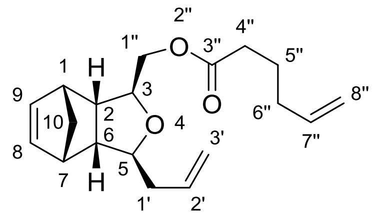

82

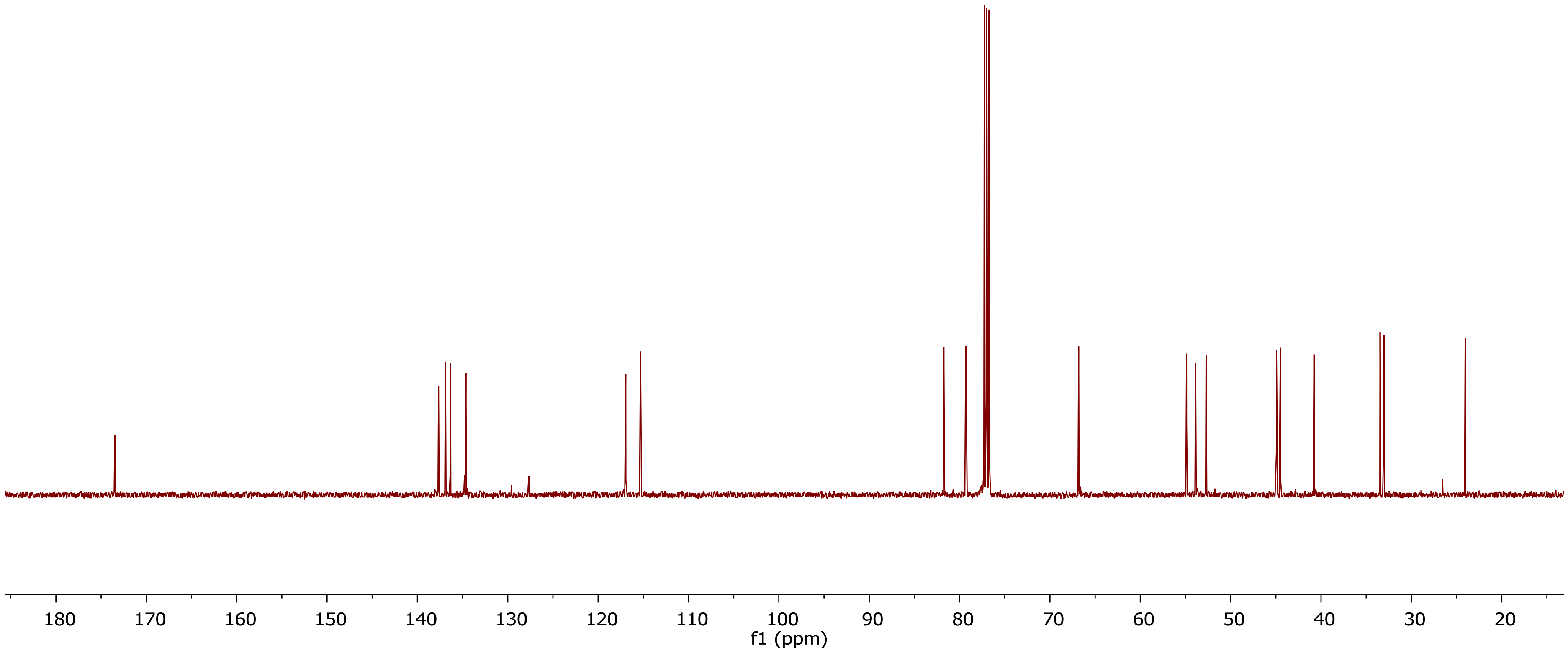

[(1S,3R,3aR,7aS)-3-(2-methyl-3-oxobutan-2-yl)-octahydro-2-benzofuran-1-yl]methyl 2-bromoacetate **89a**

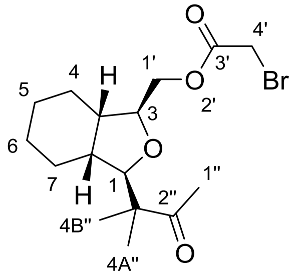

89a

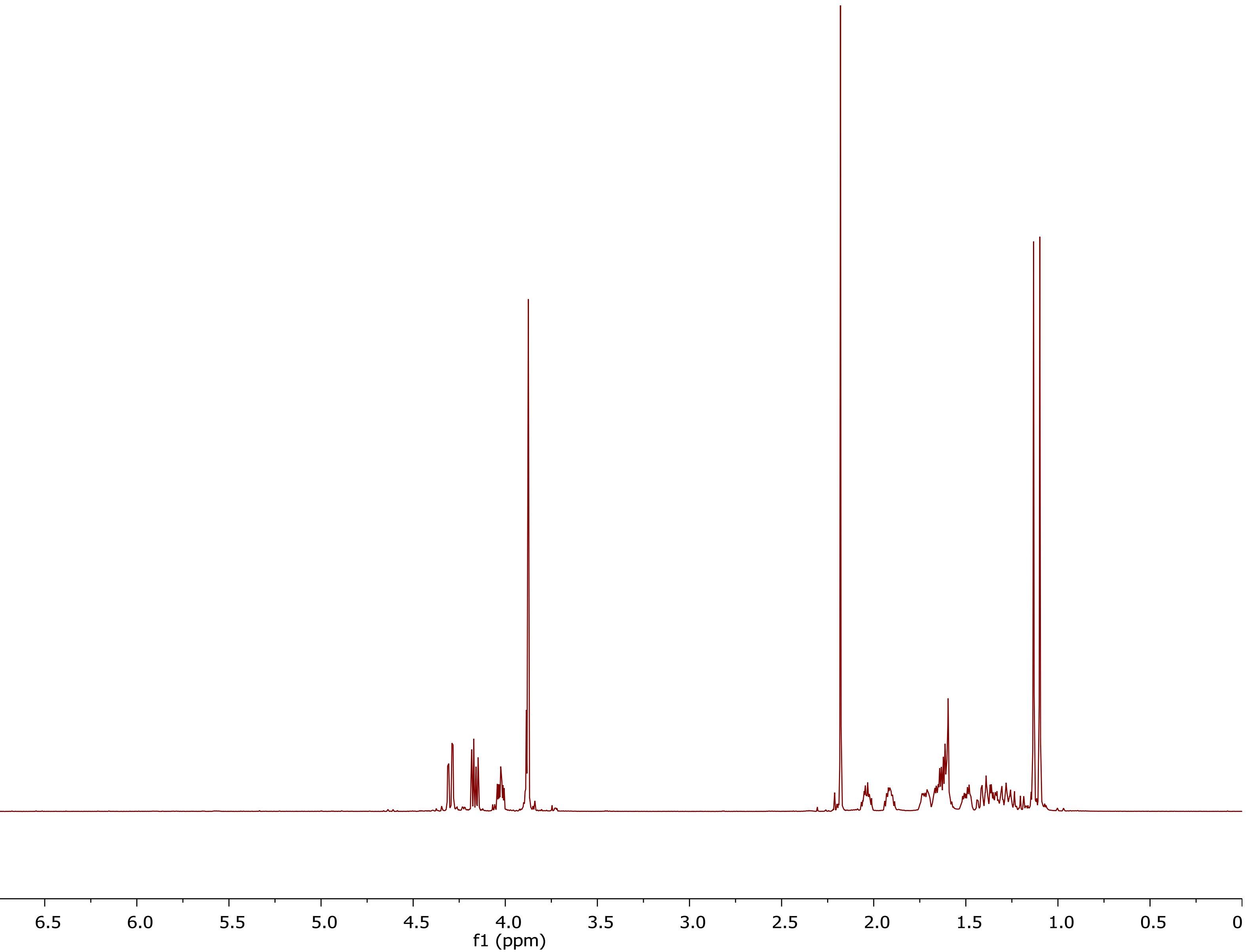

[(1S,3R,3aR,7aS)-3-(2-methyl-3-oxobutan-2-yl)-octahydro-2-benzofuran-1-yl]methyl acetate **89**

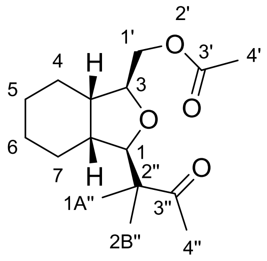

89

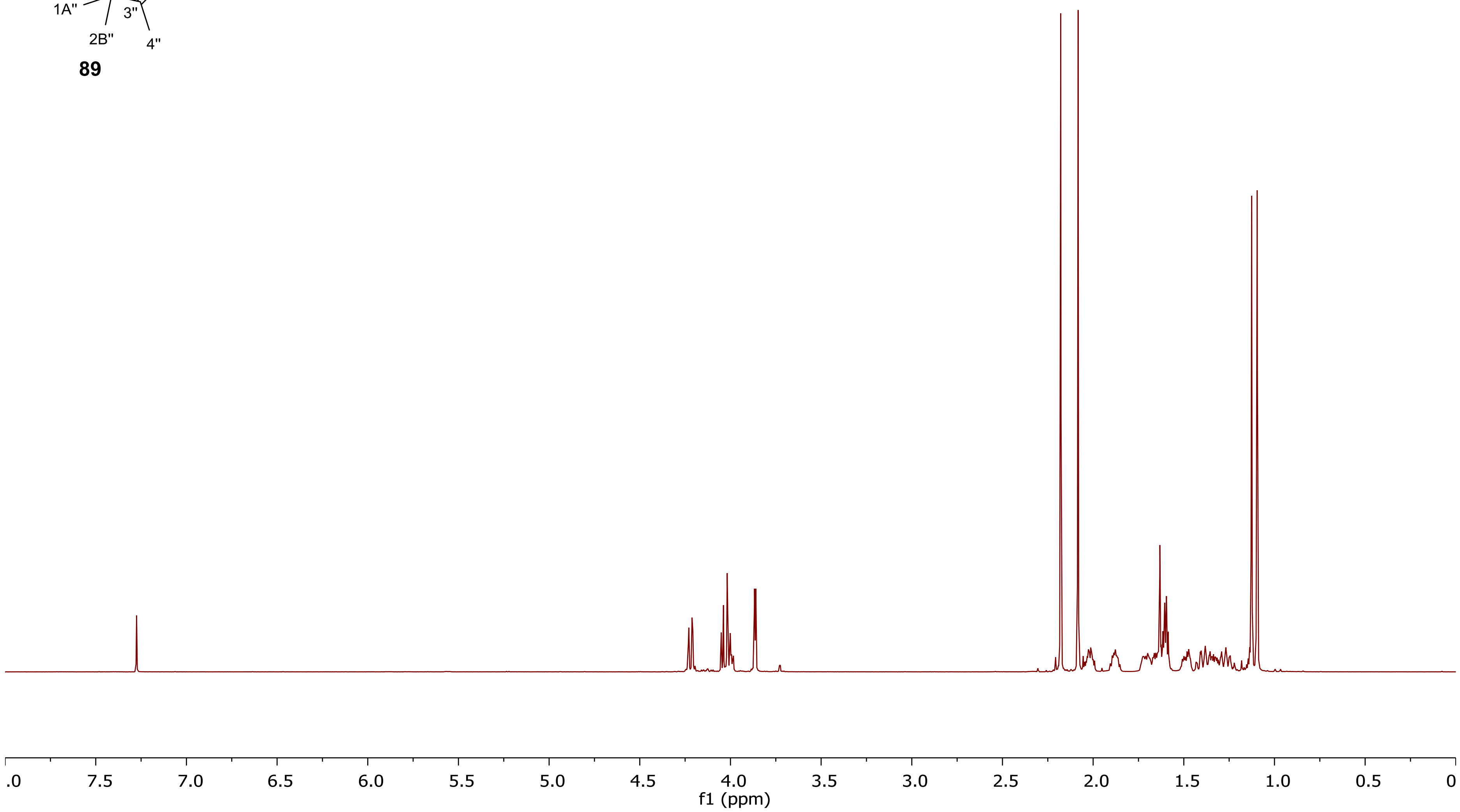

[(1S,3R,3aR,7aS)-3-(2-methyl-3-oxobutan-2-yl)-octahydro-2-benzofuran-1-yl]methyl acetate **89**

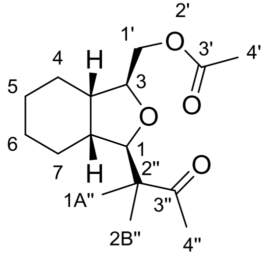

89

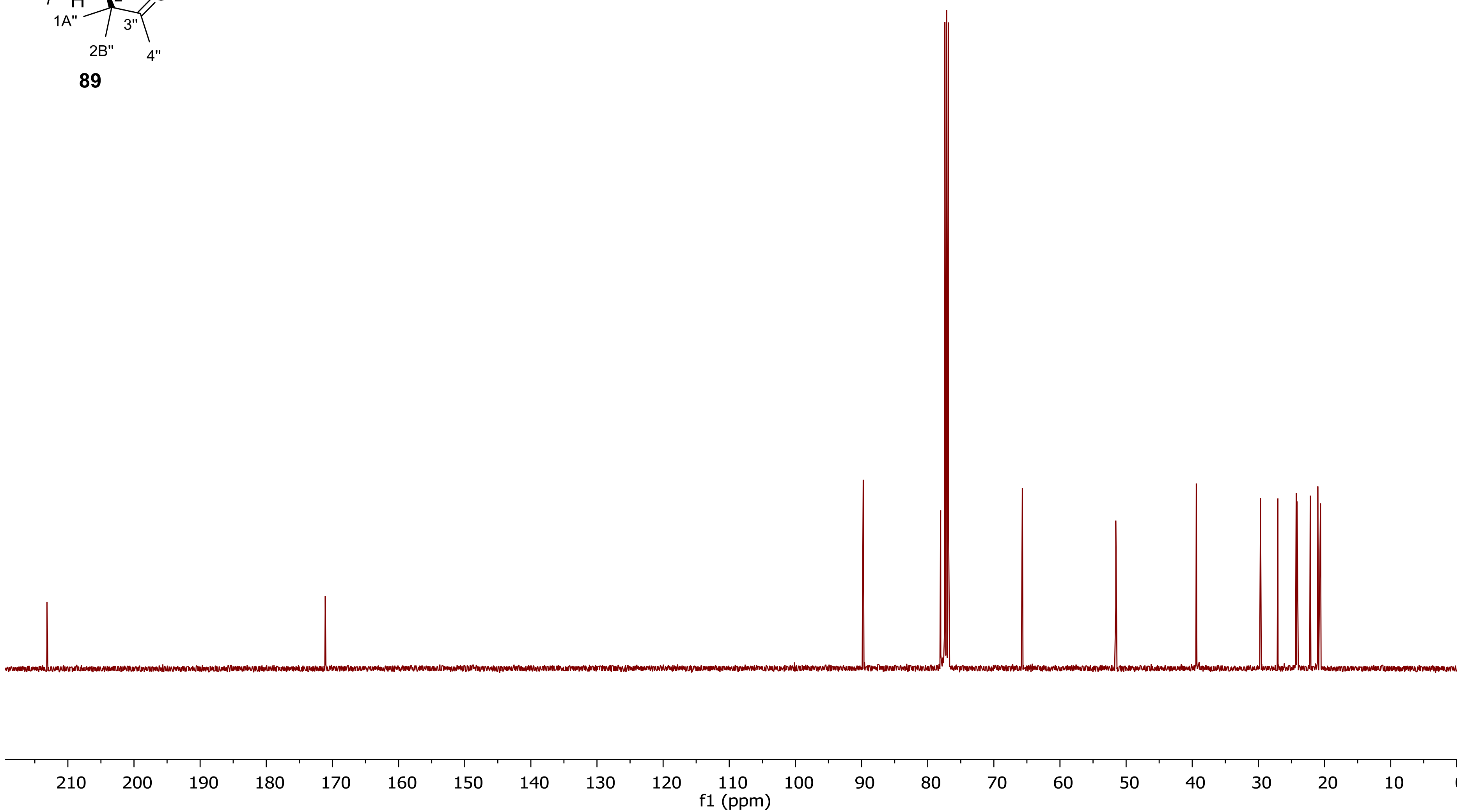

Supplement: Supplementary file 1 — Supplementary [file CHEM-22-5657-s001.pdf]
